# A Novel Immunosuppressive Effector Functions as a Bio‐Adjuvant to Enhance the Activity of *Bacillus Thuringiensis* and Chemical Insecticides

**DOI:** 10.1002/advs.78451

**Published:** 2026-11-10

**Authors:** Zhiyu Liu, Feng Chen, Liting Wan, Li Chen, Bowen Zhang, Fanling Li, Haixin Luo, Lixu Li, Alejandra Bravo, Mario Soberón, Dadong Dai, Jinshui Zheng, Ming Sun, Kai Wang, Donghai Peng

**Affiliations:** ^1^ National Key Laboratory of Agricultural Microbiology Huazhong Agricultural University Wuhan Hubei China; ^2^ Guangxi Institute of Botany Chinese Academy of Sciences Guilin China; ^3^ Instituto de Biotecnología Universidad Nacional Autónoma De México Cuernavaca Morelos México

**Keywords:** *Bacillus thuringiensis*, biopesticide, DAF‐16 / FOXO, immune suppression, Patatin‐like protein, PlcR‐PapR system, quorum sensing

## Abstract

Host innate immunity is a major barrier to microbial infection and often limits the efficacy of biological and chemical pest‐control agents. While microbial immunosuppressive effectors have been described, whether quorum‐sensing systems coordinate virulence and immune suppression during host infection remains largely unknown. We demonstrated that the PlcR‐PapR quorum‐sensing system acts as a master regulator that promotes both processes (virulence and immune suppression) to enhance *Bacillus thuringiensis* (Bt) pathogenicity. During infection, PlcR‐PapR signaling induces production of the heptapeptide PapR_7_, which activates expression of patatin‐like protein PisP, a previously uncharacterized and widely conserved protein. PisP suppresses host immunity by inhibiting nuclear translocation of the transcription factor DAF‐16, thereby attenuating the expression of antimicrobial defense genes. Consistent with this mechanism, Bt strains overexpressing *pisP* display enhanced virulence against both nematodes and insect hosts. Importantly, exogenous PisP markedly potentiates the activity of Bt Cry5Ba toxin and several commercial chemical insecticides, enabling effective pest control at reduced doses. Together, these findings reveal a quorum‐sensing‐controlled mechanism that links virulence regulation to immune suppression during Bt infection and identify PisP as a conserved Bt‐encoded immunosuppressive effector with strong potential as a bio‐adjuvant for improving the efficacy and sustainability of biological and chemical pest‐management strategies.

## Graphical abstract

A previously unrecognized link between quorum sensing and host immune suppression is revealed in *Bacillus thuringiensis*(Bt). The PlcR‐PapR system induces the novel immunosuppressive effector PisP, which inhibits DAF‐16 nuclear translocation and downstream immune defenses, thereby promoting Bt pathogenicity. PisP also enhances the activity of Cry5Ba toxin and chemical insecticides.

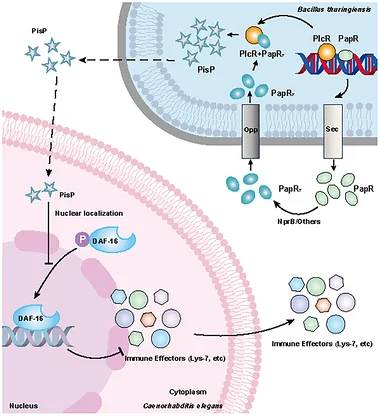

## 1 Introduction

Environmentally friendly microbial pesticides are increasingly being adopted as sustainable alternatives to conventional chemical pesticides for agricultural pest management [1]. However, their widespread application is often constrained by slower killing kinetics and lower field efficacy compared with chemical pesticides. To overcome these limitations, numerous strategies have been developed, including genetic engineering to enhance pathogen virulence, formulation improvements to increase environmental persistence and stability [2], and the use of synergistic adjuvants to boost insecticidal activity [3]. More recently, manipulation of host immunity has emerged as a promising approach for improving biological control. Pathogens have evolved sophisticated mechanisms to suppress host immune defenses during infection, and understanding these natural immunomodulatory strategies may enable the identification of effector molecules that can be exploited through microbial engineering or formulation technologies to accelerate pathogen establishment while minimizing environmental impact and non‐target effects.

The outcome of microbial infection is largely determined by the dynamic interplay between pathogen virulence and host immune defenses. To establish successful infections, pathogens from diverse taxonomic groups, viruses, bacteria, and fungi, have evolved mechanisms that evade immune recognition or actively suppress host defense responses [4, 5]. For instance, *Yersinia pseudotuberculosis* secretes a Mn^2+^‐chelating protein that lowers Mn^2+^ concentration in the cytoplasm, thereby suppressing STING‐dependent immune activation [6]. Such immunosuppressive strategies can contribute to pathogen survival, colonization, and virulence [7]. Importantly, pathogen‐derived immunosuppressive factors may also provide opportunities to improve the efficacy of biological pest‐control agents.

*Bacillus thuringiensis* (Bt) is one of the most widely used microbial pesticides and serves as a major source of biological insecticides in agricultural pest management. Its pesticidal activity is primarily attributed to parasporal‐crystal (Cry) proteins, which exhibit potent activity against a broad range of insects, nematodes, mites, and other invertebrate pests [8]. In addition to Cry toxins, Bt produces multiple virulence factors, including chitinases and metalloproteinases, involved in host colonization, tissue degradation, and nutrient acquisition [9]. Many of these factors are regulated by the PlcR‐PapR quorum‐sensing system. Quorum sensing is a cell‐density‐dependent communication mechanism in which bacteria produce, release, and detect signaling molecules to coordinate gene expression and collective behaviors, including virulence factor production, nutrient acquisition, and adaptation to changes in environmental conditions [9]. Specifically, the PlcR‐PapR quorum‐sensing (QS) system has been described as a major regulatory network that coordinates the expression of genes required for pathogenicity [10, 11, 12]. PlcR is a helix‐turn‐helix (HTH)‐type transcriptional regulator [13], whereas the *papR* gene, located immediately downstream of *plcR*, encodes a precursor peptide [14]. The PapR precursor is secreted through the Sec pathway and processed extracellularly to generate the mature signaling heptapeptide PapR_7_ [15]. Upon reaching a threshold concentration, PapR_7_ is imported into the bacterial cell and binds PlcR, promoting formation of an active PlcR‐PapR_7_ complex that recognizes target promoters and activates transcription of PlcR‐regulated genes, including multiple virulence‐associated factors [13]. During the early stages of infection, PlcR facilitates Bt colonization and multiplication within the host [16]. Thus, the PlcR‐PapR system links bacterial population density to the coordinated expression of virulence‐associated functions, promoting successful infection.

However, Bt must also overcome robust innate immune defenses mounted by its hosts. The nematode *Caenorhabditis elegans* activates multiple conserved defense pathways in response to Bt or its Cry toxins, including the p38 *mitogen‐activated protein kinase (*MAPK) cascade and the DAF‐2/DAF‐16 insulin‐like *signaling* pathway, leading to the induction of protective immune effectors [17, 18, 19, 20]. Among these pathways, DAF‐16, a Forkhead transcription factor, functions as a critical regulator of innate immunity [21]. Under basal conditions, AKT‐1 and related kinases phosphorylate DAF‐16, promoting its retention in the cytoplasm [22]. During pathogen infection, DAF‐16 can become dephosphorylated and translocate into the nucleus, where it activates the expression of numerous downstream defense‐associated genes, including immune effector genes such as *lys‐7* and *spp‐12*, which contribute to resistance against pathogens [23, 24]. Although the PlcR‐PapR system is well established as a major regulator of Bt virulence, it remains unclear whether this quorum‐sensing system also coordinates the active suppression of host immune defenses during infection.

Here, we identify a quorum‐sensing‐regulated mechanism that links virulence regulation to host immune suppression. We show that activation of the PlcR‐PapR system induces the expression of Patatin‐like protein (PisP) that functions as an immunosuppressive effector. PisP inhibits the nuclear translocation of the conserved host transcription factor DAF‐16, thereby suppressing the expression of downstream immune effectors and compromising host defenses. As a result, Bt pathogenicity is significantly enhanced. Furthermore, overexpression of the Bt *pisP* gene, or supplementation with purified PisP, markedly increases the toxicity of Cry5Ba toxin and commercial Bt formulations against both nematodes and insect hosts. Notably, PisP is widely conserved among Bt strains, suggesting that immune suppression may represent a common but previously unrecognized component of Bt pathogenicity. Together, our findings reveal that the PlcR‐PapR quorum‐sensing system coordinates both virulence factor production and host immune suppression, and identify PisP as a promising immunomodulatory bio‐adjuvant for enhancing the efficacy and sustainability of both biological and chemical pest‐control strategies.

## 2 Results

### 2.1 Suppression of Host Stress Responses During Bt Infection

During infection, Bt encounters robust host innate immune defenses [9, 25]. To investigate whether Bt actively suppresses these responses in *C. elegans*, we compared the transcriptional profiles of nematodes exposed to the nematicidal Bt strain BMB171 expressing Cry5Ba (BMB171/Cry5Ba) with those of nematodes exposed to a non‐pathogenic *Escherichia coli* strain BL21 expressing the same toxin (BL21/Cry5Ba). This experimental design allowed us to distinguish host responses elicited by Cry5Ba toxin from those effects specifically associated with Bt virulence factors.

Our transcriptomic (RNA‐seq) analysis identified 1,869 differentially expressed genes (DEGs) in *C. elegans* nematodes exposed to BMB171/Cry5Ba compared with BL21/Cry5Ba, including 893 upregulated and 976 downregulated genes (Figure 1A, Table S1). Gene Ontology (GO) analysis revealed that upregulated genes were mainly associated with developmental processes, including autophagy and defense responses, whereas downregulated genes were enriched in metabolic and enzymatic functions, including metallocarboxypeptidase, oxidoreductase, and acyl‐CoA oxidase functions (Figure S1). To discriminate between Cry5Ba‐induced and Bt‐specific transcriptional responses, we compared these RNA‐seq data with our previously published microarray dataset of *C. elegans* N2 nematode exposed to BL21/Cry5Ba toxin compared with BL21 [26]. This comparison identified a common set of 128 upregulated and 113 downregulated genes shared between treatments, representing the canonical host response to Cry5Ba intoxication (Figure 1A). In contrast, genes uniquely regulated in the BMB171/Cry5Ba treatment likely reflected Bt‐specific effects on the host. Functional classification of these Bt‐specific genes using Wormcat [27] revealed significant enrichment of extracellular matrix components, stress regulation, and signaling pathways among the uniquely upregulated genes (Table S1). Notably, the three most significantly enriched categories among uniquely downregulated genes were all associated with stress response categories (Figure 1B), suggesting that Bt actively represses host defense mechanisms during infection. To experimentally validate these findings, we performed qPCR analysis of 14 randomly selected DEGs between the BMB171/Cry5Ba and BL21/Cry5Ba treatments. Among the 14 validated genes, four have been previously characterized as DAF‐16‐regulated immune defense genes [23, 24, 28], whereas the remaining ten genes, which are not associated with the DAF‐16 pathway, were also significantly downregulated during Bt infection. (Figure 1C). These results confirm that their repression is specifically associated with Bt infection in comparison to Cry5Ba intoxication alone.

**FIGURE 1:**
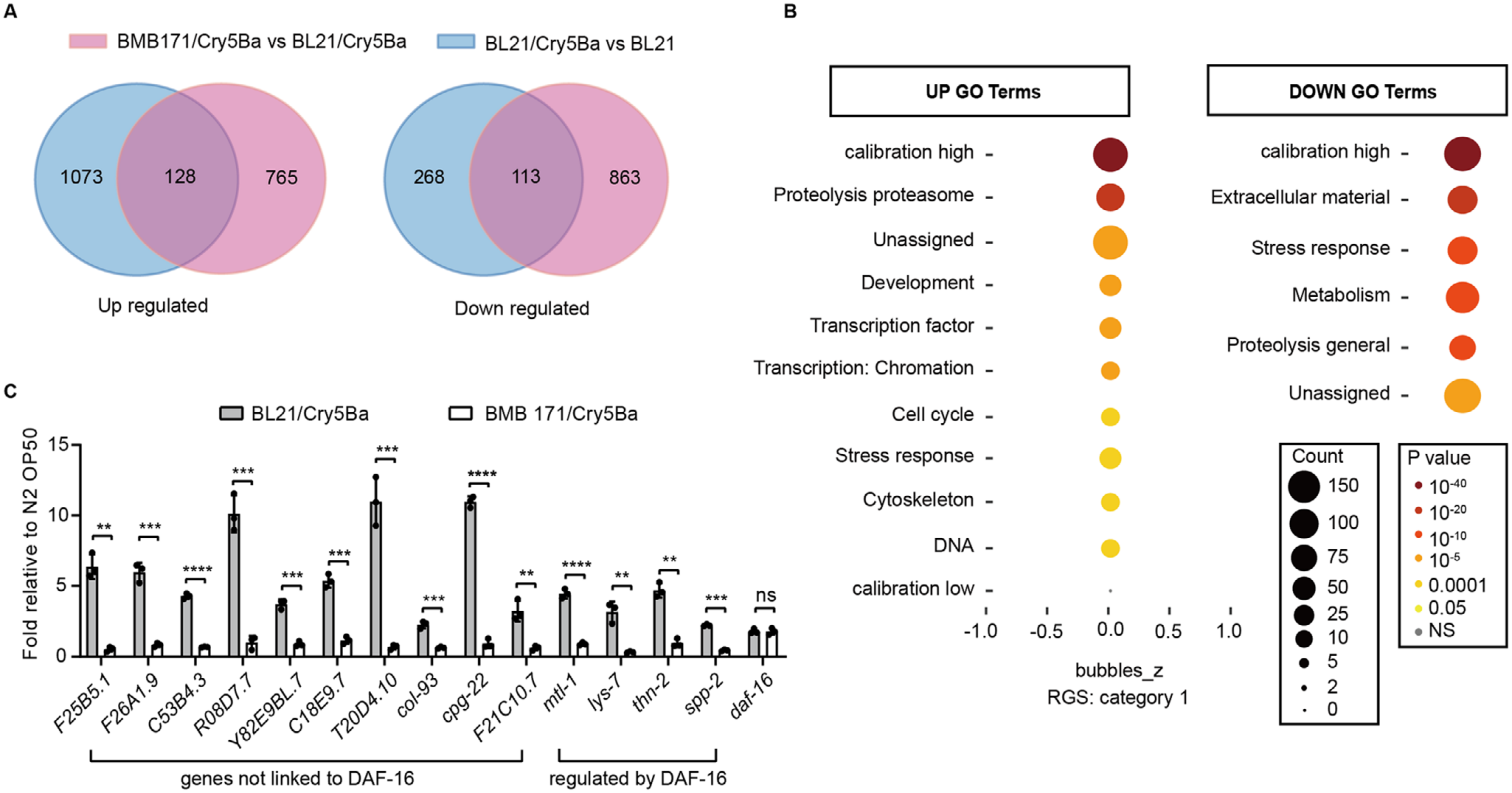
Nematicidal Bt infection suppresses innate immune responses in *C. elegans*. (A) Venn diagram showing the overlap between differentially expressed genes identified by RNA‐Seq analysis of worms exposed to BMB171/Cry5Ba (Bt/toxin) versus BL21/Cry5Ba (Ec/toxin) (pink color) and those identified by microarray analysis of worms exposed to BL21/Cry5Ba versus BL21 (blue color). (B) Gene ontology enrichment analysis (GO‐WormCat enrichment analysis, Table S1) of up‐regulated and down‐regulated genes in response to BMB171/Cry5Ba, performed using WormCat. Functional categories associated with extracellular components, stress response, and metabolic processes were significantly suppressed in Bt‐infected nematodes. (C) qPCR validation of representative genes in worms exposed to BL21/Cry5Ba or BMB171/Cry5Ba. Data are presented as mean ± SD from three independent biological replicates. Statistical significance was determined using unpaired t‐tests. ^**^ *p* < 0.01, ^***^ *p* < 0.001, ^****^ *p* < 0.0001; ns, not significant.

Collectively, these results demonstrated that Bt not only induces toxin‐dependent host responses, but also actively suppresses stress‐ and defense‐related gene expression. This transcriptional signature is consistent with the presence of specific Bt‐derived immunosuppressive factors that counteract host defenses and promote successful infection.

### 2.2 BMB171/Cry5Ba Suppresses *C. elegans* Innate Immunity by Blocking DAF‐16 Nuclear Translocation

Following pathogen recognition, *C. elegans* activates multiple conserved innate immune pathways, including the p38/MAPK, unfolded protein response (UPR), transforming growth factor‐beta (TGF‐β), and DAF‐2/DAF‐16 signaling pathways [29]. Previous studies have shown that exposure to Cry5Ba toxin alone activates both the p38/MAPK and insulin DAF‐2/DAF‐16 pathways, resulting in the induction of protective immune responses [21]. To identify the immune pathway targeted during Bt infection, we further analyzed the stress‐ and defense‐related genes that were selectively repressed in nematodes exposed to BMB171/Cry5Ba (Bt/toxin). Strikingly, transcriptomic analysis identified the DAF‐2/DAF‐16 signaling pathway as the principal target of Bt‐mediated immune suppression. Among the 113 genes specifically downregulated in the BMB171/Cry5Ba treatment, 72 are known downstream targets of DAF‐2/DAF‐16 (Table S2), suggesting that this conserved immune regulatory pathway is broadly inhibited during Bt infection. Although the DAF‐2/DAF‐16 signaling pathway is best known for its role in lifespan regulation, it also functions as a central component of innate immunity, contributing to host resistance against diverse bacterial pathogens, including *Pseudomonas aeruginosa* and *Staphylococcus aureus* [20]. Activation of the FOXO transcription factor DAF‐16 requires its dephosphorylation and its translocation from the cytoplasm to the nucleus, where it induces the expression of immune and stress‐response genes [21]. We therefore investigated whether Bt infection (BMB171/Cry5Ba treatment) interferes with DAF‐16 nuclear localization. To directly visualize DAF‐16 localization, we used the transgenic N2‐TJ356 worms expressing a functional DAF‐16::GFP fusion protein. Exposure to Ec/toxin (BL21/Cry5Ba) induced robust nuclear accumulation of DAF‐16::GFP, consistent with activation of the host defense response. In contrast, nematodes exposed to Bt/toxin (BMB171/Cry5Ba) exhibited a marked reduction in nuclear localization of DAF‐16::GFP (Figure 2A,B), indicating that Bt actively suppresses DAF‐16 activation. This observation was further confirmed by western blot analysis of isolated nuclear and cytoplasmic fractions from the treated worms, which showed a significant decrease in nuclear DAF‐16::GFP levels in worms treated with BMB171/Cry5Ba compared to those treated with Cry5Ba alone (Figure 2C,D).

**FIGURE 2:**
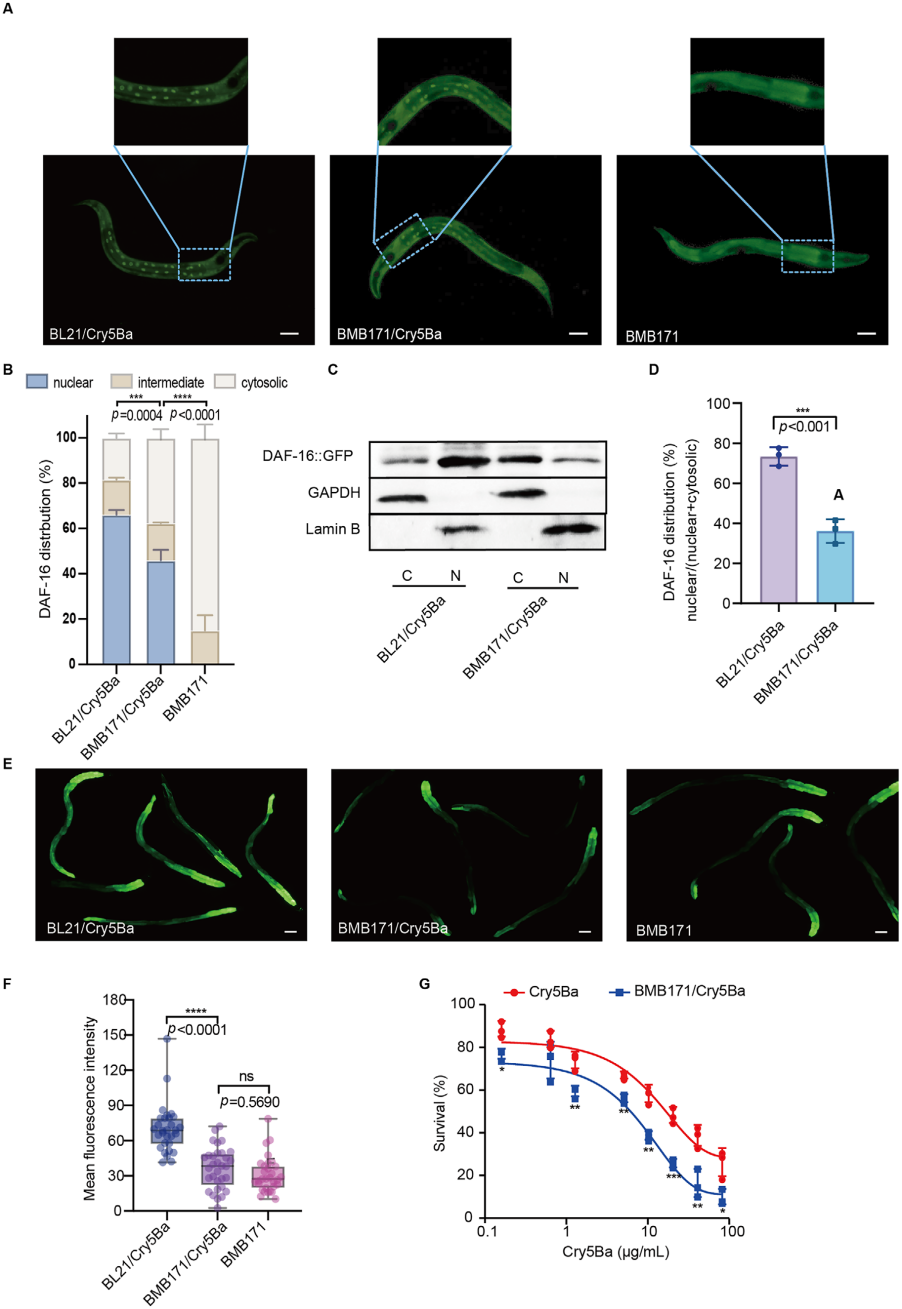
Bt treatment suppresses innate immune responses in *C. elegans* by inhibiting DAF‐16 nuclear translocation. (A–D) Bt treatment inhibits DAF‐16 nuclear translocation. (A) Representative fluorescence images of DAF‐16::GFP localization in adult TJ356 (*daf‐16*::*gfp*) worms under the indicated treatments. Exposure to BL21/Cry5Ba (Ec/toxin) induces nuclear accumulation of DAF‐16::GFP, whereas BMB171/Cry5Ba (Bt/toxin) markedly reduces DAF‐16 nuclear localization. BMB171 alone does not induce DAF‐16::GFP nuclear translocation. Scale bar = 100 µm. (B) Quantification of DAF‐16::GFP localization, classified as nuclear, intermediate, or cytosolic. Data represent three independent experiments, each analyzing > 30 worms per condition. (C) Biochemical confirmation of nuclear and cytoplasmic localization by western blot analysis of DAF‐16 protein in nuclear and cytosolic fractions after 4 h treatments. Proteins were analyzed by SDS‐PAGE, and DAF‐16::GFP was detected using an anti‐GFP antibody. Lamin B1 and GAPDH served as nuclear or cytosolic markers, respectively. A representative blot from three independent experiments is shown. (D) Quantification of DAF‐16::GFP levels in nuclear and cytosolic fractions, normalized to Lamin B1 (nuclear) and GAPDH (cytosolic), respectively. Data are presented as mean ± SD from three independent experiments. (E, F) Bt treatment suppresses downstream DAF‐16‐dependent immune response. (E) Representative fluorescence images of the antimicrobial peptide reporter *lys‐7*::*gfp* worms following the indicated treatments. Scale bar = 100 µm. (F) Quantification of Lys‐7::GFP fluorescence intensity after 4 h exposure. Data are presented as mean ± SD from three independent experiments, each analyzing > 20 worms per condition. (G) Bt treatment increases host susceptibility to Cry5Ba. Dose‐response mortality assays comparing the susceptibility of wild‐type N2 worms to purified Cry5Ba toxin and BMB171/Cry5Ba. Data are presented as means ± SD from three independent experiments, each performed in triplicate with > 30 worms per condition. Statistical significance was determined by an unpaired t‐test. ^*^ *p* < 0.05, ^**^ *p* < 0.01, ^***^ *p* < 0.001, ^****^ *p* < 0.0001; ns, not significant.

We next examined whether inhibition of DAF‐16 nuclear translocation affected the expression of its downstream immune effectors. In N2 worms, the transcription levels of four previously characterized DAF‐16‐regulated immune defense genes, *mtl‐1*, *lys‐7*, *thn‐2*, and *spp‐2*, were significantly lower following exposure to BMB171/Cry5Ba compared to BL21/Cry5Ba (Figure 1C). In contrast, in the *daf‐16* deletion mutant, the transcription levels of these genes did not differ significantly between the BMB171/Cry5Ba and BL21/Cry5Ba treatments (Figure S2), further supporting their regulation by DAF‐16. Consistent with these results, analysis of *lys‐7*::*gfp* reporter worms showed that BL21/Cry5Ba exposure induced *lys‐7* expression, whereas BMB171/Cry5Ba exposure markedly reduced GFP fluorescence (Figure 2E,F). Together, these findings demonstrate that suppression of DAF‐16 nuclear localization is associated with reduced activation of downstream immune defense genes.

Consistent with the inhibition of host immunity, dose response assays revealed that BMB171/Cry5Ba was significantly more pathogenic than Cry5Ba alone, exhibiting an LC_50_ value of 3.374 µg/mL, (95% confidence interval: 0.692–5.841 µg/mL), compared with LC_50_ values of 25.679 µg/mL (95% confidence interval: 19.704–34.427 µg/mL) for Cry5Ba alone (Figure 2G, Table S3).

Together, these results demonstrate that Bt suppresses host innate immunity by preventing DAF‐16 nuclear translocation, thereby blocking the expression of downstream defense genes and enhancing pathogen virulence. These findings identify inhibition of DAF‐16 activation as a central mechanism by which Bt suppresses host immunity during infection.

### 2.3 The Global Regulator PlcR is Essential for Bt‐mediated Suppression of Host Innate Immunity

Bacterial pathogens commonly suppress host immunity through secreted effectors that target key specific defense pathways [30, 31, 32]. Having established that Bt actively inhibits DAF‐16 nuclear translocation during infection, we next sought to identify the bacterial regulatory network responsible for this immunosuppressive activity. In *Bacillus* species, the global regulators PlcR, NprR, and LuxS control quorum‐sensing and coordinate the expression of numerous extracellular virulence factors [33, 34, 35]. We therefore hypothesized that one or more of these regulators may contribute to Bt‐mediated immune suppression. To test this possibility, we generated in‐frame deletion mutants of *plcR*, *nprR*, and *luxS* (Δ*plcR*, Δ*nprR*, and Δ*luxS*) in the acrystalliferous Bt strain BMB171 and evaluated their virulence in the presence of Cry5Ba. Although all three regulators contributed to pathogenicity, deletion of (Δ*plcR*) caused the most pronounced virulence attenuation. Specifically, the LC_50_ of the BMB171Δ*plcR*/Cry5Ba was 7.981 µg/mL (95% confidence interval: 6.31–9.795 µg/mL), compared with 3.374 µg/mL (95% confidence interval: 0.692–5.841 µg/mL) for wild‐type BMB171/Cry5Ba (Figure 3A, Table S3). In contrast, deletion of *nprR* and *luxS* (Δ*nprR* and Δ*luxS)* produced substantially smaller effects on virulence, indicating that PlcR plays a dominant role in Bt pathogenicity. To determine whether the reduced virulence of the Δ*plcR* mutant was linked to impaired suppression of DAF‐16‐mediated immunity, we compared its toxicity in the wild‐type N2 and *daf‐16*(mu86) null mutant worms. Notably, the virulence difference between wild‐type Bt and the Δ*plcR* mutant was largely eliminated in the *daf‐16* mutant worms (Figure 3B), demonstrating that suppression of DAF‐16‐dependent immunity is a major contributor to PlcR‐dependent virulence.

**FIGURE 3:**
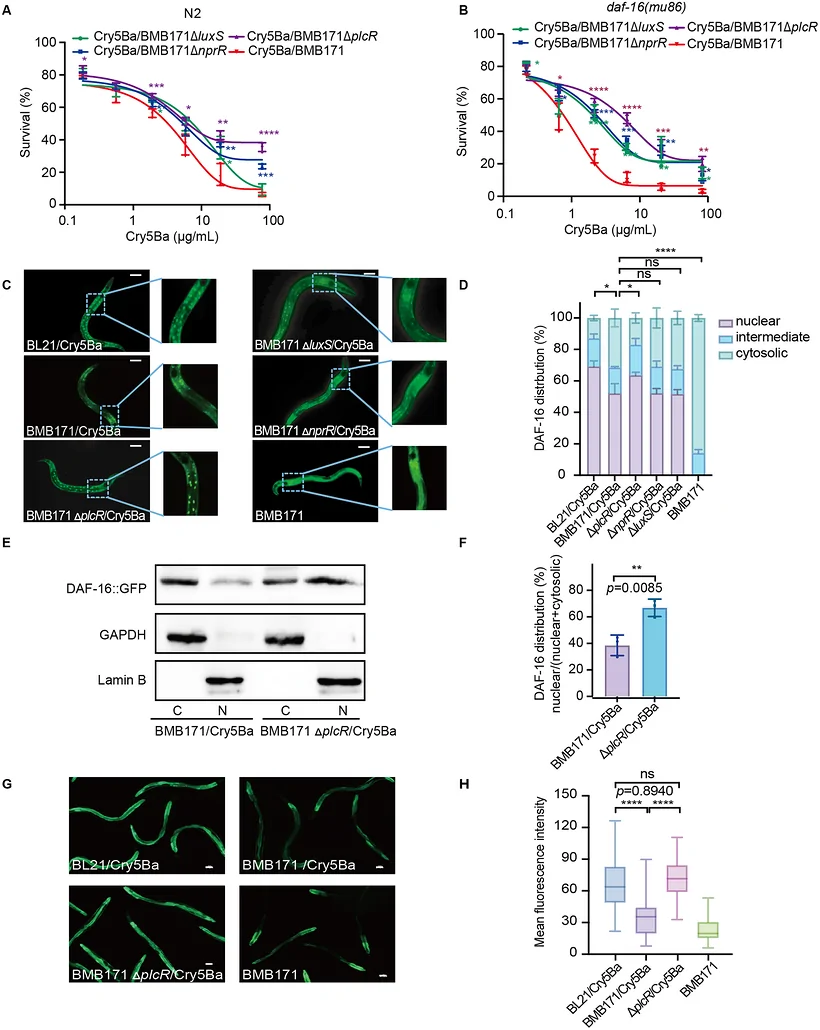
PlcR is required for Bt‐mediated suppression of innate immune responses in *C. elegans*. (A, B) Dose‐response mortality assays comparing the susceptibility of wild‐type N2 (A) and *daf‐16(mu86)* mutant worms (B) to spores of BMB171△*plcR*, BMB171△*nprR*, BMB171△*luxS*, and the parental BMB171 strain, each administered in combination with equivalent amounts of Cry5Ba toxin. Data are presented as mean ± SD from three independent experiments, each performed in triplicate with > 30 worms per condition. (C) Representative fluorescence images showing DAF‐16::GFP localization in adult *TJ356 (daf‐16::gfp)* worms following the indicated treatment. BL21/Cry5Ba and BMB171△*plcR*/Cry5Ba induce nuclear accumulation of DAF‐16::GFP, whereas BMB171/Cry5Ba, BMB171△*nprR* /Cry5Ba and BMB171△*luxS* /Cry5Ba inhibit DAF‐16::GFP nuclear localization. BMB171 alone does not induce DAF‐16::GFP nuclear translocation. Scale bar = 100 µm. (D) Quantification of DAF‐16::GFP localization in *TJ356*(*daf‐16::gfp*) worms, classified as nuclear, intermediate, or cytosolic. The percentage of worms exhibiting nuclear DAF‐16::GFP localization was calculated as the number of worms classified as showing nuclear localization divided by the total number of worms scored. Data represent three independent experiments, each analyzing > 30 worms per condition. (E) Immuno‐blot analysis of DAF‐16::GFP in nuclear and cytosolic fractions isolated from N2‐*TJ356*(*daf‐16::gfp*) worms after 4 h exposure to the indicated treatments. Proteins were analyzed by SDS‐PAGE, and DAF‐16 was detected using a primary anti‐GFP antibody. Lamin B1 and GAPDH were used as nuclear and cytosolic markers, respectively. A representative blot from three independent experiments is shown. (F) Quantification of DAF‐16::GFP levels in nuclear and cytosolic fractions, normalized to Lamin B1 (nuclear) or to GAPDH (cytosolic) markers. Data are presented as mean ± SD from three independent experiments. (G) Representative fluorescence images of the antimicrobial peptide reporter *lys‐7::gfp* following exposure to the indicated treatments. Scale bar = 100 µm. (H) Quantification of Lys‐7::GFP fluorescence intensity after 4 h treatment. Data are presented as mean ± SD from three independent experiments, each analyzing > 20 worms per condition. Statistical significance in (A, B, D, F, and H) was determined by one‐way ANOVA or unpaired t‐test as appropriate. ^*^ *p* < 0.05, ^**^ *p* < 0.01, ^***^ *p* < 0.001, ^****^ *p* < 0.0001; ns, no significant difference.

We next examined whether PlcR is required for suppression of DAF‐16 activation. Using the DAF‐16::GFP reporter worms TJ356, we found that infection with BMB171 Δ*nprR*/Cry5Ba or BMB171 Δ*luxS*/Cry5Ba maintained the ability to prevent DAF‐16 nuclear accumulation, similar to wild‐type BMB171/Cry5Ba. In contrast, the BMB171Δ*plcR*/Cry5Ba mutant showed a significantly reduced activity to induce robust nuclear translocation of DAF‐16, comparable to that observed in worms exposed to the BL21/Cry5Ba control (Figure 3C,D). This phenotype was further validated by western blot analysis of nuclear and cytoplasmic fractions, which confirmed the restoration of nuclear DAF‐16::GFP accumulation in nematodes infected with the Δ*plcR* mutant (Figure 3E,F).

Consistent with these observations, loss of *plcR* also restored the expression of DAF‐16‐dependent downstream immune effectors. Using the *lys‐7::gfp* reporter strain, we observed that while wild‐type BMB171/Cry5Ba strongly suppressed *lys‐7* expression, treatment with the BMB171Δ*plcR*/Cry5Ba mutant significantly rescued *lys‐7*::*gfp* expression (Figure 3G,H). Together, these results identify PlcR as the principal regulator required for Bt‐mediated suppression of host innate immunity. By controlling factors that inhibit DAF‐16 activation and downstream immune gene expression, PlcR links bacterial quorum‐sensing‐regulated virulence to active evasion of host innate immunity defenses.

### 2.4 Genome‐Wide Identification of PlcR Targets Reveals PisP as an Immunosuppressive Effector

Having established that PlcR is required for suppression of DAF‐16‐dependent immunity, we next propose to identify the specific PlcR‐regulated downstream effector(s) responsible for this activity. To define the PlcR regulon, we performed chromatin immunoprecipitation coupled with high‐throughput sequencing (ChIP‐seq). For this purpose, we constructed a functional BMB171 strain expressing an N‐terminally 3×FLAG‐tagged PlcR under the control of its native promoter (Δ*plcR*/pHT304‐*plcR*::flag) (Figure S3). Genome‐wide ChIP‐seq analysis identified 74 protein‐coding genes significantly enriched at PlcR binding sites, revealing an extensive PlcR regulatory network (Figure 4A, Table S4). To validate these high‐throughput results, we selected representative binding peak targets (Figure 4B). The ChIP‐PCR validation confirmed specific PlcR occupancy at the promoter regions of several target genes, including *inhA*, *pisP*, *nheA*, *mogR*, *BMB171_RS08810*, and *BMB171_RS12715*, compared to the IgG control (Figure 4C).

**FIGURE 4:**
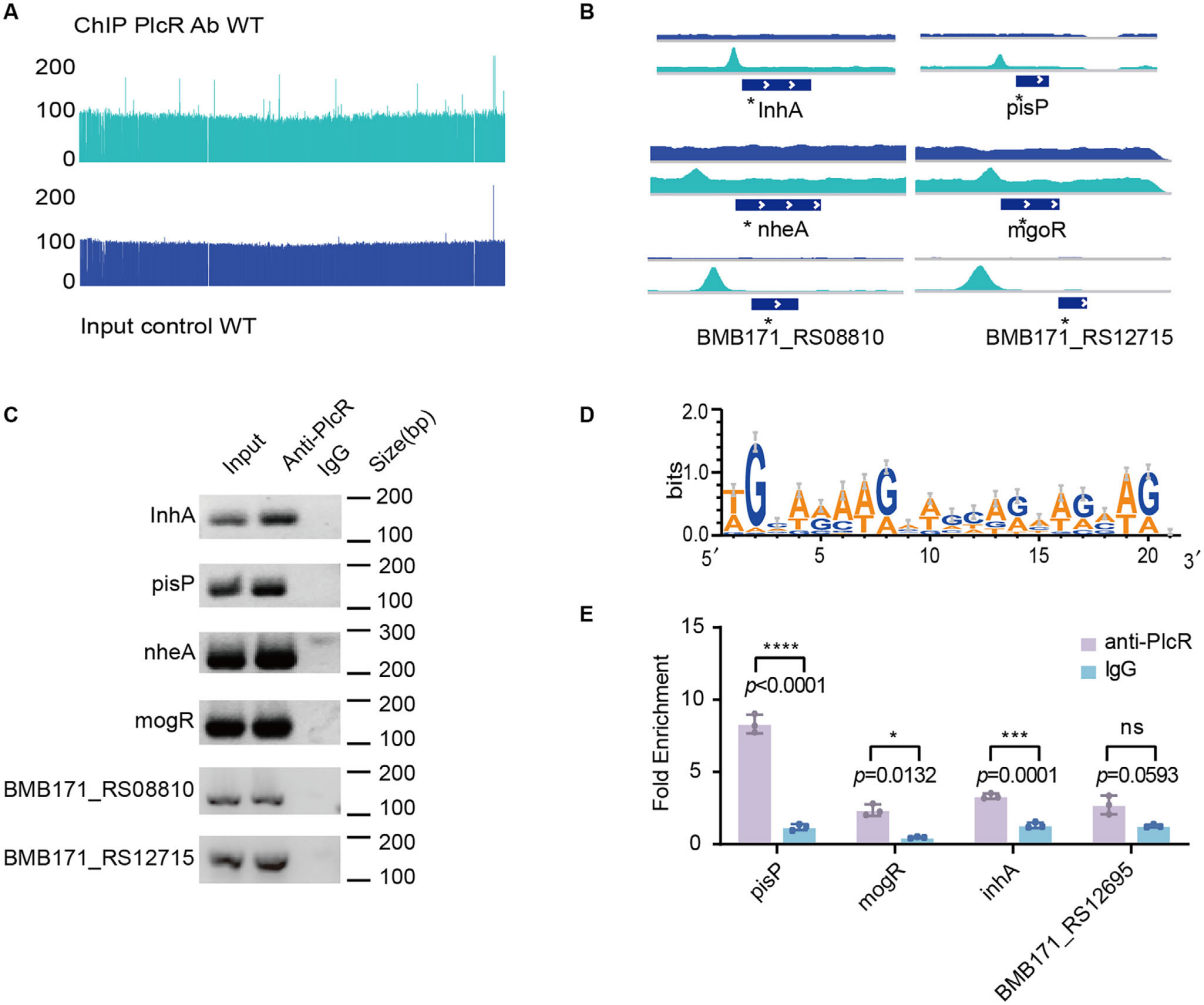
Genome‐wide ChIP‐seq analysis identifies direct transcriptional targets of PlcR in the BMB171 strain. (A) Genome‐wide visualization of ChIP‐seq data obtained from 4 h ChIP samples. Blue peaks indicate genomic regions enriched for PlcR binding in the anti‐FLAG ChIP sample, compared to the input control (BMB171). Grey lines represent genome scaffolds. (B) Representative ChIP‐seq binding profiles for selected target genes (*inhA*, *pisP*, *nheA*, *mgoR*, *BMB171_RS08810* and *BMB171_RS12715*). These loci displayed strong PlcR enrichment in the anti‐FLAG ChIP sample (lower panels) compared with the IgG control (upper panels). Binding peaks are located upstream of the corresponding translational start sites. Asterisks indicate gene positions within each 3 kb genomic region. (C) ChIP‐PCR validation of PlcR binding to selected promoter regions using an anti‐PlcR antibody, with IgG as a negative control. Amplified products correspond to the expected product sizes (100–300 bp). PlcR binding was detected at all tested promoter regions. (D) Consensus PlcR‐binding motif identified using MEME and visualized with WebLogo. Letter height reflects the relative frequency of each nucleotide at the corresponding position. (E) ChIP‐qPCR quantification of PlcR enrichment at selected promoter regions, expressed as a percentage of input DNA. Data are presented as mean ± SD from three independent experiments performed in duplicate.

Subsequent motif analysis of the identified peaks of PlcR‐enriched regions using MEME revealed a conserved 21‐bp PlcR‐binding consensus sequence (5'‐TGnnnnGnAnnnGnAGnAG‐3') that shares key features with the canonical PlcR box previously reported in *B. thuringiensis* (5'‐TATGNAN4TNCATA‐3') [36] (Figure 4D), further validating the specificity of the ChIP‐seq dataset. The newly derived motif retains partial 5' recognition elements but introduces a novel AG‐enriched pattern in the 3' region, suggesting potential variability in target recognition within this regulon [33, 37]. Gene Ontology (GO) functional annotation of the 74 PlcR‐bound genes revealed enrichment for processes associated with bacterial virulence, transport, metabolism, and motility (Figure S4), consistent with the established pleiotropic role of PlcR as a global regulator of Bt pathogenicity [38, 39].

To identify candidate PlcR‐regulated effectors potentially involved in host immune suppression, we first established that *plcR* transcript levels reach their highest levels during the late stationary phase (68 h) (Figure S5A). Based on this, we hypothesized that genes mediating PlcR‐dependent immune suppression would likely be strongly induced at this time point under these infection conditions. To test this hypothesis, we performed qRT‐PCR to compare the expression of selected PlcR target candidate genes between the wild‐type strain and the Δ*plcR* mutant at 68 h. Among the genes examined, a previously uncharacterized gene encoding a patatin‐like protein exhibited the strongest PlcR‐dependent upregulation (Figure S5B). We named this protein PisP (PlcR‐induced suppressor of immunity Protein). Notably, the high *pisP* expression during the stationary phase is consistent with the enrichment of PlcR at the *pisP* promoter region observed by ChIP‐qPCR assays (Figure 4E), indicating that *pisP* may be a direct target of PlcR. Patatin‐like proteins from diverse intracellular bacterial pathogens (e.g., *Rickettsia parkeri*) are known to be implicated in host‐pathogen interactions, facilitate escape from host vacuoles, and suppress immune responses such as autophagy [40]. The strongly PlcR‐dependent regulation of *pisP*, together with the documented immunomodulatory activities of related Patatin‐like proteins, suggested that PisP may function as a previously unrecognized Bt effector involved in host immune suppression. We therefore selected PisP for further functional characterization.

### 2.5 PlcR Directly Controls pisP Expression to Suppress Host Innate Immunity

To determine whether PlcR directly regulates *pisP* expression during infection, we first examined the interaction between PlcR and the *pisP* promoter. Electrophoretic Mobility Shift Assays (EMSAs) showed clear, concentration‐dependent shifts of FAM‐labeled *pisP* promoter DNA fragments upon incubation with increasing amounts of PlcR protein (Figure 5A, lanes 2–8). This shift was abolished by excess unlabeled probe (lane 9), and deletion of the predicted PlcR‐binding motif abolished the detectable binding of PlcR to the *pisP* promoter (lane 10), further supporting the sequence specificity of PlcR binding. Consistent with these results, a P*pisP*‐lacZ reporter exhibited significantly higher β‐galactosidase activity in the wild‐type BMB171 strain compared to the Δ*plcR* mutant (Figure 5B). Likewise, a GFP‐tagged PisP fusion demonstrated markedly reduced PisP protein accumulation in the Δ*plcR* background during infection, compared to the wild‐type strain (Figure 5C). Together, these results demonstrate that the global transcription factor PlcR directly and positively regulates *pisP* expression.

**FIGURE 5:**
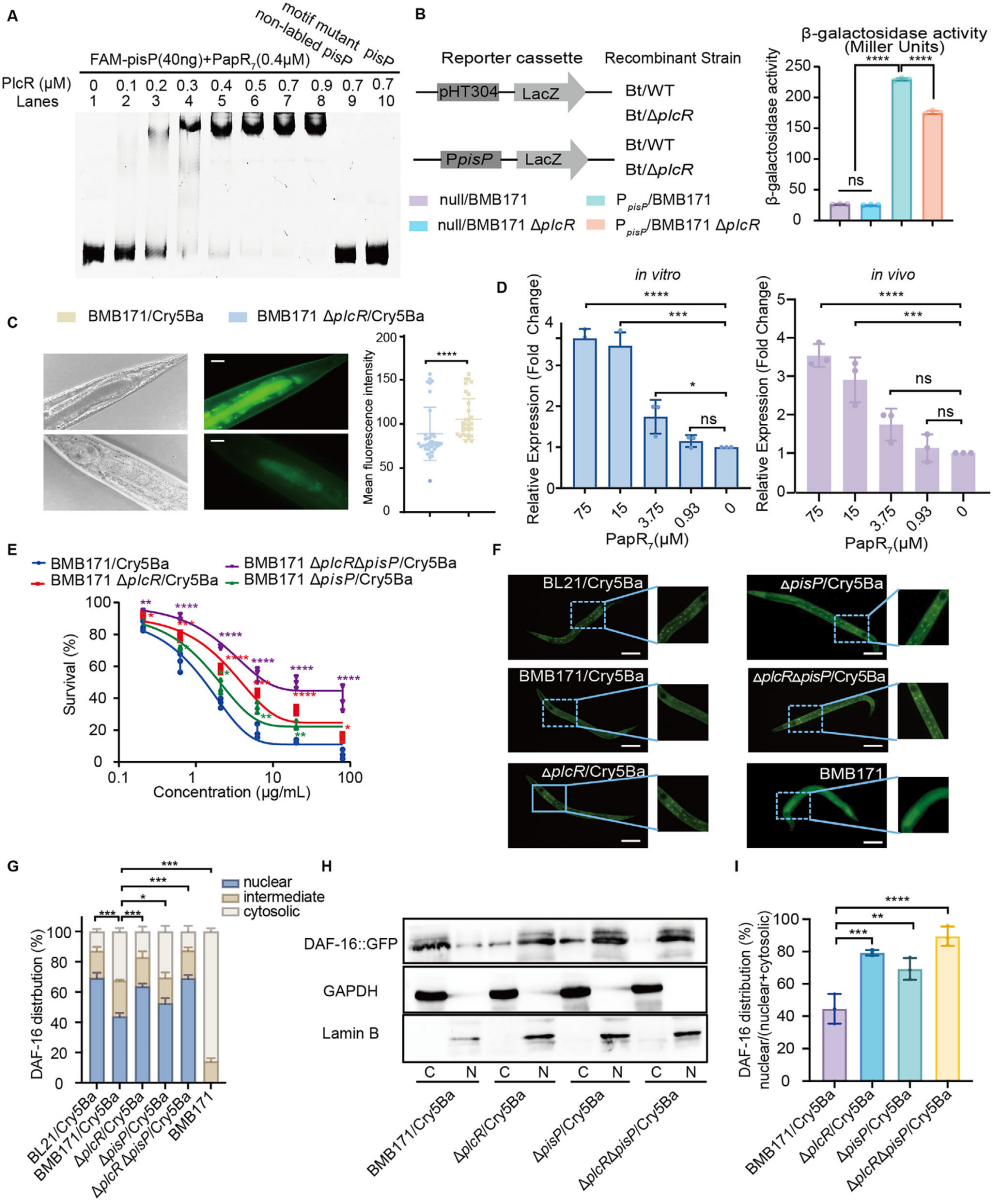
PlcR directly regulates *pisP* expression to suppress the innate immune responses in *C. elegans*. (A) Electrophoretic mobility shift assays (EMSA) demonstrating PlcR binding to the *pisP* promoter. A FAM‐labeled DNA probe was incubated with increasing concentrations of PlcR (Lanes 1–8). Excess unlabeled probe was added as a specific competitor (Lane 9, negative control), and a motif‐mutant *pisP* promoter (Lane 10, negative control) was added as a control. (B) *β*‐galactosidase activity of the P*_pisP_*‐lacZ reporter promoter in wild‐type BMB171 and BMB 171Δ*plcR* strains. A promoter‐less lacZ construct served as a negative control. Plates were incubated for 1–2 days. Quantification of β‐galactosidase activity is presented as Miller units (right panel). Data are presented as mean ± SD from three independent experiments. (C) Representative fluorescence images (left) and quantification analysis of mean fluorescence intensity (right) of GFP‐labeled PisP expression in wild‐type *C. elegans* (N2) exposed to BMB171/Cry5Ba or BMB171Δ*plcR*/Cry5Ba strains. Data are the mean ± SD from three independent experiments, each analyzing > 20 worms per condition. (D) Dose‐dependent induction of *pisP* expression in BMB171 after treatment with increasing concentrations of synthetic PapR_7_ peptide (0, 0.93, 3.75, 15, 75 µM) measured by qPCR in vitro (left panel) or in vivo (right panel). The expression levels were normalized to the 16S rRNA gene. Data are mean ± SD values from three independent experiments. (E) Dose‐response mortality assay comparing wild‐type N2 worms exposed to BMB171Δ*plcR*/Cry5Ba, BMB171 △*pisP*/Cry5Ba, BMB171△*plcR△pisP*/Cry5Ba, and the parental BMB171/Cry5Ba. Data are means ± SD from three independent experiments, each performed in triplicate with > 30 worms per condition. (F) Representative fluorescence images showing DAF‐16::GFP localization in adult *TJ356* (*daf‐16::gfp*) worms following the indicated treatments. BL21/Cry5Ba and BMB171△*plcR*/Cry5Ba induced nuclear localization, whereas BMB171/Cry5Ba and BMB171△*pisP*/Cry5Ba suppressed nuclear localization. BMB171 alone did not induce nuclear DAF‐16::GFP nuclear translocation. Scale bar = 100 µm. (G) Quantification of DAF‐16::GFP localization in *TJ356*(*daf‐16::gfp*) worms, classified as nuclear, intermediate, or cytosolic. The percentage of worms exhibiting nuclear DAF‐16::GFP localization was calculated as the number of worms classified as showing nuclear localization divided by the total number of worms scored. Data represent three independent experiments, each analyzing > 30 worms per condition. (H) Immuno‐blot analysis of DAF‐16::GFP protein in nuclear and cytosolic fractions isolated from *TJ356*(*daf‐16::gfp* worms after 4 h exposure to the indicated treatments. DAF‐16::GFP protein was detected using a primary anti‐GFP antibody. Lamin B1 and GAPDH were used as nuclear and cytosolic markers, respectively. Increased nuclear accumulation was observed in worms treated with BMB171Δ*plcR*/Cry5Ba, BMB171Δ*pisP*/Cry5Ba, and BMB171Δ*plcR*Δ*pisP*/Cry5Ba compared to BMB171/Cry5Ba. A representative blot from three independent experiments is shown. (I) Quantification of DAF‐16::GFP protein levels in nuclear and cytosolic fractions, normalized to Lamin B1 (nuclear) or GAPDH (cytosolic) markers. Data are mean ± SD from three independent experiments. Statistical significance in (B–E, G, and I) was determined by one‐way ANOVA or unpaired t‐test, as appropriate. ^*^ *p* < 0.05, ^**^ *p* < 0.01, ^***^ *p* < 0.001, ^****^ *p* < 0.0001; ns, not significant difference.

Given that quorum‐sensing (QS) systems in the *Bacillus cereus* sensu lato group are critical for coordination between gene expression, cell development, and infection [16], and that PlcR activity is controlled by the PlcR‐PapR QS system, we next examined whether *pisP* expression responds quantitatively to QS signaling in a cell density‐dependent manner. In this system, the signaling peptide PapR is secreted, processed to its active heptapeptide form (PapR_7_), and subsequently imported into the bacterial cells via the oligopeptide permease Opp [41]. Once PapR_7_ interacts with PlcR, their binding activates PlcR, enabling the PlcR‐PapR_7_ complex to recognize and bind to the “PlcR box” sequence in target promoters [42]. Recent studies in other QS systems have revealed that QS‐regulated genes frequently exhibit graded responses to signaling inputs, rather than binary ON/OFF behavior switches [43]. Consistent with a QS‐dependent regulatory mechanism, we found that the transcription level of *papR* progressively increased during the vegetative phase of Bt infection in *C. elegans*, reached its highest levels at 68 h post‐infection, coinciding with the maximal *plcR* expression, and was associated with high bacterial density. Subsequently, declined following host death and bacterial sporulation (Figure S6). To directly determine if *pisP* expression quantitatively responds to PapR_7_ levels, bacteria were exposed to increasing concentrations of synthetic PapR_7_ peptide, resulting in a clear dose‐dependent induction of *pisP* transcription, reaching maximal activation at 75 µM (Figure 5D). Notably, a similar increase in *pisP* transcription was observed in the Δ*papR* mutant supplemented with PapR_7_ under host‐associated conditions in *C. elegans*, indicating that PapR_7_‐dependent regulation of *pisP* is maintained during infection (Figure 5D). To further characterize this interaction, we performed Isothermal Titration Calorimetry (ITC) analysis. The PlcR‐PapR_7_ complex displayed its highest binding affinity for the *pisP* promoter at a 1:1 molar ratio (*K*_d_ = (4.915E ± 1.26E‐6)), whereas lower PapR_7_ concentrations substantially reduced promoter binding, and PapR_7_ alone showed no detectable DNA binding (Figure S7). These results indicate that activation of *pisP* transcription is quantitatively modulated by the PlcR‐PapR QS system. Collectively, these findings support a model in which *pisP* expression is progressively induced by the accumulation of the PapR_7_ signal during infection.

To define the functional contribution of PisP to PlcR‐dependent virulence, we constructed *ΔpisP* and *ΔplcRΔpisP* mutants and evaluated their pathogenicity in *C. elegans*. Single deletion of *pisP* had only a modest effect on acute toxicity (LC_50_ value of 4.489 µg/ml; 95% confidence interval of 3.16–6.043 µg/mL) relative to the wild‐type Bt/toxin BMB171/Cry5Ba strain (LC_50_ value of 3.374 µg/mL; 95% confidence interval of 0.692–5.841 µg/mL). In contrast, deletion of the global regulator *plcR* markedly reduced virulence (LC_50_ value of 7.981 µg/mL; 95% confidence interval of 6.31–9.795 µg/mL). Strikingly, the double mutation (*ΔplcRΔpisP*) exhibited a substantially greater reduction, a synergistic loss of toxicity (LC_50_ value of 15.624 µg/mL; 95% confidence interval of 12.562–19.521 µg/mL) (Figure 5E, Table S3), suggesting that PisP acts cooperatively with additional PlcR‐regulated effectors to promote full Bt pathogenesis. To further investigate whether the contribution of PisP to virulence depends on DAF‐16, we evaluated the toxicity of BMB171/Cry5Ba and BMB171*ΔpisP*/Cry5Ba in *daf‐16* mutant worms. In contrast to wild‐type N2 worms, the two strains exhibited no significant difference in toxicity toward *daf‐16* mutant worms (Figure S8). These findings indicate that PisP suppresses Cry5Ba‐induced host immune responses in a DAF‐16‐dependent manner.

To determine whether PisP can enter host intestinal cells, we labeled purified PisP with rhodamine and fed L4‐stage *C. elegans* with either rhodamine‐labeled PisP or rhodamine alone for approximately 4–6 h. Fluorescence microscopy analysis revealed that rhodamine‐labeled PisP was detected in the intestinal tract, with the fluorescent signal extended beyond the lumen into the surrounding intestinal cells (Figure S9). In contrast, the fluorescence signal from rhodamine alone remained largely restricted to the intestinal lumen tract under the same conditions. These observations indicate that labeled PisP can cross the luminal boundary and potentially enter into the host midgut cells. We then asked whether PisP specifically contributes to suppressing host innate immunity. Fluorescence microscopy of N2‐TJ356 worms demonstrated that, in contrast to wild‐type infection, which efficiently prevented DAF‐16::GFP nuclear translocation, *ΔplcR* and *ΔplcRΔpisP* mutants all failed to inhibit DAF‐16 activation, while the *ΔpisP mutant* showed a partial defect, resulting in robust nuclear localization comparable to that observed in worms exposed to BL21/Cry5Ba (Figure 5F,G). This phenotype was confirmed by western blot analysis of nuclear fractions (Figure 5H,I). Consistent with these findings, the expression levels of DAF‐16‐dependent immune defense genes (*mtl‐1*, *lys‐7*, *thn‐2*, and *spp‐2*) were significantly increased in worms exposed to BMB171Δ*pisP*/Cry5Ba compared with those exposed to BMB171/Cry5Ba (Figure S10). Collectively, these data define a functional division within the PlcR regulon. While acute Bt virulence depends on the combined action of multiple PlcR‐regulated effectors, the specific suppression of host innate immunity depends critically on PlcR‐mediated expression of *pisP*, identifying PisP as a key PlcR‐regulated effector responsible for antagonizing DAF‐16‐dependent immune responses.

### 2.6 Exploiting Conserved PisP‐Mediated Immunosuppression Enhances Pesticidal Efficacy

Having established that PisP is a key immunosuppressive effector that targets the host DAF‐16 signaling pathway, we next investigated its distribution among Bt strains. To assess the conservation of PisP, we analyzed 889 publicly available Bt genomes and searched for PisP homologs using Basic Local Alignment Search Tool for proteins (BLASTP)Individual Culture Sporulation Medium with thresholds of > 80% sequence coverage and >80% amino acid identity. Remarkably, PisP homologs were identified in 70.98% (631/889) of the analyzed genomes, including highly virulent strains such as HD1 (Figure S11), indicating that PisP is broadly conserved across Bt populations.

The widespread distribution of PisP suggests that immune suppression may represent a common strategy of Bt pathogenesis. Given its ability to suppress host defenses during infection, we hypothesized that enhancing PisP activity could improve Bt efficacy. To test this possibility, we constructed a *pisP* overexpression strain (BMB171 *pisP^OE^*) and evaluated its toxicity against N2 worms. Overexpression of *pisP* significantly increased nematicidal activity compared to the parental strain (Figure 6A), demonstrating that boosting immune suppression enhances pathogenic efficacy.

**FIGURE 6:**
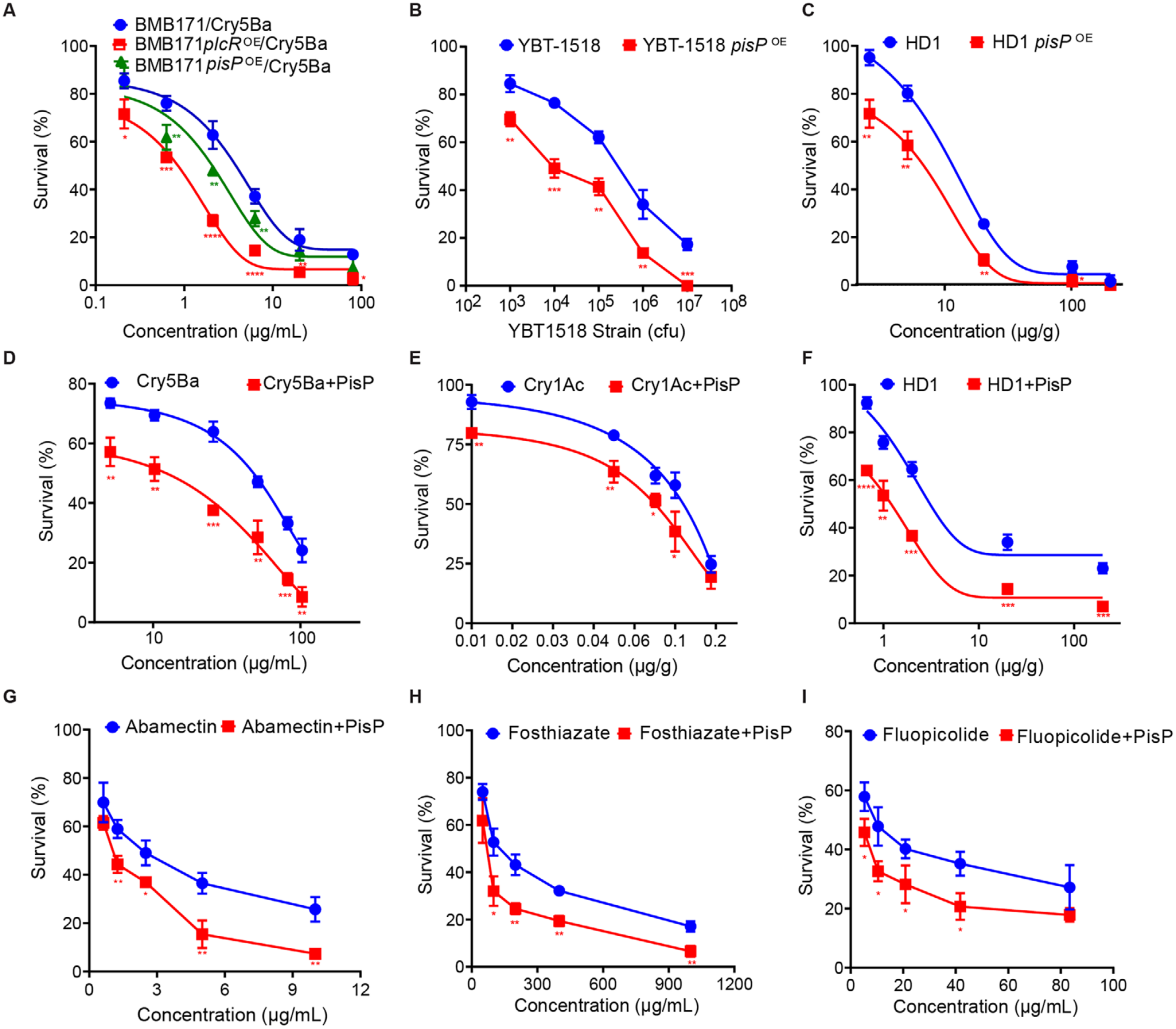
Genetic overexpression and exogenous co‐application of PisP enhance pesticidal efficacy across multiple target species. (A) Dose‐response mortality assays comparing the susceptibility of wild‐type N2 worms exposed to the parental BMB171 strain, or to the BMB171 *pisP^OE^* (*pisP*‐overexpressing) strain and BMB171*plcR^OE^* (*plcR*‐overexpressing) strain. Data are presented as mean ± SD from three independent experiments, each performed with triplicate samples with > 30 worms per condition. Cry5Ba was used at concentrations from 0 to 100 µg/mL. (B) Survival analysis of *D. destructor* exposed to increasing concentrations of the parental YBT‐1518 strain (10^3^–10^7^ CFU) or a PisP‐overexpressing derivative YBT‐1518*pisP^OE^* strain (10^3^–10^7^ CFU). (C) Toxicity comparison between the parental HD1 strain and a PisP‐overexpressing derivative HD1*pisP^OE^* strain against *Helicoverpa armigera* larvae. Data in (B, C) are presented as mean ± SD from three independent experiments, each performed in triplicate with > 30 worms or larvae per condition. (D) Dose‐response toxicity assays of *D. destructor* treated with purified Cry5Ba toxin alone (from 5.12 to 100 µg/mL) or in combination with purified PisP protein (200 µg/mL). (E) Toxicity comparison of purified Cry1Ac toxin alone (from 0 to 0.2 µg/mL) or combined with purified PisP (200 µg/mL) against *H. armigera* larvae. (F) Toxicity of the HD1 strain alone or supplemented with purified PisP protein (200 µg/mL) against *S. frugiperda* larvae over a range of concentrations (from 0 to 200 µg/g). (G–I) Motility assays evaluating the effects of the nematicides, Abamectin (0–10 µg/mL) (G), Fosthiazate (0–1000 µg/mL) (H), and Fluopicolide (0–80 µg/mL) (I), applied alone or in combination with purified PisP protein (200 µg/mL) against *D. destructor*. Data are presented as mean ± SD from three independent experiments, each performed in triplicate with > 30 organisms per condition. Statistical significance in (A–I) was determined by one‐way ANOVA or unpaired t‐test as appropriate. ^*^ *p* < 0.05, ^**^ *p* < 0.01, ^***^ *p* < 0.001, ^****^ *p* < 0.0001; ns, not significant difference.

DAF‐16 is the *C. elegans* ortholog of the evolutionarily conserved FOXO family of transcription factors, which is widely distributed across metazoans and plays important roles in the regulation of stress responses, metabolism, longevity, and innate immune functions [24, 44, 45]. To examine the evolutionary conservation of FOXO among different pest species, we performed comparative sequence analysis of FOXO proteins from *C. elegans*, *Ditylenchus destructor*, *Helicoverpa armigera*, *Spodoptera frugiperda*, and other representative pest species. These proteins retained the characteristic Forkhead DNA‐binding domain, with substantial sequence conservation within this region (Figure S12). Given the conservation of this domain and the broad host range of Bt among nematodes and insects, we next investigated whether PisP could broadly enhance Bt activity against different agricultural pests. Introduction of the *pisP* overexpression construct into the highly virulent Bt strain YBT1518 (YBT1518‐*pisP^OE^*) significantly enhanced its efficacy against the destructive plant‐parasitic nematode *D. destructor* (Figure 6B). Similarly, overexpression of *pisP* in the commercial Bt strain HD1 (HD1‐*pisP^OE^*) enhanced insecticidal activity against the cotton bollworm, *H. armigera* (Figure 6C). These findings indicate that increased PisP production can enhance Bt efficacy against both nematode and insect hosts.

We further explored whether PisP could function as an exogenous bio‐adjuvant for existing pest control formulations. Co‐application of purified PisP along with Cry5Ba toxin significantly increased mortality in both *C. elegans* N2 and the plant‐parasitic *D. destructor* compared with Cry5B toxin alone (Figure S13, Figure 6D). Similarly, PisP enhanced Cry1Ac toxicity against *H. armigera* (Figure 6E) and increased the efficacy of HD1 against the fall armyworm, *S.frugiperda*, in a dose‐dependent manner (Figure 6F).

Finally, we tested whether PisP could potentiate conventional chemical nematicide formulations. Co‐formulation with PisP significantly enhanced the efficacy of three widely used commercial nematicides with distinct modes of action: abamectin (a chloride channel activator), tioxazafen (an acetylcholinesterase inhibitor), and fluopyram (a mitochondrial complex II inhibitor). PisP significantly increased the nematicidal activity of abamectin and tioxazafen (Figure 6G,I). Notably, PisP also significantly improved the activity of fluopyram at lower chemical doses, although this effect plateaued at higher, saturating concentrations (Figure 6H).

To further investigate whether PisP‐mediated enhancement of pest‐control activity was associated with modulation of host immune responses in insects, we focused on *S. frugiperda*. We selected two groups of genes for transcriptional analysis: orthologs of genes known to be regulated by DAF‐16 in *C. elegans* and immune‐related genes previously characterized in *S. frugiperda* [46]. Following treatment with PisP in combination with Cry1Ac or abamectin, several genes from both groups showed reduced transcript levels compared with the corresponding treatments without PisP (Figure S14). Notably, the expression of representative immune effector genes, including genes encoding antimicrobial peptides, was also reduced in the presence of PisP. These results indicate that PisP can modulate the expression of host defense‐ and immune‐related genes in *S. frugiperda*, providing additional evidence that the immunomodulatory effects associated with PisP may extend beyond *C. elegans*.

Collectively, these results indicate that PisP enhances the efficacy of Bt, other biological and chemical pest‐control agents against multiple agricultural pests. Together with the observed modulation of host defense‐ and immune‐related gene expression in *S. frugiperda*, these findings support the potential application of PisP as a bio‐adjuvant to improve pest‐control efficacy.

## 3 Discussion

The evolutionary arms race between microbial pathogens and their hosts is driven by continuous cycles of immune activation and pathogen evasion. Successful pathogens must not only damage host tissues but also overcome host immune defenses. While microbial immune suppressors have been identified in diverse host‐pathogen systems, how bacterial pathogens coordinate immune suppressors with virulence factor production during infection remains poorly understood. In this study, we demonstrate that the PlcR‐PapR quorum‐sensing system of Bt integrates these two processes. Specifically, we show that PlcR directly induces expression of the conserved effector PisP, which suppresses host immunity by preventing nuclear translocation of the FOXO transcription factor DAF‐16, a central regulator of innate immune responses, thereby disabling a central node of host immunity (Figure 7). These findings expand the established role of the PlcR‐PapR system beyond the regulation of classical virulence effectors and reveal that quorum sensing can coordinate both host damage through virulence expression and immune suppression to maximize infection success.

**FIGURE 7:**
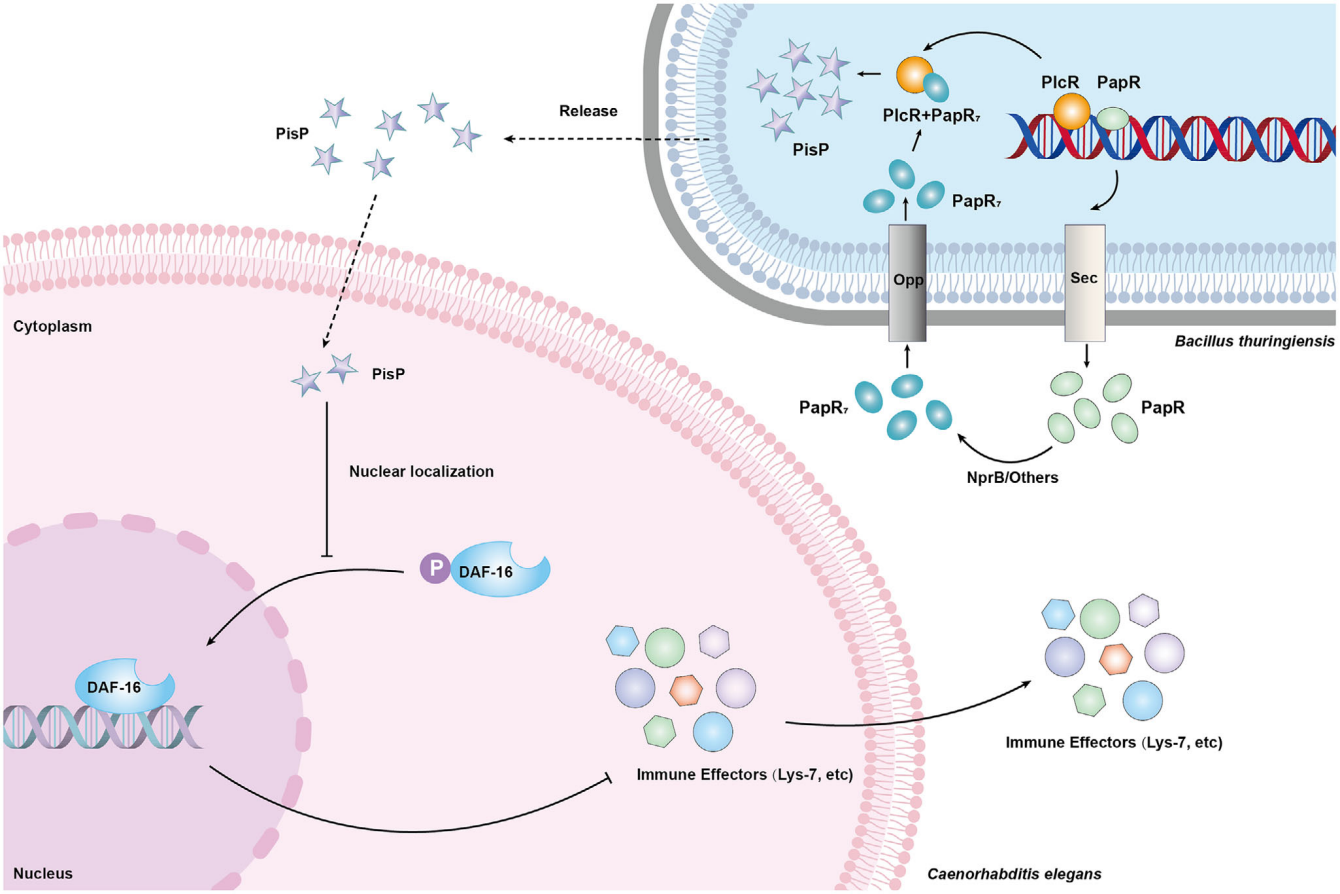
Model of Bt‐mediated immune evasion through the PlcR‐PisP‐DAF‐16 axis. PapR is synthesized as a precursor peptide, secreted extracellularly, and processed by extracellular proteases (e.g., NprB) into the mature signaling heptapeptide PapR_7_ [15]. PapR_7_ is imported back into bacterial cells through the oligopeptide permease (Opp) system [42]. During infection, accumulated PapR_7_ binds to and activates the global transcriptional regulator PlcR [14, 47]. The quorum‐sensing regulator PlcR activates the expression of PisP in *B. thuringiensis*. During infection, PisP modulates the host's innate immune signaling by inhibiting the nuclear translocation of the transcription factor DAF‐16. The resulting reduction in nuclear DAF‐16 leads to decreased expression of downstream defense genes (e.g., *lys‐7*), thereby suppressing *C. elegans* innate immune responses and contributing to maximal Bt pathogenicity. The cellular entry of PisP is represented by a dashed line, indicating that the experimental data support the uptake of PisP into host intestinal cells, whereas the mechanism responsible for its cellular entry remains unknown.

A key finding of this study is the functional specialization of PisP as an immunomodulatory effector rather than a classical toxin. Purified PisP exhibited no detectable toxicity when administered alone (Figure S15) yet markedly enhanced the activity of Cry toxins and Bt strains during infection. This observation highlights a functional division of labor within the Bt virulence arsenal. Whereas Cry toxins directly damage host tissues, PisP lowers the host immune barrier, thereby creating a physiological environment in which toxin activity can achieve maximal efficacy. Similar cooperative interactions between tissue‐damaging factors and immune‐suppressive effectors have been reported in diverse bacterial pathogens, suggesting that the coordinated deployment of these distinct virulence functions may represent a general strategy for overcoming host defenses.

Our genetic analyses further support this model. Deletion of *pisP* alone did not significantly affect Bt virulence, whereas deletion of (Δ*plcR*) caused only a moderate reduction in pathogenicity. In contrast, simultaneous deletion of both genes caused a dramatic loss of virulence (approximately 10‐fold decrease). This phenotype is consistent with the complex architecture of the PlcR regulon, which controls numerous virulence‐associated genes, including factors involved in immune suppression. For example, the metalloprotease InhA2 has previously been shown to contribute to both virulence and immune evasion across diverse bacterial species [48]. The modest phenotype of the Δ*pisP* mutant therefore likely reflects functional compensation by other PlcR‐dependent factors. Conversely, the relatively mild phenotype of the Δ*plcR* mutant may reflect residual *pisP* expression through alternative regulatory pathways. Indeed, *pisP* transcription is reduced but not completely abolished in the Δ*plcR* background (Figure S16), indicating additional regulatory mechanisms involved in *pisP* expression. Previous studies have shown that members of the GntR/HutC family, including NupR, can regulate subsets of PlcR‐dependent genes [49, 50], which may contribute to this residual expression of *pisP* in Bt. Furthermore, global regulators outside the PlcR network may maintain expression of other virulence factors that partially compensate for PlcR loss [51]. Notably, the severe reduction in virulence observed in the double mutant Δ*plcRΔpisP* therefore likely reflects the simultaneous disruption of two complementary processes: coordinated virulence gene expression (regulated by PlcR), and immune suppression (mediated by PisP), resulting in a marked loss of pathogenicity.

Our study also identifies PisP as a previously uncharacterized member of the patatin‐like protein family. We showed that PisP suppresses host immunity by inhibiting the nuclear translocation of the transcription factor DAF‐16. However, the molecular mechanism and mode of action appear to differ fundamentally from canonical patatin‐like phospholipases. Although PisP contains a conserved patatin and cPLA2 domain (Figure S17A), its predicted structure differs substantially from those of the well‐characterized bacterial phospholipases VipD from *Legionella pneumophila* and ExoU from *Pseudomonas aeruginosa* [52, 53]. The overall sequence identity between PisP and these homologs is low, although certain conserved motifs are retained (Figure S17B). To further explore their structural relationship, we predicted the three‐dimensional structure of PisP using AlphaFold3 and performed comparative structural analyses with VipD and ExoU. Overall, PisP exhibits low structural similarity to both proteins, with RMSD) values of 3.057 and 4.198, respectively, and the limited similarity is primarily confined to the N‐terminal region (Figure S17C). Notably, VipD and ExoU require a C‐terminal domain for activation of their phospholipase activity [52, 53], whereas PisP lacks the corresponding C‐terminal domain, suggesting a distinct structural and functional organization. Consistent with this observation, no detectable phospholipase activity was observed for PisP in vitro, as analyzed by the egg yolk plate assay, suggesting that PisP may have diverged functionally from canonical patatin‐like phospholipases and may operate through a distinct mechanism. While it remains possible that PisP requires host cofactors or specific intracellular conditions for activation, its structural divergence raises the alternative possibility that it operates through a non‐enzymatic mechanism, potentially as a protein–protein interaction module. Defining the biochemical activity of PisP, therefore, remains a key objective for future work.

At the mechanistic level, how PisP interferes with DAF‐16 nuclear translocation remains to be elucidated. PisP could potentially act through direct interaction with DAF‐16 or indirectly through regulatory factors that control DAF‐16 localization. However, our yeast two‐hybrid analysis did not detect a direct interaction between PisP and DAF‐16 under the conditions tested (Figure S18). Moreover, treatment with BMB171 in combination with Cry5Ba, under which PisP is present, did not significantly alter *daf‐16* transcript levels compared with BL21/Cry5Ba treatment (Figure 1C). In contrast, comparison with the corresponding Δ*pisP* mutant under the same Cry5Ba treatment conditions showed that the presence of PisP markedly inhibited DAF‐16 nuclear translocation and reduced the expression of downstream immune defense genes (Figure 5F–I). Together, these findings suggest that PisP is unlikely to substantially regulate *daf‐16* at the transcriptional level or through a direct physical interaction with DAF‐16, but may instead affect DAF‐16 localization through upstream or intermediate regulatory mechanisms. To explore the potential involvement of such intermediate regulators, we examined several well‐characterized components of the DAF‐16 regulatory network, including AKT‐1, PDK‐1, AGE‐1, INS‐1, INS‐18, INS‐39, and PAR‐1. Yeast two‐hybrid assays did not detect interactions between PisP and any of these candidate factors (Figure S18). Although these negative results do not exclude their potential involvement in vivo, they suggest that PisP is unlikely to act through direct interaction with the specific regulatory factors tested. In *C. elegans*, DAF‐16 localization is tightly controlled by the insulin/IGF‐1 signaling pathway [21, 44]; therefore, it is possible that PisP influences one or more components of this signaling network, or additional host regulatory factors involved in nuclear transport or DAF‐16 associates signaling. Identifying the intermediate factors and molecular mechanisms linking PisP to DAF‐16 will be important for understanding how Bt modulates host immune signaling at the molecular level.

An additional important finding in this work is the remarkable conservation of PisP across the majority of Bt strains (Figure S11). PisP homologs were identified in approximately 71% of the 889 Bt genomes analyzed, including several highly virulent strains. Notably, both *pisP*‐positive and *pisP*‐negative strains carried highly diverse pesticidal protein repertoires, ranging from 0 to 16 proteins per genome, and were associated with broad and largely overlapping potential target spectra, including Coleoptera, Blattodea, Lepidoptera, Hemiptera, Diptera, Rhabditida, and Orthoptera (Table S5). Thus, the presence of PisP was not clearly associated with a specific pesticidal protein repertoire or predicted target spectrum, suggesting that PisP‐mediated immune modulation may function as a complementary virulence strategy across diverse Bt toxin backgrounds. Beyond Bt, PisP‐like proteins were identified across diverse bacterial taxa, including multiple members of the *Bacillus* group and other bacterial species. Under stringent criteria of ≥ 80% query coverage and ≥ 80% sequence identity, PisP homologs were detected in 53.0% of *B. cereus* genomes. When the sequence identity threshold was relaxed to ≥ 25% while retaining ≥ 80% query coverage, more distantly related PisP‐like proteins were detected in > 98% of the examined *B. cereus*, *B. mycoides*, *B. toyonensis*, and *B. wiedmannii* genomes, as well as 100% of *Brevibacillus laterosporus* and 99.3% of *Serratia marcescens* genomes (Table S6). Despite their lower sequence identity, these proteins retained the characteristic PisP‐associated domain, supporting their classification as PisP‐like proteins. The broad distribution and conservation of this domain suggest that PisP‐related proteins may have conserved functions in bacterial interactions with their host or environment. However, whether these more distantly related PisP‐like proteins retain the immunomodulatory activity similar to that of Bt PisP remains to be experimentally determined.

Beyond fundamental mechanisms, our findings have important direct practical implications for agricultural biotechnology. For decades, the widespread use of Bt‐based biopesticides and transgenic crops has been central to sustainable pest control, significantly reducing our reliance on agrochemicals. However, their long‐term efficacy is increasingly threatened by the evolution of pest resistance, often associated with enhanced host immune responses and alterations in toxin receptors [54, 55, 56]. Growing evidence indicates that host immune responses can mitigate Cry toxin activity and accelerate resistance development [57]. Our data demonstrate that increasing PisP activity, either through genetic overexpression in commercial Bt strains (e.g., YBT1518, HD1), or through exogenous application of purified PisP in combination with commercial Bt formulations, significantly enhances pesticidal efficacy across diverse targets. Importantly, co‐application of purified PisP with chemical nematicides (abamectin, tioxazafen, fluopyram) allowed effective control at reduced doses of these chemical nematicides while maintaining or even improving pest mortality. These findings establish immune suppression as a tractable target for improving pest control performance.

The immunosuppressive mechanism of PisP in insect hosts remains unclear. Although FOXO proteins are conserved among *C. elegans*, other nematodes, and the insect pests examined in this study, this conservation alone does not establish the involvement of FOXO in PisP‐mediated effects in insects. Transcriptional analyses in *S. frugiperda* showed that PisP‐containing treatments reduced the expression of several genes associated with host defense and immunity, including antimicrobial peptide genes (Figure S14), suggesting that PisP may also modulate host immune responses in insects. Further studies examining FOXO localization and activity in Lepidoptera will be required to determine whether these effects involve FOXO signaling or other conserved immune‐regulatory pathways.

In conclusion, our study reveals a previously unrecognized mechanism by which the PlcR–PapR quorum‐sensing system coordinates virulence and immune suppression during Bt infection. By identifying PisP as a conserved DAF‐16‐targeting immune suppressor and demonstrating its utility as a pesticidal bio‑adjuvant, we provide both mechanistic insight into Bt pathogenesis and a framework for the development of next‐generation biological control strategies based on manipulation of host immunity that would impact and delay the onset of pest resistance.

## 4 Experimental Section

### 4.1 Bacterial Strains, Culture Conditions and Cry5Ba and PisP Purification

The bacterial strains, plasmids and primers used in this study are listed in Table S7. *Escherichia coli* strains were cultured in Luria‐Bertani (LB) medium (10 g/L tryptone, 5 g/L yeast extract, 5 g/L NaCl) at 37°C. *B. thuringiensis* strains were grown in LB or Individual Culture Sporulation Medium (ICPM) medium [12] (6 g/L tryptone, 8 g/L Glucose, 1 g/L CaCO_3_, 0.5 g/L MgSO_4_, 0.5 g/L KH_2_PO_4_) at 28°C. When required, antibiotics were added at the following concentrations: erythromycin (25 µg/mL), ampicillin (100 µg/mL), kanamycin (100 µg/mL), and spectinomycin (50 µg/mL). In‐frame deletion mutants of *plcR*, *nprR*, and *luxS* (Δ*plcR*, Δ*nprR*, and Δ*luxS*) were generated in the acrystalliferous Bt strain BMB171 as described below in Section 4.14. The construction of *pisP* and *papR* knockout mutants using the CRISPR‐Cas9 system is also described in detail in that section.

For recombinant PisP production, the *pisP* gene was cloned into pET‐21b and heterologously expressed in *E. coli*. Recombinant His‐tagged PisP was purified by nickel‐affinity chromatography. The Bt crystal inclusions were prepared as previously described [58]. Cry5Ba was purified directly from Bt cultures using a previously established isoelectric precipitation‐based purification method [59].

### 4.2 *C. elegans*, *D. destructor* and Culture Conditions

The wild‐type *C. elegans* strain N2 and the mutant strains *daf‐16*(mu86)*, daf‐16::gfp*(TJ356)*, lys‐7::gfp*(SAL105) were obtained from the *Caenorhabditis Genetics Center* (CGC, http://www.cbs.umn.edu/CGC/). Worms were maintained at 20°C on nematode growth medium (NGM) plates (0.3% NaCl, 0.25% tryptone, and 1.5% agar) seeded with *E. coli* strain OP50 as their food source. Synchronized L4 stage worms were prepared as previously described [60]. The plant‐parasitic nematode *D. destructor* strain Dd01was originally isolated from infected sweet potatoes in Wuhan, China, and was cultured and extracted as previously described [61].

### 4.3 Quantitative RT‐PCR

Gene expression analyses were performed by quantitative reverse transcription PCR (qRT‐PCR) as previously described [26]. For *C. elegans* gene expression analysis, synchronized L4‐stage worms were exposed to *E. coli* strain BL21/Cry5Ba or Bt BMB171/Cry5Ba for 4 h at 25°C. For bacterial gene expression analyses during infection, worms were exposed to BMB171/Cry5Ba or BMB171 Δ*plcR*/Cry5Ba for 21, 36, and 68 h at 25°C. For *S. frugiperda* gene expression analysis, *S. frugiperda* larvae were fed a diet containing 0.06 µg/g abamectin or 0.05 µg/g Cry1Ac for 48 h. Control larvae were fed on untreated diet. Total RNA was extracted using TRIzol reagent (Invitrogen, Carlsbad, California, USA). Three independent biological replicates were analyzed, with each sample measured in technical duplicate. Relative transcript abundance was calculated using 2^−△△^Ct method with *tba‐1* (*C. elegans*), *RPL18* (*S. frugiperda*) or *16S rRNA* (Bt) as the internal reference genes. The antimicrobial peptide (AMP) genes selected for analysis were *gloverin*, *cecropin*, *moricin*, *lebocin2*, *gallerimycin*, *spodoptericin*, and *lepidoptin* [46]. In addition, orthologs of *C. elegans* DAF‐16 target genes were also included, comprising *cyp‐35A*, *Hsp‐12*, *nnt‐1*, and *XP_050561655.1*.

### 4.4 Nematode and Insect Mortality Bioassays

For *C. elegans* bioassays, synchronized worms (with > 30 worms per condition) were exposed to different concentrations of purified Cry5Ba toxin or crystal inclusions (from 0 to 100 µg/mL) in S‐medium using 96‐well plates. Each treatment was performed in triplicate and repeated in three independent experiments. The plates were incubated at 25°C for 120 h, after which survival rates were recorded. Worms were scored as dead when they failed to respond to touch and pharyngeal pumping was no longer observed. For co‐application assays, the purified PisP was used at 200 µg/mL in bioassays. For insect bioassays, *H. armigera* and *S. frugiperda* neonates were provided by Keyun, which was co‐founded by the State Key Laboratory of Integrated Management of Pest Insects and Rodents. The bioassays were performed using the surface contamination method as previously described [62]. Five different concentrations of crystal inclusions or purified Cry proteins were applied to the diet in 24‐well polystyrene plates (one larva per well and one plate per toxin concentration). Each treatment was performed in triplicate. Larvae fed on untreated diet served as negative control. The plates were incubated at 26°C, and mortality was recorded after seven days. Larvae were considered dead if no movement was detected upon gentle stimulation. The LC_50_ values were determined using Probit statistical analysis.

### 4.5 Western Blotting Analysis

After feeding the TJ356(*daf‐16::gfp*) worms with the spores of BMB171/Δ*plcR*, BMB171/Δ*nprR*, BMB171*/*Δ*luxS*, BMB171/Δ*pisP*, BL21/Cry5Ba, or BMB171 (all supplemented with equivalent amounts of Cry5Ba,) the worms were washed three times with ice‐cold M9 buffer and washed two times in extraction buffer (10 mM HEPES (pH 7.1), 5 mM MgCl_2_, 2 mM dithiothreitol, 10% glycerol, and protease inhibitor 1 mM PMSF). Samples were snap‐frozen in liquid nitrogen, thawed on ice, and homogenized. Crude extract lysates were centrifuged at 1000 × g for 5 min to separate the nuclear (pellet) and cytosolic (supernatant) fractions. Nuclear pellets were washed twice with extraction buffer and resuspended in 2× Laemmli buffer. Cytosolic proteins were precipitated with ice‐cold acetone and resuspended in 2× Laemmli buffer [63]. Both the nuclear and cytosolic fractions were resolved by SDS‐PAGE (10% polyacrylamide) and transferred to nitrocellulose (NC) membranes (Life Technologies). Membranes were blocked with 5% BSA in PBS containing 0.05% Tween 20 (PBST), washed three times with PBST, and incubated with primary antibody (1:1000 dilution) against GAPDH, GFP, and Lamin B1 (HUABIO, China, Lot: H651800022, H640610052 and H681468041, respectively). After washing membranes three times with PBST, they were incubated with HRP‐coupled secondary antibody (Proteintech) (1:5000 dilution) and washed three times again. Signals were visualized using SuperSignal West Pico chemiluminescent substrate (Thermo Scientific).

### 4.6 RNA‐Sequencing and Transcriptome Analysis

Total RNA was extracted from worms exposed to BMB171/Cry5Ba and BL21/Cry5Ba using Trizol reagent (Invitrogen, USA). RNA quality assessment, mRNA purification, cDNA synthesis, and the synthesis of the second strand of cDNA were performed as previously described [64]. Non‐strand‐specific, paired‐end RNA‐seq libraries were generated using the TruSeq RNA Sample Prep kit (Illumina, USA). About 150 nucleotides of sequence data were obtained from both ends of each cDNA fragment utilizing the HiSeq platform (Illumina), following the manufacturer's instructions. After sequencing, reads of low quality, those contaminated with adapters, and sequences with a high ratio of unknown bases (designated as N) were removed to obtain clean reads, which were de novo assembled using Trinity, annotated, and aligned to the *C. elegans* reference genome (WormBase release WS235) using Tophat2. Lists of unregulated genes were exported, curated, and updated to WormBase WS270. Gene Ontology (GO) terms analysis was performed using g: Profiler (https://biit.cs.ut.ee/gprofiler/gost) and WormCat (http://wormcat.com/). Results are listed in Table S1.

### 4.7 Fluorescence Analysis

GFP fluorescence intensity was quantified using ImageJ software (NIH). Worms were exposed to GFP‐tagged strains (BMB171 *pisP‐gfp* or BMB171 Δ*plcR*‐ *pisP‐gfp*) for 4 h at 25°C, washed twice with M9 buffer, and mounted on 2% agarose pads. Images were acquired using an Olympus BX51 fluorescence and phase contrast microscope at 20× magnification with identical image settings for all treatment groups. (Olympus, Tokyo, Japan). Fluorescence intensity was quantified as the integrated pixel intensity normalized to the imaged area. Three plates were analyzed for each condition, and all experiments were repeated independently three times.

### 4.8 DAF‐16 Nuclear Localization Assay

N2 or TJ356 worms expressing DAF‐16GFP (*daf‐16::gfp*), were exposed to the indicated bacterial strains (BMB171, BMB171/Cry5Ba, BMB171 Δ*plcR*/Cry5Ba, BMB171 Δ*nprR*/Cry5Ba, BMB171 Δ*luxS*/Cry5Ba, BMB171 Δ*pisP*/Cry5Ba, and BL21/Cry5Ba) for 3 h, at 30°C. Worms were then washed twice with M9 buffer and mounted on 2% agarose pads for imaging using an Olympus BX51 fluorescence and phase contrast microscope (Olympus, Tokyo, Japan) at 20× magnification. Identical image settings were used for all treatment groups. More than 30 worms were analyzed per condition. DAF‐16::GFP localization was classified into three categories [65, 66, 67]: “nuclear”, characterized by predominant nuclear accumulation, with fluorescent puncta concentrated in intestinal nuclei along the body, and little residual cytoplasmic signal; “cytosolic”, characterized by diffuse cytoplasmic fluorescence, with no detectable nuclear enrichment; or “intermediate”, characterized by mixed nuclear and cytoplasmic localization, with fluorescence detected in both the cytoplasm and a subset of intestinal nuclei. Similar cytosolic/intermediate/nuclear classification schemes have been used in previous studies. For each experiment, the percentage of worms in each localization category was calculated blinded to treatment relative to the total number of worms scored. The percentage of worms exhibiting nuclear DFA‐16::GFP localization was used for statistical comparisons among treatments as previously reported [68]. Three independent experiments were performed.

### 4.9 Electrophoretic Mobility Shift Assays (EMSA)

A ∼300 bp fragment corresponding to the *pisP* promoter region was amplified by PCR using primers listed in Table S7 and labeled with 6‐carboxyfluorescein (FAM) fluorophore. The binding reactions contained ∼1.5 µM labelled DNA probe, 1 mM MnCl_2_, poly (dI: dC), varied concentrations of PlcR and PapR_7_, in binding buffer (10 mM Tris HCl, pH 8.0, 5% glycerol, 50 mM NaCl, 1 mM DTT, 50 µg/mL BSA). The reactions were incubated for 20 min at room temperature and resolved on 5% native polyacrylamide gels in 0.5×‐TBE buffer. Fluorescent signals were detected using a Typhoon phosphor imager (GE Healthcare).

### 4.10 Chromatin Immunoprecipitation and Sequencing

Chromatin immunoprecipitation (ChIP) was performed using a functional FLAG‐tagged PlcR strain. Cells from 50 mL aliquots were harvested at the indicated time points and stored at −80°C until processing. Pellets were washed and resuspended with CA buffer (10 mM Na_2_HPO_4_, 2 mM KH_2_PO_4_, 137 mM NaCl, and 2.7 mM KCl, pH 7.4), crosslinked with 1% formaldehyde for 20–30 min at room temperature, and quenched with 133 mM Glycine (pH 7.5) for 30 min at 4°C. Bacterial cells were collected by centrifugation, washed twice with CB buffer (50 mM Tris‐HCl, 150 mM NaCl, 1 mM EDTA, pH 7.4), and resuspended in 0.5 mL CB buffer. Cell lysis was achieved by incubation with 400 µg lysozyme and 1% Triton X‐100 for 1 h at room temperature, followed by sonication (Bioruptor, at 4°C for 20 min). After centrifugation, the supernatant was collected, and the total protein concentration was quantified using the Bradford assay. Aliquots containing 400 µg of total protein were stored at −80°C. An aliquot corresponding to 1% volume of the lysate was diluted with CB buffer and stored at −80°C to serve as the input control (1% of input DNA). For immunoprecipitation, anti‐FLAG M2 magnetic agarose beads (Sigma, Cat# M8823) were washed and resuspended in 400 µL CB buffer. Protein extracts containing 400 µg of total protein were incubated with the washed magnetic beads overnight at 4°C, with gentle rotation. The beads were subsequently recovered using a magnetic stand and washed twice with 500 µL of CB buffer. The protein‐DNA complexes were eluted using 3X‐FLAG peptide according to the manufacturer's instructions. All samples, including 1% input DNA, were reverse crosslinked overnight at 65°C as previously described. Co‐immunoprecipitated DNA was purified using a PCR purification Kit, quantified using a NanoDrop spectrophotometer, and used for library preparation with approximately 15 ng of immunoprecipitated DNA. Libraries were sequenced on an Illumina HiSeq X Ten platform. To generate 40‐bp single‐end reads. Sequencing reads were deposited in NCBI Sequence Read Archive (SRA, http://www.ncbi.nlm.nih.gov/Traces/sra) under the accession number PRJNA1187336. The ChIP‐qPCR validation was performed using appropriately diluted DNA and gene‐specific primers targeting the promoter regions of selected genes (Table S7).

### 4.11 ChIP‐Seq Data Analysis

Sequence reads were aligned to the Bt BMB171 reference genome (NCBI locus NC_014171.1) using Bowtie 2‐2.0.6, and processed using SAMtools [69]. Enriched regions were identified using MACS by comparing immunoprecipitated DNA samples against the corresponding input controls (1% input DNA). Peak annotation was performed using the R package ChIPSeeker. Significant peaks were defined as regions exhibiting ≥ 1.5‐fold enrichment relative to the input control, positive strand‐shift correlation, a q‐value < 0.01, and detection in at least two independent biological replicates. Genome‐wide visualization of ChIP‐seq signals was performed using the Integrative Genomics Viewer (IGV, version 2.3) [70]. Motif enrichment analysis was conducted on sequences extending ± 100 bp around peak summits using the Multiple Expectation Maximization for Motif Elicitation (MEME Suite) (Simple Enrichment Analysis; SEA) at https://meme‐suite.org/meme/tools/sea.

### 4.12 β‐Galactosidase Assay

Promoter‐lacZ transcriptional fusions were generated using the pHT304‐18z reporter vector and introduced into BMB171 and BMB171Δ*plcR* strains. Transformants were cultured on LB agar supplemented with Erythromycin and X‐Gal at 28°C for 12 h. For quantitative analysis, strains were grown in LB broth containing erythromycin at 28°C to an OD_600_ of 0.5–0.8. β‐Galactosidase activity was measured in Miller units as previously described. For qualitative visualization, cultures were plated on LB agar supplemented with Erythromycin and X‐gal and incubated at 28°C prior to imaging.

### 4.13 Rhodamine Labeling of PisP and Fluorescence Observation in *C. elegans*

Purified PisP was incubated with rhodamine dye (NHS‐Rhodamine, Thermo Scientific, Cat# 46406) at a dye‐to‐protein molar ratio of 10:1 for approximately 2 h at room temperature. Unconjugated dye was subsequently removed using a desalting column, and the rhodamine‐labeled protein was collected. *C. elegans* worms were synchronized and cultured to the L4‐stage. Groups of L4 worms were incubated with the same concentration of rhodamine‐labeled PisP or free rhodamine (control) for 4–6 h. Following incubation, the nematodes were thoroughly washed to remove residual free rhodamine or labeled protein from the exterior of the worms. Fluorescence microscopy was then performed to examine the distribution of the fluorescent signal within the nematodes.

### 4.14 Gene Deletion

The *plcR* gene was replaced with a spectinomycin resistance gene (*spc*) by homologous recombination. The upstream and downstream homologous arms of *plcR* were amplified from BMB171 genomic DNA, and the *spc* cassette was amplified from the spectinomycin‐resistant plasmid pBMB2062. These fragments were cloned into temperature‐sensitive plasmid pHT304Ts, and the resulting recombinant plasmid was transformed into BMB171. Transformants were selected on LB agar containing spectinomycin, and the temperature‐sensitive plasmid was subsequently cured by incubation at 42°C. Correct replacement of *plcR* with *spc* was verified by colony PCR and Sanger sequencing using gene‐specific primers (Figure S19).

The *pisp* and *papR* knockout mutants were constructed using the CRISPR‐Cas9 system based on plasmid pJOE8999, as previously described [71]. Single guide RNAs (sgRNAs) targeting *pisP* and *papR* were designed using CRISPy‐web [72]. The sgRNA cassettes were generated using gene‐specific primer pairs and cloned into pJOE8999 through the Eco31I restriction sites. The recombinant plasmids were transformed into *E. coli* DH5α and verified by PCR and Sanger sequencing. For each gene, upstream and downstream homologous arms of approximately 0.9–1 kb were amplified from BMB171 genomic DNA and fused by splicing by overlap extension PCR (SOE‐PCR) (Table S7). The resulting repair templates were inserted into the corresponding pJOE8999‐sgRNA constructs through the SfiI restriction sites, generating knockout plasmids containing both the sgRNA cassette and the corresponding repair template. The knockout constructs were introduced into BMB171 by electroporation. Transformants were cultured in LB medium at 28°C, and single colonies were screened by colony PCR using gene‐specific primer pairs (Table S7). Deletions of *pisp* and *papR* were further confirmed by Sanger sequencing (Figure S19). Sequencing reads spanning the mutated regions were aligned to the wild‐type BMB171 reference genome using SnapGene with default parameters and visualized in Jalview to confirm the precise deletion. Finally, the temperature‐sensitive pJOE8999 plasmid was cured by culturing the mutants at 42°C, and plasmid elimination was confirmed by antibiotic sensitivity screening and PCR using pJOE8999‐specific primers.

### 4.15 Isothermal Titration Calorimetry (ITC)

ITC experiments were performed using a Nano ITC instrument (TA Instruments, USA) to determine whether PapR_7_ influences the binding of PlcR to the PisP promoter. PlcR, the PapR_7_ peptide, and the PisP promoter DNA fragment were prepared in PBS buffer (pH 7.4; 8.0 g NaCl, 0.2 g KCl, 1.44 g Na_2_HPO_4_, and 0.24 g KH_2_PO_4_ per liter). All titrations were performed at 25°C with continuous stirring at 250 rpm. To ensure consistency among titration experiments, the concentration of the PisP promoter DNA fragment was fixed at 0.67 µM (the maximum concentration achievable after purification). Accordingly, the concentration of PlcR was maintained at 0.05 µM in all experiments. Before ITC measurements, PlcR was incubated with PapR_7_ at the indicated molar ratios on ice for 2 h to allow complex formation. The sample cell contained PlcR alone, PapR alone, or the pre‐incubated PlcR‐PapR_7_ complex, whereas the syringe contained the PisP promoter DNA fragment. Each experiment consisted of 25 injections of 2 µL at 150 s intervals. Raw thermograms were analyzed using NanoAnalyze software. Binding isotherms were fitted using an independent 1:1 binding model to determine the dissociation constant (*K*d), binding stoichiometry (*N*), enthalpy change (*ΔH*), entropy change (*ΔS*), and standard deviation of the fit.

### 4.16 Yeast Two‐Hybrid Assay

Using the primers listed in Table S7, the coding regions of PisP and the candidate target proteins (DAF‐16, AKT‐1, PDK‐1, AGE‐1, INS‐1, INS‐18, INS‐39, and PAR‐1) were amplified by PCR and cloned into the pGBKT7 and pGADT7 vectors, respectively, using the ClonExpress II One Step Cloning Kit (Vazyme, Cat No. C112). The resulting bait plasmid, pGBKT7‐PisP, and each corresponding prey plasmid, pGADT7‐target, were co‐transformed into *Saccharomyces cerevisiae* strain AH109. Transformants were selected on synthetic dropout (SD) medium lacking tryptophan and leucine (SD/‐Trp/‐Leu) at 30°C for 3 days. Single colonies were inoculated into SD/‐Trp/‐Leu liquid medium and cultured to the same OD_600_. Cells were collected by centrifugation at 5000 × g for 30 s, washed once with sterile water, and resuspended in 1 mL sterile water. Ten‐fold serial dilutions (10^−1^, 10^−2^, and 10^−3^) were prepared, and 3 µL of each dilution was spotted onto SD/‐Trp/‐Leu medium and SD medium lacking tryptophan, leucine, adenine, and histidine (SD/‐Trp/‐Leu/‐Ade/‐His). Plates were incubated at 30°C for 3–5 days and photographed.

The pGBKT7‐53 (BD‐p53) and pGADT7‐T (AD‐SV40 large T antigen) pair was used as the positive control, whereas the pGBKT7‐Lam (BD‐lamin C) and pGADT7‐T (AD‐SV40 large T antigen), pGBKT7‐PisP and pGADT7 pair, and pGBKT7 and pGADT7‐prey pair were used as the negative controls. Growth on SD/‐Trp/‐Leu plates confirmed yeast transformation and maintenance of both plasmids, whereas growth on SD/‐Trp/‐Leu/‐Ade/‐His plates was used to assess protein–protein interactions.

### 4.17 Structure Prediction of PisP and Structural Comparison

The three‐dimensional structure of PisP was predicted using AlphaFold 3. Experimentally determined structures of representative patatin‐like phospholipases, including ExoU (PDB: 3TU3) and VipD (PDB: 4AKF), were retrieved from the Protein Data Bank for comparative analyses. Structural alignments were performed using the “align” function in PyMOL (version 3.1.0). Structural similarity was evaluated by calculating root‐mean‐square deviation (RMSD) values based on aligned Cα atoms. Conserved and divergent structural regions were identified from the resulting alignments and visualized using PyMOL.

### 4.18 Pesticidal Protein Repertoire and Potential Target Spectrum Analysis

The pesticidal protein repertoires of 889 Bt genomes were analyzed using Bt_toxin_digger [73]. For each genome, predicted pesticidal proteins were identified and assigned to their corresponding protein families or major pesticidal protein categories. The number and composition of pesticidal proteins were recorded for each genome and compared between PisP‐positive and PisP‐negative strains. The complete dataset, including the pesticidal proteins identified in each Bt genome and their corresponding classifications, is provided in Table S5.

To further characterize the potential pesticidal target spectrum of Bt strains, experimentally reported target information for individual pesticidal proteins carried by 889 Bt strains was compiled from the published literature and the Bacterial Pesticidal Protein Resource Center (BPPRC http://www.bpprc‐db.org/) (Table S8). For each pesticidal protein, documented susceptible host taxa were manually curated and assigned primarily at the taxonomic order level, with representative pest species retained to illustrate the major target groups within each order. The curated protein–target associations were then mapped to the pesticidal protein repertoires of the 889 Bt genomes to infer the potential target spectra of individual strains. The resulting strain‐level inferred target spectra are provided in Table S5.

The inferred target spectrum represents predictions based on the presence of pesticidal proteins with previously reported activity against members of a given taxonomic group and should not be interpreted as experimentally validated host range. The presence of a pesticidal protein in a genome does not necessarily indicate that the protein is expressed, properly produced or processed, biologically active, or sufficient to confer toxicity under a given assay condition. Therefore, these assignments are referred to as “potential target spectra” throughout the manuscript.

### 4.19 Distribution of PisP‐Like Proteins Across Bacterial Species

To investigate the distribution of PisP‐like proteins beyond the Bt genome, protein sequence datasets were compiled from NCBI. The dataset included 12 bacterial species or taxa representing members of the *Bacillus* group and other host‐associated or pathogenic bacteria: *B. cereus* (2501 genomes), *B. subtilis* (1268), *B. mycoides* (266), *B. cytotoxicus* (87), *B. pumilus* (385), *B. toyonensis* (378), *B. velezensis* (912), *B. wiedmannii* (279), *Brevibacillus laterosporus* (72), *Lysinibacillus sphaericus* (53), *Paenibacillus larvae* (80), and *Serratia marcescens* (1801). Protein sequences predicted from these genomes were searched against the PisP reference protein using BLASTP.

Candidate PisP‐like proteins were initially identified based on query coverage and amino acid sequence identity. Two sequence‐similarity criteria were applied to distinguish closely related PisP homologs from more distantly related PisP‐like proteins. The stringent criterion required ≥ 80% query coverage and ≥ 80% amino acid sequence identity, whereas the relaxed criterion required ≥ 80% query coverage and ≥ 25% amino acid sequence identity. Proteins identified using either criterion were subsequently analyzed using the NCBI Conserved Domain Database (CD‐Search; https://www.ncbi.nlm.nih.gov/Structure/cdd/wrpsb.cgi) to verify the presence of the characteristic PisP‐associated patatin/cPLA2 domain.

Proteins meeting the respective sequence‐similarity criteria and retaining the characteristic patatin/cPLA2 domain were considered PisP‐like proteins and included in the downstream distribution analysis (Table S6). For each bacterial species, the number and proportion of genomes containing at least one qualifying PisP‐like protein were calculated. Results obtained using the stringent and relaxed criteria were analyzed separately to distinguish closely related PisP homologs from more distantly related PisP‐like proteins (Table S6).

### 4.20 Quantification and Statistical Analysis

All experiments were performed with at least three independent biological replicates unless otherwise indicated. Statistical analyses were conducted using SPSS (version 13.0; IBM Chicago, IL, USA) or GraphPad Prism (v9.0). LC50 values were estimated by Probit analysis, and dose ‐response curves were generated by nonlinear regression analysis. Comparisons between two groups were performed using a two‐tailed Student´s *t*‐test. Comparisons among multiple groups were analyzed by one‐way or two‐way ANOVA followed by Tukey´s multiple‐comparison test. Data are presented as means ± SD unless otherwise indicated. Statistical significance was defined as follows: ns, not significant; *p* < 0.05; *p* < 0.01; *p* < 0.001; and *p* < 0.0001.

## Author Contributions

Z.L., F.C., L.W., L.C., M.S., K.W., J.Z., and D.P. conceived and designed the study. Z.L., F.C., L.W., B.Z., and L.C. performed the experiments. Z.L., F.C., B.Z., F.L., H.L., D.D., and L.L. performed data analysis, bioinformatics analysis, and visualization. Z.L., F.C., K.W., M.S., A.B., and D.P. drafted and revised the manuscript. J.Z., D.P., and M.S. provided study supervision, project administration, and funding acquisition.

## Funding

This work was supported by grants from the National Key Research and Development Program of China (grant no. 2024YFD1200205 to D.P.) and the National Natural Science Foundation of China (grant no. 32470071 and 31970075 to D.P.).

## Conflicts of Interest

The authors declare no conflicts of interest.

## Supporting information




**Supporting File**: advs78451‐sup‐0001‐SuppMat.pdf.

## Data Availability

The ChIP‐Seq data are available on the NCBI Gene Expression Omnibus (GEO) (https://www.ncbi.nlm.nih.gov/geo/), under the record PRJNA1187336.
