# Supplementary material for: A Novel Immunosuppressive Effector Functions as a Bio‐Adjuvant to Enhance the Activity of Bacillus Thuringiensis and Chemical Insecticides
Source: Adv Sci (Weinh). 2026 Nov 10:e78451. Online ahead of print. doi: 10.1002/advs.78451 (PMC13752580; doi:10.1002/advs.78451)
Supplement: Supplementary file 1 [file ADVS-9999-e78451-s001.pdf]

Supplementary Materials for

A Novel Immunosuppressive Effector Functions as a Bio-Adjuvant to Enhance the Activity of *Bacillus thuringiensis* and Chemical Insecticides

Zhiyu Liu[#], Feng Chen[#], Liting Wan, Li Chen, Bowen Zhang, Fanling Li, Haixin Luo, Lixu Li, Alejandra Bravo, Mario Soberón, Dadong Dai, Jinshui Zheng, Ming Sun, Kai Wang,[*] Donghai Peng,[*]

[#]These authors contributed equally to this work.

Z. Liu, L. Wan, L. Chen, B. Zhang, F. Li, H. Luo, D. Dai, J. Zheng, M. Sun, K. Wang, D. Peng

National Key Laboratory of Agricultural Microbiology

Huazhong Agricultural University

Wuhan, Hubei 430070, China

E-mail: wangkai325@webmail.hzau.edu.cn; donghaipeng@mail.hzau.edu.cn

A. Bravo, M. Soberón

Instituto de Biotecnología

Universidad Nacional Autónoma de México, Apdo

postal 510-3, Cuernavaca, Morelos 62250, México

F. Chen, L. Li

Guangxi Institute of Botany

Chinese Academy of Sciences
Guilin 541006, China

Supplement Figures and Tables

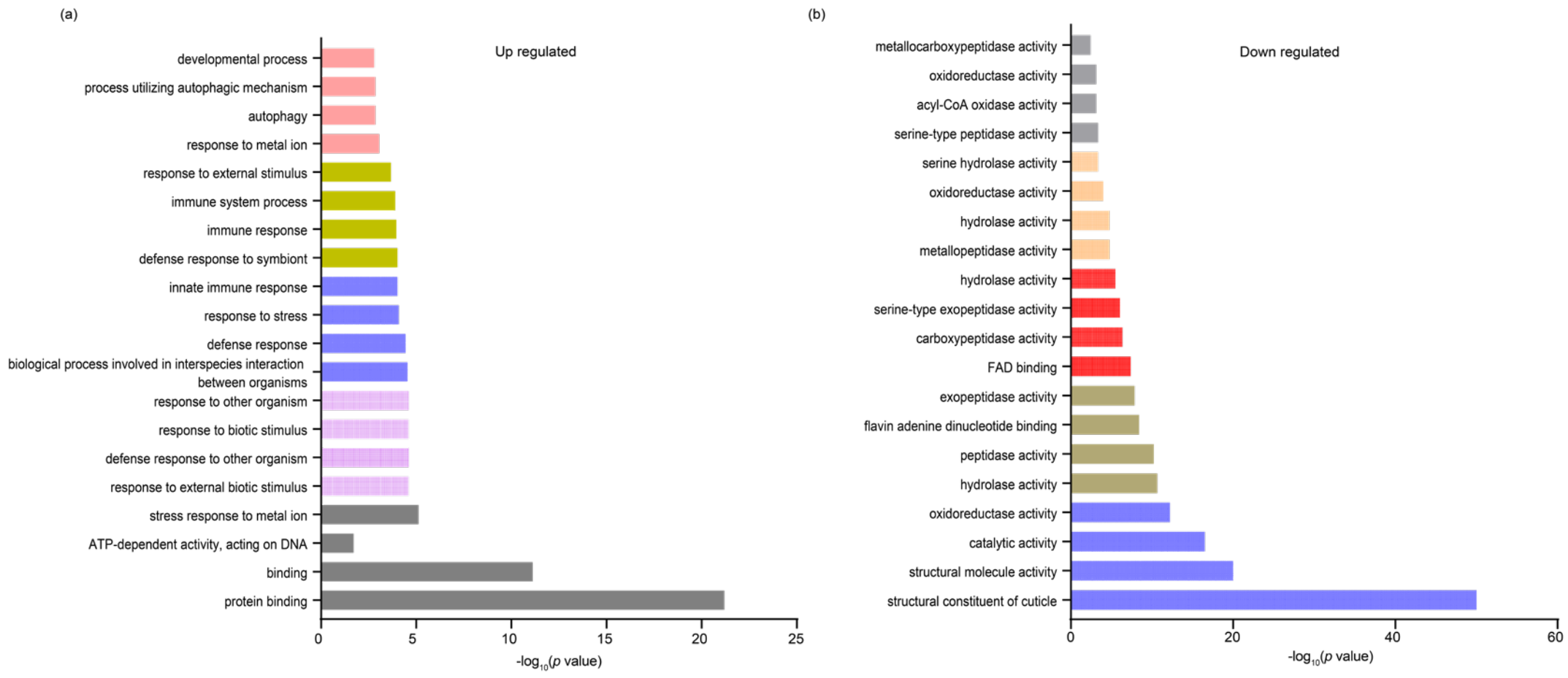

Figure S1. Gene Ontology (GO) enrichment analysis of differentially expressed genes in *C. elegans* upon BMB171/Cry5Ba infection.

(A) Top enriched GO terms among genes significantly upregulated in *C. elegans* following BMB171/Cry5Ba treatment. **(B)** Top enriched GO terms among significantly downregulated genes under the same conditions. Differentially expressed genes (DEGs) were defined with adjusted $p < 0.05$ and |log2 fold change| ⩾ 1. In both panels, the y-axis indicates the enriched GO terms, including biological processes or molecular functions, and the x-axis indicates enrichment significance, expressed as -log10 (Benjamini-Hochberg adjusted p-value/ FDR<0.05).

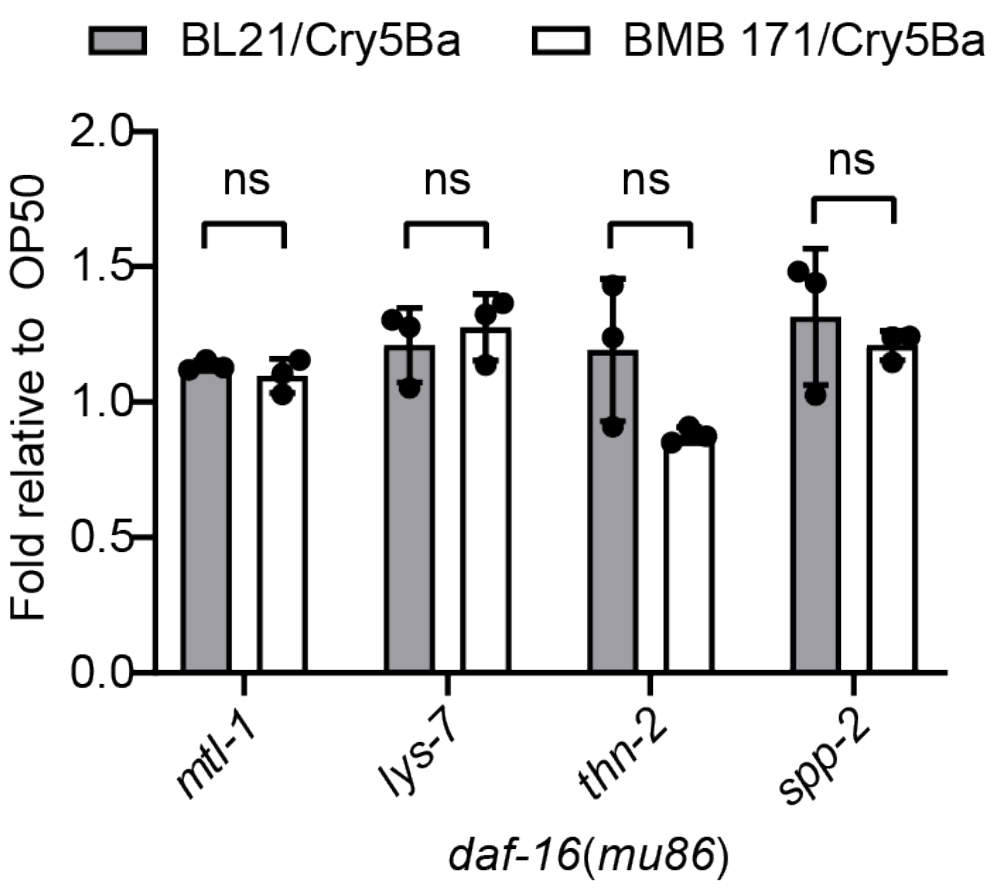

Figure S2. DAF-16 dependence of immune defense gene suppression during Bt infection.

Relative mRNA levels (fold change) of *mtl-1*, *lys-7*, *thn-2*, and *spp-2* in *daf-16(mu86)* mutant worms exposed to BL21/Cry5Ba or BMB171/Cry5Ba. Transcription levels were determined by qRT-PCR and normalized to those of the OP50 control (set to 1). No significant differences were observed between the two treatments for any of the genes examined, supporting the involvement of functional DAF-16 in the differential regulation of these immune defense genes. Data are presented as mean ± SD, with individual biological replicates shown as dots, $n = 3$. Statistical significance was determined using unpaired t-tests (ns, not significant difference).

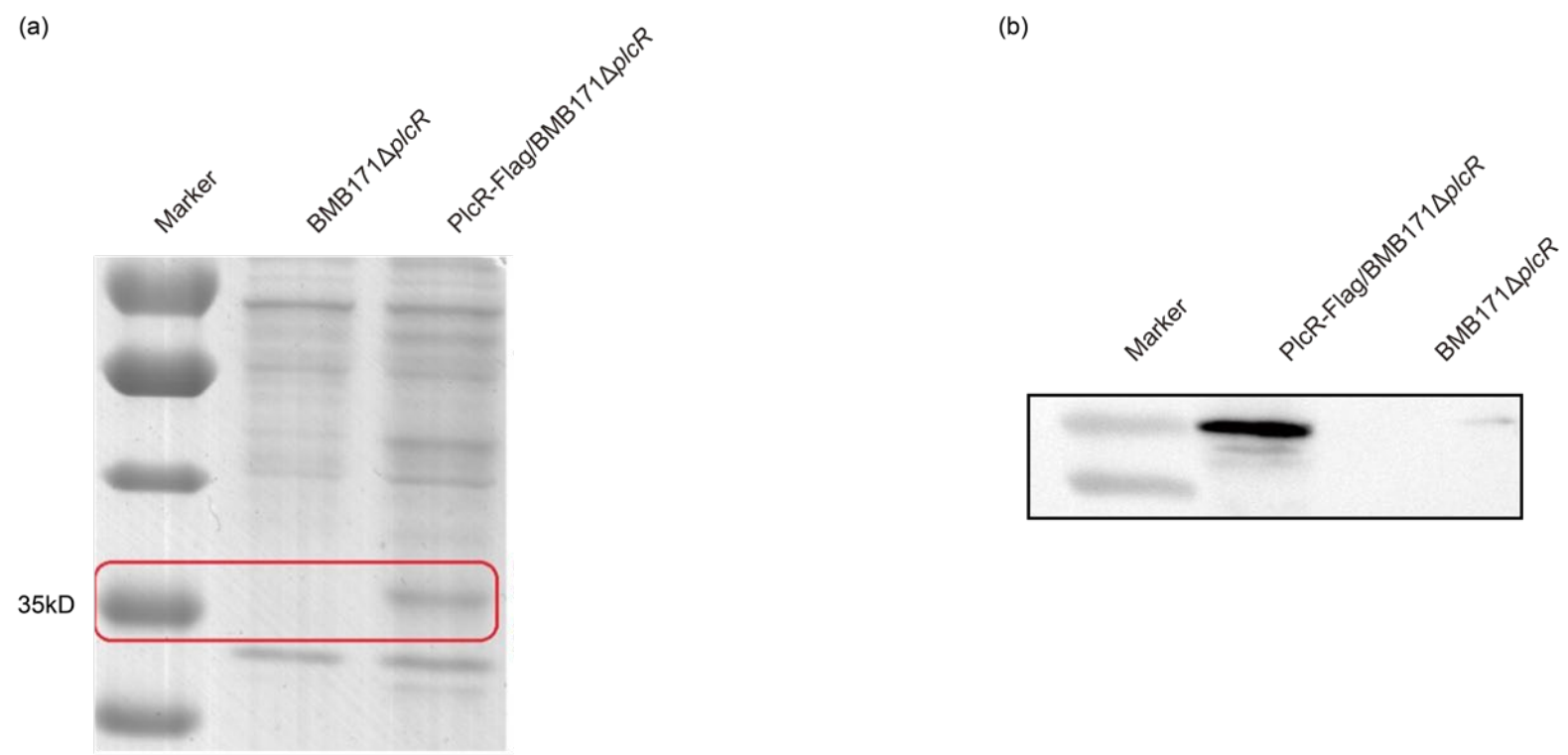

Figure S3. Validation of PlcR-FLAG expression in the engineered BMB171 strain. **(A)** SDS-PAGE analysis of total cellular proteins stained with Coomassie Brilliant Blue. The red boxed region indicates the region containing the protein band corresponding to PlcR-FLAG (~ 35 kDa), which was detected in the complemented strain (PlcR-FLAG/BMB171 △*plcR*) but was absent in the *plcR* deletion mutant (BMB171 △*plcR*). **(B)** Western blot analysis of the same samples using an anti-FLAG antibody. A specific signal band corresponding to the PlcR-FLAG/BMB171 △*plcR* strain, confirming expression of the FLAG-tagged PlcR protein used for subsequent ChIP-seq analysis.

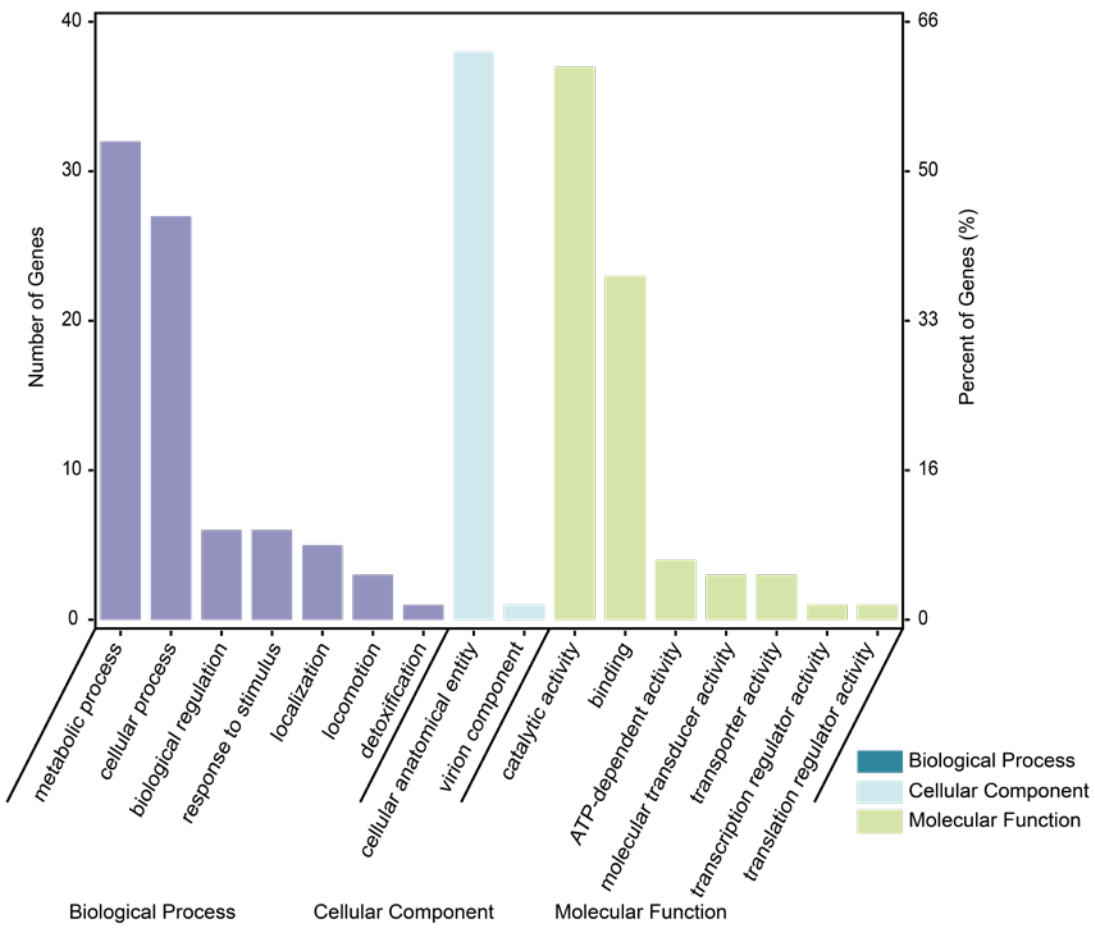

Figure S4. Gene Ontology (GO) functional classification of PlcR target genes. Functional classification of the 74 direct PlcR target genes identified by ChIP-seq in BMB171 according to the three primary GO categories: Biological Process, Cellular Component, and Molecular Function. The left y-axis indicates the number of genes assigned to each GO term, whereas the right y-axis indicates the corresponding percentage of of the 74 annotated PlcR target genes. The x-axis shows individual GO subcategories.

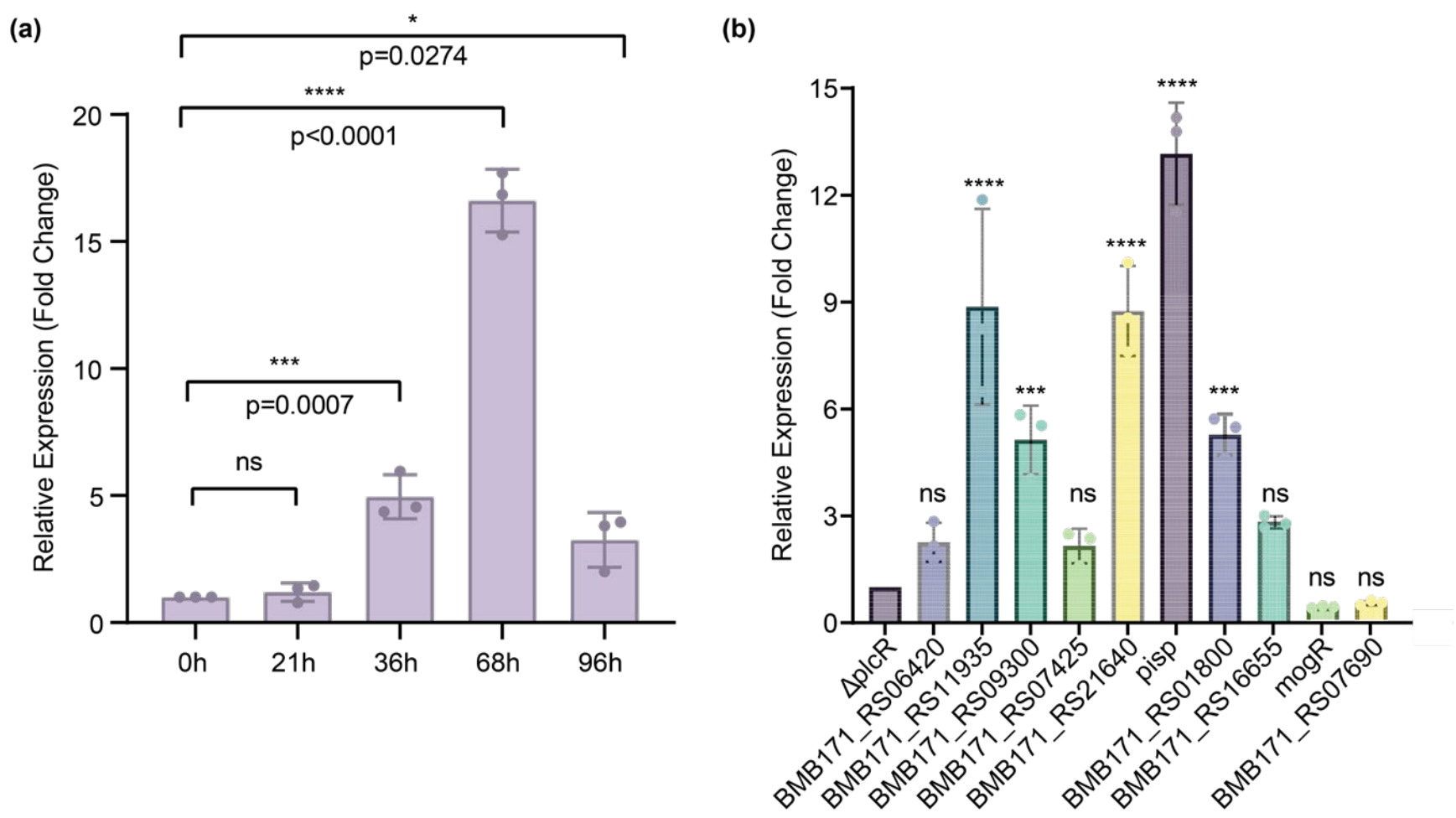

Figure S5. Transcriptional analysis of PlcR and PlcR target genes during Bt infection of *C. elegans*.

(A) Temporal expression of *plcR* in the wild-type BMB171 strain during infection. Relative *plcR* mRNA levels (fold change) were determined by qRT-PCR at the indicated time points (21, 36, 68, and 96 h post-inoculation) and normalized to the 0 h baseline, which was set to 1. Expression levels were normalized to the 0 h time point baseline (set to 1). The *plcR* transcript levels increased progressively over time. **(B)** Relative mRNA levels (fold change) of selected PlcR target genes in the BMB171 strain compared to the BMB171*ΔplcR* mutant. Total RNA was extracted at 68 h post-inoculation, and transcript levels were determined by qRT-PCR and normalized to those in the Δ*plcR* mutant, which were set to 1. Among the PlcR target genes examined, *pisP* gene showed the highest PlcR-dependent increase in transcript abundance. All qRT-PCR transcript data were normalized to the internal reference gene *16S rRNA*. Data are presented as mean ± SD, with individual biological replicates shown as dots. n = 3 biological replicates. Statistical significance was determined by one-way ANOVA or unpaired t-test, as appropriate. *P < 0.05, **P < 0.01, ***P < 0.001, ****P < 0.0001; ns, not significant difference.

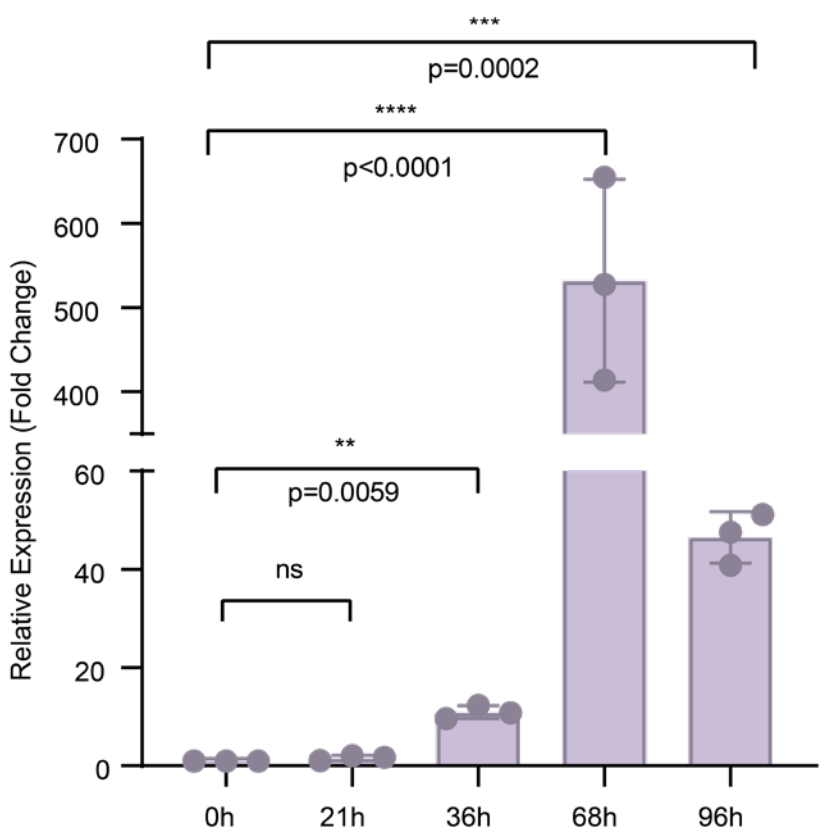

Figure S6. Transcriptional analysis of *papR* during the Bt infection of *C. elegans*. Relative mRNA levels (fold change) of *papR* in BMB171 strain were determined by qRT-PCR at the indicated time points (21, 36, 68, and 96 h post-inoculation) and normalized to the 0 h baseline which was set to 1. The transcript levels of *papR* increased progressively over time, reaching a maximum at 68 h (high bacterial density), and subsequently decreased at 96 h when host death and bacterial sporulation were observed. All qRT-PCR transcript data were normalized to the internal reference gene *16S rRNA*. Data are presented as mean ± SD, with individual biological replicates shown as dots. n=3 biological replicates. Statistical significance was determined by one-way ANOVA. **P < 0.01, ***P < 0.001, ****P < 0.0001; ns, not significant difference.

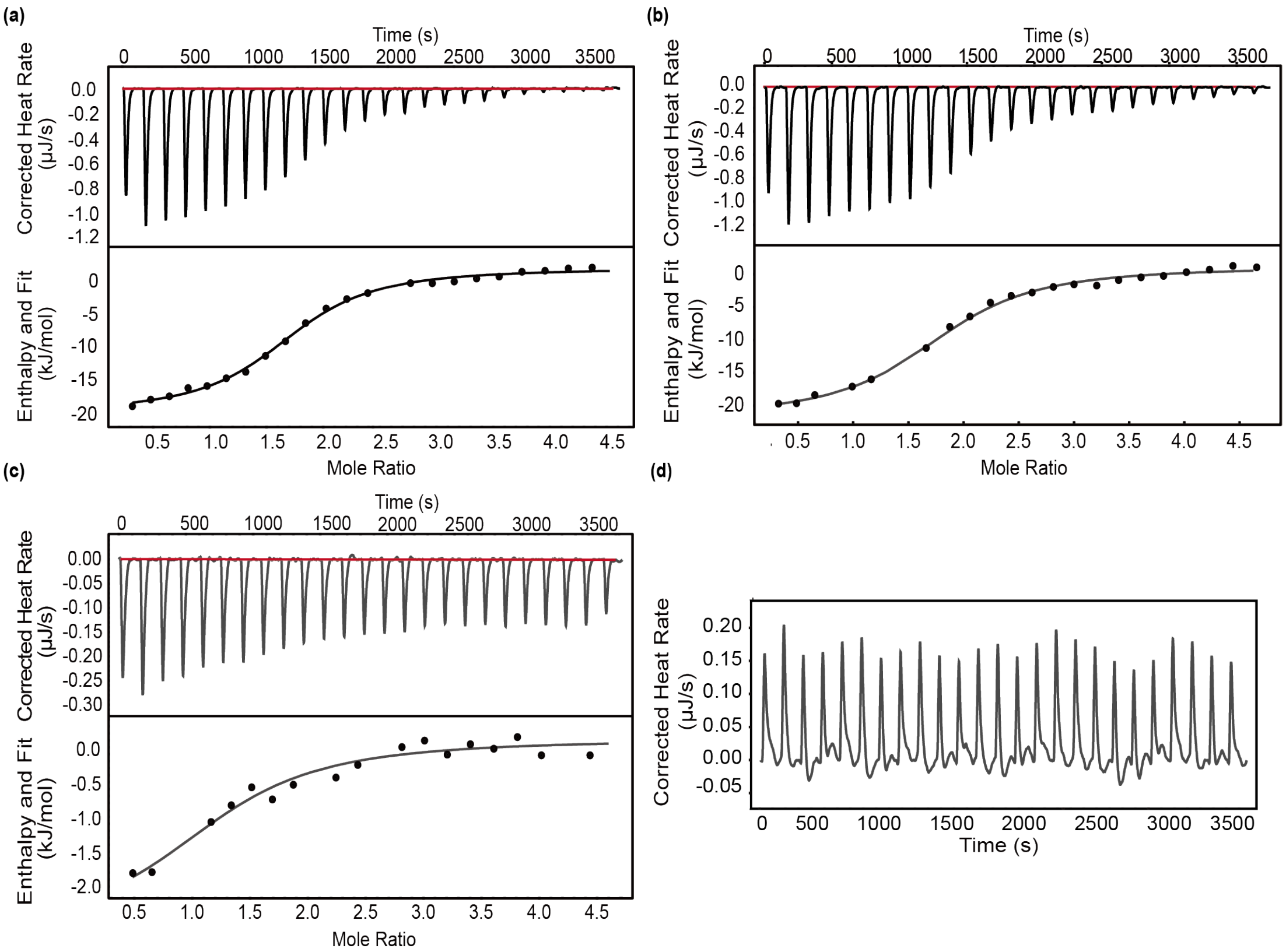

(e)

Sample	*Kd*(M)	*ΔH*(kJ/mol)	*ΔS*(J/mol·K)	*n*	Standard Deviation Around Fit
PlcR-PapR$_7$(1:1)	4.915E-6±1.267E-6	-22.01±1.140	27.820	1.702±0.044	0.437
PlcR-PapR$_7$(1:0.5)	6.085E-6±2.124E-6	-23.11±1.720	22.330	1.757±0.065	0.791
PlcR	3.045E-5±7.471E-5	-4.766±45.140	70.480	0.813±0.752	0.830

Figure S7. Isothermal Titration Calorimetry (ITC) analysis of PlcR-PapR$_7$ binding to the *pisP* promoter.

Representative ITC thermograms (upper panels) and corresponding integrated binding isotherms (lower panels) for the interaction between PlcR and the *pisP* promoter DNA in the presence of different PapR$_7$ molar ratios: **(A)** 1:1 ratio, **(B)** 1:0.5 ratio, and **(C)** PlcR alone (1:0 ratio). **(D)** Negative control using PapR$_7$ alone in the absence of PlcR, showing no detectable specific interaction with the *pisP* promoter DNA. **(E)** Comparison of the dissociation constants (K_D) determined under the different conditions. The highest DNA-binding affinity was observed at 1:1 PlcR: PapR$_7$ molar ratio, supporting a PapR$_7$-dependent enhancement of PlcR binding to the *pisP* promoter. Details of this procedure are described in the Experimental section in the main text of this work.

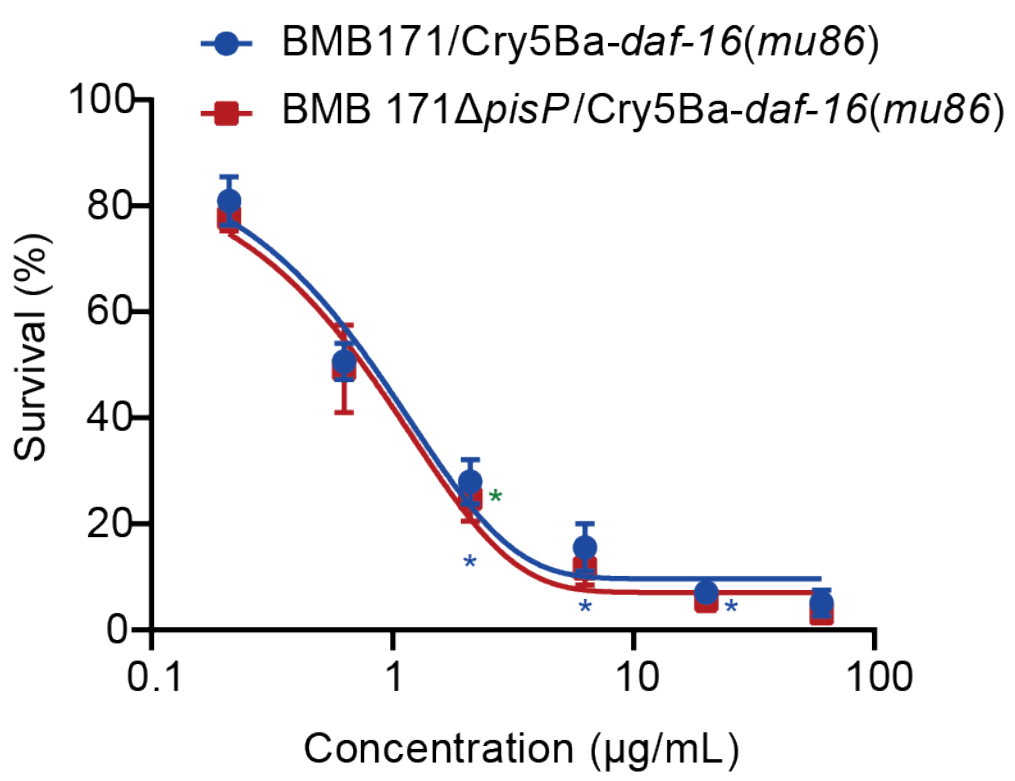

Figure S8. DAF-16 is required for PisP-mediated enhancement of Bt virulence in *C. elegans*.

Dose-response toxicity assays in *daf-16*(*mu86*) mutant worms exposed to BMB171/Cry5Ba or BMB171Δ*pisP*/Cry5Ba. Data are presented as mean ± SD from three independent experiments, each performed in triplicate with > 30 worms per condition. Statistical significance was determined by an unpaired t-test. *$p < 0.05$.

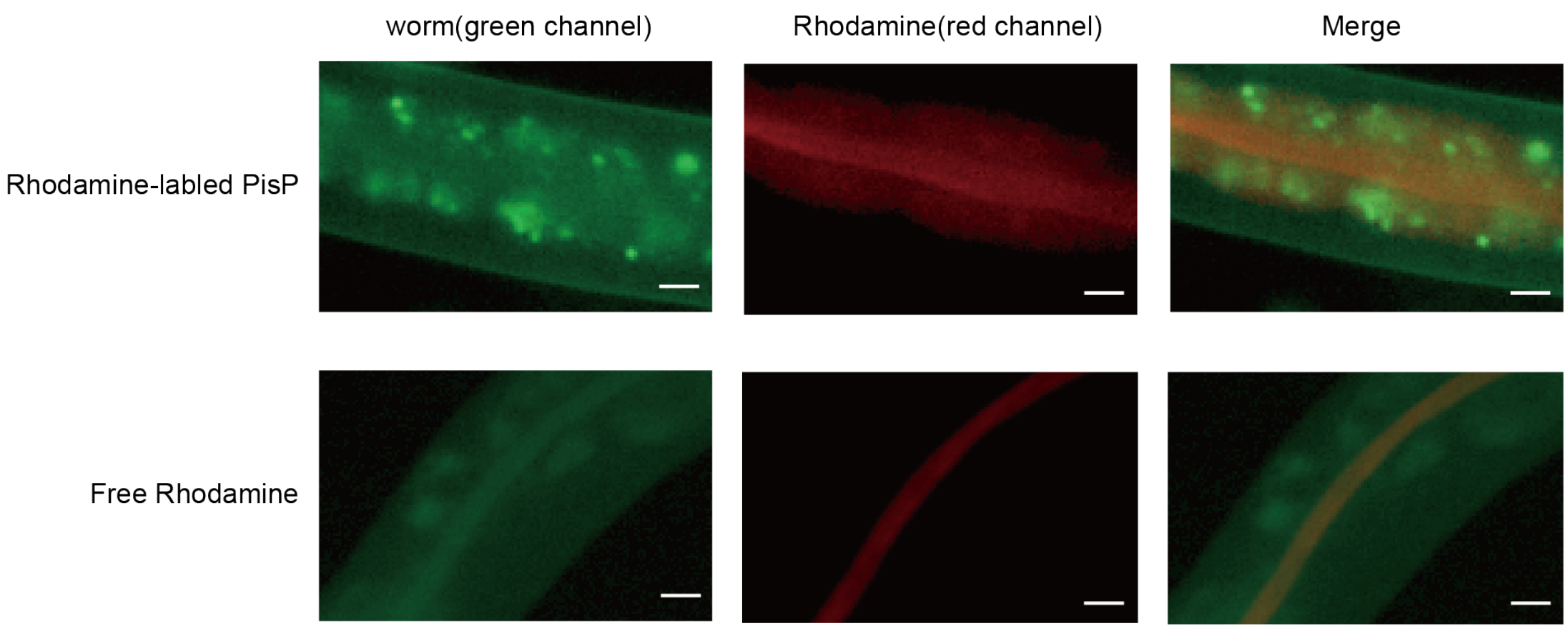

Figure S9. Uptake of Rhodamine-labeled PisP by intestinal cells in *C. elegans*. L4-stage *C. elegans* were fed rhodamine-labeled PisP (top row) or free rhodamine (bottom row) at a final concentration of 2 μM for approximately 4-6 h and examined by fluorescence microscopy. Green channel: worm tissue; red channel: rhodamine signal; Merge: overlay of green and red channels. In worms fed with rhodamine-labeled PisP, the red fluorescent signal was detected in the intestinal tract and extended beyond the intestinal lumen into both sides of the surrounding intestinal tissue, consisting with uptake of labeled PisP by intestinal cells. In contrast, fluorescence from free rhodamine remained restricted to the intestinal lumen under the same conditions. Scale bars: 5 μm. These observations support the ability of PisP to cross the intestinal lumen boundary and enter intestinal tissue. Data are representative of 3 independent experiments, with a total of >10 worms examined.

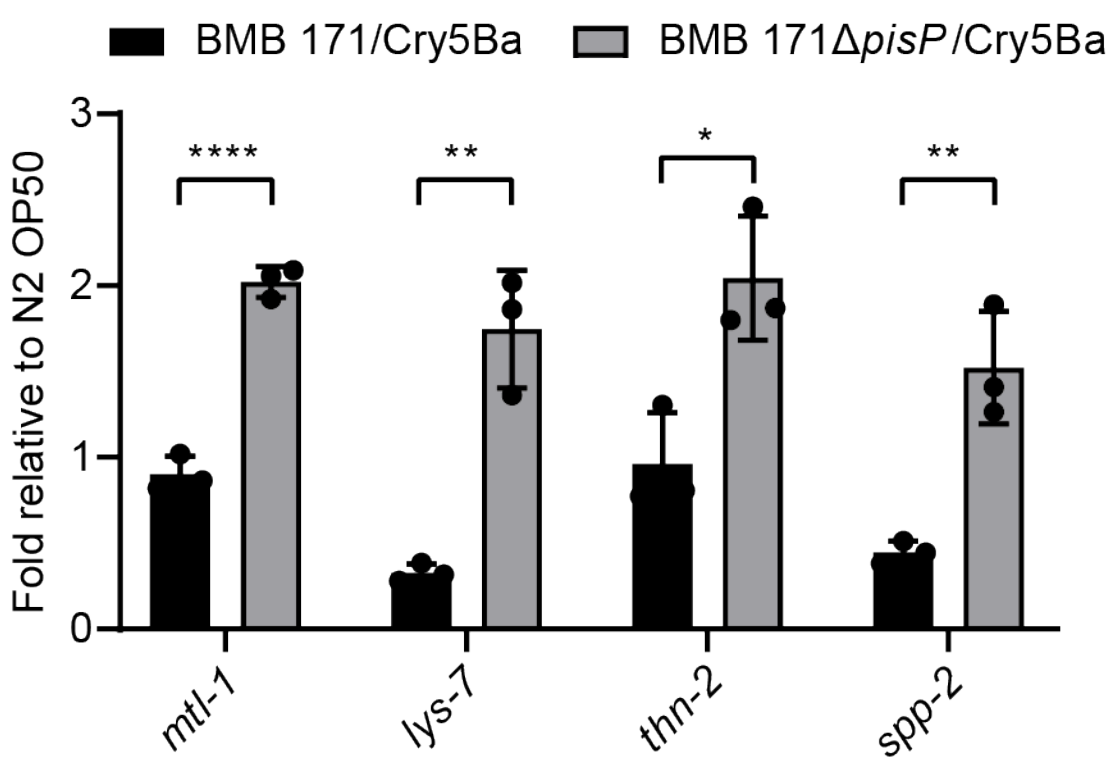

Figure S10. PisP suppresses DAF-16-associated immune defense genes in *C. elegans* during Bt infection.

Relative mRNA levels (fold change) of *mtl-1*, *lys-7*, *tnn-2*, and *spp-2* in wild-type N2 worms treated with BMB171/Cry5Ba or BMB171Δ*pisP*/Cry5Ba for 4 h at 25 ℃. Transcript levels were determined by qRT-PCR and normalized to those in the N2 worms fed OP50, which were set to 1. The expression of all four genes was significantly lower in worms treated with BMB171/Cry5Ba than in those treated with BMB171Δ *pisP*/Cry5Ba, supporting a role for PisP in suppressing DAF-16-associated immune defense genes during Bt infection. All qRT-PCR transcript data were normalized to the internal reference gene *tba-1*. Data are presented as mean ± SD, with individual biological replicates shown as dots. n=3. Statistical significance was determined using unpaired t-tests. *P < 0.05, **P < 0.01, ***P < 0.001, ****P < 0.0001; ns, not significant difference.

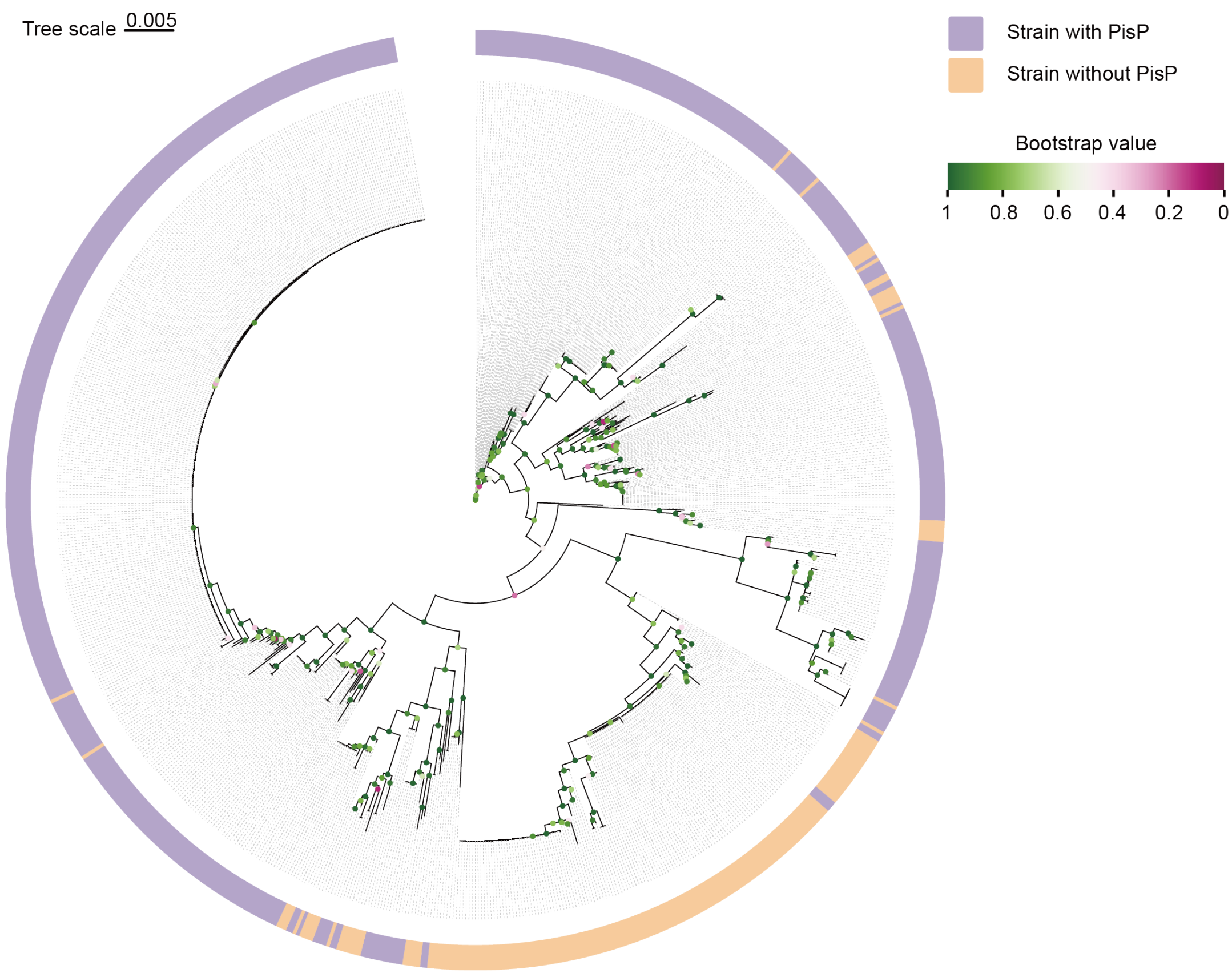

Figure S11. Widespread distribution of PisP among Bt strains.

A maximum likelihood (ML) phylogenetic tree of 889 Bt genomes was constructed using PhyML based on concatenated protein sequences of single-copy core genes and the best-fit amino acid substitution model (JTT+I+G). Branch support was assessed using 1,000 bootstrap replicates. The outer ring indicates the presence (lavender) or absence (apricot) of PisP homolog in each strain.

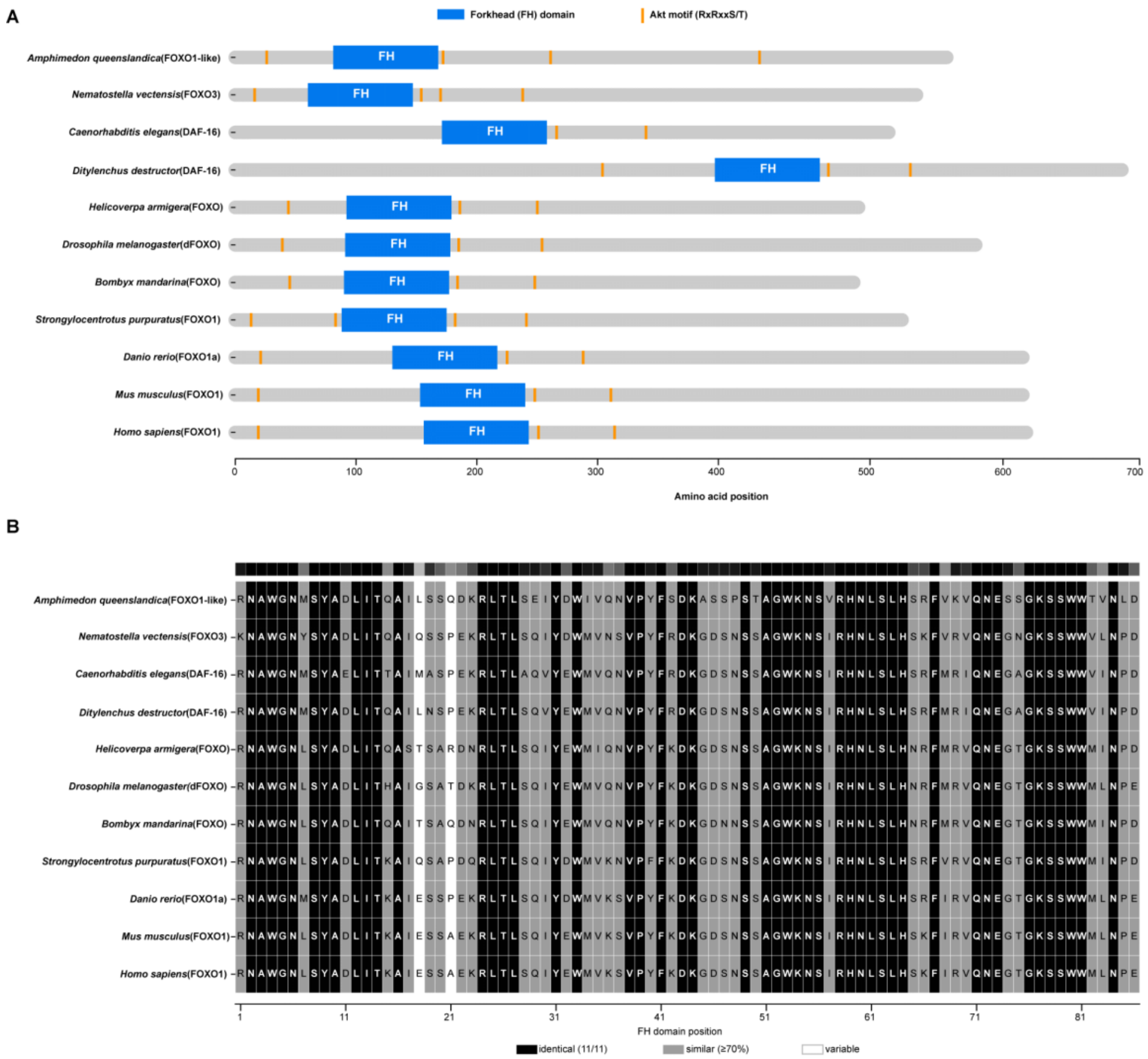

Figure S12. Evolutionarily conservation of DAF-16/FOXO proteins across metazoans.

(A) Domain architecture of DAF-16/FOXO orthologs from 11 metazoan species representing six phyla (Porifera, Cnidaria, Nematoda, Arthropoda, Echinodermata, and Chordata), shown to scale. The forkhead (FH) DNA-binding domain is shown in blue, and orange ticks indicate predicted Akt phosphorylation motifs (RxRxxS/T) associated with regulation of DAF-16/FOXO subcellular localization. Both the FH domain and the predictedAkt-regulatory motifs are conserved across all species examined, including *Caenorhabditis elegans* and pest species *Ditylenchus destructor*, *Helicoverpa armigera, and Spodoptera frugiperda*. **(B)** Multiple sequence alignment of the 86-amino-acid FH domain from the same 11 species. Black background shading indicates residues identical across all 11 species; gray background indicates conserved

substitutions (≥70% similarity based on BLOSUM62); and white background indicates variable positions. The grayscale track above the alignment represents sequence identity at each position, with darker shading indicating greater conservation. The FH domain shares 79-94% sequence identity among the three pest species mentioned above and 71-100% sequence identity across the 11 metazoan species analyzed, demonstrating strong evolutionary conservation of the DAF-16/FOXO DNA-binding domain.

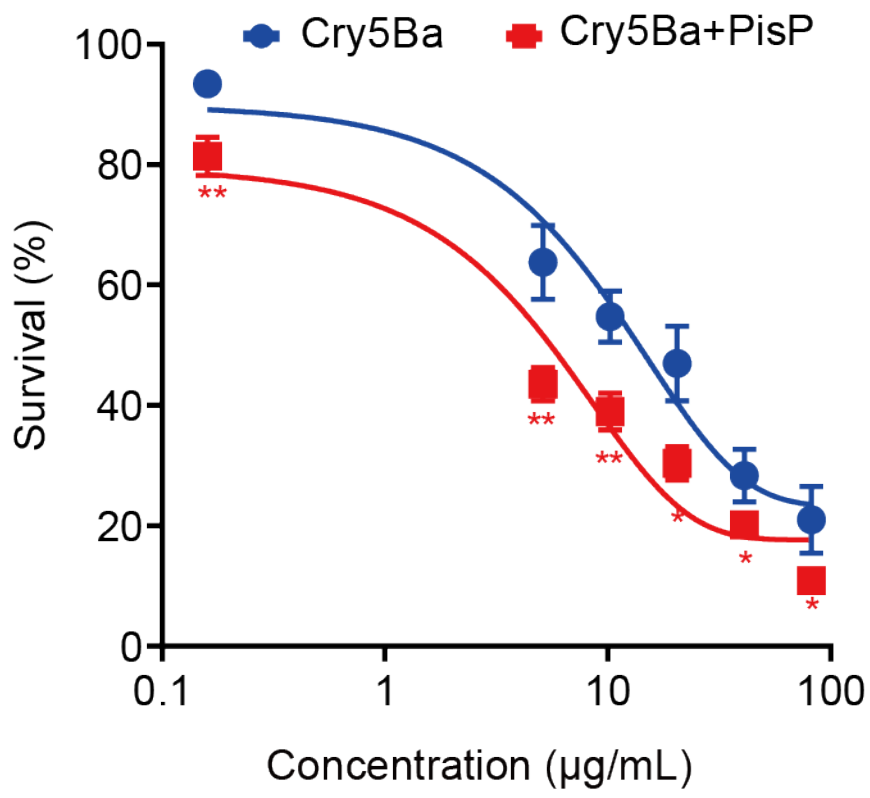

Figure S13. PisP enhances Cry5Ba toxicity against *C. elegans*.

Dose-response toxicity assays of purified Cry5Ba toxin alone (0-100 μg/mL), or in combination with purified PisP (200 μg/mL) against in wild-type *C. elegans* (N2) for 120 h. Data are presented as mean ± SD from three independent experiments, each performed in triplicate with > 30 worms per condition. Statistical significance was determined by unpaired t-tests. $^*p < 0.05$, $^{**}p < 0.01$.

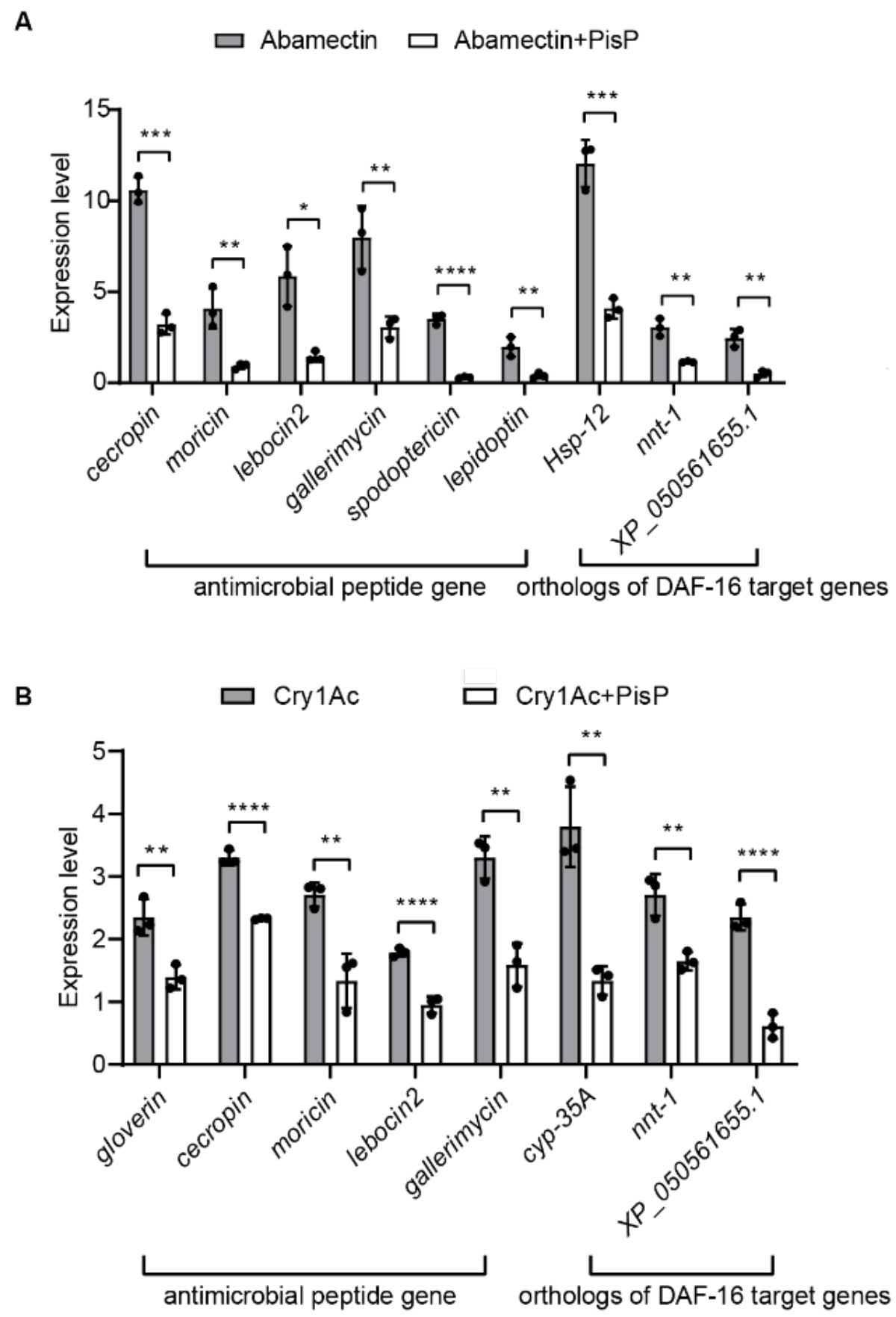

Figure S14. PisP suppresses the expression of antimicrobial peptide genes and orthologs FOXO/DAF-16 target genes in *S. frugiperda*.

(A, B) *S. frugiperda* larvae were fed an artificial diet containing 0.06 μg/g abamectin (A) or 0.05 μg/g Cry1Ac (B) alone or in combination with PisP (200 μg/mL) for 48 h. Larvae fed on an untreated diet served as controls. Relative transcript levels of representative antimicrobial peptide genes and *S. frugiperda* orthologs of FOXO/DAF-16 target genes were determined by qRT-PCR and normalized to those in the untreated control, which were set to 1. Co-treatment with PisP reduced the transcript levels of these genes compared with abamectin or Cry1Ac alone. All qRT-PCR transcript data were normalized to the internal reference gene *RPL18*. Data are presented as mean ± SD, with individual biological replicates shown as dots. n=3. Statistical significance was determined using unpaired t-tests. *P < 0.05, **P < 0.01, ***P < 0.001, ****P < 0.0001; ns, not significant difference.

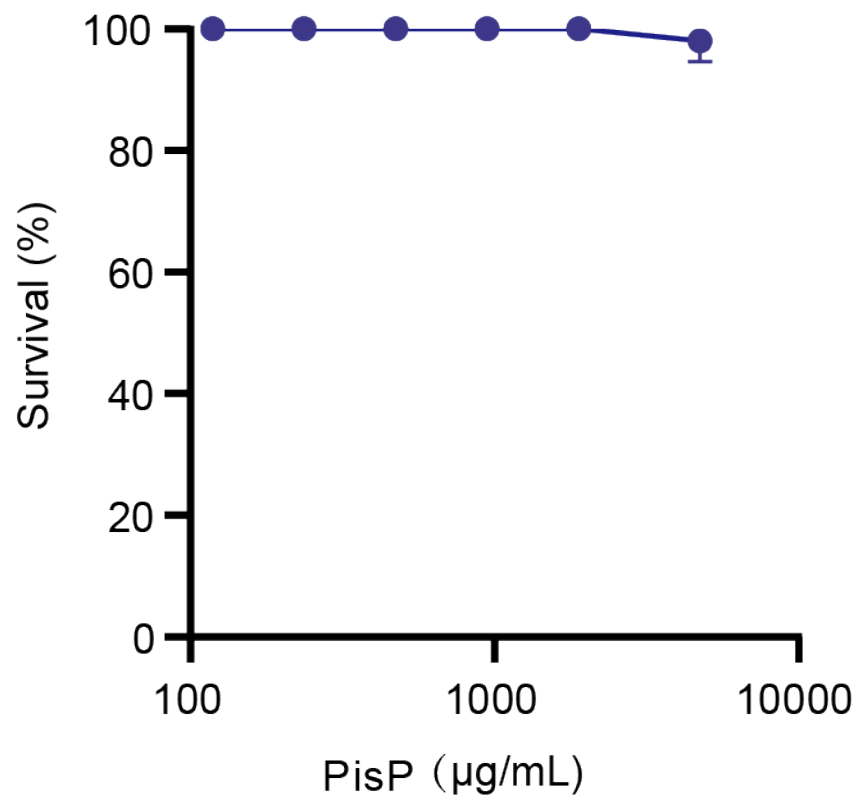

Figure S15. PisP exhibits no detectable toxicity when administered alone to *C. elegans*.

Dose-response toxicity assays were performed in wild-type *C. elegans* (N2) treated with purified PisP alone. Data are presented as mean ± SD from three independent experiments, with triplicate samples of > 30 organisms per condition.

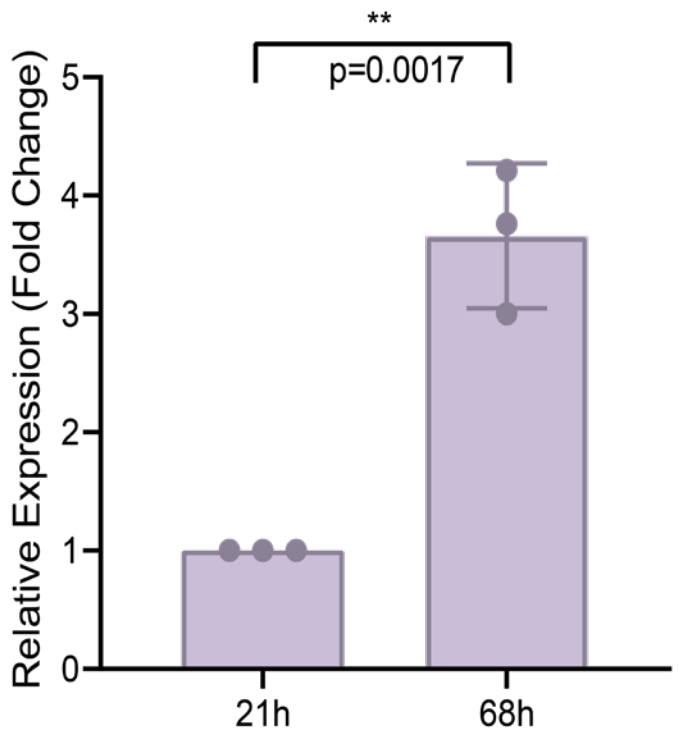

Figure S16. Transcriptional analysis of *pisP* in the Δ*plcR* mutant during Bt infection of *C. elegans*.

Relative *pisP* mRNA levels (fold change) in the *ΔplcR* mutant were measured by qRT-PCR at 21 and 68 h during infection of *C. elegans*. Expression levels were normalized to those at 21 h (set to 1). At 68 h, *pisP* transcript levels were approximately three-fold higher than those at 21 h, suggesting that *pisP* expression during infection may also be regulated through PlcR-independent mechanisms. All qRT-PCR transcript data were normalized to the internal reference gene *16S rRNA*. Data are presented as mean ± SD, with individual biological replicates shown as dots. n=3. Statistical significance was determined using an unpaired t-test. **P < 0.01.

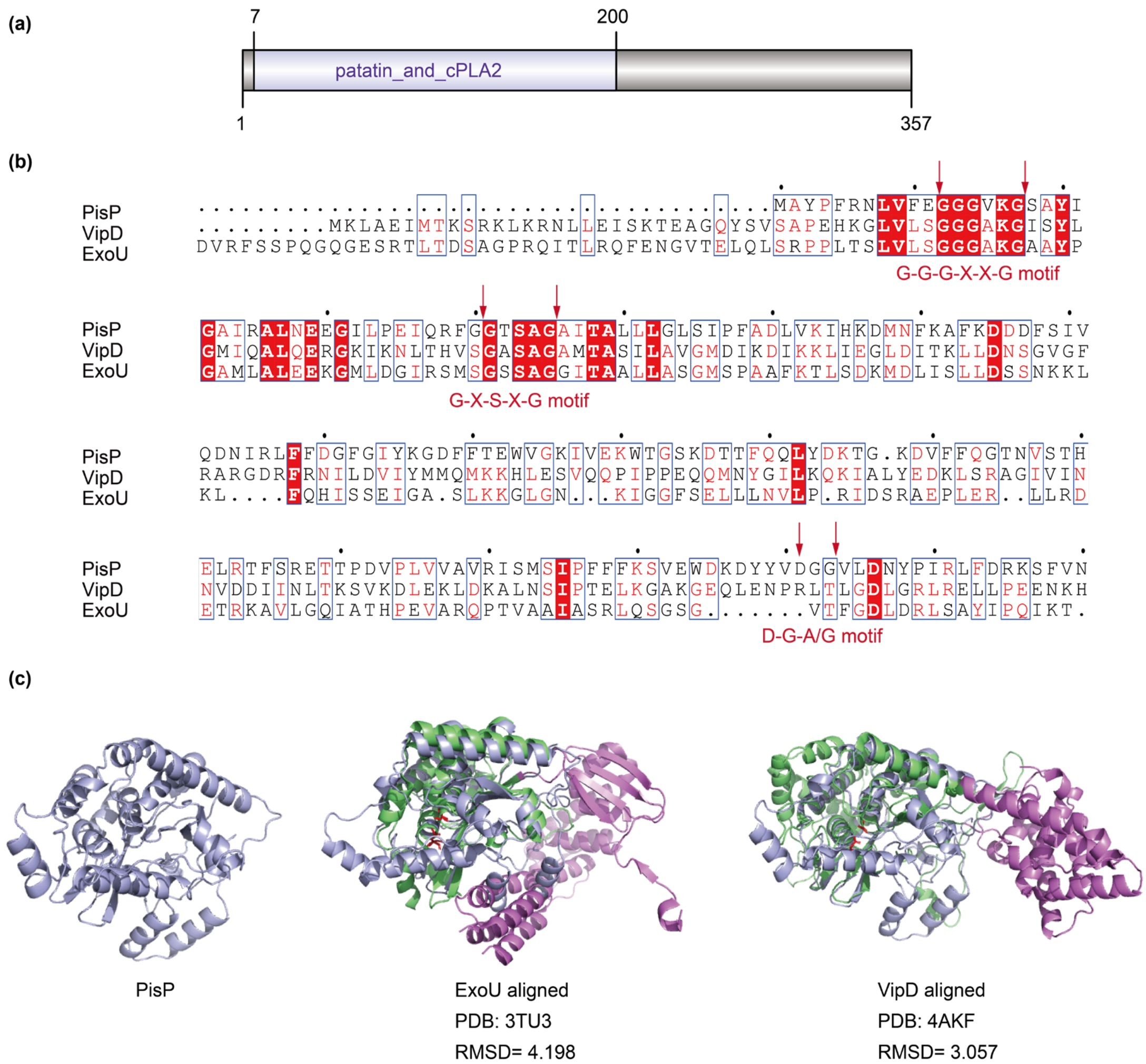

Figure S17. Sequence and structural divergence of the Bt Patatin-like protein (PisP).

(A) Conserved domain architecture of PisP predicted by CDD analysis, showing the patatin and cPLA2 phospholipase domain. **(B)** Multiple sequence alignment of PisP with characterized phospholipases, including VipD (PDB: 4AKF) and ExoU (PDB: 3TU3). Conserved catalytic motifs (G–G–G–X–X–G and G–X–S–X–G) and putative essential catalytic residues are indicated by arrows, while conserved amino acids are highlighted with a red background. **(C)** AlphaFold3-predicted three-dimensional structure of PisP and structural alignment with ExoU (middle; PDB: 3TU3, RMSD = 4.198 Å) and VipD (right; PDB: 4AKF, RMSD = 3.057 Å). PisP is shown in purple; regions exhibiting partial structural similarity, primarily in the N-terminal region, are shown in green, and structurally divergent regions, particularly the C-terminal region

lacking correspondence to ExoU and VipD, are shown in magenta. The left panel shows the predicted structure of PisP alone.

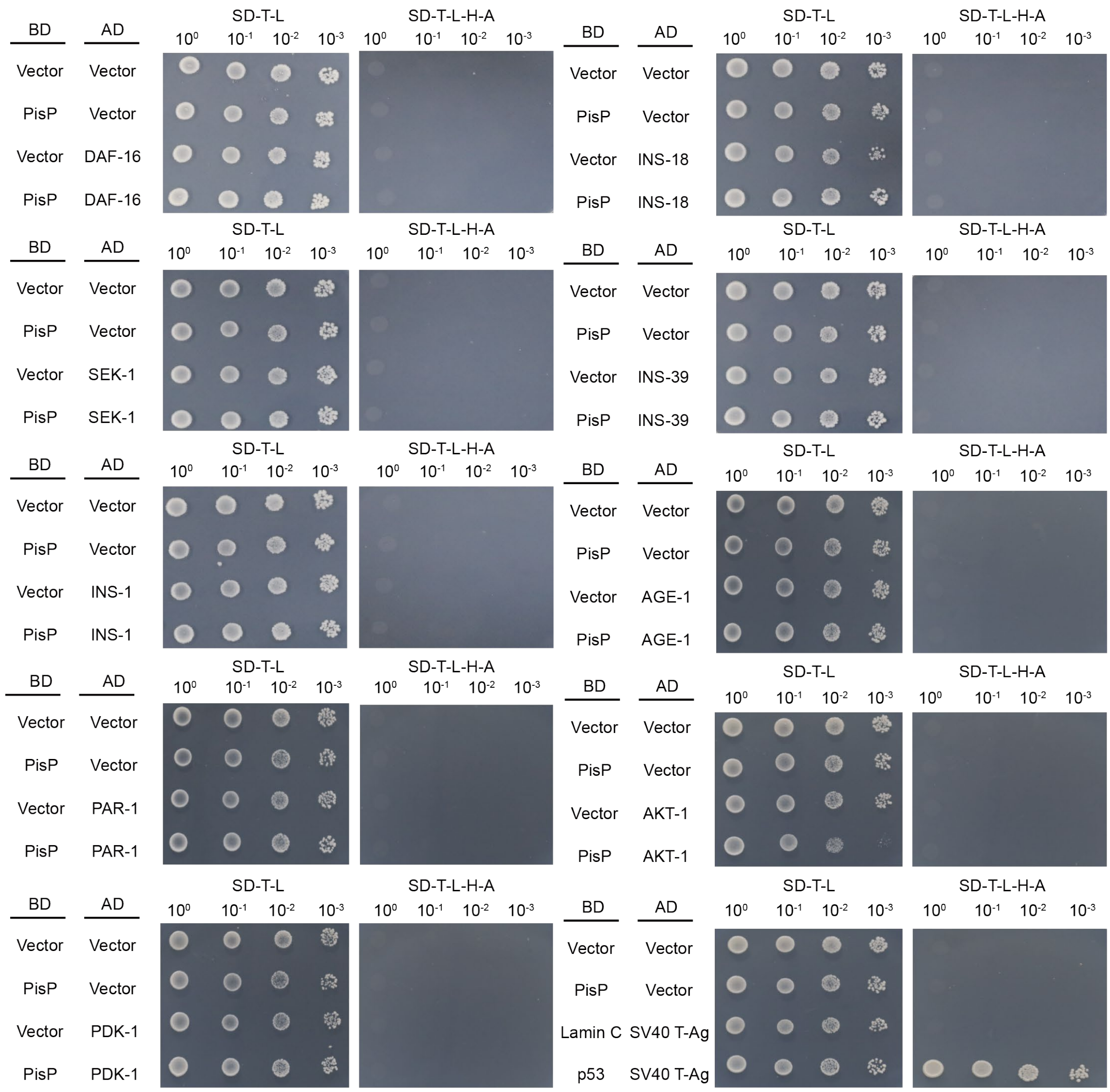

Figure S18. Yeast two-hybrid analysis of interactions between PisP and candidate components of the DAF-16 and insulin-like pathways.

The coding regions of *pisP* and the candidate target genes (*daf-16*, *sek-1*, *ins-1*, *par-1*, *pdk-1*, *ins-18*, *ins-39*, *age-1*, and *akt-1*) were amplified by PCR and cloned into pGBKT7 (BD) or pGADT7 (AD) vectors using the ClonExpress II One Step Cloning Kit. The bait plasmid pGBKT7-PisP and each prey plasmid pGADT7-target prey-plasmid were co-transformed into yeast *Saccharomyces cerevisiae* strain AH109. Transformants were selected on SD medium lacking tryptophan and leucine (SD/-Trp/-Leu) at 30 °C for 3 days and subsequently tested for interaction on SD medium lacking tryptophan, leucine, histidine, and adenine (SD/-Trp/-Leu/-His/-Ade). Only the positive

control (p53/SV40 T-Antigen) grew on SD/-Trp/-Leu/-His/-Ade plates, whereas yeast co-expressing PisP with any of the tested candidate proteins, as well as the negative controls (pGBKT7-PisP/pGADT7; pGBKT7/pGADT7-prey; Lamin C/SV40 T-Antigen), showed no grow. These results indicate that no direct interactions between PisP and the tested candidate proteins were detected under the conditions of the yeast two-hybrid assay.

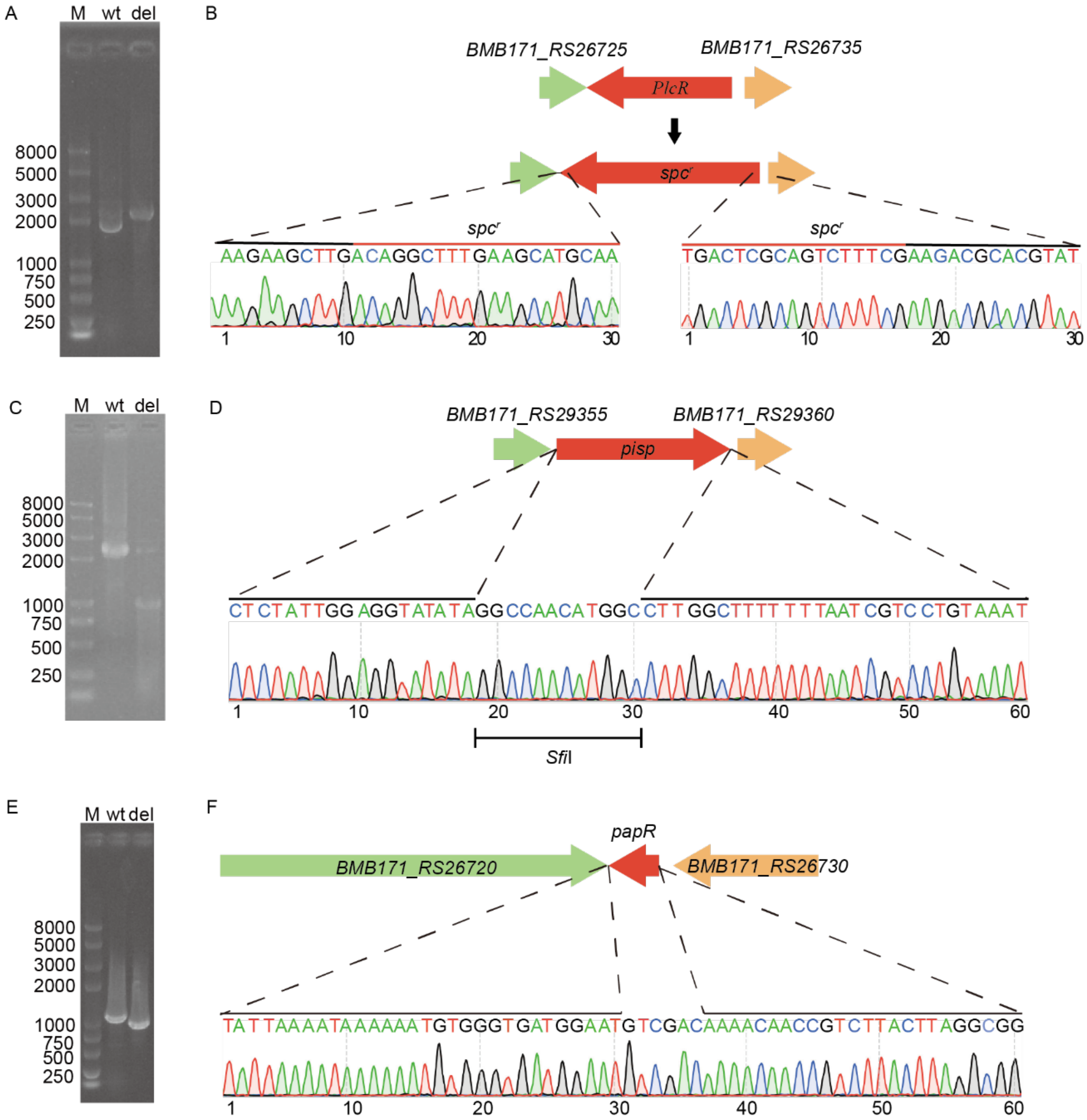

Figure S19. Validation of *plcR, pisP* and *papR* knockouts in BMB171.

The *plcR* gene was replaced by a spectinomycin resistance gene (*spc*) by homologous recombination, whereas the *pisP* and *papR* knockout mutants were constructed using the CRISPR-Cas9 system as described in the Experimental Section. **(A, C, E)** PCR verification of the *plcR*, *pisP*, and *papR* knockout mutants, respectively using different primer pairs. Genomic DNA from wild-type (wt) and corresponding deletion mutant (del) strains was used as a template. **(B, D, F)** Sanger sequencing chromatograms confirming the corresponding genetic modifications.

Table S1. WormCat enrichment analysis of differentially expressed genes (DEGs) uniquely responsive to the live BMB171/Cry5Ba strain.

	Wormbase.ID	Sequence.ID	Category.1	Category.2	Category.3	Automated. Description
1	WBGene00000002	F27C8.1	Transmembrane transport	Transmembrane transport: amino acid	Transmembrane transport: amino acid	aat-1 is an ortholog of human SLC7A5 (solute carrier family 7 member 5) and SLC7A8 (solute carrier family 7 member 8)\; contributes to L-amino acid transmembrane transporter activity\; is involved in amino acid transmembrane transport\; localizes to the amino acid transport complex\; is expressed in several tissues, including the alimentary system, the excretory system, the nervous system, the reproductive system, and the tail\; human orthologs of this gene are implicated in lysinuric protein intolerance.
2	WBGene00000024	AC3.3	Stress response	Stress response: ER	Stress response: ER	abu-1 is involved in endoplasmic reticulum unfolded protein response\; localizes to the integral component of endoplasmic reticulum membrane\; is expressed in the pharynx.
3	WBGene00000029	C03A7.7	Unassigned	Unassigned	Unassigned	abu-6 is involved in pharynx development\; is expressed in the pharyngeal muscle cell.

4	WBGene00000030	C03A7.8	Unassigned	Unassigned	Unassigned	abu-7 is involved in pharynx development\; is expressed in the grinder.
5	WBGene00000033	F35A5.3	Stress response	Stress response: ER	Stress response: ER	abu-10 is involved in endoplasmic reticulum unfolded protein response.
6	WBGene00000034	T01D1.6	Stress response	Stress response: ER	Stress response: ER	abu-11 is involved in endoplasmic reticulum unfolded protein response.
7	WBGene00000039	C42D8.5	Development	Development: somatic	Development: somatic	acn-1 is an ortholog of human ACE2\; is predicted to have metallopeptidase activity and peptidyl-dipeptidase activity\; is involved in ecdysis, collagen and cuticulin-based cuticle, nematode male tail tip morphogenesis, and plasma membrane fusion\; is expressed in the epithelial system, the excretory gland cell, the male, and the vulva\; human orthologs of this gene are implicated in Alzheimer's disease.
8	WBGene00000066	M03F4.2	Cytoskeleton	Cytoskeleton: actin function	Cytoskeleton: actin function: actin	act-4 is an ortholog of human ACTB, ACTRT1 (actin related protein T1), and ACTL10 (actin like 10) and members of the human ACT (Actins) gene family including ACTC1, ACTA2, and ACTA1\; is predicted to be a structural constituent of

						cytoskeleton\; localizes to the striated muscle thin filament\; is expressed in several tissues, including the body wall muscle quadrant, the muscular system, the pharynx, and the reproductive system\; human orthologs of this gene are implicated in several diseases, including artery disease (multiple), intrinsic cardiomyopathy (multiple), and myopathy (multiple).
9	WBGene00000082	C02B4.1	Proteolysis general	Proteolysis general: metallopeptidase	Proteolysis general: metallopeptidase	adt-1 is an ortholog of human ADAMTS14 (ADAM metallopeptidase with thrombospondin type 1 motif 14), ADAMTS2 (ADAM metallopeptidase with thrombospondin type 1 motif 2), and ADAMTS3 (ADAM metallopeptidase with thrombospondin type 1 motif 3)\; is predicted to have metal ion binding activity and metalloendopeptidase activity\; is expressed in several tissues, including the muscular system, the nervous system, and the vulva.
10	WBGene00000088	F32A7.6	Proteolysis general	Proteolysis general: serine	Proteolysis general: serine	aex-5 is an ortholog of human PCSK1 (proprotein convertase subtilisin\/kexin type 1)\; exhibits

						serine-type endopeptidase activity\; is involved in several processes, including defecation, lipid transport involved in lipid storage, and positive regulation of transport\; localizes to the extracellular space.
11	WBGene00000210	C53B7.4	Metabolism	Metabolism: mitochondria	Metabolism: mitochondria: complex V	asg-2 is an ortholog of human ATP5L2 and ATP5MG (ATP synthase membrane subunit g)\; is predicted to have proton transmembrane transporter activity\; localizes to the mitochondrion and non-motile cilium.
12	WBGene00000215	T18H9.2	Proteolysis general	Proteolysis general: aspartate	Proteolysis general: aspartate: unassigned	asp-2 is an ortholog of human PGA4, NAPSA, and PGA5\; is predicted to have aspartic-type endopeptidase activity.
13	WBGene00000216	H22K11.1	Proteolysis general	Proteolysis general: aspartate	Proteolysis general: aspartate: unassigned	asp-3 is an ortholog of human PGA4, NAPSA, and PGA5\; is predicted to have aspartic-type endopeptidase activity\; is involved in necrotic cell death\; localizes to the lysosome\; is expressed in the intestine and uterus.
14	WBGene0000022	W04D2.1	Cytoskeleton	Cytoskeleton:	Cytoskeleton:	atn-1 is an ortholog of human ACTN1 (actinin

	8			actin function	actin function: actin	alpha 1), ACTN4 (actinin alpha 4), and ACTN3 (actinin alpha 3 (gene\/pseudogene))\; is predicted to have actin binding activity\; localizes to the striated muscle dense body and striated muscle thin filament\; is expressed in several tissues, including the body wall, the muscular system, the pharynx, the reproductive system, and the tail\; human orthologs of this gene are implicated in dilated cardiomyopathy 1AA, focal segmental glomerulosclerosis 1, and platelet-type bleeding disorder 15.
15	WBGene00000256	Y73B6BL.34	Extracellular material	Extracellular material: collagen	Extracellular material: collagen	bli-6 is an ortholog of human MARCO (macrophage receptor with collagenous structure)\; is predicted to be a structural constituent of cuticle.
16	WBGene00000298	F32G8.6	Neuronal function	Neuronal function: synaptic function	Neuronal function: synaptic function: neurotransmitter metabolism	cat-4 is an ortholog of human GCH1\; exhibits GTP cyclohydrolase I activity\; is involved in dopamine biosynthetic process from tyrosine, melanin biosynthetic process, and tetrahydrobiopterin biosynthetic process\; is expressed in several tissues, including the alimentary system, the hyp7

17						syncytium, the muscular system, and the nervous system\; human orthologs of this gene are implicated in dystonia 5.
17	WBGene00000596	C15A11.5	Extracellular material	Extracellular material: collagen	Extracellular material: collagen	col-7 is an ortholog of human SFTPD (surfactant protein D)\; is predicted to be a structural constituent of cuticle\; is expressed in the seam cell.
18	WBGene00000599	B0222.8	Extracellular material	Extracellular material: collagen	Extracellular material: collagen	col-10 is an ortholog of human SFTPD (surfactant protein D)\; is predicted to be a structural constituent of cuticle.
19	WBGene00000601	F15H10.1	Extracellular material	Extracellular material: collagen	Extracellular material: collagen	col-12 is an ortholog of human MARCO (macrophage receptor with collagenous structure)\; is predicted to be a structural constituent of cuticle.
20	WBGene00000602	F15H10.2	Extracellular material	Extracellular material: collagen	Extracellular material: collagen	col-13 is an ortholog of human MARCO (macrophage receptor with collagenous structure)\; is predicted to be a structural constituent of cuticle.
21	WBGene00000603	C46A5.3	Extracellular material	Extracellular material: collagen	Extracellular material: collagen	col-14 is an ortholog of human MARCO (macrophage receptor with collagenous structure)\; is predicted to be a structural constituent of cuticle.
22	WBGene00000606	F11G11.10	Extracellular material	Extracellular material:	Extracellular material:	col-17 is enriched in the DA neuron, the VA neuron, the germline precursor cell, and the

				collagen	collagen	hypodermis based on tiling array and microarray studies\; is affected by several genes including daf-16, daf-2, and glp-1 based on microarray and RNA-seq studies\; is affected by twenty-one chemicals including hydrogen sulfide, Nitric Oxide, and methylmercury hydroxide based on microarray and RNA-seq studies.
23	WBGene00000615	F54C9.4	Extracellular material	Extracellular material: collagen	Extracellular material: collagen	col-38 is an ortholog of human SFTPD (surfactant protein D)\; is predicted to be a structural constituent of cuticle\; is expressed in the hyp7 syncytium and the seam cell.
24	WBGene00000626	K09H9.3	Extracellular material	Extracellular material: collagen	Extracellular material: collagen	col-49 is an ortholog of human SFTPD (surfactant protein D)\; is predicted to be a structural constituent of cuticle\; is expressed in the hyp7 syncytium and the seam cell.
25	WBGene00000634	F26B1.4	Extracellular material	Extracellular material: collagen	Extracellular material: collagen	col-58 is an ortholog of human COL16A1 (collagen type XVI alpha 1 chain) and COLQ\; is predicted to be a structural constituent of cuticle.
26	WBGene00000636	F22D6.10	Extracellular material	Extracellular material:	Extracellular material:	col-60 is an ortholog of human SFTPD (surfactant protein D)\; is predicted to be a structural

				collagen	collagen	constituent of cuticle.
27	WBGene00000637	C01H6.1	Extracellular material	Extracellular material: collagen	Extracellular material: collagen	col-61 is an ortholog of human SFTPD (surfactant protein D)\; is predicted to be a structural constituent of cuticle.
28	WBGene00000638	C15A11.6	Extracellular material	Extracellular material: collagen	Extracellular material: collagen	col-62 is an ortholog of human SFTPD (surfactant protein D)\; is predicted to be a structural constituent of cuticle.
29	WBGene00000639	ZK265.2	Extracellular material	Extracellular material: collagen	Extracellular material: collagen	col-63 is an ortholog of human SFTPD (surfactant protein D)\; is predicted to be a structural constituent of cuticle.
30	WBGene00000645	K01A2.7	Extracellular material	Extracellular material: collagen	Extracellular material: collagen	col-69 is an ortholog of human SFTPD (surfactant protein D)\; is predicted to be a structural constituent of cuticle.
31	WBGene00000646	H17B01.2	Extracellular material	Extracellular material: collagen	Extracellular material: collagen	H17B01.2 is affected by several genes including daf-2, glp-1, and let-7 based on microarray, tiling array, proteomic, and RNA-seq studies\; is affected by fifteen chemicals including Mercuric Chloride, Rotenone, and Tunicamycin based on microarray and RNA-seq studies.
32	WBGene0000064	Y49F6B.10	Extracellular	Extracellular	Extracellular	col-71 is an ortholog of human COLQ\; is predicted

	7		material	material: collagen	material: collagen	to be a structural constituent of cuticle\; is expressed in the hyp7 syncytium and the seam cell.
33	WBGene00000649	F11G11.12	Extracellular material	Extracellular material: collagen	Extracellular material: collagen	col-73 is an ortholog of human MARCO (macrophage receptor with collagenous structure)\; is predicted to be a structural constituent of cuticle.
34	WBGene00000653	M195.1	Extracellular material	Extracellular material: collagen	Extracellular material: collagen	col-77 is an ortholog of human MARCO (macrophage receptor with collagenous structure)\; is predicted to be a structural constituent of cuticle.
35	WBGene00000657	F38A3.1	Extracellular material	Extracellular material: collagen	Extracellular material: collagen	col-81 is an ortholog of human MARCO (macrophage receptor with collagenous structure)\; is predicted to be a structural constituent of cuticle.
36	WBGene00000663	W05G11.3	Extracellular material	Extracellular material: collagen	Extracellular material: collagen	col-88 is an ortholog of human SFTPD (surfactant protein D)\; is predicted to be a structural constituent of cuticle.
37	WBGene00000664	F17C8.2	Extracellular material	Extracellular material: collagen	Extracellular material: collagen	col-89 is an ortholog of human SFTPD (surfactant protein D)\; is predicted to be a structural constituent of cuticle\; is expressed in several tissues, including the head, the intestine, the nervous system, the reproductive system, and the tail.

38	WBGene00000665	C29E4.1	Extracellular material	Extracellular material: collagen	Extracellular material: collagen	col-90 is an ortholog of human SFTPD (surfactant protein D)\; is predicted to be a structural constituent of cuticle.
39	WBGene00000666	F09G8.6	Extracellular material	Extracellular material: collagen	Extracellular material: collagen	col-91 is an ortholog of human SFTPD (surfactant protein D)\; is predicted to be a structural constituent of cuticle.
40	WBGene00000667	W05B2.6	Extracellular material	Extracellular material: collagen	Extracellular material: collagen	col-92 is an ortholog of human SFTPD (surfactant protein D)\; is predicted to be a structural constituent of cuticle\; is expressed in the hypodermis.
41	WBGene00000668	W05B2.5	Extracellular material	Extracellular material: collagen	Extracellular material: collagen	col-93 is an ortholog of human SFTPD (surfactant protein D)\; is predicted to be a structural constituent of cuticle.
42	WBGene00000669	W05B2.1	Extracellular material	Extracellular material: collagen	Extracellular material: collagen	col-94 is an ortholog of human SFTPD (surfactant protein D)\; is predicted to be a structural constituent of cuticle.
43	WBGene00000672	ZK1010.7	Extracellular material	Extracellular material: collagen	Extracellular material: collagen	col-97 is predicted to be a structural constituent of cuticle.
44	WBGene0000067	F58F6.1	Extracellular	Extracellular	Extracellular	col-104 is an ortholog of human OTOL1,

	8		material	material: collagen	material: collagen	C1QTNF7 (C1q and TNF related 7), and C1QTNF9 (C1q and TNF related 9)\; is predicted to be a structural constituent of cuticle.
45	WBGene00000681	C34H4.4	Extracellular material	Extracellular material: collagen	Extracellular material: collagen	col-107 is an ortholog of human SFTPD (surfactant protein D)\; is predicted to be a structural constituent of cuticle.
46	WBGene00000683	Y38C1BA.3	Extracellular material	Extracellular material: collagen	Extracellular material: collagen	col-109 is predicted to be a structural constituent of cuticle\; is involved in endoplasmic reticulum unfolded protein response.
47	WBGene00000684	F19C7.7	Extracellular material	Extracellular material: collagen	Extracellular material: collagen	col-110 is predicted to be a structural constituent of cuticle.
48	WBGene00000685	F29B9.9	Extracellular material	Extracellular material: collagen	Extracellular material: collagen	col-111 is an ortholog of human OTOL1, C1QTNF7 (C1q and TNF related 7), and C1QTNF9 (C1q and TNF related 9)\; is predicted to be a structural constituent of cuticle.
49	WBGene00000687	C34D4.15	Extracellular material	Extracellular material: collagen	Extracellular material: collagen	col-113 is an ortholog of human OTOL1, C1QTNF7 (C1q and TNF related 7), and C1QTNF9 (C1q and TNF related 9)\; is predicted to be a structural constituent of cuticle.

50	WBGene00000689	Y42H9B.1	Extracellular material	Extracellular material: collagen	Extracellular material: collagen	col-115 is an ortholog of human COL16A1 (collagen type XVI alpha 1 chain)\; human COL16A1 exhibits integrin binding activity.
51	WBGene00000694	Y11D7A.11	Extracellular material	Extracellular material: collagen	Extracellular material: collagen	col-120 is an ortholog of human MARCO (macrophage receptor with collagenous structure)\; is predicted to be a structural constituent of cuticle.
52	WBGene00000699	C29F4.1	Extracellular material	Extracellular material: collagen	Extracellular material: collagen	col-125 is an ortholog of human MARCO (macrophage receptor with collagenous structure)\; is predicted to be a structural constituent of cuticle.
53	WBGene00000702	F12F6.9	Extracellular material	Extracellular material: collagen	Extracellular material: collagen	col-128 is an ortholog of human COL11A1 (collagen type XI alpha 1 chain), COL5A1 (collagen type V alpha 1 chain), and COL5A3 (collagen type V alpha 3 chain)\; human COL5A1 exhibits heparin binding activity, platelet-derived growth factor binding activity, and proteoglycan binding activity.
54	WBGene00000703	M18.1	Extracellular material	Extracellular material: collagen	Extracellular material: collagen	col-129 is an ortholog of human SFTPD (surfactant protein D)\; is predicted to be a structural constituent of cuticle\; is expressed in the hypodermis and the intestine.

55	WBGene00000704	F08G5.4	Extracellular material	Extracellular material: collagen	Extracellular material: collagen	col-130 is an ortholog of human SFTPD (surfactant protein D)\; is predicted to be a structural constituent of cuticle\; is expressed in the intestine and the vulva.
56	WBGene00000707	F52B11.4	Extracellular material	Extracellular material: collagen	Extracellular material: collagen	col-133 is an ortholog of human MARCO (macrophage receptor with collagenous structure)\; is predicted to be a structural constituent of cuticle.
57	WBGene00000710	Y51H4A.9	Extracellular material	Extracellular material: collagen	Extracellular material: collagen	col-137 is an ortholog of human MARCO (macrophage receptor with collagenous structure)\; is predicted to be a structural constituent of cuticle.
58	WBGene00000711	C52D10.13	Extracellular material	Extracellular material: collagen	Extracellular material: collagen	col-138 is an ortholog of human MARCO (macrophage receptor with collagenous structure)\; is predicted to be a structural constituent of cuticle.
59	WBGene00000714	T15B7.5	Extracellular material	Extracellular material: collagen	Extracellular material: collagen	col-141 is an ortholog of human SFTPD (surfactant protein D)\; is predicted to be a structural constituent of cuticle.
60	WBGene00000717	B0222.6	Extracellular material	Extracellular material: collagen	Extracellular material: collagen	col-144 is an ortholog of human SFTPD (surfactant protein D)\; is predicted to be a structural constituent of cuticle\; is expressed in the hypodermis.

61	WBGene00000718	B0222.7	Extracellular material	Extracellular material: collagen	Extracellular material: collagen	col-145 is an ortholog of human SFTPD (surfactant protein D)\; is predicted to be a structural constituent of cuticle.
62	WBGene00000719	T06E4.6	Extracellular material	Extracellular material: collagen	Extracellular material: collagen	col-146 is an ortholog of human SFTPD (surfactant protein D)\; is predicted to be a structural constituent of cuticle.
63	WBGene00000720	T06E4.4	Extracellular material	Extracellular material: collagen	Extracellular material: collagen	col-147 is an ortholog of human SFTPD (surfactant protein D)\; is predicted to be a structural constituent of cuticle.
64	WBGene00000723	B0024.2	Extracellular material	Extracellular material: collagen	Extracellular material: collagen	col-150 is an ortholog of human SFTPD (surfactant protein D)\; is predicted to be a structural constituent of cuticle.
65	WBGene00000725	F32G8.5	Extracellular material	Extracellular material: collagen	Extracellular material: collagen	col-152 is an ortholog of human SFTPD (surfactant protein D)\; is predicted to be a structural constituent of cuticle.
66	WBGene00000727	F55C10.2	Extracellular material	Extracellular material: collagen	Extracellular material: collagen	col-154 is an ortholog of human MARCO (macrophage receptor with collagenous structure)\; is predicted to be a structural constituent of cuticle.
67	WBGene00000728	F55C10.3	Extracellular material	Extracellular material:	Extracellular material:	col-155 is an ortholog of human MARCO (macrophage receptor with collagenous structure)\;

				collagen	collagen	is predicted to be a structural constituent of cuticle.
68	WBGene00000729	F57B7.3	Extracellular material	Extracellular material: collagen	Extracellular material: collagen	col-156 is an ortholog of human SFTPD (surfactant protein D)\; is predicted to be a structural constituent of cuticle.
69	WBGene00000730	T11F9.9	Extracellular material	Extracellular material: collagen	Extracellular material: collagen	col-157 is an ortholog of human MARCO (macrophage receptor with collagenous structure)\; is predicted to be a structural constituent of cuticle.
70	WBGene00000731	D2023.7	Extracellular material	Extracellular material: collagen	Extracellular material: collagen	col-158 is an ortholog of human COLQ\; is predicted to be a structural constituent of cuticle.
71	WBGene00000732	F57B1.3	Extracellular material	Extracellular material: collagen	Extracellular material: collagen	col-159 is an ortholog of human MARCO (macrophage receptor with collagenous structure)\; is predicted to be a structural constituent of cuticle.
72	WBGene00000734	C50B6.4	Extracellular material	Extracellular material: collagen	Extracellular material: collagen	col-161 is an ortholog of human SFTPD (surfactant protein D)\; is predicted to be a structural constituent of cuticle.
73	WBGene00000735	Y2H9A.3	Extracellular material	Extracellular material: collagen	Extracellular material: collagen	col-162 is an ortholog of human SFTPD (surfactant protein D)\; is predicted to be a structural constituent of cuticle.
74	WBGene0000074	T10E10.2	Extracellular	Extracellular	Extracellular	col-167 is an ortholog of human MARCO

	0		material	material: collagen	material: collagen	(macrophage receptor with collagenous structure)\; is predicted to be a structural constituent of cuticle.
75	WBGene00000741	T10E10.1	Extracellular material	Extracellular material: collagen	Extracellular material: collagen	col-168 is an ortholog of human MARCO (macrophage receptor with collagenous structure)\; is predicted to be a structural constituent of cuticle.
76	WBGene00000742	T10E10.5	Extracellular material	Extracellular material: collagen	Extracellular material: collagen	col-169 is an ortholog of human MARCO (macrophage receptor with collagenous structure)\; is predicted to be a structural constituent of cuticle.
77	WBGene00000743	T10E10.6	Extracellular material	Extracellular material: collagen	Extracellular material: collagen	col-170 is an ortholog of human MARCO (macrophage receptor with collagenous structure)\; is predicted to be a structural constituent of cuticle.
78	WBGene00000746	F41C6.5	Extracellular material	Extracellular material: collagen	Extracellular material: collagen	col-173 is an ortholog of human MARCO (macrophage receptor with collagenous structure)\; is predicted to be a structural constituent of cuticle.
79	WBGene00000748	C35B8.1	Extracellular material	Extracellular material: collagen	Extracellular material: collagen	col-175 is an ortholog of human SFTPD (surfactant protein D)\; is predicted to be a structural constituent of cuticle.
80	WBGene00000749	ZC373.7	Extracellular material	Extracellular material: collagen	Extracellular material: collagen	col-176 is an ortholog of human SFTPD (surfactant protein D)\; is predicted to be a structural constituent of cuticle.

81	WBGene00000753	C44C10.1	Extracellular material	Extracellular material: collagen	Extracellular material: collagen	col-180 is an ortholog of human MARCO (macrophage receptor with collagenous structure)\; is predicted to be a structural constituent of cuticle.
82	WBGene00000755	R09A8.4	Pseudogene	Pseudogene	Pseudogene	col-182 is an ortholog of human OTOL1, C1QTNF7 (C1q and TNF related 7), and C1QTNF9 (C1q and TNF related 9)\; is predicted to be a structural constituent of cuticle.
83	WBGene00000785	W07B8.5	Proteolysis general	Proteolysis general: cysteine	Proteolysis general: cysteine: unassigned	cpr-5 is an ortholog of human CTSB (cathepsin B)\; is predicted to have cysteine-type peptidase activity.
84	WBGene00000822	F40E10.3	Signaling	Signaling: calcium	Signaling: calcium: unassigned	csq-1 is an ortholog of human CASQ1 (calsequestrin 1) and CASQ2 (calsequestrin 2)\; exhibits calcium ion binding activity\; is involved in calcium ion homeostasis\; localizes to several cellular components, including the apical plasma membrane, cytoplasm, and nucleus\; is expressed in isthmus, the body wall musculature, the terminal bulb, and the vulval muscle\; human orthologs of this gene are implicated in catecholaminergic polymorphic ventricular tachycardia 1 and

						catecholaminergic polymorphic ventricular tachycardia 2.
85	WBGene00000851	C47G2.1	Extracellular material	Extracellular material: cuticlin	Extracellular material: cuticlin	cut-1 is predicted to be a structural constituent of cuticle\; localizes to the collagen and cuticulin-based cuticle extracellular matrix\; is expressed in the seam cell.
86	WBGene00000878	ZK520.5	Chaperone	Chaperone: cyclophilin	Chaperone: cyclophilin	cyn-2 is an ortholog of human PPIA (peptidylprolyl isomerase A), PPIAL4D, and PPIAL4G\; is predicted to have peptidyl-prolyl cis-trans isomerase activity.
87	WBGene00000882	F42G9.2	Chaperone	Chaperone: cyclophilin	Chaperone: cyclophilin	cyn-6 is an ortholog of human PPIB (peptidylprolyl isomerase B)\; is predicted to have peptidyl-prolyl cis-trans isomerase activity\; is expressed in the intestine\; human orthologs of this gene are implicated in osteogenesis imperfecta type 9.
88	WBGene00000930	ZC373.6	Unassigned	Unassigned	Unassigned	dao-4 is expressed in the hyp7 syncytium and the seam cell.
89	WBGene00000966	F55A12.4	Metabolism	Metabolism: lipid	Metabolism: lipid: sterol	dhs-2 is an ortholog of human HSD17B6 (hydroxysteroid 17-beta dehydrogenase 6), RDH5 (retinol dehydrogenase 5), and DHRS9

						(dehydrogenase\/reductase 9)\; human orthologs of this gene are implicated in fundus albipunctatus\; human DHRS9 exhibits alcohol dehydrogenase (NAD) activity, retinol dehydrogenase activity, and testosterone dehydrogenase (NAD+) activity.
90	WBGene00000973	Y32H12A.3	Metabolism	Metabolism: short chain dehydrogenase	Metabolism: short chain dehydrogenase	dhs-9 is an ortholog of human DHRS1 (dehydrogenase\/reductase 1)\; is expressed in several tissues, including the alimentary system, the head, and the nervous system\; is predicted to encode a protein with the following domains: Short-chain dehydrogenase\/reductase SDR and NAD(P)-binding domain superfamily.
91	WBGene00000981	C45B11.3	Metabolism	Metabolism: short chain dehydrogenase	Metabolism: short chain dehydrogenase	dhs-18 is an ortholog of human HSDL2 (hydroxysteroid dehydrogenase like 2)\; is predicted to encode a protein with the following domains: Short-chain dehydrogenase\/reductase SDR and NAD(P)-binding domain superfamily.
92	WBGene00000983	F35B12.2	Metabolism	Metabolism: short chain dehydrogenase	Metabolism: short chain dehydrogenase	dhs-20 is an ortholog of human HSD17B6 (hydroxysteroid 17-beta dehydrogenase 6), DHRS9 (dehydrogenase\/reductase 9), and RDH5 (retinol

						dehydrogenase 5)\; human orthologs of this gene are implicated in fundus albipunctatus\; human DHRS9 exhibits alcohol dehydrogenase (NAD) activity, retinol dehydrogenase activity, and testosterone dehydrogenase (NAD+) activity.
93	WBGene00000984	R11D1.11	Metabolism	Metabolism: carbohydrate	Metabolism: carbohydrate	dhs-21 is an ortholog of human DCXR (dicarbonyl and L-xylulose reductase)\; exhibits L-xylulose reductase (NADP+) activity and carbonyl reductase (NADPH) activity\; is involved in xylulose catabolic process\; localizes to the plasma membrane\; is expressed in several tissues, including the gonad, the hypodermis, and the intestine.
94	WBGene00000991	M03A8.1	Metabolism	Metabolism: lipid	Metabolism: lipid: sterol	dhs-28 is an ortholog of human SCP2D1\; is involved in several processes, including acyl-CoA metabolic process, ascaroside biosynthetic process, and dauer entry\; localizes to the peroxisome\; is expressed in the hypodermis\; is used to study obesity.
95	WBGene0000100	C18A11.7	Muscle	Muscle function	Muscle function	dim-1 is an ortholog of human TTN, MYBPC2,

	0		function			and MYBPC3\; is expressed in the body wall musculature\; human TTN exhibits several functions, including cellular protein modification process, cytoskeletal protein binding activity, and enzyme binding activity.
96	WBGene00001059	F44B9.1	Proteolysis general	Proteolysis general: serine	Proteolysis general: serine	dpf-6 is predicted to have aminopeptidase activity and serine-type peptidase activity\; is expressed in the intestine and the pharynx.
97	WBGene00001067	F27C1.8	Extracellular material	Extracellular material: collagen	Extracellular material: collagen	dpy-5 is an ortholog of human SFTPD (surfactant protein D)\; is a structural constituent of collagen and cuticulin-based cuticle\; is involved in cuticle development involved in collagen and cuticulin-based cuticle molting cycle, post-embryonic body morphogenesis, and regulation of growth\; localizes to the collagen and cuticulin-based cuticle extracellular matrix\; is expressed in the hypodermis.
98	WBGene00001070	C31H2.2	Extracellular material	Extracellular material: collagen	Extracellular material: collagen	dpy-8 is predicted to be a structural constituent of cuticle\; is involved in cuticle development involved in collagen and cuticulin-based cuticle

						molting cycle.
99	WBGene00001071	T21D12.2	Extracellular material	Extracellular material: collagen	Extracellular material: collagen	dpy-9 is predicted to be a structural constituent of cuticle.
100	WBGene00001074	F30B5.1	Extracellular material	Extracellular material: collagen	Extracellular material: collagen	dpy-13 is an ortholog of human OTOL1, C1QTNF7 (C1q and TNF related 7), and C1QTNF9 (C1q and TNF related 9)\; is predicted to be a structural constituent of cuticle\; is involved in cuticle development involved in collagen and cuticulin-based cuticle molting cycle\; localizes to the collagen and cuticulin-based cuticle extracellular matrix.
101	WBGene00001113	F25H8.5	Unassigned	Unassigned	Unassigned	dur-1 is enriched in the BAG, the body wall musculature, the germ line, and the muscle cell based on proteomic, tiling array, RNA-seq, and microarray studies\; is affected by several genes including daf-2, glp-1, and let-7 based on microarray, RNA-seq, and proteomic studies\; is affected by fourteen chemicals including methylmercury hydroxide, 1-methylnicotinamide,

						and Rotenone based on RNA-seq and microarray studies.
102	WBGene00001132	T11B7.4	Cytoskeleton	Cytoskeleton: actin function	Cytoskeleton: actin function: binding	alp-1 is an ortholog of human LDB3 (LIM domain binding 3) and PDLIM5 (PDZ and LIM domain 5)\; is predicted to have metal ion binding activity\; is involved in determination of adult lifespan and pharyngeal pumping\; localizes to several cellular components, including the apical part of cell, basal part of cell, and intracellular organelle\; is expressed in several tissues, including the epithelial system, the muscular system, and the nervous system\; is used to study cardiomyopathy and myopathy\; human orthologs of this gene are implicated in myofibrillar myopathy 4.
103	WBGene00001157	F01G10.2	Metabolism	Metabolism: lipid	Metabolism: lipid: beta oxidation	ech-8 is an ortholog of human EHHADH\; is predicted to have 3-hydroxyacyl-CoA dehydrogenase activity\; human orthologs of this gene are implicated in Fanconi syndrome.
104	WBGene00001171	F16B3.1	Transmembrane transport	Transmembrane transport:	Transmembrane transport:	egl-2 is an ortholog of human KCNH1 (potassium voltage-gated channel subfamily H member 1) and

				potassium channel	potassium channel	KCNH5 (potassium voltage-gated channel subfamily H member 5)\; exhibits protein kinase binding activity\; is involved in chemosensory behavior, muscle contraction, and oviposition\; localizes to the microvillus membrane\; is expressed in several tissues, including the head, the intestinal cell, the nervous system, and the vulval muscle.
105	WBGene00001242	C40H1.4	Metabolism	Metabolism: lipid	Metabolism: lipid: fatty acid	elo-4 is an ortholog of human ELOVL3 and ELOVL6\; is predicted to have transferase activity, transferring acyl groups other than amino-acyl groups.
106	WBGene00001387	F15B9.1	Metabolism	Metabolism: lipid	Metabolism: lipid: binding	far-3 is predicted to have lipid binding activity\; is expressed in the hypodermis, the intestine, the male, and the vulva.
107	WBGene00001428	C05C8.3	Chaperone	Chaperone: cyclophilin	Chaperone: cyclophilin	fkb-3 is an ortholog of human FKBP10 (FKBP prolyl isomerase 10) and FKBP9 (FKBP prolyl isomerase 9)\; is predicted to have peptidyl-prolyl cis-trans isomerase activity\; is expressed in the hypodermis.

108	WBGene00001429	ZC455.10	Chaperone	Chaperone: cyclophilin	Chaperone: cyclophilin	fkb-4 is an ortholog of human FKBP10 (FKBP prolyl isomerase 10) and FKBP9 (FKBP prolyl isomerase 9)\; is predicted to have peptidyl-prolyl cis-trans isomerase activity.
109	WBGene00001430	C50F2.6	Chaperone	Chaperone: cyclophilin	Chaperone: cyclophilin	fkb-5 is an ortholog of human FKBP10 (FKBP prolyl isomerase 10) and FKBP9 (FKBP prolyl isomerase 9)\; is predicted to have peptidyl-prolyl cis-trans isomerase activity\; is expressed in the hypodermis.
110	WBGene00001478	Y39A1A.19	Metabolism	Metabolism: FMO	Metabolism: FMO	fmo-3 is an ortholog of human FMO5 (flavin containing monooxygenase 5)\; is predicted to have N,N-dimethylaniline monooxygenase activity, NADP binding activity, and flavin adenine dinucleotide binding activity\; is expressed in the hypodermis.
111	WBGene00001479	F53F4.5	Metabolism	Metabolism: FMO	Metabolism: FMO	fmo-4 is an ortholog of human FMO5 (flavin containing monooxygenase 5)\; is predicted to have N,N-dimethylaniline monooxygenase activity, NADP binding activity, and flavin adenine dinucleotide binding activity\; is expressed in

						several tissues, including the epithelial system, the intestine, the nervous system, and the reproductive system.
112	WBGene00001578	R12A1.4	Proteolysis general	Proteolysis general: unassigned	Proteolysis general: unassigned	ges-1 is an ortholog of human ACHE (acetylcholinesterase (Cartwright blood group)) and CES2 (carboxylesterase 2)\; exhibits carboxylic ester hydrolase activity\; localizes to the peroxisome\; is expressed in several tissues, including the Eala, the Ealaa, the Ealp, the Eara, and the alimentary system.
113	WBGene00001690	R08B4.1	Signaling	Signaling: hedgehog-like	Signaling: hedgehog-like	grd-1 is predicted to have peptidase activity\; is expressed in the rect_D, the rect_VL, and the rect_VR.
114	WBGene00001691	F46B3.5	Signaling	Signaling: hedgehog-like	Signaling: hedgehog-like	grd-2 is predicted to have cholesterol binding activity, endopeptidase activity, and signaling receptor binding activity\; is expressed in the excretory duct and the excretory pore.
115	WBGene00001692	W05E7.1	Signaling	Signaling: hedgehog-like	Signaling: hedgehog-like	grd-3 is predicted to have signaling receptor binding activity\; is involved in defense response to Gram-negative bacterium\; is expressed in the

						hypodermis.
116	WBGene00001694	F41E6.2	Signaling	Signaling: hedgehog-like	Signaling: hedgehog-like	grd-5 is predicted to have signaling receptor binding activity\; is involved in embryo development\; is expressed in several tissues, including pharyngeal neurons, the epithelial system, and the reproductive system.
117	WBGene00001699	F09D12.1	Signaling	Signaling: hedgehog-like	Signaling: hedgehog-like	grd-10 is expressed in the seam cell\; is predicted to encode a protein with the following domain: Ground-like domain.
118	WBGene00001703	T01B10.2	Signaling	Signaling: hedgehog-like	Signaling: hedgehog-like	grd-14 is expressed in several tissues, including the alimentary system, the epithelial system, and the muscular system.
119	WBGene00001706	Y102A5C.34	Pseudogene	Pseudogene	Pseudogene	grd-17 is affected by several genes including clk-1, npr-1, and prg-1 based on RNA-seq and microarray studies\; is affected by Rotenone and D-glucose based on RNA-seq studies.
120	WBGene00001714	Y47D7A.5	Signaling	Signaling: hedgehog-like	Signaling: hedgehog-like	grl-5 is expressed in the hypodermis\; is predicted to encode a protein with the following domain: Ground-like domain.
121	WBGene0000171	T02E9.2	Signaling	Signaling:	Signaling:	grl-7 is expressed in the hypodermis and the

	6			hedgehog-like	hedgehog-like	intestine\; is predicted to encode a protein with the following domain: Ground-like domain.
122	WBGene00001724	Y75B8A.20	Signaling	Signaling: hedgehog-like	Signaling: hedgehog-like	grl-15 is expressed in the hypodermis, the midbody, and the reproductive system\; is predicted to encode a protein with the following domain: Ground-like domain.
123	WBGene00001725	Y65B4BR.6	Signaling	Signaling: hedgehog-like	Signaling: hedgehog-like	grl-16 is enriched in the hypodermis, the intestine, the nervous system, and the reproductive system based on proteomic, tiling array, RNA-seq, and microarray studies\; is affected by several genes including daf-16, daf-2, and glp-1 based on microarray, proteomic, and RNA-seq studies\; is affected by fifteen chemicals including hydrogen sulfide, 1-methylnicotinamide, and nicotinic acid based on microarray and RNA-seq studies\; is predicted to encode a protein with the following domain: Ground-like domain.
124	WBGene00001728	R02D3.6	Signaling	Signaling: hedgehog-like	Signaling: hedgehog-like	grl-19 is expressed in the excretory duct, the excretory pore, and the intestine\; is predicted to encode a protein with the following domain:

						Ground-like domain.
125	WBGene00001730	ZC168.5	Signaling	Signaling: hedgehog-like	Signaling: hedgehog-like	grl-21 is expressed in the arcade cell and the hypodermis\; is predicted to encode a protein with the following domain: Ground-like domain.
126	WBGene00001731	W03A5.3	Signaling	Signaling: hedgehog-like	Signaling: hedgehog-like	grl-22 is enriched in the hypodermis based on tiling array studies\; is affected by several genes including daf-2, dpy-10, and clk-1 based on microarray, tiling array, and RNA-seq studies\; is affected by eight chemicals including methylmercuric chloride, Rotenone, and Tunicamycin based on microarray and RNA-seq studies\; is predicted to encode a protein with the following domain: Ground-like domain.
127	WBGene00001753	R03D7.6	Stress response	Stress response: detoxification	Stress response: detoxification: GST	gst-5 is an ortholog of human GSTA4 (glutathione S-transferase alpha 4), GSTA5 (glutathione S-transferase alpha 5), and HPGDS (hematopoietic prostaglandin D synthase)\; is predicted to have glutathione transferase activity\; is involved in innate immune response\; is expressed in the intestine and the nerve ring.

128	WBGene00001754	F11G11.3	Stress response	Stress response: detoxification	Stress response: detoxification: GST	gst-6 is an ortholog of human GSTA4 (glutathione S-transferase alpha 4), GSTA5 (glutathione S-transferase alpha 5), and HPGDS (hematopoietic prostaglandin D synthase)\; is predicted to have glutathione transferase activity\; is involved in innate immune response.
129	WBGene00001758	Y45G12C.2	Stress response	Stress response: detoxification	Stress response: detoxification: GST	gst-10 is an ortholog of human GSTP1 (glutathione S-transferase pi 1)\; exhibits glutathione transferase activity\; is involved in several processes, including determination of adult lifespan, glutathione metabolic process, and heat acclimation\; is expressed in several tissues, including the anal depressor muscle, the head, the hypodermis, the intestine, and the nervous system\; is used to study obesity.
130	WBGene00001760	F37B1.2	Stress response	Stress response: detoxification	Stress response: detoxification: GST	gst-12 is an ortholog of human GSTA4 (glutathione S-transferase alpha 4), GSTA5 (glutathione S-transferase alpha 5), and HPGDS (hematopoietic prostaglandin D synthase)\; is predicted to have transferase activity.

131	WBGene00001774	Y53F4B.29	Stress response	Stress response: detoxification	Stress response: detoxification: GST	gst-26 is an ortholog of human GSTA4 (glutathione S-transferase alpha 4), GSTA5 (glutathione S-transferase alpha 5), and HPGDS (hematopoietic prostaglandin D synthase)\; is predicted to have transferase activity.
132	WBGene00001775	Y53F4B.30	Stress response	Stress response: detoxification	Stress response: detoxification: GST	gst-27 is an ortholog of human GSTA4 (glutathione S-transferase alpha 4), GSTA5 (glutathione S-transferase alpha 5), and HPGDS (hematopoietic prostaglandin D synthase)\; is predicted to have transferase activity.
133	WBGene00001843	W06D4.1	Metabolism	Metabolism: amino acid	Metabolism: amino acid: breakdown	hgo-1 is an ortholog of human HGD\; is predicted to have homogentisate 1,2-dioxygenase activity and metal ion binding activity\; localizes to the striated muscle dense body\; is expressed in the body wall musculature, the hypodermis, the intestine, the pharynx, and the vulval muscle\; human orthologs of this gene are implicated in alkaptonuria.
134	WBGene00001944	E03A3.4	Transcription: chromatin	Transcription: chromatin structure	Transcription: chromatin structure: histone	his-70 is an ortholog of human CENPA (centromere protein A), HIST2H3A (histone cluster 2 H3 a), and HIST2H3PS2 (histone cluster 2 H3 pseudogene 2)\;

						is predicted to have DNA binding activity and protein heterodimerization activity.
135	WBGene00002012	F38E11.1	Stress response	Stress response: heat	Stress response: heat	hsp-12.3 is an ortholog of human CRYAA (crystallin alpha A), CRYAB (crystallin alpha B), and HSPB2 (heat shock protein family B (small) member 2)\; exhibits heat shock protein binding activity and unfolded protein binding activity.
136	WBGene00002050	F38B2.1	Cytoskeleton	Cytoskeleton: intermediate filament protein	Cytoskeleton: intermediate filament protein	ifa-1 is an ortholog of human LMNB2 (lamin B2)\; is predicted to have structural molecule activity\; localizes to the intermediate filament\; is expressed in several tissues, including the alimentary system, the excretory cell, the nervous system, and the reproductive system\; human orthologs of this gene are implicated in several diseases, including Charcot-Marie-Tooth disease (multiple), eye disease (multiple), and hypotrichosis (multiple).
137	WBGene00002199	M176.6	Signaling	Signaling: Y kinase	Signaling: Y kinase	kin-15 is an ortholog of human FGFR4 (fibroblast growth factor receptor 4)\; is predicted to have ATP binding activity and transmembrane receptor protein tyrosine kinase activity\; is expressed in the

						hyp6 and the hyp7 syncytium.
138	WBGene00002257	W02D3.7	Metabolism	Metabolism: lipid	Metabolism: lipid: binding	lbp-5 is an ortholog of human FABP3 (fatty acid binding protein 3), FABP4 (fatty acid binding protein 4), and FABP7 (fatty acid binding protein 7)\; exhibits fatty acid binding activity\; localizes to the nucleus and perinuclear region of cytoplasm\; is expressed in several tissues, including the alimentary system, the epithelial system, the reproductive system, and the tail.
139	WBGene00002259	T22G5.2	Metabolism	Metabolism: lipid	Metabolism: lipid: binding	lbp-7 is an ortholog of human FABP3 (fatty acid binding protein 3), FABP4 (fatty acid binding protein 4), and FABP7 (fatty acid binding protein 7)\; is predicted to have lipid binding activity.
140	WBGene00002267	C44F1.3	Extracellular material	Extracellular material: galectin	Extracellular material: galectin	lec-4 is an ortholog of human LGALS9 (galectin 9) and LGALS9B (galectin 9B)\; exhibits galactoside binding activity\; localizes to the membrane raft.
141	WBGene00002497	F52H3.1	Extracellular material	Extracellular material: PGI	Extracellular material: PGI	let-268 is an ortholog of human PLOD1, PLOD2, and PLOD3\; exhibits UDP-glucose:glycoprotein glucosyltransferase activity\; is involved in collagen biosynthetic process and protein

						glycosylation\; is expressed in the GLR and the body wall musculature\; human orthologs of this gene are implicated in Bruck syndrome.
142	WBGene00003015	W03C9.4	Development	Development: somatic	Development: somatic	lin-29 is an ortholog of human MECOM, ZNF384, and ZNF362 (zinc finger protein 362)\; exhibits RNA polymerase II regulatory region sequence-specific DNA binding activity\; is involved in negative regulation of dauer larval development, regulation of development, heterochronic, and regulation of programmed cell death\; localizes to the nucleus\; is expressed in several tissues, including the epithelial system, the male, the nervous system, the reproductive system, and the set precursor.
143	WBGene00003055	F48E8.1	Signaling	Signaling: TGF beta	Signaling: TGF beta	lon-1 is an ortholog of human CRISP1 (cysteine rich secretory protein 1), CRISP2 (cysteine rich secretory protein 2), and CRISP3 (cysteine rich secretory protein 3)\; is involved in several processes, including negative regulation of DNA endoreduplication, negative regulation of

						transforming growth factor beta receptor signaling pathway, and regulation of growth\; localizes to the cell-cell adherens junction and integral component of plasma membrane\; is expressed in the head, the hypodermis, the intestine, and the tail.
144	WBGene00003075	T04H1.6	Unassigned	Unassigned	Unassigned	lrx-1 is an ortholog of human LRP10 (LDL receptor related protein 10), LRP12 (LDL receptor related protein 12), and LRP3 (LDL receptor related protein 3)\; is predicted to encode a protein with the following domains: Low-density lipoprotein (LDL) receptor class A repeat and LDL receptor-like superfamily.
145	WBGene00003095	F58B3.3	Proteolysis general	Proteolysis general: lysozyme	Proteolysis general: lysozyme	lys-6 is predicted to have lysozyme activity.
146	WBGene00003096	C02A12.4	Stress response	Stress response: pathogen	Stress response: pathogen: lysozyme	lys-7 is involved in several processes, including defense response to bacterium, defense response to other organism, and innate immune response\; is expressed in head neurons, the intestine, and the rectal gland cell.

147	WBGene00003104	ZC13.4	Development	Development: somatic	Development: somatic	mab-7 is an ortholog of human THAP4 (THAP domain containing 4)\; is expressed in the PQR, the hypodermis, the ray, and the vulva\; human THAP4 exhibits heme binding activity and protein homodimerization activity.
148	WBGene00003369	C36E6.3	Muscle function	Muscle function	Muscle function	mlc-1 is an ortholog of human MYL10, MYLPF, and MYL5\; is predicted to have calcium ion binding activity\; is involved in several processes, including locomotion, muscle organ development, and nematode larval development\; localizes to the muscle myosin complex\; is expressed in the anal depressor muscle, the body wall musculature, the pharynx, and the vulval muscle\; human orthologs of this gene are implicated in hypertrophic cardiomyopathy 10.
149	WBGene00003370	C36E6.5	Muscle function	Muscle function	Muscle function	mlc-2 is an ortholog of human MYL10, MYLPF, and MYL5\; is predicted to have calcium ion binding activity\; is involved in several processes, including locomotion, nematode larval development, and regulation of muscle

150						contraction\; is expressed in the body wall musculature, the pharynx, and the vulval muscle\; human orthologs of this gene are implicated in hypertrophic cardiomyopathy 10.
150	WBGene00003371	F09F7.2	Muscle function	Muscle function	Muscle function	mlc-3 is an ortholog of human MYL6B, MYL6, and MYL3\; is predicted to have calcium ion binding activity\; localizes to the striated muscle myosin thick filament\; is expressed in several tissues, including the muscular system, the nervous system, and the pharynx\; human orthologs of this gene are implicated in hypertrophic cardiomyopathy 8.
151	WBGene00003375	T04C9.4	Unassigned	Unassigned	Unassigned	mlp-1 is an ortholog of human CSRP1 (cysteine and glycine rich protein 1), CSRP2 (cysteine and glycine rich protein 2), and CSRP3\; is predicted to have metal ion binding activity\; localizes to the striated muscle dense body\; is expressed in several tissues, including the alimentary system, the hypodermis, the muscular system, the nervous system, and the reproductive system\; human

						orthologs of this gene are implicated in dilated cardiomyopathy 1M and hypertrophic cardiomyopathy 12.
152	WBGene00003409	E03G2.2	Transmembrane transport	Transmembrane transport: ABC	Transmembrane transport: ABC	mrp-3 is an ortholog of human ABCC2 (ATP binding cassette subfamily C member 2) and ABCC3 (ATP binding cassette subfamily C member 3)\; is predicted to have ATP binding activity and ATPase activity, coupled to transmembrane movement of substances\; human orthologs of this gene are implicated in several diseases, including Dubin-Johnson syndrome, arterial calcification of infancy, and oculocutaneous albinism.
153	WBGene00003426	F36H12.7	Major sperm protein	Major sperm protein	Major sperm protein	msp-19 is expressed in the reproductive system\; is predicted to encode a protein with the following domains: Major sperm protein (MSP) domain, PapD-like superfamily, and Immunoglobulin-like fold.
154	WBGene00003431	R05F9.8	Major sperm protein	Major sperm protein	Major sperm protein	msp-33 is expressed in the reproductive system\; is predicted to encode a protein with the following

						domains: Major sperm protein (MSP) domain, PapD-like superfamily, and Immunoglobulin-like fold.
155	WBGene00003435	C33F10.9	Major sperm protein	Major sperm protein	Major sperm protein	msp-40 is expressed in the reproductive system\; is predicted to encode a protein with the following domains: Major sperm protein (MSP) domain, PapD-like superfamily, and Immunoglobulin-like fold.
156	WBGene00003438	F58A6.8	Major sperm protein	Major sperm protein	Major sperm protein	msp-45 is expressed in the reproductive system\; is predicted to encode a protein with the following domains: Major sperm protein (MSP) domain, PapD-like superfamily, and Immunoglobulin-like fold.
157	WBGene00003444	ZK354.5	Major sperm protein	Major sperm protein	Major sperm protein	msp-51 is expressed in the reproductive system\; is predicted to encode a protein with the following domains: Major sperm protein (MSP) domain, PapD-like superfamily, and Immunoglobulin-like fold.
158	WBGene00003448	C09B9.6	Major sperm protein	Major sperm protein	Major sperm protein	msp-55 is expressed in the reproductive system\; is predicted to encode a protein with the following

						domains: Major sperm protein (MSP) domain, PapD-like superfamily, and Immunoglobulin-like fold.
159	WBGene00003450	R13H9.2	Major sperm protein	Major sperm protein	Major sperm protein	msp-57 is expressed in the reproductive system\; is predicted to encode a protein with the following domains: Major sperm protein (MSP) domain, PapD-like superfamily, and Immunoglobulin-like fold.
160	WBGene00003452	ZK354.11	Major sperm protein	Major sperm protein	Major sperm protein	msp-59 is expressed in the reproductive system\; is predicted to encode a protein with the following domains: Major sperm protein (MSP) domain, PapD-like superfamily, and Immunoglobulin-like fold.
161	WBGene00003457	ZK1248.6	Major sperm protein	Major sperm protein	Major sperm protein	msp-64 is expressed in the reproductive system\; is predicted to encode a protein with the following domains: Major sperm protein (MSP) domain, PapD-like superfamily, and Immunoglobulin-like fold.
162	WBGene00003458	ZK354.1	Major sperm protein	Major sperm protein	Major sperm protein	msp-65 is expressed in the reproductive system\; is predicted to encode a protein with the following

						domains: Major sperm protein (MSP) domain, PapD-like superfamily, and Immunoglobulin-like fold.
163	WBGene00003460	F59A6.8	Pseudogene	Pseudogene	Pseudogene	msp-71 is expressed in the reproductive system.
164	WBGene00003467	K07F5.1	Major sperm protein	Major sperm protein	Major sperm protein	msp-81 is expressed in the reproductive system\; is predicted to encode a protein with the following domains: Major sperm protein (MSP) domain, PapD-like superfamily, and Immunoglobulin-like fold.
165	WBGene00003468	ZK354.4	Major sperm protein	Major sperm protein	Major sperm protein	msp-113 is expressed in the reproductive system\; is predicted to encode a protein with the following domains: Major sperm protein (MSP) domain, PapD-like superfamily, and Immunoglobulin-like fold.
166	WBGene00003473	K11G9.6	Stress response	Stress response: heavy metal	Stress response: heavy metal	mtl-1 is predicted to have cadmium ion binding activity, copper ion binding activity, and zinc ion binding activity\; is involved in several processes, including response to cadmium ion, response to heat, and response to lead ion\; is expressed in the

						intestine and the pharynx.
167	WBGene00003485	W10G6.3	Cytoskeleton	Cytoskeleton: intermediate filament protein	Cytoskeleton: intermediate filament protein	mua-6 is an ortholog of human KRT82 (keratin 82), KRT71 (keratin 71), and KRT76 (keratin 76) and members of the human LMN (Lamins) gene family including LMNB2, LMNB1, and LMNA\; is predicted to have structural molecule activity\; is involved in cell-cell adhesion\; localizes to the hemidesmosome\; is expressed in several tissues, including the gonad, the hypodermis, and the nervous system\; human orthologs of this gene are implicated in several diseases, including Charcot-Marie-Tooth disease (multiple), eye disease (multiple), and hypotrichosis (multiple).
168	WBGene00003497	K07D8.1	Transmembrane protein	Transmembrane protein: unassigned	Transmembrane protein: unassigned	mup-4 is an ortholog of human FBN1, FBN2, and FBN3\; is predicted to have calcium ion binding activity\; localizes to the hemidesmosome and hemidesmosome associated protein complex\; is expressed in the body wall musculature, the hypodermis, the pharynx, and touch receptor neurons\; human orthologs of this gene are

						implicated in Weill-Marchesani syndrome, distal arthrogryposis, and eye disease (multiple).
169	WBGene00003523	C05D11.6	Proteolysis general	Proteolysis general: metallopeptidase	Proteolysis general: metallopeptidase	nas-4 is an ortholog of human ASTL (astacin like metalloendopeptidase)\; is predicted to have metalloendopeptidase activity and zinc ion binding activity\; is expressed in the intestine and the pharynx.
170	WBGene00003527	C34D4.9	Proteolysis general	Proteolysis general: metallopeptidase	Proteolysis general: metallopeptidase	nas-8 is an ortholog of human ASTL (astacin like metalloendopeptidase)\; is predicted to have metalloendopeptidase activity and zinc ion binding activity.
171	WBGene00003533	F09E8.6	Proteolysis general	Proteolysis general: metallopeptidase	Proteolysis general: metallopeptidase	nas-14 is an ortholog of human ASTL (astacin like metalloendopeptidase)\; is predicted to have metalloendopeptidase activity and zinc ion binding activity\; is expressed in the pharynx.
172	WBGene00003534	T04G9.2	Proteolysis general	Proteolysis general: metallopeptidase	Proteolysis general: metallopeptidase	nas-15 is predicted to have metalloendopeptidase activity and zinc ion binding activity\; is expressed in the marginal cell, the pm3, the pm4, the pm5, and the pm6.
173	WBGene0000354	Y95B8A.1	Proteolysis	Proteolysis	Proteolysis	nas-30 is predicted to have metalloendopeptidase

	8		general	general: metallopeptidase	general: metallopeptidase	activity and zinc ion binding activity\; is expressed in the intestine, the nervous system, and the rectal epithelium.
174	WBGene00003552	C26C6.3	Extracellular material	Extracellular material: unassigned	Extracellular material: unassigned	nas-36 is predicted to have metalloendopeptidase activity and zinc ion binding activity\; is involved in molting cycle\; is expressed in the hypodermis and the vulva.
175	WBGene00003554	F57C12.1	Proteolysis general	Proteolysis general: metallopeptidase	Proteolysis general: metallopeptidase	nas-38 is predicted to have metalloendopeptidase activity and zinc ion binding activity\; is expressed in the hypodermis and the reproductive system.
176	WBGene00003658	H12C20.3	Transcription factor	Transcription factor: NHR	Transcription factor: NHR	nhr-68 is predicted to have DNA-binding activity transcription factor activity, sequence-specific DNA binding activity, and zinc ion binding activity\; is expressed in several tissues, including the ABarpaaaa, the ABarpaaap, the ABarpaapa, the ABarpaapp, and the head.
177	WBGene00003738	F54F3.1	Cytoskeleton	Cytoskeleton: unassigned	Cytoskeleton: unassigned	nid-1 is an ortholog of human LRP6 (LDL receptor related protein 6), LTBP3 (latent transforming growth factor beta binding protein 3), and NID2\; exhibits extracellular matrix structural constituent\;

						is involved in several processes, including cholinergic synaptic transmission, nervous system development, and protein localization to synapse\; localizes to the basal part of cell and basement membrane\; is expressed in several tissues, including the alimentary system, the muscular system, the nervous system, the reproductive system, and the sensillum.
178	WBGene00003762	F35B12.7	Neuronal function	Neuronal function: synaptic function	Neuronal function: synaptic function: neuropeptide	nlp-24 is expressed in pharyngeal neurons, the ASIL, the ASIR, the hypodermis, and the spermatheca.
179	WBGene00003764	Y43F8C.2	Neuronal function	Neuronal function: synaptic function	Neuronal function: synaptic function: neuropeptide	nlp-26 is expressed in the hypodermis.
180	WBGene00003771	T19C4.7	Stress response	Stress response: pathogen	Stress response: pathogen: NLP	nlp-33 is involved in defense response to fungus and innate immune response.

181	WBGene00003778	C15H9.1	Stress response	Stress response: unassigned	Stress response: unassigned	nnt-1 is an ortholog of human NNT\; is predicted to have NAD(P)+ transhydrogenase activity\; localizes to the mitochondrion\; is expressed in several tissues, including the alimentary system, the coelomocyte, the hypodermis, and the nervous system.
182	WBGene00003816	F25G6.6	Metabolism	Metabolism: amino acid	Metabolism: amino acid: synthesis	asns-1 is an ortholog of human ASNS\; is predicted to have ATP binding activity and asparagine synthase (glutamine-hydrolyzing) activity.
183	WBGene00003879	F57H12.3	Unassigned	Unassigned	Unassigned: regulated by multiple stresses	ora-1 is affected by several genes including daf-16, daf-2, and glp-1 based on microarray, tiling array, and RNA-seq studies\; is affected by nineteen chemicals including methylmercury hydroxide, 1-methylnicotinamide, and Rotenone based on RNA-seq and microarray studies.
184	WBGene00003929	F54F2.1	Cytoskeleton	Cytoskeleton: integrin	Cytoskeleton: integrin	pat-2 is an ortholog of human ITGA2B, ITGA8 (integrin subunit alpha 8), and ITGAV\; exhibits transmembrane signaling receptor activity\; is involved in several processes, including mitochondrion organization, muscle cell cellular

185						homeostasis, and positive regulation of cellular component organization\; localizes to the M band, cell surface, and striated muscle dense body\; is expressed in several tissues, including the hypodermis, the muscular system, the nervous system, and the reproductive system\; human orthologs of this gene are implicated in Glanzmann's thrombasthenia, platelet-type bleeding disorder 16, and renal agenesis.
185	WBGene0000393 4	F54C1.7	Muscle function	Muscle function	Muscle function	pat-10 exhibits troponin I binding activity\; is involved in embryo development, muscle contraction, and skeletal muscle thin filament assembly\; localizes to the contractile fiber\; is expressed in the body wall musculature, the gonadal sheath cell, the rectal muscle, and the vulval muscle.
186	WBGene0000395 6	ZK112.1	Proteolysis general	Proteolysis general: carboxypeptidase	Proteolysis general: carboxypeptidase	pcp-1 is an ortholog of human PRCP\; is predicted to have serine-type peptidase activity\; is expressed in the intestine.
187	WBGene0000398	F18G5.2	Transmembran	Transmembrane	Transmembrane	pes-8 is expressed in several tissues, including the

	1		e protein	protein: unassigned	protein: unassigned	alimentary system and the gonad.
188	WBGene0000399 1	K03E6.6	Muscle function	Muscle function	Muscle function	pfn-3 is predicted to have actin binding activity\; is involved in muscle thin filament assembly\; localizes to the striated muscle dense body\; is expressed in the body wall musculature, the intestine, and the rectal gland cell.
189	WBGene0000399 5	K08E7.9	Transmembran e transport	Transmembrane transport: ABC	Transmembrane transport: ABC	pgp-1 is an ortholog of human ABCB1 (ATP binding cassette subfamily B member 1), ABCB11 (ATP binding cassette subfamily B member 11), and ABCB4 (ATP binding cassette subfamily B member 4)\; is predicted to have efflux transmembrane transporter activity\; is involved in several processes, including defense response to Gram-negative bacterium, innate immune response, and response to metal ion\; localizes to the apical plasma membrane\; is expressed in the intestine and the pharynx.
190	WBGene0000399 6	C34G6.4	Transmembran e transport	Transmembrane transport: ABC	Transmembrane transport: ABC	pgp-2 is predicted to have ATP binding activity and ATPase activity, coupled to transmembrane

						movement of substances\; is involved in lipid storage and organelle organization\; localizes to the gut granule membrane\; is expressed in several tissues, including the Eala, the Ealp, the Eara, the alimentary system, and the nervous system.
191	WBGene00004017	F27E5.4	Unassigned	Unassigned	Unassigned	phg-1 is involved in multicellular organism development and negative regulation of cell cycle G1\/S phase transition\; localizes to the anchored component of plasma membrane\; is expressed in the muscle cell and the pharynx.
192	WBGene00004098	AC3.4	Unassigned	Unassigned	Unassigned: prion domain	pqn-2 is enriched in the pharynx based on RNA-seq studies\; is affected by several genes including clk-1, ced-1, and npr-1 based on microarray, tiling array, and RNA-seq studies\; is affected by five chemicals including aldicarb, Ethanol, and methylmercury hydroxide based on microarray and RNA-seq studies.
193	WBGene00004099	C03A7.4	Unassigned	Unassigned	Unassigned: prion domain	abu-15 is involved in endoplasmic reticulum unfolded protein response and pharynx development\; is expressed in the pharyngeal

						muscle cell.
194	WBGene00004112	C46G7.4	Unassigned	Unassigned	Unassigned: prion domain	pqn-22 is an ortholog of human PRICKLE1 (prickle planar cell polarity protein 1), PRICKLE2 (prickle planar cell polarity protein 2), and PRICKLE3 (prickle planar cell polarity protein 3)\; is predicted to encode a protein with the following domain: Zasp-like motif.
195	WBGene00004127	F52D1.3	Unassigned	Unassigned	Unassigned: prion domain	abu-12 is enriched in the OLL, the PVD, and the hypodermis based on microarray and tiling array studies\; is affected by several genes including daf-16, daf-12, and dpy-10 based on microarray, tiling array, and RNA-seq studies\; is affected by eleven chemicals including hydrogen sulfide, methylmercury hydroxide, and 1-methylnicotinamide based on microarray and RNA-seq studies.
196	WBGene00004133	F57B9.9	Unassigned	Unassigned	Unassigned: prion domain	abu-13 is enriched in the OLL, the PLM, the PVD, and the hypodermis based on microarray, tiling array, and RNA-seq studies\; is affected by several genes including daf-16, daf-2, and skn-1 based on

197						microarray and RNA-seq studies\; is affected by fifteen chemicals including Ethanol, Nitric Oxide, and methylmercury hydroxide based on RNA-seq and microarray studies.
197	WBGene00004139	R09B5.5	Unassigned	Unassigned	Unassigned: prion domain	pqn-54 is enriched in the DA neuron and the pharynx based on microarray studies\; is affected by several genes including daf-16, glp-1, and skn-1 based on microarray, tiling array, and RNA-seq studies\; is affected by twenty chemicals including hydrogen sulfide, Ethanol, and methylmercury hydroxide based on microarray and RNA-seq studies.
198	WBGene00004146	T06E4.11	Unassigned	Unassigned	Unassigned: prion domain	pqn-63 is enriched in the DA neuron and the pharynx based on microarray studies\; is affected by several genes including daf-16, skn-1, and daf-12 based on microarray, tiling array, and RNA-seq studies\; is affected by nineteen chemicals including cholesterol, lathosterol, and hydrogen sulfide based on microarray and RNA-seq studies.
199	WBGene0000419	ZK809.7	Peroxisome	Peroxisome:	Peroxisome:	prx-2 is an ortholog of human PEX2 (peroxisomal

	2			biogenesis	biogenesis	biogenesis factor 2)\; is predicted to encode a protein with the following domains: Zinc finger, RING-type, Pex, N-terminal, and Zinc finger, RING\/FYVE\/PHD-type.
200	WBGene00004216	C24B5.3	Signaling	Signaling: hedgehog-like	Signaling: hedgehog-like	ptr-1 is an ortholog of human PTCHD1 and PTCHD4\; is involved in molting cycle and nematode male tail tip morphogenesis.
201	WBGene00004219	C45B2.7	Signaling	Signaling: hedgehog-like	Signaling: hedgehog-like	ptr-4 is an ortholog of human PTCHD1 and PTCHD4\; is involved in endocytosis, molting cycle, and nematode male tail tip morphogenesis.
202	WBGene00004225	F56C11.2	Signaling	Signaling: hedgehog-like	Signaling: hedgehog-like	ptr-11 is an ortholog of human PTCHD1 and PTCHD4\; is involved in molting cycle.
203	WBGene00004228	R09H10.4	Signaling	Signaling: hedgehog-like	Signaling: hedgehog-like	ptr-14 is an ortholog of human PTCHD1 and PTCHD4\; is predicted to have transmembrane transporter activity\; is involved in molting cycle.
204	WBGene00004230	T21H3.2	Signaling	Signaling: hedgehog-like	Signaling: hedgehog-like	ptr-16 is an ortholog of human PTCHD1 and PTCHD4\; is predicted to encode a protein with the following domain: Protein patched\/dispatched.
205	WBGene00004237	ZK270.1	Signaling	Signaling: hedgehog-like	Signaling: hedgehog-like	ptr-23 is an ortholog of human PTCHD1 and PTCHD4\; is involved in endocytosis, molting

						cycle, and nematode male tail tip morphogenesis\; is expressed in the hypodermis.
206	WBGene00004256	ZK994.3	Metabolism	Metabolism: unassigned	Metabolism: unassigned	pxn-1 is an ortholog of human PXDN (peroxidasin) and PXDNL (peroxidasin like)\; is predicted to have heme binding activity, metal ion binding activity, and peroxidase activity\; localizes to the basement membrane\; is expressed in several tissues, including the excretory cell, the hypodermis, the intestine, the muscular system, and the nervous system.
207	WBGene00004264	T05C12.10	Signaling	Signaling: hedgehog-like	Signaling: hedgehog-like	qua-1 is predicted to have peptidase activity\; is involved in molting cycle and nematode larval development\; localizes to the collagen and cuticulin-based cuticle extracellular matrix\; is expressed in several tissues, including the alimentary system, the epithelial system, the muscular system, the reproductive system, and the ventral cord blast cell.
208	WBGene00004394	Y57A10A.11	Extracellular material	Extracellular material:	Extracellular material:	rol-1 is predicted to be a structural constituent of cuticle.

				collagen	collagen	
209	WBGene00004397	T01B7.7	Extracellular material	Extracellular material: collagen	Extracellular material: collagen	rol-6 is an ortholog of human COLQ\; is a structural constituent of collagen and cuticulin-based cuticle\; is involved in cuticle development involved in collagen and cuticulin-based cuticle molting cycle\; localizes to the collagen and cuticulin-based cuticle extracellular matrix\; is expressed in the hypodermis.
210	WBGene00004398	ZK1290.3	Extracellular material	Extracellular material: collagen	Extracellular material: collagen	rol-8 is an ortholog of human MARCO (macrophage receptor with collagenous structure)\; is predicted to be a structural constituent of cuticle.
211	WBGene00004890	D1037.2	Development	Development: somatic	Development: somatic	smp-2 is an ortholog of human SEMA4D, SEMA4G, and SEMA4A (semaphorin 4A)\; is involved in nematode male tail mating organ morphogenesis\; is expressed in several tissues, including inner labial neurons, the alimentary system, the male, the muscular system, and the reproductive system\; human orthologs of this gene are implicated in CHARGE syndrome and hypogonadotropic hypogonadism 16 with or

						without anosmia.
212	WBGene00004904	Y46G5A.30	Transmembrane transport	Transmembrane transport: solute carrier	Transmembrane transport: solute carrier	snf-5 is an ortholog of human SLC6A16 (solute carrier family 6 member 16) and SLC6A7 (solute carrier family 6 member 7)\; is predicted to have metal ion binding activity and neurotransmitter:sodium symporter activity\; is expressed in several tissues, including the alimentary system and the nervous system.
213	WBGene00004932	C08A9.1	Stress response	Stress response: oxidative	Stress response: oxidative	sod-3 is an ortholog of human Sod2\; exhibits protein homodimerization activity and superoxide dismutase activity\; is involved in removal of superoxide radicals\; localizes to the mitochondrial respiratory chain supercomplex\; is expressed in several tissues, including the alimentary system, the head, the tail, and the vulva.
214	WBGene00004964	AC3.10	Transmembrane protein	Transmembrane protein: unassigned	Transmembrane protein: unassigned	spe-10 is an ortholog of human ZDHHC2 (zinc finger DHHC-type containing 2) and ZDHHC20 (zinc finger DHHC-type containing 20)\; is predicted to have protein-cysteine S-palmitoyltransferase activity\; is involved in several

						processes, including flagellated sperm motility, membrane fusion, and nuclear migration\; localizes to the membrane-bounded organelle\; is expressed in the spermatid.
215	WBGene00004965	F48C1.7	Development	Development: general	Development: general	spe-11 is involved in eggshell formation and prevention of polyspermy\; localizes to the nucleus.
216	WBGene00004987	T08A9.12	Stress response	Stress response: pathogen	Stress response: pathogen: saposin	spp-2 is involved in defense response to Gram-positive bacterium.
217	WBGene00004993	C28C12.5	Stress response	Stress response: pathogen	Stress response: pathogen: saposin	spp-8 is an ortholog of human PSAP (prosaposin) and PSAPL1\; human PSAP exhibits several functions, including anion binding activity, beta-galactosidase activity, and protein homodimerization activity.
218	WBGene00004998	F08F1.6	Stress response	Stress response: pathogen	Stress response: pathogen: saposin	spp-13 is an ortholog of human PSAP (prosaposin), PSAPL1, and SFTPB\; human PSAP exhibits several functions, including anion binding activity, beta-galactosidase activity, and protein homodimerization activity.
219	WBGene0000500	F27C8.4	Stress response	Stress response:	Stress response:	spp-18 is involved in innate immune response.

	3			pathogen	pathogen: saposin	
220	WBGene00005017	C01B12.1	Extracellular material	Extracellular material: collagen	Extracellular material: collagen	sqt-2 is an ortholog of human MARCO (macrophage receptor with collagenous structure)\; is predicted to be a structural constituent of cuticle\; is involved in cuticle development involved in collagen and cuticulin-based cuticle molting cycle.
221	WBGene00005644	Y32G9A.4	Proteolysis general	Proteolysis general: inhibitor	Proteolysis general: inhibitor: serine	srp-3 is an ortholog of human SERPINE1 (serpin family E member 1), SERPINB5 (serpin family B member 5), and SERPINE2 (serpin family E member 2)\; is predicted to have peptidase activity\; is expressed in the body wall musculature, the intestine, and the muscle cell.
222	WBGene00006050	K07F5.11	Unassigned	Unassigned	Unassigned	ssq-1 is enriched in the amphid sheath cell, the germ line, the intestine, and the male based on RNA-seq and microarray studies\; is affected by several genes including daf-2, age-1, and dpy-10 based on microarray and RNA-seq studies\; is affected by eighteen chemicals including Ethanol, methylmercury hydroxide, and nicotinic acid based

						on RNA-seq and microarray studies.
223	WBGene00006051	T28H11.5	Unassigned	Unassigned	Unassigned: regulated by multiple stresses	ssq-2 is enriched in the amphid sheath cell and the intestine based on RNA-seq and microarray studies\; is affected by several genes including daf-2, age-1, and daf-12 based on microarray and RNA-seq studies\; is affected by twenty chemicals including Ethanol, methylmercury hydroxide, and 1-methylnicotinamide based on RNA-seq and microarray studies.
224	WBGene00006060	ZC513.12	Unassigned	Unassigned	Unassigned	sth-1 localizes to the plasma membrane\; is expressed in the germ line and the spermatheca.
225	WBGene00006310	C54D2.4	Metabolism	Metabolism: unassigned	Metabolism: unassigned	sul-3 is an ortholog of human ARSB (arylsulfatase B), ARSI (arylsulfatase I), and ARSJ (arylsulfatase J)\; is predicted to have sulfuric ester hydrolase activity\; is expressed in several tissues, including the body wall musculature, the epithelial system, the nervous system, and the reproductive system\; human orthologs of this gene are implicated in mucopolysaccharidosis VI.
226	WBGene0000640	T14G12.3	Unassigned	Unassigned	Unassigned	tag-18 is expressed in the anal depressor muscle,

	8					the body wall musculature, and the reproductive system.
227	WBGene00006416	T13A10.11	Metabolism	Metabolism: 1CC	Metabolism: 1CC: methionine	sams-5 is an ortholog of human MAT1A and MAT2A\; is predicted to have ATP binding activity, metal ion binding activity, and methionine adenosyltransferase activity\; is expressed in several tissues, including the alimentary system, the excretory gland cell, the nervous system, and the reproductive system.
228	WBGene00006487	C30H6.2	Transmembrane transport	Transmembrane transport: solute carrier	Transmembrane transport: solute carrier	zipt-17 is an ortholog of human SLC39A12 (solute carrier family 39 member 12) and SLC39A4 (solute carrier family 39 member 4)\; is predicted to have metal ion transmembrane transporter activity\; is expressed in several tissues, including the alimentary system, the hypodermis, and the reproductive system\; human orthologs of this gene are implicated in acrodermatitis enteropathica.
229	WBGene00006585	T20B3.2	Muscle function	Muscle function	Muscle function	tni-3 is an ortholog of human TNNI1 (troponin I1, slow skeletal type), TNNI2 (troponin I2, fast skeletal type), and TNNI3\; exhibits troponin C

						binding activity\; is involved in muscle contraction, oviposition, and post-embryonic body morphogenesis\; localizes to the sarcomere\; is expressed in several tissues, including the male, the muscular system, and the rectal muscle\; human orthologs of this gene are implicated in distal arthrogryposis and intrinsic cardiomyopathy (multiple).
230	WBGene00006587	F53A9.10	Muscle function	Muscle function	Muscle function	tnt-2 is an ortholog of human TNNT1, TNNT2 (troponin T2, cardiac type), and TNNT3 (troponin T3, fast skeletal type)\; is expressed in the muscular system and the reproductive system\; human TNNT3 exhibits calcium-dependent protein binding activity, troponin C binding activity, and troponin I binding activity.
231	WBGene00006609	W05E10.4	Metabolism	Metabolism: carbohydrate	Metabolism: carbohydrate	tre-3 is an ortholog of human TREH (trehalase)\; is predicted to have alpha,alpha-trehalase activity\; localizes to the membrane raft\; is expressed in several tissues, including the alimentary system, the body wall musculature, the head, the nervous

						system, and the reproductive system.
232	WBGene00006610	F15A2.2	Metabolism	Metabolism: carbohydrate	Metabolism: carbohydrate	tre-4 is an ortholog of human TREH (trehalase)\; is predicted to have alpha,alpha-trehalase activity\; is expressed in several tissues, including the alimentary system, the excretory cell, the hypodermis, the nervous system, and the reproductive system.
233	WBGene00006677	F55C5.3	Transmembrane transport	Transmembrane transport: potassium channel	Transmembrane transport: potassium channel	twk-24 is predicted to have potassium channel activity.
234	WBGene00006679	C33D12.3	Transmembrane transport	Transmembrane transport: potassium channel	Transmembrane transport: potassium channel	twk-26 is predicted to have potassium channel activity.
235	WBGene00006759	ZK617.1	Extracellular material	Extracellular material: matrix	Extracellular material: matrix	unc-22 exhibits protein kinase binding activity and protein serine\/threonine kinase activity\; is involved in several processes, including adult locomotory behavior, behavioral response to nicotine, and cellular protein modification process\;

						localizes to the A band\; is expressed in the body wall musculature, the pharyngeal muscle cell, and the vulval muscle.
236	WBGene00006764	ZK721.2	Muscle function	Muscle function	Muscle function	unc-27 is an ortholog of human TNNI1 (troponin I1, slow skeletal type), TNNI2 (troponin I2, fast skeletal type), and TNNI3\; exhibits troponin C binding activity\; is involved in locomotion and sarcomere organization\; localizes to the sarcomere\; is expressed in several tissues, including the alimentary system and the muscular system\; human orthologs of this gene are implicated in distal arthrogryposis and intrinsic cardiomyopathy (multiple).
237	WBGene00006819	F08B6.4	Muscle function	Muscle function	Muscle function	unc-87 exhibits actin filament binding activity and myosin binding activity\; is involved in several processes, including actin cytoskeleton organization, negative regulation of muscle filament sliding, and negative regulation of plus-end directed microfilament motor activity\; localizes to the cytoplasmic part and intracellular

						organelle\; is expressed in several tissues, including the alimentary system, the head, the muscular system, and the reproductive system.
238	WBGene00006826	F14D12.2	Cytoskeleton	Cytoskeleton: unassigned	Cytoskeleton: unassigned	unc-97 is an ortholog of human LIMS1 (LIM zinc finger domain containing 1) and LIMS2 (LIM zinc finger domain containing 2)\; exhibits myosin binding activity\; is involved in several processes, including cell-cell junction organization, mitochondrion organization, and muscle cell cellular homeostasis\; localizes to several cellular components, including the adherens junction, basal plasma membrane, and intracellular organelle\; colocalizes with the integrin complex\; is expressed in the muscular system and the nervous system.
239	WBGene00006920	Y38F2AL.3	Lysosome	Lysosome: Vacuolar ATPase	Lysosome: Vacuolar ATPase	vha-11 is an ortholog of human ATP6V1C1 (ATPase H+ transporting V1 subunit C1) and ATP6V1C2 (ATPase H+ transporting V1 subunit C2)\; is predicted to have proton transmembrane transporter activity\; is involved in embryo development and ovulation\; localizes to the

						intracellular organelle\; is expressed in the excretory cell, the hypodermis, and the intestinal cell.
240	WBGene00006950	ZK678.5	Signaling	Signaling: hedgehog-like	Signaling: hedgehog-like	wrt-4 is predicted to have peptidase activity\; is expressed in the hyp10, the hyp6, the hyp7 syncytium, the hyp8, and the hyp9.
241	WBGene00006952	ZK377.1	Signaling	Signaling: hedgehog-like	Signaling: hedgehog-like	wrt-6 is predicted to have peptidase activity\; is expressed in the epithelial system, the nervous system, and the sensillum.
242	WBGene00006954	C29F3.2	Signaling	Signaling: hedgehog-like	Signaling: hedgehog-like	wrt-8 is predicted to have peptidase activity\; is expressed in the hypodermis and the ventral cord blast cell.
243	WBGene00007071	AC3.5	Proteolysis general	Proteolysis general: unassigned	Proteolysis general: unassigned	AC3.5 is an ortholog of human NPEPPS (aminopeptidase puromycin sensitive)\; is predicted to have metallopeptidase activity and zinc ion binding activity.
244	WBGene00007072	AC3.7	Stress response	Stress response: detoxification	Stress response: detoxification: ugt	ugt-1 is an ortholog of human UGT3A1 (UDP glycosyltransferase family 3 member A1) and UGT3A2 (UDP glycosyltransferase family 3 member A2)\; is predicted to have transferase

						activity, transferring hexosyl groups.
245	WBGene00007142	B0334.1	Unassigned	Unassigned	Unassigned: TransThyretin-Related family domain	ttr-18 is enriched in the PLM, the cephalic sheath cell, the coelomocyte, the hypodermis, and the intestine based on tiling array, RNA-seq, and microarray studies\; is affected by several genes including daf-16, daf-2, and glp-1 based on microarray, proteomic, and RNA-seq studies\; is affected by nine chemicals including Rotenone, D-glucose, and nicotine based on RNA-seq, proteomic, and microarray studies\; is predicted to encode a protein with the following domains: Transthyretin-like and Transthyretin-like superfamily.
246	WBGene00007155	B0379.1	Unassigned	Unassigned	Unassigned: regulated by multiple stresses	B0379.1 is enriched in the body wall musculature, the germ line, and the muscle cell based on proteomic, tiling array, and microarray studies\; is affected by several genes including daf-2, dpy-10, and clk-1 based on microarray and RNA-seq studies\; is affected by seven chemicals including Rotenone, D-glucose, and Chlorpyrifos based on

						RNA-seq and microarray studies.
247	WBGene00007159	B0379.7	Unassigned	Unassigned	Unassigned: regulated by multiple stresses	B0379.7 is enriched in the arcade cell, the germ line, the male, the muscle cell, and the pharyngeal-intestinal valve cell based on RNA-seq and microarray studies\; is affected by several genes including daf-2, dpy-10, and eat-2 based on proteomic, microarray, and RNA-seq studies\; is affected by fourteen chemicals including Ethanol, methylmercuric chloride, and Rotenone based on RNA-seq and microarray studies.
248	WBGene00007228	C01G6.7	Metabolism	Metabolism: lipid	Metabolism: lipid: fatty acid	acs-7 is predicted to have catalytic activity\; is expressed in the intestinal cell.
249	WBGene00007254	C01H6.4	Metabolism	Metabolism: unassigned	Metabolism: unassigned	C01H6.4 is predicted to have N,N-dimethylaniline monooxygenase activity, NADP binding activity, and flavin adenine dinucleotide binding activity\; is expressed in the intestine and the nervous system.
250	WBGene00007264	C02F4.4	Unassigned	Unassigned	Unassigned: regulated by multiple stresses	C02F4.4 is enriched in the ASER, the PLM, the germline precursor cell, and the hypodermis based on tiling array and RNA-seq studies\; is affected by several genes including daf-16, daf-12, and lin-4

						based on RNA-seq and microarray studies\; is affected by eleven chemicals including Ethanol, methylmercury hydroxide, and D-glucose based on RNA-seq and microarray studies.
251	WBGene00007269	C03C10.2	Cytoskeleton	Cytoskeleton: microtubule	Cytoskeleton: microtubule: tau tubulin kinase	C03C10.2 is an ortholog of human CTD-2116N17.1, TTBK1 (tau tubulin kinase 1), and TTBK2\; is predicted to have ATP binding activity and protein serine\/threonine kinase activity.
252	WBGene00007307	C04G2.8	Transcription: chromatin	Transcription: chromatin structure	Transcription: chromatin structure: unassigned	spch-1 is involved in male meiotic nuclear division\; localizes to the nuclear chromatin\; is expressed in the sperm.
253	WBGene00007311	C04H4.1	Unassigned	Unassigned	Unassigned	C04H4.1 is enriched in the nervous system based on tiling array studies\; is affected by rsr-2, csr-1, and mir-34 based on tiling array, RNA-seq, and microarray studies.
254	WBGene00007340	C05D12.2	Metabolism	Metabolism: amino sugar	Metabolism: amino sugar	C05D12.2 is enriched in the intestine based on RNA-seq studies\; is affected by several genes including daf-16, daf-2, and glp-1 based on microarray and RNA-seq studies\; is affected by

						five chemicals including Rotenone, Zidovudine, and Quercetin based on RNA-seq and microarray studies.
255	WBGene00007343	C05E7.1	Unassigned	Unassigned	Unassigned: regulated by multiple stresses	C05E7.1 is enriched in GABAergic neurons, the germline precursor cell, and the hypodermis based on microarray, tiling array, and RNA-seq studies\; is affected by several genes including daf-16, daf-12, and dpy-10 based on tiling array, RNA-seq, and microarray studies\; is affected by twelve chemicals including 1-methylnicotinamide, D-glucose, and Neurotoxins based on RNA-seq and microarray studies\; is predicted to encode a protein with the following domain: GM2-AP, lipid-recognition domain superfamily.
256	WBGene00007344	C05E7.2	Proteolysis general	Proteolysis general: unassigned	Proteolysis general: unassigned	C05E7.2 is enriched in the DA neuron and the hypodermis based on tiling array, RNA-seq, and microarray studies\; is affected by several genes including daf-16, daf-2, and glp-1 based on proteomic, tiling array, RNA-seq, and microarray studies\; is affected by eighteen chemicals

						including Heme, aldicarb, and 1-methylnicotinamide based on microarray and RNA-seq studies\; is predicted to encode a protein with the following domain: GM2-AP, lipid-recognition domain superfamily.
257	WBGene00007362	C06B3.3	Stress response	Stress response: detoxification	Stress response: detoxification: CYP	cyp-35C1 is an ortholog of human CYP21A2 (cytochrome P450 family 21 subfamily A member 2) and members of the human CYP2 (Cytochrome P450 family 2) gene family including CYP2R1, CYP2U1, and CYP2J2\; is predicted to have heme binding activity, iron ion binding activity, and oxidoreductase activity\; is involved in response to xenobiotic stimulus\; human orthologs of this gene are implicated in congenital adrenal hyperplasia.
258	WBGene00007446	C08F8.4	Metabolism	Metabolism: lipid	Metabolism: lipid: phospholipid	mboa-4 is an ortholog of human MBOAT2 (membrane bound O-acyltransferase domain containing 2)\; is predicted to have transferase activity, transferring acyl groups.
259	WBGene00007461	C08F11.14	Pseudogene	Pseudogene	Pseudogene	C08F11.14 is enriched in the male based on RNA-seq studies\; is affected by several genes including

						clk-1, eat-2, and npr-1 based on microarray and RNA-seq studies\; is affected by eight chemicals including hydrogen sulfide, methylmercuric chloride, and multi-walled carbon nanotube based on microarray and RNA-seq studies.
260	WBGene00007544	C12D8.17	Unassigned	Unassigned	Unassigned: regulated by multiple stresses	fipr-6 is affected by several genes including daf-16, skn-1, and daf-12 based on RNA-seq and microarray studies\; is affected by D-glucose and Zidovudine based on RNA-seq studies.
261	WBGene00007549	C13C4.5	Metabolism	Metabolism: lipid	Metabolism: lipid: sphingolipid	spin-1 is an ortholog of human SPNS1, SPNS2, and SPNS3\; is expressed in several tissues, including the alimentary system, the excretory cell, the hypodermis, the nervous system, and the reproductive system\; is predicted to encode a protein with the following domains: Major facilitator superfamily and MFS transporter superfamily.
262	WBGene00007565	C14A6.1	Stress response	Stress response: C-type Lectin	Stress response: C-type Lectin	clec-48 is an ortholog of human COLEC11, CD302, and LY75\; is predicted to have carbohydrate binding activity.

263	WBGene00007584	C14C10.1	Transmembrane transport	Transmembrane transport: mitochondrial	Transmembrane transport: mitochondrial: solute carrier	C14C10.1 is an ortholog of human SLC25A3 (solute carrier family 25 member 3)\; is predicted to encode a protein with the following domains: Mitochondrial substrate\/solute carrier and Mitochondrial carrier domain superfamily.
264	WBGene00007591	C14H10.1	Transmembrane transport	Transmembrane transport: solute carrier	Transmembrane transport: solute carrier	zipt-13 is an ortholog of human SLC39A13 (solute carrier family 39 member 13)\; is predicted to have metal ion transmembrane transporter activity.
265	WBGene00007593	C14H10.3	Unassigned	Unassigned	Unassigned	C14H10.3 is an ortholog of human PDXDC1\; is predicted to have catalytic activity.
266	WBGene00007687	C18E9.7	Unassigned	Unassigned	Unassigned: regulated by multiple stresses	C18E9.7 is affected by several genes including daf-16, skn-1, and daf-12 based on microarray, tiling array, and RNA-seq studies\; is affected by eight chemicals including 1-methylnicotinamide, D-glucose, and stavudine based on RNA-seq and microarray studies\; is predicted to encode a protein with the following domains: Fibronectin type III, Immunoglobulin-like fold, and Fibronectin type III superfamily.
267	WBGene0000769	C23H4.3	Transmembran	Transmembrane	Transmembrane	C23H4.3 is enriched in the intestine based on

	2		e protein	protein: unassigned	protein: unassigned	RNA-seq studies\; is affected by several genes including daf-16, hsf-1, and clk-1 based on microarray and RNA-seq studies\; is affected by Cadmium, Cry5B, and Sirolimus based on microarray studies\; is predicted to encode a protein with the following domains: Carboxylesterase, type B and Alpha\/Beta hydrolase fold.
268	WBGene00007724	C25F9.4	Unassigned	Unassigned	Unassigned	C25F9.4 is predicted to have ATP binding activity.
269	WBGene00007755	C27A7.3	Metabolism	Metabolism: nucleotide	Metabolism: nucleotide	C27A7.3 is an ortholog of human ENPP1, ENPP2, and ENPP3 (ectonucleotide pyrophosphatase\/phosphodiesterase 3)\; is predicted to have hydrolase activity and metal ion binding activity\; human orthologs of this gene are implicated in several diseases, including arterial calcification of infancy, autosomal recessive hypophosphatemic rickets, and obesity.
270	WBGene00007794	C28D4.7	Unassigned	Unassigned	Unassigned: regulated by multiple stresses	C28D4.7 is enriched in the germ line and the male based on RNA-seq studies\; is affected by several genes including dpy-10, hsf-1, and eat-2 based on

						microarray, RNA-seq, and tiling array studies\; is affected by fourteen chemicals including Ethanol, methylmercuric chloride, and D-glucose based on RNA-seq and microarray studies\; is predicted to encode a protein with the following domain: Domain of unknown function DUF1248.
271	WBGene00007808	C29F4.2	Transmembrane transport	Transmembrane transport: chloride channel	Transmembrane transport: chloride channel	best-7 is an ortholog of human BEST2 (bestrophin 2) and BEST4 (bestrophin 4)\; is predicted to have chloride channel activity\; human orthologs of this gene are implicated in bestrophinopathy, retinitis pigmentosa 50, and vitelliform macular dystrophy.
272	WBGene00007823	C30H6.5	Unassigned	Unassigned	Unassigned: regulated by multiple stresses	C30H6.5 is enriched in the AFD and the hypodermis based on tiling array and RNA-seq studies\; is affected by several genes including daf-16, daf-12, and lin-4 based on tiling array, microarray, and RNA-seq studies\; is affected by sixteen chemicals including tryptophan, 1-methylnicotinamide, and nicotinic acid based on microarray and RNA-seq studies\; is predicted to encode a protein with the following domain:

						PAN\/Apple domain.
273	WBGene00007868	C32H11.5	Unassigned	Unassigned	Unassigned	C32H11.5 is enriched in GABAergic neurons, the ALM, the PLM, and the pharynx based on microarray and RNA-seq studies\; is affected by several genes including daf-16, daf-12, and lin-4 based on microarray and RNA-seq studies\; is affected by six chemicals including 1-methylnicotinamide, nicotinic acid, and D-glucose based on RNA-seq and microarray studies\; is predicted to encode a protein with the following domain: Domain of unknown function DUF148.
274	WBGene00007903	C33G3.3	Transmembrane transport	Transmembrane transport: extracellular ligand gated channel	Transmembrane transport: extracellular ligand gated channel	lgc-21 is an ortholog of human CHRNB3 (cholinergic receptor nicotinic beta 3 subunit), CHRNA4 (cholinergic receptor nicotinic alpha 4 subunit), and CHRNA1 (cholinergic receptor nicotinic alpha 1 subunit)\; is predicted to have extracellular ligand-gated ion channel activity and transmembrane signaling receptor activity.
275	WBGene00007930	C34D1.2	Transcription factor	Transcription factor: ZF	Transcription factor: ZF	dmd-11 is an ortholog of human DMRT3, DMRTA1, and DMRTC1\; is predicted to have

						DNA-binding activity transcription factor activity, metal ion binding activity, and sequence-specific DNA binding activity\; is expressed in head neurons.
276	WBGene00007934	C34E7.4	Unassigned	Unassigned	Unassigned	C34E7.4 is enriched in the PLM and the hypodermis based on tiling array and RNA-seq studies\; is affected by several genes including daf-16, daf-2, and daf-12 based on microarray, RNA-seq, and proteomic studies\; is affected by nine chemicals including D-glucose, bisphenol S, and Neurotoxins based on RNA-seq and microarray studies\; is predicted to encode a protein with the following domain: GM2-AP, lipid-recognition domain superfamily.
277	WBGene00007938	C34F6.1	Proteolysis general	Proteolysis general: inhibitor	Proteolysis general: inhibitor: serine	C34F6.1 is predicted to have serine-type endopeptidase inhibitor activity.
278	WBGene00007947	C35A5.3	Transmembrane transport	Transmembrane transport: solute carrier	Transmembrane transport: solute carrier	C35A5.3 is enriched in the coelomocyte, the epithelial system, the germline precursor cell, and the nervous system based on tiling array and RNA-

279						seq studies\; is affected by several genes including daf-2, glp-1, and skn-1 based on microarray, RNA-seq, and proteomic studies\; is affected by twenty-two chemicals including cholesterol, lathosterol, and methylmercuric chloride based on microarray and RNA-seq studies\; is predicted to encode a protein with the following domains: Major facilitator superfamily and MFS transporter superfamily.
279	WBGene00007963	C36A4.1	Stress response	Stress response: detoxification	Stress response: detoxification: CYP	cyp-25A1 is an ortholog of human CYP3A7-CYP3A51P and TBXAS1 (thromboxane A synthase 1) and members of the human CYP3 (Cytochrome P450 family 3) gene family including CYP3A4, CYP3A43, and CYP3A7\; is predicted to have heme binding activity, iron ion binding activity, and oxidoreductase activity\; human orthologs of this gene are implicated in essential hypertension and platelet-type bleeding disorder 14.
280	WBGene00007964	C36A4.2	Stress response	Stress response: detoxification	Stress response: detoxification:	cyp-25A2 is an ortholog of human CYP3A7-CYP3A51P and TBXAS1 (thromboxane A synthase

					CYP	1) and members of the human CYP3 (Cytochrome P450 family 3) gene family including CYP3A4, CYP3A43, and CYP3A7\; is predicted to have heme binding activity, iron ion binding activity, and oxidoreductase activity\; human orthologs of this gene are implicated in essential hypertension and platelet-type bleeding disorder 14.
281	WBGene00007996	C38C6.3	Unassigned	Unassigned	Unassigned: regulated by multiple stresses	C38C6.3 is enriched in the PLM, the body wall musculature, the germline precursor cell, and the hypodermis based on tiling array, RNA-seq, and microarray studies\; is affected by several genes including daf-16, daf-12, and dpy-10 based on tiling array, microarray, and RNA-seq studies\; is affected by thirteen chemicals including methylmercury hydroxide, 1-methylnicotinamide, and Alovudine based on RNA-seq and microarray studies.
282	WBGene00007999	C38C6.6	Unassigned	Unassigned	Unassigned	tag-297 is expressed in head neurons and the hypodermis.
283	WBGene0000803	C40C9.3	Unassigned	Unassigned	Unassigned	C40C9.3 is enriched in the coelomocyte based on

	6					tiling array and RNA-seq studies\; is affected by several genes including hsf-1, eat-2, and pmk-1 based on microarray, tiling array, and RNA-seq studies\; is affected by Rotenone, resveratrol, and adsorbable organic bromine compound based on RNA-seq and microarray studies.
284	WBGene00008047	C40H5.4	Unassigned	Unassigned	Unassigned	lact-7 is affected by several genes including daf-16, daf-12, and sir-2.1 based on microarray, tiling array, and RNA-seq studies\; is affected by eight chemicals including D-glucose, stavudine, and resveratrol based on RNA-seq and microarray studies\; is predicted to encode a protein with the following domains: Beta-lactamase-related and Beta-lactamase\/transpeptidase-like.
285	WBGene00008150	C47E12.6	Unassigned	Unassigned	Unassigned: regulated by multiple stresses	mam-2 is enriched in several tissues, including the ASER, the Z1.p, the Z4.a, the reproductive system, and the somatic gonad precursor based on tiling array and RNA-seq studies\; is affected by several genes including lin-4, lin-14, and dpy-10 based on microarray, tiling array, and RNA-seq studies\; is

						affected by thirteen chemicals including Ethanol, methylmercury hydroxide, and 1-methylnicotinamide based on RNA-seq and microarray studies\; is predicted to encode a protein with the following domains: MAM domain and Concanavalin A-like lectin\/glucanase domain superfamily.
286	WBGene00008154	C47E12.11	Unassigned	Unassigned	Unassigned: regulated by multiple stresses	C47E12.11 is enriched in the germ line and the male based on RNA-seq studies\; is affected by several genes including hsf-1, eat-2, and sir-2.1 based on microarray and RNA-seq studies\; is affected by eleven chemicals including Ethanol, methylmercuric chloride, and manganese chloride based on RNA-seq and microarray studies.
287	WBGene00008215	C49F8.3	Unassigned	Unassigned	Unassigned: regulated by multiple stresses	C49F8.3 is enriched in the PLM, the germline precursor cell, and the hypodermis based on tiling array and RNA-seq studies\; is affected by several genes including daf-16, age-1, and daf-12 based on microarray, RNA-seq, proteomic, and tiling array studies\; is affected by thirteen chemicals including

						methylmercury hydroxide, 1-methylnicotinamide, and Rotenone based on RNA-seq and microarray studies\; is predicted to encode a protein with the following domain: Domain of unknown function DB.
288	WBGene00008221	C50B6.8	Transcription factor	Transcription factor: NHR	Transcription factor: NHR	nhr-168 is an ortholog of human HNF4A (hepatocyte nuclear factor 4 alpha) and HNF4G (hepatocyte nuclear factor 4 gamma)\; is predicted to have several functions, including regulation of cellular process, sequence-specific DNA binding activity, and zinc ion binding activity\; is expressed in the H cell, the V cell, the seam cell, and the tail precursor cell.
289	WBGene00008239	C50H2.1	Neuronal function	Neuronal function: synaptic function	Neuronal function: synaptic function: neurotransmitter receptor	fshr-1 is an ortholog of human FSHR (follicle stimulating hormone receptor), LHCGR (luteinizing hormone\/choriogonadotropin receptor), and TSHR (thyroid stimulating hormone receptor)\; is predicted to have G protein-coupled receptor activity and protein-hormone receptor activity\; is expressed in several tissues, including

						the alimentary system, the nervous system, and the reproductive system\; human orthologs of this gene are implicated in cryptorchidism.
290	WBGene00008254	C51E3.9	Unassigned	Unassigned	Unassigned	C51E3.9 is enriched in the body wall musculature and the nervous system based on tiling array and microarray studies\; is affected by several genes including daf-2, let-7, and gld-1 based on microarray, RNA-seq, and proteomic studies\; is affected by D-glucose and Chlorpyrifos based on RNA-seq and microarray studies\; is predicted to encode a protein with the following domain: S-adenosyl-L-methionine-dependent methyltransferase.
291	WBGene00008258	C52E4.5	Trafficking	Trafficking: ER/Golgi	Trafficking: ER/Golgi: mannosidase	mans-2 is an ortholog of human MAN1A2 (mannosidase alpha class 1A member 2)\; is predicted to have calcium ion binding activity and mannosyl-oligosaccharide 1,2-alpha-mannosidase activity.
292	WBGene00008260	C52G5.2	Unassigned	Unassigned	Unassigned	C52G5.2 is an ortholog of human THAP4 (THAP domain containing 4)\; human THAP4 exhibits

						heme binding activity and protein homodimerization activity.
293	WBGene00008273	C53B4.3	Transmembrane transport	Transmembrane transport: solute carrier	Transmembrane transport: solute carrier	C53B4.3 is an ortholog of human SLC22A18 (solute carrier family 22 member 18)\; is predicted to have transporter activity\; localizes to the nucleus\; human orthologs of this gene are implicated in breast cancer, embryonal rhabdomyosarcoma, and lung cancer.
294	WBGene00008284	C53D6.7	Extracellular material	Extracellular material: galectin	Extracellular material: galectin	C53D6.7 is an ortholog of human LGALS2 (galectin 2), LGALS1, and LGALS14 (galectin 14)\; is predicted to have carbohydrate binding activity.
295	WBGene00008296	C54D10.1	Stress response	Stress response: heavy metal	Stress response: heavy metal	cdr-2 is an ortholog of human FAXC\; is involved in regulation of protein deneddylation and stress response to cadmium ion\; is expressed in several tissues, including the alimentary system, the nervous system, and the reproductive system.
296	WBGene00008304	C54D10.10	Proteolysis general	Proteolysis general: serine	Proteolysis general: serine	C54D10.10 is an ortholog of human TFPI and TFPI2\; is predicted to have serine-type endopeptidase inhibitor activity\; is expressed in

						several tissues, including the nervous system, the pharynx, the reproductive system, and the seam cell.
297	WBGene00008324	C55A1.7	Unassigned	Unassigned	Unassigned	C55A1.7 is affected by several genes including daf-16, eat-2, and sir-2.1 based on RNA-seq, microarray, and tiling array studies\; is predicted to encode a protein with the following domain: Protein of unknown function DUF4473.
298	WBGene00008333	C55A6.4	Metabolism	Metabolism: short chain dehydrogenase	Metabolism: short chain dehydrogenase	C55A6.4 is enriched in the hypodermis, the intestine, and the male based on tiling array, RNA-seq, and microarray studies\; is affected by several genes including daf-16, daf-2, and age-1 based on microarray, RNA-seq, and proteomic studies\; is affected by nine chemicals including Tunicamycin, bisphenol A, and juglone based on microarray and RNA-seq studies\; is predicted to encode a protein with the following domains: Short-chain dehydrogenase\/reductase SDR and NAD(P)-binding domain superfamily.
299	WBGene0000833	C55A6.5	Unassigned	Unassigned	Unassigned:	sdz-8 is enriched in the body wall muscle cell based

	4				regulated by multiple stresses	on tiling array studies\; is affected by several genes including daf-16, daf-2, and age-1 based on microarray, RNA-seq, and proteomic studies\; is affected by twenty-four chemicals including tryptophan, 1-methylnicotinamide, and methylmercuric chloride based on microarray and RNA-seq studies\; is predicted to encode a protein with the following domains: Short-chain dehydrogenase\/reductase SDR and NAD(P)-binding domain superfamily.
300	WBGene00008336	C55A6.7	Metabolism	Metabolism: short chain dehydrogenase	Metabolism: short chain dehydrogenase	C55A6.7 is involved in defense response to Gram-negative bacterium and innate immune response.
301	WBGene00008352	CTEL54X.1	Proteolysis proteasome	Proteolysis proteasome: E3	Proteolysis proteasome: E3: F box	fbxa-6 is enriched in dopaminergic neurons, the OLL, the PVD, and the intestine based on tiling array and RNA-seq studies\; is affected by several genes including daf-16, daf-2, and dpy-10 based on microarray, tiling array, and RNA-seq studies\; is affected by six chemicals including levamisole, stavudine, and Cry5B based on microarray and

302						RNA-seq studies\; is predicted to encode a protein with the following domains: F-box domain and Domain of unknown function DUF38, Caenorhabditis species.
302	WBGene00008358	D1025.7	Unassigned	Unassigned	Unassigned	nspc-18 is affected by several genes including daf-16, daf-2, and glp-1 based on microarray, tiling array, and RNA-seq studies\; is affected by six chemicals including hydrogen sulfide, Mianserin, and D-glucose based on microarray and RNA-seq studies.
303	WBGene00008375	D1054.8	Metabolism	Metabolism: lipid	Metabolism: lipid: sterol	D1054.8 is an ortholog of human HSD17B14\; is predicted to encode a protein with the following domains: Short-chain dehydrogenase\/reductase SDR and NAD(P)-binding domain superfamily.
304	WBGene00008390	D1086.3	Unassigned	Unassigned	Unassigned: regulated by multiple stresses	D1086.3 is enriched in the Z1.p, the Z4.a, the epithelial system, and the male distal tip cell based on tiling array, RNA-seq, and microarray studies\; is affected by several genes including daf-16, daf-2, and glp-1 based on microarray, tiling array, RNA-seq, and proteomic studies\; is affected by nineteen

						chemicals including Heme, methylmercuric chloride, and Tunicamycin based on microarray and RNA-seq studies\; is predicted to encode a protein with the following domains: Domain of unknown function DUF19 and Uncharacterised protein family UPF0376.
305	WBGene00008436	DH11.2	Unassigned	Unassigned	Unassigned: regulated by multiple stresses	DH11.2 is enriched in the AFD, the ASER, the PLM, the intestine, and the muscle cell based on RNA-seq and microarray studies\; is affected by several genes including daf-16, daf-2, and daf-12 based on microarray, tiling array, and RNA-seq studies\; is affected by sixteen chemicals including 1-methylnicotinamide, Rotenone, and D-glucopyranose based on RNA-seq and microarray studies.
306	WBGene00008448	E01G4.6	Unassigned	Unassigned	Unassigned: regulated by multiple stresses	E01G4.6 is enriched in the PLM and the amphid sheath cell based on RNA-seq studies\; is affected by several genes including daf-16, daf-2, and glp-1 based on proteomic, microarray, and RNA-seq studies\; is affected by fourteen chemicals including

						Heme, hydrogen sulfide, and methylmercury hydroxide based on microarray and RNA-seq studies.
307	WBGene00008449	E01G6.1	Proteolysis general	Proteolysis general: inhibitor	Proteolysis general: inhibitor: serine	E01G6.1 is predicted to have chitin binding activity and serine-type endopeptidase inhibitor activity.
308	WBGene00008482	E04D5.3	Extracellular material	Extracellular material: cuticlin	Extracellular material: cuticlin	cut-4 is affected by several genes including daf-16, daf-2, and daf-12 based on tiling array, RNA-seq, and microarray studies\; is affected by eleven chemicals including aldicarb, hydrogen sulfide, and Rotenone based on microarray and RNA-seq studies\; is predicted to encode a protein with the following domain: Zona pellucida domain.
309	WBGene00008496	F01D5.6	Unassigned	Unassigned	Unassigned: regulated by multiple stresses	F01D5.6 is enriched in the germline precursor cell and the hypodermis based on tiling array studies\; is affected by several genes including daf-16, glp-1, and skn-1 based on microarray and RNA-seq studies\; is affected by fourteen chemicals including Ethanol, methylmercury hydroxide, and 1-methylnicotinamide based on RNA-seq and

						microarray studies\; is predicted to encode a protein with the following domain: Modulator of levamisole receptor-1.
310	WBGene00008512	F01G10.10	Metabolism	Metabolism: lipid	Metabolism: lipid: binding	F01G10.10 is an ortholog of human PLTP (phospholipid transfer protein), BPI (bactericidal permeability increasing protein), and LBP (lipopolysaccharide binding protein)\; is predicted to have lipid binding activity.
311	WBGene00008541	F07A5.2	Unassigned	Unassigned	Unassigned	F07A5.2 is enriched in the germ line and the male based on proteomic and RNA-seq studies\; is affected by several genes including daf-2, glp-1, and age-1 based on microarray, RNA-seq, and proteomic studies\; is affected by twenty-three chemicals including Ethanol, methylmercury hydroxide, and nicotinic acid based on RNA-seq and microarray studies.
312	WBGene00008559	F07H5.8	Unassigned	Unassigned	Unassigned: regulated by multiple stresses	F07H5.8 is enriched in GABAergic neurons and the DA neuron based on microarray studies\; is affected by several genes including daf-16, skn-1, and elt-2 based on tiling array, microarray, and

						RNA-seq studies\; is affected by twelve chemicals including methylmercury hydroxide, Rotenone, and sodium arsenite based on RNA-seq and microarray studies\; is predicted to encode a protein with the following domain: Cysteine rich repeat, tripleX.
313	WBGene00008560	F07H5.9	Lysosome	Lysosome: acid phosphatase	Lysosome: acid phosphatase	pho-13 is an ortholog of human ACP2 (acid phosphatase 2, lysosomal), ACP4 (acid phosphatase 4), and ACPP (acid phosphatase, prostate)\; human ACPP exhibits phosphatase activity and protein homodimerization activity.
314	WBGene00008596	F08H9.8	Stress response	Stress response: C-type Lectin	Stress response: C-type Lectin	clec-54 is an ortholog of human CDCP2\; is predicted to have carbohydrate binding activity.
315	WBGene00008597	F08H9.9	Stress response	Stress response: C-type Lectin	Stress response: C-type Lectin	clec-55 is an ortholog of human CDCP2\; is predicted to have carbohydrate binding activity.
316	WBGene00008621	F09C8.1	Signaling	Signaling: lipid	Signaling: lipid: phospholipase B	F09C8.1 is an ortholog of human PLB1 (phospholipase B1)\; is predicted to have hydrolase activity, acting on ester bonds\; is expressed in the intestine.
317	WBGene00008634	F10A3.4	Unassigned	Unassigned	Unassigned	F10A3.4 is expressed in tail neurons and the intestine\; is predicted to encode a protein with the

						following domain: CUB-like domain.
318	WBGene00008651	F10D11.5	Unassigned	Unassigned	Unassigned: regulated by multiple stresses	F10D11.5 is enriched in the NSM, the germ line, and the male based on RNA-seq and tiling array studies\; is affected by several genes including clk-1, eat-2, and npr-1 based on microarray, tiling array, and RNA-seq studies\; is affected by six chemicals including nicotinic acid, manganese chloride, and Hydrolyzable Tannins based on RNA-seq and microarray studies\; is predicted to encode a protein with the following domain: PDZ superfamily.
319	WBGene00008652	F10D11.6	Development	Development: general	Development: general	F10D11.6 is an ortholog of human PLTP (phospholipid transfer protein), BPI (bactericidal permeability increasing protein), and LBP (lipopolysaccharide binding protein)\; is predicted to have lipid binding activity.
320	WBGene00008655	F10F2.3	Metabolism	Metabolism: lipid	Metabolism: lipid: lipase	lips-3 is predicted to have hydrolase activity.
321	WBGene00008724	F13A7.1	Unassigned	Unassigned	Unassigned: regulated by multiple stresses	F13A7.1 is enriched in the germ line and the male based on RNA-seq studies\; is affected by several genes including age-1, dpy-10, and eat-2 based on

						microarray and RNA-seq studies\; is affected by sixteen chemicals including Ethanol, methylmercury hydroxide, and nicotinic acid based on RNA-seq and microarray studies\; is predicted to encode a protein with the following domains: Major sperm protein (MSP) domain, PapD-like superfamily, and Immunoglobulin-like fold.
322	WBGene00008741	F13D12.6	Metabolism	Metabolism: carbohydrate	Metabolism: carbohydrate	F13D12.6 is an ortholog of human CTSA (cathepsin A)\; is predicted to have serine-type carboxypeptidase activity\; is expressed in the intestine.
323	WBGene00008810	F14F7.3	Stress response	Stress response: detoxification	Stress response: detoxification: CYP	cyp-13A12 is predicted to have heme binding activity, iron ion binding activity, and oxidoreductase activity\; is expressed in the marginal cell.
324	WBGene00008851	F15B9.8	Unassigned	Unassigned	Unassigned: regulated by multiple stresses	F15B9.8 is enriched in several tissues, including the AFD, the alimentary system, the germline precursor cell, the hypodermis, and the somatic gonad precursor based on tiling array and RNA-seq studies\; is affected by several genes including daf-

						16, daf-2, and daf-12 based on microarray and RNA-seq studies\; is affected by twenty-one chemicals including Ethanol, Nitric Oxide, and methylmercury hydroxide based on RNA-seq and microarray studies\; is predicted to encode a protein with the following domains: Thrombospondin type-1 (TSP1) repeat and Thrombospondin type-1 (TSP1) repeat superfamily.
325	WBGene00008964	F19H8.4	Unassigned	Unassigned	Unassigned: regulated by multiple stresses	mltn-9 is enriched in the OLL, the PVD, and the hypodermis based on microarray, tiling array, and RNA-seq studies\; is affected by several genes including daf-16, daf-2, and daf-12 based on microarray and RNA-seq studies\; is affected by thirteen chemicals including methylmercury hydroxide, Rotenone, and juglone based on RNA-seq and microarray studies\; is predicted to encode a protein with the following domain: Moulting cycle MLT-10-like protein.
326	WBGene00008993	F21A3.3	Extracellular material	Extracellular material: secreted	Extracellular material: secreted	F21A3.3 is an ortholog of human THAP4 (THAP domain containing 4)\; human THAP4 exhibits

				protein	protein	heme binding activity and protein homodimerization activity.
327	WBGene00009028	F21H7.2	Unassigned	Unassigned	Unassigned: regulated by multiple stresses	F21H7.2 is enriched in the coelomocyte, the germ line, the male, and the muscle cell based on tiling array, RNA-seq, and microarray studies\; is affected by several genes including daf-16, daf-2, and daf-12 based on microarray and RNA-seq studies\; is affected by fourteen chemicals including Ethanol, nicotinic acid, and methylmercuric chloride based on RNA-seq and microarray studies.
328	WBGene00009031	F21H7.5	Unassigned	Unassigned	Unassigned: regulated by multiple stresses	F21H7.5 is enriched in the amphid sheath cell, the germ line, the hypodermis, the male, and the muscle cell based on proteomic, RNA-seq, and microarray studies\; is affected by several genes including daf-2, age-1, and let-7 based on microarray, RNA-seq, tiling array, and proteomic studies\; is affected by fourteen chemicals including Ethanol, nicotinic acid, and methylmercuric chloride based on RNA-seq and microarray studies\; is predicted to encode a protein with the

						following domains: Major sperm protein (MSP) domain, PapD-like superfamily, and Immunoglobulin-like fold.
329	WBGene00009043	F22B5.5	Unassigned	Unassigned	Unassigned: regulated by multiple stresses	F22B5.5 is enriched in the FLP, the germ line, and the male based on microarray and RNA-seq studies\; is affected by several genes including daf-2, dpy-10, and eat-2 based on microarray, tiling array, and RNA-seq studies\; is affected by eight chemicals including methylmercuric chloride, D-glucose, and Quercetin based on microarray and RNA-seq studies\; is predicted to encode a protein with the following domain: Domain of unknown function DUF1248.
330	WBGene00009094	F23H12.5	Unassigned	Unassigned	Unassigned	F23H12.5 is an ortholog of human SEMA6D, SEMA4D, and SEMA6A (semaphorin 6A)\; human SEMA4D exhibits identical protein binding activity, semaphorin receptor binding activity, and transmembrane signaling receptor activity.
331	WBGene00009110	F25D1.5	Metabolism	Metabolism: lipid	Metabolism: lipid: sterol	F25D1.5 is an ortholog of human HSD17B14\; is predicted to have nucleotide binding activity.

332	WBGene00009133	F25H8.6	Nucleic acid	Nucleic acid: binding	Nucleic acid: binding: unassigned	bed-3 exhibits RNA polymerase II regulatory region sequence-specific DNA binding activity\; is involved in molting cycle, regulation of cell division, and vulval cell fate specification\; is expressed in the M lineage cell, the hypodermal cell, and the vulval cell.
333	WBGene00009205	F28C6.5	Unassigned	Unassigned	Unassigned	F28C6.5 is enriched in the germline precursor cell, the hypodermis, the muscle cell, and the nervous system based on tiling array and microarray studies\; is affected by several genes including dpy-10, sir-2.1, and pmk-1 based on microarray and RNA-seq studies\; is affected by five chemicals including Heme, estrogen, and Atrazine based on microarray studies.
334	WBGene00009215	F28D1.5	Stress response	Stress response: pathogen	Stress response: pathogen: unassigned	thn-2 is involved in defense response to Gram-negative bacterium and defense response to Gram-positive bacterium.
335	WBGene00009226	F28G4.1	Stress response	Stress response: detoxification	Stress response: detoxification: CYP	cyp-37B1 is an ortholog of human CYP19A1 (cytochrome P450 family 19 subfamily A member 1) and CYP4V2 (cytochrome P450 family 4

						subfamily V member 2)\; is predicted to have heme binding activity, iron ion binding activity, and oxidoreductase activity\; is involved in defense response to Gram-positive bacterium\; is expressed in the intestinal cell.
336	WBGene00009311	F32A11.7	Transmembrane protein	Transmembrane protein: unassigned	Transmembrane protein: unassigned	mltn-1 is enriched in the Z1, the Z4, and the hypodermis based on RNA-seq studies\; is affected by several genes including daf-16, daf-2, and glp-1 based on microarray and RNA-seq studies\; is affected by methylmercury hydroxide, Rotenone, and manganese chloride based on RNA-seq studies\; is predicted to encode a protein with the following domain: Moulting cycle MLT-10-like protein.
337	WBGene00009321	F32B6.4	Unassigned	Unassigned	Unassigned: regulated by multiple stresses	F32B6.4 is enriched in the germ line and the male based on proteomic and RNA-seq studies\; is affected by several genes including age-1, dpy-10, and hsf-1 based on microarray and RNA-seq studies\; is affected by seventeen chemicals including Heme, Ethanol, and methylmercuric

						chloride based on microarray and RNA-seq studies.
338	WBGene00009324	F32B6.10	Cytoskeleton	Cytoskeleton: microtubule	Cytoskeleton: microtubule: tau tubulin kinase	ttbk-3 is an ortholog of human CTD-2116N17.1, TTBK1 (tau tubulin kinase 1), and TTBK2\; is predicted to have ATP binding activity and protein kinase activity\; is expressed in the amphid sensillum, the head, and the nervous system.
339	WBGene00009331	F32D8.7	Proteolysis general	Proteolysis general: inhibitor	Proteolysis general: inhibitor: serine	F32D8.7 is predicted to have serine-type endopeptidase inhibitor activity.
340	WBGene00009334	F32D8.12	Metabolism	Metabolism: glycolysis	Metabolism: glycolysis	F32D8.12 is an ortholog of human LDHD\; is predicted to have flavin adenine dinucleotide binding activity and oxidoreductase activity.
341	WBGene00009356	F33A8.10	Unassigned	Unassigned	Unassigned	F33A8.10 is enriched in the ASER based on RNA-seq studies\; is affected by several genes including daf-12, dpy-21, and smg-2 based on tiling array, RNA-seq, and microarray studies\; is affected by four chemicals including Rotenone, multi-walled carbon nanotube, and D-glucose based on RNA-seq and microarray studies\; is predicted to encode a protein with the following domain: Domain of

						unknown function DUF148.
342	WBGene00009358	F33C8.4	Unassigned	Unassigned	Unassigned: regulated by multiple stresses	F33C8.4 is enriched in GABAergic neurons, the ASER, the PLM, and the muscle cell based on tiling array, RNA-seq, proteomic, and microarray studies\; is affected by several genes including daf-2, let-7, and dpy-10 based on proteomic, RNA-seq, and microarray studies\; is affected by six chemicals including methylmercuric chloride, Rotenone, and D-glucopyranose based on microarray and RNA-seq studies.
343	WBGene00009430	F35E12.6	Stress response	Stress response: pathogen	Stress response: pathogen: unassigned	F35E12.6 is involved in innate immune response.
344	WBGene00009471	F36D3.5	Transmembrane protein	Transmembrane protein: unassigned	Transmembrane protein: unassigned	F36D3.5 is enriched in the germ line and the male based on RNA-seq studies\; is affected by several genes including eat-2, sir-2.1, and npr-1 based on microarray, RNA-seq, and tiling array studies\; is affected by eleven chemicals including hydrogen sulfide, methylmercuric chloride, and Rotenone based on microarray and RNA-seq studies\; is

						predicted to encode a protein with the following domains: BRCT domain, Ankyrin repeat, Domain of unknown function WSN, BRCT domain superfamily, and Ankyrin repeat-containing domain superfamily.
345	WBGene00009515	F38A1.1	Stress response	Stress response: C-type Lectin	Stress response: C-type Lectin	clec-170 is predicted to have carbohydrate binding activity.
346	WBGene00009528	F38A6.4	Unassigned	Unassigned	Unassigned: regulated by multiple stresses	F38A6.4 is enriched in the AFD, the PLM, the germline precursor cell, and the hypodermis based on tiling array and RNA-seq studies\; is affected by several genes including daf-16, daf-12, and clk-1 based on microarray and RNA-seq studies\; is affected by four chemicals including D-glucose, stavudine, and Zidovudine based on RNA-seq and microarray studies.
347	WBGene00009531	F38B2.4	Metabolism	Metabolism: nucleotide	Metabolism: nucleotide	F38B2.4 is an ortholog of human AK1 (adenylate kinase 1)\; is predicted to have ATP binding activity and adenylate kinase activity.
348	WBGene00009573	F40E10.5	Unassigned	Unassigned	Unassigned: regulated by	F40E10.5 is enriched in the alimentary system, the digestive tract, and the nervous system based on

					multiple stresses	microarray, tiling array, and proteomic studies\; is affected by several genes including daf-16, skn-1, and daf-12 based on microarray, tiling array, and RNA-seq studies\; is affected by thirteen chemicals including 1-methylnicotinamide, Rotenone, and multi-walled carbon nanotube based on RNA-seq and microarray studies.
349	WBGene00009582	F40F9.5	Transmembrane transport	Transmembrane transport: major facilitator	Transmembrane transport: major facilitator	F40F9.5 is an ortholog of human SLC46A1, SLC46A2 (solute carrier family 46 member 2), and SLC46A3 (solute carrier family 46 member 3)\; is predicted to encode a protein with the following domains: Major facilitator superfamily and MFS transporter superfamily.
350	WBGene00009590	F40F12.1	Pseudogene	Pseudogene	Pseudogene	ttr-4 is affected by several genes including daf-16, daf-2, and dpy-10 based on microarray and RNA-seq studies\; is affected by six chemicals including Cry5B, Chlorpyrifos, and Diazinon based on microarray studies.
351	WBGene00009621	F41E7.5	Unassigned	Unassigned	Unassigned: regulated by	fipr-21 is enriched in the germline precursor cell and the intestine based on tiling array and

					multiple stresses	microarray studies\; is affected by several genes including daf-16, daf-2, and age-1 based on microarray, RNA-seq, proteomic, and tiling array studies\; is affected by twelve chemicals including nicotinic acid, Rotenone, and D-glucose based on RNA-seq and microarray studies.
352	WBGene00009634	F42F12.1	Unassigned	Unassigned	Unassigned	nspc-9 is enriched in the intestine based on microarray studies\; is affected by several genes including daf-16, daf-2, and glp-1 based on microarray and RNA-seq studies\; is affected by six chemicals including Rotenone, Mianserin, and D-glucose based on RNA-seq and microarray studies.
353	WBGene00009645	F42G10.1	Proteolysis general	Proteolysis general: metallopeptidase	Proteolysis general: metallopeptidase	F42G10.1 is predicted to have metallopeptidase activity.
354	WBGene00009677	F44A6.5	Unassigned	Unassigned	Unassigned	F44A6.5 is enriched in the ASER, the PLM, the germline precursor cell, and the intestine based on tiling array and RNA-seq studies\; is affected by several genes including daf-16, daf-2, and age-1 based on microarray and RNA-seq studies\; is

						affected by thirteen chemicals including Ethanol, Tunicamycin, and bisphenol S based on RNA-seq and microarray studies.
355	WBGene00009682	F44D12.5	Unassigned	Unassigned	Unassigned	msd-2 localizes to the cell body and pseudopodium.
356	WBGene00009686	F44D12.9	Transmembrane transport	Transmembrane transport: solute carrier	Transmembrane transport: solute carrier	ent-7 is an ortholog of human SLC29A1 (solute carrier family 29 member 1 (Augustine blood group)), SLC29A2 (solute carrier family 29 member 2), and SLC29A3 (solute carrier family 29 member 3)\; is predicted to have nucleoside transmembrane transporter activity.
357	WBGene00009754	F46A9.1	Unassigned	Unassigned	Unassigned: regulated by multiple stresses	F46A9.1 is enriched in the germ line and the male based on RNA-seq studies\; is affected by several genes including daf-2, dpy-10, and hsf-1 based on microarray and RNA-seq studies\; is affected by thirteen chemicals including nicotinic acid, Rotenone, and manganese chloride based on RNA-seq and microarray studies.
358	WBGene00009773	F46B6.8	Metabolism	Metabolism: lipid	Metabolism: lipid: lipase	lipl-2 is an ortholog of human LIPJ (lipase J), LIPK (lipase K), and LIPF (lipase F, gastric type)\; is

						predicted to have hydrolase activity, acting on ester bonds\; human orthologs of this gene are implicated in autosomal recessive congenital ichthyosis 8 and lysosomal and lipase deficiency.
359	WBGene00009791	F46F3.3	Unassigned	Unassigned	Unassigned	F46F3.3 is enriched in the germline precursor cell based on tiling array studies\; is affected by several genes including daf-16, daf-2, and hsf-1 based on microarray, RNA-seq, and tiling array studies\; is affected by eight chemicals including methylmercury hydroxide, Alovudine, and stavudine based on RNA-seq and microarray studies.
360	WBGene00009815	F47B10.4	Unassigned	Unassigned	Unassigned	F47B10.4 is affected by hpl-2, rsr-2, and eat-2 based on tiling array, RNA-seq, and microarray studies\; is affected by Atrazine and allantoin based on microarray studies.
361	WBGene00009830	F47G9.3	Extracellular material	Extracellular material: cuticlin	Extracellular material: cuticlin	cutl-18 is enriched in the intestine based on RNA-seq studies\; is affected by several genes including daf-16, daf-2, and daf-12 based on tiling array, RNA-seq, and microarray studies\; is affected by

						nine chemicals including Heme, methylmercuric chloride, and stavudine based on microarray and RNA-seq studies\; is predicted to encode a protein with the following domains: Zona pellucida domain and PAN\/Apple domain.
362	WBGene00009877	F49C12.7	Signaling	Signaling: unassigned	Signaling: unassigned	F49C12.7 is enriched in the coelomocyte, the germ line, the intestine, and the nervous system based on proteomic, tiling array, RNA-seq, and microarray studies\; is affected by several genes including daf-16, daf-2, and glp-1 based on microarray, RNA-seq, and proteomic studies\; is affected by twelve chemicals including Ethanol, 1-methylnicotinamide, and methylmercuric chloride based on RNA-seq and microarray studies\; is predicted to encode a protein with the following domains: Protein kinase-like domain superfamily, Uncharacterised oxidoreductase Dhs-27, and CHK kinase-like.
363	WBGene00009889	F49E11.2	Protein modification	Protein modification:	Protein modification:	F49E11.2 is enriched in the NSM and the hypodermis based on tiling array and RNA-seq

				methyltransferase	methyltransferase	studies\; is affected by several genes including daf-16, skn-1, and daf-12 based on tiling array, microarray, and RNA-seq studies\; is affected by six chemicals including 1-methylnicotinamide, manganese chloride, and Colistin based on RNA-seq and microarray studies.
364	WBGene00009943	F52G2.3	Unassigned	Unassigned	Unassigned: regulated by multiple stresses	F52G2.3 is enriched in the OLL, the PVD, the body wall muscle cell, and the intestine based on microarray, tiling array, and RNA-seq studies\; is affected by several genes including glp-1, dpy-10, and clk-1 based on microarray and RNA-seq studies\; is affected by five chemicals including Alovudine, stavudine, and Cry5B based on RNA-seq and microarray studies\; is predicted to encode a protein with the following domain: RSD-2, N-terminal.
365	WBGene00009948	F52H3.6	Signaling	Signaling: phosphatase	Signaling: phosphatase: unassigned	F52H3.6 is an ortholog of human PPP1CB (protein phosphatase 1 catalytic subunit beta)\; is predicted to have phosphoprotein phosphatase activity.
366	WBGene0000995	F53B6.4	Unassigned	Unassigned	Unassigned:	F53B6.4 is enriched in the germ line and the male

	9				membrane spanning domain	based on proteomic and RNA-seq studies\; is affected by several genes including daf-2, age-1, and let-7 based on microarray, RNA-seq, and proteomic studies\; is affected by seventeen chemicals including Ethanol, nicotinic acid, and methylmercuric chloride based on RNA-seq and microarray studies\; is predicted to encode a protein with the following domains: Major sperm protein (MSP) domain, PapD-like superfamily, and Immunoglobulin-like fold.
367	WBGene00009982	F53F1.4	Unassigned	Unassigned	Unassigned: regulated by multiple stresses	F53F1.4 is enriched in the AFD, the ASER, the germline precursor cell, and the hypodermis based on tiling array and RNA-seq studies\; is affected by several genes including daf-16, daf-2, and glp-1 based on microarray, RNA-seq, and tiling array studies\; is affected by seventeen chemicals including aldicarb, 1-methylnicotinamide, and nicotinic acid based on microarray and RNA-seq studies.
368	WBGene0001003	F54B11.11	Stress response	Stress response:	Stress response:	F54B11.11 is enriched in the intestine based on

	4			pathogen	pathogen: unassigned	RNA-seq studies\; is affected by several genes including daf-2, hsf-1, and clk-1 based on tiling array, microarray, and RNA-seq studies\; is affected by seven chemicals including bisphenol A, Zidovudine, and Chlorpyrifos based on RNA-seq and microarray studies\; is predicted to encode a protein with the following domain: CUB-like domain.
369	WBGene00010062	F54F3.3	Metabolism	Metabolism: lipid	Metabolism: lipid: lipase	lipl-1 is an ortholog of human LIPJ (lipase J), LIPK (lipase K), and LIPF (lipase F, gastric type)\; is predicted to have hydrolase activity, acting on ester bonds\; localizes to the lysosome\; is expressed in the intestine\; human orthologs of this gene are implicated in autosomal recessive congenital ichthyosis 8 and lysosomal and lipase deficiency.
370	WBGene00010116	F55F3.2	Metabolism	Metabolism: unassigned	Metabolism: unassigned	F55F3.2 is an ortholog of human CES2 (carboxylesterase 2), Tg, and CEL (carboxyl ester lipase) and members of the human NRLGN (Neuroligins) gene family including NLGN4X, NLGN1, and NLGN4Y\; is predicted to have

						hydrolase activity\; human orthologs of this gene are implicated in maturity-onset diabetes of the young type 8.
371	WBGene00010117	F55F3.3	Transmembrane transport	Transmembrane transport: unassigned	Transmembrane transport: unassigned	nkb-3 is an ortholog of human ATP1B2 (ATPase Na+\/K+ transporting subunit beta 2), ATP1B3, and ATP1B1 (ATPase Na+\/K+ transporting subunit beta 1)\; human orthologs of this gene are implicated in essential hypertension\; human ATP1B1 exhibits ATPase activator activity and ATPase binding activity.
372	WBGene00010135	F55H12.4	Unassigned	Unassigned	Unassigned	F55H12.4 is expressed in anus, the hypodermis, the pharynx, and the vulva.
373	WBGene00010157	F56G4.1	Metabolism	Metabolism: lipid	Metabolism: lipid: o-acyl transferase	oac-34 is predicted to have transferase activity, transferring acyl groups other than amino-acyl groups.
374	WBGene00010267	F58G1.5	Metabolism	Metabolism: lipid	Metabolism: lipid: lipase	lips-9 is predicted to have hydrolase activity.
375	WBGene00010277	F58G6.7	Transmembrane transport	Transmembrane transport: solute carrier	Transmembrane transport: solute carrier	F58G6.7 is an ortholog of human SLC31A1 (solute carrier family 31 member 1) and SLC31A2 (solute carrier family 31 member 2)\; is predicted to have

						copper ion transmembrane transporter activity.
376	WBGene00010321	F59B10.5	Unassigned	Unassigned	Unassigned	F59B10.5 is enriched in the NSM, the coelomocyte, and the hypodermis based on tiling array and RNA-seq studies\; is affected by several genes including daf-16, daf-2, and daf-12 based on microarray, tiling array, and RNA-seq studies\; is affected by nine chemicals including Heme, D-glucose, and Atrazine based on microarray and RNA-seq studies.
377	WBGene00010336	F59F4.1	Metabolism	Metabolism: lipid	Metabolism: lipid: beta oxidation	acox-1.6 is an ortholog of human ACOX1\; is predicted to have FAD binding activity and acyl-CoA oxidase activity\; is expressed in the intestine and the pharynx\; human orthologs of this gene are implicated in peroxisomal acyl-CoA oxidase deficiency.
378	WBGene00010366	H05L14.1	Cytoskeleton	Cytoskeleton: microtubule	Cytoskeleton: microtubule: tau tubulin kinase	H05L14.1 is an ortholog of human CTD-2116N17.1, TTBK1 (tau tubulin kinase 1), and TTBK2\; is predicted to have ATP binding activity and protein kinase activity.
379	WBGene0001036	H06A10.1	Unassigned	Unassigned	Unassigned:	H06A10.1 is an ortholog of human THBD

	8				regulated by multiple stresses	(thrombomodulin)\; is involved in nematode larval development.
380	WBGene00010424	H36L18.2	Non-coding RNA	Non-coding RNA: linc	Non-coding RNA: linc	lep-5 is enriched in several tissues, including the Psub1, the Z1.p, the Z4.a, the male distal tip cell, and the somatic gonad precursor based on RNA-seq studies\; is affected by several genes including daf-16, glp-1, and age-1 based on microarray, tiling array, and RNA-seq studies\; is affected by Rotenone, D-glucose, and Zidovudine based on RNA-seq studies.
381	WBGene00010456	K01C8.1	Metabolism	Metabolism: amino acid	Metabolism: amino acid: breakdown	K01C8.1 is predicted to have L-threonine ammonia-lyase activity.
382	WBGene00010471	K01D12.12	Transmembrane protein	Transmembrane protein: unassigned	Transmembrane protein: unassigned	cdr-6 is an ortholog of human FAXC\; is expressed in the intestine, the pharynx, and the rectal gland cell\; is predicted to encode a protein with the following domains: Thioredoxin-like fold, Metaxin, glutathione S-transferase domain, Thioredoxin-like superfamily, and Glutathione S-transferase, C-terminal domain superfamily.

383	WBGene00010485	K01H12.2	Transmembrane transport	Transmembrane transport: mitochondrial	Transmembrane transport: mitochondrial: solute carrier	ant-1.3 is an ortholog of human SLC25A4 (solute carrier family 25 member 4), SLC25A5 (solute carrier family 25 member 5), and SLC25A6 (solute carrier family 25 member 6)\; is predicted to have adenine nucleotide transmembrane transporter activity\; is expressed in the VA11 and the intestine.
384	WBGene00010486	K01H12.4	Unassigned	Unassigned	Unassigned: regulated by multiple stresses	K01H12.4 is enriched in the germ line and the male based on proteomic and RNA-seq studies\; is affected by several genes including glp-1, age-1, and daf-12 based on microarray, tiling array, RNA-seq, and proteomic studies\; is affected by fourteen chemicals including Ethanol, nicotinic acid, and methylmercuric chloride based on RNA-seq and microarray studies.
385	WBGene00010510	K02E11.1	Transmembrane transport	Transmembrane transport: solute carrier	Transmembrane transport: solute carrier	ent-3 is an ortholog of human SLC29A1 (solute carrier family 29 member 1 (Augustine blood group)), SLC29A2 (solute carrier family 29 member 2), and SLC29A3 (solute carrier family 29 member 3)\; is predicted to have nucleoside transmembrane transporter activity.

386	WBGene00010574	K04H4.5	Unassigned	Unassigned	Unassigned: regulated by multiple stresses	K04H4.5 is enriched in GABAergic neurons, the germ line, the male, the muscle cell, and the somatic gonad precursor based on RNA-seq and microarray studies\; is affected by several genes including daf-2, age-1, and lin-4 based on microarray and RNA-seq studies\; is affected by fourteen chemicals including Ethanol, nicotinic acid, and methylmercuric chloride based on RNA-seq and microarray studies.
387	WBGene00010593	K06A4.3	Cytoskeleton	Cytoskeleton: unassigned	Cytoskeleton: unassigned	gsnl-1 is an ortholog of human CAPG (capping actin protein, gelsolin like), VILL (villin like), and VIL1 (villin 1)\; exhibits actin filament binding activity, actin monomer binding activity, and phosphatidylinositol-4,5-bisphosphate binding activity\; is involved in several processes, including actin filament depolymerization, actin filament severing, and barbed-end actin filament capping\; localizes to the actin cytoskeleton and striated muscle dense body\; is expressed in the hypodermis, the muscular system, and the

						reproductive system.
388	WBGene00010651	K08C9.2	Unassigned	Unassigned	Unassigned	K08C9.2 is expressed in the spermatheca.
389	WBGene00010657	K08D8.3	Stress response	Stress response: pathogen	Stress response: pathogen: CUB	K08D8.3 is enriched in the ASER and the intestine based on RNA-seq and microarray studies\; is affected by several genes including daf-16, daf-2, and hsf-1 based on microarray and RNA-seq studies\; is affected by eight chemicals including Rotenone, Tunicamycin, and D-glucose based on RNA-seq and microarray studies\; is predicted to encode a protein with the following domain: CUB-like domain.
390	WBGene00010661	K08E3.1	Neuronal function	Neuronal function: synaptic function	Neuronal function: synaptic function: neurotransmitter metabolism	tyr-2 is an ortholog of human DCT, TYR, and TYRP1\; exhibits dopachrome isomerase activity\; is involved in cellular metabolic process\; is expressed in the hypodermis and the uterine muscle.
391	WBGene00010684	K08F9.1	Transmembrane transport	Transmembrane transport: solute	Transmembrane transport: solute	K08F9.1 is an ortholog of human SLC2A7 (solute carrier family 2 member 7), SLC2A9 (solute carrier

				carrier	carrier	family 2 member 9), and SLC2A5 (solute carrier family 2 member 5)\; is predicted to have transmembrane transporter activity.
392	WBGene00010714	K09B11.10	Unassigned	Unassigned	Unassigned: regulated by multiple stresses	mam-3 is enriched in the germline precursor cell and the hypodermis based on tiling array studies\; is affected by several genes including daf-16, daf-12, and dpy-10 based on microarray and RNA-seq studies\; is affected by eleven chemicals including Ethanol, Nitric Oxide, and methylmercury hydroxide based on RNA-seq and microarray studies\; is predicted to encode a protein with the following domain: Concanavalin A-like lectin\/glucanase domain superfamily.
393	WBGene00010723	K09E4.6	Extracellular material	Extracellular material: secreted protein	Extracellular material: secreted protein	cpg-7 is enriched in the hypodermis based on tiling array studies\; is affected by several genes including daf-16, daf-2, and glp-1 based on RNA-seq and microarray studies\; is affected by D-glucose, Cadmium Chloride, and Sodium Chloride based on RNA-seq studies.
394	WBGene0001072	K09G1.1	Transmembran	Transmembrane	Transmembrane	K09G1.1 is an ortholog of human MARVELD1

	7		e protein	protein: unassigned	protein: unassigned	(MARVEL domain containing 1), MAL2 (mal, T cell differentiation protein 2 (gene\/pseudogene)), and MAL (mal, T cell differentiation protein) and members of the human CMTM (CKLF like MARVEL transmembrane domain containing) gene family including CMTM4, CMTM6, and CMTM8.
395	WBGene0001076 0	K10H10.4	Unassigned	Unassigned	Unassigned: regulated by multiple stresses	K10H10.4 is enriched in the ASER, the hypodermis, and the pharyngeal muscle cell based on tiling array and RNA-seq studies\; is affected by several genes including daf-16, skn-1, and daf-12 based on microarray, tiling array, and RNA-seq studies\; is affected by fourteen chemicals including 1-methylnicotinamide, nicotinic acid, and Rotenone based on RNA-seq and microarray studies.
396	WBGene0001076 9	K11D2.2	Metabolism	Metabolism: lipid	Metabolism: lipid: sphingolipid	asah-1 is an ortholog of human ASAH1\; is predicted to have N-acylsphingosine amidohydrolase activity and ceramidase activity\; is expressed in the intestine\; human orthologs of this gene are implicated in Farber lipogranulomatosis.
397	WBGene0001079	LLC1.2	Unassigned	Unassigned	Unassigned	LLC1.2 is enriched in the alimentary system, the

	3					digestive tract, and the nervous system based on tiling array, RNA-seq, and microarray studies\; is affected by several genes including daf-2, let-7, and dpy-10 based on microarray, proteomic, and RNA-seq studies\; is affected by seventeen chemicals including methylmercuric chloride, Rotenone, and Mianserin based on microarray and RNA-seq studies.
398	WBGene00010811	M01F1.5	Transmembrane transport	Transmembrane transport: lipid	Transmembrane transport: lipid	hmit-1.3 is an ortholog of human SLC2A13 (solute carrier family 2 member 13)\; is predicted to have transmembrane transporter activity\; localizes to the apical plasma membrane\; is expressed in the head, the intestine, and the nervous system.
399	WBGene00010831	M02G9.2	Unassigned	Unassigned	Unassigned: regulated by multiple stresses	M02G9.2 is enriched in the intestine based on tiling array studies\; is affected by several genes including daf-16, skn-1, and daf-12 based on tiling array, RNA-seq, and microarray studies\; is affected by eleven chemicals including methylmercury hydroxide, methylmercuric chloride, and sodium arsenite based on RNA-seq and microarray

						studies\; is predicted to encode a protein with the following domain: Protein of unknown function DUF1096.
400	WBGene00010900	M28.9	Transmembrane protein	Transmembrane protein: unassigned	Transmembrane protein: unassigned	M28.9 is enriched in the germ line and the male based on RNA-seq studies\; is affected by several genes including daf-2, clk-1, and npr-1 based on microarray, RNA-seq, and tiling array studies\; is affected by ten chemicals including hydrogen sulfide, Ethanol, and nicotinic acid based on microarray and RNA-seq studies\; is predicted to encode a protein with the following domain: Prominin.
401	WBGene00010924	M153.1	Metabolism	Metabolism: amino acid	Metabolism: amino acid: synthesis	M153.1 is an ortholog of human PYCR1 and PYCR2\; is predicted to have pyrroline-5-carboxylate reductase activity\; human orthologs of this gene are implicated in autosomal recessive cutis laxa type IIB, autosomal recessive cutis laxa type IIIB, and hypomyelinating leukodystrophy 10.
402	WBGene00010943	M176.4	Transmembrane protein	Transmembrane protein: ER	Transmembrane protein: ER	M176.4 is an ortholog of human TMEM86A and TMEM86B\; localizes to the endoplasmic

						reticulum\; is expressed in the arcade cell and the intestine.
403	WBGene00010957	MTCE.3	Metabolism	Metabolism: mitochondria	Metabolism: mitochondria: complex I	nduo-6 is predicted to have NADH dehydrogenase (ubiquinone) activity.
404	WBGene00011095	R07B7.11	Metabolism	Metabolism: carbohydrate	Metabolism: carbohydrate	gana-1 is an ortholog of human NAGA (alpha-N-acetylgalactosaminidase)\; is predicted to have hydrolase activity, hydrolyzing O-glycosyl compounds\; is involved in glycoside catabolic process\; localizes to the cytoplasm\; is expressed in the alimentary system, the body wall musculature, the coelomocyte, the digestive tract, and the epithelial system\; is used to study Fabry disease.
405	WBGene00011112	R07E5.4	Metabolism	Metabolism: unassigned	Metabolism: unassigned	R07E5.4 is an ortholog of human IFI30\; is expressed in the excretory cell, the hypodermis, and the pharynx\; human IFI30 exhibits oxidoreductase activity, acting on a sulfur group of donors.
406	WBGene00011135	R08A2.4	Proteolysis proteasome	Proteolysis proteasome: E3	Proteolysis proteasome: E3:	fbxa-147 is enriched in the AFD based on RNA-seq studies\; is affected by several genes including bar-

407					F box	1, hpl-2, and drh-3 based on tiling array, microarray, and RNA-seq studies\; is predicted to encode a protein with the following domains: F-box domain, Domain of unknown function DUF38, Caenorhabditis species, and F-box-like domain superfamily.
407	WBGene00011147	R08D7.7	Metabolism	Metabolism: carbohydrate	Metabolism: carbohydrate	R08D7.7 is an ortholog of human XYLB\; is predicted to have kinase activity and phosphotransferase activity, alcohol group as acceptor.
408	WBGene00011217	R10E11.5	Unassigned	Unassigned	Unassigned	R10E11.5 is enriched in the nervous system based on tiling array, RNA-seq, and microarray studies\; is affected by several genes including daf-2, daf-12, and sir-2.1 based on microarray, tiling array, and RNA-seq studies\; is affected by Rotenone and Zidovudine based on RNA-seq studies.
409	WBGene00011235	R11A5.7	Proteolysis general	Proteolysis general: carboxypeptidase	Proteolysis general: carboxypeptidase	suro-1 is an ortholog of human CPA4 (carboxypeptidase A4), CPA1 (carboxypeptidase A1), and CPA6 (carboxypeptidase A6)\; is predicted to have metallocarboxypeptidase activity

						and zinc ion binding activity\; localizes to the collagen and cuticulin-based cuticle extracellular matrix and cytoplasm\; is expressed in the hypodermis\; human orthologs of this gene are implicated in familial temporal lobe epilepsy 5.
410	WBGene00011362	T02B5.1	Stress response	Stress response: detoxification	Stress response: detoxification: unassigned	cest-1 is an ortholog of human ACHE (acetylcholinesterase (Cartwright blood group)) and CES2 (carboxylesterase 2)\; is predicted to have hydrolase activity.
411	WBGene00011364	T02B5.3	Metabolism	Metabolism: unassigned	Metabolism: unassigned	T02B5.3 is an ortholog of human ACHE (acetylcholinesterase (Cartwright blood group)) and CES2 (carboxylesterase 2)\; is predicted to have hydrolase activity.
412	WBGene00011379	T02E1.7	Transmembrane protein	Transmembrane protein: unassigned	Transmembrane protein: unassigned	T02E1.7 is enriched in the male based on RNA-seq studies\; is affected by several genes including dpy-10, clk-1, and eat-2 based on microarray and RNA-seq studies\; is affected by seven chemicals including methylmercuric chloride, D-glucose, and Acrylamide based on microarray and RNA-seq studies\; is predicted to encode a protein with the

						following domain: Surfeit locus 4.
413	WBGene00011393	T03D8.6	Metabolism	Metabolism: glutathione	Metabolism: glutathione	T03D8.6 is an ortholog of human GGT1 (gamma-glutamyltransferase 1) and GGT2 (gamma-glutamyltransferase 2)\; is predicted to have glutathione hydrolase activity.
414	WBGene00011397	T03E6.8	Transmembrane protein	Transmembrane protein: unassigned	Transmembrane protein: unassigned	T03E6.8 is enriched in the Z1, the Z4, and the intestine based on RNA-seq studies\; is affected by several genes including daf-16, daf-2, and glp-1 based on RNA-seq and microarray studies\; is affected by seven chemicals including Rotenone, Tunicamycin, and 4-bromodiphenyl ether based on RNA-seq and microarray studies\; is predicted to encode a protein with the following domain: 7TM GPCR, serpentine receptor class h (Srh).
415	WBGene00011422	T04B2.3	Unassigned	Unassigned	Unassigned: regulated by multiple stresses	T04B2.3 is enriched in several tissues, including the muscular system, the nervous system, and the pharynx based on Chronogram, microarray, tiling array, RNA-seq, and proteomic studies\; is affected by several genes including let-60, dpy-10, and sir-2.1 based on RNA-seq and microarray studies\; is

						affected by eleven chemicals including methylmercury hydroxide, Rotenone, and stavudine based on RNA-seq and microarray studies.
416	WBGene00011436	T04F3.1	Unassigned	Unassigned	Unassigned	T04F3.1 is an ortholog of human ACCS and ACCSL\; is predicted to have catalytic activity and pyridoxal phosphate binding activity.
417	WBGene00011460	T05A10.3	Unassigned	Unassigned	Unassigned: regulated by multiple stresses	ttr-14 is enriched in the amphid sheath cell, the germ line, the hypodermis, and the intestine based on proteomic, RNA-seq, and microarray studies\; is affected by several genes including glp-1, dpy-10, and hsf-1 based on microarray, proteomic, tiling array, and RNA-seq studies\; is affected by sixteen chemicals including Ethanol, methylmercury hydroxide, and nicotinic acid based on RNA-seq and microarray studies\; is predicted to encode a protein with the following domains: Transthyretin-like and Transthyretin-like superfamily.
418	WBGene00011493	T05F1.8	Transmembrane transport	Transmembrane transport: mitochondrial	Transmembrane transport: mitochondrial:	T05F1.8 is an ortholog of human SLC25A3 (solute carrier family 25 member 3)\; is predicted to encode a protein with the following domains:

					solute carrier	Mitochondrial substrate\/solute carrier and Mitochondrial carrier domain superfamily.
419	WBGene00011495	T05F1.11	Stress response	Stress response: thioredoxin	Stress response: thioredoxin	T05F1.11 is an ortholog of human NXNL2 (nucleoredoxin like 2)\; is predicted to encode a protein with the following domains: Thioredoxin-like fold and Thioredoxin-like superfamily.
420	WBGene00011516	T06C12.9	Transmembrane protein	Transmembrane protein: unassigned	Transmembrane protein: unassigned	T06C12.9 is enriched in the ASER based on RNA-seq studies\; is affected by several genes including daf-12, sir-2.1, and mir-34 based on tiling array and microarray studies\; is predicted to encode a protein with the following domain: Proteolipid membrane potential modulator.
421	WBGene00011530	T06D8.10	Unassigned	Unassigned	Unassigned	T06D8.10 is predicted to have heme binding activity and peroxidase activity.
422	WBGene00011535	T06E4.8	Stress response	Stress response: ER	Stress response: ER	T06E4.8 is involved in endoplasmic reticulum unfolded protein response.
423	WBGene00011536	T06E4.9	Unassigned	Unassigned	Unassigned: regulated by multiple stresses	T06E4.9 is enriched in the germ line, the intestine, and the pharynx based on microarray, proteomic, and RNA-seq studies\; is affected by several genes including daf-16, skn-1, and daf-12 based on

						microarray, proteomic, tiling array, and RNA-seq studies\; is affected by fourteen chemicals including Ethanol, methylmercury hydroxide, and Rotenone based on RNA-seq and microarray studies.
424	WBGene00011548	T06G6.6	Unassigned	Unassigned	Unassigned: regulated by multiple stresses	T06G6.6 is enriched in the OLL, the PVD, and the hypodermis based on microarray and tiling array studies\; is affected by several genes including daf-16, daf-12, and lin-4 based on microarray and RNA-seq studies\; is affected by eleven chemicals including methylmercury hydroxide, 1-methylnicotinamide, and nicotinic acid based on RNA-seq and microarray studies.
425	WBGene00011556	T07A5.3	Transmembrane transport	Transmembrane transport: solute carrier	Transmembrane transport: solute carrier	vglu-3 is an ortholog of human SLC17A6 (solute carrier family 17 member 6), SLC17A7 (solute carrier family 17 member 7), and SLC17A8 (solute carrier family 17 member 8)\; is predicted to have symporter activity\; human orthologs of this gene are implicated in autosomal dominant nonsyndromic deafness 25.
426	WBGene00011587	T07F10.1	Proteolysis	Proteolysis	Proteolysis	anp-1 is an ortholog of human LNPEP, ERAP1, and

			general	general: unassigned	general: unassigned	ERAP2\; is predicted to have aminopeptidase activity, metallopeptidase activity, and zinc ion binding activity\; is expressed in the excretory cell, the intestine, the nervous system, and the pharynx.
427	WBGene00011590	T07F10.4	Transmembrane protein	Transmembrane protein: unassigned	Transmembrane protein: unassigned	bus-19 is an ortholog of human TMEM41A\; is predicted to encode a protein with the following domain: SNARE associated Golgi protein.
428	WBGene00011617	T08G3.4	Pseudogene	Pseudogene	Pseudogene	T08G3.4 is enriched in the male based on RNA-seq studies\; is affected by several genes including dpy-10, clk-1, and eat-2 based on microarray and RNA-seq studies\; is affected by six chemicals including nicotinic acid, methylmercuric chloride, and manganese chloride based on RNA-seq and microarray studies.
429	WBGene00011621	T08G3.13	Transmembrane transport	Transmembrane transport: potassium channel	Transmembrane transport: potassium channel	T08G3.13 is an ortholog of human KCTD10 (potassium channel tetramerization domain containing 10), KCTD13, and TNFAIP1 (TNF alpha induced protein 1)\; human TNFAIP1 exhibits GTP-Rho binding activity and identical protein binding activity.

430	WBGene00011668	T09F5.9	Stress response	Stress response: C-type Lectin	Stress response: C-type Lectin	clec-47 is an ortholog of human CLEC4A, CLEC4C, and CD209 and members of the human CLEC (C-type lectin domain containing) gene family including CLEC6A and CLEC4E\; is predicted to have carbohydrate binding activity\; is involved in response to gamma radiation.
431	WBGene00011753	T13F3.6	Unassigned	Unassigned	Unassigned: regulated by multiple stresses	T13F3.6 is enriched in the AFD and the hypodermis based on tiling array and RNA-seq studies\; is affected by several genes including daf-16, daf-2, and skn-1 based on microarray, tiling array, and RNA-seq studies\; is affected by twenty-one chemicals including methylmercuric chloride, Mercuric Chloride, and Rotenone based on microarray and RNA-seq studies\; is predicted to encode a protein with the following domain: Domain of unknown function DUF19.
432	WBGene00011767	T14D7.1	Metabolism	Metabolism: amino acid	Metabolism: amino acid: breakdown	agxt-1 is an ortholog of human AGXT\; is predicted to have alanine-glyoxylate transaminase activity and serine-pyruvate transaminase activity\; is expressed in the body wall musculature and the

						intestine.
433	WBGene00011798	T16G1.4	Unassigned	Unassigned	Unassigned: regulated by multiple stresses	T16G1.4 is enriched in several tissues, including the coelomocyte, the germ line, the intestine, and the nervous system based on proteomic, microarray, tiling array, and RNA-seq studies\; is affected by several genes including daf-16, daf-2, and glp-1 based on proteomic, microarray, RNA-seq, and tiling array studies\; is affected by seventeen chemicals including tryptophan, Tunicamycin, and Rifampin based on microarray and RNA-seq studies\; is predicted to encode a protein with the following domains: Protein kinase-like domain superfamily, Uncharacterised oxidoreductase Dhs-27, and CHK kinase-like.
434	WBGene00011800	T16G1.6	Unassigned	Unassigned	Unassigned	T16G1.6 is expressed in head neurons and the intestine\; is predicted to encode a protein with the following domains: Protein kinase-like domain superfamily, Uncharacterised oxidoreductase Dhs-27, and CHK kinase-like.
435	WBGene00011830	T19B10.1	Stress response	Stress response:	Stress response:	cyp-29A2 is an ortholog of human CYP19A1

				detoxification	detoxification: CYP	(cytochrome P450 family 19 subfamily A member 1) and CYP4V2 (cytochrome P450 family 4 subfamily V member 2)\; is predicted to have heme binding activity, iron ion binding activity, and oxidoreductase activity, acting on paired donors, with incorporation or reduction of molecular oxygen\; is expressed in several tissues, including the alimentary system, the body wall musculature, the hypodermis, and the nervous system.
436	WBGene00011831	T19B10.2	Unassigned	Unassigned	Unassigned	T19B10.2 is expressed in the hypodermis.
437	WBGene00011844	T19C9.8	Stress response	Stress response: pathogen	Stress response: pathogen: CUB	T19C9.8 is enriched in the DA neuron, the VA neuron, the coelomocyte, the intestine, and the male based on tiling array, RNA-seq, and microarray studies\; is affected by several genes including daf-16, daf-2, and glp-1 based on tiling array, microarray, and RNA-seq studies\; is affected by eighteen chemicals including Heme, tryptophan, and methylmercury hydroxide based on microarray and RNA-seq studies.
438	WBGene00011888	T21B10.6	Extracellular	Extracellular	Extracellular	cutl-15 is enriched in several tissues, including the

			material	material: cuticlin	material: cuticlin	body wall musculature, the hypodermis, the intestine, and the nervous system based on microarray, tiling array, and RNA-seq studies\; is affected by several genes including daf-16, daf-2, and let-7 based on microarray, tiling array, and RNA-seq studies\; is affected by nine chemicals including Rotenone, Alovudine, and stavudine based on RNA-seq and microarray studies\; is predicted to encode a protein with the following domain: Zona pellucida domain.
439	WBGene00011893	T21C9.6	Signaling	Signaling: unassigned	Signaling: unassigned	T21C9.6 is predicted to have ATP binding activity and kinase activity.
440	WBGene00011895	T21C9.9	Unassigned	Unassigned	Unassigned: regulated by multiple stresses	T21C9.9 is enriched in the hypodermis based on tiling array and RNA-seq studies\; is affected by several genes including daf-16, daf-2, and skn-1 based on microarray, tiling array, RNA-seq, and proteomic studies\; is affected by ten chemicals including 1-methylnicotinamide, nicotinic acid, and D-glucose based on RNA-seq and microarray studies.

441	WBGene00011949	T23F6.1	Unassigned	Unassigned	Unassigned	T23F6.1 is enriched in the DA neuron and the germ line based on RNA-seq and microarray studies\; is affected by several genes including daf-16, daf-2, and skn-1 based on microarray, tiling array, and RNA-seq studies\; is affected by nine chemicals including D-glucose, Neurotoxins, and resveratrol based on RNA-seq and microarray studies.
442	WBGene00011958	T23G4.2	Proteolysis general	Proteolysis general: unassigned	Proteolysis general: unassigned	T23G4.2 is an ortholog of human LONP1 (lon peptidase 1, mitochondrial)\; is predicted to have ATP-dependent peptidase activity and serine-type endopeptidase activity.
443	WBGene00011968	T23G11.1	Transmembran e protein	Transmembrane protein: unassigned	Transmembrane protein: unassigned	T23G11.1 is enriched in the male and the nervous system based on RNA-seq studies\; is affected by several genes including let-7, dpy-10, and gld-1 based on microarray, RNA-seq, and proteomic studies\; is affected by nineteen chemicals including Ethanol, nicotinic acid, and methylmercuric chloride based on RNA-seq and microarray studies.
444	WBGene0001203	T26C5.2	Unassigned	Unassigned	Unassigned:	T26C5.2 is enriched in the OLL, the PVD, the

	2				regulated by multiple stresses	hypodermis, and the intestine based on microarray, tiling array, and RNA-seq studies\; is affected by several genes including daf-16, skn-1, and daf-12 based on microarray and RNA-seq studies\; is affected by thirteen chemicals including Ethanol, methylmercury hydroxide, and 1-methylnicotinamide based on RNA-seq and microarray studies\; is predicted to encode a protein with the following domain: PAN\/Apple domain.
445	WBGene00012076	NA	NA	NA	NA	NA
446	WBGene00012087	T27E7.1	Unassigned	Unassigned	Unassigned: regulated by multiple stresses	T27E7.1 is enriched in the germ line and the male based on proteomic and RNA-seq studies\; is affected by several genes including daf-2, glp-1, and age-1 based on microarray, RNA-seq, and proteomic studies\; is affected by eighteen chemicals including Ethanol, nicotinic acid, and methylmercuric chloride based on RNA-seq and microarray studies.
447	WBGene00012115	T28B8.4	Proteolysis	Proteolysis	Proteolysis	T28B8.4 is an ortholog of human PSME4

			proteasome	proteasome: unassigned	proteasome: unassigned	(proteasome activator subunit 4)\; is predicted to have lysine-acetylated histone binding activity, peptidase activator activity, and proteasome binding activity.
448	WBGene00012134	T28F3.8	Unassigned	Unassigned	Unassigned: regulated by multiple stresses	T28F3.8 is enriched in the DA neuron, the I5 neuron, the SAB, and the retrovesicular ganglion based on microarray studies\; is affected by several genes including daf-16, daf-2, and glp-1 based on microarray and RNA-seq studies\; is affected by nine chemicals including bisphenol S, stavudine, and Cholestanol based on RNA-seq and microarray studies.
449	WBGene00012149	VF13D12L.3	Metabolism	Metabolism: unassigned	Metabolism: unassigned	VF13D12L.3 is predicted to have oxidoreductase activity.
450	WBGene00012168	W01A8.6	Proteolysis general	Proteolysis general: carboxypeptidase	Proteolysis general: carboxypeptidase	W01A8.6 is an ortholog of human CPA4 (carboxypeptidase A4), CPB2 (carboxypeptidase B2), and CPA1 (carboxypeptidase A1)\; is predicted to have metallocarboxypeptidase activity and zinc ion binding activity\; human orthologs of this gene are implicated in familial temporal lobe

						epilepsy 5.
451	WBGene00012184	W01F3.1	Unassigned	Unassigned	Unassigned	mnr-1 is an ortholog of human FAM151A and FAM151B\; is involved in several processes, including dendrite morphogenesis, dendrite morphogenesis, and generation of neurons\; localizes to the extracellular space and plasma membrane\; is expressed in several tissues, including the alimentary system, the excretory cell, the hypodermis, the nervous system, and the reproductive system.
452	WBGene00012249	W04E12.4	Transmembrane protein	Transmembrane protein: unassigned	Transmembrane protein: unassigned	W04E12.4 is enriched in the body wall muscle cell, the epithelial system, the germline precursor cell, the male, and the nervous system based on tiling array and RNA-seq studies\; is affected by several genes including age-1, eat-2, and npr-1 based on microarray and RNA-seq studies\; is affected by ten chemicals including copper sulfate, methylmercuric chloride, and Tunicamycin based on microarray and RNA-seq studies.
453	WBGene0001225	W04E12.7	Proteolysis	Proteolysis	Proteolysis	W04E12.7 is an ortholog of human PGPEP1 and

	2		general	general: unassigned	general: unassigned	PGPEP1L\; is predicted to encode a protein with the following domains: Putative pyroglutamyl peptidase 1, Peptidase C15, pyroglutamyl peptidase I-like, and Peptidase C15, pyroglutamyl peptidase I-like superfamily.
454	WBGene00012257	W04G3.3	Transmembrane transport	Transmembrane transport: lipid	Transmembrane transport: lipid	lpr-4 is an ortholog of human APOD (apolipoprotein D)\; is expressed in the body wall musculature and the muscle cell\; human APOD exhibits cholesterol binding activity.
455	WBGene00012346	W08G11.1	Transmembrane protein	Transmembrane protein: unassigned	Transmembrane protein: unassigned	W08G11.1 is enriched in the hypodermis based on tiling array studies\; is affected by several genes including daf-16, daf-12, and pmk-1 based on microarray, tiling array, and RNA-seq studies\; is affected by nine chemicals including methylmercury hydroxide, methylmercuric chloride, and D-glucose based on RNA-seq and microarray studies\; is predicted to encode a protein with the following domain: Protein of unknown function DUF268, Caenorhabditis species.
456	WBGene0001239	Y6E2A.4	Unassigned	Unassigned	Unassigned:	Y6E2A.4 is enriched in several tissues, including

	8				regulated by multiple stresses	dopaminergic neurons, the Z1.p, the Z4.a, the male distal tip cell, and the somatic gonad precursor based on tiling array and RNA-seq studies\; is affected by several genes including daf-16, daf-2, and glp-1 based on microarray and RNA-seq studies\; is affected by nine chemicals including Tunicamycin, levamisole, and Sodium Chloride based on microarray and RNA-seq studies\; is predicted to encode a protein with the following domain: Protein of unknown function DUF713.
457	WBGene00012404	Y6G8.2	Unassigned	Unassigned	Unassigned: regulated by multiple stresses	Y6G8.2 is enriched in the OLL, the PVD, and the intestine based on microarray and RNA-seq studies\; is affected by several genes including daf-16, daf-2, and glp-1 based on microarray and RNA-seq studies\; is affected by four chemicals including Tunicamycin, D-glucopyranose, and tert-Butylhydroperoxide based on microarray and RNA-seq studies\; is predicted to encode a protein with the following domains: F-box domain and Domain of unknown function DUF38,

						Caenorhabditis species.
458	WBGene00012415	Y7A5A.9	Unassigned	Unassigned	Unassigned	Y7A5A.9 is enriched in the male based on RNA-seq studies\; is affected by several genes including pgl-1, glh-1, and hpl-2 based on tiling array and RNA-seq studies.
459	WBGene00012429	Y11D7A.5	Unassigned	Unassigned	Unassigned	Y11D7A.5 is enriched in the OLL, the PLM, the PVD, the germline precursor cell, and the hypodermis based on microarray, tiling array, and RNA-seq studies\; is affected by several genes including daf-16, daf-12, and lin-4 based on RNA-seq, tiling array, and microarray studies\; is affected by fourteen chemicals including Heme, methylmercury hydroxide, and 1-methylnicotinamide based on microarray and RNA-seq studies.
460	WBGene00012433	Y11D7A.9	Stress response	Stress response: unassigned	Stress response: unassigned	Y11D7A.9 is an ortholog of human PGAP2\; is predicted to encode a protein with the following domain: Frag1\/DRAM\/Sfk1.
461	WBGene00012437	Y11D7A.14	Muscle function	Muscle function	Muscle function	nmy-3 is an ortholog of human MYH3 (myosin heavy chain 3), MYH15 (myosin heavy chain 15),

						and MYH7B (myosin heavy chain 7B)\; is predicted to have ATP binding activity, actin filament binding activity, and motor activity.
462	WBGene00012520	Y32B12A.5	Unassigned	Unassigned	Unassigned	Y32B12A.5 is affected by rsr-2 based on tiling array studies\; is affected by four chemicals including nanoparticle, Cadmium, and Microcystin-LR based on microarray studies.
463	WBGene00012531	Y32F6B.1	Transmembrane transport	Transmembrane transport: solute carrier	Transmembrane transport: solute carrier	Y32F6B.1 is an ortholog of human SLCO3A1 (solute carrier organic anion transporter 3A1)\; is predicted to have transporter activity.
464	WBGene00012629	Y38H6C.17	Transmembrane transport	Transmembrane transport: amino acid	Transmembrane transport: amino acid	slc-36.3 is an ortholog of human SLC36A3 (solute carrier family 36 member 3), SLC36A2 (solute carrier family 36 member 2), and SLC36A1 (solute carrier family 36 member 1)\; human SLC36A2 exhibits glycine transmembrane transporter activity.
465	WBGene00012689	Y39B6A.30	Signaling	Signaling: phosphatase	Signaling: phosphatase: Y	Y39B6A.30 is predicted to have protein tyrosine phosphatase activity.
466	WBGene00012791	Y43D4A.5	Unassigned	Unassigned	Unassigned: regulated by multiple stresses	Y43D4A.5 is enriched in the Z1.p, the Z4.a, the male distal tip cell, and the somatic gonad precursor based on RNA-seq studies\; is affected by

						several genes including daf-16, daf-12, and hsf-1 based on microarray and RNA-seq studies\; is affected by ten chemicals including Heme, hydrogen sulfide, and methylmercury hydroxide based on microarray and RNA-seq studies.
467	WBGene00012810	Y43F8A.3	Metabolism	Metabolism: unassigned	Metabolism: unassigned	nceh-1 is an ortholog of human AADAC (arylacetamide deacetylase) and NCEH1\; is predicted to have carboxylic ester hydrolase activity.
468	WBGene00012827	Y43F8C.5	Unassigned	Unassigned	Unassigned: regulated by multiple stresses	Y43F8C.5 is enriched in the NSM and the male based on tiling array and RNA-seq studies\; is affected by several genes including dpy-10, sir-2.1, and npr-1 based on microarray and RNA-seq studies\; is affected by fifteen chemicals including Ethanol, methylmercuric chloride, and manganese chloride based on RNA-seq and microarray studies.
469	WBGene00012901	Y46G5A.10	Extracellular material	Extracellular material: mucin	Extracellular material: mucin	cnp-2 is expressed in the intestine.
470	WBGene00012917	Y46G5A.29	Unassigned	Unassigned	Unassigned	Y46G5A.29 is predicted to have calcium ion binding activity.

471	WBGene00012919	Y46G5A.34	Unassigned	Unassigned	Unassigned: regulated by multiple stresses	Y46G5A.34 is enriched in the alimentary system, the digestive tract, the muscle cell, and the nervous system based on tiling array, RNA-seq, and microarray studies\; is affected by several genes including daf-2, clk-1, and elt-2 based on microarray, tiling array, and RNA-seq studies\; is affected by five chemicals including Rotenone, manganese chloride, and Hydrolyzable Tannins based on RNA-seq and microarray studies.
472	WBGene00012979	Y48B6A.5	Unassigned	Unassigned	Unassigned	Y48B6A.5 is enriched in the male based on RNA-seq studies\; is affected by several genes including daf-16, daf-2, and dpy-10 based on microarray and RNA-seq studies\; is affected by twelve chemicals including Ethanol, methylmercuric chloride, and manganese chloride based on RNA-seq and microarray studies\; is predicted to encode a protein with the following domains: Major sperm protein (MSP) domain, PapD-like superfamily, and Immunoglobulin-like fold.
473	WBGene0001305	Y50E8A.9	Metabolism	Metabolism:	Metabolism:	scrm-7 is an ortholog of human PLSCR5

	2			lipid	lipid: phospholipid	(phospholipid scramblase 5), PLSCR4 (phospholipid scramblase 4), and PLSCR1 (phospholipid scramblase 1)\; human PLSCR1 exhibits several functions, including SH3 domain binding activity, calcium ion binding activity, and signaling receptor binding activity.
474	WBGene00013138	Y53C10A.10	Unassigned	Unassigned	Unassigned: regulated by multiple stresses	Y53C10A.10 is enriched in the amphid sheath cell, the germ line, and the male based on proteomic and RNA-seq studies\; is affected by several genes including daf-2, eat-2, and sir-2.1 based on microarray, RNA-seq, and tiling array studies\; is affected by four chemicals including hydrogen sulfide, methylmercuric chloride, and manganese chloride based on microarray and RNA-seq studies.
475	WBGene00013175	Y53F4B.36	Unassigned	Unassigned	Unassigned: regulated by multiple stresses	Y53F4B.36 is enriched in the male and the nervous system based on RNA-seq and microarray studies\; is affected by several genes including hsf-1, clk-1, and npr-1 based on microarray, tiling array, and RNA-seq studies\; is affected by D-glucose, paraquat, and Colistin based on RNA-seq and

						microarray studies.
476	WBGene00013194	Y54E2A.9	Extracellular material	Extracellular material: matrix	Extracellular material: matrix	Y54E2A.9 is an ortholog of human VMO1\; is predicted to encode a protein with the following domains: Vitelline membrane outer layer protein I (VOMI) and Vitelline membrane outer layer protein I (VOMI) superfamily.
477	WBGene00013284	Y57A10C.6	Metabolism	Metabolism: lipid	Metabolism: lipid: sterol	daf-22 is an ortholog of human Scp2\; exhibits propanoyl-CoA C-acyltransferase activity\; is involved in several processes, including acyl-CoA metabolic process, ascaroside biosynthetic process, and dauer larval development\; localizes to the peroxisome\; is expressed in several tissues, including the amphid sensillum, the body wall musculature, the hypodermis, the intestine, and the nervous system\; is used to study obesity.
478	WBGene00013352	Y59A8B.20	Extracellular material	Extracellular material: secreted protein	Extracellular material: secreted protein	lon-8 is involved in negative regulation of growth and nematode male tail tip morphogenesis\; is expressed in the hyp4, the hyp7 syncytium, and the intestine.
479	WBGene0001338	Y62E10A.19	Unassigned	Unassigned	Unassigned	Y62E10A.19 is enriched in dopaminergic neurons,

	5					the PLM, the germline precursor cell, the hypodermis, and the pharyngeal muscle cell based on tiling array and RNA-seq studies\; is affected by several genes including daf-16, daf-12, and bar-1 based on microarray and RNA-seq studies\; is affected by five chemicals including Ethanol, Rotenone, and stavudine based on RNA-seq and microarray studies.
480	WBGene00013429	Y66D12A.3	Unassigned	Unassigned	Unassigned: regulated by multiple stresses	Y66D12A.3 is enriched in the amphid sheath cell and the male based on RNA-seq studies\; is affected by several genes including daf-2, age-1, and clk-1 based on microarray and RNA-seq studies\; is affected by eight chemicals including methylmercuric chloride, manganese chloride, and D-glucose based on microarray and RNA-seq studies.
481	WBGene00013473	Y69E1A.1	Transmembrane protein	Transmembrane protein: unassigned	Transmembrane protein: unassigned	Y69E1A.1 is enriched in the body wall muscle cell, the coelomocyte, the hypodermis, the male, and the nervous system based on tiling array and RNA-seq studies\; is affected by several genes including daf-

						2, age-1, and eat-2 based on microarray and RNA-seq studies\; is affected by nine chemicals including Ethanol, methylmercury hydroxide, and methylmercuric chloride based on RNA-seq and microarray studies\; is predicted to encode a protein with the following domain: Protein of unknown function DUF856, Caenorhabditis species.
482	WBGene00013474	Y69E1A.2	Unassigned	Unassigned	Unassigned	Y69E1A.2 is enriched in the NSM and the male based on tiling array and microarray studies\; is affected by several genes including daf-2, glp-1, and age-1 based on microarray, tiling array, RNA-seq, and proteomic studies\; is affected by twenty-four chemicals including Ethanol, methylmercury hydroxide, and nicotinic acid based on RNA-seq and microarray studies\; is predicted to encode a protein with the following domain: Cytosolic motility protein.
483	WBGene00013477	Y69E1A.5	Metabolism	Metabolism: lipid	Metabolism: lipid: binding	Y69E1A.5 is an ortholog of human PEBP4 and Pebp1\; human PEBP1 exhibits protein kinase binding activity.

484	WBGene00013495	Y70C5C.5	Pseudogene	Pseudogene	Pseudogene	clec-236 is enriched in the NSM and the male based on tiling array and RNA-seq studies\; is affected by several genes including hsf-1, clk-1, and eat-2 based on RNA-seq and microarray studies\; is affected by eight chemicals including hydrogen sulfide, methylmercuric chloride, and Tunicamycin based on microarray and RNA-seq studies.
485	WBGene00013696	Y106G6A.4	Unassigned	Unassigned	Unassigned: regulated by multiple stresses	Y106G6A.4 is enriched in several tissues, including the Z1.p, the Z4.a, the excretory cell, the male distal tip cell, and the somatic gonad precursor based on tiling array, RNA-seq, and microarray studies\; is affected by several genes including age-1, daf-12, and dpy-10 based on microarray, tiling array, and RNA-seq studies\; is affected by fifteen chemicals including Ethanol, methylmercuric chloride, and D-glucose based on RNA-seq and microarray studies\; is predicted to encode a protein with the following domains: BAR domain and AH\/BAR domain superfamily.

486	WBGene00013700	Y106G6D.3	Unassigned	Unassigned	Unassigned: regulated by multiple stresses	Y106G6D.3 is enriched in the male based on RNA-seq studies\; is affected by several genes including daf-2, glp-1, and dpy-10 based on microarray and RNA-seq studies\; is affected by eighteen chemicals including nicotinic acid, methylmercuric chloride, and manganese chloride based on RNA-seq and microarray studies.
487	WBGene00013713	Y106G6G.4	Transmembrane protein	Transmembrane protein: unassigned	Transmembrane protein: unassigned	Y106G6G.4 is enriched in GABAergic neurons, the AVA, the DA neuron, the VA neuron, and the male based on microarray, tiling array, and RNA-seq studies\; is affected by several genes including daf-2, dpy-10, and gld-1 based on microarray and RNA-seq studies\; is affected by nineteen chemicals including Ethanol, nicotinic acid, and methylmercuric chloride based on RNA-seq and microarray studies.
488	WBGene00013762	Y113G7B.12	Unassigned	Unassigned	Unassigned: regulated by multiple stresses	Y113G7B.12 is an ortholog of human COL24A1 (collagen type XXIV alpha 1 chain)\; is predicted to encode a protein with the following domains: Reverse transcriptase domain,

						Endonuclease\/exonuclease\/phosphatase, and Endonuclease\/exonuclease\/phosphatase superfamily.
489	WBGene00013785	Y116A8C.4	Proteolysis general	Proteolysis general: metallopeptidase	Proteolysis general: metallopeptidase	nep-23 is an ortholog of human ECEL1 (endothelin converting enzyme like 1), MME, and PHEX (phosphate regulating endopeptidase homolog X-linked)\; is predicted to have metalloendopeptidase activity.
490	WBGene00013860	ZC247.2	Unassigned	Unassigned	Unassigned: regulated by multiple stresses	ZC247.2 is enriched in GABAergic neurons, the OLL, the PVD, and the muscle cell based on microarray, tiling array, and RNA-seq studies\; is affected by several genes including daf-16, daf-12, and eat-2 based on RNA-seq and microarray studies\; is affected by eight chemicals including aldicarb, stavudine, and Zidovudine based on microarray and RNA-seq studies.
491	WBGene00013870	ZC373.5	Metabolism	Metabolism: amino acid	Metabolism: amino acid: synthesis	ZC373.5 is an ortholog of human APIP\; is predicted to have methylthioribulose 1-phosphate dehydratase activity and zinc ion binding activity\; is expressed in several tissues, including the

						alimentary system, the epithelial system, the muscular system, the reproductive system, and the tail.
492	WBGene00013874	ZC376.2	Metabolism	Metabolism: carbohydrate	Metabolism: carbohydrate	ZC376.2 is an ortholog of human ACHE (acetylcholinesterase (Cartwright blood group)) and CES2 (carboxylesterase 2)\; human ACHE exhibits several functions, including acetylcholine binding activity, acetylcholinesterase activity, and collagen binding activity.
493	WBGene00013875	ZC376.3	Stress response	Stress response: detoxification	Stress response: detoxification: unassigned	ZC376.3 is an ortholog of human ACHE (acetylcholinesterase (Cartwright blood group)) and CES2 (carboxylesterase 2)\; human ACHE exhibits several functions, including acetylcholine binding activity, acetylcholinesterase activity, and collagen binding activity.
494	WBGene00013891	ZC434.3	Unassigned	Unassigned	Unassigned	ZC434.3 is enriched in the PLM, the germline precursor cell, and the hypodermis based on tiling array and RNA-seq studies\; is affected by several genes including daf-16, daf-2, and daf-12 based on microarray and RNA-seq studies\; is affected by

						eight chemicals including aldicarb, Ethanol, and methylmercury hydroxide based on microarray and RNA-seq studies.
495	WBGene00013895	ZC434.9	Proteolysis general	Proteolysis general: carboxypeptidase	Proteolysis general: carboxypeptidase	ZC434.9 is an ortholog of human CPA4 (carboxypeptidase A4), CPA1 (carboxypeptidase A1), and CPA6 (carboxypeptidase A6)\; is predicted to have metallocarboxypeptidase activity and zinc ion binding activity\; human orthologs of this gene are implicated in familial temporal lobe epilepsy 5.
496	WBGene00013926	ZK20.6	Proteolysis general	Proteolysis general: metallopeptidase	Proteolysis general: metallopeptidase	nep-1 is an ortholog of human ECE1 (endothelin converting enzyme 1) and ECE2 (endothelin converting enzyme 2)\; is predicted to have metal ion binding activity and metalloendopeptidase activity\; is expressed in the RIH and the pharynx.
497	WBGene00013943	ZK218.11	Unassigned	Unassigned	Unassigned	ZK218.11 is enriched in the muscle cell based on microarray studies\; is affected by several genes including daf-2, clk-1, and sir-2.1 based on tiling array, RNA-seq, and microarray studies\; is affected by resveratrol and Sirolimus based on microarray

						studies\; is predicted to encode a protein with the following domain: ShKT domain.
498	WBGene00013956	ZK265.3	Unassigned	Unassigned	Unassigned: regulated by multiple stresses	ZK265.3 is enriched in the NSM, the amphid sheath cell, and the male based on tiling array and RNA-seq studies\; is affected by several genes including daf-2, age-1, and dpy-10 based on microarray and RNA-seq studies\; is affected by seventeen chemicals including Ethanol, nicotinic acid, and methylmercuric chloride based on RNA-seq and microarray studies\; is predicted to encode a protein with the following domain: Cytosolic motility protein.
499	WBGene00013986	ZK512.7	Unassigned	Unassigned	Unassigned	ZK512.7 is affected by several genes including daf-16, daf-2, and daf-12 based on microarray, RNA-seq, and proteomic studies\; is affected by fifteen chemicals including Ethanol, Rotenone, and Tunicamycin based on RNA-seq and microarray studies.
500	WBGene00013987	ZK512.8	Unassigned	Unassigned	Unassigned: regulated by	ZK512.8 is enriched in the NSM, the arcade cell, the germ line, the male, and the pharyngeal-

					multiple stresses	intestinal valve cell based on proteomic, tiling array, and RNA-seq studies\; is affected by several genes including glp-1, let-7, and dpy-10 based on microarray, tiling array, RNA-seq, and proteomic studies\; is affected by twelve chemicals including Ethanol, nicotinic acid, and methylmercuric chloride based on RNA-seq and microarray studies\; is predicted to encode a protein with the following domain: Protein of unknown function DUF5386.
501	WBGene00014003	ZK593.3	Transmembrane protein	Transmembrane protein: unassigned	Transmembrane protein: unassigned	ZK593.3 is enriched in the coelomocyte and the intestine based on tiling array and RNA-seq studies\; is affected by several genes including daf-16, daf-2, and daf-12 based on tiling array, RNA-seq, and microarray studies\; is affected by fourteen chemicals including 1-methylnicotinamide, D-glucopyranose, and D-glucose based on RNA-seq and microarray studies.
502	WBGene00014039	ZK662.2	Unassigned	Unassigned	Unassigned: regulated by	ZK662.2 is enriched in the DA neuron and the pharynx based on microarray studies\; is affected

					multiple stresses	by several genes including daf-16, glp-1, and skn-1 based on microarray, tiling array, and RNA-seq studies\; is affected by eighteen chemicals including 1-methylnicotinamide, nicotinic acid, and Rotenone based on RNA-seq and microarray studies.
503	WBGene00014046	ZK666.6	Stress response	Stress response: C-type Lectin	Stress response: C-type Lectin	clec-60 is predicted to have carbohydrate binding activity\; is involved in defense response to Gram-positive bacterium\; is expressed in the intestine.
504	WBGene00014088	ZK809.8	Development	Development: somatic	Development: somatic	ZK809.8 is enriched in the PLM, the intestine, and the muscle cell based on tiling array, RNA-seq, and microarray studies\; is affected by several genes including daf-2, glp-1, and hsf-1 based on RNA-seq and microarray studies\; is affected by Cholestanol and paraquat based on microarray studies.
505	WBGene00014106	ZK856.5	Metabolism	Metabolism: lipid	Metabolism: lipid: sphingolipid	ZK856.5 is an ortholog of human SMPDL3A and SMPDL3B\; is predicted to have hydrolase activity.
506	WBGene00014158	ZK938.1	Signaling	Signaling: phosphatase	Signaling: phosphatase:	ZK938.1 is an ortholog of human PPP1CB (protein phosphatase 1 catalytic subunit beta)\; is predicted

					unassigned	to have phosphoprotein phosphatase activity.
507	WBGene00014207	ZK1058.9	Unassigned	Unassigned	Unassigned: regulated by multiple stresses	ZK1058.9 is enriched in the germ line and the muscle cell based on tiling array, proteomic, RNA-seq, and microarray studies\; is affected by several genes including daf-16, glp-1, and daf-12 based on tiling array, microarray, and RNA-seq studies\; is affected by ten chemicals including methylmercuric chloride, Rotenone, and D-glucose based on microarray and RNA-seq studies.
508	WBGene00014238	ZK1225.4	Unassigned	Unassigned	Unassigned: regulated by multiple stresses	ZK1225.4 is enriched in the male based on RNA-seq studies\; is affected by several genes including daf-2, dpy-10, and hsf-1 based on microarray, RNA-seq, and tiling array studies\; is affected by twelve chemicals including Ethanol, methylmercury hydroxide, and methylmercuric chloride based on RNA-seq and microarray studies.
509	WBGene00014240	ZK1251.1	Transcription: chromatin	Transcription: chromatin structure	Transcription: chromatin structure: histone	htas-1 is an ortholog of human H2AFY (H2A histone Y) and H2AFY2 (H2A histone Y2)\; is predicted to have DNA binding activity and protein heterodimerization activity\; is expressed in the

						sperm.
510	WBGene00014262	ZK1321.4	Unassigned	Unassigned	Unassigned	ZK1321.4 is an ortholog of human PDLIM5, PDLIM2, and PRICKLE4\; human PDLIM5 exhibits cadherin binding activity involved in cell-cell adhesion.
511	WBGene00014666	C05D12.3	Metabolism	Metabolism: amino sugar	Metabolism: amino sugar	C05D12.3 is enriched in the body wall muscle cell, the intestine, and the nervous system based on microarray, tiling array, and RNA-seq studies\; is affected by several genes including daf-16, daf-2, and hsf-1 based on RNA-seq and microarray studies\; is affected by twelve chemicals including nicotinic acid, Tunicamycin, and D-glucose based on RNA-seq and microarray studies\; is predicted to encode a protein with the following domain: von Willebrand factor A-like domain superfamily.
512	WBGene00014697	C36A4.3	Stress response	Stress response: detoxification	Stress response: detoxification: CYP	cyp-25A3 is an ortholog of human CYP3A7-CYP3A51P and TBXAS1 (thromboxane A synthase 1) and members of the human CYP3 (Cytochrome P450 family 3) gene family including CYP3A4, CYP3A43, and CYP3A7\; is predicted to have

						heme binding activity, iron ion binding activity, and oxidoreductase activity\; human orthologs of this gene are implicated in essential hypertension and platelet-type bleeding disorder 14.
513	WBGene00015030	B0207.7	Cytoskeleton	Cytoskeleton: microtubule	Cytoskeleton: microtubule: tau tubulin kinase	B0207.7 is an ortholog of human CTD-2116N17.1, TTBK1 (tau tubulin kinase 1), and TTBK2\; is predicted to have ATP binding activity and protein kinase activity\; is expressed in the AIYL and the AIYR.
514	WBGene00015044	B0213.15	Stress response	Stress response: detoxification	Stress response: detoxification: CYP	cyp-34A9 is an ortholog of human CYP21A2 (cytochrome P450 family 21 subfamily A member 2) and members of the human CYP2 (Cytochrome P450 family 2) gene family including CYP2R1, CYP2U1, and CYP2J2\; is predicted to have heme binding activity, iron ion binding activity, and oxidoreductase activity\; is expressed in the intestine\; human orthologs of this gene are implicated in congenital adrenal hyperplasia.
515	WBGene00015049	B0218.5	Cytoskeleton	Cytoskeleton: microtubule	Cytoskeleton: microtubule: tau	B0218.5 is an ortholog of human CTD-2116N17.1, TTBK1 (tau tubulin kinase 1), and TTBK2\; is

					tubulin kinase	predicted to have ATP binding activity and protein kinase activity.
516	WBGene00015050	B0218.6	Stress response	Stress response: C-type Lectin	Stress response: C-type Lectin	clec-51 is an ortholog of human COLEC11, CD302, and LY75\; is predicted to have carbohydrate binding activity.
517	WBGene00015057	B0222.9	Metabolism	Metabolism: nucleotide	Metabolism: nucleotide	gad-3 is an ortholog of human AOX1 and XDH\; is predicted to have several functions, including 2 iron, 2 sulfur cluster binding activity, flavin adenine dinucleotide binding activity, and oxidoreductase activity\; is involved in endoplasmic reticulum unfolded protein response.
518	WBGene00015084	B0244.9	Transmembrane protein	Transmembrane protein: unassigned	Transmembrane protein: unassigned	B0244.9 is enriched in the male based on RNA-seq studies\; is affected by several genes including age-1, dpy-10, and clk-1 based on microarray and RNA-seq studies\; is affected by twelve chemicals including methylmercuric chloride, Rotenone, and manganese chloride based on microarray and RNA-seq studies.
519	WBGene00015103	B0280.7	Unassigned	Unassigned	Unassigned	B0280.7 is expressed in the g1AR, the g1P, and the pharynx.

520	WBGene00015116	B0286.3	Metabolism	Metabolism: nucleotide	Metabolism: nucleotide	pacs-1 is an ortholog of human PAICS (phosphoribosylaminoimidazole carboxylase and phosphoribosylaminoimidazolesuccinocarboxamide synthase)\; exhibits identical protein binding activity.
521	WBGene00015137	B0310.1	Transmembrane protein	Transmembrane protein: unassigned	Transmembrane protein: unassigned	B0310.1 is enriched in the muscle cell based on tiling array, proteomic, and microarray studies\; is affected by several genes including daf-2, eat-2, and pgl-1 based on microarray and RNA-seq studies\; is affected by four chemicals including Zidovudine, resveratrol, and Chlorpyrifos based on RNA-seq and microarray studies.
522	WBGene00015169	B0403.5	Unassigned	Unassigned	Unassigned	B0403.5 is enriched in the Z1, the Z4, and the hypodermis based on tiling array and RNA-seq studies\; is affected by several genes including daf-16, glp-1, and daf-12 based on tiling array and RNA-seq studies\; is affected by four chemicals including D-glucose, 4-bromodiphenyl ether, and stavudine based on RNA-seq and microarray studies.

523	WBGene00015193	B0432.12	Stress response	Stress response: C-type Lectin	Stress response: C-type Lectin	clec-117 is predicted to have carbohydrate binding activity.
524	WBGene00015284	C01B10.10	Transmembrane protein	Transmembrane protein: unassigned	Transmembrane protein: unassigned	C01B10.10 is enriched in the intestine based on tiling array and RNA-seq studies\; is affected by several genes including daf-16, daf-2, and dpy-10 based on RNA-seq, microarray, and tiling array studies\; is affected by seven chemicals including levamisole, stavudine, and Diazinon based on microarray and RNA-seq studies\; is predicted to encode a protein with the following domains: Carboxylesterase, type B and Alpha\/Beta hydrolase fold.
525	WBGene00015300	C01F1.5	Proteolysis general	Proteolysis general: metallopeptidase	Proteolysis general: metallopeptidase	C01F1.5 is predicted to have metallopeptidase activity.
526	WBGene00015335	C02D5.1	Metabolism	Metabolism: lipid	Metabolism: lipid: unassigned	acdh-6 is an ortholog of human ACADL (acyl-CoA dehydrogenase long chain)\; is predicted to have flavin adenine dinucleotide binding activity and oxidoreductase activity, acting on the CH-CH group of donors.

527	WBGene00015342	C02E7.10	Transmembrane protein	Transmembrane protein: unassigned	Transmembrane protein: unassigned	C02E7.10 is an ortholog of human DEPDC4 and DEPDC7.
528	WBGene00015389	C03F11.3	Transmembrane protein	Transmembrane protein: unassigned	Transmembrane protein: unassigned	scav-1 is an ortholog of human SCARB1 (scavenger receptor class B member 1)\; human SCARB1 exhibits several functions, including apolipoprotein A-I binding activity, high-density lipoprotein particle receptor activity, and lipopolysaccharide binding activity.
529	WBGene00015400	C03G6.15	Stress response	Stress response: detoxification	Stress response: detoxification: CYP	cyp-35A2 is an ortholog of human CYP21A2 (cytochrome P450 family 21 subfamily A member 2) and members of the human CYP2 (Cytochrome P450 family 2) gene family including CYP2R1, CYP2U1, and CYP2J2\; is predicted to have heme binding activity, iron ion binding activity, and oxidoreductase activity\; is involved in response to xenobiotic stimulus\; is expressed in the intestine\; human orthologs of this gene are implicated in congenital adrenal hyperplasia.
530	WBGene0001540	C03H5.5	Ribosome	Ribosome: EIF	Ribosome: EIF	C03H5.5 is an ortholog of human EIF3L

	7					(eukaryotic translation initiation factor 3 subunit L)\; is predicted to have translation initiation factor activity.
531	WBGene00015453	C04G6.2	Unassigned	Unassigned	Unassigned: regulated by multiple stresses	C04G6.2 is enriched in the hypodermis based on tiling array studies\; is affected by several genes including daf-16, daf-12, and let-7 based on tiling array, microarray, and RNA-seq studies\; is affected by eight chemicals including Nitric Oxide, Rotenone, and Tunicamycin based on RNA-seq and microarray studies\; is predicted to encode a protein with the following domain: Protein of unknown function DUF282, Caenorhabditis species.
532	WBGene00015464	C05C8.7	Metabolism	Metabolism: carbohydrate	Metabolism: carbohydrate	C05C8.7 is an ortholog of human MPI\; is predicted to have mannose-6-phosphate isomerase activity and zinc ion binding activity\; is expressed in the seam cell\; human orthologs of this gene are implicated in congenital disorder of glycosylation type I.
533	WBGene00015469	C05D2.8	Unassigned	Unassigned	Unassigned: regulated by	C05D2.8 is enriched in the OLL, the PVD, the intestine, and the pharyngeal muscle cell based on

					multiple stresses	SAGE, microarray, tiling array, RNA-seq, and proteomic studies\; is affected by several genes including daf-16, daf-2, and glp-1 based on microarray, tiling array, and RNA-seq studies\; is affected by eight chemicals including resveratrol, Cry5B, and Quercetin based on microarray studies.
534	WBGene00015472	C05D9.3	Cytoskeleton	Cytoskeleton: integrin	Cytoskeleton: integrin	C05D9.3 is an ortholog of human ITGBL1 (integrin subunit beta like 1)\; localizes to the plasma membrane.
535	WBGene00015516	C06A8.6	Cytoskeleton	Cytoskeleton: motor protein	Cytoskeleton: motor protein: dynein	C06A8.6 is an ortholog of human DRC3 (dynein regulatory complex subunit 3)\; is predicted to encode a protein with the following domains: Leucine-rich repeat, typical subtype, U2A'\/phosphoprotein 32 family A, C-terminal, Leucine rich repeat 4, and Leucine-rich repeat domain superfamily.
536	WBGene00015522	C06E1.7	Protein modification	Protein modification: carbohydrate	Protein modification: carbohydrate	C06E1.7 is predicted to have galactoside 2-alpha-L-fucosyltransferase activity.
537	WBGene0001552	C06E2.2	Unassigned	Unassigned	Unassigned	C06E2.2 is affected by pgrn-1, clk-1, and daf-16

	8					based on RNA-seq and microarray studies.
538	WBGene00015534	C06E4.5	Unassigned	Unassigned	Unassigned	C06E4.5 is an ortholog of human GSG2\; human HASPIN exhibits ATP binding activity and histone kinase activity (H3-T3 specific).
539	WBGene00015544	C06E8.5	Unassigned	Unassigned	Unassigned	C06E8.5 is predicted to have lipid binding activity.
540	WBGene00015545	C06G1.1	Unassigned	Unassigned	Unassigned	C06G1.1 is predicted to have lipid binding activity\; is expressed in the epithelial system, the excretory system, and the vulval muscle.
541	WBGene00015634	C09D4.3	Cytoskeleton	Cytoskeleton: microtubule	Cytoskeleton: microtubule: tau tubulin kinase	C09D4.3 is an ortholog of human CTD-2116N17.1, TTBK1 (tau tubulin kinase 1), and TTBK2\; is predicted to have ATP binding activity and protein kinase activity.
542	WBGene00015645	C09E8.2	Metabolism	Metabolism: lipid	Metabolism: lipid: lipase	lips-7 is predicted to have hydrolase activity\; is expressed in the excretory system, the head, the rectum, and the vulva.
543	WBGene00015646	C09E8.3	Development	Development: somatic	Development: somatic	mlt-10 is involved in cuticle development involved in collagen and cuticulin-based cuticle molting cycle and epidermis development\; localizes to the extracellular space\; is expressed in the epithelial

						system.
544	WBGene00015688	C10G11.8	Proteolysis proteasome	Proteolysis proteasome: 19S	Proteolysis proteasome: 19S	C10G11.8 is an ortholog of human PSMC1 (proteasome 26S subunit, ATPase 1)\; is predicted to have ATP binding activity and hydrolase activity.
545	WBGene00015695	C10H11.6	Stress response	Stress response: detoxification	Stress response: detoxification: ugt	ugt-26 is an ortholog of human UGT3A1 (UDP glycosyltransferase family 3 member A1) and UGT3A2 (UDP glycosyltransferase family 3 member A2)\; is predicted to have glucuronosyltransferase activity.
546	WBGene00015725	C13A2.9	Transmembrane protein	Transmembrane protein: unassigned	Transmembrane protein: unassigned	C13A2.9 is affected by several genes including daf-16, daf-12, and sir-2.1 based on RNA-seq and microarray studies\; is affected by methylmercuric chloride and single-walled carbon nanotube based on microarray studies\; is predicted to encode a protein with the following domain: Methyltransferase FkbM.
547	WBGene00015739	C13D9.9	Stress response	Stress response: detoxification	Stress response: detoxification: ugt	ugt-7 is an ortholog of human UGT3A1 (UDP glycosyltransferase family 3 member A1) and UGT3A2 (UDP glycosyltransferase family 3 member A2)\; is predicted to have transferase

						activity, transferring hexosyl groups.
548	WBGene00015756	C14C6.2	Unassigned	Unassigned	Unassigned	C14C6.2 is enriched in the OLL, the PVD, and the intestine based on microarray, tiling array, and RNA-seq studies\; is affected by several genes including daf-16, daf-2, and glp-1 based on microarray, tiling array, and RNA-seq studies\; is affected by eight chemicals including Ethanol, D-glucopyranose, and stavudine based on RNA-seq and microarray studies\; is predicted to encode a protein with the following domain: ShKT domain.
549	WBGene00015761	C14C6.7	Pseudogene	Pseudogene	Pseudogene	C14C6.7 is affected by several genes including daf-2, isp-1, and dpy-21 based on tiling array, RNA-seq, and microarray studies\; is affected by Cadmium based on microarray studies.
550	WBGene00015780	C14F11.4	Unassigned	Unassigned	Unassigned: regulated by multiple stresses	C14F11.4 is enriched in the germ line and the intestine based on RNA-seq and microarray studies\; is affected by several genes including daf-2, glp-1, and daf-12 based on microarray, proteomic, tiling array, and RNA-seq studies\; is affected by fourteen chemicals including Ethanol,

						Rotenone, and manganese chloride based on RNA-seq and microarray studies.
551	WBGene00015794	C15F1.1	Unassigned	Unassigned	Unassigned	C15F1.1 is an ortholog of human THAP4 (THAP domain containing 4)\; human THAP4 exhibits heme binding activity and protein homodimerization activity.
552	WBGene00015795	C15F1.2	Unassigned	Unassigned	Unassigned: regulated by multiple stresses	C15F1.2 is enriched in the OLL, the PVD, and the male based on microarray studies\; is affected by several genes including daf-2, let-60, and sir-2.1 based on microarray, tiling array, and RNA-seq studies\; is affected by fifteen chemicals including Lithium Chloride, methylmercury hydroxide, and 1-methylnicotinamide based on microarray and RNA-seq studies.
553	WBGene00015802	C15H9.7	Metabolism	Metabolism: amino acid	Metabolism: amino acid: breakdown	kynu-1 is an ortholog of human KYNU\; is predicted to have kynureninase activity and pyridoxal phosphate binding activity.
554	WBGene00015860	C16D9.5	Unassigned	Unassigned	Unassigned: regulated by multiple stresses	C16D9.5 is enriched in the male and the nervous system based on tiling array, RNA-seq, and microarray studies\; is affected by several genes

						including daf-2, clk-1, and eat-2 based on microarray and RNA-seq studies\; is affected by five chemicals including stavudine, Zidovudine, and paraquat based on RNA-seq and microarray studies\; is predicted to encode a protein with the following domain: Protein of unknown function DUF1647.
555	WBGene00015887	C17C3.1	Metabolism	Metabolism: lipid	Metabolism: lipid: beta oxidation	C17C3.1 is an ortholog of human ACOT8 (acyl-CoA thioesterase 8)\; is predicted to have acyl-CoA hydrolase activity.
556	WBGene00015890	C17C3.5	Unassigned	Unassigned	Unassigned	C17C3.5 is enriched in the body wall muscle cell based on tiling array studies\; is affected by several genes including hsf-1, clk-1, and sir-2.1 based on tiling array, microarray, and RNA-seq studies\; is affected by eleven chemicals including multi-walled carbon nanotube, Ethanol, and Atrazine based on RNA-seq and microarray studies\; is predicted to encode a protein with the following domain: Domain of unknown function DUF38, Caenorhabditis species.

557	WBGene00015931	C17H12.5	Signaling	Signaling: phosphatase	Signaling: phosphatase: Y	C17H12.5 is predicted to have protein tyrosine phosphatase activity.
558	WBGene00015954	C18B2.3	Unassigned	Unassigned	Unassigned	C18B2.3 is expressed in the anal depressor muscle, the body wall musculature, and the vulval muscle.
559	WBGene00015992	C18H2.5	Transmembrane protein	Transmembrane protein: unassigned	Transmembrane protein: unassigned	C18H2.5 is enriched in the germ line and the male based on RNA-seq studies\; is affected by several genes including daf-2, eat-2, and npr-1 based on RNA-seq and tiling array studies\; is affected by methylmercuric chloride, manganese chloride, and D-glucose based on microarray and RNA-seq studies\; is predicted to encode a protein with the following domains: BRCT domain, Ankyrin repeat, Domain of unknown function WSN, BRCT domain superfamily, and Ankyrin repeat-containing domain superfamily.
560	WBGene00016013	C23G10.6	Stress response	Stress response: detoxification	Stress response: detoxification: ugt	ugt-66 is an ortholog of human UGT3A1 (UDP glycosyltransferase family 3 member A1) and UGT3A2 (UDP glycosyltransferase family 3 member A2)\; is predicted to have glucuronosyltransferase activity.

561	WBGene00016046	C24B5.4	Unassigned	Unassigned	Unassigned: hydrolase	C24B5.4 is an ortholog of human C11orf54\; is expressed in the body wall musculature\; human C11orf54 exhibits hydrolase activity, acting on ester bonds and zinc ion binding activity.
562	WBGene00016048	C24B9.3	Extracellular material	Extracellular material: matrix	Extracellular material: matrix	C24B9.3 is enriched in the intestine based on tiling array and RNA-seq studies\; is affected by several genes including daf-16, daf-12, and let-7 based on microarray, proteomic, and RNA-seq studies\; is affected by eleven chemicals including methylmercuric chloride, stavudine, and Cry5B based on microarray and RNA-seq studies\; is predicted to encode a protein with the following domains: von Willebrand factor, type A and von Willebrand factor A-like domain superfamily.
563	WBGene00016052	C24B9.9	Transmembrane protein	Transmembrane protein: unassigned	Transmembrane protein: unassigned	dod-3 is an ortholog of human DEPDC4 and DEPDC7.
564	WBGene00016106	C25F6.7	Cytoskeleton	Cytoskeleton: unassigned	Cytoskeleton: unassigned	C25F6.7 is an ortholog of human ITM2A (integral membrane protein 2A), ITM2B (integral membrane protein 2B), and ITM2C (integral membrane

						protein 2C)\; human orthologs of this gene are implicated in ITM2B-related cerebral amyloid angiopathy 1 and ITM2B-related cerebral amyloid angiopathy 2\; human ITM2C exhibits amyloid-beta binding activity.
565	WBGene00016132	C26B9.2	Unassigned	Unassigned	Unassigned	C26B9.2 is enriched in the hypodermis, the muscle cell, and the nervous system based on tiling array, RNA-seq, and microarray studies\; is affected by several genes including daf-16, let-60, and daf-12 based on tiling array, microarray, and RNA-seq studies\; is affected by eight chemicals including methylmercury hydroxide, multi-walled carbon nanotube, and stavudine based on RNA-seq and microarray studies.
566	WBGene00016133	C26B9.3	Unassigned	Unassigned	Unassigned: regulated by multiple stresses	C26B9.3 is enriched in the PLM, the germ line, and the hypodermis based on tiling array and RNA-seq studies\; is affected by several genes including daf-16, let-60, and daf-12 based on microarray, tiling array, and RNA-seq studies\; is affected by thirteen chemicals including Ethanol, methylmercury

						hydroxide, and D-glucose based on RNA-seq and microarray studies\; is predicted to encode a protein with the following domain: GM2-AP, lipid-recognition domain superfamily.
567	WBGene00016152	C27A2.4	Lysosome	Lysosome: acid phosphatase	Lysosome: acid phosphatase	pho-12 is an ortholog of human ACP2 (acid phosphatase 2, lysosomal), ACP4 (acid phosphatase 4), and ACPP (acid phosphatase, prostate)\; human ACPP exhibits phosphatase activity and protein homodimerization activity.
568	WBGene00016201	C28H8.11	Metabolism	Metabolism: amino acid	Metabolism: amino acid: breakdown	tdo-2 is an ortholog of human TDO2\; is predicted to have heme binding activity, metal ion binding activity, and tryptophan 2,3-dioxygenase activity\; is expressed in the body wall musculature and the hypodermis.
569	WBGene00016210	C29F5.1	Unassigned	Unassigned	Unassigned	C29F5.1 localizes to the sarcoplasmic reticulum\; is expressed in the anal depressor muscle, the anal sphincter muscle, the body wall musculature, and the vulval muscle.
570	WBGene00016217	C29F9.2	Unassigned	Unassigned	Unassigned	C29F9.2 is enriched in the ASER, the intestine, and the male based on tiling array, RNA-seq, and

						microarray studies\; is affected by several genes including daf-2, elt-2, and pmk-1 based on tiling array, microarray, and RNA-seq studies\; is affected by nine chemicals including hydrogen sulfide, methylmercury hydroxide, and procyanidin based on microarray and RNA-seq studies.
571	WBGene00016247	C30B5.6	Unassigned	Unassigned	Unassigned: regulated by multiple stresses	C30B5.6 is enriched in the coelomocyte, the excretory cell, and the nervous system based on tiling array and microarray studies\; is affected by several genes including daf-16, daf-2, and daf-12 based on microarray and RNA-seq studies\; is affected by eight chemicals including methylmercury hydroxide, Tunicamycin, and D-glucose based on RNA-seq and microarray studies\; is predicted to encode a protein with the following domain: Uncharacterised protein family, transmembrane-17.
572	WBGene00016266	C30F12.7	Metabolism	Metabolism: mitochondria	Metabolism: mitochondria: citric acid cycle	idhg-2 is an ortholog of human IDH3G\; is predicted to have NAD binding activity, isocitrate dehydrogenase (NAD+) activity, and magnesium

						ion binding activity.
573	WBGene00016274	C30G12.2	Metabolism	Metabolism: short chain dehydrogenase	Metabolism: short chain dehydrogenase	C30G12.2 is enriched in the intestine based on SAGE, tiling array, RNA-seq, and microarray studies\; is affected by several genes including daf-2, glp-1, and daf-12 based on proteomic, microarray, tiling array, and RNA-seq studies\; is affected by twenty-two chemicals including Ethanol, tryptophan, and 1-methylnicotinamide based on RNA-seq and microarray studies\; is predicted to encode a protein with the following domains: Short-chain dehydrogenase\/reductase SDR and NAD(P)-binding domain superfamily.
574	WBGene00016329	C32E12.3	Stress response	Stress response: osmotic	Stress response: osmotic	osr-1 is expressed in the hypodermis and the intestine.
575	WBGene00016351	C33F10.1	Unassigned	Unassigned	Unassigned: regulated by multiple stresses	C33F10.1 is enriched in the amphid sheath cell, the body wall muscle cell, and the male based on tiling array and RNA-seq studies\; is affected by several genes including daf-2, daf-12, and dpy-10 based on microarray and RNA-seq studies\; is affected by twenty chemicals including Heme, Ethanol, and

						methylmercury hydroxide based on microarray and RNA-seq studies.
576	WBGene00016358	C33F10.12	Transmembrane transport	Transmembrane transport: mitochondrial	Transmembrane transport: mitochondrial: solute carrier	C33F10.12 is an ortholog of human SLC25A3 (solute carrier family 25 member 3)\; is predicted to encode a protein with the following domains: Mitochondrial substrate\/solute carrier and Mitochondrial carrier domain superfamily.
577	WBGene00016369	C33G8.13	Transmembrane protein	Transmembrane protein: unassigned	Transmembrane protein: unassigned	C33G8.13 is affected by several genes including daf-16, daf-12, and prg-1 based on tiling array and RNA-seq studies\; is affected by methylmercuric chloride based on microarray studies\; is predicted to encode a protein with the following domain: Methyltransferase FkbM.
578	WBGene00016403	C34D4.11	Unassigned	Unassigned	Unassigned: regulated by multiple stresses	grsp-3 is enriched in the alimentary system, the digestive tract, the hypodermis, the muscular system, and the nervous system based on Chronogram, tiling array, RNA-seq, and microarray studies\; is affected by several genes including daf-16, daf-2, and daf-12 based on microarray, tiling array, and RNA-seq studies\; is affected by eight

						chemicals including Rotenone, manganese chloride, and D-glucose based on RNA-seq and microarray studies.
579	WBGene00016416	C34F11.5	Signaling	Signaling: Y kinase	Signaling: Y kinase	C34F11.5 is an ortholog of human CSK (C-terminal Src kinase) and MATK (megakaryocyte-associated tyrosine kinase)\; is predicted to have ATP binding activity and non-membrane spanning protein tyrosine kinase activity.
580	WBGene00016440	C35D10.2	Signaling	Signaling: unassigned	Signaling: unassigned	gipc-1 is an ortholog of human GIPC1 (GIPC PDZ domain containing 1), GIPC2 (GIPC PDZ domain containing 2), and GIPC3\; is expressed in the intestine\; is used to study Parkinson's disease\; human orthologs of this gene are implicated in autosomal recessive nonsyndromic deafness 15\; human GIPC1 exhibits signaling receptor binding activity.
581	WBGene00016462	C35E7.10	Signaling	Signaling: Y kinase	Signaling: Y kinase	C35E7.10 is an ortholog of human FER (FER tyrosine kinase)\; is predicted to have ATP binding activity and non-membrane spanning protein tyrosine kinase activity.

582	WBGene00016523	C39B5.5	Proteolysis general	Proteolysis general: inhibitor	Proteolysis general: inhibitor: cysteine	C39B5.5 is predicted to have cysteine-type endopeptidase inhibitor activity.
583	WBGene00016531	C39D10.3	Metabolism	Metabolism: lipid	Metabolism: lipid: phospholipid	pcyt-2.2 is an ortholog of human PCYT2\; is predicted to have nucleotidyltransferase activity\; is expressed in the body wall musculature.
584	WBGene00016535	C39D10.8	Unassigned	Unassigned	Unassigned: regulated by multiple stresses	C39D10.8 is enriched in the ASER, the OLL, the PVD, the hypodermis, and the intestine based on microarray, tiling array, RNA-seq, and proteomic studies\; is affected by several genes including daf-16, daf-2, and glp-1 based on microarray and RNA-seq studies\; is affected by five chemicals including Ethanol, manganese chloride, and D-glucose based on RNA-seq and microarray studies\; is predicted to encode a protein with the following domains: Domain of unknown function DUF4440 and NTF2-like domain superfamily.
585	WBGene00016595	C42D4.2	Metabolism	Metabolism: unassigned	Metabolism: unassigned	C42D4.2 is enriched in the intestine based on RNA-seq studies\; is affected by several genes

						including daf-2, dpy-10, and hsf-1 based on microarray, tiling array, and RNA-seq studies\; is affected by ten chemicals including methylmercuric chloride, Tunicamycin, and manganese chloride based on microarray and RNA-seq studies\; is predicted to encode a protein with the following domains: Carboxylesterase, type B and Alpha\/Beta hydrolase fold.
586	WBGene00016596	C42D4.3	Unassigned	Unassigned	Unassigned	C42D4.3 is expressed in the hypodermis, the spermatheca, and the vulva\; is predicted to encode a protein with the following domain: Domain of unknown function DB.
587	WBGene00016598	C42D4.13	Unassigned	Unassigned	Unassigned	C42D4.13 is enriched in the NSM, the germline precursor cell, the hypodermis, and the pharyngeal muscle cell based on tiling array and RNA-seq studies\; is affected by several genes including daf-16, age-1, and daf-12 based on microarray, RNA-seq, and tiling array studies\; is affected by eleven chemicals including methylmercury hydroxide, 1-methylnicotinamide, and stavudine based on RNA-

						seq and microarray studies.
588	WBGene00016658	C45B2.1	Unassigned	Unassigned	Unassigned: regulated by multiple stresses	C45B2.1 is enriched in the epithelial system, the excretory cell, the germ line, the intestine, and the nervous system based on tiling array and RNA-seq studies\; is affected by several genes including daf-2, glp-1, and age-1 based on microarray, RNA-seq, and tiling array studies\; is affected by twenty-two chemicals including Ethanol, Rotenone, and Rifampin based on RNA-seq and microarray studies.
589	WBGene00016659	C45B2.2	Unassigned	Unassigned	Unassigned: regulated by multiple stresses	C45B2.2 is enriched in several tissues, including the alimentary system, the germline precursor cell, the hypodermis, and the nervous system based on tiling array, RNA-seq, and microarray studies\; is affected by several genes including glp-1, skn-1, and let-7 based on microarray, RNA-seq, and proteomic studies\; is affected by seventeen chemicals including 1-methylnicotinamide, Tunicamycin, and D-glucose based on RNA-seq and microarray studies.

590	WBGene00016662	C45B2.8	Unassigned	Unassigned	Unassigned: regulated by multiple stresses	C45B2.8 is enriched in GABAergic neurons, the AFD, the excretory cell, and the hypodermis based on tiling array and RNA-seq studies\; is affected by several genes including daf-16, age-1, and daf-12 based on microarray, tiling array, and RNA-seq studies\; is affected by eleven chemicals including methylmercury hydroxide, multi-walled carbon nanotube, and Sodium Chloride based on RNA-seq and microarray studies.
591	WBGene00016664	C45E5.1	Metabolism	Metabolism: unassigned	Metabolism: unassigned	C45E5.1 is an ortholog of human PDXP (pyridoxal phosphatase) and PGP\; is predicted to have phosphatase activity.
592	WBGene00016675	C45G9.4	Unassigned	Unassigned	Unassigned: regulated by multiple stresses	C45G9.4 is enriched in the germ line and the male based on RNA-seq studies\; is affected by several genes including daf-2, eat-2, and sir-2.1 based on microarray and RNA-seq studies\; is affected by six chemicals including Ethanol, methylmercuric chloride, and resveratrol based on RNA-seq and microarray studies.
593	WBGene0001668	C45G9.9	Unassigned	Unassigned	Unassigned:	C45G9.9 is enriched in the germ line and the male

	0				regulated by multiple stresses	based on RNA-seq studies\; is affected by several genes including daf-2, skn-1, and lin-4 based on microarray and RNA-seq studies\; is affected by thirteen chemicals including Ethanol, methylmercuric chloride, and manganese chloride based on RNA-seq and microarray studies.
594	WBGene00016698	C46A5.1	Signaling	Signaling: phosphatase	Signaling: phosphatase: Y	C46A5.1 is predicted to have protein tyrosine phosphatase activity.
595	WBGene00016733	C46H11.9	Unassigned	Unassigned	Unassigned	phat-2 is involved in pharyngeal gland morphogenesis\; is expressed in the g1AL, the g1AR, the g1P, the g2L, and the g2R.
596	WBGene00016848	C50F7.10	Metabolism	Metabolism: carbohydrate	Metabolism: carbohydrate	klo-1 is an ortholog of human LCTL (lactase like)\; is predicted to have hydrolase activity, hydrolyzing O-glycosyl compounds\; is expressed in the excretory cell and the intestine.
597	WBGene00016870	C52B9.11	Transcription factor	Transcription factor: unassigned	Transcription factor: unassigned	ets-10 is enriched in dopaminergic neurons, the OLL, and the PVD based on tiling array studies\; is affected by several genes including elt-2, pgl-1, and bar-1 based on microarray and RNA-seq studies\; is affected by D-glucose based on RNA-seq studies\;

						is predicted to encode a protein with the following domain: Winged helix DNA-binding domain superfamily.
598	WBGene00016915	C54D2.1	Unassigned	Unassigned	Unassigned: regulated by multiple stresses	C54D2.1 is enriched in GABAergic neurons and the hypodermis based on microarray and RNA-seq studies\; is affected by several genes including skn-1, hsf-1, and clk-1 based on microarray, tiling array, and RNA-seq studies\; is affected by fourteen chemicals including Ethanol, methylmercury hydroxide, and manganese chloride based on RNA-seq and microarray studies\; is predicted to encode a protein with the following domain: EB domain.
599	WBGene00016918	C54E4.2	Unassigned	Unassigned	Unassigned	test-1 is an ortholog of human SPOCK1 (SPARC (osteonectin), cwcv and kazal like domains proteoglycan 1), SPOCK2 (SPARC (osteonectin), cwcv and kazal like domains proteoglycan 2), and SPOCK3 (SPARC (osteonectin), cwcv and kazal like domains proteoglycan 3)\; is predicted to have calcium ion binding activity.
600	WBGene0001692	C54F6.3	Extracellular	Extracellular	Extracellular	C54F6.3 is predicted to have chondroitin 4-

	1		material	material: chondroitin sulfotransferase	material: chondroitin sulfotransferase	sulfotransferase activity.
601	WBGene00016942	C55B7.3	Signaling	Signaling: phosphatase	Signaling: phosphatase: Y	C55B7.3 is predicted to have protein tyrosine phosphatase activity.
602	WBGene00017023	D1022.4	Unassigned	Unassigned	Unassigned: hydrolase	D1022.4 is predicted to have hydrolase activity.
603	WBGene00017052	D2024.4	Unassigned	Unassigned	Unassigned: regulated by multiple stresses	D2024.4 is enriched in the nervous system and the pharynx based on microarray studies\; is affected by several genes including daf-16, skn-1, and daf-12 based on microarray and RNA-seq studies\; is affected by ten chemicals including 1-methylnicotinamide, multi-walled carbon nanotube, and stavudine based on RNA-seq and microarray studies.
604	WBGene00017065	D2092.4	Transmembrane protein	Transmembrane protein: ER	Transmembrane protein: ER	D2092.4 localizes to the myofibril.
605	WBGene00017089	E01A2.7	Stress response	Stress response: unassigned	Stress response: unassigned	poml-2 is an ortholog of human PON1 (paraoxonase 1), PON2 (paraoxonase 2), and PON3 (paraoxonase 3)\; is predicted to have arylesterase

						activity.
606	WBGene00017092	E02C12.6	Unassigned	Unassigned	Unassigned: regulated by multiple stresses	E02C12.6 is enriched in the OLL, the PVD, and the intestine based on tiling array, RNA-seq, and proteomic studies\; is affected by several genes including skn-1, hsf-1, and clk-1 based on microarray and RNA-seq studies\; is affected by eight chemicals including tryptophan, bisphenol A, and bisphenol S based on microarray and RNA-seq studies\; is predicted to encode a protein with the following domains: Protein kinase-like domain superfamily, Uncharacterised oxidoreductase Dhs-27, and CHK kinase-like.
607	WBGene00017103	E02H9.5	Metabolism	Metabolism: carbohydrate	Metabolism: carbohydrate	klo-2 is an ortholog of human LCTL (lactase like)\; is predicted to have hydrolase activity, hydrolyzing O-glycosyl compounds\; is expressed in the hypodermis and the intestine.
608	WBGene00017112	E03H12.5	Unassigned	Unassigned	Unassigned: regulated by multiple stresses	E03H12.5 is enriched in the AVE, the germ line, and the male based on proteomic, tiling array, and RNA-seq studies\; is affected by several genes including daf-16, daf-2, and age-1 based on

						microarray, RNA-seq, and tiling array studies\; is affected by seventeen chemicals including Ethanol, methylmercury hydroxide, and nicotinic acid based on RNA-seq and microarray studies.
609	WBGene00017123	E04F6.3	Metabolism	Metabolism: lipid	Metabolism: lipid: beta oxidation	maoc-1 is an ortholog of human HSD17B4 (hydroxysteroid 17-beta dehydrogenase 4)\; exhibits identical protein binding activity\; is involved in defense response to Gram-negative bacterium and innate immune response\; is used to study obesity\; human orthologs of this gene are implicated in D-bifunctional protein deficiency and Perrault syndrome.
610	WBGene00017128	E04F6.9	Unassigned	Unassigned	Unassigned: regulated by multiple stresses	E04F6.9 is enriched in the epithelial system, the muscle cell, the nervous system, and the sensillum based on tiling array, RNA-seq, and microarray studies\; is affected by several genes including daf-16, daf-2, and glp-1 based on microarray, proteomic, RNA-seq, and tiling array studies\; is affected by seven chemicals including Rotenone, nicotine, and Zidovudine based on RNA-seq,

						proteomic, and microarray studies.
611	WBGene00017131	E04F6.15	Unassigned	Unassigned	Unassigned: regulated by multiple stresses	E04F6.15 is an ortholog of human WWOX (WW domain containing oxidoreductase)\; is predicted to encode a protein with the following domains: Short-chain dehydrogenase\/reductase SDR and NAD(P)-binding domain superfamily.
612	WBGene00017216	F07F6.2	Transmembrane protein	Transmembrane protein: unassigned	Transmembrane protein: unassigned	F07F6.2 is enriched in the AVA and the NSM based on tiling array studies\; is affected by several genes including daf-12, hsf-1, and sir-2.1 based on RNA-seq, tiling array, and microarray studies\; is affected by five chemicals including Rotenone, Tunicamycin, and paraquat based on RNA-seq and microarray studies.
613	WBGene00017218	F07F6.5	Stress response	Stress response: pathogen	Stress response: pathogen: unassigned	dct-5 is enriched in the hypodermis based on tiling array and RNA-seq studies\; is affected by several genes including daf-16, daf-2, and daf-12 based on microarray, tiling array, and RNA-seq studies\; is affected by ten chemicals including Heme, 1-methylnicotinamide, and nicotinic acid based on microarray and RNA-seq studies\; is predicted to

						encode a protein with the following domain: Protein of unknown function DUF5332.
614	WBGene00017307	F09F9.2	Unassigned	Unassigned	Unassigned: regulated by multiple stresses	F09F9.2 is enriched in the germline precursor cell, the hypodermis, the intestine, and the nervous system based on microarray, tiling array, and RNA-seq studies\; is affected by several genes including daf-16, age-1, and daf-12 based on microarray, tiling array, and RNA-seq studies\; is affected by fifteen chemicals including methylmercury hydroxide, 1-methylnicotinamide, and manganese chloride based on RNA-seq and microarray studies.
615	WBGene00017336	F10D2.11	Stress response	Stress response: detoxification	Stress response: detoxification: ugt	ugt-41 is an ortholog of human UGT3A1 (UDP glycosyltransferase family 3 member A1) and UGT3A2 (UDP glycosyltransferase family 3 member A2)\; is predicted to have transferase activity, transferring hexosyl groups.
616	WBGene00017349	F10E7.8	Cytoskeleton	Cytoskeleton: unassigned	Cytoskeleton: unassigned	farl-11 is an ortholog of human STRIP1 (striatin interacting protein 1) and STRIP2 (striatin interacting protein 2)\; is involved in endoplasmic reticulum organization, epithelial tube

						morphogenesis, and positive regulation of endocytic recycling\; localizes to the endoplasmic reticulum\; is expressed in the germ line.
617	WBGene00017364	F10G2.3	Stress response	Stress response: C-type Lectin	Stress response: C-type Lectin	clec-7 is an ortholog of human CLEC3A (C-type lectin domain family 3 member A) and CLEC3B (C-type lectin domain family 3 member B)\; is predicted to have carbohydrate binding activity\; is involved in defense response to Gram-positive bacterium.
618	WBGene00017374	F10G7.11	Unassigned	Unassigned	Unassigned: TransThyretin-Related family domain	ttr-41 is enriched in the intestine and the muscle cell based on tiling array, RNA-seq, and microarray studies\; is affected by several genes including daf-16, daf-2, and glp-1 based on microarray, RNA-seq, and proteomic studies\; is affected by six chemicals including Rotenone, Mianserin, and D-glucose based on RNA-seq and microarray studies\; is predicted to encode a protein with the following domains: Transthyretin-like and Transthyretin-like superfamily.
619	WBGene0001742	F13B9.2	Unassigned	Unassigned	Unassigned:	F13B9.2 is enriched in the PLM and the

	0				regulated by multiple stresses	hypodermis based on tiling array and RNA-seq studies\; is affected by several genes including daf-16, daf-2, and let-60 based on microarray, tiling array, and RNA-seq studies\; is affected by eight chemicals including methylmercury hydroxide, D-glucose, and sesamin based on RNA-seq and microarray studies.
620	WBGene00017427	F13D11.1	Lysosome	Lysosome: acid phosphatase	Lysosome: acid phosphatase	acp-5 is an ortholog of human ACP2 (acid phosphatase 2, lysosomal), ACP4 (acid phosphatase 4), and ACPP (acid phosphatase, prostate)\; human ACPP exhibits phosphatase activity and protein homodimerization activity.
621	WBGene00017431	F13H6.3	Unassigned	Unassigned	Unassigned: regulated by multiple stresses	F13H6.3 is predicted to have carboxylic ester hydrolase activity.
622	WBGene00017438	F13H8.5	Unassigned	Unassigned	Unassigned	F13H8.5 is enriched in the germline precursor cell and the hypodermis based on tiling array studies\; is affected by several genes including daf-16, daf-2, and glp-1 based on microarray, tiling array, RNA-seq, and proteomic studies\; is affected by twelve

						chemicals including hydrogen sulfide, Ethanol, and 1-methylnicotinamide based on microarray and RNA-seq studies\; is predicted to encode a protein with the following domain: Domain of unknown function DB.
623	WBGene00017560	F18C5.5	Unassigned	Unassigned	Unassigned: regulated by multiple stresses	F18C5.5 is enriched in the OLL, the PVD, the germline precursor cell, and the hypodermis based on microarray, tiling array, and RNA-seq studies\; is affected by several genes including daf-16, daf-12, and lin-4 based on microarray, tiling array, and RNA-seq studies\; is affected by thirteen chemicals including methylmercury hydroxide, methylmercuric chloride, and manganese chloride based on RNA-seq and microarray studies.
624	WBGene00017565	F18E3.7	Metabolism	Metabolism: amino acid	Metabolism: amino acid: breakdown	ddo-2 is an ortholog of human DDO and dao\; exhibits D-aspartate oxidase activity and D-glutamate oxidase activity\; is involved in oxidation-reduction process\; is expressed in the body wall musculature, the intestinal cell, and the pharyngeal muscle cell.

625	WBGene00017640	F20D6.11	Metabolism	Metabolism: unassigned	Metabolism: unassigned	F20D6.11 is an ortholog of human AIFM3\; is predicted to have 2 iron, 2 sulfur cluster binding activity, flavin adenine dinucleotide binding activity, and oxidoreductase activity\; is expressed in the muscular system, the pharynx, the reproductive system, and the tail.
626	WBGene00017657	F21C10.7	Unassigned	Unassigned	Unassigned: regulated by multiple stresses	F21C10.7 is enriched in the body wall musculature, the germ line, the male, and the muscle cell based on proteomic, RNA-seq, and microarray studies\; is affected by several genes including daf-2, dpy-10, and sir-2.1 based on microarray and RNA-seq studies\; is affected by twelve chemicals including hydrogen sulfide, methylmercuric chloride, and D-glucose based on microarray and RNA-seq studies\; is predicted to encode a protein with the following domains: Immunoglobulin subtype 2, Immunoglobulin subtype, Immunoglobulin I-set, Immunoglobulin-like fold, and Immunoglobulin-like domain superfamily.
627	WBGene0001765	F21C10.9	Unassigned	Unassigned	Unassigned	F21C10.9 is enriched in the hypodermis, the

	8					intestine, the nervous system, and the sensillum based on tiling array, RNA-seq, and microarray studies\; is affected by several genes including daf-16, daf-2, and daf-12 based on microarray, tiling array, proteomic, and RNA-seq studies\; is affected by twenty-eight chemicals including Nitric Oxide, 1-methylnicotinamide, and methylmercuric chloride based on RNA-seq and microarray studies\; is predicted to encode a protein with the following domain: Acyl-CoA N-acyltransferase.
628	WBGene00017673	F21F3.3	Protein modification	Protein modification: methyltransferase	Protein modification: methyltransferase	icmt-1 is an ortholog of human ICMT\; is predicted to have protein C-terminal S-isoprenylcysteine carboxyl O-methyltransferase activity.
629	WBGene00017678	F21F8.4	Proteolysis general	Proteolysis general: aspartate	Proteolysis general: aspartate: cathepsin	asp-12 is an ortholog of human PGA4, NAPSA, and PGA5\; is predicted to have aspartic-type endopeptidase activity\; is involved in innate immune response.
630	WBGene00017688	F22A3.2	Unassigned	Unassigned	Unassigned: TransThyretin-	ttr-35 is enriched in the intestine and the muscle cell based on RNA-seq and microarray studies\; is

					Related family domain	affected by several genes including daf-2, let-7, and dpy-10 based on proteomic, microarray, and RNA-seq studies\; is affected by seven chemicals including Zidovudine, resveratrol, and Chlorpyrifos based on RNA-seq and microarray studies\; is predicted to encode a protein with the following domains: Transthyretin-like and Transthyretin-like superfamily.
631	WBGene00017716	F22F4.1	Unassigned	Unassigned	Unassigned	sups-1 is enriched in the OLL, the PVD, the germline precursor cell, and the hypodermis based on microarray, tiling array, and RNA-seq studies\; is affected by several genes including daf-16, daf-2, and glp-1 based on microarray and RNA-seq studies\; is affected by fourteen chemicals including Ethanol, methylmercury hydroxide, and 1-methylnicotinamide based on RNA-seq and microarray studies.
632	WBGene00017773	F25B5.1	Unassigned	Unassigned	Unassigned	dct-6 is expressed in several tissues, including the alimentary system, the epithelial system, the muscular system, and the reproductive system\; is

						predicted to encode a protein with the following domains: WD40\/YVTN repeat-like-containing domain superfamily and WD40-repeat-containing domain superfamily.
633	WBGene00017781	F25E2.3	Metabolism	Metabolism: lipid	Metabolism: lipid: beta oxidation	F25E2.3 is an ortholog of human ACOT8 (acyl-CoA thioesterase 8)\; is predicted to have acyl-CoA hydrolase activity.
634	WBGene00017807	F26A1.9	Unassigned	Unassigned	Unassigned	F26A1.9 is enriched in the Z1, the Z4, the germline precursor cell, the hypodermis, and the male based on tiling array and RNA-seq studies\; is affected by several genes including daf-16, daf-2, and glp-1 based on RNA-seq and microarray studies\; is affected by five chemicals including resveratrol, Diazinon, and Colistin based on microarray studies.
635	WBGene00017841	F26G1.5	Unassigned	Unassigned	Unassigned	F26G1.5 is enriched in the germline precursor cell, the hypodermis, the nervous system, and the somatic gonad precursor based on tiling array, RNA-seq, and microarray studies\; is affected by several genes including daf-16, daf-2, and age-1 based on microarray, tiling array, and RNA-seq

						studies\; is affected by thirteen chemicals including 1-methylnicotinamide, Rotenone, and D-glucose based on RNA-seq and microarray studies\; is predicted to encode a protein with the following domain: Domain of unknown function DB.
636	WBGene00017944	F31D5.1	Transmembrane transport	Transmembrane transport: major facilitator	Transmembrane transport: major facilitator	F31D5.1 is an ortholog of human MFSD11\; is predicted to encode a protein with the following domains: Ion channel regulatory protein, UNC-93 and MFS transporter superfamily.
637	WBGene00017962	F31F4.15	Proteolysis proteasome	Proteolysis proteasome: E3	Proteolysis proteasome: E3: F box	fbxa-72 is enriched in several tissues, including the Z1.p, the Z4.a, the hypodermis, the male distal tip cell, and the somatic gonad precursor based on tiling array, RNA-seq, proteomic, and microarray studies\; is affected by several genes including daf-2, daf-12, and eat-2 based on microarray and RNA-seq studies\; is affected by eleven chemicals including Tunicamycin, sodium arsenite, and indole based on microarray and RNA-seq studies\; is predicted to encode a protein with the following domain: Domain of unknown function DUF38,

						Caenorhabditis species.
638	WBGene00017968	F32A5.2	Unassigned	Unassigned	Unassigned	skpo-3 is an ortholog of human PXDN (peroxidasin), LPO, and MPO\; is predicted to have heme binding activity and peroxidase activity.
639	WBGene00017991	F32E10.3	Stress response	Stress response: C-type Lectin	Stress response: C-type Lectin	clec-180 is predicted to have carbohydrate binding activity.
640	WBGene00017998	F33D4.6	Unassigned	Unassigned	Unassigned: regulated by multiple stresses	F33D4.6 is enriched in the germline precursor cell, the hypodermis, the intestine, and the nervous system based on microarray, tiling array, and RNA-seq studies\; is affected by several genes including daf-16, skn-1, and daf-12 based on microarray, proteomic, and RNA-seq studies\; is affected by eighteen chemicals including Ethanol, Nitric Oxide, and methylmercury hydroxide based on RNA-seq and microarray studies.
641	WBGene00018031	F35B3.4	Unassigned	Unassigned	Unassigned: regulated by multiple stresses	F35B3.4 is enriched in the DA neuron, the hypodermis, and the intestine based on tiling array, RNA-seq, and microarray studies\; is affected by several genes including daf-16, daf-2, and glp-1 based on microarray, tiling array, and RNA-seq

						studies\; is affected by eighteen chemicals including Heme, hydrogen sulfide, and Nitric Oxide based on microarray and RNA-seq studies\; is predicted to encode a protein with the following domain: Domain of unknown function DB.
642	WBGene00018055	F35F10.5	Unassigned	Unassigned	Unassigned: regulated by multiple stresses	F35F10.5 is enriched in the ASER, the hypodermis, and the muscle cell based on tiling array, RNA-seq, and microarray studies\; is affected by several genes including daf-16, daf-2, and glp-1 based on RNA-seq and microarray studies\; is affected by six chemicals including Ethanol, Tunicamycin, and D-glucose based on RNA-seq and microarray studies.
643	WBGene00018073	F35H10.10	Signaling	Signaling: heteromeric G protein	Signaling: heteromeric G protein: receptor	F35H10.10 is predicted to have G protein-coupled receptor activity.
644	WBGene00018113	F36H9.5	Transmembrane protein	Transmembrane protein: unassigned	Transmembrane protein: unassigned	F36H9.5 is an ortholog of human PGAP2.
645	WBGene00018134	F37A4.4	Unassigned	Unassigned	Unassigned: regulated by	F37A4.4 is enriched in the germ line, the hypodermis, the male, and the muscle cell based on

					multiple stresses	RNA-seq and microarray studies\; is affected by several genes including daf-2, dpy-10, and clk-1 based on microarray, RNA-seq, and tiling array studies\; is affected by eighteen chemicals including hydrogen sulfide, Ethanol, and methylmercuric chloride based on microarray and RNA-seq studies\; is predicted to encode a protein with the following domains: BRCT domain, Ankyrin repeat, Domain of unknown function WSN, BRCT domain superfamily, and Ankyrin repeat-containing domain superfamily.
646	WBGene00018135	F37A4.5	Proteolysis proteasome	Proteolysis proteasome: 19S	Proteolysis proteasome: 19S	F37A4.5 is an ortholog of human PSMD14 (proteasome 26S subunit, non-ATPase 14)\; is predicted to have metal ion binding activity and metallopeptidase activity.
647	WBGene00018166	F38A5.9	Unassigned	Unassigned	Unassigned	nspb-5 is affected by several genes including clk-1, sir-2.1, and npr-1 based on quantitative PCR, microarray, and tiling array studies\; is affected by Colistin based on microarray studies\; is predicted to encode a protein with the following domain:

						Protein of unknown function DUF1459.
648	WBGene00018167	F38A5.10	Unassigned	Unassigned	Unassigned: regulated by multiple stresses	nspb-4 is enriched in the germ line and the pharynx based on RNA-seq studies\; is affected by several genes including sir-2.1, lin-35, and npr-1 based on microarray, quantitative PCR, and RNA-seq studies\; is affected by four chemicals including hydrogen sulfide, D-glucose, and Chlorpyrifos based on microarray and RNA-seq studies\; is predicted to encode a protein with the following domain: Protein of unknown function DUF1459.
649	WBGene00018221	F40A3.6	Unassigned	Unassigned	Unassigned: regulated by multiple stresses	F40A3.6 is enriched in the PLM and the muscle cell based on RNA-seq, proteomic, and microarray studies\; is affected by several genes including glp-1, let-7, and dpy-10 based on microarray, RNA-seq, and proteomic studies\; is affected by six chemicals including methylmercuric chloride, Rotenone, and Cadmium based on microarray and RNA-seq studies.
650	WBGene00018280	F41C6.7	Transmembrane transport	Transmembrane transport: ion	Transmembrane transport: ion	F41C6.7 is predicted to have cation transmembrane transporter activity.

651	WBGene00018292	F41E6.11	Stress response	Stress response: ER	Stress response: ER	F41E6.11 is involved in endoplasmic reticulum unfolded protein response.
652	WBGene00018293	F41E6.12	Unassigned	Unassigned	Unassigned	F41E6.12 is enriched in the OLL, the PVD, the germline precursor cell, and the hypodermis based on microarray and tiling array studies\; is affected by several genes including daf-2, daf-12, and dpy-10 based on microarray, tiling array, and RNA-seq studies\; is affected by ten chemicals including Rotenone, Tunicamycin, and D-glucopyranose based on RNA-seq and microarray studies.
653	WBGene00018334	F42A9.5	Stress response	Stress response: detoxification	Stress response: detoxification: CYP	cyp-33E2 is an ortholog of human CYP2J2 (cytochrome P450 family 2 subfamily J member 2), CYP2R1 (cytochrome P450 family 2 subfamily R member 1), and CYP2U1 (cytochrome P450 family 2 subfamily U member 1)\; exhibits monooxygenase activity\; is involved in long-chain fatty acid metabolic process and regulation of pharyngeal pumping\; localizes to the intracellular membrane-bounded organelle\; is expressed in the interfacial epithelial cell and the pharynx\; human

						orthologs of this gene are implicated in hereditary spastic paraplegia 56, lung cancer, and rickets.
654	WBGene00018336	F42A9.7	Unassigned	Unassigned	Unassigned: regulated by multiple stresses	F42A9.7 is enriched in the germ line and the male based on proteomic and RNA-seq studies\; is affected by several genes including age-1, hsf-1, and eat-2 based on microarray, RNA-seq, and tiling array studies\; is affected by thirteen chemicals including Ethanol, methylmercuric chloride, and manganese chloride based on RNA-seq and microarray studies\; is predicted to encode a protein with the following domains: Major sperm protein (MSP) domain, PapD-like superfamily, and Immunoglobulin-like fold.
655	WBGene00018340	F42A10.3	Protein modification	Protein modification: methyltransferase	Protein modification: methyltransferase	anmt-2 is an ortholog of human INMT (indolethylamine N-methyltransferase) and PNMT (phenylethanolamine N-methyltransferase)\; is predicted to have methyltransferase activity\; is expressed in several tissues, including the ABalpaaapa, the ABalpaapap, the ABaraapapa, the MSaaaapp, and the alimentary system.

656	WBGene00018342	F42A10.6	Proteolysis proteasome	Proteolysis proteasome: E3	Proteolysis proteasome: E3: F box	F42A10.6 is expressed in the intestine.
657	WBGene00018359	F42G8.8	Signaling	Signaling: phosphatase	Signaling: phosphatase: unassigned	F42G8.8 is an ortholog of human PPP3CA (protein phosphatase 3 catalytic subunit alpha), PPP3CB (protein phosphatase 3 catalytic subunit beta), and PPP3CC (protein phosphatase 3 catalytic subunit gamma)\; is predicted to have phosphoprotein phosphatase activity.
658	WBGene00018393	F43E2.5	Metabolism	Metabolism: 1CC	Metabolism: 1CC: unassigned	msra-1 is an ortholog of human MSRA\; exhibits L-methionine:thioredoxin-disulfide S-oxidoreductase activity and peptide-methionine (S)-S-oxide reductase activity\; is involved in oxidation-reduction process\; is expressed in the hypodermis and the nervous system\; is used to study inclusion body myositis.
659	WBGene00018407	F44B9.2	Unassigned	Unassigned	Unassigned	F44B9.2 is expressed in the muscular system and the reproductive system.
660	WBGene00018487	F46C8.8	Unassigned	Unassigned	Unassigned: regulated by	F46C8.8 is enriched in several tissues, including the germline precursor cell, the hypodermis, the

					multiple stresses	intestine, the nervous system, and the somatic gonad precursor based on microarray, tiling array, and RNA-seq studies\; is affected by several genes including daf-16, daf-2, and daf-12 based on microarray, tiling array, and RNA-seq studies\; is affected by thirteen chemicals including hydrogen sulfide, Ethanol, and 1-methylnicotinamide based on microarray and RNA-seq studies.
661	WBGene00018504	F46F5.13	Unassigned	Unassigned	Unassigned	F46F5.13 is an ortholog of human VMO1\; is predicted to encode a protein with the following domains: Vitelline membrane outer layer protein I (VOMI) and Vitelline membrane outer layer protein I (VOMI) superfamily.
662	WBGene00018507	F46F5.16	Unassigned	Unassigned	Unassigned: membrane spanning domain	F46F5.16 is affected by several genes including daf-2, sir-2.1, and hpl-2 based on microarray, tiling array, and RNA-seq studies\; is affected by multi-walled carbon nanotube and D-glucose based on RNA-seq studies.
663	WBGene00018548	F47C12.4	Stress response	Stress response: C-type Lectin	Stress response: C-type Lectin	clec-79 is predicted to have carbohydrate binding activity.

664	WBGene00018569	F47F2.1	Muscle function	Muscle function	Muscle function	F47F2.1 is an ortholog of human PRKX (protein kinase X-linked)\; is predicted to have ATP binding activity and cAMP-dependent protein kinase activity\; localizes to the cytoplasm.
665	WBGene00018572	F47F6.1	Development	Development: somatic	Development: somatic	lin-42 is an ortholog of human PER1, PER2, and PER3\; exhibits RNA polymerase II transcription factor binding activity\; is involved in negative regulation of dauer larval development, negative regulation of pri-miRNA transcription by RNA polymerase II, and regulation of development, heterochronic\; localizes to the cytoplasm and nucleus\; is expressed in several tissues, including the H1L.app, the H1R.app, the H2L.ppp, the alimentary system, and the hypodermal cell\; human orthologs of this gene are implicated in advanced sleep phase syndrome 1.
666	WBGene00018645	F49F1.5	Development	Development: apoptosis	Development: apoptosis	F49F1.5 is enriched in the OLL, the PVD, and the intestine based on tiling array, RNA-seq, and microarray studies\; is affected by several genes including daf-16, skn-1, and daf-12 based on

						microarray, tiling array, and RNA-seq studies\; is affected by seventeen chemicals including Heme, hydrogen sulfide, and Tunicamycin based on microarray and RNA-seq studies\; is predicted to encode a protein with the following domain: ShKT domain.
667	WBGene00018656	F49H12.5	Unassigned	Unassigned	Unassigned: regulated by multiple stresses	txdc-12.2 is an ortholog of human AGR2 (anterior gradient 2, protein disulphide isomerase family member), AGR3 (anterior gradient 3, protein disulphide isomerase family member), and TXNDC12 (thioredoxin domain containing 12)\; human AGR2 exhibits dystroglycan binding activity, epidermal growth factor receptor binding activity, and protein homodimerization activity.
668	WBGene00018682	F52D1.1	Metabolism	Metabolism: carbohydrate	Metabolism: carbohydrate	aagr-4 is an ortholog of human GANAB (glucosidase II alpha subunit) and GANC (glucosidase alpha, neutral C)\; is predicted to have carbohydrate binding activity and hydrolase activity, hydrolyzing O-glycosyl compounds.
669	WBGene0001870	F52F10.2	Transmembran	Transmembrane	Transmembrane	F52F10.2 is an ortholog of human MFSD8\; human

	6		e transport	transport: major facilitator	transport: major facilitator	orthologs of this gene are implicated in neuronal ceroid lipofuscinosis 7\; is predicted to encode a protein with the following domains: Major facilitator superfamily and MFS transporter superfamily.
670	WBGene00018737	F53B1.4	Metabolism	Metabolism: unassigned	Metabolism: unassigned	F53B1.4 is an ortholog of human TGDS (TDP-glucose 4,6-dehydratase)\; is involved in endoplasmic reticulum unfolded protein response.
671	WBGene00018760	F53E10.4	Stress response	Stress response: pathogen	Stress response: pathogen: unassigned	irg-3 is involved in innate immune response.
672	WBGene00018877	F55D10.1	Trafficking	Trafficking: ER/Golgi	Trafficking: ER/Golgi: mannosidase	aman-1 is an ortholog of human MAN2B1 (mannosidase alpha class 2B member 1)\; is predicted to have alpha-mannosidase activity, carbohydrate binding activity, and metal ion binding activity\; is expressed in the intestine and the nervous system\; human orthologs of this gene are implicated in alpha-mannosidosis.
673	WBGene00018937	F56B6.6	Unassigned	Unassigned	Unassigned	F56B6.6 is expressed in tail neurons and the pharyngeal gland cell\; is predicted to encode a

						protein with the following domain: Cysteine-rich repeat.
674	WBGene00018965	F56D2.3	Unassigned	Unassigned	Unassigned: regulated by multiple stresses	F56D2.3 is enriched in the OLL, the PVD, and the pharynx based on microarray and RNA-seq studies\; is affected by several genes including daf-16, daf-2, and skn-1 based on microarray, tiling array, and RNA-seq studies\; is affected by thirteen chemicals including 1-methylnicotinamide, manganese chloride, and D-glucose based on RNA-seq and microarray studies.
675	WBGene00018969	F56D3.1	Unassigned	Unassigned	Unassigned: regulated by multiple stresses	F56D3.1 is enriched in the OLL, the PVD, the germ line, and the hypodermis based on proteomic, microarray, and tiling array studies\; is affected by several genes including daf-16, daf-2, and glp-1 based on microarray, RNA-seq, and proteomic studies\; is affected by eighteen chemicals including hydrogen sulfide, methylmercury hydroxide, and 1-methylnicotinamide based on microarray and RNA-seq studies.
676	WBGene0001898	F56F10.1	Proteolysis	Proteolysis	Proteolysis	F56F10.1 is an ortholog of human PRSS16 (serine

	4		general	general: serine	general: serine	protease 16)\; is predicted to have serine-type peptidase activity\; is involved in innate immune response\; localizes to the membrane raft.
677	WBGene00018994	F56H1.3	Pseudogene	Pseudogene	Pseudogene	F56H1.3 is enriched in the OLL, the PVD, the germ line, and the male based on microarray and RNA-seq studies\; is affected by several genes including hsf-1, eat-2, and sir-2.1 based on RNA-seq, tiling array, and microarray studies\; is affected by four chemicals including hydrogen sulfide, methylmercuric chloride, and manganese chloride based on microarray and RNA-seq studies\; is predicted to encode a protein with the following domains: BRCT domain, Domain of unknown function WSN, BRCT domain superfamily, and Ankyrin repeat-containing domain superfamily.
678	WBGene00019021	F57H12.6	Unassigned	Unassigned	Unassigned: regulated by multiple stresses	F57H12.6 is enriched in the OLL, the PVD, the hypodermis, the intestine, and the male based on microarray and tiling array studies\; is affected by several genes including daf-16, daf-2, and glp-1 based on proteomic, microarray, RNA-seq, and

						tiling array studies\; is affected by twenty chemicals including Nitric Oxide, methylmercury hydroxide, and 1-methylnicotinamide based on RNA-seq and microarray studies.
679	WBGene00019022	F58A6.1	Metabolism	Metabolism: lipid	Metabolism: lipid: beta oxidation	F58A6.1 is an ortholog of human ECH1\; is predicted to have catalytic activity.
680	WBGene00019060	F58F9.7	Metabolism	Metabolism: lipid	Metabolism: lipid: beta oxidation	acox-3 is an ortholog of human ACOX3\; is predicted to have FAD binding activity and acyl-CoA oxidase activity.
681	WBGene00019111	F59E11.5	Transmembrane protein	Transmembrane protein: unassigned	Transmembrane protein: unassigned	F59E11.5 is enriched in the Z1, the Z4, and the hypodermis based on tiling array and RNA-seq studies\; is affected by several genes including daf-2, glp-1, and dpy-10 based on microarray, RNA-seq, proteomic, and tiling array studies\; is affected by five chemicals including Rotenone, D-glucose, and Cry5B based on RNA-seq and microarray studies\; is predicted to encode a protein with the following domain: Domain of unknown function DUF19.

682	WBGene00019148	H03E18.1	Unassigned	Unassigned	Unassigned: regulated by multiple stresses	H03E18.1 is enriched in the OLL, the PLM, the PVD, and the hypodermis based on microarray, tiling array, and RNA-seq studies\; is affected by several genes including daf-16, daf-2, and skn-1 based on microarray, tiling array, and RNA-seq studies\; is affected by eighteen chemicals including methylmercury hydroxide, 1-methylnicotinamide, and manganese chloride based on RNA-seq and microarray studies\; is predicted to encode a protein with the following domain: PAN\/Apple domain.
683	WBGene00019150	H04J21.1	Unassigned	Unassigned	Unassigned: membrane spanning domain	H04J21.1 is enriched in the male based on RNA-seq studies\; is affected by several genes including daf-2, eat-2, and sir-2.1 based on microarray and RNA-seq studies\; is affected by four chemicals including tryptophan, manganese chloride, and multi-walled carbon nanotube based on microarray and RNA-seq studies\; is predicted to encode a protein with the following domains: Domain of unknown function WSN and Ubiquitin-like domain

						superfamily.
684	WBGene00019152	H04M03.2	Unassigned	Unassigned	Unassigned: regulated by multiple stresses	nspb-6 is affected by several genes including daf-16, skn-1, and daf-12 based on tiling array, RNA-seq, and microarray studies\; is affected by ten chemicals including hydrogen sulfide, manganese chloride, and D-glucose based on microarray and RNA-seq studies\; is predicted to encode a protein with the following domain: Protein of unknown function DUF1459.
685	WBGene00019184	H10E21.4	Unassigned	Unassigned	Unassigned	H10E21.4 is predicted to have calcium ion binding activity.
686	WBGene00019204	H14N18.4	Metabolism	Metabolism: glutathione	Metabolism: glutathione	H14N18.4 is an ortholog of human GGT1 (gamma-glutamyltransferase 1) and GGT2 (gamma-glutamyltransferase 2)\; is predicted to have glutathione hydrolase activity.
687	WBGene00019236	H23N18.5	Signaling	Signaling: lipid	Signaling: lipid: phospholipase A	H23N18.5 is predicted to have phospholipase A2 activity.
688	WBGene00019257	H32C10.3	Transmembrane protein	Transmembrane protein: unassigned	Transmembrane protein: unassigned	dhhc-13 is an ortholog of human ANKRD35 (ankyrin repeat domain 35)\; is predicted to have protein-cysteine S-palmitoyltransferase activity.

689	WBGene00019272	H42K12.3	Unassigned	Unassigned	Unassigned: regulated by multiple stresses	H42K12.3 is enriched in the OLL, the PVD, and the hypodermis based on microarray and tiling array studies\; is affected by several genes including daf-16, daf-2, and daf-12 based on microarray, tiling array, and RNA-seq studies\; is affected by seventeen chemicals including hydrogen sulfide, methylmercury hydroxide, and 1-methylnicotinamide based on microarray and RNA-seq studies\; is predicted to encode a protein with the following domains: PAN\/Apple domain and Thrombospondin type-1 (TSP1) repeat superfamily.
690	WBGene00019309	K02E7.4	Transmembrane transport	Transmembrane transport: potassium channel	Transmembrane transport: potassium channel	K02E7.4 is an ortholog of human KCTD10 (potassium channel tetramerization domain containing 10), KCTD13, and TNFAIP1 (TNF alpha induced protein 1)\; human KCTD13 exhibits GTP-Rho binding activity and identical protein binding activity.
691	WBGene00019368	K03H6.2	Unassigned	Unassigned	Unassigned: regulated by multiple stresses	K03H6.2 is predicted to have hydrolase activity.

692	WBGene00019406	K05F1.3	Metabolism	Metabolism: lipid	Metabolism: lipid: beta oxidation	acdh-8 is an ortholog of human ACADM (acyl-CoA dehydrogenase medium chain)\; is predicted to have flavin adenine dinucleotide binding activity and oxidoreductase activity, acting on the CH-CH group of donors\; human orthologs of this gene are implicated in medium chain acyl-CoA dehydrogenase deficiency.
693	WBGene00019422	K05G3.1	Unassigned	Unassigned	Unassigned	K05G3.1 is affected by smg-2, drh-3, and sir-2.1 based on RNA-seq and microarray studies\; is affected by multi-walled carbon nanotube and Sirolimus based on RNA-seq and microarray studies\; is predicted to encode a protein with the following domain: Ribonuclease H-like superfamily.
694	WBGene00019430	K06A5.2	Transmembrane protein	Transmembrane protein: unassigned	Transmembrane protein: unassigned	K06A5.2 is enriched in the male based on RNA-seq studies\; is affected by several genes including daf-2, age-1, and dpy-10 based on microarray and RNA-seq studies\; is affected by twenty-one chemicals including Ethanol, nicotinic acid, and methylmercuric chloride based on RNA-seq and

						microarray studies\; is predicted to encode a protein with the following domain: Protein of unknown function DUF856, Caenorhabditis species.
695	WBGene00019451	K06H6.3	Transmembrane transport	Transmembrane transport: solute carrier	Transmembrane transport: solute carrier	nstp-7 is an ortholog of human SLC35A2 (solute carrier family 35 member A2)\; is predicted to have pyrimidine nucleotide-sugar transmembrane transporter activity.
696	WBGene00019454	K06H6.6	Pseudogene	Pseudogene	Pseudogene	K06H6.6 is affected by several genes including clk-1, eat-2, and lin-35 based on microarray and tiling array studies\; is affected by Chlorpyrifos based on microarray studies.
697	WBGene00019473	K07C6.5	Stress response	Stress response: detoxification	Stress response: detoxification: CYP	cyp-35A5 is an ortholog of human CYP21A2 (cytochrome P450 family 21 subfamily A member 2) and members of the human CYP2 (Cytochrome P450 family 2) gene family including CYP2R1, CYP2U1, and CYP2J2\; is predicted to have heme binding activity, iron ion binding activity, and oxidoreductase activity\; is involved in response to xenobiotic stimulus\; is expressed in the intestine\; human orthologs of this gene are implicated in

						congenital adrenal hyperplasia.
698	WBGene00019489	K07E1.1	Metabolism	Metabolism: unassigned	Metabolism: unassigned	K07E1.1 is an ortholog of human ADI1\; is predicted to have acireductone dioxygenase [iron(II)-requiring] activity.
699	WBGene00019506	K07H8.5	Transmembrane protein	Transmembrane protein: unassigned	Transmembrane protein: unassigned	K07H8.5 is enriched in the germ line, the male, and the nervous system based on RNA-seq studies\; is affected by several genes including daf-16, daf-2, and dpy-10 based on microarray, RNA-seq, and tiling array studies\; is affected by six chemicals including Ethanol, Tunicamycin, and manganese chloride based on RNA-seq and microarray studies.
700	WBGene00019515	K08B4.3	Stress response	Stress response: detoxification	Stress response: detoxification: ugt	ugt-19 is an ortholog of human UGT3A1 (UDP glycosyltransferase family 3 member A1) and UGT3A2 (UDP glycosyltransferase family 3 member A2)\; is predicted to have transferase activity, transferring hexosyl groups.
701	WBGene00019550	K09C4.5	Transmembrane transport	Transmembrane transport: solute carrier	Transmembrane transport: solute carrier	K09C4.5 is an ortholog of human SLC2A7 (solute carrier family 2 member 7), SLC2A9 (solute carrier family 2 member 9), and SLC2A5 (solute carrier family 2 member 5)\; is predicted to have

						transmembrane transporter activity.
702	WBGene00019563	K09C6.9	Unassigned	Unassigned	Unassigned: regulated by multiple stresses	K09C6.9 is enriched in the amphid sheath cell and the intestine based on RNA-seq and microarray studies\; is affected by several genes including daf-16, daf-2, and daf-12 based on microarray, tiling array, and RNA-seq studies\; is affected by nine chemicals including nicotinic acid, methylmercuric chloride, and Rotenone based on RNA-seq and microarray studies.
703	WBGene00019586	K09F6.3	Signaling	Signaling: phosphatase	Signaling: phosphatase: Y	K09F6.3 is predicted to have protein tyrosine phosphatase activity.
704	WBGene00019587	K09F6.4	Pseudogene	Pseudogene	Pseudogene	K09F6.4 is enriched in the intestine and the male based on RNA-seq studies\; is affected by several genes including daf-2, hsf-1, and clk-1 based on RNA-seq and microarray studies\; is affected by four chemicals including Rotenone, stavudine, and Zidovudine based on RNA-seq and microarray studies\; is predicted to encode a protein with the following domain: Domain of unknown function WSN.

705	WBGene00019601	K09H11.4	Unassigned	Unassigned	Unassigned: regulated by multiple stresses	K09H11.4 is enriched in the amphid sheath cell based on RNA-seq studies\; is affected by several genes including hsf-1, eat-2, and sir-2.1 based on RNA-seq and microarray studies\; is affected by six chemicals including hydrogen sulfide, multi-walled carbon nanotube, and D-glucose based on microarray and RNA-seq studies.
706	WBGene00019619	K10C2.3	Proteolysis general	Proteolysis general: aspartate	Proteolysis general: aspartate: unassigned	asp-14 is an ortholog of human PGA3, PGA5, and CTSE (cathepsin E)\; is predicted to have aspartic-type endopeptidase activity\; is involved in innate immune response.
707	WBGene00019620	K10C2.4	Metabolism	Metabolism: amino acid	Metabolism: amino acid: breakdown	fah-1 is an ortholog of human FAH\; is predicted to have fumarylacetoacetase activity\; is expressed in the hypodermis\; is used to study tyrosinemia type I\; human orthologs of this gene are implicated in tyrosinemia type I.
708	WBGene00019640	K10G6.2	Unassigned	Unassigned	Unassigned: regulated by multiple stresses	dos-2 is enriched in the germline precursor cell based on tiling array studies\; is affected by several genes including daf-16, daf-12, and clk-1 based on microarray and RNA-seq studies\; is affected by

						eight chemicals including methylmercury hydroxide, D-glucose, and Chlorpyrifos based on RNA-seq and microarray studies.
709	WBGene00019653	K11G9.2	Unassigned	Unassigned	Unassigned: hydrolase	K11G9.2 is predicted to have hydrolase activity\; is expressed in several tissues, including the alimentary system, the epithelial system, the nervous system, and the reproductive system.
710	WBGene00019682	K12H4.7	Proteolysis general	Proteolysis general: serine	Proteolysis general: serine	K12H4.7 is an ortholog of human PRSS16 (serine protease 16)\; is predicted to have serine-type peptidase activity\; is involved in innate immune response\; is expressed in the intestine.
711	WBGene00019727	M02D8.1	Unassigned	Unassigned	Unassigned: regulated by multiple stresses	zig-12 localizes to the endoplasmic reticulum, sarcomere, and striated muscle dense body\; is expressed in the body wall musculature and the vulval muscle.
712	WBGene00019761	M03F8.1	Transmembrane protein	Transmembrane protein: unassigned	Transmembrane protein: unassigned	M03F8.1 is enriched in the germline precursor cell and the hypodermis based on tiling array studies\; is affected by several genes including daf-16, daf-12, and lin-4 based on tiling array, RNA-seq, and microarray studies\; is affected by fifteen chemicals

						including aldicarb, Ethanol, and methylmercury hydroxide based on microarray and RNA-seq studies.
713	WBGene00019783	M70.1	Transmembrane protein	Transmembrane protein: unassigned	Transmembrane protein: unassigned	M70.1 is enriched in the germ line and the male based on proteomic and RNA-seq studies\; is affected by several genes including daf-2, eat-2, and npr-1 based on microarray, RNA-seq, and tiling array studies\; is affected by eleven chemicals including hydrogen sulfide, methylmercuric chloride, and manganese chloride based on microarray and RNA-seq studies\; is predicted to encode a protein with the following domain: Domain of unknown function WSN.
714	WBGene00019803	PDB1.1	Stress response	Stress response: pathogen	Stress response: pathogen: unassigned	PDB1.1 is predicted to have cation transmembrane transporter activity\; is involved in innate immune response.
715	WBGene00019839	R02F11.1	Unassigned	Unassigned	Unassigned: regulated by multiple stresses	R02F11.1 is enriched in the ALM, the OLL, the PLM, the PVD, and the pharynx based on microarray and tiling array studies\; is affected by several genes including daf-16, skn-1, and daf-12

						based on microarray and RNA-seq studies\; is affected by fourteen chemicals including methylmercury hydroxide, 1-methylnicotinamide, and D-glucose based on RNA-seq and microarray studies.
716	WBGene00019846	R03G5.5	Stress response	Stress response: unassigned	Stress response: unassigned	gpx-7 is an ortholog of human GPX4\; is predicted to have glutathione peroxidase activity.
717	WBGene00019895	R05F9.12	Metabolism	Metabolism: carbohydrate	Metabolism: carbohydrate	aagr-2 is an ortholog of human MGAM (maltase-glucoamylase)\; exhibits alpha-1,4-glucosidase activity\; is involved in glycogen catabolic process.
718	WBGene00019929	R07C3.13	Unassigned	Unassigned	Unassigned: regulated by multiple stresses	R07C3.13 is enriched in the germ line and the male based on RNA-seq studies\; is affected by several genes including hsf-1, clk-1, and eat-2 based on tiling array, microarray, and RNA-seq studies\; is affected by thirteen chemicals including methylmercuric chloride, Rotenone, and manganese chloride based on microarray and RNA-seq studies.
719	WBGene00019939	R07G3.2	Metabolism	Metabolism: lipid	Metabolism: lipid: lipase	lips-17 is predicted to have hydrolase activity.

720	WBGene00020014	R11G11.6	Unassigned	Unassigned	Unassigned: regulated by multiple stresses	R11G11.6 is enriched in the OLL, the PVD, and the pharyngeal muscle cell based on microarray and tiling array studies\; is affected by several genes including daf-16, daf-12, and lin-4 based on microarray, tiling array, and RNA-seq studies\; is affected by seven chemicals including 1-methylnicotinamide, methylmercuric chloride, and Tunicamycin based on RNA-seq and microarray studies\; is predicted to encode a protein with the following domain: Domain of unknown function DUF148.
721	WBGene00020016	R11G11.14	Metabolism	Metabolism: lipid	Metabolism: lipid: lipase	lipl-3 is an ortholog of human LIPJ (lipase J), LIPK (lipase K), and LIPF (lipase F, gastric type)\; is predicted to have hydrolase activity, acting on ester bonds\; localizes to the lysosome\; human orthologs of this gene are implicated in autosomal recessive congenital ichthyosis 8 and lysosomal and lipase deficiency.
722	WBGene00020033	R12E2.7	Unassigned	Unassigned	Unassigned	R12E2.7 is expressed in several tissues, including the alimentary system, the hypodermis, the

						muscular system, the vulC, and the vulval syncytia precursor.
723	WBGene00020040	R12E2.15	Unassigned	Unassigned	Unassigned: regulated by multiple stresses	R12E2.15 is enriched in several tissues, including the alimentary system, the germline precursor cell, and the nervous system based on tiling array, RNA-seq, and microarray studies\; is affected by several genes including daf-16, age-1, and skn-1 based on microarray, RNA-seq, and tiling array studies\; is affected by twenty-one chemicals including hydrogen sulfide, methylmercury hydroxide, and 1-methylnicotinamide based on microarray and RNA-seq studies.
724	WBGene00020082	R57.1	Metabolism	Metabolism: 1CC	Metabolism: 1CC: folate	gcp-2.1 is an ortholog of human FOLH1, NAALAD2, and NAALADL1\; is predicted to have metallocarboxypeptidase activity.
725	WBGene00020105	R148.7	Unassigned	Unassigned	Unassigned: regulated by multiple stresses	R148.7 is enriched in the amphid sheath cell, the germ line, the intestine, the male, and the muscle cell based on RNA-seq and microarray studies\; is affected by several genes including age-1, dpy-10, and eat-2 based on microarray, RNA-seq, and tiling

						array studies\; is affected by fourteen chemicals including 1-methylnicotinamide, nicotinic acid, and methylmercuric chloride based on RNA-seq and microarray studies.
726	WBGene00020140	T01B11.4	Transmembrane transport	Transmembrane transport: mitochondrial	Transmembrane transport: mitochondrial: solute carrier	ant-1.4 is an ortholog of human SLC25A4 (solute carrier family 25 member 4), SLC25A5 (solute carrier family 25 member 5), and SLC25A6 (solute carrier family 25 member 6)\; is predicted to have transmembrane transporter activity\; localizes to the mitochondrion\; is expressed in several tissues, including the alimentary system, the muscular system, and the nervous system.
727	WBGene00020191	T03F1.10	Stress response	Stress response: C-type Lectin	Stress response: C-type Lectin	clec-53 is an ortholog of human COLEC11, CD302, and LY75\; is predicted to have carbohydrate binding activity.
728	WBGene00020232	T05A8.6	Unassigned	Unassigned	Unassigned	T05A8.6 is enriched in the male based on RNA-seq studies\; is affected by several genes including daf-16, daf-12, and aak-2 based on RNA-seq and microarray studies\; is affected by four chemicals including methylmercuric chloride, sodium

						arsenite, and single-walled carbon nanotube based on microarray and RNA-seq studies\; is predicted to encode a protein with the following domain: Domain of unknown function DUF281.
729	WBGene00020237	T05B4.3	Unassigned	Unassigned	Unassigned	phat-4 is involved in pharyngeal gland morphogenesis\; is expressed in the g1AL, the g1AR, the g1P, the g2L, and the g2R.
730	WBGene00020256	T05C3.6	Signaling	Signaling: lipid	Signaling: lipid: phospholipase: unassigned	T05C3.6 is an ortholog of human PLD3 (phospholipase D 3), PLD4 (phospholipase D 4), and PLD5 (phospholipase D 5)\; is predicted to have catalytic activity\; human orthologs of this gene are implicated in Alzheimer's disease 19.
731	WBGene00020258	T05E7.1	Metabolism	Metabolism: lipid	Metabolism: lipid: beta oxidation	T05E7.1 is an ortholog of human ACOT4 (acyl-CoA thioesterase 4), ACOT2 (acyl-CoA thioesterase 2), and BAAT\; is predicted to have thiolester hydrolase activity.
732	WBGene00020265	T05H4.2	Proteolysis proteasome	Proteolysis proteasome: E3	Proteolysis proteasome: E3: F box	fbxa-196 is enriched in the male based on RNA-seq studies\; is affected by several genes including daf-2, age-1, and hsf-1 based on microarray, RNA-seq, and tiling array studies\; is affected by nine

						chemicals including methylmercuric chloride, Tunicamycin, and manganese chloride based on microarray and RNA-seq studies\; is predicted to encode a protein with the following domains: F-box domain and Domain of unknown function DUF38, Caenorhabditis species.
733	WBGene00020281	T06A4.1	Proteolysis general	Proteolysis general: carboxypeptidase	Proteolysis general: carboxypeptidase	T06A4.1 is an ortholog of human CPA4 (carboxypeptidase A4), CPA1 (carboxypeptidase A1), and CPA6 (carboxypeptidase A6)\; is predicted to have metallocarboxypeptidase activity and zinc ion binding activity\; human orthologs of this gene are implicated in familial temporal lobe epilepsy 5.
734	WBGene00020283	T06A4.3	Proteolysis general	Proteolysis general: carboxypeptidase	Proteolysis general: carboxypeptidase	T06A4.3 is an ortholog of human CPA4 (carboxypeptidase A4), CPA1 (carboxypeptidase A1), and CPA6 (carboxypeptidase A6)\; is predicted to have metallocarboxypeptidase activity and zinc ion binding activity\; human orthologs of this gene are implicated in familial temporal lobe epilepsy 5.

735	WBGene00020289	T06C10.3	Signaling	Signaling: Y kinase	Signaling: Y kinase	T06C10.3 is an ortholog of human FER (FER tyrosine kinase)\; is predicted to have ATP binding activity and non-membrane spanning protein tyrosine kinase activity.
736	WBGene00020356	T08B6.9	Unassigned	Unassigned	Unassigned: regulated by multiple stresses	T08B6.9 is enriched in the male based on RNA-seq studies\; is affected by several genes including daf-2, dpy-10, and hsf-1 based on microarray, RNA-seq, and tiling array studies\; is affected by fifteen chemicals including Ethanol, methylmercury hydroxide, and nicotinic acid based on RNA-seq and microarray studies.
737	WBGene00020369	T08H10.1	Metabolism	Metabolism: short chain dehydrogenase	Metabolism: short chain dehydrogenase	T08H10.1 is an ortholog of human AKR1C1 (aldo-keto reductase family 1 member C1), AKR1C2 (aldo-keto reductase family 1 member C2), and AKR1D1 (aldo-keto reductase family 1 member D1)\; is predicted to have oxidoreductase activity.
738	WBGene00020413	T10E9.3	Unassigned	Unassigned	Unassigned: regulated by multiple stresses	T10E9.3 is enriched in the hypodermis based on tiling array studies\; is affected by several genes including daf-16, daf-2, and glp-1 based on tiling array, RNA-seq, and microarray studies\; is affected

						by twelve chemicals including hydrogen sulfide, nicotinic acid, and manganese chloride based on microarray and RNA-seq studies\; is predicted to encode a protein with the following domain: DOMON domain.
739	WBGene00020414	T10E9.4	Transmembrane protein	Transmembrane protein: unassigned	Transmembrane protein: unassigned	T10E9.4 is enriched in the germ line and the male based on proteomic and RNA-seq studies\; is affected by several genes including daf-2, let-7, and dpy-10 based on microarray, RNA-seq, and proteomic studies\; is affected by eighteen chemicals including Ethanol, methylmercury hydroxide, and 1-methylnicotinamide based on RNA-seq and microarray studies\; is predicted to encode a protein with the following domain: Domain of unknown function DX.
740	WBGene00020483	T13C5.2	Unassigned	Unassigned	Unassigned	T13C5.2 is enriched in the amphid sheath cell based on RNA-seq studies\; is affected by several genes including daf-16, daf-12, and alg-1 based on tiling array and RNA-seq studies\; is affected by methylmercuric chloride and multi-walled carbon

						nanotube based on microarray and RNA-seq studies\; is predicted to encode a protein with the following domain: Protein of unknown function DUF282, Caenorhabditis species.
741	WBGene00020551	T17H7.7	Unassigned	Unassigned	Unassigned: regulated by multiple stresses	T17H7.7 is enriched in the germline precursor cell, the hypodermis, the nervous system, and the somatic gonad precursor based on tiling array, RNA-seq, and microarray studies\; is affected by several genes including daf-16, daf-2, and daf-12 based on tiling array, RNA-seq, and microarray studies\; is affected by fourteen chemicals including Ethanol, 1-methylnicotinamide, and D-glucose based on RNA-seq and microarray studies.
742	WBGene00020554	T19A5.3	Development	Development: somatic	Development: somatic	T19A5.3 is enriched in the germline precursor cell and the hypodermis based on tiling array and RNA-seq studies\; is affected by several genes including daf-16, skn-1, and daf-12 based on microarray, tiling array, and RNA-seq studies\; is affected by seventeen chemicals including hydrogen sulfide, methylmercury hydroxide, and 1-

						methylnicotinamide based on microarray and RNA-seq studies.
743	WBGene00020560	T19C3.2	Unassigned	Unassigned	Unassigned: regulated by multiple stresses	T19C3.2 is enriched in the NSM, the germline precursor cell, and the hypodermis based on tiling array and RNA-seq studies\; is affected by several genes including daf-16, daf-12, and dpy-10 based on microarray and RNA-seq studies\; is affected by thirteen chemicals including hydrogen sulfide, Nitric Oxide, and methylmercury hydroxide based on microarray and RNA-seq studies.
744	WBGene00020567	T19D2.1	Proteolysis general	Proteolysis general: metallopeptidase	Proteolysis general: metallopeptidase	adt-3 is an ortholog of human ADAMTS14 (ADAM metallopeptidase with thrombospondin type 1 motif 14), ADAMTS2 (ADAM metallopeptidase with thrombospondin type 1 motif 2), and ADAMTS3 (ADAM metallopeptidase with thrombospondin type 1 motif 3)\; is predicted to have metalloendopeptidase activity\; is expressed in the rectal epithelium and the rectal gland cell.
745	WBGene00020616	T20D4.10	Unassigned	Unassigned	Unassigned: regulated by	T20D4.10 is enriched in the OLL, the PVD, and the muscle cell based on tiling array and RNA-seq

					multiple stresses	studies\; is affected by several genes including daf-16, daf-2, and glp-1 based on tiling array, RNA-seq, and microarray studies\; is affected by eight chemicals including hydrogen sulfide, 1-methylnicotinamide, and nicotinic acid based on microarray and RNA-seq studies\; is predicted to encode a protein with the following domains: Domain of unknown function DUF19 and Uncharacterised protein family UPF0376.
746	WBGene00020646	T21D11.1	Proteolysis general	Proteolysis general: metallopeptidase	Proteolysis general: metallopeptidase	zmp-6 is an ortholog of human MMP26 (matrix metallopeptidase 26)\; is predicted to have metalloendopeptidase activity and zinc ion binding activity.
747	WBGene00020649	T21D12.9	Signaling	Signaling: TGF beta	Signaling: TGF beta	sma-10 is an ortholog of human LRIG1 (leucine rich repeats and immunoglobulin like domains 1), LRIG2 (leucine rich repeats and immunoglobulin like domains 2), and LRIG3 (leucine rich repeats and immunoglobulin like domains 3)\; exhibits type I transforming growth factor beta receptor binding activity and type II transforming growth factor beta

						receptor binding activity\; is involved in several processes, including positive regulation of growth, positive regulation of transforming growth factor beta receptor signaling pathway, and regulation of mesodermal cell fate specification\; localizes to the integral component of plasma membrane\; is expressed in the hypodermis, the intestine, the pharynx, and the rectal gland cell.
748	WBGene00020674	T22B7.7	Metabolism	Metabolism: lipid	Metabolism: lipid: beta oxidation	T22B7.7 is an ortholog of human ACOT9 (acyl-CoA thioesterase 9)\; is predicted to encode a protein with the following domain: HotDog domain superfamily.
749	WBGene00020713	T23B3.5	Unassigned	Unassigned	Unassigned: regulated by multiple stresses	T23B3.5 is enriched in the germ line, the intestine, and the male based on proteomic, RNA-seq, and microarray studies\; is affected by several genes including daf-2, age-1, and daf-12 based on microarray, RNA-seq, and tiling array studies\; is affected by nineteen chemicals including Ethanol, nicotinic acid, and methylmercuric chloride based on RNA-seq and microarray studies.

750	WBGene00020741	T23F4.3	Unassigned	Unassigned	Unassigned: hydrolase	dlhd-1 is predicted to have hydrolase activity.
751	WBGene00020766	T24C12.4	Transmembrane protein	Transmembrane protein: unassigned	Transmembrane protein: unassigned	T24C12.4 is affected by several genes including daf-16, daf-2, and skn-1 based on microarray, tiling array, and RNA-seq studies\; is affected by eleven chemicals including 1-methylnicotinamide, multi-walled carbon nanotube, and D-glucose based on RNA-seq and microarray studies.
752	WBGene00020808	T25F10.6	Unassigned	Unassigned	Unassigned	clik-1 is enriched in the body wall musculature, the germ line, and the muscle cell based on tiling array, proteomic, RNA-seq, and microarray studies\; is affected by several genes including sir-2.1, lin-35, and pgl-1 based on RNA-seq, microarray, and proteomic studies\; is affected by eight chemicals including D-glucose, Aspirin, and Cholestanol based on RNA-seq and microarray studies\; is predicted to encode a protein with the following domain: Calponin repeat.
753	WBGene00020836	T27A1.4	Transmembrane transport	Transmembrane transport:	Transmembrane transport:	lgc-34 is an ortholog of human GABRR3 (gamma-aminobutyric acid type A receptor rho3 subunit

				extracellular ligand gated channel	extracellular ligand gated channel	(gene\/pseudogene)), GABRB2 (gamma-aminobutyric acid type A receptor beta2 subunit), and GABRP (gamma-aminobutyric acid type A receptor pi subunit)\; is predicted to have extracellular ligand-gated ion channel activity and transmembrane signaling receptor activity\; localizes to the plasma membrane\; is expressed in the head, the muscular system, and the reproductive system.
754	WBGene00020841	T27A3.5	Signaling	Signaling: phosphatase	Signaling: phosphatase: Y	T27A3.5 is predicted to have protein tyrosine phosphatase activity.
755	WBGene00020846	T27A10.6	Unassigned	Unassigned	Unassigned	T27A10.6 is expressed in the seam cell.
756	WBGene00020871	T28A11.4	Unassigned	Unassigned	Unassigned	T28A11.4 is enriched in dopaminergic neurons, the muscle cell, and the ventral nerve cord based on tiling array and RNA-seq studies\; is affected by several genes including daf-16, daf-12, and sir-2.1 based on tiling array, RNA-seq, and microarray studies\; is affected by copper sulfate and multi-walled carbon nanotube based on microarray and

						RNA-seq studies.
757	WBGene00020875	T28A11.13	Metabolism	Metabolism: unassigned	Metabolism: unassigned	T28A11.13 is an ortholog of human NXNL1 (nucleoredoxin like 1) and NXNL2 (nucleoredoxin like 2)\; is predicted to encode a protein with the following domains: Thioredoxin-like fold and Thioredoxin-like superfamily.
758	WBGene00020905	T28H11.7	Unassigned	Unassigned	Unassigned: regulated by multiple stresses	T28H11.7 is enriched in the excretory cell, the intestine, and the male based on tiling array, RNA-seq, and microarray studies\; is affected by several genes including daf-2, age-1, and hsf-1 based on microarray, tiling array, and RNA-seq studies\; is affected by twelve chemicals including Ethanol, nicotinic acid, and methylmercuric chloride based on RNA-seq and microarray studies\; is predicted to encode a protein with the following domain: AH\/BAR domain superfamily.
759	WBGene00020909	W01A11.1	Metabolism	Metabolism: lipid	Metabolism: lipid: epoxide hydrolase	W01A11.1 is an ortholog of human EPHX1\; is predicted to have cis-stilbene-oxide hydrolase activity\; is expressed in several tissues, including the alimentary system, the muscular system, the

						nervous system, and the reproductive system\; human orthologs of this gene are implicated in pre-eclampsia.
760	WBGene00020954	W02G9.4	Stress response	Stress response: pathogen	Stress response: pathogen: unassigned	W02G9.4 is an ortholog of human PCOLCE and PCOLCE2\; human PCOLCE exhibits collagen binding activity, heparin binding activity, and peptidase activator activity.
761	WBGene00020981	W03D2.6	Pseudogene	Pseudogene	Pseudogene	W03D2.6 is enriched in the OLL, the PVD, and the intestine based on microarray studies\; is affected by several genes including daf-16, daf-2, and glp-1 based on microarray, tiling array, and RNA-seq studies\; is affected by eight chemicals including Rotenone, manganese chloride, and D-glucopyranose based on RNA-seq and microarray studies.
762	WBGene00021070	W07B8.1	Proteolysis general	Proteolysis general: aspartate	Proteolysis general: aspartate: cathepsin	cpr-8 is an ortholog of human CTSB (cathepsin B)\; is predicted to have cysteine-type peptidase activity.
763	WBGene0002108	W08E12.3	Unassigned	Unassigned	Unassigned:	W08E12.3 is enriched in several tissues, including

	4				regulated by multiple stresses	the muscular system, the nervous system, and the pharynx based on microarray and Chronogram studies\; is affected by several genes including daf-16, daf-2, and skn-1 based on microarray, tiling array, and RNA-seq studies\; is affected by nine chemicals including Heme, Neurotoxins, and sesamin based on microarray and RNA-seq studies.
764	WBGene00021095	W08F4.6	Development	Development: somatic	Development: somatic	mlt-8 is an ortholog of human GM2A\; is predicted to have enzyme activator activity\; is expressed in the hypodermis.
765	WBGene00021128	W10C8.5	Metabolism	Metabolism: creatine kinase	Metabolism: creatine kinase	W10C8.5 is an ortholog of human CKMT1B, CKB, and CKM\; is predicted to have ATP binding activity and kinase activity.
766	WBGene00021160	Y4C6B.6	Metabolism	Metabolism: lipid	Metabolism: lipid: sphingolipid	gba-4 is an ortholog of human GBA (glucosylceramidase beta)\; is predicted to have glucosylceramidase activity.
767	WBGene00021288	Y25C1A.1	Stress response	Stress response: C-type Lectin	Stress response: C-type Lectin	clec-123 is predicted to have carbohydrate binding activity.
768	WBGene00021326	Y34B4A.10	Unassigned	Unassigned	Unassigned: regulated by	Y34B4A.10 is enriched in the germline precursor cell, the hypodermis, and the intestine based on

					multiple stresses	tiling array and microarray studies\; is affected by several genes including daf-16, daf-2, and daf-12 based on microarray and RNA-seq studies\; is affected by ten chemicals including hydrogen sulfide, Ethanol, and methylmercury hydroxide based on microarray and RNA-seq studies.
769	WBGene00021358	Y37E11AL.2	Transmembrane protein	Transmembrane protein: unassigned	Transmembrane protein: unassigned	Y37E11AL.2 is predicted to have protein phosphatase inhibitor activity.
770	WBGene00021389	Y38A10A.2	Unassigned	Unassigned	Unassigned	Y38A10A.2 is enriched in the germline precursor cell, the hypodermis, the nervous system, the sensillum, and the somatic gonad precursor based on microarray, tiling array, and RNA-seq studies\; is affected by several genes including daf-16, daf-12, and dpy-10 based on microarray, tiling array, and RNA-seq studies\; is affected by fourteen chemicals including Ethanol, tryptophan, and methylmercury hydroxide based on RNA-seq and microarray studies\; is predicted to encode a protein with the following domain: Protein of unknown

						function DUF229.
771	WBGene00021582	Y46C8AL.4	Stress response	Stress response: C-type Lectin	Stress response: C-type Lectin	clec-71 is predicted to have carbohydrate binding activity\; is involved in defense response to Gram-positive bacterium.
772	WBGene00021583	Y46C8AL.5	Stress response	Stress response: C-type Lectin	Stress response: C-type Lectin	clec-72 is predicted to have carbohydrate binding activity.
773	WBGene00021627	Y47D7A.15	Unassigned	Unassigned	Unassigned: regulated by multiple stresses	Y47D7A.15 is enriched in the coelomocyte and the ventral nerve cord based on tiling array studies\; is affected by several genes including daf-16, daf-2, and glp-1 based on RNA-seq and microarray studies\; is affected by seventeen chemicals including hydrogen sulfide, Ethanol, and 1-methylnicotinamide based on microarray and RNA-seq studies.
774	WBGene00021645	Y47G6A.19	Proteolysis general	Proteolysis general: carboxypeptidase	Proteolysis general: carboxypeptidase	Y47G6A.19 is an ortholog of human CPA4 (carboxypeptidase A4), CPA1 (carboxypeptidase A1), and CPA6 (carboxypeptidase A6)\; is predicted to have metallocarboxypeptidase activity and zinc ion binding activity\; human orthologs of this gene are implicated in familial temporal lobe

						epilepsy 5.
775	WBGene00021772	Y51H7BM.1	Unassigned	Unassigned	Unassigned: meprin-associated Traf homology domain	math-45 is enriched in the intestine based on tiling array and RNA-seq studies\; is affected by several genes including daf-2, skn-1, and hsf-1 based on microarray, tiling array, and RNA-seq studies\; is affected by five chemicals including Rotenone, Mianserin, and D-glucose based on RNA-seq and microarray studies\; is predicted to encode a protein with the following domains: MATH\/TRAF domain and TRAF-like.
776	WBGene00021779	Y51H7C.1	Unassigned	Unassigned	Unassigned: regulated by multiple stresses	Y51H7C.1 is enriched in the amphid sheath cell based on RNA-seq and microarray studies\; is affected by several genes including daf-2, glp-1, and dpy-10 based on microarray and RNA-seq studies\; is affected by twenty chemicals including Ethanol, 1-methylnicotinamide, and methylmercuric chloride based on RNA-seq and microarray studies.
777	WBGene00021791	Y51H7C.13	Unassigned	Unassigned	Unassigned: regulated by	Y51H7C.13 is enriched in the DA neuron, the NSM, the VA neuron, and the amphid sheath cell

					multiple stresses	based on tiling array, RNA-seq, and microarray studies\; is affected by several genes including daf-16, daf-2, and glp-1 based on microarray and RNA-seq studies\; is affected by seventeen chemicals including Ethanol, 1-methylnicotinamide, and nicotinic acid based on RNA-seq and microarray studies.
778	WBGene00021870	Y54G2A.4	Transmembrane transport	Transmembrane transport: major facilitator	Transmembrane transport: major facilitator	samt-1 is an ortholog of human MFSD5\; is predicted to have molybdate ion transmembrane transporter activity\; is expressed in the intestine, the pharyngeal gland cell, and the seam cell.
779	WBGene00021931	Y55F3AM.14	Nucleic acid	Nucleic acid: binding	Nucleic acid: binding: zinc finger	Y55F3AM.14 is an ortholog of human ZNF333 (zinc finger protein 333), ZNF454 (zinc finger protein 454), and ZNF354B (zinc finger protein 354B)\; is predicted to have nucleic acid binding activity\; is expressed in the dorsal nerve cord, the head muscle, the intestine, and the ventral nerve cord.
780	WBGene00021969	Y57G7A.6	Transmembrane protein	Transmembrane protein:	Transmembrane protein:	Y57G7A.6 is expressed in the hypodermis\; is predicted to encode a protein with the following

				unassigned	unassigned	domain: Domain of unknown function WSN.
781	WBGene00021996	Y59E9AL.6	Unassigned	Unassigned	Unassigned: regulated by multiple stresses	Y59E9AL.6 is enriched in the body wall muscle cell, the germ line, the hypodermis, the male, and the nervous system based on proteomic, tiling array, and RNA-seq studies\; is affected by several genes including daf-2, age-1, and dpy-10 based on microarray and RNA-seq studies\; is affected by twenty-one chemicals including Ethanol, methylmercury hydroxide, and 1-methylnicotinamide based on RNA-seq and microarray studies.
782	WBGene00022032	Y65B4A.9	Cytoskeleton	Cytoskeleton: microtubule	Cytoskeleton: microtubule: tau tubulin kinase	Y65B4A.9 is an ortholog of human CTD-2116N17.1, TTBK1 (tau tubulin kinase 1), and TTBK2\; is predicted to have ATP binding activity and protein kinase activity.
783	WBGene00022076	Y69A2AR.5	Metabolism	Metabolism: amino acid	Metabolism: amino acid: breakdown	daao-1 is an ortholog of human dao\; exhibits D-amino-acid oxidase activity\; is involved in oxidation-reduction process\; is expressed in the intestinal cell\; human orthologs of this gene are implicated in schizophrenia.

784	WBGene00022079	Y69A2AR.8	Pseudogene	Pseudogene	Pseudogene	Y69A2AR.8 is enriched in the male based on RNA-seq studies\; is affected by several genes including daf-12, dpy-10, and clk-1 based on tiling array, microarray, and RNA-seq studies\; is affected by thirteen chemicals including hydrogen sulfide, Ethanol, and methylmercuric chloride based on microarray and RNA-seq studies.
785	WBGene00022090	Y69A2AR.19	Signaling	Signaling: phosphatase	Signaling: phosphatase: Y	Y69A2AR.19 is predicted to have protein tyrosine phosphatase activity.
786	WBGene00022103	Y71D11A.1	Cytoskeleton	Cytoskeleton: cadherin	Cytoskeleton: cadherin	cdh-12 is an ortholog of human CELSR1 (cadherin EGF LAG seven-pass G-type receptor 1), CELSR2 (cadherin EGF LAG seven-pass G-type receptor 2), and CELSR3 (cadherin EGF LAG seven-pass G-type receptor 3)\; is predicted to have calcium ion binding activity.
787	WBGene00022155	Y71G12B.17	Signaling	Signaling: lipid	Signaling: lipid: unassigned	Y71G12B.17 is an ortholog of human PITPNA (phosphatidylinositol transfer protein alpha) and PITPNB (phosphatidylinositol transfer protein beta)\; is predicted to have phospholipid transporter activity.

788	WBGene00022245	Y73B6BL.24	Lysosome	Lysosome: acid phosphatase	Lysosome: acid phosphatase	acp-6 is an ortholog of human ACP2 (acid phosphatase 2, lysosomal), ACP4 (acid phosphatase 4), and ACPP (acid phosphatase, prostate)\; human ACPP exhibits phosphatase activity and protein homodimerization activity.
789	WBGene00022283	Y74E4A.1	Transmembrane transport	Transmembrane transport: extracellular ligand gated channel	Transmembrane transport: extracellular ligand gated channel	lgc-27 is an ortholog of human CHRNB3 (cholinergic receptor nicotinic beta 3 subunit), CHRNA4 (cholinergic receptor nicotinic alpha 4 subunit), and CHRNA1 (cholinergic receptor nicotinic alpha 1 subunit)\; is predicted to have extracellular ligand-gated ion channel activity\; is expressed in the AIYL and the AIYR.
790	WBGene00022287	Y75B7AR.1	Unassigned	Unassigned	Unassigned: regulated by multiple stresses	Y75B7AR.1 is enriched in several tissues, including the muscular system, the nervous system, and the pharynx based on Chronogram, microarray, and tiling array studies\; is affected by several genes including daf-16, daf-2, and glp-1 based on microarray and RNA-seq studies\; is affected by fifteen chemicals including 1-methylnicotinamide, nicotinic acid, and Mercuric Chloride based on

						RNA-seq and microarray studies.
791	WBGene00022324	Y82E9BL.7	Proteolysis proteasome	Proteolysis proteasome: E3	Proteolysis proteasome: E3: F box	fbxa-26 is enriched in the intestine based on RNA-seq studies\; is affected by several genes including daf-16, daf-2, and daf-12 based on microarray, tiling array, and RNA-seq studies\; is affected by seven chemicals including methylmercury hydroxide, methylmercuric chloride, and Mercuric Chloride based on RNA-seq and microarray studies\; is predicted to encode a protein with the following domains: F-box domain, Domain of unknown function DUF38, Caenorhabditis species, and F-box-like domain superfamily.
792	WBGene00022383	NA	NA	NA	NA	NA
793	WBGene00022415	Y102A11A.5	Unassigned	Unassigned	Unassigned: regulated by multiple stresses	Y102A11A.5 is enriched in the PLM, the hypodermis, and the intestine based on tiling array and RNA-seq studies\; is affected by several genes including daf-16, daf-12, and dpy-10 based on microarray and RNA-seq studies\; is affected by twelve chemicals including aldicarb,

						methylmercury hydroxide, and 1-methylnicotinamide based on microarray and RNA-seq studies.
794	WBGene00022421	Y102E9.3	Unassigned	Unassigned	Unassigned	Y102E9.3 is enriched in the NSM and the coelomocyte based on tiling array studies\; is affected by hpl-2, sir-2.1, and mir-243 based on tiling array and microarray studies\; is affected by Microcystin-LR based on microarray studies.
795	WBGene00022474	Y119C1B.9	Unassigned	Unassigned	Unassigned	zig-9 is enriched in the intestine based on tiling array and RNA-seq studies\; is affected by several genes including daf-16, daf-2, and glp-1 based on tiling array, proteomic, RNA-seq, and microarray studies\; is affected by diallyl trisulfide and Mianserin based on microarray and RNA-seq studies\; is predicted to encode a protein with the following domain: Immunoglobulin-like domain superfamily.
796	WBGene00022505	ZC21.3	Unassigned	Unassigned	Unassigned	ZC21.3 is expressed in the nervous system and the pharynx.
797	WBGene0002251	ZC64.2	Unassigned	Unassigned	Unassigned:	ttr-48 is enriched in the OLL, the PVD, the

	5				TransThyretin-Related family domain	intestine, and the muscle cell based on tiling array, RNA-seq, and microarray studies\; is affected by several genes including daf-16, daf-2, and glp-1 based on microarray, RNA-seq, tiling array, and proteomic studies\; is affected by nine chemicals including Rotenone, Mianserin, and D-glucose based on RNA-seq and microarray studies\; is predicted to encode a protein with the following domains: Transthyretin-like and Transthyretin-like superfamily.
798	WBGene00022545	ZC196.1	Pseudogene	Pseudogene	Pseudogene	ZC196.1 is enriched in the OLL and the PVD based on microarray studies\; is affected by several genes including daf-16, daf-2, and age-1 based on microarray, tiling array, and RNA-seq studies\; is affected by stavudine and Colistin based on RNA-seq and microarray studies.
799	WBGene00022578	ZC262.1	Pseudogene	Pseudogene	Pseudogene	ZC262.1 is affected by several genes including rrf-3, aak-2, and isp-1 based on RNA-seq and microarray studies\; is affected by five chemicals including aldicarb, resveratrol, and paraquat based

						on microarray studies\; is predicted to encode a protein with the following domains: Major sperm protein (MSP) domain, PapD-like superfamily, and Immunoglobulin-like fold.
800	WBGene00022621	ZC477.7	Unassigned	Unassigned	Unassigned: regulated by multiple stresses	ZC477.7 is enriched in the intestine and the male based on RNA-seq and microarray studies\; is affected by several genes including age-1, dpy-10, and hsf-1 based on microarray, tiling array, and RNA-seq studies\; is affected by eighteen chemicals including Ethanol, methylmercuric chloride, and manganese chloride based on RNA-seq and microarray studies\; is predicted to encode a protein with the following domain: AH\/BAR domain superfamily.
801	WBGene00022628	ZC513.3	Transmembrane protein	Transmembrane protein: unassigned	Transmembrane protein: unassigned	ZC513.3 is enriched in the male based on RNA-seq studies\; is affected by several genes including let-60, eat-2, and npr-1 based on microarray, RNA-seq, and tiling array studies\; is affected by twelve chemicals including methylmercuric chloride, manganese chloride, and D-glucose based on

						microarray and RNA-seq studies.
802	WBGene00022650	ZK84.2	Metabolism	Metabolism: lipid	Metabolism: lipid: sterol	ZK84.2 is enriched in the AFD and the male based on RNA-seq studies\; is affected by several genes including dpy-10, hsf-1, and clk-1 based on microarray and RNA-seq studies\; is affected by eight chemicals including methylmercuric chloride, manganese chloride, and D-glucose based on microarray and RNA-seq studies.
803	WBGene00022680	ZK180.6	Unassigned	Unassigned	Unassigned: regulated by multiple stresses	ZK180.6 is enriched in the DA neuron, the OLL, the PLM, and the PVD based on microarray and RNA-seq studies\; is affected by several genes including daf-16, daf-2, and glp-1 based on microarray, proteomic, and RNA-seq studies\; is affected by fourteen chemicals including hydrogen sulfide, Nitric Oxide, and methylmercury hydroxide based on microarray and RNA-seq studies.
804	WBGene00022743	ZK430.8	Extracellular material	Extracellular material: PGI	Extracellular material: PGI	mlt-7 is an ortholog of human PXDN (peroxidasin), LPO, and MPO\; exhibits peroxidase activity\; is involved in several processes, including cuticle

						development involved in collagen and cuticulin-based cuticle molting cycle, nematode larval development, and peptide cross-linking\; is expressed in the hypodermis.
805	WBGene00022751	ZK484.5	Unassigned	Unassigned	Unassigned: regulated by multiple stresses	msrp-2 is enriched in the amphid sheath cell and the male based on RNA-seq studies\; is affected by several genes including daf-2, glp-1, and age-1 based on microarray, tiling array, RNA-seq, and proteomic studies\; is affected by twenty-three chemicals including Ethanol, 1-methylnicotinamide, and nicotinic acid based on RNA-seq and microarray studies.
806	WBGene00022849	ZK1127.2	Metabolism	Metabolism: lipid	Metabolism: lipid: fatty acid	acs-6 is predicted to have catalytic activity.
807	WBGene00022856	ZK1127.10	Metabolism	Metabolism: 1CC	Metabolism: 1CC: transsulfuration	cth-2 is an ortholog of human CTH\; is predicted to have carbon-sulfur lyase activity and pyridoxal phosphate binding activity\; localizes to the endoplasmic reticulum\; is expressed in the anal depressor muscle, the body wall musculature, the hypodermis, and the reproductive system\; human

						orthologs of this gene are implicated in cystathioninuria.
808	WBGene00022858	ZK1193.2	Unassigned	Unassigned	Unassigned: regulated by multiple stresses	ZK1193.2 is enriched in the DA neuron, the I5 neuron, the SAB, the intestine, and the retrovesicular ganglion based on RNA-seq and microarray studies\; is affected by several genes including daf-16, daf-2, and glp-1 based on microarray, tiling array, proteomic, and RNA-seq studies\; is affected by fifteen chemicals including Ethanol, methylmercuric chloride, and Mercuric Chloride based on RNA-seq and microarray studies\; is predicted to encode a protein with the following domains: von Willebrand factor, type A and von Willebrand factor A-like domain superfamily.
809	WBGene00022926	C16B8.5	Non-coding RNA	Non-coding RNA: ncRNA	Non-coding RNA: ncRNA	C16B8.5 is affected by rsr-2 based on tiling array studies.
810	WBGene00023424	C53D6.10	Transmembrane protein	Transmembrane protein: unassigned	Transmembrane protein: unassigned	C53D6.10 is enriched in the male based on RNA-seq studies\; is affected by several genes including age-1, dpy-10, and hsf-1 based on microarray, tiling

						array, and RNA-seq studies\; is affected by thirteen chemicals including Ethanol, methylmercuric chloride, and Tunicamycin based on RNA-seq and microarray studies.
811	WBGene00044011	T06E4.12	Unassigned	Unassigned	Unassigned	T06E4.12 is predicted to be a structural constituent of cell wall.
812	WBGene00044019	C55A6.12	Proteolysis proteasome	Proteolysis proteasome: E3	Proteolysis proteasome: E3: unassigned	C55A6.12 is predicted to have poly-ADP-D-ribose binding activity and ubiquitin protein ligase activity.
813	WBGene00044109	K02E11.10	Unassigned	Unassigned	Unassigned: regulated by multiple stresses	K02E11.10 is affected by several genes including daf-16, glp-1, and skn-1 based on microarray and RNA-seq studies\; is affected by twelve chemicals including hydrogen sulfide, Ethanol, and Nitric Oxide based on microarray and RNA-seq studies.
814	WBGene00044184	F11A5.16	Unassigned	Unassigned	Unassigned	F11A5.16 is enriched in the germ line based on RNA-seq studies\; is affected by rsr-2 and smg-2 based on tiling array and RNA-seq studies.
815	WBGene00044192	F46B6.13	Unassigned	Unassigned	Unassigned	F46B6.13 is enriched in the NSM and the male based on tiling array and RNA-seq studies\; is affected by several genes including clk-1, sma-2,

						and sma-4 based on tiling array, microarray, and RNA-seq studies.
816	WBGene00044243	F16C3.4	Unassigned	Unassigned	Unassigned	F16C3.4 is enriched in the amphid sheath cell, the germ line, the intestine, and the male based on RNA-seq studies\; is affected by several genes including let-60, hsf-1, and clk-1 based on tiling array, microarray, and RNA-seq studies\; is affected by resveratrol and Colistin based on microarray studies.
817	WBGene00044257	Y45F10D.16	Transmembrane protein	Transmembrane protein: unassigned	Transmembrane protein: unassigned	Y45F10D.16 is enriched in the male based on RNA-seq studies\; is affected by several genes including bar-1, lem-2, and emr-1 based on microarray and RNA-seq studies\; is affected by multi-walled carbon nanotube and Colistin based on RNA-seq and microarray studies.
818	WBGene00044317	F41G3.19	Unassigned	Unassigned	Unassigned	F41G3.19 is affected by rsr-2 and dpy-21 based on tiling array and RNA-seq studies\; is predicted to encode a protein with the following domain: ShKT domain.
819	WBGene0004443	T23F4.5	Pseudogene	Pseudogene	Pseudogene	T23F4.5 is affected by several genes including dpy-

	0					21, hpl-2, and smg-2 based on tiling array and RNA-seq studies.
820	WBGene00044445	C02F5.14	Unassigned	Unassigned	Unassigned: regulated by multiple stresses	C02F5.14 is enriched in the PLM and the hypodermis based on tiling array and RNA-seq studies\; is affected by several genes including daf-2, hsf-1, and bar-1 based on RNA-seq, microarray, and tiling array studies\; is affected by five chemicals including 1-methylnicotinamide, nicotinic acid, and Rotenone based on RNA-seq and microarray studies.
821	WBGene00044521	Y5H2A.4	Unassigned	Unassigned	Unassigned	Y5H2A.4 is enriched in the DA neuron, the VA neuron, the body wall muscle cell, and the hypodermis based on tiling array studies\; is affected by several genes including clk-1, pmk-1, and aak-2 based on tiling array, RNA-seq, and microarray studies\; is affected by hydrogen sulfide, dafa#1, and adsorbable organic bromine compound based on microarray studies.
822	WBGene00044676	Y54F10BL.1	Proteolysis proteasome	Proteolysis proteasome: E3	Proteolysis proteasome: E3:	fbxa-42 is enriched in the AVE based on tiling array studies\; is affected by several genes including daf-

					F box	2, eat-2, and npr-1 based on tiling array, RNA-seq, and microarray studies\; is affected by four chemicals including multi-walled carbon nanotube, Alovudine, and Zidovudine based on RNA-seq and microarray studies\; is predicted to encode a protein with the following domains: F-box domain and Domain of unknown function DUF38, Caenorhabditis species.
823	WBGene00044697	K05F6.11	Stress response	Stress response: unassigned	Stress response: unassigned	poml-4 is an ortholog of human PON1 (paraoxonase 1), PON2 (paraoxonase 2), and PON3 (paraoxonase 3)\; is predicted to have arylesterase activity.
824	WBGene00044724	R02E4.3	Unassigned	Unassigned	Unassigned: regulated by multiple stresses	R02E4.3 is enriched in several tissues, including the excretory cell, the hypodermis, the intestine, and the nervous system based on tiling array and RNA-seq studies\; is affected by several genes including daf-16, daf-2, and daf-12 based on tiling array, RNA-seq, and microarray studies\; is affected by twelve chemicals including Ethanol, 1-methylnicotinamide, and Tunicamycin based on

						RNA-seq and microarray studies.
825	WBGene00044917	F40F8.12	Transmembrane protein	Transmembrane protein: unassigned	Transmembrane protein: unassigned	F40F8.12 is enriched in the intestine, the male, the nervous system, and the sensillum based on RNA-seq and microarray studies\; is affected by several genes including daf-2, clk-1, and sir-2.1 based on microarray studies\; is affected by Mianserin and Colistin based on RNA-seq and microarray studies.
826	WBGene00045355	D1086.17	Unassigned	Unassigned	Unassigned: regulated by multiple stresses	D1086.17 is enriched in the germ line and the male based on RNA-seq studies\; is affected by several genes including daf-2, hsf-1, and eat-2 based on microarray and RNA-seq studies\; is affected by eight chemicals including Ethanol, Rotenone, and manganese chloride based on RNA-seq and microarray studies.
827	WBGene00045456	C10A4.9	Transmembrane protein	Transmembrane protein: unassigned	Transmembrane protein: unassigned	C10A4.9 is affected by several genes including hsf-1, bar-1, and alg-1 based on RNA-seq and microarray studies\; is affected by Rotenone and Alovudine based on RNA-seq studies.
828	WBGene00045484	F34D10.9	Unassigned	Unassigned	Unassigned: regulated by	F34D10.9 is enriched in the Z1.p, the Z4.a, the gon_male_dtc anterior, the gon_male_dtc posterior,

					multiple stresses	and the male based on RNA-seq studies\; is affected by several genes including lin-4, lin-14, and hsf-1 based on microarray and RNA-seq studies\; is affected by eight chemicals including manganese chloride, Mianserin, and Zidovudine based on RNA-seq and microarray studies.
829	WBGene00045494	ZK662.6	Unassigned	Unassigned	Unassigned	ZK662.6 is enriched in the NSM based on tiling array studies\; is affected by several genes including bar-1, alg-1, and dpy-21 based on microarray, RNA-seq, and tiling array studies\; is affected by D-glucose based on RNA-seq studies.
830	WBGene00077489	C04G6.13	Unassigned	Unassigned	Unassigned: regulated by multiple stresses	C04G6.13 is enriched in the body wall muscle cell based on tiling array studies\; is affected by several genes including clk-1, pmk-1, and pgl-1 based on tiling array, RNA-seq, and microarray studies\; is affected by five chemicals including sodium arsenite, D-glucose, and tetrabromobisphenol A based on RNA-seq and microarray studies\; is predicted to encode a protein with the following domain: Protein of unknown function DUF282,

						Caenorhabditis species.
831	WBGene00077565	W07G4.7	Unassigned	Unassigned	Unassigned	W07G4.7 is affected by several genes including daf-16, daf-12, and clk-1 based on microarray and RNA-seq studies\; is affected by tryptophan and dafa#1 based on microarray studies.
832	WBGene00077577	R02D5.9	Transmembrane protein	Transmembrane protein: unassigned	Transmembrane protein: unassigned	R02D5.9 is enriched in the muscle cell and the nervous system based on microarray studies\; is affected by dpy-21 based on RNA-seq studies\; is affected by Sirolimus based on microarray studies.
833	WBGene00077691	T06E4.14	Unassigned	Unassigned	Unassigned: regulated by multiple stresses	T06E4.14 is enriched in the hypodermis, the intestine, and the pharyngeal muscle cell based on tiling array, RNA-seq, and microarray studies\; is affected by several genes including daf-16, skn-1, and daf-12 based on tiling array, RNA-seq, and microarray studies\; is affected by eleven chemicals including Ethanol, methylmercury hydroxide, and 1-methylnicotinamide based on RNA-seq and microarray studies.
834	WBGene00077697	F58E6.13	Unassigned	Unassigned	Unassigned: regulated by	F58E6.13 is enriched in the PLM, the Z1, the Z4, and the hypodermis based on tiling array and RNA-

					multiple stresses	seq studies\; is affected by several genes including daf-2, lin-4, and lin-14 based on microarray, proteomic, tiling array, and RNA-seq studies\; is affected by eighteen chemicals including Nitric Oxide, methylmercury hydroxide, and nicotinic acid based on RNA-seq and microarray studies\; is predicted to encode a protein with the following domains: EGF-like domain, Growth factor receptor cysteine-rich domain superfamily, Tyrosine-protein kinase ephrin type A\/B receptor-like, and EGF domain.
835	WBGene00077701	E01A2.10	Stress response	Stress response: unassigned	Stress response: unassigned	poml-3 is an ortholog of human PON1 (paraoxonase 1), PON2 (paraoxonase 2), and PON3 (paraoxonase 3)\; human PON1 exhibits several functions, including calcium ion binding activity, hydrolase activity, acting on ester bonds, and phospholipid binding activity.
836	WBGene00086556	F36D1.12	Unassigned	Unassigned	Unassigned	F36D1.12 is enriched in the nervous system based on RNA-seq studies\; is affected by daf-16 based on RNA-seq studies\; is affected by tryptophan

						based on microarray studies.
837	WBGene00138711	R07B1.13	Unassigned	Unassigned	Unassigned: regulated by multiple stresses	R07B1.13 is enriched in the AFD, the PLM, the coelomocyte, and the hypodermis based on tiling array and RNA-seq studies\; is affected by several genes including daf-16, daf-12, and hsf-1 based on RNA-seq and microarray studies\; is affected by seven chemicals including methylmercury hydroxide, 1-methylnicotinamide, and D-glucose based on RNA-seq and microarray studies\; is predicted to encode a protein with the following domain: C6 domain.
838	WBGene00194670	K07E3.9	Transmembrane protein	Transmembrane protein: unassigned	Transmembrane protein: unassigned	K07E3.9 is enriched in the amphid sheath cell based on RNA-seq studies\; is affected by several genes including alg-1, alg-2, and set-9 based on RNA-seq and tiling array studies\; is affected by Rotenone and multi-walled carbon nanotube based on RNA-seq studies.
839	WBGene00194703	F26B1.8	Unassigned	Unassigned	Unassigned: regulated by multiple stresses	F26B1.8 is enriched in the intestine, the male, and the seam cell based on RNA-seq and microarray studies\; is affected by several genes including daf-

						2, dpy-10, and gld-1 based on RNA-seq and microarray studies\; is affected by thirteen chemicals including Ethanol, Rotenone, and manganese chloride based on RNA-seq and microarray studies.
840	WBGene00195048	K08D10.13	Unassigned	Unassigned	Unassigned	K08D10.13 is affected by nhr-114, let-418, and chd-3 based on microarray and RNA-seq studies\; is predicted to encode a protein with the following domain: ShKT domain.
841	WBGene00195068	Y48G8AL.16	Unassigned	Unassigned	Unassigned	Y48G8AL.16 is enriched in the AFD based on RNA-seq studies\; is affected by several genes including aak-2, isp-1, and hpl-2 based on RNA-seq and microarray studies\; is affected by Chlorpyrifos and Colistin based on microarray studies.
842	WBGene00195144	C16D2.3	Unassigned	Unassigned	Unassigned	N.A.
843	WBGene00195356	F57H12.10	Non-coding RNA	Non-coding RNA: ncRNA	Non-coding RNA: ncRNA	N.A.
844	WBGene0019672	T27F2.6	Non-coding	Non-coding	Non-coding	N.A.

	4		RNA	RNA: ncRNA	RNA: ncRNA	
845	WBGene00197520	C05C8.10	Non-coding RNA	Non-coding RNA: ncRNA	Non-coding RNA: ncRNA	C05C8.10 is affected by multi-walled carbon nanotube based on RNA-seq studies.
846	WBGene00198564	F49H12.12	Non-coding RNA	Non-coding RNA: ncRNA	Non-coding RNA: ncRNA	F49H12.12 is affected by several genes including daf-16, daf-12, and prg-1 based on RNA-seq studies.
847	WBGene00198728	W04D2.10	Non-coding RNA	Non-coding RNA: ncRNA	Non-coding RNA: ncRNA	W04D2.10 is affected by cep-1 based on RNA-seq studies.
848	WBGene00198906	R166.8	Non-coding RNA	Non-coding RNA: ncRNA	Non-coding RNA: ncRNA	N.A.
849	WBGene00199504	Y49G5B.4	Non-coding RNA	Non-coding RNA: ncRNA	Non-coding RNA: ncRNA	Y49G5B.4 is affected by cep-1 based on RNA-seq studies.
850	WBGene00200400	C16E9.21	Non-coding RNA	Non-coding RNA: ncRNA	Non-coding RNA: ncRNA	C16E9.21 is affected by several genes including pgl-1, glh-1, and pgl-3 based on RNA-seq studies.
851	WBGene00202113	B0457.18	Non-coding RNA	Non-coding RNA: ncRNA	Non-coding RNA: ncRNA	B0457.18 is affected by cep-1 based on RNA-seq studies.
852	WBGene00206360	T13C5.10	Unassigned	Unassigned	Unassigned	T13C5.10 is enriched in the hypodermis based on RNA-seq studies\; is affected by several genes including eri-1, rrf-1, and mut-2 based on microarray studies\; is affected by single-walled

						carbon nanotube and allantoin based on microarray studies\; is predicted to encode a protein with the following domain: Protein of unknown function DUF282, Caenorhabditis species.
853	WBGene00206373	C27A2.12	Lysosome	Lysosome: acid phosphatase	Lysosome: acid phosphatase	C27A2.12 is an ortholog of human ACP2 (acid phosphatase 2, lysosomal), ACP4 (acid phosphatase 4), and ACPP (acid phosphatase, prostate)\; human ACPP exhibits phosphatase activity and protein homodimerization activity.
854	WBGene00206537	Y71H9A.10	Unassigned	Unassigned	Unassigned	Y71H9A.10 is affected by daf-12, daf-16, and dpy-21 based on RNA-seq studies\; is predicted to encode a protein with the following domain: Saposin-like.
855	WBGene00219294	F43G6.16	Unassigned	Unassigned	Unassigned	N.A.
856	WBGene00219644	B0507.16	Non-coding RNA	Non-coding RNA: linc	Non-coding RNA: linc	N.A.
857	WBGene00220073	T19E7.29	Non-coding RNA	Non-coding RNA: snoRNA	Non-coding RNA: snoRNA	T19E7.29 is affected by cep-1 based on RNA-seq studies.
858	WBGene0022026	ZC434.10	Unassigned	Unassigned	Unassigned	ZC434.10 is enriched in the PLM based on RNA-

	1					seq studies\; is affected by several genes including daf-16, daf-12, and lin-4 based on RNA-seq and microarray studies\; is affected by Zidovudine, Colistin, and Sirolimus based on RNA-seq and microarray studies.
859	WBGene0023528 1	Y4C6A.68	Pseudogene	Pseudogene	Pseudogene	Y4C6A.68 is enriched in the male based on microarray studies\; is affected by isp-1 and nuo-6 based on RNA-seq studies\; is affected by Sodium Chloride based on RNA-seq studies.
860	WBGene0023532 6	F22F7.11	Unassigned	Unassigned	Unassigned	N.A.
861	WBGene0025537 5	F08B4.8	Unassigned	Unassigned	Unassigned	F08B4.8 is enriched in the nervous system based on RNA-seq studies\; is affected by several genes including daf-2, dpy-10, and gld-1 based on RNA-seq and microarray studies\; is affected by nine chemicals including Atrazine, Diazinon, and Quercetin based on microarray studies.
862	WBGene0025570 2	C06A12.19	Transmembran e protein	Transmembrane protein: unassigned	Transmembrane protein: unassigned	N.A.

863	WBGene00255709	Y116A8C.471	Non-coding RNA	Non-coding RNA: ncRNA	Non-coding RNA: ncRNA	N.A.

Table S2. The 72 uniquely downregulated stress- and defense-related genes in *C. elegans* are associated with the DAF-2/DAF-16 pathway.

Wormbase.ID	Sequence.ID	Category.2	log_2FC	p-value	q-value（FDR）	Regulate
WBGene00000024	*AC3.3(abu-1)*	Stress response: ER	-3.43472	0.0002	0.00345935	*daf-2,daf-16*
WBGene00000033	*F35A5.3(abu-10)*	Stress response: ER	-2.444786667	0.00035	0.00529575	*daf-16*
WBGene00000034	*T01D1.6(abu-11)*	Stress response: ER	-2.55859	0.00005	0.00110206	*daf-16*
WBGene00001753	*R03D7.6(gst-5)*	Stress response: detoxification	-2.009426	0.0006	0.012147	*daf-2,daf-16*
WBGene00001754	*F11G11.3(gst-6)*	Stress response: detoxification	-1.935388	0.001	0.0113532	*daf-2,daf-16*
WBGene00001758	*Y45G12C.2(gst-10)*	Stress response: detoxification	-2.903745	0.00005	0.00193593	*daf-2,daf-16*
WBGene00001760	*F37B1.2(gst-12)*	Stress response: detoxification	-2.672795	0.0012	0.0829538	*daf-16*
WBGene00001774	*Y53F4B.29(gst-26)*	Stress response: detoxification	-2.541996	0.00055	0.00740175	*daf-2,daf-16*
WBGene00001775	*Y53F4B.30(gst-27)*	Stress response: detoxification	-1.707338	0.00135	0.0190682	*daf-16*
WBGene00002012	*F38E11.1*	Stress response: heat	-2.951026	0.00045	0.018779	*daf-2,daf-16*
WBGene00003096	*C02A12.4 (lys-7)*	Stress response: pathogen	-3.5237775	0.00005	0.00110206	*daf-2,daf-16*
WBGene00003473	*K11G9.6(mtl-1)*	Stress response: heavy metal	-3.439072	0.0003	0.0269886	*daf-2,daf-16*
WBGene00003771	*T19C4.7(nlp-33)*	Stress response: pathogen	-1.913573333	0.0097	0.103324	*daf-16*

WBGene00003778	*C15H9.1*	Stress response: unassigned	-2.277972	0.00005	0.00110206	*daf-2,daf-16*
WBGene00004932	*C08A9.1*	Stress response: oxidative	-3.335956	0.00005	0.00471148	*daf-2,daf-16*
WBGene00004987	*T08A9.12(spp-2)*	Stress response: pathogen	-3.86695	0.00005	0.00110206	*daf-2,daf-16*
WBGene00004993	*C28C12.5(spp-8)*	Stress response: pathogen	-1.719284	0.00005	0.00197766	*daf-2*
WBGene00004998	*F08F1.6(spp-13)*	Stress response: pathogen	-2.846145	0.0003	0.0302499	*daf-2,daf-16*
WBGene00005003	*F27C8.4(spp-18)*	Stress response: pathogen	-1.940476	0.00005	0.00687417	*daf-2,daf-16*
WBGene00007072	*AC3.7(ugt-1)*	Stress response: detoxification	-2.716073333	0.0003	0.00687417	*daf-2,daf-16*
WBGene00007362	*C06B3.3(cyp-35C1)*	Stress response: detoxification	-1.7577975	0.0023	0.0418043	*daf-2,daf-16*
WBGene00007565	*C14A6.1*	Stress response: C-type Lectin	-1.2927225	0.00105	0.0117915	*daf-2,daf-16*
WBGene00007963	*C36A4.1*	Stress response: detoxification	-3.469282	0.00005	0.00728649	*daf-2,daf-16*
WBGene00007964	*C36A4.2*	Stress response: detoxification	-3.197956	0.00005	0.00193593	*daf-2,daf-16*
WBGene00008296	*C54D10.1*	Stress response: heavy metal	-2.993795	0.00005	0.00110206	*daf-2,daf-16*
WBGene00008596	*F08H9.8(clec-54)*	Stress response: C-type Lectin	-2.0772	0.00005	0.00464441	*daf-16*
WBGene00008597	*F08H9.9(clec-55)*	Stress response: C-type Lectin	−2.8320	0.00545	0.04065	*daf-2,daf-16*
WBGene00008810	*F14F7.3(cyp-13A12)*	Stress response: detoxification	-3.63139	0.002	0.0362498	*daf-2,daf-16*
WBGene00009215	*F28D1.5(thn-2)*	Stress response: pathogen	-2.346864	0.00005	0.00110206	*daf-2,daf-16*

WBGene00009226	*F28G4.1(cyp-37B1)*	Stress response: detoxification	-2.415194	0.00005	0.00110206	*daf-2,daf-16*
WBGene00009430	*F35E12.6*	Stress response: pathogen	-1.794743333	0.00005	0.00110206	*daf-2,daf-16*
WBGene00009515	*F38A1.1*	Stress response: C-type Lectin	-3.037026667	0.0025	0.0237149	*daf-2,daf-16*
WBGene00010034	*F54B11.11*	Stress response: pathogen	-2.655736667	0.0067	0.0535184	*daf-2*
WBGene00010657	*K08D8.3*	Stress response: pathogen	-3.04081	0.00065	0.0083666	*daf-2,daf-16*
WBGene00011362	*T02B5.1(cest-1.1)*	Stress response: detoxification	-6.235842	0.00005	0.00652902	*daf-2,daf-16*
WBGene00011495	*T05F1.11*	Stress response: thioredoxin	-3.321295	0.00005	0.00464441	*daf-2,daf-16*
WBGene00011535	*T06E4.8(idpc-5)*	Stress response: ER	-3.904316667	0.00055	0.0109508	*daf-2,daf-16*
WBGene00011668	*T09F5.9(clec-47)*	Stress response: C-type Lectin	-1.32268	0.00155	0.0155028	*daf-2,daf-16*
WBGene00011830	*T19B10.1*	Stress response: detoxification	-1.6922175	0.00075	0.0183359	*daf-2,daf-16*
WBGene00011844	*T19C9.8*	Stress response: pathogen	-3.9184875	0.00165	0.0330974	*daf-2,daf-16*
WBGene00012433	*Y11D7A.9(bus-24)*	Stress response: unassigned	-2.8252375	0.00035	0.00529575	*daf-2,daf-16*
WBGene00013875	*ZC376.3(cest-2.1)*	Stress response: detoxification	-1.9754325	0.0019	0.0177989	*daf-2,daf-16*
WBGene00014046	*ZK666.6(clec-60)*	Stress response: C-type Lectin	-3.832824	0.00005	0.00110206	*daf-2,daf-16*
WBGene00014697	*C36A4.3(cyp-25A3)*	Stress response: detoxification	-1.52297	0.0103	0.0933144	*daf-2,daf-16*
WBGene00015044	*B0213.15(cyp-34A9)*	Stress response: detoxification	-2.120404	0.00005	0.00193593	*daf-2,daf-16*

WBGene00015050	*B0218.6(clec-51)*	Stress response: C-type Lectin	-2.653158	0.00075	0.0315165	*daf-2,daf-16*
WBGene00015193	*B0432.12(clec-117)*	Stress response: C-type Lectin	-2.09621	0.0159	0.0685398	*daf-2*
WBGene00015400	*C03G6.15(cyp-35A2)*	Stress response: detoxification	-2.684624	0.00005	0.00110206	*daf-2,daf-16*
WBGene00015695	*C10H11.6(ugt-26)*	Stress response: detoxification	-1.950406667	0.0019	0.0177989	*daf-2,daf-16*
WBGene00015739	*C13D9.9(ugt-7)*	Stress response: detoxification	-1.8422	0.00615	0.0788232	*daf-16*
WBGene00016013	*C23G10.6(ugt-66)*	Stress response: detoxification	-1.89515	0.00445	0.054264	*daf-2,daf-16*
WBGene00016329	*C32E12.3(osr-1)*	Stress response: osmotic	-3.015046667	0.00185	0.0174989	*daf-2,daf-16*
WBGene00017089	*E01A2.7(poml-2)*	Stress response: unassigned	-2.83387	0.0017	0.0164851	*daf-2,daf-16*
WBGene00017218	*F07F6.5(dct-5)*	Stress response: pathogen	-2.7625325	0.00125	0.0164851	*daf-2,daf-16*
WBGene00017336	*F10D2.11(ugt-41)*	Stress response: detoxification	-2.2549	0.00005	0.00193593	*daf-2,daf-16*
WBGene00017364	*F10G2.3(clec-70)*	Stress response: C-type Lectin	-3.62486	0.0024	0.03806	*daf-2,daf-16*
WBGene00017991	*F32E10.3(clec-180)*	Stress response: C-type Lectin	-2.5891575	0.00015	0.0027619	*daf-2,daf-16*
WBGene00018292	*F41E6.11(idpc-2)*	Stress response: ER	-2.546946667	0.00045	0.00637744	*daf-16*
WBGene00018334	*F42A9.5(cyp-33E2)*	Stress response: detoxification	-2.01168	0.00145	0.014816	*daf-2,daf-16*
WBGene00018548	*F47C12.4(clec-79)*	Stress response: C-type Lectin	-1.51947	0.0033	0.0253951	*daf-2*
WBGene00018760	*F53E10.4(irg-3)*	Stress response: pathogen	-2.094006667	0.01035	0.0721776	*daf-2,daf-16*

WBGene00019473	*K07C6.5(cyp-35A5)*	Stress response: detoxification	-2.49468	0.00615	0.0382854	*daf-2,daf-16*
WBGene00019515	*K08B4.3(ugt-19)*	Stress response: detoxification	-2.512896	0.00005	0.00728649	*daf-2*
WBGene00019803	*PDB1.1*	Stress response: pathogen	-2.217126	0.0001	0.00345935	*daf-2,daf-16*
WBGene00019846	*R03G5.5(gpx-7)*	Stress response: unassigned	-3.117166	0.0004	0.0144568	*daf-2,daf-16*
WBGene00020191	*T03F1.10(clec-53)*	Stress response: C-type Lectin	-2.83204	0.00545	0.0406502	*daf-2*
WBGene00020954	*W02G9.4*	Stress response: pathogen	-2.189734	0.00005	0.00728649	*daf-2*
WBGene00021288	*Y25C1A.1*	Stress response: C-type Lectin	-1.495613333	0.00515	0.0340992	*daf-2,daf-16*
WBGene00021582	*Y46C8AL.4(clec-71)*	Stress response: C-type Lectin	-3.289934	0.00015	0.0175826	*daf-2,daf-16*
WBGene00021583	*Y46C8AL.5(clec-72)*	Stress response: C-type Lectin	-2.78023	0.0004	0.00586466	*daf-16*
WBGene00044697	*K05F6.11(poml-4)*	Stress response: unassigned	-2.57607	0.00975	0.0638383	*daf-2*
WBGene00077701	*E01A2.10(poml-3)*	Stress response: unassigned	-2.019878	0.0003	0.00529575	*daf-2,daf-16*

Table S3 Toxicity assays of different samples against *C. elegans* nematodes.

Strain	LC_{50}（μg/mL）	95%CL		Intercept	Slope	X^2(df)
		Lower	Upper			
Cry5Ba	25.679	19.704	34.427	-0.806	0.031	5.271（5）
BMB 171/Cry5Ba	3.374	0.692	5.841	-0.462	0.137	10.875（5）
BMB 171Δ*plcR*/Cry5Ba	7.981	6.31	9.795	-0.935	0.117	6.697（4）
BMB 171Δ*plcRpisP*/Cry5Ba	15.624	12.562	19.521	-1.121	0.072	4.950（4）
BMB 171Δ*pisP*/Cry5Ba	4.489	3.16	6.043	-0.595	0.133	3.711（3）

Table S4. List of the 74 protein-coding genes enriched at PlcR binding sites identified by ChIP-seq.

Gene_ID	Transcript_ID	Annotation	Function
BMB171_RS01115	BMB171_RS01115	Promoter	Glucose uptake
BMB171_RS01260	BMB171_RS01260	Promoter	Belongs to the ABC transporter superfamily
BMB171_RS01645	BMB171_RS01645	Promoter	Methyltransferase domain
BMB171_RS01685	BMB171_RS01685	Promoter	EamA-like transporter family
BMB171_RS01800	ligA	Promoter	DNA ligase that catalyzes the formation of phosphodiester linkages between 5'-phosphoryl and 3'-hydroxyl groups in double-stranded DNA using NAD as a coenzyme and as the energy source for the reaction. It is essential for DNA replication and repair of damaged DNA
BMB171_RS30755	BMB171_RS30755	Promoter	#N/A
BMB171_RS02000	mtnA	Promoter	Catalyzes the interconversion of methylthioribose-1- phosphate (MTR-1-P) into methylthioribulose-1-phosphate (MTRu-1- P)
BMB171_RS02570	pflA	Promoter	Activation of pyruvate formate-lyase under anaerobic conditions by generation of an organic free radical, using S-adenosylmethionine and reduced flavodoxin as cosubstrates to produce 5'-deoxy-adenosine

BMB171_RS02995	BMB171_RS02995	Promoter	chemotaxis protein
BMB171_RS03425	inhA	Promoter	Peptidase M6
BMB171_RS04130	BMB171_RS04130	Promoter	COG1388 FOG LysM repeat
BMB171_RS30775	BMB171_RS30775	Promoter	#N/A
BMB171_RS04275	BMB171_RS04275	Promoter	DoxX
BMB171_RS30310	BMB171_RS30310	Promoter	N-acetylmuramoyl-L-alanine amidase family 2
BMB171_RS05525	BMB171_RS05525	Promoter	Bacterial PH domain
BMB171_RS05740	aceA	Promoter	Isocitrate lyase
BMB171_RS06125	BMB171_RS06125	Promoter	Peptidase_G2, IMC autoproteolytic cleavage domain
BMB171_RS29085	BMB171_RS29085	Promoter	Enoyl-(Acyl carrier protein) reductase
BMB171_RS06435	BMB171_RS06435	Promoter	#N/A
BMB171_RS06790	queF	Promoter	Catalyzes the NADPH-dependent reduction of 7-cyano-7- deazaguanine (preQ0) to 7-aminomethyl-7-deazaguanine (preQ1)
BMB171_RS07425	dacB	Promoter	Belongs to the peptidase S11 family
BMB171_RS07590	BMB171_RS07590	Promoter	YIEGIA protein

BMB171_RS07760	bshA	Promoter	N-acetyl-alpha-D-glucosaminyl L-malate synthase
BMB171_RS07925	ypwA	Exon	Broad specificity carboxypetidase that releases amino acids sequentially from the C-terminus, including neutral, aromatic, polar and basic residues
BMB171_RS08490	BMB171_RS08490	Promoter	DNA binding domain of the motility gene repressor (MogR)
BMB171_RS08795	BMB171_RS08795	Promoter	Bacterial PH domain
BMB171_RS11925	BMB171_RS11925	Promoter	#N/A
BMB171_RS11935	BMB171_RS11935	Promoter	Transmembrane secretion effector
BMB171_RS12190	BMB171_RS12190	Promoter	#N/A
BMB171_RS12210	BMB171_RS12210	Promoter	hmm pf01734
BMB171_RS12695	BMB171_RS12695	Promoter	COG1167 Transcriptional regulators containing a DNA-binding HTH domain and an aminotransferase domain (MocR family) and their eukaryotic orthologs
BMB171_RS12710	BMB171_RS12710	Promoter	Protein of unknown function (DUF3937)
BMB171_RS12940	BMB171_RS12940	Promoter	peptidoglycan catabolic process
BMB171_RS13460	pulA	Promoter	Belongs to the glycosyl hydrolase 13 family

BMB171_RS13575	kynA	Promoter	Heme-dependent dioxygenase that catalyzes the oxidative cleavage of the L-tryptophan (L-Trp) pyrrole ring and converts L- tryptophan to N-formyl-L-kynurenine. Catalyzes the oxidative cleavage of the indole moiety
BMB171_RS13660	BMB171_RS13660	Promoter	-
BMB171_RS13970	BMB171_RS13970	Promoter	-
BMB171_RS14490	BMB171_RS14490	Promoter	drug exporters of the RND superfamily
BMB171_RS15775	BMB171_RS15775	Promoter	DUF218 domain
BMB171_RS15910	BMB171_RS15910	Promoter	Beta-lactamase
BMB171_RS15965	BMB171_RS15965	Promoter	-
BMB171_RS16250	BMB171_RS16250	Promoter	Bacterial capsule synthesis protein PGA_cap
BMB171_RS16385	BMB171_RS16385	Promoter	Ribonuclease
BMB171_RS16745	BMB171_RS16745	Promoter	chemotaxis protein
BMB171_RS17475	colA	Promoter	collagenase
BMB171_RS18460	BMB171_RS18460	Promoter	chitinase
BMB171_RS18645	BMB171_RS18645	Promoter	Bacterial pre-peptidase C-terminal domain

BMB171_RS19315	ileS2	Promoter	amino acids such as valine, to avoid such errors it has two additional distinct tRNA(Ile)-dependent editing activities. One activity is designated as 'pretransfer' editing and involves the hydrolysis of activated Val-AMP. The other activity is designated 'posttransfer' editing and involves deacylation of mischarged Val-tRNA(Ile)
BMB171_RS19555	ctaD	Promoter	Cytochrome c oxidase is the component of the respiratory chain that catalyzes the reduction of oxygen to water. Subunits 1-3 form the functional core of the enzyme complex. CO I is the catalytic subunit of the enzyme. Electrons originating in cytochrome c are transferred via the copper A center of subunit 2 and heme A of subunit 1 to the bimetallic center formed by heme A3 and copper B
BMB171_RS20315	BMB171_RS20315	Promoter	YtkA-like
BMB171_RS20375	BMB171_RS20375	Promoter	Belongs to the 5'-nucleotidase family
BMB171_RS21115	BMB171_RS21115	Promoter	#N/A
BMB171_RS21620	aroE	Promoter	Involved in the biosynthesis of the chorismate, which leads to the biosynthesis of aromatic amino acids. Catalyzes the reversible NADPH linked reduction of 3-dehydroshikimate (DHSA) to yield shikimate (SA)

BMB171_RS21640	BMB171_RS21640	Promoter	Catalyzes the formation of phosphatidylethanolamine (PtdEtn) from phosphatidylserine (PtdSer)
BMB171_RS21700	BMB171_RS21700	Promoter	-
BMB171_RS22285	lon	Promoter	ATP-dependent serine protease that mediates the selective degradation of mutant and abnormal proteins as well as certain short-lived regulatory proteins. Required for cellular homeostasis and for survival from DNA damage and developmental changes induced by stress. Degrades polypeptides processively to yield small peptide fragments that are 5 to 10 amino acids long. Binds to DNA in a double-stranded, site-specific manner
BMB171_RS22675	BMB171_RS22675	Promoter	Belongs to the short-chain dehydrogenases reductases (SDR) family
BMB171_RS23720	pckA	Promoter	Involved in the gluconeogenesis. Catalyzes the conversion of oxaloacetate (OAA) to phosphoenolpyruvate (PEP) through direct phosphoryl transfer between the nucleoside triphosphate and OAA
BMB171_RS23915	BMB171_RS23915	Promoter	DUF218 domain
BMB171_RS24130	BMB171_RS24130	Promoter	COG0577, ABC-type antimicrobial peptide transport system, permease component

BMB171_RS24745	BMB171_RS24745	Promoter	-
BMB171_RS24765	glpX	Promoter	fructose-1,6-bisphosphatase
BMB171_RS24945	BMB171_RS24945	Promoter	CAAX protease self-immunity
BMB171_RS25110	BMB171_RS25110	Promoter	Protein of unknown function (DUF3978)
BMB171_RS25400	BMB171_RS25400	Promoter	ABC transporter, ATP-binding protein
BMB171_RS26595	BMB171_RS26595	Promoter	#N/A
BMB171_RS26725	BMB171_RS26725	Promoter	Bacillus PapR protein
BMB171_RS26770	BMB171_RS26770	Promoter	PA domain
BMB171_RS26810	BMB171_RS26810	Promoter	Protein of unknown function (DUF1093)
BMB171_RS26855	BMB171_RS26855	Promoter	Peptidase M50
BMB171_RS27260	BMB171_RS27260	Promoter	ABC-type bacteriocin lantibiotic exporters, contain an N-terminal double-glycine peptidase domain
BMB171_RS27270	BMB171_RS27270	Promoter	#N/A
BMB171_RS28020	BMB171_RS28020	Distal Intergenic	-
BMB171_RS28405	BMB171_RS28405	Promoter	chemotaxis protein

Table S5. Pesticidal protein repertoires and inferred potential target spectra of 889 *Bacillus thuringiensis* strains.

Genome accession	Organism Name	Strain	Pesticidal protein(s)	Number of pesticidal proteins	PisP status	Matched pesticidal protein class(es)	Potential target order(s)	Representative pest species
GCF_979908215.1	*Bacillus thuringiensis*	SRL368	Cry11Aa3,Cry10Aa4,Cry4Ba7,Cry4Aa5,Cyt1Ca1,Cyt1Aa11,Cyt2Ba17,Mpp60Ba3,Mpp60Aa3	9	No	cry11A,cry10A,cry4B,cry4A,cyt1A,cyt2B,Mpp60B,Mpp60A	Diptera;Coleoptera;Hemiptera;Lepidoptera	*Aedes aegypti;Anopheles albimanus;Culex quinquefasciatu;Culex pipiens;cotton boll weevil;Anopheles gambiae;Diaphorina citri;Culex quinquefasciatus;Acyrthosiphon pisum;Culex pipien;Plutella xylostella;Anopheles stephensi;Bradysia difformis*
GCF_979895345.1	*Bacillus thuringiensis*	SRL215	App4Aa1,Spp1Aa1	2	No	App4A,Spp1A	Lepidoptera;Hemiptera;Blattodea	*Plutella xylostella;Laodelphax striatellus;Spodoptera litura;Blattella germanica*
GCF_918202735.1	*Bacillus thuringiensis*	ISI 2824	Cry2Aa22,Cry2Ab4,Cry1Ia42,Cry1Ea7,Vip3Aa59,Spp1Aa1	6	Yes	cry2A,cry2A,cry1I,cry1E,vip3Aa,Spp1A	Lepidoptera;Diptera;Hemiptera;Coleoptera;Rhabditida;Blattodea;Aedes aegypti	*Helicoverpa armigera;Aedes aegypti;Diaphorina citri;Leptinotarsa decemlineata;Ostrinia furnacalis;Lobesia botrana;Plutella xylostella;Earias insulana;cotton boll weevil;Meloidogyne incognita;Spodoptera exigua;Spodoptera frugiperda;Malacosoma disstria;Bursaphelenchus*

								xylophilus;Helicoverpa punctigera;Helicoverpa zea;Chilo partellus;Blattella germanica;Spodoptera litura
GCF_901007655.1	*Bacillus thuringiensis serovar israelensis*	AR23	Cry11Aa3,Cry10Aa4,Cry4Ba7,Cry4Aa5,Cyt1Ca1,Cyt1Aa11,Cyt2Ba17	7	No	cry11A,cry10A,cry4B,cry4A,cyt1A,cyt2B	Diptera;Coleoptera;Hemiptera;Lepidoptera	*Aedes aegypti;Anopheles albimanus;Culex quinquefasciatu;Culex pipiens;cotton boll weevil;Anopheles gambiae;Diaphorina citri;Culex quinquefasciatus;Acyrthosiphon pisum;Culex pipien;Plutella xylostella;Anopheles stephensi;Bradysia difformis*
GCF_900094685.1	*Bacillus thuringiensis*	IEBC_T61001	Spp1Aa1	1	No	Spp1A	Lepidoptera;Blattodea	*Spodoptera litura;Blattella germanica*
GCF_058133255.1	*Bacillus thuringiensis*	T381	Cry1Ia42,Cry2Aa22,Cry2Ab41,Vip3Aa79,Spp1Aa1	5	Yes	cry1I,cry2A,cry2A,vip3Aa,Spp1A	Coleoptera;Lepidoptera;Rhabditida;Diptera;Hemiptera;Blattodea;Aedes aegypti	*Leptinotarsa decemlineata;Ostrinia furnacalis;Lobesia botrana;Plutella xylostella;Earias insulana;cotton boll weevil;Meloidogyne incognita;Helicoverpa armigera;Aedes aegypti;Diaphorina citri;Helicoverpa punctigera;Spodoptera frugiperda;Helicoverpa zea;Chilo partellus;Blattella germanica;Spodoptera litura*
GCF_057	*Bacillus*	SC125	Sip1A2-other,Spp1Aa1	2	No	Spp1A	Lepidoptera;Blatto	*Spodoptera litura;Blattella*

731695.1	*thuringiensis*	3					dea	*germanica*
GCF_057657355.1	*Bacillus thuringiensis*	Soil_C_13			No			
GCF_057656775.1	*Bacillus thuringiensis*	Soil_C_4			No			
GCF_057656475.1	*Bacillus thuringiensis*	Soil_C_56	Spp1Aa1	1	Yes	Spp1A	Lepidoptera;Blattodea	*Spodoptera litura;Blattella germanica*
GCF_057624775.1	*Bacillus thuringiensis*	BUPM2016	Cry1Ia42,Cry2Aa22,Cry2Ab41,Vip3Aa79,Spp1Aa1	5	Yes	cry1I,cry2A,cry2A,vip3Aa,Spp1A	Coleoptera;Lepidoptera;Rhabditida;Diptera;Hemiptera;Blattodea;Aedes aegypti	*Leptinotarsa decemlineata;Ostrinia furnacalis;Lobesia botrana;Plutella xylostella;Earias insulana;cotton boll weevil;Meloidogyne incognita;Helicoverpa armigera;Aedes aegypti;Diaphorina citri;Helicoverpa punctigera;Spodoptera frugiperda;Helicoverpa zea;Chilo partellus;Blattella germanica;Spodoptera litura*
GCF_057624755.1	*Bacillus thuringiensis*	MEB18	Cry1Ia42,Cry2Aa22,Cry2Ab41,Vip3Aa79,Spp1Aa1	5	Yes	cry1I,cry2A,cry2A,vip3Aa,Spp1A	Coleoptera;Lepidoptera;Rhabditida;Diptera;Hemiptera;Blattodea;Aedes aegypti	*Leptinotarsa decemlineata;Ostrinia furnacalis;Lobesia botrana;Plutella xylostella;Earias insulana;cotton boll weevil;Meloidogyne incognita;Helicoverpa armigera;Aedes aegypti;Diaphorina*

								citri;Helicoverpa punctigera;Spodoptera frugiperda;Helicoverpa zea;Chilo partellus;Blattella germanica;Spodoptera litura
GCF_057624695.1	*Bacillus thuringiensis*	BST30	Cry70Ba1,App4Aa1,Sip1A2-other,Spp1Aa1,Xpp22Bb1	5	Yes	App4A,Spp1A,Xpp22B	Lepidoptera;Hemiptera;Blattodea;Coleoptera	*Plutella xylostella;Laodelphax striatellus;Spodoptera litura;Blattella germanica;Anthonomus grandis*
GCF_057624655.1	*Bacillus thuringiensis*	BLB250	Cry1Ia42,Cry2Ab41,Cry9Ea12,Cry1Ca18,Cry1Da2,Cry1Aa3,Vip3Aa79,Spp1Aa1	8	Yes	cry1I,cry2A,Cry9E,cry1C,cry1D,cry1A,vip3Aa,Spp1A	Coleoptera;Lepidoptera;Rhabditida;Diptera;Hemiptera;Dipteran;Blattodea;Aedes aegypti	*Leptinotarsa decemlineata;Ostrinia furnacalis;Lobesia botrana;Plutella xylostella;Earias insulana;cotton boll weevil;Meloidogyne incognita;Helicoverpa armigera;Aedes aegypti;Diaphorina citri;Agrotis exclamationis;Cydia pomonella;Myzus persicae;Ostrinia nubilalis;Spodoptera exigua;Spodoptera frugiperda;Bombyx mori;Spodoptera litura;Epiphyas postvittana;Leucinodes orbonalis;Trichoplusia ni;Hyphantria cunea;Pieris rapae;Chilo suppressalis;Helicoverpa punctigera;Helicoverpa zea;Chilo partellus;Blattella germanica*
GCF_057624615.1	*Bacillus thuringi*	BST28	Spp1Aa1	1	Yes	Spp1A	Lepidoptera;Blattodea	*Spodoptera litura;Blattella germanica*

	ensis							
GCF_057474585.1	*Bacillus thuringiensis*	CH100	Xpp22Ba1	1	Yes	Xpp22B	Coleoptera;Lepidoptera	*Anthonomus grandis;Plutella xylostella*
GCF_057167325.1	*Bacillus thuringiensis*	G10	Cry8Ia1,Cry8La1,Spp1Aa1	3	No	cry8I,Spp1A	Coleoptera;Lepidoptera;Blattodea	*Holotrichia parallela;Spodoptera litura;Blattella germanica*
GCF_057167305.1	*Bacillus thuringiensis*	S15	Cry1Ia42,Cry2Aa22,Cry2Ab41,Cry1Ab9,Vip3Aa79,Spp1Aa1	6	Yes	cry1I,cry2A,cry2A,cry1A,vip3Aa,Spp1A	Coleoptera;Lepidoptera;Rhabditida;Diptera;Hemiptera;Blattodea;Aedes aegypti	*Leptinotarsa decemlineata;Ostrinia furnacalis;Lobesia botrana;Plutella xylostella;Earias insulana;cotton boll weevil;Meloidogyne incognita;Helicoverpa armigera;Aedes aegypti;Diaphorina citri;Leucinodes orbonalis;Trichoplusia ni;Hyphantria cunea;Spodoptera frugiperda;Pieris rapae;Chilo suppressalis;Helicoverpa punctigera;Helicoverpa zea;Chilo partellus;Blattella germanica;Spodoptera litura*
GCF_057167285.1	*Bacillus thuringiensis*	TYR	Cry1Ia42,Cry2Aa22,Cry2Ab41,Cry1Aa3,Vip3Aa79,Spp1Aa1	6	No	cry1I,cry2A,cry2A,cry1A,vip3Aa,Spp1A	Coleoptera;Lepidoptera;Rhabditida;Diptera;Hemiptera;Blattodea;Aedes aegypti	*Leptinotarsa decemlineata;Ostrinia furnacalis;Lobesia botrana;Plutella xylostella;Earias insulana;cotton boll weevil;Meloidogyne incognita;Helicoverpa armigera;Aedes aegypti;Diaphorina citri;Leucinodes*

								orbonalis;Trichoplusia ni;Hyphantria cunea;Spodoptera frugiperda;Pieris rapae;Chilo suppressalis;Helicoverpa punctigera;Helicoverpa zea;Chilo partellus;Blattella germanica;Spodoptera litura
GCF_057167245.1	*Bacillus thuringiensis*	D1	Cry8Ia1,Cry8La1,Spp1Aa1	3	No	cry8I,Spp1A	Coleoptera;Lepidoptera;Blattodea	*Holotrichia parallela;Spodoptera litura;Blattella germanica*
GCF_057167205.1	*Bacillus thuringiensis*	Bz87	Cry1Ia42,Cry2Aa22,Cry2Ab41,Vip3Aa79,Spp1Aa1	5	Yes	cry1I,cry2A,cry2A,vip3Aa,Spp1A	Coleoptera;Lepidoptera;Rhabditida;Diptera;Hemiptera;Blattodea;Aedes aegypti	*Leptinotarsa decemlineata;Ostrinia furnacalis;Lobesia botrana;Plutella xylostella;Earias insulana;cotton boll weevil;Meloidogyne incognita;Helicoverpa armigera;Aedes aegypti;Diaphorina citri;Helicoverpa punctigera;Spodoptera frugiperda;Helicoverpa zea;Chilo partellus;Blattella germanica;Spodoptera litura*
GCF_056833535.1	*Bacillus thuringiensis serovar israelensis*	VCRC-B651	Cry11Aa3,Cry10Aa4,Cry4Ba7,Cyt1Ca1,Cyt1Aa11,Cyt2Ba17,Mpp60Ba3,Mpp60Aa3	8	No	cry11A,cry10A,cry4B,cyt1A,cyt2B,Mpp60B,Mpp60A	Diptera;Coleoptera;Hemiptera;Lepidoptera	*Aedes aegypti;Anopheles albimanus;Culex quinquefasciatu;Culex pipiens;cotton boll weevil;Anopheles gambiae;Diaphorina citri;Culex quinquefasciatus;Culex pipien;Plutella xylostella;Anopheles*

								stephensi;Bradysia difformis
GCF_056832985.1	*Bacillus thuringiensis*	MH778713	Spp1Aa1	1	No	Spp1A	Lepidoptera;Blattodea	*Spodoptera litura;Blattella germanica*
GCF_056826315.1	*Bacillus thuringiensis*	Bt_BJ	Spp1Aa1	1	Yes	Spp1A	Lepidoptera;Blattodea	*Spodoptera litura;Blattella germanica*
GCF_056158625.1	*Bacillus thuringiensis*	ABM4_481	Cry11Aa3,Cry10Aa4,Cry4Ba7,Cry4Aa5,Cyt1Ca1,Cyt1Aa11,Cyt2Ba17,Mpp60Ba3,Mpp60Aa3	9	No	cry11A,cry10A,cry4B,cry4A,cyt1A,cyt2B,Mpp60B,Mpp60A	Diptera;Coleoptera;Hemiptera;Lepidoptera	*Aedes aegypti;Anopheles albimanus;Culex quinquefasciatu;Culex pipiens;cotton boll weevil;Anopheles gambiae;Diaphorina citri;Culex quinquefasciatus;Acyrthosiphon pisum;Culex pipien;Plutella xylostella;Anopheles stephensi;Bradysia difformis*
GCF_056158525.1	*Bacillus thuringiensis*	ABR4_001	Cry11Aa3,Cry4Aa5,Cyt1Ca1,Cyt1Aa11,Cyt2Ba17,Mpp60Ba3,Mpp60Aa3	7	No	cry11A,cry4A,cyt1A,cyt2B,Mpp60B,Mpp60A	Diptera;Hemiptera;Coleoptera;Lepidoptera	*Aedes aegypti;Anopheles albimanus;Culex quinquefasciatu;Culex pipiens;Culex quinquefasciatus;Acyrthosiphon pisum;Culex pipien;Diaphorina citri;Plutella xylostella;Anopheles stephensi;Anopheles gambiae;Bradysia difformis*
GCF_055633145.1	*Bacillus thuringiensis*	ESY57	Spp1Aa1	1	Yes	Spp1A	Lepidoptera;Blattodea	*Spodoptera litura;Blattella germanica*
GCF_055	*Bacillus*	GIFSP	Spp1Aa1	1	Yes	Spp1A	Lepidoptera;Blatto	*Spodoptera litura;Blattella*

269965.1	*thuringiensis*	R-111					dea	*germanica*
GCF_054332455.1	*Bacillus thuringiensis*	B-T-3	Spp1Aa1	1	Yes	Spp1A	Lepidoptera;Blattodea	*Spodoptera litura;Blattella germanica*
GCF_054058055.1	*Bacillus thuringiensis*	TE21534			Yes			
GCF_053712235.1	*Bacillus thuringiensis*	RDA_24Y_64			Yes			
GCF_053712195.1	*Bacillus thuringiensis*	RDA_24Y_67	Spp1Aa1	1	Yes	Spp1A	Lepidoptera;Blattodea	*Spodoptera litura;Blattella germanica*
GCF_053711855.1	*Bacillus thuringiensis*	RDA_B_21	Spp1Aa1	1	Yes	Spp1A	Lepidoptera;Blattodea	*Spodoptera litura;Blattella germanica*
GCF_053711815.1	*Bacillus thuringiensis*	RDA_B_32	Spp1Aa1	1	Yes	Spp1A	Lepidoptera;Blattodea	*Spodoptera litura;Blattella germanica*
GCF_053629975.1	*Bacillus thuringiensis*	141350017-1	Spp1Aa1	1	Yes	Spp1A	Lepidoptera;Blattodea	*Spodoptera litura;Blattella germanica*
GCF_053578475.1	*Bacillus thuringiensis*	T419	Cry1Ia42,Cry2Aa22,Cry2Ab41,Vip3Aa79,Spp1Aa1	5	Yes	cry1I,cry2A,cry2A,vip3Aa,Spp1A	Coleoptera;Lepidoptera;Rhabditida;Diptera;Hemiptera;Blattodea;Aedes	*Leptinotarsa decemlineata;Ostrinia furnacalis;Lobesia botrana;Plutella xylostella;Earias insulana;cotton boll weevil;Meloidogyne*

							aegypti	*incognita;Helicoverpa armigera;Aedes aegypti;Diaphorina citri;Helicoverpa punctigera;Spodoptera frugiperda;Helicoverpa zea;Chilo partellus;Blattella germanica;Spodoptera litura*
GCF_052938895.1	*Bacillus thuringiensis*	T407	Cry1Ia42,Cry2Aa22,Cry2Ab41,Vip3Aa79,Spp1Aa1	5	Yes	cry1I,cry2A,cry2A,vip3Aa,Spp1A	Coleoptera;Lepidoptera;Rhabditida;Diptera;Hemiptera;Blattodea;Aedes aegypti	*Leptinotarsa decemlineata;Ostrinia furnacalis;Lobesia botrana;Plutella xylostella;Earias insulana;cotton boll weevil;Meloidogyne incognita;Helicoverpa armigera;Aedes aegypti;Diaphorina citri;Helicoverpa punctigera;Spodoptera frugiperda;Helicoverpa zea;Chilo partellus;Blattella germanica;Spodoptera litura*
GCF_051853075.1	*Bacillus thuringiensis*	1Fo	Cry1Ia42,Cry2Aa22,Cry2Ab41,Vip3Aa79,Spp1Aa1	5	Yes	cry1I,cry2A,cry2A,vip3Aa,Spp1A	Coleoptera;Lepidoptera;Rhabditida;Diptera;Hemiptera;Blattodea;Aedes aegypti	*Leptinotarsa decemlineata;Ostrinia furnacalis;Lobesia botrana;Plutella xylostella;Earias insulana;cotton boll weevil;Meloidogyne incognita;Helicoverpa armigera;Aedes aegypti;Diaphorina citri;Helicoverpa punctigera;Spodoptera frugiperda;Helicoverpa zea;Chilo partellus;Blattella*

								germanica;Spodoptera litura
GCF_051453465.1	*Bacillus thuringiensis*	MPOBBt1	Cry1Ab9,Cry9Ea12,Cry1Ca18,Cry1Da2,Spp1Aa1	5	Yes	cry1A,Cry9E,cry1C,cry1D,Spp1A	Lepidoptera;Diptera;Blattodea	*Plutella xylostella;Ostrinia furnacalis;Helicoverpa armigera;Leucinodes orbonalis;Trichoplusia ni;Hyphantria cunea;Spodoptera frugiperda;Pieris rapae;Chilo suppressalis;Agrotis exclamationis;Cydia pomonella;Myzus persicae;Aedes aegypti;Ostrinia nubilalis;Spodoptera exigua;Bombyx mori;Spodoptera litura;Epiphyas postvittana;Blattella germanica*
GCF_051390695.1	*Bacillus thuringiensis serovar kurstaki*	SA_11	Cry2Aa22,Cry2Ab41,Vip3Aa79,Spp1Aa1	4	Yes	cry2A,cry2A,vip3Aa,Spp1A	Lepidoptera;Diptera;Hemiptera;Blattodea;Aedes aegypti	*Helicoverpa armigera;Aedes aegypti;Diaphorina citri;Helicoverpa punctigera;Spodoptera frugiperda;Helicoverpa zea;Chilo partellus;Blattella germanica;Spodoptera litura*
GCF_051146765.1	*Bacillus thuringiensis*	LBA001	Cry1Ia42,Cry2Ab41,Cry9Ea12,Cry1Da2,Cry1Ca18,Vip3Aa79,Spp1Aa1	7	Yes	cry1I,cry2A,Cry9E,cry1D,cry1C,vip3Aa,Spp1A	Coleoptera;Lepidoptera;Rhabditida;Diptera;Hemiptera;Dipteran;Blattodea;Aedes aegypti	*Leptinotarsa decemlineata;Ostrinia furnacalis;Lobesia botrana;Plutella xylostella;Earias insulana;cotton boll weevil;Meloidogyne incognita;Helicoverpa armigera;Aedes aegypti;Diaphorina citri;Agrotis exclamationis;Cydia pomonella;Ostrinia nubilalis;Spodoptera*

								exigua;Spodoptera frugiperda;Bombyx mori;Spodoptera litura;Epiphyas postvittana;Myzus persicae;Helicoverpa punctigera;Helicoverpa zea;Chilo partellus;Blattella germanica
GCF_051027395.1	*Bacillus thuringiensis*	wqa40	Spp1Aa1	1	No	Spp1A	Lepidoptera;Blattodea	*Spodoptera litura;Blattella germanica*
GCF_050703525.1	*Bacillus thuringiensis*	B3	Cry1Ac9,Cry1Ia42,Cry2Ab41,Cry9Ea12,Cry1Ca18,Cry1Da2,Cry1Aa3,Vip3Aa79,Spp1Aa1	9	Yes	cry1A,cry1I,cry2A,Cry9E,cry1C,cry1D,cry1A,vip3Aa,Spp1A	Lepidoptera;Coleoptera;Rhabditida;Diptera;Hemiptera;Dipteran;Blattodea;Aedes aegypti	*Plutella xylostella;Ostrinia furnacalis;Helicoverpa armigera;Leucinodes orbonalis;Trichoplusia ni;Hyphantria cunea;Spodoptera frugiperda;Pieris rapae;Chilo suppressalis;Leptinotarsa decemlineata;Lobesia botrana;Earias insulana;cotton boll weevil;Meloidogyne incognita;Aedes aegypti;Diaphorina citri;Agrotis exclamationis;Cydia pomonella;Myzus persicae;Ostrinia nubilalis;Spodoptera exigua;Bombyx mori;Spodoptera litura;Epiphyas postvittana;Helicoverpa punctigera;Helicoverpa zea;Chilo partellus;Blattella germanica*
GCF_050	*Bacillus*	ZJ192	Cry70Ba1,Cry52Aa1,Si	6	No	Spp1A,Tpp80A,Tpp8	Lepidoptera;Blatto	*Spodoptera litura;Blattella*

327825.1	*thuringiensis*		p1A2-other,Spp1Aa1,Tpp80Aa2,Tpp80Ad1			0A	dea;Diptera;Hemiptera	*germanica;Culex pipiens pallens;Culex quinquefasciatus;Aedes aegypti;Anopheles gambiae;Laodelphax striatellus*
GCF_050272815.1	*Bacillus thuringiensis*	NPDC046185	Spp1Aa1	1	Yes	Spp1A	Lepidoptera;Blattodea	*Spodoptera litura;Blattella germanica*
GCF_050270215.1	*Bacillus thuringiensis*	NPDC071044	Cry1Ba8,Cry1Ab9,Mpp5Ab1,Spp1Aa1,Vpa2Bb4,Vpb1Bb2,Vpb1Bc1	7	Yes	cry1B,cry1A,Mpp5A,Spp1A	Lepidoptera;Hemiptera;Diptera;Coleoptera;Blattodea	*Plutella xylostella;Ostrinia nubilalis;Spodoptera frugiperda;Maruca vitrata;Manduca sexta;cotton boll weevil;Ostrinia furnacalis;Helicoverpa armigera;Leucinodes orbonalis;Trichoplusia ni;Hyphantria cunea;Pieris rapae;Chilo suppressalis;Colaphellus bowringi;Leptinotarsa decemlineata;Spodoptera litura;Blattella germanica*
GCF_050107415.1	*Bacillus thuringiensis*	159b	Spp1Aa1	1	Yes	Spp1A	Lepidoptera;Blattodea	*Spodoptera litura;Blattella germanica*
GCF_050107095.1	*Bacillus thuringiensis*	452a	Spp1Aa1	1	No	Spp1A	Lepidoptera;Blattodea	*Spodoptera litura;Blattella germanica*
GCF_049367375.1	*Bacillus thuringi*	74	Spp1Aa1	1	No	Spp1A	Lepidoptera;Blattodea	*Spodoptera litura;Blattella germanica*

	ensis							
GCF_048984675.1	*Bacillus thuringiensis*	barcode 15	Spp1Aa1	1	No	Spp1A	Lepidoptera;Blattodea	*Spodoptera litura;Blattella germanica*
GCF_048159105.1	*Bacillus thuringiensis*	M4	Spp1Aa1	1	No	Spp1A	Lepidoptera;Blattodea	*Spodoptera litura;Blattella germanica*
GCF_047783265.1	*Bacillus thuringiensis*	SC136	Spp1Aa1	1	Yes	Spp1A	Lepidoptera;Blattodea	*Spodoptera litura;Blattella germanica*
GCF_047130435.1	*Bacillus thuringiensis*	G03	Cry1Ia42,Cry2Ab41,Cry9Ea12,Cry1Ca18,Cry1Da2,Cry1Aa3,Vip3Aa79,Spp1Aa1	8	Yes	cry1I,cry2A,Cry9E,cry1C,cry1D,cry1A,vip3Aa,Spp1A	Coleoptera;Lepidoptera;Rhabditida;Diptera;Hemiptera;Dipteran;Blattodea;Aedes aegypti	*Leptinotarsa decemlineata;Ostrinia furnacalis;Lobesia botrana;Plutella xylostella;Earias insulana;cotton boll weevil;Meloidogyne incognita;Helicoverpa armigera;Aedes aegypti;Diaphorina citri;Agrotis exclamationis;Cydia pomonella;Myzus persicae;Ostrinia nubilalis;Spodoptera exigua;Spodoptera frugiperda;Bombyx mori;Spodoptera litura;Epiphyas postvittana;Leucinodes orbonalis;Trichoplusia ni;Hyphantria cunea;Pieris rapae;Chilo suppressalis;Helicoverpa punctigera;Helicoverpa zea;Chilo partellus;Blattella germanica*

GCF_047049475.1	*Bacillus thuringiensis serovar sumiyoshiensis*	4AO1	Cry1Ie3,Cry9Da4,Cry1Na2,Cry9Ee3,Cry9Aa4,Cry9Eb3,Vip3Aa51,Spp1Aa1	8	Yes	cry1I,Cry9D,cry1N,Cry9E,Cry9A,Cry9E,vip3Aa,Spp1A	Coleoptera;Lepidoptera;Rhabditida;Blattodea;Aedes aegypti	*Leptinotarsa decemlineata;Ostrinia furnacalis;Lobesia botrana;Plutella xylostella;Earias insulana;cotton boll weevil;Meloidogyne incognita;Bombyx mori;Hyphantria cunea;Colaphellus bowringi;Agrotis exclamationis;Cydia pomonella;Pieris brassicae;Phthorimaea operculella;Chilo suppressalis;Helicoverpa punctigera;Spodoptera frugiperda;Helicoverpa zea;Chilo partellus;Blattella germanica;Spodoptera litura*
GCF_047049285.1	*Bacillus thuringiensis serovar fukuokaensis*	4AP1	Cry1Ie3,Cry9Aa4,Cry1Na2,Cry9Ee3,Cry9Da4,Vip3Aa51,Spp1Aa1	7	Yes	cry1I,Cry9A,cry1N,Cry9E,Cry9D,vip3Aa,Spp1A	Coleoptera;Lepidoptera;Rhabditida;Blattodea;Aedes aegypti	*Leptinotarsa decemlineata;Ostrinia furnacalis;Lobesia botrana;Plutella xylostella;Earias insulana;cotton boll weevil;Meloidogyne incognita;Pieris brassicae;Phthorimaea operculella;Chilo suppressalis;Colaphellus bowringi;Agrotis exclamationis;Cydia pomonella;Bombyx mori;Hyphantria cunea;Helicoverpa punctigera;Spodoptera frugiperda;Helicoverpa zea;Chilo*

								partellus;Blattella germanica;Spodoptera litura
GCF_046717365.1	*Bacillus thuringiensis*	HQB-200	Spp1Aa1	1	No	Spp1A	Lepidoptera;Blattodea	*Spodoptera litura;Blattella germanica*
GCF_046619515.1	*Bacillus thuringiensis*	MPB12			No			
GCF_046599155.1	*Bacillus thuringiensis*	HD-1	Cry1Ab9,Cry1Ia42,Cry2Aa22,Cry2Ab41,Cry1Aa17,Cry1Ac5,Vip3Aa79,Spp1Aa1	8	Yes	cry1A,cry1I,cry2A,cry2A,cry1A,cry1A,vip3Aa,Spp1A	Lepidoptera;Coleoptera;Rhabditida;Diptera;Hemiptera;Blattodea;Aedes aegypti	*Plutella xylostella;Ostrinia furnacalis;Helicoverpa armigera;Leucinodes orbonalis;Trichoplusia ni;Hyphantria cunea;Spodoptera frugiperda;Pieris rapae;Chilo suppressalis;Leptinotarsa decemlineata;Lobesia botrana;Earias insulana;cotton boll weevil;Meloidogyne incognita;Aedes aegypti;Diaphorina citri;Helicoverpa punctigera;Helicoverpa zea;Chilo partellus;Blattella germanica;Spodoptera litura*
GCF_045557615.1	*Bacillus thuringiensis serovar israelensis*	VCRC-B649	Cry11Aa3,Cry10Aa4,Cry4Ba7,Cyt1Ca1,Cyt1Aa11,Cyt2Ba17,Mpp60Ba3,Mpp60Aa3	8	No	cry11A,cry10A,cry4B,cyt1A,cyt2B,Mpp60B,Mpp60A	Diptera;Coleoptera;Hemiptera;Lepidoptera	*Aedes aegypti;Anopheles albimanus;Culex quinquefasciatu;Culex pipiens;cotton boll weevil;Anopheles gambiae;Diaphorina citri;Culex quinquefasciatus;Culex*

								pipien;Plutella xylostella;Anopheles stephensi;Bradysia difformis
GCF_045557575.1	*Bacillus thuringiensis*	VCRC B647	Cry11Aa3,Cry10Aa4,Cry4Ba7,Cyt1Ca1,Cyt1Aa11,Cyt2Ba17,Mpp60Ba3,Mpp60Aa3	8	No	cry11A,cry10A,cry4B,cyt1A,cyt2B,Mpp60B,Mpp60A	Diptera;Coleoptera;Hemiptera;Lepidoptera	*Aedes aegypti;Anopheles albimanus;Culex quinquefasciatu;Culex pipiens;cotton boll weevil;Anopheles gambiae;Diaphorina citri;Culex quinquefasciatus;Culex pipien;Plutella xylostella;Anopheles stephensi;Bradysia difformis*
GCF_045557285.1	*Bacillus thuringiensis serovar israelensis*	VCRC-B646	Cry11Aa3,Cry10Aa4,Cry4Ba7,Cyt1Ca1,Cyt1Aa11,Cyt2Ba17,Mpp60Ba3,Mpp60Aa3	8	No	cry11A,cry10A,cry4B,cyt1A,cyt2B,Mpp60B,Mpp60A	Diptera;Coleoptera;Hemiptera;Lepidoptera	*Aedes aegypti;Anopheles albimanus;Culex quinquefasciatu;Culex pipiens;cotton boll weevil;Anopheles gambiae;Diaphorina citri;Culex quinquefasciatus;Culex pipien;Plutella xylostella;Anopheles stephensi;Bradysia difformis*
GCF_044903025.1	*Bacillus thuringiensis*	NPDC026629	Cry1Ba8,Mpp5Ab1,Spp1Aa1,Vpa2Bb4,Vpb1Bb2,Vpb1Bc1	6	Yes	cry1B,Mpp5A,Spp1A	Lepidoptera;Hemiptera;Diptera;Coleoptera;Blattodea	*Plutella xylostella;Ostrinia nubilalis;Spodoptera frugiperda;Maruca vitrata;Manduca sexta;cotton boll weevil;Colaphellus bowringi;Leptinotarsa decemlineata;Spodoptera litura;Blattella germanica*
GCF_044769975.1	*Bacillus thuringiensis*	BZRG2	Spp1Aa1	1	Yes	Spp1A	Lepidoptera;Blattodea	*Spodoptera litura;Blattella germanica*

GCF_044769715.1	*Bacillus thuringiensis*	BZRG1	Spp1Aa1	1	Yes	Spp1A	Lepidoptera;Blattodea	*Spodoptera litura;Blattella germanica*
GCF_044769615.1	*Bacillus thuringiensis*	BZRG3	Spp1Aa1	1	Yes	Spp1A	Lepidoptera;Blattodea	*Spodoptera litura;Blattella germanica*
GCF_044761275.1	*Bacillus thuringiensis*	NPDC097802	Spp1Aa1	1	No	Spp1A	Lepidoptera;Blattodea	*Spodoptera litura;Blattella germanica*
GCF_044761265.1	*Bacillus thuringiensis*	NPDC097818	Spp1Aa1	1	Yes	Spp1A	Lepidoptera;Blattodea	*Spodoptera litura;Blattella germanica*
GCF_044761195.1	*Bacillus thuringiensis*	NPDC097837	Spp1Aa1	1	No	Spp1A	Lepidoptera;Blattodea	*Spodoptera litura;Blattella germanica*
GCF_044755965.1	*Bacillus thuringiensis*	NPDC092047	Cry1Aa3,Cry1Ia42,Cry2Aa22,Cry2Ab41,Vip3Aa79,Mpp5Ab1,Spp1Aa1,Vpa2Bb4,Vpb1Bb2,Vpb1Bc1	10	Yes	cry1A,cry1I,cry2A,cry2A,vip3Aa,Mpp5A,Spp1A	Lepidoptera;Coleoptera;Rhabditida;Diptera;Hemiptera;Blattodea;Aedes aegypti	*Plutella xylostella;Ostrinia furnacalis;Helicoverpa armigera;Leucinodes orbonalis;Trichoplusia ni;Hyphantria cunea;Spodoptera frugiperda;Pieris rapae;Chilo suppressalis;Leptinotarsa decemlineata;Lobesia botrana;Earias insulana;cotton boll weevil;Meloidogyne incognita;Aedes aegypti;Diaphorina citri;Helicoverpa punctigera;Helicoverpa zea;Chilo partellus;Blattella*

								germanica;Colaphellus bowringi;Spodoptera litura
GCF_044626355.1	*Bacillus thuringiensis*	T07	Cry1Ia42,Cry2Ab41,Cry9Ea12,Cry1Ca18,Cry1Da2,Cry3Aa6,Cry70Bb1,Vip3Aa79,Spp1Aa1	9	Yes	cry1I,cry2A,Cry9E,cry1C,cry1D,Cry3A,vip3Aa,Spp1A	Coleoptera;Lepidoptera;Rhabditida;Diptera;Hemiptera;Dipteran;Blattodea;Aedes aegypti	*Leptinotarsa decemlineata;Ostrinia furnacalis;Lobesia botrana;Plutella xylostella;Earias insulana;cotton boll weevil;Meloidogyne incognita;Helicoverpa armigera;Aedes aegypti;Diaphorina citri;Agrotis exclamationis;Cydia pomonella;Myzus persicae;Ostrinia nubilalis;Spodoptera exigua;Spodoptera frugiperda;Bombyx mori;Spodoptera litura;Epiphyas postvittana;Tenebrio molitor;Hypera postica;Macrosiphum euphorbiae;Helicoverpa punctigera;Helicoverpa zea;Chilo partellus;Blattella germanica*
GCF_044626315.1	*Bacillus thuringiensis*	T04	Spp1Aa1	1	No	Spp1A	Lepidoptera;Blattodea	*Spodoptera litura;Blattella germanica*
GCF_044626295.1	*Bacillus thuringiensis*	T23	Cry1Ia42,Cry2Ab41,Cry9Ea12,Cry1Ca18,Cry1Ac9,Vip3Aa79,Spp1Aa1	7	Yes	cry1I,cry2A,Cry9E,cry1C,cry1A,vip3Aa,Spp1A	Coleoptera;Lepidoptera;Rhabditida;Diptera;Hemiptera;Dipteran;Blattodea;Aedes aegypti	*Leptinotarsa decemlineata;Ostrinia furnacalis;Lobesia botrana;Plutella xylostella;Earias insulana;cotton boll weevil;Meloidogyne incognita;Helicoverpa armigera;Aedes aegypti;Diaphorina*

								citri;Agrotis exclamationis;Cydia pomonella;Myzus persicae;Leucinodes orbonalis;Trichoplusia ni;Hyphantria cunea;Spodoptera frugiperda;Pieris rapae;Chilo suppressalis;Helicoverpa punctigera;Helicoverpa zea;Chilo partellus;Blattella germanica;Spodoptera litura
GCF_042884125.1	*Bacillus thuringiensis*	NPDC058153	Cry70Ba1,Spp1Aa1,Tpp80Aa2	3	No	Spp1A,Tpp80A	Lepidoptera;Blattodea;Diptera;Hemiptera	*Spodoptera litura;Blattella germanica;Culex pipiens pallens;Culex quinquefasciatus;Aedes aegypti;Anopheles gambiae;Laodelphax striatellus*
GCF_042264985.1	*Bacillus thuringiensis*	NPDC056204	Spp1Aa1	1	Yes	Spp1A	Lepidoptera;Blattodea	*Spodoptera litura;Blattella germanica*
GCF_042262165.1	*Bacillus thuringiensis*	NPDC056426	Spp1Aa1	1	Yes	Spp1A	Lepidoptera;Blattodea	*Spodoptera litura;Blattella germanica*
GCF_042261275.1	*Bacillus thuringiensis*	NPDC056408	Spp1Aa1	1	Yes	Spp1A	Lepidoptera;Blattodea	*Spodoptera litura;Blattella germanica*
GCF_040719735.1	*Bacillus thuringiensis*	RHF1.1-17	Spp1Aa1	1	Yes	Spp1A	Lepidoptera;Blattodea	*Spodoptera litura;Blattella germanica*

GCF_040719715.1	*Bacillus thuringiensis*	RHF2.1-6	Spp1Aa1	1	Yes	Spp1A	Lepidoptera;Blattodea	*Spodoptera litura;Blattella germanica*
GCF_040719695.1	*Bacillus thuringiensis*	RHF1.1-2	Spp1Aa1	1	Yes	Spp1A	Lepidoptera;Blattodea	*Spodoptera litura;Blattella germanica*
GCF_040716475.1	*Bacillus thuringiensis*	RHF4.1-24	Spp1Aa1	1	Yes	Spp1A	Lepidoptera;Blattodea	*Spodoptera litura;Blattella germanica*
GCF_040716445.1	*Bacillus thuringiensis*	RHF5.1-15			Yes			
GCF_040716435.1	*Bacillus thuringiensis*	RHF3.1-21	Spp1Aa1	1	Yes	Spp1A	Lepidoptera;Blattodea	*Spodoptera litura;Blattella germanica*
GCF_040716415.1	*Bacillus thuringiensis*	RHF5.1-28			Yes			
GCF_040712275.1	*Bacillus thuringiensis*	JJ1216	Spp1Aa1	1	Yes	Spp1A	Lepidoptera;Blattodea	*Spodoptera litura;Blattella germanica*
GCF_040492495.1	*Bacillus thuringiensis*	JJ1253	Spp1Aa1	1	Yes	Spp1A	Lepidoptera;Blattodea	*Spodoptera litura;Blattella germanica*
GCF_040337655.1	*Bacillus thuringiensis*	SDF0030	Cry62Aa1	1	No			

GCF_040151435.1	*Bacillus thuringiensis*	NAHVS5F			Yes			
GCF_040151295.1	*Bacillus thuringiensis*	M17VL1H	Spp1Aa1	1	Yes	Spp1A	Lepidoptera;Blattodea	*Spodoptera litura;Blattella germanica*
GCF_040105475.1	*Bacillus thuringiensis*	B4	Cry1Ia42,Cry2Aa22,Cry2Ab41,Vip3Aa79,Spp1Aa1	5	Yes	cry1I,cry2A,cry2A,vip3Aa,Spp1A	Coleoptera;Lepidoptera;Rhabditida;Diptera;Hemiptera;Blattodea;Aedes aegypti	*Leptinotarsa decemlineata;Ostrinia furnacalis;Lobesia botrana;Plutella xylostella;Earias insulana;cotton boll weevil;Meloidogyne incognita;Helicoverpa armigera;Aedes aegypti;Diaphorina citri;Helicoverpa punctigera;Spodoptera frugiperda;Helicoverpa zea;Chilo partellus;Blattella germanica;Spodoptera litura*
GCF_039954905.1	*Bacillus thuringiensis*	9.1	Spp1Aa1	1	Yes	Spp1A	Lepidoptera;Blattodea	*Spodoptera litura;Blattella germanica*
GCF_038886575.1	*Bacillus thuringiensis*	V-CO 3.3	Cry5Ba2	1	Yes	cry5B	Rhabditida	*Caenorhabditis elegans;Ancylostoma ceylanicum;Heligmosomoides polygyrus bakeri;Meloidogyne incognita*
GCF_038561975.1	*Bacillus thuringiensis*	YB6			No			
GCF_038	*Bacillus*	Q3-6	Spp1Aa1	1	Yes	Spp1A	Lepidoptera;Blatto	*Spodoptera litura;Blattella*

039675.1	*thuringiensis*						dea	*germanica*
GCF_037152995.1	*Bacillus thuringiensis*	USMM087	Spp1Aa1	1	Yes	Spp1A	Lepidoptera;Blattodea	*Spodoptera litura;Blattella germanica*
GCF_037152955.1	*Bacillus thuringiensis*	USMM086	Spp1Aa1	1	Yes	Spp1A	Lepidoptera;Blattodea	*Spodoptera litura;Blattella germanica*
GCF_037152875.1	*Bacillus thuringiensis*	USMM088	Cry11Aa3,Cry10Aa4,Cyt1Ca1,Cyt1Aa11,Cyt2Ba17,Mpp60Ba3,Mpp60Aa3	7	No	cry11A,cry10A,cyt1A,cyt2B,Mpp60B,Mpp60A	Diptera;Coleoptera;Hemiptera;Lepidoptera	*Aedes aegypti;Anopheles albimanus;Culex quinquefasciatu;Culex pipiens;cotton boll weevil;Culex quinquefasciatus;Culex pipien;Diaphorina citri;Plutella xylostella;Anopheles stephensi;Anopheles gambiae;Bradysia difformis*
GCF_037145095.1	*Bacillus thuringiensis*	USHLN193	Spp1Aa1	1	Yes	Spp1A	Lepidoptera;Blattodea	*Spodoptera litura;Blattella germanica*
GCF_037075445.1	*Bacillus thuringiensis*	V-AB8.18	Cry5Ba1,App6Aa2,Spp1Aa1	3	Yes	cry5B,App6A,Spp1A	Rhabditida;Lepidoptera;Blattodea	*Caenorhabditis elegans;Ancylostoma ceylanicum;Heligmosomoides polygyrus bakeri;Meloidogyne incognita;Bursaphelenchus xylophilus;Spodoptera litura;Blattella germanica*
GCF_036967125.1	*Bacillus thuringi*	JJ1237	Spp1Aa1	1	Yes	Spp1A	Lepidoptera;Blattodea	*Spodoptera litura;Blattella germanica*

	ensis							
GCF_036967025.1	*Bacillus thuringiensis*	JJ1714	Spp1Aa1	1	Yes	Spp1A	Lepidoptera;Blattodea	*Spodoptera litura;Blattella germanica*
GCF_036966645.1	*Bacillus thuringiensis*	JJ1083	Spp1Aa1	1	Yes	Spp1A	Lepidoptera;Blattodea	*Spodoptera litura;Blattella germanica*
GCF_036963325.1	*Bacillus thuringiensis*	JJ218	Spp1Aa1	1	Yes	Spp1A	Lepidoptera;Blattodea	*Spodoptera litura;Blattella germanica*
GCF_036962385.1	*Bacillus thuringiensis*	JJ499	Spp1Aa1	1	Yes	Spp1A	Lepidoptera;Blattodea	*Spodoptera litura;Blattella germanica*
GCF_036621355.1	*Bacillus thuringiensis*	B20	Spp1Aa1	1	Yes	Spp1A	Lepidoptera;Blattodea	*Spodoptera litura;Blattella germanica*
GCF_036621335.1	*Bacillus thuringiensis*	B21	Spp1Aa1	1	No	Spp1A	Lepidoptera;Blattodea	*Spodoptera litura;Blattella germanica*
GCF_036621275.1	*Bacillus thuringiensis*	B6	Bmp1-other	1	No			
GCF_036350635.1	*Bacillus thuringiensis*	GS1	Spp1Aa1	1	Yes	Spp1A	Lepidoptera;Blattodea	*Spodoptera litura;Blattella germanica*
GCF_036219465.1	*Bacillus thuringi*	B-345	Spp1Aa1	1	Yes	Spp1A	Lepidoptera;Blattodea	*Spodoptera litura;Blattella germanica*

	ensis							
GCF_036219285.1	*Bacillus thuringiensis*	B-1868	Spp1Aa1	1	Yes	Spp1A	Lepidoptera;Blattodea	*Spodoptera litura;Blattella germanica*
GCF_036219265.1	*Bacillus thuringiensis*	B-2172	Cry1Ba8,Cry1Ab9,Mpp5Ab1,Spp1Aa1,Vpa2Bb4,Vpb1Bb2,Vpb1Bc1	7	Yes	cry1B,cry1A,Mpp5A,Spp1A	Lepidoptera;Hemiptera;Diptera;Coleoptera;Blattodea	*Plutella xylostella;Ostrinia nubilalis;Spodoptera frugiperda;Maruca vitrata;Manduca sexta;cotton boll weevil;Ostrinia furnacalis;Helicoverpa armigera;Leucinodes orbonalis;Trichoplusia ni;Hyphantria cunea;Pieris rapae;Chilo suppressalis;Colaphellus bowringi;Leptinotarsa decemlineata;Spodoptera litura;Blattella germanica*
GCF_036219145.1	*Bacillus thuringiensis*	B-3439	Spp1Aa1	1	No	Spp1A	Lepidoptera;Blattodea	*Spodoptera litura;Blattella germanica*
GCF_036219085.1	*Bacillus thuringiensis*	B-4048			No			
GCF_036219065.1	*Bacillus thuringiensis*	B-4039	Cry1Ba8,Cry1Ab9,Mpp5Ab1,Spp1Aa1,Vpa2Bb4	5	Yes	cry1B,cry1A,Mpp5A,Spp1A	Lepidoptera;Hemiptera;Diptera;Coleoptera;Blattodea	*Plutella xylostella;Ostrinia nubilalis;Spodoptera frugiperda;Maruca vitrata;Manduca sexta;cotton boll weevil;Ostrinia furnacalis;Helicoverpa armigera;Leucinodes*

								orbonalis;Trichoplusia ni;Hyphantria cunea;Pieris rapae;Chilo suppressalis;Colaphellus bowringi;Leptinotarsa decemlineata;Spodoptera litura;Blattella germanica
GCF_036219045.1	*Bacillus thuringiensis*	B-4058	Cry1Ea7,Spp1Aa1	2	No	cry1E,Spp1A	Lepidoptera;Hemiptera;Rhabditida;Blattodea	*Spodoptera exigua;Spodoptera frugiperda;Malacosoma disstria;Bursaphelenchus xylophilus;Spodoptera litura;Blattella germanica*
GCF_036219025.1	*Bacillus thuringiensis*	B-4059	Cry1Bb1,Cry1Ab24,Cry1Ja2,Cry1Da3,Cry1Hb2,Cry1Id2,Cry1Nb1,Cry2Ad1,Vip3Af3,App4Aa1,Spp1Aa1,Vpa2Ac2,Vpb1Ca1	13	Yes	cry1B,cry1A,cry1J,cry1D,cry1H,cry1I,cry1N,cry2A,vip3Af,App4A,Spp1A	Lepidoptera;Hemiptera;Diptera;Coleoptera;Rhabditida;Blattodea	*Plutella xylostella;Ostrinia nubilalis;Spodoptera frugiperda;Maruca vitrata;Manduca sexta;cotton boll weevil;Ostrinia furnacalis;Helicoverpa armigera;Leucinodes orbonalis;Trichoplusia ni;Hyphantria cunea;Pieris rapae;Chilo suppressalis;Lobesia botrana;Diabrotica undecimpunctata howardi;Spodoptera exigua;Bombyx mori;Spodoptera litura;Epiphyas postvittana;Leptinotarsa decemlineata;Earias insulana;Meloidogyne incognita;Colaphellus bowringi;Aedes aegypti;Diaphorina*

								citri;Agrotis ipsilon;Laodelphax striatellus;Blattella germanica
GCF_036218885.1	*Bacillus thuringiensis*	B-4557	Cry11Aa3,Cry10Aa4,Cyt1Ca1,Cyt1Aa11,Cyt2Ba17,Mpp60Ba3,Mpp60Aa3	7	No	cry11A,cry10A,cyt1A,cyt2B,Mpp60B,Mpp60A	Diptera;Coleoptera;Hemiptera;Lepidoptera	*Aedes aegypti;Anopheles albimanus;Culex quinquefasciatu;Culex pipiens;cotton boll weevil;Culex quinquefasciatus;Culex pipien;Diaphorina citri;Plutella xylostella;Anopheles stephensi;Anopheles gambiae;Bradysia difformis*
GCF_036218785.1	*Bacillus thuringiensis*	B-14591	App4Aa1,Tpp80Ad1,Xpp22Ba1	3	Yes	App4A,Tpp80A,Xpp22B	Lepidoptera;Hemiptera;Diptera;Coleoptera	*Plutella xylostella;Laodelphax striatellus;Culex pipiens pallens;Culex quinquefasciatus;Aedes aegypti;Anopheles gambiae;Anthonomus grandis*
GCF_036218445.1	*Bacillus thuringiensis*	B-14966	Cry11Aa3,Cry10Aa4,Cyt1Ca1,Cyt1Aa11,Cyt2Ba17,Mpp60Ba3,Mpp60Aa3	7	No	cry11A,cry10A,cyt1A,cyt2B,Mpp60B,Mpp60A	Diptera;Coleoptera;Hemiptera;Lepidoptera	*Aedes aegypti;Anopheles albimanus;Culex quinquefasciatu;Culex pipiens;cotton boll weevil;Culex quinquefasciatus;Culex pipien;Diaphorina citri;Plutella xylostella;Anopheles stephensi;Anopheles gambiae;Bradysia difformis*
GCF_036218405.1	*Bacillus thuringi*	B-14983	Cry11Aa3,Cry10Aa4,Cyt1Ca1,Cyt1Aa11,Cyt2	7	No	cry11A,cry10A,cyt1A,cyt2B,Mpp60B,Mpp6	Diptera;Coleoptera;Hemiptera;Lepid	*Aedes aegypti;Anopheles albimanus;Culex*

	ensis		Ba17,Mpp60Ba3,Mpp60Aa3			0A	optera	*quinquefasciatu;Culex pipiens;cotton boll weevil;Culex quinquefasciatus;Culex pipien;Diaphorina citri;Plutella xylostella;Anopheles stephensi;Anopheles gambiae;Bradysia difformis*
GCF_036218365.1	*Bacillus thuringiensis*	B-14986	Cry10Aa4,Cyt1Ca1,Mpp60Ba3,Mpp60Aa3	4	No	cry10A,Mpp60B,Mpp60A	Diptera;Coleoptera	*Aedes aegypti;cotton boll weevil;Culex quinquefasciatus*
GCF_036218305.1	*Bacillus thuringiensis*	B-14998	Cry11Aa3,Cry10Aa4,Cyt1Ca1,Cyt1Aa11,Cyt2Ba17,Mpp60Ba3,Mpp60Aa3	7	No	cry11A,cry10A,cyt1A,cyt2B,Mpp60B,Mpp60A	Diptera;Coleoptera;Hemiptera;Lepidoptera	*Aedes aegypti;Anopheles albimanus;Culex quinquefasciatu;Culex pipiens;cotton boll weevil;Culex quinquefasciatus;Culex pipien;Diaphorina citri;Plutella xylostella;Anopheles stephensi;Anopheles gambiae;Bradysia difformis*
GCF_036218285.1	*Bacillus thuringiensis*	B-14990	Cry5Ca2,Cry5Ad1,Spp1Aa1	3	Yes	cry5C,cry5A,Spp1A	Rhabditida;Lepidoptera;Hymenoptera;Blattodea	*Meloidogyne incognita;Caenorhabditis elegans;Haemonchus contortus;Trichostrongylus colubriformis;Ostertagia circumcincta;Diprion pini;Cephacia abietis;Helicoverpa armigera;Spodoptera litura;Blattella germanica*

GCF_036218245.1	*Bacillus thuringiensis*	B-14999	Spp1Aa1	1	Yes	Spp1A	Lepidoptera;Blattodea	*Spodoptera litura;Blattella germanica*
GCF_036218185.1	*Bacillus thuringiensis*	B-23020	Cry11Aa3,Cry10Aa4,Cyt1Ca1,Cyt1Aa11,Cyt2Ba17,Mpp60Ba3,Mpp60Aa3	7	No	cry11A,cry10A,cyt1A,cyt2B,Mpp60B,Mpp60A	Diptera;Coleoptera;Hemiptera;Lepidoptera	*Aedes aegypti;Anopheles albimanus;Culex quinquefasciatu;Culex pipiens;cotton boll weevil;Culex quinquefasciatus;Culex pipien;Diaphorina citri;Plutella xylostella;Anopheles stephensi;Anopheles gambiae;Bradysia difformis*
GCF_036218145.1	*Bacillus thuringiensis*	B-23025	Cry1Bb1,Cry1Id2,Cry1Nb1,Cry2Ad1,Vip3Af3,App4Aa1,Spp1Aa1,Vpa2Ac2,Vpb1Ca1	9	Yes	cry1B,cry1I,cry1N,cry2A,vip3Af,App4A,Spp1A	Lepidoptera;Hemiptera;Diptera;Coleoptera;Rhabditida;Blattodea	*Plutella xylostella;Ostrinia nubilalis;Spodoptera frugiperda;Maruca vitrata;Manduca sexta;cotton boll weevil;Leptinotarsa decemlineata;Ostrinia furnacalis;Lobesia botrana;Earias insulana;Meloidogyne incognita;Colaphellus bowringi;Helicoverpa armigera;Aedes aegypti;Diaphorina citri;Agrotis ipsilon;Spodoptera exigua;Chilo suppressalis;Laodelphax striatellus;Spodoptera litura;Blattella germanica*
GCF_036	*Bacillus*	B-230	Cry11Aa3,Cry10Aa4,C	7	No	cry11A,cry10A,cyt1A	Diptera;Coleopter	*Aedes aegypti;Anopheles*

218125.1	*thuringiensis*	43	yt1Ca1,Cyt1Aa11,Cyt2Ba17,Mpp60Ba3,Mpp60Aa3			,cyt2B,Mpp60B,Mpp60A	a;Hemiptera;Lepidoptera	*albimanus;Culex quinquefasciatu;Culex pipiens;cotton boll weevil;Culex quinquefasciatus;Culex pipien;Diaphorina citri;Plutella xylostella;Anopheles stephensi;Anopheles gambiae;Bradysia difformis*
GCF_036218085.1	*Bacillus thuringiensis*	B-23143	Cry1Ie3,Cry1Na2,Cry9Aa4,Vip3Aa51,Spp1Aa1	5	Yes	cry1I,cry1N,Cry9A,vip3Aa,Spp1A	Coleoptera;Lepidoptera;Rhabditida;Blattodea;Aedes aegypti	*Leptinotarsa decemlineata;Ostrinia furnacalis;Lobesia botrana;Plutella xylostella;Earias insulana;cotton boll weevil;Meloidogyne incognita;Colaphellus bowringi;Pieris brassicae;Phthorimaea operculella;Chilo suppressalis;Helicoverpa punctigera;Spodoptera frugiperda;Helicoverpa zea;Chilo partellus;Blattella germanica;Spodoptera litura*
GCF_036218065.1	*Bacillus thuringiensis*	B-23005			No			
GCF_036218045.1	*Bacillus thuringiensis*	B-23199	Spp1Aa1	1	No	Spp1A	Lepidoptera;Blattodea	*Spodoptera litura;Blattella germanica*
GCF_036	*Bacillus*	B-230	Cry11Aa3,Cry10Aa4,C	5	No	cry11A,cry10A,cyt1A	Diptera;Coleopter	*Aedes aegypti;Anopheles*

218005.1	*thuringiensis*	06	yt1Ca1,Cyt1Aa11,Cyt2Ba17			,cyt2B	a;Hemiptera;Lepidoptera	*albimanus;Culex quinquefasciatu;Culex pipiens;cotton boll weevil;Culex quinquefasciatus;Culex pipien;Diaphorina citri;Plutella xylostella;Anopheles stephensi;Anopheles gambiae;Bradysia difformis*
GCF_036217985.1	*Bacillus thuringiensis*	B-23014	Spp1Aa1	1	Yes	Spp1A	Lepidoptera;Blattodea	*Spodoptera litura;Blattella germanica*
GCF_036217945.1	*Bacillus thuringiensis*	B-23017	Cry11Aa3,Cry10Aa4,Cyt1Ca1,Cyt1Aa11,Cyt2Ba17,Mpp60Ba3,Mpp60Aa3	7	No	cry11A,cry10A,cyt1A,cyt2B,Mpp60B,Mpp60A	Diptera;Coleoptera;Hemiptera;Lepidoptera	*Aedes aegypti;Anopheles albimanus;Culex quinquefasciatu;Culex pipiens;cotton boll weevil;Culex quinquefasciatus;Culex pipien;Diaphorina citri;Plutella xylostella;Anopheles stephensi;Anopheles gambiae;Bradysia difformis*
GCF_036217925.1	*Bacillus thuringiensis*	B-23022	Cry1Bb1,Cry1Id2,Cry1Nb1,Cry2Ad1,Cry1Hb1,Vip3Af3,App4Aa1,Spp1Aa1,Vpa2Ac2,Vpb1Ca1	10	Yes	cry1B,cry1I,cry1N,cry2A,cry1H,vip3Af,App4A,Spp1A	Lepidoptera;Hemiptera;Diptera;Coleoptera;Rhabditida;Blattodea	*Plutella xylostella;Ostrinia nubilalis;Spodoptera frugiperda;Maruca vitrata;Manduca sexta;cotton boll weevil;Leptinotarsa decemlineata;Ostrinia furnacalis;Lobesia botrana;Earias insulana;Meloidogyne incognita;Colaphellus*

								bowringi;Helicoverpa armigera;Aedes aegypti;Diaphorina citri;Spodoptera exigua;Agrotis ipsilon;Chilo suppressalis;Laodelphax striatellus;Spodoptera litura;Blattella germanica
GCF_036217905.1	*Bacillus thuringiensis*	B-23023	Cry1Id2,Cry1Nb1,Cry2Ad1,Cry1Bb1,Vip3Af3,App4Aa1,Spp1Aa1,Vpa2Ac2,Vpb1Ca1	9	Yes	cry1I,cry1N,cry2A,cry1B,vip3Af,App4A,Spp1A	Coleoptera;Lepidoptera;Rhabditida;Diptera;Hemiptera;Blattodea	*Leptinotarsa decemlineata;Ostrinia furnacalis;Lobesia botrana;Plutella xylostella;Earias insulana;cotton boll weevil;Meloidogyne incognita;Colaphellus bowringi;Helicoverpa armigera;Aedes aegypti;Diaphorina citri;Ostrinia nubilalis;Spodoptera frugiperda;Maruca vitrata;Manduca sexta;Agrotis ipsilon;Spodoptera exigua;Chilo suppressalis;Laodelphax striatellus;Spodoptera litura;Blattella germanica*
GCF_036217825.1	*Bacillus thuringiensis*	B-23126	Spp1Aa1	1	Yes	Spp1A	Lepidoptera;Blattodea	*Spodoptera litura;Blattella germanica*
GCF_036217805.1	*Bacillus thuringiensis*	B-23102	Cry11Aa3,Cry10Aa4,Cyt1Ca1,Cyt1Aa11,Cyt2Ba17,Mpp60Ba3,Mpp60Aa3	7	No	cry11A,cry10A,cyt1A,cyt2B,Mpp60B,Mpp60A	Diptera;Coleoptera;Hemiptera;Lepidoptera	*Aedes aegypti;Anopheles albimanus;Culex quinquefasciatu;Culex pipiens;cotton boll weevil;Culex*

								quinquefasciatus;Culex pipien;Diaphorina citri;Plutella xylostella;Anopheles stephensi;Anopheles gambiae;Bradysia difformis
GCF_036217765.1	*Bacillus thuringiensis*	B-23173	Cry1Bb1,Cry1Ab24,Cry1Ja2,Cry1Da3,Cry1Hb2,Cry1Id2,Cry1Nb1,Cry2Ad1,Cry1Ka1,Cry1Ib5,Cry1Fb4,Vip3Af3,Spp1Aa1,Vpa2Ac2,Vpb1Ca1	15	No	cry1B,cry1A,cry1J,cry1D,cry1H,cry1I,cry1N,cry2A,cry1K,cry1I,cry1F,vip3Af,Spp1A	Lepidoptera;Hemiptera;Diptera;Coleoptera;Rhabditida;Blattodea	*Plutella xylostella;Ostrinia nubilalis;Spodoptera frugiperda;Maruca vitrata;Manduca sexta;cotton boll weevil;Ostrinia furnacalis;Helicoverpa armigera;Leucinodes orbonalis;Trichoplusia ni;Hyphantria cunea;Pieris rapae;Chilo suppressalis;Lobesia botrana;Diabrotica undecimpunctata howardi;Spodoptera exigua;Bombyx mori;Spodoptera litura;Epiphyas postvittana;Leptinotarsa decemlineata;Earias insulana;Meloidogyne incognita;Colaphellus bowringi;Aedes aegypti;Diaphorina citri;Artogeia rapae;Agrotis ipsilon;Diabrotica virgifera virgifera;Blattella germanica*
GCF_036217705.1	*Bacillus thuringiensis*	B-23232	Cry11Aa3,Cry10Aa4,Cyt1Ca1,Cyt1Aa11,Cyt2Ba17	5	No	cry11A,cry10A,cyt1A,cyt2B	Diptera;Coleoptera;Hemiptera;Lepidoptera	*Aedes aegypti;Anopheles albimanus;Culex quinquefasciatu;Culex pipiens;cotton*

								boll weevil;Culex quinquefasciatus;Culex pipien;Diaphorina citri;Plutella xylostella;Anopheles stephensi;Anopheles gambiae;Bradysia difformis
GCF_036217625.1	*Bacillus thuringiensis*	B-23250	Cry11Aa3,Cry10Aa4,Cyt1Ca1,Cyt1Aa11,Cyt2Ba17,Mpp60Ba3,Mpp60Aa3	7	No	cry11A,cry10A,cyt1A,cyt2B,Mpp60B,Mpp60A	Diptera;Coleoptera;Hemiptera;Lepidoptera	*Aedes aegypti;Anopheles albimanus;Culex quinquefasciatu;Culex pipiens;cotton boll weevil;Culex quinquefasciatus;Culex pipien;Diaphorina citri;Plutella xylostella;Anopheles stephensi;Anopheles gambiae;Bradysia difformis*
GCF_036217585.1	*Bacillus thuringiensis*	B-23251	Cry11Aa3,Cry10Aa4,Cyt1Ca1,Cyt1Aa11,Cyt2Ba17,Mpp60Ba3,Mpp60Aa3	7	No	cry11A,cry10A,cyt1A,cyt2B,Mpp60B,Mpp60A	Diptera;Coleoptera;Hemiptera;Lepidoptera	*Aedes aegypti;Anopheles albimanus;Culex quinquefasciatu;Culex pipiens;cotton boll weevil;Culex quinquefasciatus;Culex pipien;Diaphorina citri;Plutella xylostella;Anopheles stephensi;Anopheles gambiae;Bradysia difformis*
GCF_036217545.1	*Bacillus thuringiensis*	B-23254	Cry11Aa3,Cry10Aa4,Cyt1Ca1,Cyt1Aa11,Cyt2Ba17,Mpp60Ba3,Mpp60Aa3	7	No	cry11A,cry10A,cyt1A,cyt2B,Mpp60B,Mpp60A	Diptera;Coleoptera;Hemiptera;Lepidoptera	*Aedes aegypti;Anopheles albimanus;Culex quinquefasciatu;Culex pipiens;cotton boll weevil;Culex*

								quinquefasciatus;Culex pipien;Diaphorina citri;Plutella xylostella;Anopheles stephensi;Anopheles gambiae;Bradysia difformis
GCF_036217485.1	*Bacillus thuringiensis*	B-23363	Cry11Aa3,Cry10Aa4,Cyt1Ca1,Cyt1Aa11,Cyt2Ba17,Mpp60Ba3,Mpp60Aa3	7	No	cry11A,cry10A,cyt1A,cyt2B,Mpp60B,Mpp60A	Diptera;Coleoptera;Hemiptera;Lepidoptera	*Aedes aegypti;Anopheles albimanus;Culex quinquefasciatu;Culex pipiens;cotton boll weevil;Culex quinquefasciatus;Culex pipien;Diaphorina citri;Plutella xylostella;Anopheles stephensi;Anopheles gambiae;Bradysia difformis*
GCF_036217465.1	*Bacillus thuringiensis*	B-23368	Cry11Aa3,Cry10Aa4,Cyt1Ca1,Cyt1Aa11,Cyt2Ba17	5	No	cry11A,cry10A,cyt1A,cyt2B	Diptera;Coleoptera;Hemiptera;Lepidoptera	*Aedes aegypti;Anopheles albimanus;Culex quinquefasciatu;Culex pipiens;cotton boll weevil;Culex quinquefasciatus;Culex pipien;Diaphorina citri;Plutella xylostella;Anopheles stephensi;Anopheles gambiae;Bradysia difformis*
GCF_036217405.1	*Bacillus thuringiensis*	B-23377	Cry11Aa3,Cry10Aa4,Cyt1Ca1,Cyt1Aa11,Cyt2Ba17,Mpp60Ba3,Mpp60Aa3	7	No	cry11A,cry10A,cyt1A,cyt2B,Mpp60B,Mpp60A	Diptera;Coleoptera;Hemiptera;Lepidoptera	*Aedes aegypti;Anopheles albimanus;Culex quinquefasciatu;Culex pipiens;cotton boll weevil;Culex quinquefasciatus;Culex*

								pipien;Diaphorina citri;Plutella xylostella;Anopheles stephensi;Anopheles gambiae;Bradysia difformis
GCF_036217265.1	*Bacillus thuringiensis*	B-23416	Spp1Aa1,Xpp22Bb1	2	No	Spp1A,Xpp22B	Lepidoptera;Blattodea;Coleoptera	*Spodoptera litura;Blattella germanica;Anthonomus grandis;Plutella xylostella*
GCF_036217185.1	*Bacillus thuringiensis*	B-23433	Cry10Aa4,Cry11Aa3,Cyt1Ca1,Cyt1Aa11,Cyt2Ba17	5	No	cry10A,cry11A,cyt1A,cyt2B	Diptera;Coleoptera;Hemiptera;Lepidoptera	*Aedes aegypti;cotton boll weevil;Anopheles albimanus;Culex quinquefasciatu;Culex pipiens;Culex quinquefasciatus;Culex pipien;Diaphorina citri;Plutella xylostella;Anopheles stephensi;Anopheles gambiae;Bradysia difformis*
GCF_036217145.1	*Bacillus thuringiensis*	B-23435	Cry11Aa3,Cry10Aa4,Cyt1Ca1,Cyt1Aa11,Cyt2Ba17,Mpp60Ba3,Mpp60Aa3	7	No	cry11A,cry10A,cyt1A,cyt2B,Mpp60B,Mpp60A	Diptera;Coleoptera;Hemiptera;Lepidoptera	*Aedes aegypti;Anopheles albimanus;Culex quinquefasciatu;Culex pipiens;cotton boll weevil;Culex quinquefasciatus;Culex pipien;Diaphorina citri;Plutella xylostella;Anopheles stephensi;Anopheles gambiae;Bradysia difformis*
GCF_036217125.1	*Bacillus thuringiensis*	B-23436	Cry1Ab24,Cry1Da3,Cry1Hb2,Cry1Id2,Cry1Nb1,Cry2Ad1,Cry1Ka1,Cry1Ib5,Cry1Fb4,Cry1Bb	14	No	cry1A,cry1D,cry1H,cry1I,cry1N,cry2A,cry1K,cry1I,cry1F,cry1B,cry1J,Spp1A	Lepidoptera;Coleoptera;Rhabditida;Diptera;Hemiptera;Blattodea	*Plutella xylostella;Ostrinia furnacalis;Helicoverpa armigera;Leucinodes orbonalis;Trichoplusia ni;Hyphantria*

			1,Cry1Ja2,Spp1Aa1,Vpa2Ac2,Vpb1Ca1					*cunea;Spodoptera frugiperda;Pieris rapae;Chilo suppressalis;Ostrinia nubilalis;Spodoptera exigua;Bombyx mori;Spodoptera litura;Epiphyas postvittana;Leptinotarsa decemlineata;Lobesia botrana;Earias insulana;cotton boll weevil;Meloidogyne incognita;Colaphellus bowringi;Aedes aegypti;Diaphorina citri;Artogeia rapae;Agrotis ipsilon;Diabrotica virgifera virgifera;Maruca vitrata;Manduca sexta;Diabrotica undecimpunctata howardi;Blattella germanica*
GCF_036217085.1	*Bacillus thuringiensis*	B-23451			No			
GCF_036217065.1	*Bacillus thuringiensis*	B-23452	Cry11Aa3,Cry10Aa4,Cyt1Ca1,Cyt1Aa11,Cyt2Ba17,Mpp60Ba3,Mpp60Aa3	7	No	cry11A,cry10A,cyt1A,cyt2B,Mpp60B,Mpp60A	Diptera;Coleoptera;Hemiptera;Lepidoptera	*Aedes aegypti;Anopheles albimanus;Culex quinquefasciatu;Culex pipiens;cotton boll weevil;Culex quinquefasciatus;Culex pipien;Diaphorina citri;Plutella xylostella;Anopheles stephensi;Anopheles gambiae;Bradysia difformis*
GCF_036	*Bacillus*	B-239	Spp1Aa1	1	Yes	Spp1A	Lepidoptera;Blatto	*Spodoptera litura;Blattella*

217025.1	*thuringiensis*	65					dea	*germanica*
GCF_036217005.1	*Bacillus thuringiensis*	B-41006	Spp1Aa1	1	Yes	Spp1A	Lepidoptera;Blattodea	*Spodoptera litura;Blattella germanica*
GCF_036216765.1	*Bacillus thuringiensis*	B-51075	Spp1Aa1	1	Yes	Spp1A	Lepidoptera;Blattodea	*Spodoptera litura;Blattella germanica*
GCF_036216705.1	*Bacillus thuringiensis*	B-59759	Spp1Aa1	1	Yes	Spp1A	Lepidoptera;Blattodea	*Spodoptera litura;Blattella germanica*
GCF_036216685.1	*Bacillus thuringiensis*	BD-37	Cry1Aa9,Spp1Aa1	2	Yes	cry1A,Spp1A	Lepidoptera;Blattodea	*Plutella xylostella;Ostrinia furnacalis;Helicoverpa armigera;Leucinodes orbonalis;Trichoplusia ni;Hyphantria cunea;Spodoptera frugiperda;Pieris rapae;Chilo suppressalis;Spodoptera litura;Blattella germanica*
GCF_036216605.1	*Bacillus thuringiensis*	BD-340	Spp1Aa1	1	Yes	Spp1A	Lepidoptera;Blattodea	*Spodoptera litura;Blattella germanica*
GCF_036216545.1	*Bacillus thuringiensis*	BD-353	Spp1Aa1	1	No	Spp1A	Lepidoptera;Blattodea	*Spodoptera litura;Blattella germanica*
GCF_036216505.1	*Bacillus thuringiensis*	BD-354	Spp1Aa1	1	No	Spp1A	Lepidoptera;Blattodea	*Spodoptera litura;Blattella germanica*

GCF_036216365.1	*Bacillus thuringiensis*	BD-365	Spp1Aa1	1	Yes	Spp1A	Lepidoptera;Blattodea	*Spodoptera litura;Blattella germanica*
GCF_036216345.1	*Bacillus thuringiensis*	BD-366	Spp1Aa1	1	No	Spp1A	Lepidoptera;Blattodea	*Spodoptera litura;Blattella germanica*
GCF_036216305.1	*Bacillus thuringiensis*	BD-369	Spp1Aa1	1	Yes	Spp1A	Lepidoptera;Blattodea	*Spodoptera litura;Blattella germanica*
GCF_036216285.1	*Bacillus thuringiensis*	BD-370	Spp1Aa1	1	No	Spp1A	Lepidoptera;Blattodea	*Spodoptera litura;Blattella germanica*
GCF_036216205.1	*Bacillus thuringiensis*	BD-494	Spp1Aa1	1	No	Spp1A	Lepidoptera;Blattodea	*Spodoptera litura;Blattella germanica*
GCF_036216185.1	*Bacillus thuringiensis*	BD-501	Spp1Aa1	1	Yes	Spp1A	Lepidoptera;Blattodea	*Spodoptera litura;Blattella germanica*
GCF_036216045.1	*Bacillus thuringiensis*	HD-2	Cry1Ba8,Cry1Ab9,Mpp5Ab1,Spp1Aa1	4	Yes	cry1B,cry1A,Mpp5A,Spp1A	Lepidoptera;Hemiptera;Diptera;Coleoptera;Blattodea	*Plutella xylostella;Ostrinia nubilalis;Spodoptera frugiperda;Maruca vitrata;Manduca sexta;cotton boll weevil;Ostrinia furnacalis;Helicoverpa armigera;Leucinodes orbonalis;Trichoplusia ni;Hyphantria cunea;Pieris rapae;Chilo suppressalis;Colaphellus bowringi;Leptinotarsa*

								decemlineata;Spodoptera litura;Blattella germanica
GCF_036216005.1	*Bacillus thuringiensis*	HD-15	Cry1Aa3,Cry1Ia42,Cry2Aa22,Cry2Ab41,Vip3Aa79,Mpp5Ab1,Spp1Aa1,Vpa2Bb4	8	Yes	cry1A,cry1I,cry2A,cry2A,vip3Aa,Mpp5A,Spp1A	Lepidoptera;Coleoptera;Rhabditida;Diptera;Hemiptera;Blattodea;Aedes aegypti	*Plutella xylostella;Ostrinia furnacalis;Helicoverpa armigera;Leucinodes orbonalis;Trichoplusia ni;Hyphantria cunea;Spodoptera frugiperda;Pieris rapae;Chilo suppressalis;Leptinotarsa decemlineata;Lobesia botrana;Earias insulana;cotton boll weevil;Meloidogyne incognita;Aedes aegypti;Diaphorina citri;Helicoverpa punctigera;Helicoverpa zea;Chilo partellus;Blattella germanica;Colaphellus bowringi;Spodoptera litura*
GCF_036215985.1	*Bacillus thuringiensis*	HD-17	Cry1Aa3,Cry1Ia42,Cry2Aa22,Cry2Ab41,Vip3Aa79,Mpp5Ab1,Spp1Aa1,Vpb1Bc1	8	Yes	cry1A,cry1I,cry2A,cry2A,vip3Aa,Mpp5A,Spp1A	Lepidoptera;Coleoptera;Rhabditida;Diptera;Hemiptera;Blattodea;Aedes aegypti	*Plutella xylostella;Ostrinia furnacalis;Helicoverpa armigera;Leucinodes orbonalis;Trichoplusia ni;Hyphantria cunea;Spodoptera frugiperda;Pieris rapae;Chilo suppressalis;Leptinotarsa decemlineata;Lobesia botrana;Earias insulana;cotton boll weevil;Meloidogyne incognita;Aedes aegypti;Diaphorina citri;Helicoverpa*

								punctigera;Helicoverpa zea;Chilo partellus;Blattella germanica;Colaphellus bowringi;Spodoptera litura
GCF_036215965.1	*Bacillus thuringiensis*	HD-18	Mpp5Ab1,Spp1Aa1,Vpa2Bb4	3	Yes	Mpp5A,Spp1A	Coleoptera;Lepidoptera;Blattodea	*Colaphellus bowringi;Leptinotarsa decemlineata;Spodoptera litura;Blattella germanica*
GCF_036215945.1	*Bacillus thuringiensis*	HD-28	Cry1Ba8,Spp1Aa1,Vpa2Bb4	3	Yes	cry1B,Spp1A	Lepidoptera;Hemiptera;Diptera;Coleoptera;Blattodea	*Plutella xylostella;Ostrinia nubilalis;Spodoptera frugiperda;Maruca vitrata;Manduca sexta;cotton boll weevil;Spodoptera litura;Blattella germanica*
GCF_036215905.1	*Bacillus thuringiensis*	HD-39	Cry1Aa3,Cry1Ia42,Cry2Aa22,Cry2Ab15,Vip3Aa79,Mpp5Ab1,Spp1Aa1,Vpa2Bb4	8	Yes	cry1A,cry1I,cry2A,cry2A,vip3Aa,Mpp5A,Spp1A	Lepidoptera;Coleoptera;Rhabditida;Diptera;Hemiptera;Blattodea;Aedes aegypti	*Plutella xylostella;Ostrinia furnacalis;Helicoverpa armigera;Leucinodes orbonalis;Trichoplusia ni;Hyphantria cunea;Spodoptera frugiperda;Pieris rapae;Chilo suppressalis;Leptinotarsa decemlineata;Lobesia botrana;Earias insulana;cotton boll weevil;Meloidogyne incognita;Aedes aegypti;Diaphorina citri;Helicoverpa punctigera;Helicoverpa zea;Chilo partellus;Blattella germanica;Colaphellus bowringi;Spodoptera litura*
GCF_036	*Bacillus*	HD-43	Spp1Aa1	1	Yes	Spp1A	Lepidoptera;Blatto	*Spodoptera litura;Blattella*

215885.1	*thuringiensis*						dea	*germanica*
GCF_036215865.1	*Bacillus thuringiensis*	HD-48	Cry1Aa9,Spp1Aa1	2	Yes	cry1A,Spp1A	Lepidoptera;Blattodea	*Plutella xylostella;Ostrinia furnacalis;Helicoverpa armigera;Leucinodes orbonalis;Trichoplusia ni;Hyphantria cunea;Spodoptera frugiperda;Pieris rapae;Chilo suppressalis;Spodoptera litura;Blattella germanica*
GCF_036215845.1	*Bacillus thuringiensis*	HD-54	Cry1Ba8,Cry1Ab9,Mpp5Ab1,Spp1Aa1,Vpa2Bb4,Vpb1Bb2,Vpb1Bc1	7	Yes	cry1B,cry1A,Mpp5A,Spp1A	Lepidoptera;Hemiptera;Diptera;Coleoptera;Blattodea	*Plutella xylostella;Ostrinia nubilalis;Spodoptera frugiperda;Maruca vitrata;Manduca sexta;cotton boll weevil;Ostrinia furnacalis;Helicoverpa armigera;Leucinodes orbonalis;Trichoplusia ni;Hyphantria cunea;Pieris rapae;Chilo suppressalis;Colaphellus bowringi;Leptinotarsa decemlineata;Spodoptera litura;Blattella germanica*
GCF_036215825.1	*Bacillus thuringiensis*	HD-51	Cry1Ba8,Spp1Aa1	2	Yes	cry1B,Spp1A	Lepidoptera;Hemiptera;Diptera;Coleoptera;Blattodea	*Plutella xylostella;Ostrinia nubilalis;Spodoptera frugiperda;Maruca vitrata;Manduca sexta;cotton boll weevil;Spodoptera litura;Blattella germanica*
GCF_036215805.1	*Bacillus thuringi*	HD-57	Cry1Ba8,Cry1Ab9,Mpp5Ab1,Spp1Aa1,Vpa2Bb	5	Yes	cry1B,cry1A,Mpp5A,Spp1A	Lepidoptera;Hemiptera;Diptera;Cole	*Plutella xylostella;Ostrinia nubilalis;Spodoptera*

	ensis		4				optera;Blattodea	*frugiperda;Maruca vitrata;Manduca sexta;cotton boll weevil;Ostrinia furnacalis;Helicoverpa armigera;Leucinodes orbonalis;Trichoplusia ni;Hyphantria cunea;Pieris rapae;Chilo suppressalis;Colaphellus bowringi;Leptinotarsa decemlineata;Spodoptera litura;Blattella germanica*
GCF_036215785.1	*Bacillus thuringiensis*	HD-61	Cry1Ba8,Cry1Ab9,Spp1Aa1	3	Yes	cry1B,cry1A,Spp1A	Lepidoptera;Hemiptera;Diptera;Coleoptera;Blattodea	*Plutella xylostella;Ostrinia nubilalis;Spodoptera frugiperda;Maruca vitrata;Manduca sexta;cotton boll weevil;Ostrinia furnacalis;Helicoverpa armigera;Leucinodes orbonalis;Trichoplusia ni;Hyphantria cunea;Pieris rapae;Chilo suppressalis;Spodoptera litura;Blattella germanica*
GCF_036215765.1	*Bacillus thuringiensis*	HD-59	Cry1Ba8,Cry1Ab9,Mpp5Ab1,Spp1Aa1,Vpa2Bb4	5	Yes	cry1B,cry1A,Mpp5A,Spp1A	Lepidoptera;Hemiptera;Diptera;Coleoptera;Blattodea	*Plutella xylostella;Ostrinia nubilalis;Spodoptera frugiperda;Maruca vitrata;Manduca sexta;cotton boll weevil;Ostrinia furnacalis;Helicoverpa armigera;Leucinodes orbonalis;Trichoplusia ni;Hyphantria cunea;Pieris rapae;Chilo*

								suppressalis;Colaphellus bowringi;Leptinotarsa decemlineata;Spodoptera litura;Blattella germanica
GCF_036215745.1	*Bacillus thuringiensis*	HD-65	Cry1Ba8,Cry1Aa3,Cry2Aa22,Vip3Aa79,Mpp5Ab1,Spp1Aa1,Vpa2Bb4	7	Yes	cry1B,cry1A,cry2A,vip3Aa,Mpp5A,Spp1A	Lepidoptera;Hemiptera;Diptera;Coleoptera;Blattodea;Aedes aegypti	*Plutella xylostella;Ostrinia nubilalis;Spodoptera frugiperda;Maruca vitrata;Manduca sexta;cotton boll weevil;Ostrinia furnacalis;Helicoverpa armigera;Leucinodes orbonalis;Trichoplusia ni;Hyphantria cunea;Pieris rapae;Chilo suppressalis;Aedes aegypti;Diaphorina citri;Helicoverpa punctigera;Helicoverpa zea;Chilo partellus;Blattella germanica;Colaphellus bowringi;Leptinotarsa decemlineata;Spodoptera litura*
GCF_036215725.1	*Bacillus thuringiensis*	HD-69	Vip3Aa80,Spp1Aa1,Vpa2Ae3,Vpb1Ac2	4	No	Vpa2Ae + Vpb1Ac,vip3Aa,Spp1A	Hemiptera;Lepidoptera;Blattodea;Aedes aegypti	*Aphis gossypii;Helicoverpa punctigera;Spodoptera frugiperda;Helicoverpa zea;Chilo partellus;Blattella germanica;Spodoptera litura*
GCF_036215705.1	*Bacillus thuringiensis*	HD-120	Cry1Ia42,Cry2Aa22,Cry2Ab41,Cry1Ba8,Vip3Aa79,Mpp5Ab1,Spp1Aa1,Vpa2Bb4,Vpb1Bb2,	10	Yes	cry1I,cry2A,cry2A,cry1B,vip3Aa,Mpp5A,Spp1A	Coleoptera;Lepidoptera;Rhabditida;Diptera;Hemiptera;Blattodea;Aedes	*Leptinotarsa decemlineata;Ostrinia furnacalis;Lobesia botrana;Plutella xylostella;Earias insulana;cotton boll weevil;Meloidogyne*

			Vpb1Bc1				aegypti	*incognita;Helicoverpa armigera;Aedes aegypti;Diaphorina citri;Ostrinia nubilalis;Spodoptera frugiperda;Maruca vitrata;Manduca sexta;Helicoverpa punctigera;Helicoverpa zea;Chilo partellus;Blattella germanica;Colaphellus bowringi;Spodoptera litura*
GCF_036215685.1	*Bacillus thuringiensis*	HD-138	Cry1Ba8,Cry1Ab9,Mpp5Ab1,Spp1Aa1,Vpa2Bb4,Vpb1Bb2,Vpb1Bc1	7	Yes	cry1B,cry1A,Mpp5A,Spp1A	Lepidoptera;Hemiptera;Diptera;Coleoptera;Blattodea	*Plutella xylostella;Ostrinia nubilalis;Spodoptera frugiperda;Maruca vitrata;Manduca sexta;cotton boll weevil;Ostrinia furnacalis;Helicoverpa armigera;Leucinodes orbonalis;Trichoplusia ni;Hyphantria cunea;Pieris rapae;Chilo suppressalis;Colaphellus bowringi;Leptinotarsa decemlineata;Spodoptera litura;Blattella germanica*
GCF_036215645.1	*Bacillus thuringiensis*	HD-140	Mpp5Ab1,Spp1Aa1,Vpa2Bb4,Vpb1Bb2	4	Yes	Mpp5A,Spp1A	Coleoptera;Lepidoptera;Blattodea	*Colaphellus bowringi;Leptinotarsa decemlineata;Spodoptera litura;Blattella germanica*
GCF_036215625.1	*Bacillus thuringiensis*	HD-148	Spp1Aa1	1	Yes	Spp1A	Lepidoptera;Blattodea	*Spodoptera litura;Blattella germanica*
GCF_036	*Bacillus*	HD-20	Cry1Ea7,Cry2Ba1,Cry1	6	No	cry1E,cry2B,cry1M,M	Lepidoptera;Hemi	*Spodoptera exigua;Spodoptera*

215605.1	*thuringiensis*	0	Ma1,Mpp15Aa1,Mpp93Aa1,Spp1Aa1			pp15A,Mpp93A,Spp1A	ptera;Rhabditida;Diptera;Pieris rapae;Cydia pomonella;Blattodea	*frugiperda;Malacosoma disstria;Bursaphelenchus xylophilus;Manduca sexta;Ostrinia nubilalis;Aedes aegypti;corn earworm (Helicoverpa zea);black cut worm (Agrotis ipsilon);cabbage looper (Trichoplusia ni);saltmarch caterpillar (Estigmene acrea);tobacco budworm (Heliothis virescens);and lettuce armyworm (Pseudaletia unipuncta).;Spodoptera litura;Blattella germanica*
GCF_036215585.1	*Bacillus thuringiensis*	HD-204	Cry1Ba8,Cry1Aa3,Cry1Ia42,Cry2Aa22,Cry2Ab41,Vip3Aa79,Spp1Aa1	7	Yes	cry1B,cry1A,cry1I,cry2A,cry2A,vip3Aa,Spp1A	Lepidoptera;Hemiptera;Diptera;Coleoptera;Rhabditida;Blattodea;Aedes aegypti	*Plutella xylostella;Ostrinia nubilalis;Spodoptera frugiperda;Maruca vitrata;Manduca sexta;cotton boll weevil;Ostrinia furnacalis;Helicoverpa armigera;Leucinodes orbonalis;Trichoplusia ni;Hyphantria cunea;Pieris rapae;Chilo suppressalis;Leptinotarsa decemlineata;Lobesia botrana;Earias insulana;Meloidogyne incognita;Aedes aegypti;Diaphorina citri;Helicoverpa punctigera;Helicoverpa zea;Chilo partellus;Blattella germanica;Spodoptera litura*

GCF_036215565.1	*Bacillus thuringiensis*	HD-201	Cry1Bb1,Cry1Ab24,Cry1Ja2,Cry1Da3,Cry1Hb2,Cry1Id2,Cry1Nb1,Cry2Ad1,Vip3Af3,App4Aa1,Spp1Aa1,Vpa2Ac2,Vpb1Ca1	13	Yes	cry1B,cry1A,cry1J,cry1D,cry1H,cry1I,cry1N,cry2A,vip3Af,App4A,Spp1A	Lepidoptera;Hemiptera;Diptera;Coleoptera;Rhabditida;Blattodea	*Plutella xylostella;Ostrinia nubilalis;Spodoptera frugiperda;Maruca vitrata;Manduca sexta;cotton boll weevil;Ostrinia furnacalis;Helicoverpa armigera;Leucinodes orbonalis;Trichoplusia ni;Hyphantria cunea;Pieris rapae;Chilo suppressalis;Lobesia botrana;Diabrotica undecimpunctata howardi;Spodoptera exigua;Bombyx mori;Spodoptera litura;Epiphyas postvittana;Leptinotarsa decemlineata;Earias insulana;Meloidogyne incognita;Colaphellus bowringi;Aedes aegypti;Diaphorina citri;Agrotis ipsilon;Laodelphax striatellus;Blattella germanica*
GCF_036215525.1	*Bacillus thuringiensis*	HD-260	Cry1Ia42,Cry2Aa22,Cry2Ab41,Cry1Ba8,Vip3Aa79,Mpp5Ab1,Spp1Aa1,Vpa2Bb4	8	Yes	cry1I,cry2A,cry2A,cry1B,vip3Aa,Mpp5A,Spp1A	Coleoptera;Lepidoptera;Rhabditida;Diptera;Hemiptera;Blattodea;Aedes aegypti	*Leptinotarsa decemlineata;Ostrinia furnacalis;Lobesia botrana;Plutella xylostella;Earias insulana;cotton boll weevil;Meloidogyne incognita;Helicoverpa armigera;Aedes aegypti;Diaphorina citri;Ostrinia nubilalis;Spodoptera frugiperda;Maruca vitrata;Manduca sexta;Helicoverpa*

								punctigera;Helicoverpa zea;Chilo partellus;Blattella germanica;Colaphellus bowringi;Spodoptera litura
GCF_036215505.1	*Bacillus thuringiensis*	HD-280	Cry1Ba8,Cry1Aa3,Cry1Ia42,Cry2Aa22,Cry2Ab41,Vip3Aa79,Mpp5Ab1,Spp1Aa1,Vpa2Bb4	9	Yes	cry1B,cry1A,cry1I,cry2A,cry2A,vip3Aa,Mpp5A,Spp1A	Lepidoptera;Hemiptera;Diptera;Coleoptera;Rhabditida;Blattodea;Aedes aegypti	*Plutella xylostella;Ostrinia nubilalis;Spodoptera frugiperda;Maruca vitrata;Manduca sexta;cotton boll weevil;Ostrinia furnacalis;Helicoverpa armigera;Leucinodes orbonalis;Trichoplusia ni;Hyphantria cunea;Pieris rapae;Chilo suppressalis;Leptinotarsa decemlineata;Lobesia botrana;Earias insulana;Meloidogyne incognita;Aedes aegypti;Diaphorina citri;Helicoverpa punctigera;Helicoverpa zea;Chilo partellus;Blattella germanica;Colaphellus bowringi;Spodoptera litura*
GCF_036215485.1	*Bacillus thuringiensis*	HD-271	Cry1Ba8,Mpp5Ab1,Spp1Aa1,Vpa2Bb4	4	Yes	cry1B,Mpp5A,Spp1A	Lepidoptera;Hemiptera;Diptera;Coleoptera;Blattodea	*Plutella xylostella;Ostrinia nubilalis;Spodoptera frugiperda;Maruca vitrata;Manduca sexta;cotton boll weevil;Colaphellus bowringi;Leptinotarsa decemlineata;Spodoptera litura;Blattella germanica*

GCF_036215465.1	*Bacillus thuringiensis*	HD-281	Cry1Ba8,Cry1Aa3,Cry1Ia42,Cry2Aa22,Cry2Ab41,Vip3Aa79,Mpp5Ab1,Spp1Aa1	8	Yes	cry1B,cry1A,cry1I,cry2A,cry2A,vip3Aa,Mpp5A,Spp1A	Lepidoptera;Hemiptera;Diptera;Coleoptera;Rhabditida;Blattodea;Aedes aegypti	*Plutella xylostella;Ostrinia nubilalis;Spodoptera frugiperda;Maruca vitrata;Manduca sexta;cotton boll weevil;Ostrinia furnacalis;Helicoverpa armigera;Leucinodes orbonalis;Trichoplusia ni;Hyphantria cunea;Pieris rapae;Chilo suppressalis;Leptinotarsa decemlineata;Lobesia botrana;Earias insulana;Meloidogyne incognita;Aedes aegypti;Diaphorina citri;Helicoverpa punctigera;Helicoverpa zea;Chilo partellus;Blattella germanica;Colaphellus bowringi;Spodoptera litura*
GCF_036215425.1	*Bacillus thuringiensis*	HD-289	Cry1Ba8,Cry1Ia42,Cry2Aa22,Cry2Ab41,Vip3Aa79,Mpp5Ab1,Spp1Aa1,Vpa2Bb4,Vpb1Bc1,Vpb1Bb2	10	Yes	cry1B,cry1I,cry2A,cry2A,vip3Aa,Mpp5A,Spp1A	Lepidoptera;Hemiptera;Diptera;Coleoptera;Rhabditida;Blattodea;Aedes aegypti	*Plutella xylostella;Ostrinia nubilalis;Spodoptera frugiperda;Maruca vitrata;Manduca sexta;cotton boll weevil;Leptinotarsa decemlineata;Ostrinia furnacalis;Lobesia botrana;Earias insulana;Meloidogyne incognita;Helicoverpa armigera;Aedes aegypti;Diaphorina citri;Helicoverpa punctigera;Helicoverpa zea;Chilo*

								partellus;Blattella germanica;Colaphellus bowringi;Spodoptera litura
GCF_036215385.1	*Bacillus thuringiensis*	HD-298	Cry1Ba8,Cry1Ia42,Cry1Aa17,Vip3Aa79,Mpp5Ab1,Spp1Aa1	6	Yes	cry1B,cry1I,cry1A,vip3Aa,Mpp5A,Spp1A	Lepidoptera;Hemiptera;Diptera;Coleoptera;Rhabditida;Blattodea;Aedes aegypti	*Plutella xylostella;Ostrinia nubilalis;Spodoptera frugiperda;Maruca vitrata;Manduca sexta;cotton boll weevil;Leptinotarsa decemlineata;Ostrinia furnacalis;Lobesia botrana;Earias insulana;Meloidogyne incognita;Helicoverpa armigera;Leucinodes orbonalis;Trichoplusia ni;Hyphantria cunea;Pieris rapae;Chilo suppressalis;Helicoverpa punctigera;Helicoverpa zea;Chilo partellus;Blattella germanica;Colaphellus bowringi;Spodoptera litura*
GCF_036215325.1	*Bacillus thuringiensis*	HD-327	Cry1Ba8,Cry1Aa3,Cry1Ia42,Vip3Aa79,Mpp5Ab1,Spp1Aa1,Vpa2Bb4	7	Yes	cry1B,cry1A,cry1I,vip3Aa,Mpp5A,Spp1A	Lepidoptera;Hemiptera;Diptera;Coleoptera;Rhabditida;Blattodea;Aedes aegypti	*Plutella xylostella;Ostrinia nubilalis;Spodoptera frugiperda;Maruca vitrata;Manduca sexta;cotton boll weevil;Ostrinia furnacalis;Helicoverpa armigera;Leucinodes orbonalis;Trichoplusia ni;Hyphantria cunea;Pieris rapae;Chilo suppressalis;Leptinotarsa*

								decemlineata;Lobesia botrana;Earias insulana;Meloidogyne incognita;Helicoverpa punctigera;Helicoverpa zea;Chilo partellus;Blattella germanica;Colaphellus bowringi;Spodoptera litura
GCF_036215305.1	*Bacillus thuringiensis*	HD-368	Cry1Aa9,Spp1Aa1	2	Yes	cry1A,Spp1A	Lepidoptera;Blattodea	*Plutella xylostella;Ostrinia furnacalis;Helicoverpa armigera;Leucinodes orbonalis;Trichoplusia ni;Hyphantria cunea;Spodoptera frugiperda;Pieris rapae;Chilo suppressalis;Spodoptera litura;Blattella germanica*
GCF_036215285.1	*Bacillus thuringiensis*	HD-369	Cry1Aa9,Spp1Aa1	2	Yes	cry1A,Spp1A	Lepidoptera;Blattodea	*Plutella xylostella;Ostrinia furnacalis;Helicoverpa armigera;Leucinodes orbonalis;Trichoplusia ni;Hyphantria cunea;Spodoptera frugiperda;Pieris rapae;Chilo suppressalis;Spodoptera litura;Blattella germanica*
GCF_036215265.1	*Bacillus thuringiensis*	HD-371	Cry1Aa9,Spp1Aa1	2	Yes	cry1A,Spp1A	Lepidoptera;Blattodea	*Plutella xylostella;Ostrinia furnacalis;Helicoverpa armigera;Leucinodes orbonalis;Trichoplusia ni;Hyphantria cunea;Spodoptera frugiperda;Pieris rapae;Chilo suppressalis;Spodoptera litura;Blattella germanica*

GCF_036215245.1	*Bacillus thuringiensis*	HD-372	Cry1Aa9,Spp1Aa1	2	Yes	cry1A,Spp1A	Lepidoptera;Blattodea	*Plutella xylostella;Ostrinia furnacalis;Helicoverpa armigera;Leucinodes orbonalis;Trichoplusia ni;Hyphantria cunea;Spodoptera frugiperda;Pieris rapae;Chilo suppressalis;Spodoptera litura;Blattella germanica*
GCF_036215225.1	*Bacillus thuringiensis*	HD-373	Cry1Aa9,Spp1Aa1	2	Yes	cry1A,Spp1A	Lepidoptera;Blattodea	*Plutella xylostella;Ostrinia furnacalis;Helicoverpa armigera;Leucinodes orbonalis;Trichoplusia ni;Hyphantria cunea;Spodoptera frugiperda;Pieris rapae;Chilo suppressalis;Spodoptera litura;Blattella germanica*
GCF_036215205.1	*Bacillus thuringiensis*	HD-379	Cry1Aa9,Spp1Aa1	2	Yes	cry1A,Spp1A	Lepidoptera;Blattodea	*Plutella xylostella;Ostrinia furnacalis;Helicoverpa armigera;Leucinodes orbonalis;Trichoplusia ni;Hyphantria cunea;Spodoptera frugiperda;Pieris rapae;Chilo suppressalis;Spodoptera litura;Blattella germanica*
GCF_036215185.1	*Bacillus thuringiensis*	HD-377	Cry1Aa9,Spp1Aa1	2	Yes	cry1A,Spp1A	Lepidoptera;Blattodea	*Plutella xylostella;Ostrinia furnacalis;Helicoverpa armigera;Leucinodes orbonalis;Trichoplusia ni;Hyphantria cunea;Spodoptera frugiperda;Pieris rapae;Chilo suppressalis;Spodoptera litura;Blattella germanica*

GCF_036215165.1	*Bacillus thuringiensis*	HD-381	Cry1Aa9,Spp1Aa1	2	Yes	cry1A,Spp1A	Lepidoptera;Blattodea	*Plutella xylostella;Ostrinia furnacalis;Helicoverpa armigera;Leucinodes orbonalis;Trichoplusia ni;Hyphantria cunea;Spodoptera frugiperda;Pieris rapae;Chilo suppressalis;Spodoptera litura;Blattella germanica*
GCF_036215145.1	*Bacillus thuringiensis*	HD-384	Cry1Aa9,Spp1Aa1	2	Yes	cry1A,Spp1A	Lepidoptera;Blattodea	*Plutella xylostella;Ostrinia furnacalis;Helicoverpa armigera;Leucinodes orbonalis;Trichoplusia ni;Hyphantria cunea;Spodoptera frugiperda;Pieris rapae;Chilo suppressalis;Spodoptera litura;Blattella germanica*
GCF_036215125.1	*Bacillus thuringiensis*	HD-382	Cry1Aa9,Spp1Aa1	2	Yes	cry1A,Spp1A	Lepidoptera;Blattodea	*Plutella xylostella;Ostrinia furnacalis;Helicoverpa armigera;Leucinodes orbonalis;Trichoplusia ni;Hyphantria cunea;Spodoptera frugiperda;Pieris rapae;Chilo suppressalis;Spodoptera litura;Blattella germanica*
GCF_036215105.1	*Bacillus thuringiensis*	HD-389	Cry1Aa9,Spp1Aa1	2	Yes	cry1A,Spp1A	Lepidoptera;Blattodea	*Plutella xylostella;Ostrinia furnacalis;Helicoverpa armigera;Leucinodes orbonalis;Trichoplusia ni;Hyphantria cunea;Spodoptera frugiperda;Pieris rapae;Chilo suppressalis;Spodoptera litura;Blattella germanica*

GCF_036215085.1	*Bacillus thuringiensis*	HD-386	Cry1Aa9,Spp1Aa1	2	Yes	cry1A,Spp1A	Lepidoptera;Blattodea	*Plutella xylostella;Ostrinia furnacalis;Helicoverpa armigera;Leucinodes orbonalis;Trichoplusia ni;Hyphantria cunea;Spodoptera frugiperda;Pieris rapae;Chilo suppressalis;Spodoptera litura;Blattella germanica*
GCF_036215065.1	*Bacillus thuringiensis*	HD-391	Cry1Aa9,Spp1Aa1	2	Yes	cry1A,Spp1A	Lepidoptera;Blattodea	*Plutella xylostella;Ostrinia furnacalis;Helicoverpa armigera;Leucinodes orbonalis;Trichoplusia ni;Hyphantria cunea;Spodoptera frugiperda;Pieris rapae;Chilo suppressalis;Spodoptera litura;Blattella germanica*
GCF_036215045.1	*Bacillus thuringiensis*	HD-394	Cry1Aa9,Spp1Aa1	2	Yes	cry1A,Spp1A	Lepidoptera;Blattodea	*Plutella xylostella;Ostrinia furnacalis;Helicoverpa armigera;Leucinodes orbonalis;Trichoplusia ni;Hyphantria cunea;Spodoptera frugiperda;Pieris rapae;Chilo suppressalis;Spodoptera litura;Blattella germanica*
GCF_036215025.1	*Bacillus thuringiensis*	HD-392	Cry1Aa9,Spp1Aa1	2	Yes	cry1A,Spp1A	Lepidoptera;Blattodea	*Plutella xylostella;Ostrinia furnacalis;Helicoverpa armigera;Leucinodes orbonalis;Trichoplusia ni;Hyphantria cunea;Spodoptera frugiperda;Pieris rapae;Chilo suppressalis;Spodoptera litura;Blattella germanica*

GCF_036215005.1	*Bacillus thuringiensis*	HD-402	Cry1Aa9,Spp1Aa1	2	Yes	cry1A,Spp1A	Lepidoptera;Blattodea	*Plutella xylostella;Ostrinia furnacalis;Helicoverpa armigera;Leucinodes orbonalis;Trichoplusia ni;Hyphantria cunea;Spodoptera frugiperda;Pieris rapae;Chilo suppressalis;Spodoptera litura;Blattella germanica*
GCF_036214985.1	*Bacillus thuringiensis*	HD-397	Cry1Aa9,Spp1Aa1	2	Yes	cry1A,Spp1A	Lepidoptera;Blattodea	*Plutella xylostella;Ostrinia furnacalis;Helicoverpa armigera;Leucinodes orbonalis;Trichoplusia ni;Hyphantria cunea;Spodoptera frugiperda;Pieris rapae;Chilo suppressalis;Spodoptera litura;Blattella germanica*
GCF_036214965.1	*Bacillus thuringiensis*	HD-403	Cry1Aa9,Spp1Aa1	2	Yes	cry1A,Spp1A	Lepidoptera;Blattodea	*Plutella xylostella;Ostrinia furnacalis;Helicoverpa armigera;Leucinodes orbonalis;Trichoplusia ni;Hyphantria cunea;Spodoptera frugiperda;Pieris rapae;Chilo suppressalis;Spodoptera litura;Blattella germanica*
GCF_036214945.1	*Bacillus thuringiensis*	HD-405	Cry1Aa9,Spp1Aa1	2	Yes	cry1A,Spp1A	Lepidoptera;Blattodea	*Plutella xylostella;Ostrinia furnacalis;Helicoverpa armigera;Leucinodes orbonalis;Trichoplusia ni;Hyphantria cunea;Spodoptera frugiperda;Pieris rapae;Chilo suppressalis;Spodoptera litura;Blattella germanica*

GCF_036214825.1	*Bacillus thuringiensis*	HD-380	Cry1Aa9,Spp1Aa1	2	Yes	cry1A,Spp1A	Lepidoptera;Blattodea	*Plutella xylostella;Ostrinia furnacalis;Helicoverpa armigera;Leucinodes orbonalis;Trichoplusia ni;Hyphantria cunea;Spodoptera frugiperda;Pieris rapae;Chilo suppressalis;Spodoptera litura;Blattella germanica*
GCF_036214805.1	*Bacillus thuringiensis*	HD-385	Cry1Aa9,Spp1Aa1	2	Yes	cry1A,Spp1A	Lepidoptera;Blattodea	*Plutella xylostella;Ostrinia furnacalis;Helicoverpa armigera;Leucinodes orbonalis;Trichoplusia ni;Hyphantria cunea;Spodoptera frugiperda;Pieris rapae;Chilo suppressalis;Spodoptera litura;Blattella germanica*
GCF_036214785.1	*Bacillus thuringiensis*	HD-387	Cry1Aa9,Spp1Aa1	2	Yes	cry1A,Spp1A	Lepidoptera;Blattodea	*Plutella xylostella;Ostrinia furnacalis;Helicoverpa armigera;Leucinodes orbonalis;Trichoplusia ni;Hyphantria cunea;Spodoptera frugiperda;Pieris rapae;Chilo suppressalis;Spodoptera litura;Blattella germanica*
GCF_036214765.1	*Bacillus thuringiensis*	HD-383	Cry1Aa9,Spp1Aa1	2	Yes	cry1A,Spp1A	Lepidoptera;Blattodea	*Plutella xylostella;Ostrinia furnacalis;Helicoverpa armigera;Leucinodes orbonalis;Trichoplusia ni;Hyphantria cunea;Spodoptera frugiperda;Pieris rapae;Chilo suppressalis;Spodoptera litura;Blattella germanica*

GCF_036214745.1	*Bacillus thuringiensis*	HD-396	Cry1Aa9,Spp1Aa1	2	Yes	cry1A,Spp1A	Lepidoptera;Blattodea	*Plutella xylostella;Ostrinia furnacalis;Helicoverpa armigera;Leucinodes orbonalis;Trichoplusia ni;Hyphantria cunea;Spodoptera frugiperda;Pieris rapae;Chilo suppressalis;Spodoptera litura;Blattella germanica*
GCF_036214725.1	*Bacillus thuringiensis*	HD-398	Cry1Aa9,Spp1Aa1	2	Yes	cry1A,Spp1A	Lepidoptera;Blattodea	*Plutella xylostella;Ostrinia furnacalis;Helicoverpa armigera;Leucinodes orbonalis;Trichoplusia ni;Hyphantria cunea;Spodoptera frugiperda;Pieris rapae;Chilo suppressalis;Spodoptera litura;Blattella germanica*
GCF_036214705.1	*Bacillus thuringiensis*	HD-409	Cry1Aa9,Spp1Aa1	2	Yes	cry1A,Spp1A	Lepidoptera;Blattodea	*Plutella xylostella;Ostrinia furnacalis;Helicoverpa armigera;Leucinodes orbonalis;Trichoplusia ni;Hyphantria cunea;Spodoptera frugiperda;Pieris rapae;Chilo suppressalis;Spodoptera litura;Blattella germanica*
GCF_036214685.1	*Bacillus thuringiensis*	HD-399	Cry1Aa9,Spp1Aa1	2	Yes	cry1A,Spp1A	Lepidoptera;Blattodea	*Plutella xylostella;Ostrinia furnacalis;Helicoverpa armigera;Leucinodes orbonalis;Trichoplusia ni;Hyphantria cunea;Spodoptera frugiperda;Pieris rapae;Chilo suppressalis;Spodoptera litura;Blattella germanica*

GCF_036214665.1	*Bacillus thuringiensis*	HD-410	Cry1Aa9,Spp1Aa1	2	Yes	cry1A,Spp1A	Lepidoptera;Blattodea	*Plutella xylostella;Ostrinia furnacalis;Helicoverpa armigera;Leucinodes orbonalis;Trichoplusia ni;Hyphantria cunea;Spodoptera frugiperda;Pieris rapae;Chilo suppressalis;Spodoptera litura;Blattella germanica*
GCF_036214645.1	*Bacillus thuringiensis*	HD-412	Cry1Aa9,Spp1Aa1	2	Yes	cry1A,Spp1A	Lepidoptera;Blattodea	*Plutella xylostella;Ostrinia furnacalis;Helicoverpa armigera;Leucinodes orbonalis;Trichoplusia ni;Hyphantria cunea;Spodoptera frugiperda;Pieris rapae;Chilo suppressalis;Spodoptera litura;Blattella germanica*
GCF_036214625.1	*Bacillus thuringiensis*	HD-411	Cry1Aa9,Spp1Aa1	2	Yes	cry1A,Spp1A	Lepidoptera;Blattodea	*Plutella xylostella;Ostrinia furnacalis;Helicoverpa armigera;Leucinodes orbonalis;Trichoplusia ni;Hyphantria cunea;Spodoptera frugiperda;Pieris rapae;Chilo suppressalis;Spodoptera litura;Blattella germanica*
GCF_036214605.1	*Bacillus thuringiensis*	HD-413	Cry1Aa9,Spp1Aa1	2	Yes	cry1A,Spp1A	Lepidoptera;Blattodea	*Plutella xylostella;Ostrinia furnacalis;Helicoverpa armigera;Leucinodes orbonalis;Trichoplusia ni;Hyphantria cunea;Spodoptera frugiperda;Pieris rapae;Chilo suppressalis;Spodoptera litura;Blattella germanica*

GCF_036214585.1	*Bacillus thuringiensis*	HD-414	Cry1Aa9,Spp1Aa1	2	Yes	cry1A,Spp1A	Lepidoptera;Blattodea	*Plutella xylostella;Ostrinia furnacalis;Helicoverpa armigera;Leucinodes orbonalis;Trichoplusia ni;Hyphantria cunea;Spodoptera frugiperda;Pieris rapae;Chilo suppressalis;Spodoptera litura;Blattella germanica*
GCF_036214565.1	*Bacillus thuringiensis*	HD-423	Cry1Aa9,Spp1Aa1	2	Yes	cry1A,Spp1A	Lepidoptera;Blattodea	*Plutella xylostella;Ostrinia furnacalis;Helicoverpa armigera;Leucinodes orbonalis;Trichoplusia ni;Hyphantria cunea;Spodoptera frugiperda;Pieris rapae;Chilo suppressalis;Spodoptera litura;Blattella germanica*
GCF_036214545.1	*Bacillus thuringiensis*	HD-415	Cry1Aa9,Spp1Aa1	2	Yes	cry1A,Spp1A	Lepidoptera;Blattodea	*Plutella xylostella;Ostrinia furnacalis;Helicoverpa armigera;Leucinodes orbonalis;Trichoplusia ni;Hyphantria cunea;Spodoptera frugiperda;Pieris rapae;Chilo suppressalis;Spodoptera litura;Blattella germanica*
GCF_036214525.1	*Bacillus thuringiensis*	HD-420	Cry1Aa9,Spp1Aa1	2	Yes	cry1A,Spp1A	Lepidoptera;Blattodea	*Plutella xylostella;Ostrinia furnacalis;Helicoverpa armigera;Leucinodes orbonalis;Trichoplusia ni;Hyphantria cunea;Spodoptera frugiperda;Pieris rapae;Chilo suppressalis;Spodoptera litura;Blattella germanica*

GCF_036214505.1	*Bacillus thuringiensis*	HD-433	Cry1Aa9,Spp1Aa1	2	Yes	cry1A,Spp1A	Lepidoptera;Blattodea	*Plutella xylostella;Ostrinia furnacalis;Helicoverpa armigera;Leucinodes orbonalis;Trichoplusia ni;Hyphantria cunea;Spodoptera frugiperda;Pieris rapae;Chilo suppressalis;Spodoptera litura;Blattella germanica*
GCF_036214485.1	*Bacillus thuringiensis*	HD-429	Cry1Aa9,Spp1Aa1	2	Yes	cry1A,Spp1A	Lepidoptera;Blattodea	*Plutella xylostella;Ostrinia furnacalis;Helicoverpa armigera;Leucinodes orbonalis;Trichoplusia ni;Hyphantria cunea;Spodoptera frugiperda;Pieris rapae;Chilo suppressalis;Spodoptera litura;Blattella germanica*
GCF_036214465.1	*Bacillus thuringiensis*	HD-434	Cry1Aa9,Spp1Aa1	2	Yes	cry1A,Spp1A	Lepidoptera;Blattodea	*Plutella xylostella;Ostrinia furnacalis;Helicoverpa armigera;Leucinodes orbonalis;Trichoplusia ni;Hyphantria cunea;Spodoptera frugiperda;Pieris rapae;Chilo suppressalis;Spodoptera litura;Blattella germanica*
GCF_036214425.1	*Bacillus thuringiensis*	HD-447	Cry2Ab41,Vip3Aa80,Spp1Aa1,Vpa2Ae3,Vpb1Ac2	5	No	Vpa2Ae + Vpb1Ac,cry2A,vip3Aa,Spp1A	Hemiptera;Lepidoptera;Diptera;Blattodea;Aedes aegypti	*Aphis gossypii;Helicoverpa armigera;Aedes aegypti;Diaphorina citri;Helicoverpa punctigera;Spodoptera frugiperda;Helicoverpa zea;Chilo partellus;Blattella germanica;Spodoptera litura*

GCF_036214145.1	*Bacillus thuringiensis*	HD-513	Cry1Ba8,Cry1Aa3,Cry1Ia42,Cry2Aa22,Cry2Ab41,Vip3Aa79,Mpp5Ab1,Spp1Aa1,Vpa2Bb4,Vpb1Bb2,Vpb1Bc1	11	Yes	cry1B,cry1A,cry1I,cry2A,cry2A,vip3Aa,Mpp5A,Spp1A	Lepidoptera;Hemiptera;Diptera;Coleoptera;Rhabditida;Blattodea;Aedes aegypti	*Plutella xylostella;Ostrinia nubilalis;Spodoptera frugiperda;Maruca vitrata;Manduca sexta;cotton boll weevil;Ostrinia furnacalis;Helicoverpa armigera;Leucinodes orbonalis;Trichoplusia ni;Hyphantria cunea;Pieris rapae;Chilo suppressalis;Leptinotarsa decemlineata;Lobesia botrana;Earias insulana;Meloidogyne incognita;Aedes aegypti;Diaphorina citri;Helicoverpa punctigera;Helicoverpa zea;Chilo partellus;Blattella germanica;Colaphellus bowringi;Spodoptera litura*
GCF_036214125.1	*Bacillus thuringiensis*	HD-515	Cry1Ba8,Cry1Aa3,Cry1Ia42,Cry2Aa22,Cry2Ab41,Vip3Aa79,Mpp5Ab1,Spp1Aa1,Vpa2Bb4,Vpb1Bb2,Vpb1Bc1	11	Yes	cry1B,cry1A,cry1I,cry2A,cry2A,vip3Aa,Mpp5A,Spp1A	Lepidoptera;Hemiptera;Diptera;Coleoptera;Rhabditida;Blattodea;Aedes aegypti	*Plutella xylostella;Ostrinia nubilalis;Spodoptera frugiperda;Maruca vitrata;Manduca sexta;cotton boll weevil;Ostrinia furnacalis;Helicoverpa armigera;Leucinodes orbonalis;Trichoplusia ni;Hyphantria cunea;Pieris rapae;Chilo suppressalis;Leptinotarsa decemlineata;Lobesia botrana;Earias insulana;Meloidogyne*

								incognita;Aedes aegypti;Diaphorina citri;Helicoverpa punctigera;Helicoverpa zea;Chilo partellus;Blattella germanica;Colaphellus bowringi;Spodoptera litura
GCF_036214105.1	*Bacillus thuringiensis*	HD-514	Cry1Ba8,Cry1Aa3,Mpp5Ab1,Spp1Aa1,Vpa2Bb4	5	Yes	cry1B,cry1A,Mpp5A,Spp1A	Lepidoptera;Hemiptera;Diptera;Coleoptera;Blattodea	*Plutella xylostella;Ostrinia nubilalis;Spodoptera frugiperda;Maruca vitrata;Manduca sexta;cotton boll weevil;Ostrinia furnacalis;Helicoverpa armigera;Leucinodes orbonalis;Trichoplusia ni;Hyphantria cunea;Pieris rapae;Chilo suppressalis;Colaphellus bowringi;Leptinotarsa decemlineata;Spodoptera litura;Blattella germanica*
GCF_036214085.1	*Bacillus thuringiensis*	HD-517	Cry1Ba8,Cry1Aa3,Cry1Ia42,Cry2Aa22,Cry2Ab41,Vip3Aa79,Mpp5Ab1,Spp1Aa1,Vpa2Bb4,Vpb1Bb2,Vpb1Bc1	11	Yes	cry1B,cry1A,cry1I,cry2A,cry2A,vip3Aa,Mpp5A,Spp1A	Lepidoptera;Hemiptera;Diptera;Coleoptera;Rhabditida;Blattodea;Aedes aegypti	*Plutella xylostella;Ostrinia nubilalis;Spodoptera frugiperda;Maruca vitrata;Manduca sexta;cotton boll weevil;Ostrinia furnacalis;Helicoverpa armigera;Leucinodes orbonalis;Trichoplusia ni;Hyphantria cunea;Pieris rapae;Chilo suppressalis;Leptinotarsa decemlineata;Lobesia botrana;Earias*

								insulana;Meloidogyne incognita;Aedes aegypti;Diaphorina citri;Helicoverpa punctigera;Helicoverpa zea;Chilo partellus;Blattella germanica;Colaphellus bowringi;Spodoptera litura
GCF_036214065.1	*Bacillus thuringiensis*	HD-522	Cry10Aa4,Mpp60Ba3,Mpp60Aa3	3	No	cry10A,Mpp60B,Mpp60A	Diptera;Coleoptera	*Aedes aegypti;cotton boll weevil;Culex quinquefasciatus*
GCF_036214045.1	*Bacillus thuringiensis*	HD-530	Cry1Ab24,Cry1Ja2,Cry1Da3,Cry1Hb2,Cry1Id2,Cry1Nb1,Cry2Ad1,Cry1Ka1,Cry1Ib5,Cry1Fb4,Cry1Bb1,Vip3Af3,Vip3Ag6,Spp1Aa1,Vpa2Ac2,Vpb1Ca1	16	No	cry1A,cry1J,cry1D,cry1H,cry1I,cry1N,cry2A,cry1K,cry1I,cry1F,cry1B,vip3Af,vip3Ag,Spp1A	Lepidoptera;Coleoptera;Rhabditida;Diptera;Hemiptera;Blattodea	*Plutella xylostella;Ostrinia furnacalis;Helicoverpa armigera;Leucinodes orbonalis;Trichoplusia ni;Hyphantria cunea;Spodoptera frugiperda;Pieris rapae;Chilo suppressalis;Manduca sexta;Lobesia botrana;Diabrotica undecimpunctata howardi;Ostrinia nubilalis;Spodoptera exigua;Bombyx mori;Spodoptera litura;Epiphyas postvittana;Leptinotarsa decemlineata;Earias insulana;cotton boll weevil;Meloidogyne incognita;Colaphellus bowringi;Aedes aegypti;Diaphorina citri;Artogeia rapae;Agrotis ipsilon;Diabrotica virgifera virgifera;Maruca vitrata;Spodoptera*

								littoralis;Chrysodeixis chalcites;Blattella germanica
GCF_036214025.1	*Bacillus thuringiensis*	HD-533	Cry1Da3,Cry1Hb2,Cry1Id2,Cry1Nb1,Cry1Ka1,Cry1Ib5,Cry1Ab24,Cry2Ad1,Cry1Bb1,Vip3Af3,Spp1Aa1,Vpa2Ac2,Vpb1Ca1	13	No	cry1D,cry1H,cry1I,cry1N,cry1K,cry1I,cry1A,cry2A,cry1B,vip3Af,Spp1A	Lepidoptera;Coleoptera;Rhabditida;Diptera;Hemiptera;Blattodea	*Ostrinia nubilalis;Plutella xylostella;Spodoptera exigua;Spodoptera frugiperda;Bombyx mori;Spodoptera litura;Epiphyas postvittana;Ostrinia furnacalis;Leptinotarsa decemlineata;Lobesia botrana;Earias insulana;cotton boll weevil;Meloidogyne incognita;Colaphellus bowringi;Artogeia rapae;Pieris rapae;Helicoverpa armigera;Leucinodes orbonalis;Trichoplusia ni;Hyphantria cunea;Chilo suppressalis;Aedes aegypti;Diaphorina citri;Maruca vitrata;Manduca sexta;Agrotis ipsilon;Blattella germanica*
GCF_036213985.1	*Bacillus thuringiensis*	HD-547	Cry1Ab37,Cry1Ia42,Cry1Bd2,Vip3Ah2	4	No	cry1A,cry1I,cry1B,vip3Ah	Lepidoptera;Coleoptera;Rhabditida;Hemiptera;Diptera	*Plutella xylostella;Ostrinia furnacalis;Helicoverpa armigera;Leucinodes orbonalis;Trichoplusia ni;Hyphantria cunea;Spodoptera frugiperda;Pieris rapae;Chilo suppressalis;Leptinotarsa decemlineata;Lobesia botrana;Earias*

								insulana;cotton boll weevil;Meloidogyne incognita;Ostrinia nubilalis;Maruca vitrata;Manduca sexta;Agrotis ipsilon
GCF_036213945.1	*Bacillus thuringiensis*	HD-561	Cry1Ba8,Cry1Ab9,Mpp5Ab1,Spp1Aa1,Vpa2Bb4,Vpb1Bb2	6	Yes	cry1B,cry1A,Mpp5A,Spp1A	Lepidoptera;Hemiptera;Diptera;Coleoptera;Blattodea	*Plutella xylostella;Ostrinia nubilalis;Spodoptera frugiperda;Maruca vitrata;Manduca sexta;cotton boll weevil;Ostrinia furnacalis;Helicoverpa armigera;Leucinodes orbonalis;Trichoplusia ni;Hyphantria cunea;Pieris rapae;Chilo suppressalis;Colaphellus bowringi;Leptinotarsa decemlineata;Spodoptera litura;Blattella germanica*
GCF_036213925.1	*Bacillus thuringiensis*	HD-563	Cry11Aa3,Cry10Aa4,Cyt1Ca1,Cyt1Aa11,Cyt2Ba17,Mpp60Ba3,Mpp60Aa3	7	No	cry11A,cry10A,cyt1A,cyt2B,Mpp60B,Mpp60A	Diptera;Coleoptera;Hemiptera;Lepidoptera	*Aedes aegypti;Anopheles albimanus;Culex quinquefasciatu;Culex pipiens;cotton boll weevil;Culex quinquefasciatus;Culex pipien;Diaphorina citri;Plutella xylostella;Anopheles stephensi;Anopheles gambiae;Bradysia difformis*
GCF_036213885.1	*Bacillus thuringi*	HD-604	Cry1Ea7,Spp1Aa1	2	No	cry1E,Spp1A	Lepidoptera;Hemiptera;Rhabditida;B	*Spodoptera exigua;Spodoptera frugiperda;Malacosoma*

	ensis						lattodea	*disstria;Bursaphelenchus xylophilus;Spodoptera litura;Blattella germanica*
GCF_036213865.1	*Bacillus thuringiensis*	HD-597	Cry1Ka1,Cry1Ib5,Vip3Ag6,Spp1Aa1,Vpa2Ac2,Vpb1Ca1	6	No	cry1K,cry1I,vip3Ag,Spp1A	Lepidoptera;Coleoptera;Rhabditida;Blattodea	*Artogeia rapae;Pieris rapae;Leptinotarsa decemlineata;Ostrinia furnacalis;Lobesia botrana;Plutella xylostella;Earias insulana;cotton boll weevil;Meloidogyne incognita;Spodoptera littoralis;Chrysodeixis chalcites;Spodoptera exigua;Spodoptera litura;Blattella germanica*
GCF_036213825.1	*Bacillus thuringiensis*	HD-609	Cry1Ea7,Spp1Aa1	2	No	cry1E,Spp1A	Lepidoptera;Hemiptera;Rhabditida;Blattodea	*Spodoptera exigua;Spodoptera frugiperda;Malacosoma disstria;Bursaphelenchus xylophilus;Spodoptera litura;Blattella germanica*
GCF_036213545.1	*Bacillus thuringiensis*	NRS-996	Cry1Ba8,Cry1Ab9,Mpp5Ab1,Spp1Aa1,Vpa2Bb4,Vpb1Bc1,Vpb1Bb2	7	Yes	cry1B,cry1A,Mpp5A,Spp1A	Lepidoptera;Hemiptera;Diptera;Coleoptera;Blattodea	*Plutella xylostella;Ostrinia nubilalis;Spodoptera frugiperda;Maruca vitrata;Manduca sexta;cotton boll weevil;Ostrinia furnacalis;Helicoverpa armigera;Leucinodes orbonalis;Trichoplusia ni;Hyphantria cunea;Pieris rapae;Chilo suppressalis;Colaphellus*

								bowringi;Leptinotarsa decemlineata;Spodoptera litura;Blattella germanica
GCF_036212925.1	*Bacillus thuringiensis*	NRS-1386	Cry1Ba8,Cry1Ab9,Mpp5Ab1,Spp1Aa1,Vpa2Bb4,Vpb1Bc1	6	Yes	cry1B,cry1A,Mpp5A,Spp1A	Lepidoptera;Hemiptera;Diptera;Coleoptera;Blattodea	*Plutella xylostella;Ostrinia nubilalis;Spodoptera frugiperda;Maruca vitrata;Manduca sexta;cotton boll weevil;Ostrinia furnacalis;Helicoverpa armigera;Leucinodes orbonalis;Trichoplusia ni;Hyphantria cunea;Pieris rapae;Chilo suppressalis;Colaphellus bowringi;Leptinotarsa decemlineata;Spodoptera litura;Blattella germanica*
GCF_036212885.1	*Bacillus thuringiensis*	NRS-1387	Cry1Ba8,Cry1Ab9,Mpp5Ab1,Spp1Aa1,Vpa2Bb4,Vpb1Bb2,Vpb1Bc1	7	Yes	cry1B,cry1A,Mpp5A,Spp1A	Lepidoptera;Hemiptera;Diptera;Coleoptera;Blattodea	*Plutella xylostella;Ostrinia nubilalis;Spodoptera frugiperda;Maruca vitrata;Manduca sexta;cotton boll weevil;Ostrinia furnacalis;Helicoverpa armigera;Leucinodes orbonalis;Trichoplusia ni;Hyphantria cunea;Pieris rapae;Chilo suppressalis;Colaphellus bowringi;Leptinotarsa decemlineata;Spodoptera litura;Blattella germanica*
GCF_036	*Bacillus*	NRS-1	Mpp5Ab1,Spp1Aa1,Vp	5	Yes	Mpp5A,Spp1A	Coleoptera;Lepido	*Colaphellus bowringi;Leptinotarsa*

212865.1	*thuringiensis*	440	a2Bb4,Vpb1Bb2,Vpb1Bc1				ptera;Blattodea	*decemlineata;Spodoptera litura;Blattella germanica*
GCF_036212835.1	*Bacillus thuringiensis*	NRS-1443	Spp1Aa1	1	Yes	Spp1A	Lepidoptera;Blattodea	*Spodoptera litura;Blattella germanica*
GCF_036212785.1	*Bacillus thuringiensis*	NRS-1458	Cry1Ba8,Cry1Ia42,Cry2Aa22,Cry2Ab41,Vip3Aa79,Mpp5Ab1,Spp1Aa1,Vpa2Bb4,Vpb1Bb2,Vpb1Bc1	10	Yes	cry1B,cry1I,cry2A,cry2A,vip3Aa,Mpp5A,Spp1A	Lepidoptera;Hemiptera;Diptera;Coleoptera;Rhabditida;Blattodea;Aedes aegypti	*Plutella xylostella;Ostrinia nubilalis;Spodoptera frugiperda;Maruca vitrata;Manduca sexta;cotton boll weevil;Leptinotarsa decemlineata;Ostrinia furnacalis;Lobesia botrana;Earias insulana;Meloidogyne incognita;Helicoverpa armigera;Aedes aegypti;Diaphorina citri;Helicoverpa punctigera;Helicoverpa zea;Chilo partellus;Blattella germanica;Colaphellus bowringi;Spodoptera litura*
GCF_036212765.1	*Bacillus thuringiensis*	NRS-1460	Cry1Ba8,Cry1Ia42,Cry2Aa22,Cry2Ab41,Vip3Aa79,Mpp5Ab1,Spp1Aa1,Vpa2Bb4,Vpb1Bb2,Vpb1Bc1	10	Yes	cry1B,cry1I,cry2A,cry2A,vip3Aa,Mpp5A,Spp1A	Lepidoptera;Hemiptera;Diptera;Coleoptera;Rhabditida;Blattodea;Aedes aegypti	*Plutella xylostella;Ostrinia nubilalis;Spodoptera frugiperda;Maruca vitrata;Manduca sexta;cotton boll weevil;Leptinotarsa decemlineata;Ostrinia furnacalis;Lobesia botrana;Earias insulana;Meloidogyne incognita;Helicoverpa armigera;Aedes aegypti;Diaphorina*

								citri;Helicoverpa punctigera;Helicoverpa zea;Chilo partellus;Blattella germanica;Colaphellus bowringi;Spodoptera litura
GCF_036212745.1	*Bacillus thuringiensis*	NRS-1477	Spp1Aa1	1	Yes	Spp1A	Lepidoptera;Blattodea	*Spodoptera litura;Blattella germanica*
GCF_036212725.1	*Bacillus thuringiensis*	NRS-1478	Cry1Ia42,Cry2Aa22,Cry2Ab41,Cry1Ba8,Vip3Aa79,Mpp5Ab1,Spp1Aa1,Vpa2Bb4	8	Yes	cry1I,cry2A,cry2A,cry1B,vip3Aa,Mpp5A,Spp1A	Coleoptera;Lepidoptera;Rhabditida;Diptera;Hemiptera;Blattodea;Aedes aegypti	*Leptinotarsa decemlineata;Ostrinia furnacalis;Lobesia botrana;Plutella xylostella;Earias insulana;cotton boll weevil;Meloidogyne incognita;Helicoverpa armigera;Aedes aegypti;Diaphorina citri;Ostrinia nubilalis;Spodoptera frugiperda;Maruca vitrata;Manduca sexta;Helicoverpa punctigera;Helicoverpa zea;Chilo partellus;Blattella germanica;Colaphellus bowringi;Spodoptera litura*
GCF_036212705.1	*Bacillus thuringiensis*	NRS-1464	Cry1Ba8,Cry1Ab9,Mpp5Ab1,Spp1Aa1,Vpa2Bb4,Vpb1Bb2,Vpb1Bc1	7	Yes	cry1B,cry1A,Mpp5A,Spp1A	Lepidoptera;Hemiptera;Diptera;Coleoptera;Blattodea	*Plutella xylostella;Ostrinia nubilalis;Spodoptera frugiperda;Maruca vitrata;Manduca sexta;cotton boll weevil;Ostrinia furnacalis;Helicoverpa armigera;Leucinodes orbonalis;Trichoplusia ni;Hyphantria*

								cunea;Pieris rapae;Chilo suppressalis;Colaphellus bowringi;Leptinotarsa decemlineata;Spodoptera litura;Blattella germanica
GCF_036212625.1	*Bacillus thuringiensis*	NRS-1554	Cry1Ba8,Cry1Ab9,Mpp5Ab1,Spp1Aa1,Vpa2Bb4,Vpb1Bb2,Vpb1Bc1	7	Yes	cry1B,cry1A,Mpp5A,Spp1A	Lepidoptera;Hemiptera;Diptera;Coleoptera;Blattodea	*Plutella xylostella;Ostrinia nubilalis;Spodoptera frugiperda;Maruca vitrata;Manduca sexta;cotton boll weevil;Ostrinia furnacalis;Helicoverpa armigera;Leucinodes orbonalis;Trichoplusia ni;Hyphantria cunea;Pieris rapae;Chilo suppressalis;Colaphellus bowringi;Leptinotarsa decemlineata;Spodoptera litura;Blattella germanica*
GCF_035818235.1	*Bacillus thuringiensis*	HD-631	Cry1Aa9,Spp1Aa1,Vpa2Ae3,Vpb1Ac2	4	Yes	Vpa2Ae + Vpb1Ac,cry1A,Spp1A	Hemiptera;Lepidoptera;Blattodea	*Aphis gossypii;Plutella xylostella;Ostrinia furnacalis;Helicoverpa armigera;Leucinodes orbonalis;Trichoplusia ni;Hyphantria cunea;Spodoptera frugiperda;Pieris rapae;Chilo suppressalis;Spodoptera litura;Blattella germanica*
GCF_035818195.1	*Bacillus thuringiensis*	HD-633	Cry1Aa9,Spp1Aa1	2	Yes	cry1A,Spp1A	Lepidoptera;Blattodea	*Plutella xylostella;Ostrinia furnacalis;Helicoverpa armigera;Leucinodes*

								orbonalis;Trichoplusia ni;Hyphantria cunea;Spodoptera frugiperda;Pieris rapae;Chilo suppressalis;Spodoptera litura;Blattella germanica
GCF_035818175.1	*Bacillus thuringiensis*	HD-634	Cry1Aa9,Spp1Aa1,Vpb1Ac2	3	Yes	cry1A,Spp1A	Lepidoptera;Blattodea	*Plutella xylostella;Ostrinia furnacalis;Helicoverpa armigera;Leucinodes orbonalis;Trichoplusia ni;Hyphantria cunea;Spodoptera frugiperda;Pieris rapae;Chilo suppressalis;Spodoptera litura;Blattella germanica*
GCF_035818115.1	*Bacillus thuringiensis*	HD-653	Mpp60Ba3,Mpp60Aa3	2	No	Mpp60B,Mpp60A	Diptera	*Culex quinquefasciatus*
GCF_035818015.1	*Bacillus thuringiensis*	HD-662	Cry1Aa9,Spp1Aa1	2	Yes	cry1A,Spp1A	Lepidoptera;Blattodea	*Plutella xylostella;Ostrinia furnacalis;Helicoverpa armigera;Leucinodes orbonalis;Trichoplusia ni;Hyphantria cunea;Spodoptera frugiperda;Pieris rapae;Chilo suppressalis;Spodoptera litura;Blattella germanica*
GCF_035817965.1	*Bacillus thuringiensis*	HD-668	Cry1Aa9,Spp1Aa1	2	Yes	cry1A,Spp1A	Lepidoptera;Blattodea	*Plutella xylostella;Ostrinia furnacalis;Helicoverpa armigera;Leucinodes orbonalis;Trichoplusia ni;Hyphantria cunea;Spodoptera frugiperda;Pieris rapae;Chilo suppressalis;Spodoptera litura;Blattella germanica*

GCF_035817955.1	*Bacillus thuringiensis*	HD-667	Cry1Aa9,Spp1Aa1	2	Yes	cry1A,Spp1A	Lepidoptera;Blattodea	*Plutella xylostella;Ostrinia furnacalis;Helicoverpa armigera;Leucinodes orbonalis;Trichoplusia ni;Hyphantria cunea;Spodoptera frugiperda;Pieris rapae;Chilo suppressalis;Spodoptera litura;Blattella germanica*
GCF_035817935.1	*Bacillus thuringiensis*	HD-666	Cry1Aa9,Spp1Aa1	2	Yes	cry1A,Spp1A	Lepidoptera;Blattodea	*Plutella xylostella;Ostrinia furnacalis;Helicoverpa armigera;Leucinodes orbonalis;Trichoplusia ni;Hyphantria cunea;Spodoptera frugiperda;Pieris rapae;Chilo suppressalis;Spodoptera litura;Blattella germanica*
GCF_035817915.1	*Bacillus thuringiensis*	HD-669	Cry1Aa9,Spp1Aa1	2	Yes	cry1A,Spp1A	Lepidoptera;Blattodea	*Plutella xylostella;Ostrinia furnacalis;Helicoverpa armigera;Leucinodes orbonalis;Trichoplusia ni;Hyphantria cunea;Spodoptera frugiperda;Pieris rapae;Chilo suppressalis;Spodoptera litura;Blattella germanica*
GCF_035817895.1	*Bacillus thuringiensis*	HD-670	Cry1Aa9,Spp1Aa1	2	Yes	cry1A,Spp1A	Lepidoptera;Blattodea	*Plutella xylostella;Ostrinia furnacalis;Helicoverpa armigera;Leucinodes orbonalis;Trichoplusia ni;Hyphantria cunea;Spodoptera frugiperda;Pieris rapae;Chilo suppressalis;Spodoptera litura;Blattella germanica*

GCF_035817875.1	*Bacillus thuringiensis*	HD-677	Cry1Aa9,Spp1Aa1	2	Yes	cry1A,Spp1A	Lepidoptera;Blattodea	*Plutella xylostella;Ostrinia furnacalis;Helicoverpa armigera;Leucinodes orbonalis;Trichoplusia ni;Hyphantria cunea;Spodoptera frugiperda;Pieris rapae;Chilo suppressalis;Spodoptera litura;Blattella germanica*
GCF_035817855.1	*Bacillus thuringiensis*	HD-672	Cry1Aa9,Spp1Aa1	2	Yes	cry1A,Spp1A	Lepidoptera;Blattodea	*Plutella xylostella;Ostrinia furnacalis;Helicoverpa armigera;Leucinodes orbonalis;Trichoplusia ni;Hyphantria cunea;Spodoptera frugiperda;Pieris rapae;Chilo suppressalis;Spodoptera litura;Blattella germanica*
GCF_035817835.1	*Bacillus thuringiensis*	HD-674	Cry1Aa9,Spp1Aa1	2	Yes	cry1A,Spp1A	Lepidoptera;Blattodea	*Plutella xylostella;Ostrinia furnacalis;Helicoverpa armigera;Leucinodes orbonalis;Trichoplusia ni;Hyphantria cunea;Spodoptera frugiperda;Pieris rapae;Chilo suppressalis;Spodoptera litura;Blattella germanica*
GCF_035817815.1	*Bacillus thuringiensis*	HD-678	Cry1Aa9,Spp1Aa1	2	Yes	cry1A,Spp1A	Lepidoptera;Blattodea	*Plutella xylostella;Ostrinia furnacalis;Helicoverpa armigera;Leucinodes orbonalis;Trichoplusia ni;Hyphantria cunea;Spodoptera frugiperda;Pieris rapae;Chilo suppressalis;Spodoptera litura;Blattella germanica*

GCF_035817795.1	*Bacillus thuringiensis*	HD-680	Cry1Aa9,Spp1Aa1	2	Yes	cry1A,Spp1A	Lepidoptera;Blattodea	*Plutella xylostella;Ostrinia furnacalis;Helicoverpa armigera;Leucinodes orbonalis;Trichoplusia ni;Hyphantria cunea;Spodoptera frugiperda;Pieris rapae;Chilo suppressalis;Spodoptera litura;Blattella germanica*
GCF_035817775.1	*Bacillus thuringiensis*	HD-683	Cry1Aa9,Spp1Aa1	2	Yes	cry1A,Spp1A	Lepidoptera;Blattodea	*Plutella xylostella;Ostrinia furnacalis;Helicoverpa armigera;Leucinodes orbonalis;Trichoplusia ni;Hyphantria cunea;Spodoptera frugiperda;Pieris rapae;Chilo suppressalis;Spodoptera litura;Blattella germanica*
GCF_035817735.1	*Bacillus thuringiensis*	HD-688	Cry1Aa9,Spp1Aa1	2	Yes	cry1A,Spp1A	Lepidoptera;Blattodea	*Plutella xylostella;Ostrinia furnacalis;Helicoverpa armigera;Leucinodes orbonalis;Trichoplusia ni;Hyphantria cunea;Spodoptera frugiperda;Pieris rapae;Chilo suppressalis;Spodoptera litura;Blattella germanica*
GCF_035817715.1	*Bacillus thuringiensis*	HD-691	Cry1Aa9,Spp1Aa1	2	Yes	cry1A,Spp1A	Lepidoptera;Blattodea	*Plutella xylostella;Ostrinia furnacalis;Helicoverpa armigera;Leucinodes orbonalis;Trichoplusia ni;Hyphantria cunea;Spodoptera frugiperda;Pieris rapae;Chilo suppressalis;Spodoptera litura;Blattella germanica*

GCF_035817695.1	*Bacillus thuringiensis*	HD-702	Cry1Ba8,Cry1Aa3,Cry1Ia42,Cry2Aa22,Cry2Ab41,Vip3Aa79,Mpp5Ab1,Spp1Aa1,Vpa2Bb4,Vpb1Bb2,Vpb1Bc1	11	Yes	cry1B,cry1A,cry1I,cry2A,cry2A,vip3Aa,Mpp5A,Spp1A	Lepidoptera;Hemiptera;Diptera;Coleoptera;Rhabditida;Blattodea;Aedes aegypti	*Plutella xylostella;Ostrinia nubilalis;Spodoptera frugiperda;Maruca vitrata;Manduca sexta;cotton boll weevil;Ostrinia furnacalis;Helicoverpa armigera;Leucinodes orbonalis;Trichoplusia ni;Hyphantria cunea;Pieris rapae;Chilo suppressalis;Leptinotarsa decemlineata;Lobesia botrana;Earias insulana;Meloidogyne incognita;Aedes aegypti;Diaphorina citri;Helicoverpa punctigera;Helicoverpa zea;Chilo partellus;Blattella germanica;Colaphellus bowringi;Spodoptera litura*
GCF_035817635.1	*Bacillus thuringiensis*	HD-703	Cry1Ba8,Cry1Ia42,Cry2Aa22,Cry2Ab41,Cry1Aa17,Vip3Aa79,Mpp5Ab1,Spp1Aa1,Vpa2Bb4,Vpb1Bb2,Vpb1Bc1	11	Yes	cry1B,cry1I,cry2A,cry2A,cry1A,vip3Aa,Mpp5A,Spp1A	Lepidoptera;Hemiptera;Diptera;Coleoptera;Rhabditida;Blattodea;Aedes aegypti	*Plutella xylostella;Ostrinia nubilalis;Spodoptera frugiperda;Maruca vitrata;Manduca sexta;cotton boll weevil;Leptinotarsa decemlineata;Ostrinia furnacalis;Lobesia botrana;Earias insulana;Meloidogyne incognita;Helicoverpa armigera;Aedes aegypti;Diaphorina citri;Leucinodes orbonalis;Trichoplusia ni;Hyphantria*

								cunea;Pieris rapae;Chilo suppressalis;Helicoverpa punctigera;Helicoverpa zea;Chilo partellus;Blattella germanica;Colaphellus bowringi;Spodoptera litura
GCF_035817615.1	*Bacillus thuringiensis*	HD-692	Cry1Aa9,Spp1Aa1	2	Yes	cry1A,Spp1A	Lepidoptera;Blattodea	*Plutella xylostella;Ostrinia furnacalis;Helicoverpa armigera;Leucinodes orbonalis;Trichoplusia ni;Hyphantria cunea;Spodoptera frugiperda;Pieris rapae;Chilo suppressalis;Spodoptera litura;Blattella germanica*
GCF_035817585.1	*Bacillus thuringiensis*	HD-706	Cry1Ba8,Cry1Aa3,Cry1Ia42,Cry2Aa22,Cry2Ab41,Vip3Aa79,Mpp5Ab1,Spp1Aa1,Vpa2Bb4,Vpb1Bb2,Vpb1Bc1	11	Yes	cry1B,cry1A,cry1I,cry2A,cry2A,vip3Aa,Mpp5A,Spp1A	Lepidoptera;Hemiptera;Diptera;Coleoptera;Rhabditida;Blattodea;Aedes aegypti	*Plutella xylostella;Ostrinia nubilalis;Spodoptera frugiperda;Maruca vitrata;Manduca sexta;cotton boll weevil;Ostrinia furnacalis;Helicoverpa armigera;Leucinodes orbonalis;Trichoplusia ni;Hyphantria cunea;Pieris rapae;Chilo suppressalis;Leptinotarsa decemlineata;Lobesia botrana;Earias insulana;Meloidogyne incognita;Aedes aegypti;Diaphorina citri;Helicoverpa punctigera;Helicoverpa zea;Chilo partellus;Blattella*

								germanica;Colaphellus bowringi;Spodoptera litura
GCF_035817555.1	*Bacillus thuringiensis*	HD-710	Cry1Bb1,Cry1Ab24,Cry1Hb2,Cry1Ka1,Cry1Ib5,Cry1Fb4,Vip3Ag6,Spp1Aa1,Vpa2Ac2,Vpb1Ca1	10	No	cry1B,cry1A,cry1H,cry1K,cry1I,cry1F,vip3Ag,Spp1A	Lepidoptera;Hemiptera;Diptera;Coleoptera;Rhabditida;Blattodea	*Plutella xylostella;Ostrinia nubilalis;Spodoptera frugiperda;Maruca vitrata;Manduca sexta;cotton boll weevil;Ostrinia furnacalis;Helicoverpa armigera;Leucinodes orbonalis;Trichoplusia ni;Hyphantria cunea;Pieris rapae;Chilo suppressalis;Spodoptera exigua;Artogeia rapae;Leptinotarsa decemlineata;Lobesia botrana;Earias insulana;Meloidogyne incognita;Agrotis ipsilon;Diabrotica virgifera virgifera;Spodoptera littoralis;Chrysodeixis chalcites;Spodoptera litura;Blattella germanica*
GCF_035817475.1	*Bacillus thuringiensis*	HD-724	Cry1Ba8,Cry1Ab9,Spp1Aa1	3	Yes	cry1B,cry1A,Spp1A	Lepidoptera;Hemiptera;Diptera;Coleoptera;Blattodea	*Plutella xylostella;Ostrinia nubilalis;Spodoptera frugiperda;Maruca vitrata;Manduca sexta;cotton boll weevil;Ostrinia furnacalis;Helicoverpa armigera;Leucinodes orbonalis;Trichoplusia ni;Hyphantria cunea;Pieris rapae;Chilo suppressalis;Spodoptera*

								litura;Blattella germanica
GCF_035817435.1	*Bacillus thuringiensis*	HD-739	Spp1Aa1	1	Yes	Spp1A	Lepidoptera;Blattodea	*Spodoptera litura;Blattella germanica*
GCF_035817335.1	*Bacillus thuringiensis*	HD-757	Mpp60Ba3,Mpp60Aa3	2	No	Mpp60B,Mpp60A	Diptera	*Culex quinquefasciatus*
GCF_035817255.1	*Bacillus thuringiensis*	HD-752	Cry1Ba8,Mpp5Ab1,Spp1Aa1,Vpa2Bb4	4	Yes	cry1B,Mpp5A,Spp1A	Lepidoptera;Hemiptera;Diptera;Coleoptera;Blattodea	*Plutella xylostella;Ostrinia nubilalis;Spodoptera frugiperda;Maruca vitrata;Manduca sexta;cotton boll weevil;Colaphellus bowringi;Leptinotarsa decemlineata;Spodoptera litura;Blattella germanica*
GCF_035817185.1	*Bacillus thuringiensis*	HD-759	Cry1Ab37,Cry1Ia42,Cry1Bd2,Vip3Ah2	4	No	cry1A,cry1I,cry1B,vip3Ah	Lepidoptera;Coleoptera;Rhabditida;Hemiptera;Diptera	*Plutella xylostella;Ostrinia furnacalis;Helicoverpa armigera;Leucinodes orbonalis;Trichoplusia ni;Hyphantria cunea;Spodoptera frugiperda;Pieris rapae;Chilo suppressalis;Leptinotarsa decemlineata;Lobesia botrana;Earias insulana;cotton boll weevil;Meloidogyne incognita;Ostrinia nubilalis;Maruca vitrata;Manduca sexta;Agrotis ipsilon*
GCF_035	*Bacillus*	HD-76	Cry1Bb1,Cry1Da3,Cry	9	No	cry1B,cry1D,cry1H,cr	Lepidoptera;Hemi	*Plutella xylostella;Ostrinia*

817175.1	*thuringiensis*	7	1Hb2,Cry1Id2,Cry1Ja1,Cry1Ab24,Spp1Aa1,Vpa2Ac2,Vpb1Ca1			y1I,cry1J,cry1A,Spp1A	ptera;Diptera;Coleoptera;Rhabditida;Blattodea	*nubilalis;Spodoptera frugiperda;Maruca vitrata;Manduca sexta;cotton boll weevil;Spodoptera exigua;Bombyx mori;Spodoptera litura;Epiphyas postvittana;Ostrinia furnacalis;Leptinotarsa decemlineata;Lobesia botrana;Earias insulana;Meloidogyne incognita;Diabrotica undecimpunctata howardi;Helicoverpa armigera;Leucinodes orbonalis;Trichoplusia ni;Hyphantria cunea;Pieris rapae;Chilo suppressalis;Blattella germanica*
GCF_035817115.1	*Bacillus thuringiensis*	HD-770	Cry1Ba8,Cry1Ab9,Spp1Aa1	3	Yes	cry1B,cry1A,Spp1A	Lepidoptera;Hemiptera;Diptera;Coleoptera;Blattodea	*Plutella xylostella;Ostrinia nubilalis;Spodoptera frugiperda;Maruca vitrata;Manduca sexta;cotton boll weevil;Ostrinia furnacalis;Helicoverpa armigera;Leucinodes orbonalis;Trichoplusia ni;Hyphantria cunea;Pieris rapae;Chilo suppressalis;Spodoptera litura;Blattella germanica*
GCF_035817075.1	*Bacillus thuringiensis*	HD-773	Cry1Aa9,Spp1Aa1	2	Yes	cry1A,Spp1A	Lepidoptera;Blattodea	*Plutella xylostella;Ostrinia furnacalis;Helicoverpa armigera;Leucinodes*

								orbonalis;Trichoplusia ni;Hyphantria cunea;Spodoptera frugiperda;Pieris rapae;Chilo suppressalis;Spodoptera litura;Blattella germanica
GCF_035817055.1	*Bacillus thuringiensis*	HD-772	Cry1Aa9,Vip3Aa80,Spp1Aa1,Vpa2Ae3,Vpb1Ac2	5	Yes	Vpa2Ae + Vpb1Ac,cry1A,vip3Aa,Spp1A	Hemiptera;Lepidoptera;Blattodea;Aedes aegypti	*Aphis gossypii;Plutella xylostella;Ostrinia furnacalis;Helicoverpa armigera;Leucinodes orbonalis;Trichoplusia ni;Hyphantria cunea;Spodoptera frugiperda;Pieris rapae;Chilo suppressalis;Helicoverpa punctigera;Helicoverpa zea;Chilo partellus;Blattella germanica;Spodoptera litura*
GCF_035816825.1	*Bacillus thuringiensis*	HD-797	Spp1Aa1	1	No	Spp1A	Lepidoptera;Blattodea	*Spodoptera litura;Blattella germanica*
GCF_035816815.1	*Bacillus thuringiensis*	HD-792	Cry11Aa3,Cry10Aa4,Cyt1Ca1,Cyt1Aa11,Cyt2Ba17,Mpp60Ba3,Mpp60Aa3	7	No	cry11A,cry10A,cyt1A,cyt2B,Mpp60B,Mpp60A	Diptera;Coleoptera;Hemiptera;Lepidoptera	*Aedes aegypti;Anopheles albimanus;Culex quinquefasciatu;Culex pipiens;cotton boll weevil;Culex quinquefasciatus;Culex pipien;Diaphorina citri;Plutella xylostella;Anopheles stephensi;Anopheles gambiae;Bradysia difformis*
GCF_035	*Bacillus*	HD-79	Cry11Aa3,Cry10Aa4,C	7	No	cry11A,cry10A,cyt1A	Diptera;Coleopter	*Aedes aegypti;Anopheles*

816755.1	*thuringiensis*	8	yt1Ca1,Cyt1Aa11,Cyt2Ba17,Mpp60Ba3,Mpp60Aa3			,cyt2B,Mpp60B,Mpp60A	a;Hemiptera;Lepidoptera	*albimanus;Culex quinquefasciatu;Culex pipiens;cotton boll weevil;Culex quinquefasciatus;Culex pipien;Diaphorina citri;Plutella xylostella;Anopheles stephensi;Anopheles gambiae;Bradysia difformis*
GCF_035816655.1	*Bacillus thuringiensis*	HD-813	Cry1Ba8,Cry1Aa3,Cry1Ia42,Cry2Aa22,Vip3Aa79,Mpp5Ab1,Spp1Aa1,Vpa2Bb4,Vpb1Bb2,Vpb1Bc1	10	Yes	cry1B,cry1A,cry1I,cry2A,vip3Aa,Mpp5A,Spp1A	Lepidoptera;Hemiptera;Diptera;Coleoptera;Rhabditida;Blattodea;Aedes aegypti	*Plutella xylostella;Ostrinia nubilalis;Spodoptera frugiperda;Maruca vitrata;Manduca sexta;cotton boll weevil;Ostrinia furnacalis;Helicoverpa armigera;Leucinodes orbonalis;Trichoplusia ni;Hyphantria cunea;Pieris rapae;Chilo suppressalis;Leptinotarsa decemlineata;Lobesia botrana;Earias insulana;Meloidogyne incognita;Aedes aegypti;Diaphorina citri;Helicoverpa punctigera;Helicoverpa zea;Chilo partellus;Blattella germanica;Colaphellus bowringi;Spodoptera litura*
GCF_035816615.1	*Bacillus thuringiensis*	HD-814	Cry11Aa3,Cry10Aa4,Cyt1Ca1,Cyt1Aa11,Cyt2Ba17,Mpp60Ba3,Mpp6	7	No	cry11A,cry10A,cyt1A,cyt2B,Mpp60B,Mpp60A	Diptera;Coleoptera;Hemiptera;Lepidoptera	*Aedes aegypti;Anopheles albimanus;Culex quinquefasciatu;Culex pipiens;cotton*

			0Aa3					*boll weevil;Culex quinquefasciatus;Culex pipien;Diaphorina citri;Plutella xylostella;Anopheles stephensi;Anopheles gambiae;Bradysia difformis*
GCF_035816455.1	*Bacillus thuringiensis*	HD-823	Cry1Ba8,Cry1Aa3,Cry1Ia42,Cry2Aa22,Vip3Aa79,Mpp5Ab1,Spp1Aa1,Vpa2Bb4,Vpb1Bb2,Vpb1Bc1	10	Yes	cry1B,cry1A,cry1I,cry2A,vip3Aa,Mpp5A,Spp1A	Lepidoptera;Hemiptera;Diptera;Coleoptera;Rhabditida;Blattodea;Aedes aegypti	*Plutella xylostella;Ostrinia nubilalis;Spodoptera frugiperda;Maruca vitrata;Manduca sexta;cotton boll weevil;Ostrinia furnacalis;Helicoverpa armigera;Leucinodes orbonalis;Trichoplusia ni;Hyphantria cunea;Pieris rapae;Chilo suppressalis;Leptinotarsa decemlineata;Lobesia botrana;Earias insulana;Meloidogyne incognita;Aedes aegypti;Diaphorina citri;Helicoverpa punctigera;Helicoverpa zea;Chilo partellus;Blattella germanica;Colaphellus bowringi;Spodoptera litura*
GCF_035815975.1	*Bacillus thuringiensis*	HD-876	Cry1Ba8,Cry1Aa3,Cry1Ia42,Cry2Aa22,Cry2Ab41,Vip3Aa79,Mpp5Ab1,Spp1Aa1,Vpa2Bb4,Vpb1Bb2,Vpb1Bc1	11	Yes	cry1B,cry1A,cry1I,cry2A,cry2A,vip3Aa,Mpp5A,Spp1A	Lepidoptera;Hemiptera;Diptera;Coleoptera;Rhabditida;Blattodea;Aedes aegypti	*Plutella xylostella;Ostrinia nubilalis;Spodoptera frugiperda;Maruca vitrata;Manduca sexta;cotton boll weevil;Ostrinia furnacalis;Helicoverpa*

								armigera;Leucinodes orbonalis;Trichoplusia ni;Hyphantria cunea;Pieris rapae;Chilo suppressalis;Leptinotarsa decemlineata;Lobesia botrana;Earias insulana;Meloidogyne incognita;Aedes aegypti;Diaphorina citri;Helicoverpa punctigera;Helicoverpa zea;Chilo partellus;Blattella germanica;Colaphellus bowringi;Spodoptera litura
GCF_035815835.1	*Bacillus thuringiensis*	HD-882	Cry1Aa9,Spp1Aa1	2	Yes	cry1A,Spp1A	Lepidoptera;Blattodea	*Plutella xylostella;Ostrinia furnacalis;Helicoverpa armigera;Leucinodes orbonalis;Trichoplusia ni;Hyphantria cunea;Spodoptera frugiperda;Pieris rapae;Chilo suppressalis;Spodoptera litura;Blattella germanica*
GCF_035815815.1	*Bacillus thuringiensis*	HD-887	Cry1Aa9,Spp1Aa1	2	Yes	cry1A,Spp1A	Lepidoptera;Blattodea	*Plutella xylostella;Ostrinia furnacalis;Helicoverpa armigera;Leucinodes orbonalis;Trichoplusia ni;Hyphantria cunea;Spodoptera frugiperda;Pieris rapae;Chilo suppressalis;Spodoptera litura;Blattella germanica*
GCF_035815795.1	*Bacillus thuringi*	HD-888	Cry1Aa9,Spp1Aa1	2	Yes	cry1A,Spp1A	Lepidoptera;Blattodea	*Plutella xylostella;Ostrinia furnacalis;Helicoverpa*

	ensis							*armigera;Leucinodes orbonalis;Trichoplusia ni;Hyphantria cunea;Spodoptera frugiperda;Pieris rapae;Chilo suppressalis;Spodoptera litura;Blattella germanica*
GCF_035815775.1	*Bacillus thuringiensis*	HD-890	Cry1Aa9,Spp1Aa1	2	Yes	cry1A,Spp1A	Lepidoptera;Blattodea	*Plutella xylostella;Ostrinia furnacalis;Helicoverpa armigera;Leucinodes orbonalis;Trichoplusia ni;Hyphantria cunea;Spodoptera frugiperda;Pieris rapae;Chilo suppressalis;Spodoptera litura;Blattella germanica*
GCF_035815755.1	*Bacillus thuringiensis*	HD-889	Cry1Aa9,Spp1Aa1	2	Yes	cry1A,Spp1A	Lepidoptera;Blattodea	*Plutella xylostella;Ostrinia furnacalis;Helicoverpa armigera;Leucinodes orbonalis;Trichoplusia ni;Hyphantria cunea;Spodoptera frugiperda;Pieris rapae;Chilo suppressalis;Spodoptera litura;Blattella germanica*
GCF_035815735.1	*Bacillus thuringiensis*	HD-891	Cry1Aa9,Spp1Aa1	2	Yes	cry1A,Spp1A	Lepidoptera;Blattodea	*Plutella xylostella;Ostrinia furnacalis;Helicoverpa armigera;Leucinodes orbonalis;Trichoplusia ni;Hyphantria cunea;Spodoptera frugiperda;Pieris rapae;Chilo suppressalis;Spodoptera litura;Blattella germanica*
GCF_035815715.1	*Bacillus thuringi*	HD-894	Cry1Aa9,Spp1Aa1	2	No	cry1A,Spp1A	Lepidoptera;Blattodea	*Plutella xylostella;Ostrinia furnacalis;Helicoverpa*

	ensis							*armigera;Leucinodes orbonalis;Trichoplusia ni;Hyphantria cunea;Spodoptera frugiperda;Pieris rapae;Chilo suppressalis;Spodoptera litura;Blattella germanica*
GCF_035815425.1	*Bacillus thuringiensis*	HD-919	Cry11Aa3,Cry10Aa4,Cyt1Ca1,Cyt1Aa11,Cyt2Ba17,Mpp60Ba3,Mpp60Aa3	7	No	cry11A,cry10A,cyt1A,cyt2B,Mpp60B,Mpp60A	Diptera;Coleoptera;Hemiptera;Lepidoptera	*Aedes aegypti;Anopheles albimanus;Culex quinquefasciatu;Culex pipiens;cotton boll weevil;Culex quinquefasciatus;Culex pipien;Diaphorina citri;Plutella xylostella;Anopheles stephensi;Anopheles gambiae;Bradysia difformis*
GCF_035815295.1	*Bacillus thuringiensis*	HD-930	Cry1Aa9,Spp1Aa1	2	Yes	cry1A,Spp1A	Lepidoptera;Blattodea	*Plutella xylostella;Ostrinia furnacalis;Helicoverpa armigera;Leucinodes orbonalis;Trichoplusia ni;Hyphantria cunea;Spodoptera frugiperda;Pieris rapae;Chilo suppressalis;Spodoptera litura;Blattella germanica*
GCF_035815275.1	*Bacillus thuringiensis*	HD-931	Cry1Ba8,Cry1Ia42,Cry2Aa22,Cry2Ab41,Vip3Aa79,Mpp5Ab1,Spp1Aa1,Vpa2Bb4,Vpb1Bb2,Vpb1Bc1	10	Yes	cry1B,cry1I,cry2A,cry2A,vip3Aa,Mpp5A,Spp1A	Lepidoptera;Hemiptera;Diptera;Coleoptera;Rhabditida;Blattodea;Aedes aegypti	*Plutella xylostella;Ostrinia nubilalis;Spodoptera frugiperda;Maruca vitrata;Manduca sexta;cotton boll weevil;Leptinotarsa decemlineata;Ostrinia furnacalis;Lobesia botrana;Earias insulana;Meloidogyne*

								incognita;Helicoverpa armigera;Aedes aegypti;Diaphorina citri;Helicoverpa punctigera;Helicoverpa zea;Chilo partellus;Blattella germanica;Colaphellus bowringi;Spodoptera litura
GCF_035815215.1	*Bacillus thuringiensis*	HD-932	Cry7Kb1,Cry7La1,Cry7Gc1,App4Aa1,Spp1Aa1	5	No	cry7G,App4A,Spp1A	Coleoptera;Lepidoptera;Hemiptera;Blattodea	*Diabrotica undecimpunctata howardi;Diabrotica virgifera virgifera;Plutella xylostella;Laodelphax striatellus;Spodoptera litura;Blattella germanica*
GCF_035814735.1	*Bacillus thuringiensis*	HD-963	Cry1Aa9,Spp1Aa1	2	Yes	cry1A,Spp1A	Lepidoptera;Blattodea	*Plutella xylostella;Ostrinia furnacalis;Helicoverpa armigera;Leucinodes orbonalis;Trichoplusia ni;Hyphantria cunea;Spodoptera frugiperda;Pieris rapae;Chilo suppressalis;Spodoptera litura;Blattella germanica*
GCF_035814675.1	*Bacillus thuringiensis*	HD-975	Cry1Aa9,Spp1Aa1	2	Yes	cry1A,Spp1A	Lepidoptera;Blattodea	*Plutella xylostella;Ostrinia furnacalis;Helicoverpa armigera;Leucinodes orbonalis;Trichoplusia ni;Hyphantria cunea;Spodoptera frugiperda;Pieris rapae;Chilo suppressalis;Spodoptera litura;Blattella germanica*
GCF_035	*Bacillus*	HD-68	Cry1Aa9,Spp1Aa1	2	Yes	cry1A,Spp1A	Lepidoptera;Blatto	*Plutella xylostella;Ostrinia*

726685.1	*thuringiensis*	1					dea	*furnacalis;Helicoverpa armigera;Leucinodes orbonalis;Trichoplusia ni;Hyphantria cunea;Spodoptera frugiperda;Pieris rapae;Chilo suppressalis;Spodoptera litura;Blattella germanica*
GCF_035726645.1	*Bacillus thuringiensis*	HD-727	Cry1Ba8,Cry1Ab9,Spp1Aa1	3	Yes	cry1B,cry1A,Spp1A	Lepidoptera;Hemiptera;Diptera;Coleoptera;Blattodea	*Plutella xylostella;Ostrinia nubilalis;Spodoptera frugiperda;Maruca vitrata;Manduca sexta;cotton boll weevil;Ostrinia furnacalis;Helicoverpa armigera;Leucinodes orbonalis;Trichoplusia ni;Hyphantria cunea;Pieris rapae;Chilo suppressalis;Spodoptera litura;Blattella germanica*
GCF_034365905.1	*Bacillus thuringiensis*	SS7	Cry1Ia42,Cry2Aa22,Cry2Ab41,Vip3Aa79,Spp1Aa1	5	Yes	cry1I,cry2A,cry2A,vip3Aa,Spp1A	Coleoptera;Lepidoptera;Rhabditida;Diptera;Hemiptera;Blattodea;Aedes aegypti	*Leptinotarsa decemlineata;Ostrinia furnacalis;Lobesia botrana;Plutella xylostella;Earias insulana;cotton boll weevil;Meloidogyne incognita;Helicoverpa armigera;Aedes aegypti;Diaphorina citri;Helicoverpa punctigera;Spodoptera frugiperda;Helicoverpa zea;Chilo partellus;Blattella germanica;Spodoptera litura*
GCF_034	*Bacillus*	SS8	Spp1Aa1,Xpp22Aa3	2	Yes	Spp1A,Xpp22A	Lepidoptera;Blatto	*Spodoptera litura;Blattella*

365855.1	*thuringiensis*						dea;Coleoptera	*germanica;Anthonomus grandis*
GCF_034318185.1	*Bacillus thuringiensis*	SS2	Cry24Da1,Spp1Aa1	2	Yes	Spp1A	Lepidoptera;Blattodea	*Spodoptera litura;Blattella germanica*
GCF_034317685.1	*Bacillus thuringiensis*	SS4	Cry1Ia42,Cry2Aa22,Cry2Ab41,Vip3Aa79,Mpp5Ab1,Spp1Aa1,Vpa2Bb4,Vpb1Bb2,Vpb1Bc1	9	Yes	cry1I,cry2A,cry2A,vip3Aa,Mpp5A,Spp1A	Coleoptera;Lepidoptera;Rhabditida;Diptera;Hemiptera;Blattodea;Aedes aegypti	*Leptinotarsa decemlineata;Ostrinia furnacalis;Lobesia botrana;Plutella xylostella;Earias insulana;cotton boll weevil;Meloidogyne incognita;Helicoverpa armigera;Aedes aegypti;Diaphorina citri;Helicoverpa punctigera;Spodoptera frugiperda;Helicoverpa zea;Chilo partellus;Blattella germanica;Colaphellus bowringi;Spodoptera litura*
GCF_034311005.1	*Bacillus thuringiensis*	Bt_UNVM-84	Vpa2Ai2	1	Yes			
GCF_034296225.1	*Bacillus thuringiensis*	SS3	Cry1Ia42,Cry2Aa22,Cry2Ab41,Vip3Aa79,Spp1Aa1	5	Yes	cry1I,cry2A,cry2A,vip3Aa,Spp1A	Coleoptera;Lepidoptera;Rhabditida;Diptera;Hemiptera;Blattodea;Aedes aegypti	*Leptinotarsa decemlineata;Ostrinia furnacalis;Lobesia botrana;Plutella xylostella;Earias insulana;cotton boll weevil;Meloidogyne incognita;Helicoverpa armigera;Aedes aegypti;Diaphorina citri;Helicoverpa punctigera;Spodoptera*

								frugiperda;Helicoverpa zea;Chilo partellus;Blattella germanica;Spodoptera litura
GCF_034045075.1	*Bacillus thuringiensis*	CFBP 9014	App4Aa1,Spp1Aa1	2	No	App4A,Spp1A	Lepidoptera;Hemiptera;Blattodea	*Plutella xylostella;Laodelphax striatellus;Spodoptera litura;Blattella germanica*
GCF_033764115.1	*Bacillus thuringiensis*	800/15	Cry1Ba8,Cry1Ab9,Spp1Aa1	3	Yes	cry1B,cry1A,Spp1A	Lepidoptera;Hemiptera;Diptera;Coleoptera;Blattodea	*Plutella xylostella;Ostrinia nubilalis;Spodoptera frugiperda;Maruca vitrata;Manduca sexta;cotton boll weevil;Ostrinia furnacalis;Helicoverpa armigera;Leucinodes orbonalis;Trichoplusia ni;Hyphantria cunea;Pieris rapae;Chilo suppressalis;Spodoptera litura;Blattella germanica*
GCF_033663765.1	*Bacillus thuringiensis serovar toumanoffi*	4059	Cry1Bb1,Cry1Ab24,Cry1Ja2,Cry1Da3,Cry1Hb2,Cry1Id2,Cry1Nb1,Cry2Ad1,Vip3Af3,App4Aa1,Spp1Aa1,Vpa2Ac2,Vpb1Ca1	13	Yes	cry1B,cry1A,cry1J,cry1D,cry1H,cry1I,cry1N,cry2A,vip3Af,App4A,Spp1A	Lepidoptera;Hemiptera;Diptera;Coleoptera;Rhabditida;Blattodea	*Plutella xylostella;Ostrinia nubilalis;Spodoptera frugiperda;Maruca vitrata;Manduca sexta;cotton boll weevil;Ostrinia furnacalis;Helicoverpa armigera;Leucinodes orbonalis;Trichoplusia ni;Hyphantria cunea;Pieris rapae;Chilo suppressalis;Lobesia botrana;Diabrotica undecimpunctata howardi;Spodoptera exigua;Bombyx mori;Spodoptera litura;Epiphyas*

								postvittana;Leptinotarsa decemlineata;Earias insulana;Meloidogyne incognita;Colaphellus bowringi;Aedes aegypti;Diaphorina citri;Agrotis ipsilon;Laodelphax striatellus;Blattella germanica
GCF_033487275.1	*Bacillus thuringiensis*	CAPE95	Spp1Aa1	1	Yes	Spp1A	Lepidoptera;Blattodea	*Spodoptera litura;Blattella germanica*
GCF_033071735.1	*Bacillus thuringiensis*	S908	Cry1Ba8,Mpp5Ab1,Vpa2Bb4	3	Yes	cry1B,Mpp5A	Lepidoptera;Hemiptera;Diptera;Coleoptera	*Plutella xylostella;Ostrinia nubilalis;Spodoptera frugiperda;Maruca vitrata;Manduca sexta;cotton boll weevil;Colaphellus bowringi;Leptinotarsa decemlineata*
GCF_033071715.1	*Bacillus thuringiensis*	S1785	Cry11Aa3,Cry10Aa4,Cyt1Ca1,Cyt1Aa11,Cyt2Ba17,Mpp60Ba3,Mpp60Aa3	7	No	cry11A,cry10A,cyt1A,cyt2B,Mpp60B,Mpp60A	Diptera;Coleoptera;Hemiptera;Lepidoptera	*Aedes aegypti;Anopheles albimanus;Culex quinquefasciatu;Culex pipiens;cotton boll weevil;Culex quinquefasciatus;Culex pipien;Diaphorina citri;Plutella xylostella;Anopheles stephensi;Anopheles gambiae;Bradysia difformis*
GCF_031337245.1	*Bacillus thuringiensis*	S1307	Cry1Ba8,Cry1Ia42,Cry2Aa22,Cry2Ab41,Vip3Aa79,Mpp5Ab1,Vpa2Bb4	7	Yes	cry1B,cry1I,cry2A,cry2A,vip3Aa,Mpp5A	Lepidoptera;Hemiptera;Diptera;Coleoptera;Rhabditida;Blattodea;Aedes	*Plutella xylostella;Ostrinia nubilalis;Spodoptera frugiperda;Maruca vitrata;Manduca sexta;cotton boll weevil;Leptinotarsa*

							aegypti	*decemlineata;Ostrinia furnacalis;Lobesia botrana;Earias insulana;Meloidogyne incognita;Helicoverpa armigera;Aedes aegypti;Diaphorina citri;Helicoverpa punctigera;Helicoverpa zea;Chilo partellus;Blattella germanica;Colaphellus bowringi*
GCF_031337235.1	*Bacillus thuringiensis*	S1287	Cry1Ja2,Cry1Hb2,Cry1Id2,Cry1Nb1,Cry1Ka2,Vip3Ag6,Spp1Aa1,Vpa2Aa3,Vpb1Aa2	9	No	cry1J,cry1H,cry1I,cry1N,cry1K,vip3Ag,Spp1A	Lepidoptera;Coleoptera;Rhabditida;Blattodea	*Plutella xylostella;Manduca sexta;Lobesia botrana;Diabrotica undecimpunctata howardi;Spodoptera exigua;Ostrinia furnacalis;Leptinotarsa decemlineata;Earias insulana;cotton boll weevil;Meloidogyne incognita;Colaphellus bowringi;Artogeia rapae;Pieris rapae;Spodoptera littoralis;Chrysodeixis chalcites;Spodoptera litura;Blattella germanica*
GCF_031317075.1	*Bacillus thuringiensis*	NR-28583	Spp1Aa1	1	Yes	Spp1A	Lepidoptera;Blattodea	*Spodoptera litura;Blattella germanica*
GCF_031317055.1	*Bacillus thuringiensis*	NR-610	Cry1Ia42,Cry2Aa22,Cry2Ab15,Vip3Aa79,Spp1Aa1	5	Yes	cry1I,cry2A,cry2A,vip3Aa,Spp1A	Coleoptera;Lepidoptera;Rhabditida;Diptera;Hemiptera	*Leptinotarsa decemlineata;Ostrinia furnacalis;Lobesia botrana;Plutella xylostella;Earias insulana;cotton boll*

							;Blattodea;Aedes aegypti	*weevil;Meloidogyne incognita;Helicoverpa armigera;Aedes aegypti;Diaphorina citri;Helicoverpa punctigera;Spodoptera frugiperda;Helicoverpa zea;Chilo partellus;Blattella germanica;Spodoptera litura*
GCF_031316455.1	*Bacillus thuringiensis*	ATCC 700872	Mpp60Ba3,Mpp60Aa3	2	No	Mpp60B,Mpp60A	Diptera	*Culex quinquefasciatus*
GCF_031316415.1	*Bacillus thuringiensis*	ATCC 13367	Cry1Ba4,Spp1Aa1	2	Yes	cry1B,Spp1A	Lepidoptera;Hemiptera;Diptera;Coleoptera;Blattodea	*Plutella xylostella;Ostrinia nubilalis;Spodoptera frugiperda;Maruca vitrata;Manduca sexta;cotton boll weevil;Spodoptera litura;Blattella germanica*
GCF_031316375.1	*Bacillus thuringiensis*	ATCC 33679	Cry1Ia42,Cry2Aa22,Cry2Ab41,Cry1Ac5,Cry1Ab9,Vip3Aa79,Spp1Aa1	7	Yes	cry1I,cry2A,cry2A,cry1A,cry1A,vip3Aa,Spp1A	Coleoptera;Lepidoptera;Rhabditida;Diptera;Hemiptera;Blattodea;Aedes aegypti	*Leptinotarsa decemlineata;Ostrinia furnacalis;Lobesia botrana;Plutella xylostella;Earias insulana;cotton boll weevil;Meloidogyne incognita;Helicoverpa armigera;Aedes aegypti;Diaphorina citri;Leucinodes orbonalis;Trichoplusia ni;Hyphantria cunea;Spodoptera frugiperda;Pieris rapae;Chilo suppressalis;Helicoverpa punctigera;Helicoverpa zea;Chilo*

								partellus;Blattella germanica;Spodoptera litura
GCF_031316355.1	*Bacillus thuringiensis*	ATCC 35646	Cry11Aa3,Cry10Aa4,Cry4Ba7,Cyt1Ca1,Cyt1Aa11,Cyt2Ba17,Mpp60Ba3,Mpp60Aa3	8	No	cry11A,cry10A,cry4B,cyt1A,cyt2B,Mpp60B,Mpp60A	Diptera;Coleoptera;Hemiptera;Lepidoptera	*Aedes aegypti;Anopheles albimanus;Culex quinquefasciatu;Culex pipiens;cotton boll weevil;Anopheles gambiae;Diaphorina citri;Culex quinquefasciatus;Culex pipien;Plutella xylostella;Anopheles stephensi;Bradysia difformis*
GCF_031171585.1	*Bacillus thuringiensis serovar tenebrionis*	ten BI 256-82 /CM3	Cry3Aa6,Mpp23Aa1,Spp1Aa1,Xpp37Aa1	4	No	Mpp23A+Xpp37A,Cry3A,Spp1A	Coleoptera;Hemiptera;Lepidoptera;Blattodea	*Cylas puncticollis;Anthonomus grandis;Leptinotarsa decemlineata;Tenebrio molitor;Hypera postica;Macrosiphum euphorbiae;Spodoptera litura;Blattella germanica*
GCF_031169915.1	*Bacillus thuringiensis serovar tenebrionis*	BI 256-82	Cry3Aa6,Mpp23Aa1,Spp1Aa1,Xpp37Aa1	4	No	Mpp23A+Xpp37A,Cry3A,Spp1A	Coleoptera;Hemiptera;Lepidoptera;Blattodea	*Cylas puncticollis;Anthonomus grandis;Leptinotarsa decemlineata;Tenebrio molitor;Hypera postica;Macrosiphum euphorbiae;Spodoptera litura;Blattella germanica*
GCF_031157435.1	*Bacillus thuringiensis serovar*	AS23	Cry1Aa3,Cry1Ia42,Cry2Ab41,Cry9Ea12,Cry1Ca18,Cry1Da2,Vip3Aa79,Spp1Aa1	8	Yes	cry1A,cry1I,cry2A,Cry9E,cry1C,cry1D,vip3Aa,Spp1A	Lepidoptera;Coleoptera;Rhabditida;Diptera;Hemiptera;Dipteran;Blattode	*Plutella xylostella;Ostrinia furnacalis;Helicoverpa armigera;Leucinodes orbonalis;Trichoplusia ni;Hyphantria*

	aizawai						a;Aedes aegypti	*cunea;Spodoptera frugiperda;Pieris rapae;Chilo suppressalis;Leptinotarsa decemlineata;Lobesia botrana;Earias insulana;cotton boll weevil;Meloidogyne incognita;Aedes aegypti;Diaphorina citri;Agrotis exclamationis;Cydia pomonella;Myzus persicae;Ostrinia nubilalis;Spodoptera exigua;Bombyx mori;Spodoptera litura;Epiphyas postvittana;Helicoverpa punctigera;Helicoverpa zea;Chilo partellus;Blattella germanica*
GCF_030984695.1	*Bacillus thuringiensis*	S1905	Cry1Ia42,Cry2Aa22,Cry2Ab41,Vip3Aa79,Spp1Aa1	5	Yes	cry1I,cry2A,cry2A,vip3Aa,Spp1A	Coleoptera;Lepidoptera;Rhabditida;Diptera;Hemiptera;Blattodea;Aedes aegypti	*Leptinotarsa decemlineata;Ostrinia furnacalis;Lobesia botrana;Plutella xylostella;Earias insulana;cotton boll weevil;Meloidogyne incognita;Helicoverpa armigera;Aedes aegypti;Diaphorina citri;Helicoverpa punctigera;Spodoptera frugiperda;Helicoverpa zea;Chilo partellus;Blattella germanica;Spodoptera litura*
GCF_030547405.1	*Bacillus thuringiensis*	C3R06_2	Mpp5Aa1,Spp1Aa1	2	No	Mpp5A,Spp1A	Coleoptera;Lepidoptera;Blattodea	*Colaphellus bowringi;Leptinotarsa decemlineata;Spodoptera litura;Blattella germanica*

GCF_030464745.1	*Bacillus thuringiensis*	S906	Cry1Ba8,Mpp5Ab1,Vpa2Bb4	3	Yes	cry1B,Mpp5A	Lepidoptera;Hemiptera;Diptera;Coleoptera	*Plutella xylostella;Ostrinia nubilalis;Spodoptera frugiperda;Maruca vitrata;Manduca sexta;cotton boll weevil;Colaphellus bowringi;Leptinotarsa decemlineata*
GCF_030386775.1	*Bacillus thuringiensis serovar galleriae*	BTG	Cry1Ga1,Cry1Ib3,Cry1Db1,Cry1Fb5,Cry2Ab4,Cry1Ab35,Cry9Ba1,Vip3Aa17,Spp1Aa1,Vpa2Ae3,Vpb1Ac1	11	Yes	Vpa2Ae + Vpb1Ac,cry1G,cry1I,cry1D,cry1F,cry2A,cry1A,Cry9B,vip3Aa,Spp1A	Hemiptera;Lepidoptera;Coleoptera;Rhabditida;Diptera;Blattodea;Aedes aegypti	*Aphis gossypii;Pieris rapae;Thaumetopoea pityocampa;Plutella xylostella;Myzus persicae;Leptinotarsa decemlineata;Ostrinia furnacalis;Lobesia botrana;Earias insulana;cotton boll weevil;Meloidogyne incognita;Ostrinia nubilalis;Spodoptera exigua;Spodoptera frugiperda;Bombyx mori;Spodoptera litura;Epiphyas postvittana;Agrotis ipsilon;Diabrotica virgifera virgifera;Helicoverpa armigera;Aedes aegypti;Diaphorina citri;Leucinodes orbonalis;Trichoplusia ni;Hyphantria cunea;Chilo suppressalis;Manduca sexta;Anticarsia gemmatalis;Helicoverpa punctigera;Helicoverpa zea;Chilo partellus;Blattella germanica*

GCF_030376635.1	*Bacillus thuringiensis*	K2-sn1398	Spp1Aa1	1	No	Spp1A	Lepidoptera;Blattodea	*Spodoptera litura;Blattella germanica*
GCF_028895365.1	*Bacillus thuringiensis*	Bti-185	Cry11Aa3,Cry10Aa4,Cyt1Ca1,Cyt1Aa11,Cyt2Ba17,Mpp60Ba3,Mpp60Aa3	7	No	cry11A,cry10A,cyt1A,cyt2B,Mpp60B,Mpp60A	Diptera;Coleoptera;Hemiptera;Lepidoptera	*Aedes aegypti;Anopheles albimanus;Culex quinquefasciatu;Culex pipiens;cotton boll weevil;Culex quinquefasciatus;Culex pipien;Diaphorina citri;Plutella xylostella;Anopheles stephensi;Anopheles gambiae;Bradysia difformis*
GCF_028765225.1	*Bacillus thuringiensis*	FC8	Cry1Ia42,Cry2Aa22,Cry2Ab41,Vip3Aa79,Spp1Aa1	5	Yes	cry1I,cry2A,cry2A,vip3Aa,Spp1A	Coleoptera;Lepidoptera;Rhabditida;Diptera;Hemiptera;Blattodea;Aedes aegypti	*Leptinotarsa decemlineata;Ostrinia furnacalis;Lobesia botrana;Plutella xylostella;Earias insulana;cotton boll weevil;Meloidogyne incognita;Helicoverpa armigera;Aedes aegypti;Diaphorina citri;Helicoverpa punctigera;Spodoptera frugiperda;Helicoverpa zea;Chilo partellus;Blattella germanica;Spodoptera litura*
GCF_028401745.1	*Bacillus thuringiensis*	45L			No			
GCF_028356755.1	*Bacillus thuringi*	BLB1	Cry1Ia42,Cry2Aa22,Cry2Ab41,Cry1Aa17,Cry	7	Yes	cry1I,cry2A,cry2A,cry1A,cry1A,vip3Aa,Spp	Coleoptera;Lepidoptera;Rhabditida;	*Leptinotarsa decemlineata;Ostrinia furnacalis;Lobesia botrana;Plutella*

	ensis		1Ac5,Vip3Aa79,Spp1Aa1			1A	Diptera;Hemiptera;Blattodea;Aedes aegypti	*xylostella;Earias insulana;cotton boll weevil;Meloidogyne incognita;Helicoverpa armigera;Aedes aegypti;Diaphorina citri;Leucinodes orbonalis;Trichoplusia ni;Hyphantria cunea;Spodoptera frugiperda;Pieris rapae;Chilo suppressalis;Helicoverpa punctigera;Helicoverpa zea;Chilo partellus;Blattella germanica;Spodoptera litura*
GCF_028356555.1	*Bacillus thuringiensis*	Lip	Cry1Ab9,Cry1Aa3,Cry1Ia42,Cry2Aa22,Cry2Ab41,Cry1Ac4,Vip3Aa79,Spp1Aa1	8	Yes	cry1A,cry1A,cry1I,cry2A,cry2A,cry1A,vip3Aa,Spp1A	Lepidoptera;Coleoptera;Rhabditida;Diptera;Hemiptera;Blattodea;Aedes aegypti	*Plutella xylostella;Ostrinia furnacalis;Helicoverpa armigera;Leucinodes orbonalis;Trichoplusia ni;Hyphantria cunea;Spodoptera frugiperda;Pieris rapae;Chilo suppressalis;Leptinotarsa decemlineata;Lobesia botrana;Earias insulana;cotton boll weevil;Meloidogyne incognita;Aedes aegypti;Diaphorina citri;Helicoverpa punctigera;Helicoverpa zea;Chilo partellus;Blattella germanica;Spodoptera litura*
GCF_027627725.1	*Bacillus thuringi*	NB125	Cry3Aa6,Mpp23Aa1,Spp1Aa1,Vpa2Ah1,Vpb1	6	No	Mpp23A+Xpp37A,Vpa2Ah +	Coleoptera;Hemiptera;Lepidoptera;B	*Cylas puncticollis;Anthonomus grandis;Aphis gossypii;Leptinotarsa*

	ensis serovar tenebrionis		Ae1,Xpp37Aa1			Vpb1Ae,Cry3A,Spp1A	lattodea	*decemlineata;Tenebrio molitor;Hypera postica;Macrosiphum euphorbiae;Spodoptera litura;Blattella germanica*
GCF_026620185.1	*Bacillus thuringiensis*	T405	Cry1Ia42,Cry2Aa22,Cry2Ab41,Vip3Aa79,Spp1Aa1	5	Yes	cry1I,cry2A,cry2A,vip3Aa,Spp1A	Coleoptera;Lepidoptera;Rhabditida;Diptera;Hemiptera;Blattodea;Aedes aegypti	*Leptinotarsa decemlineata;Ostrinia furnacalis;Lobesia botrana;Plutella xylostella;Earias insulana;cotton boll weevil;Meloidogyne incognita;Helicoverpa armigera;Aedes aegypti;Diaphorina citri;Helicoverpa punctigera;Spodoptera frugiperda;Helicoverpa zea;Chilo partellus;Blattella germanica;Spodoptera litura*
GCF_025947955.1	*Bacillus thuringiensis*	Bt Gxmzu777-1	Sip1A2-other,Spp1Aa1	2	No	Spp1A	Lepidoptera;Blattodea	*Spodoptera litura;Blattella germanica*
GCF_025677725.1	*Bacillus thuringiensis*	4F5	Cry2Aa22,Cry1Ia42,Cry1Ac2,Spp1Aa1	4	Yes	cry2A,cry1I,cry1A,Spp1A	Lepidoptera;Diptera;Hemiptera;Coleoptera;Rhabditida;Blattodea	*Helicoverpa armigera;Aedes aegypti;Diaphorina citri;Leptinotarsa decemlineata;Ostrinia furnacalis;Lobesia botrana;Plutella xylostella;Earias insulana;cotton boll weevil;Meloidogyne incognita;Leucinodes orbonalis;Trichoplusia ni;Hyphantria*

								cunea;Spodoptera frugiperda;Pieris rapae;Chilo suppressalis;Spodoptera litura;Blattella germanica
GCF_025630665.1	*Bacillus thuringiensis*	SRCM116092	Spp1Aa1	1	Yes	Spp1A	Lepidoptera;Blattodea	*Spodoptera litura;Blattella germanica*
GCF_025630655.1	*Bacillus thuringiensis*	SRCM116511	Spp1Aa1	1	Yes	Spp1A	Lepidoptera;Blattodea	*Spodoptera litura;Blattella germanica*
GCF_025210105.1	*Bacillus thuringiensis*	dipel	Cry1Ia42,Cry2Aa22,Cry2Ab41,Vip3Aa79,Spp1Aa1	5	Yes	cry1I,cry2A,cry2A,vip3Aa,Spp1A	Coleoptera;Lepidoptera;Rhabditida;Diptera;Hemiptera;Blattodea;Aedes aegypti	*Leptinotarsa decemlineata;Ostrinia furnacalis;Lobesia botrana;Plutella xylostella;Earias insulana;cotton boll weevil;Meloidogyne incognita;Helicoverpa armigera;Aedes aegypti;Diaphorina citri;Helicoverpa punctigera;Spodoptera frugiperda;Helicoverpa zea;Chilo partellus;Blattella germanica;Spodoptera litura*
GCF_025210065.1	*Bacillus thuringiensis*	xentari	Cry1Ia42,Cry2Ab41,Cry9Ea12,Cry1Ca18,Cry1Da2,Vip3Aa79,Spp1Aa1	7	Yes	cry1I,cry2A,Cry9E,cry1C,cry1D,vip3Aa,Spp1A	Coleoptera;Lepidoptera;Rhabditida;Diptera;Hemiptera;Dipteran;Blattodea;Aedes aegypti	*Leptinotarsa decemlineata;Ostrinia furnacalis;Lobesia botrana;Plutella xylostella;Earias insulana;cotton boll weevil;Meloidogyne incognita;Helicoverpa armigera;Aedes aegypti;Diaphorina citri;Agrotis exclamationis;Cydia pomonella;Myzus persicae;Ostrinia*

								nubilalis;Spodoptera exigua;Spodoptera frugiperda;Bombyx mori;Spodoptera litura;Epiphyas postvittana;Helicoverpa punctigera;Helicoverpa zea;Chilo partellus;Blattella germanica
GCF_025209995.1	*Bacillus thuringiensis*	delfin	Cry1Ia42,Cry2Aa22,Cry2Ab41,Vip3Aa79,Spp1Aa1	5	Yes	cry1I,cry2A,cry2A,vip3Aa,Spp1A	Coleoptera;Lepidoptera;Rhabditida;Diptera;Hemiptera;Blattodea;Aedes aegypti	*Leptinotarsa decemlineata;Ostrinia furnacalis;Lobesia botrana;Plutella xylostella;Earias insulana;cotton boll weevil;Meloidogyne incognita;Helicoverpa armigera;Aedes aegypti;Diaphorina citri;Helicoverpa punctigera;Spodoptera frugiperda;Helicoverpa zea;Chilo partellus;Blattella germanica;Spodoptera litura*
GCF_024915015.1	*Bacillus thuringiensis*	TOL651	Cry1Ia42,Cry2Aa22,Cry1Ac5,Spp1Aa1	4	Yes	cry1I,cry2A,cry1A,Spp1A	Coleoptera;Lepidoptera;Rhabditida;Diptera;Hemiptera;Blattodea	*Leptinotarsa decemlineata;Ostrinia furnacalis;Lobesia botrana;Plutella xylostella;Earias insulana;cotton boll weevil;Meloidogyne incognita;Helicoverpa armigera;Aedes aegypti;Diaphorina citri;Leucinodes orbonalis;Trichoplusia ni;Hyphantria cunea;Spodoptera frugiperda;Pieris rapae;Chilo suppressalis;Spodoptera*

								litura;Blattella germanica
GCF_024712765.1	*Bacillus thuringiensis*	IBL00090	Cry1Bb1,Cry1Ab24,Cry1Ja2,Cry1Da3,Cry1Hb2,Cry1Id2,Cry1Nb1,Cry2Ad1,Vip3Af3,App4Aa1,Spp1Aa1,Vpa2Ac2,Vpb1Ca1	13	Yes	cry1B,cry1A,cry1J,cry1D,cry1H,cry1I,cry1N,cry2A,vip3Af,App4A,Spp1A	Lepidoptera;Hemiptera;Diptera;Coleoptera;Rhabditida;Blattodea	*Plutella xylostella;Ostrinia nubilalis;Spodoptera frugiperda;Maruca vitrata;Manduca sexta;cotton boll weevil;Ostrinia furnacalis;Helicoverpa armigera;Leucinodes orbonalis;Trichoplusia ni;Hyphantria cunea;Pieris rapae;Chilo suppressalis;Lobesia botrana;Diabrotica undecimpunctata howardi;Spodoptera exigua;Bombyx mori;Spodoptera litura;Epiphyas postvittana;Leptinotarsa decemlineata;Earias insulana;Meloidogyne incognita;Colaphellus bowringi;Aedes aegypti;Diaphorina citri;Agrotis ipsilon;Laodelphax striatellus;Blattella germanica*
GCF_024712745.1	*Bacillus thuringiensis*	IBL00144	Cry1Ma2,Cry2Ba1,Vip3Ba2,Mpp15Aa1,Mpp93Aa1,Spp1Aa1	6	Yes	cry1M,cry2B,vip3B,Mpp15A,Mpp93A,Spp1A	Lepidoptera;Diptera;Pieris rapae;Cydia pomonella;Blattodea	*corn earworm (Helicoverpa zea);black cut worm (Agrotis ipsilon);cabbage looper (Trichoplusia ni);saltmarch caterpillar (Estigmene acrea);tobacco budworm (Heliothis virescens);and lettuce armyworm (Pseudaletia unipuncta).;Manduca*

								sexta;Ostrinia nubilalis;Aedes aegypti;Agrotis ipsilon;Spodoptera frugiperda;Helicoverpa armigera;Spodoptera exigua;Chrysodeixis includens;Spodoptera litura;Blattella germanica
GCF_024712725.1	*Bacillus thuringiensis*	IBL00171	Cry1Bb1,Cry1Ab24,Cry1Ja2,Cry1Da3,Cry1Hb2,Cry1Id2,Cry1Nb1,Cry2Ad1,Vip3Af3,App4Aa1,Spp1Aa1,Vpa2Ac2,Vpb1Ca1	13	Yes	cry1B,cry1A,cry1J,cry1D,cry1H,cry1I,cry1N,cry2A,vip3Af,App4A,Spp1A	Lepidoptera;Hemiptera;Diptera;Coleoptera;Rhabditida;Blattodea	*Plutella xylostella;Ostrinia nubilalis;Spodoptera frugiperda;Maruca vitrata;Manduca sexta;cotton boll weevil;Ostrinia furnacalis;Helicoverpa armigera;Leucinodes orbonalis;Trichoplusia ni;Hyphantria cunea;Pieris rapae;Chilo suppressalis;Lobesia botrana;Diabrotica undecimpunctata howardi;Spodoptera exigua;Bombyx mori;Spodoptera litura;Epiphyas postvittana;Leptinotarsa decemlineata;Earias insulana;Meloidogyne incognita;Colaphellus bowringi;Aedes aegypti;Diaphorina citri;Agrotis ipsilon;Laodelphax striatellus;Blattella germanica*
GCF_024712705.1	*Bacillus thuringi*	IBL00197	Cry1Ba8,Cry1Aa3,Cry1Ia42,Cry2Ab41,Cry2Aa	7	Yes	cry1B,cry1A,cry1I,cry2A,cry2A,vip3Aa,Spp	Lepidoptera;Hemiptera;Diptera;Cole	*Plutella xylostella;Ostrinia nubilalis;Spodoptera*

	ensis		22,Vip3Aa79,Spp1Aa1			1A	optera;Rhabditida;Blattodea;Aedes aegypti	*frugiperda;Maruca vitrata;Manduca sexta;cotton boll weevil;Ostrinia furnacalis;Helicoverpa armigera;Leucinodes orbonalis;Trichoplusia ni;Hyphantria cunea;Pieris rapae;Chilo suppressalis;Leptinotarsa decemlineata;Lobesia botrana;Earias insulana;Meloidogyne incognita;Aedes aegypti;Diaphorina citri;Helicoverpa punctigera;Helicoverpa zea;Chilo partellus;Blattella germanica;Spodoptera litura*
GCF_024712685.1	*Bacillus thuringiensis*	IBL00210	Cry1Ma2,Cry2Ba1,Vip3Ba2,Mpp15Aa1,Mpp93Aa1,Spp1Aa1	6	Yes	cry1M,cry2B,vip3B,Mpp15A,Mpp93A,Spp1A	Lepidoptera;Diptera;Pieris rapae;Cydia pomonella;Blattodea	*corn earworm (Helicoverpa zea);black cut worm (Agrotis ipsilon);cabbage looper (Trichoplusia ni);saltmarch caterpillar (Estigmene acrea);tobacco budworm (Heliothis virescens);and lettuce armyworm (Pseudaletia unipuncta).;Manduca sexta;Ostrinia nubilalis;Aedes aegypti;Agrotis ipsilon;Spodoptera frugiperda;Helicoverpa armigera;Spodoptera exigua;Chrysodeixis includens;Spodoptera litura;Blattella*

								germanica
GCF_024712665.1	*Bacillus thuringiensis*	IBL00503	Cry1Ia42,Cry2Aa22,Cry2Ab41,Cry1Aa17,Cry1Ac5,Vip3Aa79,Spp1Aa1	7	Yes	cry1I,cry2A,cry2A,cry1A,cry1A,vip3Aa,Spp1A	Coleoptera;Lepidoptera;Rhabditida;Diptera;Hemiptera;Blattodea;Aedes aegypti	*Leptinotarsa decemlineata;Ostrinia furnacalis;Lobesia botrana;Plutella xylostella;Earias insulana;cotton boll weevil;Meloidogyne incognita;Helicoverpa armigera;Aedes aegypti;Diaphorina citri;Leucinodes orbonalis;Trichoplusia ni;Hyphantria cunea;Spodoptera frugiperda;Pieris rapae;Chilo suppressalis;Helicoverpa punctigera;Helicoverpa zea;Chilo partellus;Blattella germanica;Spodoptera litura*
GCF_024712595.1	*Bacillus thuringiensis*	IBL01313	Cry1Ia42,Cry2Aa22,Cry2Ab41,Cry1Aa17,Cry1Ac5,Vip3Aa79,Spp1Aa1	7	Yes	cry1I,cry2A,cry2A,cry1A,cry1A,vip3Aa,Spp1A	Coleoptera;Lepidoptera;Rhabditida;Diptera;Hemiptera;Blattodea;Aedes aegypti	*Leptinotarsa decemlineata;Ostrinia furnacalis;Lobesia botrana;Plutella xylostella;Earias insulana;cotton boll weevil;Meloidogyne incognita;Helicoverpa armigera;Aedes aegypti;Diaphorina citri;Leucinodes orbonalis;Trichoplusia ni;Hyphantria cunea;Spodoptera frugiperda;Pieris rapae;Chilo suppressalis;Helicoverpa punctigera;Helicoverpa zea;Chilo partellus;Blattella*

								germanica;Spodoptera litura
GCF_024712585.1	*Bacillus thuringiensis*	IBL01259	Cry1Ia42,Cry2Aa22,Cry2Ab41,Cry1Aa17,Cry1Ac5,Vip3Aa79,Spp1Aa1	7	Yes	cry1I,cry2A,cry2A,cry1A,cry1A,vip3Aa,Spp1A	Coleoptera;Lepidoptera;Rhabditida;Diptera;Hemiptera;Blattodea;Aedes aegypti	*Leptinotarsa decemlineata;Ostrinia furnacalis;Lobesia botrana;Plutella xylostella;Earias insulana;cotton boll weevil;Meloidogyne incognita;Helicoverpa armigera;Aedes aegypti;Diaphorina citri;Leucinodes orbonalis;Trichoplusia ni;Hyphantria cunea;Spodoptera frugiperda;Pieris rapae;Chilo suppressalis;Helicoverpa punctigera;Helicoverpa zea;Chilo partellus;Blattella germanica;Spodoptera litura*
GCF_024712565.1	*Bacillus thuringiensis*	IBL01677	Cry1Ab9,Cry1Ba4,Spp1Aa1	3	Yes	cry1A,cry1B,Spp1A	Lepidoptera;Hemiptera;Diptera;Coleoptera;Blattodea	*Plutella xylostella;Ostrinia furnacalis;Helicoverpa armigera;Leucinodes orbonalis;Trichoplusia ni;Hyphantria cunea;Spodoptera frugiperda;Pieris rapae;Chilo suppressalis;Ostrinia nubilalis;Maruca vitrata;Manduca sexta;cotton boll weevil;Spodoptera litura;Blattella germanica*
GCF_024712535.1	*Bacillus thuringiensis*	IBL02897	Cry1Bb1,Cry1Ab24,Cry1Ja2,Cry1Da3,Cry1Hb2,Cry1Id2,Cry1Nb1,Cry2Ad1,Vip3Af3,App4Aa	13	Yes	cry1B,cry1A,cry1J,cry1D,cry1H,cry1I,cry1N,cry2A,vip3Af,App4A,Spp1A	Lepidoptera;Hemiptera;Diptera;Coleoptera;Rhabditida;Blattodea	*Plutella xylostella;Ostrinia nubilalis;Spodoptera frugiperda;Maruca vitrata;Manduca sexta;cotton boll weevil;Ostrinia*

			1,Spp1Aa1,Vpa2Ac2,Vpb1Ca1					*furnacalis;Helicoverpa armigera;Leucinodes orbonalis;Trichoplusia ni;Hyphantria cunea;Pieris rapae;Chilo suppressalis;Lobesia botrana;Diabrotica undecimpunctata howardi;Spodoptera exigua;Bombyx mori;Spodoptera litura;Epiphyas postvittana;Leptinotarsa decemlineata;Earias insulana;Meloidogyne incognita;Colaphellus bowringi;Aedes aegypti;Diaphorina citri;Agrotis ipsilon;Laodelphax striatellus;Blattella germanica*
GCF_024712525.2	*Bacillus thuringiensis*	IBL03111	Cry1Ia42,Cry2Aa22,Cry2Ab41,Cry1Aa17,Cry1Ac5,Vip3Aa79,Spp1Aa1	7	Yes	cry1I,cry2A,cry2A,cry1A,cry1A,vip3Aa,Spp1A	Coleoptera;Lepidoptera;Rhabditida;Diptera;Hemiptera;Blattodea;Aedes aegypti	*Leptinotarsa decemlineata;Ostrinia furnacalis;Lobesia botrana;Plutella xylostella;Earias insulana;cotton boll weevil;Meloidogyne incognita;Helicoverpa armigera;Aedes aegypti;Diaphorina citri;Leucinodes orbonalis;Trichoplusia ni;Hyphantria cunea;Spodoptera frugiperda;Pieris rapae;Chilo suppressalis;Helicoverpa punctigera;Helicoverpa zea;Chilo partellus;Blattella*

								germanica;Spodoptera litura
GCF_023502835.1	*Bacillus thuringiensis*	D3A2_S7	Spp1Aa1	1	No	Spp1A	Lepidoptera;Blattodea	*Spodoptera litura;Blattella germanica*
GCF_022809735.1	*Bacillus thuringiensis*	UFT038	Cry1Ia42,Cry2Aa22,Cry2Ab41,Cry1Aa45,Cry1Ac11,Vip3Aa79,Spp1Aa1	7	Yes	cry1I,cry2A,cry2A,cry1A,cry1A,vip3Aa,Spp1A	Coleoptera;Lepidoptera;Rhabditida;Diptera;Hemiptera;Blattodea;Aedes aegypti	*Leptinotarsa decemlineata;Ostrinia furnacalis;Lobesia botrana;Plutella xylostella;Earias insulana;cotton boll weevil;Meloidogyne incognita;Helicoverpa armigera;Aedes aegypti;Diaphorina citri;Leucinodes orbonalis;Trichoplusia ni;Hyphantria cunea;Spodoptera frugiperda;Pieris rapae;Chilo suppressalis;Helicoverpa punctigera;Helicoverpa zea;Chilo partellus;Blattella germanica;Spodoptera litura*
GCF_022095615.2	*Bacillus thuringiensis*	HSY204	Cry1Ac9,Cry40Da1,Spp1Aa1	3	No	cry1A,cry40D,Spp1A	Lepidoptera;Diptera;Blattodea	*Plutella xylostella;Ostrinia furnacalis;Helicoverpa armigera;Leucinodes orbonalis;Trichoplusia ni;Hyphantria cunea;Spodoptera frugiperda;Pieris rapae;Chilo suppressalis;Culex quinquefasciatus;Bradysia odoriphaga;Spodoptera litura;Blattella germanica*
GCF_021	*Bacillus*	x023	Cry1Ia42,Cry2Aa22,Cr	6	Yes	cry1I,cry2A,cry2A,cry	Coleoptera;Lepido	*Leptinotarsa decemlineata;Ostrinia*

651035.1	*thuringiensis*		y2Ab41,Cry1Aa17,Vip3Aa79,Spp1Aa1			1A,vip3Aa,Spp1A	ptera;Rhabditida;Diptera;Hemiptera;Blattodea;Aedes aegypti	*furnacalis;Lobesia botrana;Plutella xylostella;Earias insulana;cotton boll weevil;Meloidogyne incognita;Helicoverpa armigera;Aedes aegypti;Diaphorina citri;Leucinodes orbonalis;Trichoplusia ni;Hyphantria cunea;Spodoptera frugiperda;Pieris rapae;Chilo suppressalis;Helicoverpa punctigera;Helicoverpa zea;Chilo partellus;Blattella germanica;Spodoptera litura*
GCF_021595545.1	*Bacillus thuringiensis*	B13	Cry1Ba4,Spp1Aa1	2	Yes	cry1B,Spp1A	Lepidoptera;Hemiptera;Diptera;Coleoptera;Blattodea	*Plutella xylostella;Ostrinia nubilalis;Spodoptera frugiperda;Maruca vitrata;Manduca sexta;cotton boll weevil;Spodoptera litura;Blattella germanica*
GCF_021412465.1	*Bacillus thuringiensis*	X023PN	Cry1Ia42,Cry2Aa22,Cry2Ab41,Vip3Aa79,Spp1Aa1	5	Yes	cry1I,cry2A,cry2A,vip3Aa,Spp1A	Coleoptera;Lepidoptera;Rhabditida;Diptera;Hemiptera;Blattodea;Aedes aegypti	*Leptinotarsa decemlineata;Ostrinia furnacalis;Lobesia botrana;Plutella xylostella;Earias insulana;cotton boll weevil;Meloidogyne incognita;Helicoverpa armigera;Aedes aegypti;Diaphorina citri;Helicoverpa punctigera;Spodoptera frugiperda;Helicoverpa zea;Chilo partellus;Blattella*

								germanica;Spodoptera litura
GCF_020809245.1	*Bacillus thuringiensis*	ABTS-1857	Cry1Ab9,Cry1Aa3,Cry1Ia42,Cry2Ab41,Cry9Ea12,Cry1Ca18,Cry1Da2,Vip3Aa79,Spp1Aa1	9	Yes	cry1A,cry1A,cry1I,cry2A,Cry9E,cry1C,cry1D,vip3Aa,Spp1A	Lepidoptera;Coleoptera;Rhabditida;Diptera;Hemiptera;Dipteran;Blattodea;Aedes aegypti	*Plutella xylostella;Ostrinia furnacalis;Helicoverpa armigera;Leucinodes orbonalis;Trichoplusia ni;Hyphantria cunea;Spodoptera frugiperda;Pieris rapae;Chilo suppressalis;Leptinotarsa decemlineata;Lobesia botrana;Earias insulana;cotton boll weevil;Meloidogyne incognita;Aedes aegypti;Diaphorina citri;Agrotis exclamationis;Cydia pomonella;Myzus persicae;Ostrinia nubilalis;Spodoptera exigua;Bombyx mori;Spodoptera litura;Epiphyas postvittana;Helicoverpa punctigera;Helicoverpa zea;Chilo partellus;Blattella germanica*
GCF_020809205.1	*Bacillus thuringiensis*	GC-91	Cry1Ac9,Cry1Ia42,Cry2Ab41,Cry9Ea12,Cry1Ca18,Cry1Da2,Cry1Aa3,Vip3Aa79,Spp1Aa1	9	Yes	cry1A,cry1I,cry2A,Cry9E,cry1C,cry1D,cry1A,vip3Aa,Spp1A	Lepidoptera;Coleoptera;Rhabditida;Diptera;Hemiptera;Dipteran;Blattodea;Aedes aegypti	*Plutella xylostella;Ostrinia furnacalis;Helicoverpa armigera;Leucinodes orbonalis;Trichoplusia ni;Hyphantria cunea;Spodoptera frugiperda;Pieris rapae;Chilo suppressalis;Leptinotarsa decemlineata;Lobesia botrana;Earias insulana;cotton boll*

								weevil;Meloidogyne incognita;Aedes aegypti;Diaphorina citri;Agrotis exclamationis;Cydia pomonella;Myzus persicae;Ostrinia nubilalis;Spodoptera exigua;Bombyx mori;Spodoptera litura;Epiphyas postvittana;Helicoverpa punctigera;Helicoverpa zea;Chilo partellus;Blattella germanica
GCF_020809185.1	*Bacillus thuringiensis*	BMP144	Cry11Aa3,Cry10Aa4,Cry4Ba7,Cry4Aa5,Cyt1Ca1,Cyt1Aa11,Cyt2Ba17,Mpp60Ba3,Mpp60Aa3	9	No	cry11A,cry10A,cry4B,cry4A,cyt1A,cyt2B,Mpp60B,Mpp60A	Diptera;Coleoptera;Hemiptera;Lepidoptera	*Aedes aegypti;Anopheles albimanus;Culex quinquefasciatu;Culex pipiens;cotton boll weevil;Anopheles gambiae;Diaphorina citri;Culex quinquefasciatus;Acyrthosiphon pisum;Culex pipien;Plutella xylostella;Anopheles stephensi;Bradysia difformis*
GCF_020809145.1	*Bacillus thuringiensis*	SA11	Cry1Ia42,Cry2Aa22,Cry2Ab41,Cry1Aa17,Cry1Ab9,Vip3Aa79,Spp1Aa1	7	Yes	cry1I,cry2A,cry2A,cry1A,cry1A,vip3Aa,Spp1A	Coleoptera;Lepidoptera;Rhabditida;Diptera;Hemiptera;Blattodea;Aedes aegypti	*Leptinotarsa decemlineata;Ostrinia furnacalis;Lobesia botrana;Plutella xylostella;Earias insulana;cotton boll weevil;Meloidogyne incognita;Helicoverpa armigera;Aedes aegypti;Diaphorina citri;Leucinodes orbonalis;Trichoplusia ni;Hyphantria cunea;Spodoptera frugiperda;Pieris rapae;Chilo*

								suppressalis;Helicoverpa punctigera;Helicoverpa zea;Chilo partellus;Blattella germanica;Spodoptera litura
GCF_020809125.1	*Bacillus thuringiensis*	B401	Cry1Ab9,Cry1Ia42,Cry2Ab41,Cry9Ea12,Cry1Ca18,Cry1Da2,Cry1Aa3,Vip3Aa79,Spp1Aa1	9	Yes	cry1A,cry1I,cry2A,Cry9E,cry1C,cry1D,cry1A,vip3Aa,Spp1A	Lepidoptera;Coleoptera;Rhabditida;Diptera;Hemiptera;Dipteran;Blattodea;Aedes aegypti	*Plutella xylostella;Ostrinia furnacalis;Helicoverpa armigera;Leucinodes orbonalis;Trichoplusia ni;Hyphantria cunea;Spodoptera frugiperda;Pieris rapae;Chilo suppressalis;Leptinotarsa decemlineata;Lobesia botrana;Earias insulana;cotton boll weevil;Meloidogyne incognita;Aedes aegypti;Diaphorina citri;Agrotis exclamationis;Cydia pomonella;Myzus persicae;Ostrinia nubilalis;Spodoptera exigua;Bombyx mori;Spodoptera litura;Epiphyas postvittana;Helicoverpa punctigera;Helicoverpa zea;Chilo partellus;Blattella germanica*
GCF_020809105.1	*Bacillus thuringiensis*	ABTS-351	Cry1Ia42,Cry2Aa22,Cry2Ab41,Cry1Aa17,Cry1Ac5,Cry1Ab9,Vip3Aa79,Spp1Aa1	8	Yes	cry1I,cry2A,cry2A,cry1A,cry1A,cry1A,vip3Aa,Spp1A	Coleoptera;Lepidoptera;Rhabditida;Diptera;Hemiptera;Blattodea;Aedes aegypti	*Leptinotarsa decemlineata;Ostrinia furnacalis;Lobesia botrana;Plutella xylostella;Earias insulana;cotton boll weevil;Meloidogyne incognita;Helicoverpa armigera;Aedes aegypti;Diaphorina*

								citri;Leucinodes orbonalis;Trichoplusia ni;Hyphantria cunea;Spodoptera frugiperda;Pieris rapae;Chilo suppressalis;Helicoverpa punctigera;Helicoverpa zea;Chilo partellus;Blattella germanica;Spodoptera litura
GCF_020775995.1	*Bacillus thuringiensis*	CH_684	Cry1Ia42,Cry2Aa22,Cry2Ab41,Vip3Aa79,Spp1Aa1	5	Yes	cry1I,cry2A,cry2A,vip3Aa,Spp1A	Coleoptera;Lepidoptera;Rhabditida;Diptera;Hemiptera;Blattodea;Aedes aegypti	*Leptinotarsa decemlineata;Ostrinia furnacalis;Lobesia botrana;Plutella xylostella;Earias insulana;cotton boll weevil;Meloidogyne incognita;Helicoverpa armigera;Aedes aegypti;Diaphorina citri;Helicoverpa punctigera;Spodoptera frugiperda;Helicoverpa zea;Chilo partellus;Blattella germanica;Spodoptera litura*
GCF_020775985.1	*Bacillus thuringiensis*	CH_692	Cry1Ia42,Cry2Ab41,Cry9Ea12,Cry1Ca18,Cry1Da2,Vip3Aa79,Spp1Aa1	7	Yes	cry1I,cry2A,Cry9E,cry1C,cry1D,vip3Aa,Spp1A	Coleoptera;Lepidoptera;Rhabditida;Diptera;Hemiptera;Dipteran;Blattodea;Aedes aegypti	*Leptinotarsa decemlineata;Ostrinia furnacalis;Lobesia botrana;Plutella xylostella;Earias insulana;cotton boll weevil;Meloidogyne incognita;Helicoverpa armigera;Aedes aegypti;Diaphorina citri;Agrotis exclamationis;Cydia pomonella;Myzus persicae;Ostrinia nubilalis;Spodoptera*

								exigua;Spodoptera frugiperda;Bombyx mori;Spodoptera litura;Epiphyas postvittana;Helicoverpa punctigera;Helicoverpa zea;Chilo partellus;Blattella germanica
GCF_020775975.1	*Bacillus thuringiensis*	CH_698	Cry1Ia42,Cry2Aa22,Cry2Ab41,Cry1Ab9,Vip3Aa79,Spp1Aa1	6	Yes	cry1I,cry2A,cry2A,cry1A,vip3Aa,Spp1A	Coleoptera;Lepidoptera;Rhabditida;Diptera;Hemiptera;Blattodea;Aedes aegypti	*Leptinotarsa decemlineata;Ostrinia furnacalis;Lobesia botrana;Plutella xylostella;Earias insulana;cotton boll weevil;Meloidogyne incognita;Helicoverpa armigera;Aedes aegypti;Diaphorina citri;Leucinodes orbonalis;Trichoplusia ni;Hyphantria cunea;Spodoptera frugiperda;Pieris rapae;Chilo suppressalis;Helicoverpa punctigera;Helicoverpa zea;Chilo partellus;Blattella germanica;Spodoptera litura*
GCF_020775925.1	*Bacillus thuringiensis*	CH_733	Cry1Ia42,Cry2Ab41,Cry9Ea12,Cry1Ca18,Cry1Da2,Vip3Aa79,Spp1Aa1	7	Yes	cry1I,cry2A,Cry9E,cry1C,cry1D,vip3Aa,Spp1A	Coleoptera;Lepidoptera;Rhabditida;Diptera;Hemiptera;Dipteran;Blattodea;Aedes aegypti	*Leptinotarsa decemlineata;Ostrinia furnacalis;Lobesia botrana;Plutella xylostella;Earias insulana;cotton boll weevil;Meloidogyne incognita;Helicoverpa armigera;Aedes aegypti;Diaphorina citri;Agrotis exclamationis;Cydia pomonella;Myzus persicae;Ostrinia*

								nubilalis;Spodoptera exigua;Spodoptera frugiperda;Bombyx mori;Spodoptera litura;Epiphyas postvittana;Helicoverpa punctigera;Helicoverpa zea;Chilo partellus;Blattella germanica
GCF_020775915.1	*Bacillus thuringiensis*	CH_696	Cry1Ia42,Cry2Ab41,Cry9Ea12,Cry1Ca18,Cry1Da2,Vip3Aa79,Spp1Aa1	7	Yes	cry1I,cry2A,Cry9E,cry1C,cry1D,vip3Aa,Spp1A	Coleoptera;Lepidoptera;Rhabditida;Diptera;Hemiptera;Dipteran;Blattodea;Aedes aegypti	*Leptinotarsa decemlineata;Ostrinia furnacalis;Lobesia botrana;Plutella xylostella;Earias insulana;cotton boll weevil;Meloidogyne incognita;Helicoverpa armigera;Aedes aegypti;Diaphorina citri;Agrotis exclamationis;Cydia pomonella;Myzus persicae;Ostrinia nubilalis;Spodoptera exigua;Spodoptera frugiperda;Bombyx mori;Spodoptera litura;Epiphyas postvittana;Helicoverpa punctigera;Helicoverpa zea;Chilo partellus;Blattella germanica*
GCF_020775875.1	*Bacillus thuringiensis*	P01_1	Cry1Ia42,Cry2Ab41,Cry9Ea12,Cry1Ca18,Cry1Da2,Cry1Aa3,Cry1Ab9,Vip3Aa79,Spp1Aa1	9	Yes	cry1I,cry2A,Cry9E,cry1C,cry1D,cry1A,cry1A,vip3Aa,Spp1A	Coleoptera;Lepidoptera;Rhabditida;Diptera;Hemiptera;Dipteran;Blattodea;Aedes aegypti	*Leptinotarsa decemlineata;Ostrinia furnacalis;Lobesia botrana;Plutella xylostella;Earias insulana;cotton boll weevil;Meloidogyne incognita;Helicoverpa armigera;Aedes aegypti;Diaphorina*

								citri;Agrotis exclamationis;Cydia pomonella;Myzus persicae;Ostrinia nubilalis;Spodoptera exigua;Spodoptera frugiperda;Bombyx mori;Spodoptera litura;Epiphyas postvittana;Leucinodes orbonalis;Trichoplusia ni;Hyphantria cunea;Pieris rapae;Chilo suppressalis;Helicoverpa punctigera;Helicoverpa zea;Chilo partellus;Blattella germanica
GCF_020775865.1	*Bacillus thuringiensis*	CH_722	Cry1Ia42,Cry2Aa22,Cry2Ab41,Cry1Ab9,Vip3Aa79,Spp1Aa1	6	Yes	cry1I,cry2A,cry2A,cry1A,vip3Aa,Spp1A	Coleoptera;Lepidoptera;Rhabditida;Diptera;Hemiptera;Blattodea;Aedes aegypti	*Leptinotarsa decemlineata;Ostrinia furnacalis;Lobesia botrana;Plutella xylostella;Earias insulana;cotton boll weevil;Meloidogyne incognita;Helicoverpa armigera;Aedes aegypti;Diaphorina citri;Leucinodes orbonalis;Trichoplusia ni;Hyphantria cunea;Spodoptera frugiperda;Pieris rapae;Chilo suppressalis;Helicoverpa punctigera;Helicoverpa zea;Chilo partellus;Blattella germanica;Spodoptera litura*
GCF_020775855.1	*Bacillus thuringi*	P01_3	Cry1Ia42,Cry2Ab41,Cry9Ea12,Cry1Ca18,Cry1	9	Yes	cry1I,cry2A,Cry9E,cry1C,cry1D,cry1A,cry	Coleoptera;Lepidoptera;Rhabditida;	*Leptinotarsa decemlineata;Ostrinia furnacalis;Lobesia botrana;Plutella*

	ensis		Da2,Cry1Aa3,Cry1Ab9,Vip3Aa79,Spp1Aa1			1A,vip3Aa,Spp1A	Diptera;Hemiptera;Dipteran;Blattodea;Aedes aegypti	*xylostella;Earias insulana;cotton boll weevil;Meloidogyne incognita;Helicoverpa armigera;Aedes aegypti;Diaphorina citri;Agrotis exclamationis;Cydia pomonella;Myzus persicae;Ostrinia nubilalis;Spodoptera exigua;Spodoptera frugiperda;Bombyx mori;Spodoptera litura;Epiphyas postvittana;Leucinodes orbonalis;Trichoplusia ni;Hyphantria cunea;Pieris rapae;Chilo suppressalis;Helicoverpa punctigera;Helicoverpa zea;Chilo partellus;Blattella germanica*
GCF_020775795.1	*Bacillus thuringiensis*	CVUAS2492	Cry1Ia42,Cry2Ab41,Cry9Ea12,Cry1Ca18,Cry1Da2,Cry1Ab9,Vip3Aa79,Spp1Aa1	8	Yes	cry1I,cry2A,Cry9E,cry1C,cry1D,cry1A,vip3Aa,Spp1A	Coleoptera;Lepidoptera;Rhabditida;Diptera;Hemiptera;Dipteran;Blattodea;Aedes aegypti	*Leptinotarsa decemlineata;Ostrinia furnacalis;Lobesia botrana;Plutella xylostella;Earias insulana;cotton boll weevil;Meloidogyne incognita;Helicoverpa armigera;Aedes aegypti;Diaphorina citri;Agrotis exclamationis;Cydia pomonella;Myzus persicae;Ostrinia nubilalis;Spodoptera exigua;Spodoptera frugiperda;Bombyx mori;Spodoptera litura;Epiphyas*

								postvittana;Leucinodes orbonalis;Trichoplusia ni;Hyphantria cunea;Pieris rapae;Chilo suppressalis;Helicoverpa punctigera;Helicoverpa zea;Chilo partellus;Blattella germanica
GCF_020775725.1	*Bacillus thuringiensis*	CH_69	Cry1Ia42,Cry2Aa22,Cry2Ab41,Cry1Ab9,Vip3Aa79,Spp1Aa1	6	Yes	cry1I,cry2A,cry2A,cry1A,vip3Aa,Spp1A	Coleoptera;Lepidoptera;Rhabditida;Diptera;Hemiptera;Blattodea;Aedes aegypti	*Leptinotarsa decemlineata;Ostrinia furnacalis;Lobesia botrana;Plutella xylostella;Earias insulana;cotton boll weevil;Meloidogyne incognita;Helicoverpa armigera;Aedes aegypti;Diaphorina citri;Leucinodes orbonalis;Trichoplusia ni;Hyphantria cunea;Spodoptera frugiperda;Pieris rapae;Chilo suppressalis;Helicoverpa punctigera;Helicoverpa zea;Chilo partellus;Blattella germanica;Spodoptera litura*
GCF_020775715.1	*Bacillus thuringiensis*	CH_81	Cry1Ia42,Cry2Aa22,Cry2Ab41,Cry1Ab9,Vip3Aa79,Spp1Aa1	6	Yes	cry1I,cry2A,cry2A,cry1A,vip3Aa,Spp1A	Coleoptera;Lepidoptera;Rhabditida;Diptera;Hemiptera;Blattodea;Aedes aegypti	*Leptinotarsa decemlineata;Ostrinia furnacalis;Lobesia botrana;Plutella xylostella;Earias insulana;cotton boll weevil;Meloidogyne incognita;Helicoverpa armigera;Aedes aegypti;Diaphorina citri;Leucinodes orbonalis;Trichoplusia ni;Hyphantria*

								cunea;Spodoptera frugiperda;Pieris rapae;Chilo suppressalis;Helicoverpa punctigera;Helicoverpa zea;Chilo partellus;Blattella germanica;Spodoptera litura
GCF_020775705.1	*Bacillus thuringiensis*	CVUAS9660	Cry1Ia42,Cry2Ab41,Cry9Ea12,Cry1Ca18,Cry1Da2,Cry1Aa3,Cry1Ab9,Vip3Aa79,Spp1Aa1	9	Yes	cry1I,cry2A,Cry9E,cry1C,cry1D,cry1A,cry1A,vip3Aa,Spp1A	Coleoptera;Lepidoptera;Rhabditida;Diptera;Hemiptera;Dipteran;Blattodea;Aedes aegypti	*Leptinotarsa decemlineata;Ostrinia furnacalis;Lobesia botrana;Plutella xylostella;Earias insulana;cotton boll weevil;Meloidogyne incognita;Helicoverpa armigera;Aedes aegypti;Diaphorina citri;Agrotis exclamationis;Cydia pomonella;Myzus persicae;Ostrinia nubilalis;Spodoptera exigua;Spodoptera frugiperda;Bombyx mori;Spodoptera litura;Epiphyas postvittana;Leucinodes orbonalis;Trichoplusia ni;Hyphantria cunea;Pieris rapae;Chilo suppressalis;Helicoverpa punctigera;Helicoverpa zea;Chilo partellus;Blattella germanica*
GCF_020775695.1	*Bacillus thuringiensis*	CVUAS9659	Cry1Ia42,Cry2Ab41,Cry9Ea12,Cry1Ca18,Cry1Da2,Cry1Aa3,Cry1Ab9,Vip3Aa79,Spp1Aa1	9	Yes	cry1I,cry2A,Cry9E,cry1C,cry1D,cry1A,cry1A,vip3Aa,Spp1A	Coleoptera;Lepidoptera;Rhabditida;Diptera;Hemiptera;Dipteran;Blattode	*Leptinotarsa decemlineata;Ostrinia furnacalis;Lobesia botrana;Plutella xylostella;Earias insulana;cotton boll weevil;Meloidogyne*

							a;Aedes aegypti	*incognita;Helicoverpa armigera;Aedes aegypti;Diaphorina citri;Agrotis exclamationis;Cydia pomonella;Myzus persicae;Ostrinia nubilalis;Spodoptera exigua;Spodoptera frugiperda;Bombyx mori;Spodoptera litura;Epiphyas postvittana;Leucinodes orbonalis;Trichoplusia ni;Hyphantria cunea;Pieris rapae;Chilo suppressalis;Helicoverpa punctigera;Helicoverpa zea;Chilo partellus;Blattella germanica*
GCF_020775595.1	*Bacillus thuringiensis*	CH_709	Cry1Ia42,Cry2Ab41,Cry9Ea12,Cry1Ca18,Cry1Da2,Cry1Aa3,Cry1Ab9,Vip3Aa79,Spp1Aa1	9	Yes	cry1I,cry2A,Cry9E,cry1C,cry1D,cry1A,cry1A,vip3Aa,Spp1A	Coleoptera;Lepidoptera;Rhabditida;Diptera;Hemiptera;Dipteran;Blattodea;Aedes aegypti	*Leptinotarsa decemlineata;Ostrinia furnacalis;Lobesia botrana;Plutella xylostella;Earias insulana;cotton boll weevil;Meloidogyne incognita;Helicoverpa armigera;Aedes aegypti;Diaphorina citri;Agrotis exclamationis;Cydia pomonella;Myzus persicae;Ostrinia nubilalis;Spodoptera exigua;Spodoptera frugiperda;Bombyx mori;Spodoptera litura;Epiphyas postvittana;Leucinodes orbonalis;Trichoplusia ni;Hyphantria*

								cunea;Pieris rapae;Chilo suppressalis;Helicoverpa punctigera;Helicoverpa zea;Chilo partellus;Blattella germanica
GCF_020775565.1	*Bacillus thuringiensis*	CH_746	Cry1Ia42,Cry2Ab41,Cry9Ea12,Cry1Ca18,Cry1Da2,Cry1Aa3,Cry1Ab9,Vip3Aa79,Spp1Aa1	9	Yes	cry1I,cry2A,Cry9E,cry1C,cry1D,cry1A,cry1A,vip3Aa,Spp1A	Coleoptera;Lepidoptera;Rhabditida;Diptera;Hemiptera;Dipteran;Blattodea;Aedes aegypti	*Leptinotarsa decemlineata;Ostrinia furnacalis;Lobesia botrana;Plutella xylostella;Earias insulana;cotton boll weevil;Meloidogyne incognita;Helicoverpa armigera;Aedes aegypti;Diaphorina citri;Agrotis exclamationis;Cydia pomonella;Myzus persicae;Ostrinia nubilalis;Spodoptera exigua;Spodoptera frugiperda;Bombyx mori;Spodoptera litura;Epiphyas postvittana;Leucinodes orbonalis;Trichoplusia ni;Hyphantria cunea;Pieris rapae;Chilo suppressalis;Helicoverpa punctigera;Helicoverpa zea;Chilo partellus;Blattella germanica*
GCF_020775555.1	*Bacillus thuringiensis*	CH_748	Cry1Ia42,Cry2Ab41,Cry9Ea12,Cry1Ca18,Cry1Da2,Cry1Aa3,Vip3Aa79,Spp1Aa1	8	Yes	cry1I,cry2A,Cry9E,cry1C,cry1D,cry1A,vip3Aa,Spp1A	Coleoptera;Lepidoptera;Rhabditida;Diptera;Hemiptera;Dipteran;Blattodea;Aedes aegypti	*Leptinotarsa decemlineata;Ostrinia furnacalis;Lobesia botrana;Plutella xylostella;Earias insulana;cotton boll weevil;Meloidogyne incognita;Helicoverpa armigera;Aedes aegypti;Diaphorina*

								citri;Agrotis exclamationis;Cydia pomonella;Myzus persicae;Ostrinia nubilalis;Spodoptera exigua;Spodoptera frugiperda;Bombyx mori;Spodoptera litura;Epiphyas postvittana;Leucinodes orbonalis;Trichoplusia ni;Hyphantria cunea;Pieris rapae;Chilo suppressalis;Helicoverpa punctigera;Helicoverpa zea;Chilo partellus;Blattella germanica
GCF_020775435.1	*Bacillus thuringiensis*	CH_65	Cry1Ia42,Cry2Ab41,Cry9Ea12,Cry1Ca18,Cry1Da2,Vip3Aa79,Spp1Aa1	7	Yes	cry1I,cry2A,Cry9E,cry1C,cry1D,vip3Aa,Spp1A	Coleoptera;Lepidoptera;Rhabditida;Diptera;Hemiptera;Dipteran;Blattodea;Aedes aegypti	*Leptinotarsa decemlineata;Ostrinia furnacalis;Lobesia botrana;Plutella xylostella;Earias insulana;cotton boll weevil;Meloidogyne incognita;Helicoverpa armigera;Aedes aegypti;Diaphorina citri;Agrotis exclamationis;Cydia pomonella;Myzus persicae;Ostrinia nubilalis;Spodoptera exigua;Spodoptera frugiperda;Bombyx mori;Spodoptera litura;Epiphyas postvittana;Helicoverpa punctigera;Helicoverpa zea;Chilo partellus;Blattella germanica*
GCF_020	*Bacillus*	CH_72	Cry1Aa3,Cry1Ia42,Cry	8	Yes	cry1A,cry1I,cry2A,Cr	Lepidoptera;Coleo	*Plutella xylostella;Ostrinia*

775425.1	*thuringiensis*		2Ab41,Cry9Ea12,Cry1Ca18,Cry1Da2,Vip3Aa79,Spp1Aa1			y9E,cry1C,cry1D,vip3Aa,Spp1A	ptera;Rhabditida;Diptera;Hemiptera;Dipteran;Blattodea;Aedes aegypti	*furnacalis;Helicoverpa armigera;Leucinodes orbonalis;Trichoplusia ni;Hyphantria cunea;Spodoptera frugiperda;Pieris rapae;Chilo suppressalis;Leptinotarsa decemlineata;Lobesia botrana;Earias insulana;cotton boll weevil;Meloidogyne incognita;Aedes aegypti;Diaphorina citri;Agrotis exclamationis;Cydia pomonella;Myzus persicae;Ostrinia nubilalis;Spodoptera exigua;Bombyx mori;Spodoptera litura;Epiphyas postvittana;Helicoverpa punctigera;Helicoverpa zea;Chilo partellus;Blattella germanica*
GCF_020775395.1	*Bacillus thuringiensis*	AGES_2_27_S	Cry1Ia42,Cry2Ab41,Cry9Ea12,Cry1Ca18,Cry1Da2,Cry1Aa3,Cry1Ab9,Vip3Aa79,Spp1Aa1	9	Yes	cry1I,cry2A,Cry9E,cry1C,cry1D,cry1A,cry1A,vip3Aa,Spp1A	Coleoptera;Lepidoptera;Rhabditida;Diptera;Hemiptera;Dipteran;Blattodea;Aedes aegypti	*Leptinotarsa decemlineata;Ostrinia furnacalis;Lobesia botrana;Plutella xylostella;Earias insulana;cotton boll weevil;Meloidogyne incognita;Helicoverpa armigera;Aedes aegypti;Diaphorina citri;Agrotis exclamationis;Cydia pomonella;Myzus persicae;Ostrinia nubilalis;Spodoptera exigua;Spodoptera frugiperda;Bombyx mori;Spodoptera*

								litura;Epiphyas postvittana;Leucinodes orbonalis;Trichoplusia ni;Hyphantria cunea;Pieris rapae;Chilo suppressalis;Helicoverpa punctigera;Helicoverpa zea;Chilo partellus;Blattella germanica
GCF_020775365.1	*Bacillus thuringiensis*	AGES_6_27_S	Cry1Aa3,Cry1Ia42,Cry2Ab41,Cry9Ea12,Cry1Ca18,Cry1Da2,Vip3Aa79,Spp1Aa1	8	Yes	cry1A,cry1I,cry2A,Cry9E,cry1C,cry1D,vip3Aa,Spp1A	Lepidoptera;Coleoptera;Rhabditida;Diptera;Hemiptera;Dipteran;Blattodea;Aedes aegypti	*Plutella xylostella;Ostrinia furnacalis;Helicoverpa armigera;Leucinodes orbonalis;Trichoplusia ni;Hyphantria cunea;Spodoptera frugiperda;Pieris rapae;Chilo suppressalis;Leptinotarsa decemlineata;Lobesia botrana;Earias insulana;cotton boll weevil;Meloidogyne incognita;Aedes aegypti;Diaphorina citri;Agrotis exclamationis;Cydia pomonella;Myzus persicae;Ostrinia nubilalis;Spodoptera exigua;Bombyx mori;Spodoptera litura;Epiphyas postvittana;Helicoverpa punctigera;Helicoverpa zea;Chilo partellus;Blattella germanica*
GCF_020775315.1	*Bacillus thuringiensis*	18_614	Cry1Ia42,Cry2Aa22,Cry2Ab41,Vip3Aa79,Spp1Aa1	5	Yes	cry1I,cry2A,cry2A,vip3Aa,Spp1A	Coleoptera;Lepidoptera;Rhabditida;Diptera;Hemiptera	*Leptinotarsa decemlineata;Ostrinia furnacalis;Lobesia botrana;Plutella xylostella;Earias insulana;cotton boll*

	serovar kurstaki						;Blattodea;Aedes aegypti	*weevil;Meloidogyne incognita;Helicoverpa armigera;Aedes aegypti;Diaphorina citri;Helicoverpa punctigera;Spodoptera frugiperda;Helicoverpa zea;Chilo partellus;Blattella germanica;Spodoptera litura*
GCF_020775295.1	*Bacillus thuringiensis serovar kurstaki*	18_421	Cry1Ia42,Cry2Aa22,Cry2Ab41,Vip3Aa79,Spp1Aa1	5	Yes	cry1I,cry2A,cry2A,vip3Aa,Spp1A	Coleoptera;Lepidoptera;Rhabditida;Diptera;Hemiptera;Blattodea;Aedes aegypti	*Leptinotarsa decemlineata;Ostrinia furnacalis;Lobesia botrana;Plutella xylostella;Earias insulana;cotton boll weevil;Meloidogyne incognita;Helicoverpa armigera;Aedes aegypti;Diaphorina citri;Helicoverpa punctigera;Spodoptera frugiperda;Helicoverpa zea;Chilo partellus;Blattella germanica;Spodoptera litura*
GCF_020775275.1	*Bacillus thuringiensis serovar kurstaki*	18_216	Cry1Ia42,Cry2Aa22,Cry2Ab41,Vip3Aa79,Spp1Aa1	5	Yes	cry1I,cry2A,cry2A,vip3Aa,Spp1A	Coleoptera;Lepidoptera;Rhabditida;Diptera;Hemiptera;Blattodea;Aedes aegypti	*Leptinotarsa decemlineata;Ostrinia furnacalis;Lobesia botrana;Plutella xylostella;Earias insulana;cotton boll weevil;Meloidogyne incognita;Helicoverpa armigera;Aedes aegypti;Diaphorina citri;Helicoverpa punctigera;Spodoptera frugiperda;Helicoverpa zea;Chilo*

								partellus;Blattella germanica;Spodoptera litura
GCF_020775255.1	*Bacillus thuringiensis serovar kurstaki*	18_218	Cry1Ia42,Cry2Aa22,Cry2Ab41,Cry1Ab9,Vip3Aa79,Spp1Aa1	6	Yes	cry1I,cry2A,cry2A,cry1A,vip3Aa,Spp1A	Coleoptera;Lepidoptera;Rhabditida;Diptera;Hemiptera;Blattodea;Aedes aegypti	*Leptinotarsa decemlineata;Ostrinia furnacalis;Lobesia botrana;Plutella xylostella;Earias insulana;cotton boll weevil;Meloidogyne incognita;Helicoverpa armigera;Aedes aegypti;Diaphorina citri;Leucinodes orbonalis;Trichoplusia ni;Hyphantria cunea;Spodoptera frugiperda;Pieris rapae;Chilo suppressalis;Helicoverpa punctigera;Helicoverpa zea;Chilo partellus;Blattella germanica;Spodoptera litura*
GCF_020686045.1	*Bacillus thuringiensis*	IZSPB_BC56B	Spp1Aa1	1	No	Spp1A	Lepidoptera;Blattodea	*Spodoptera litura;Blattella germanica*
GCF_020171765.1	*Bacillus thuringiensis*	AmN0-4	Spp1Aa1	1	No	Spp1A	Lepidoptera;Blattodea	*Spodoptera litura;Blattella germanica*
GCF_020005185.2	*Bacillus thuringiensis*	ZZQ-130	Cry1Aa3,Cry1Ia42,Cry2Aa22,Cry2Ab41,Vip3Aa79,Spp1Aa1,Vpa2Ah1,Vpb1Ae1	8	No	Vpa2Ah + Vpb1Ae,cry1A,cry1I,cry2A,cry2A,vip3Aa,Spp1A	Hemiptera;Lepidoptera;Coleoptera;Rhabditida;Diptera;Blattodea;Aedes aegypti	*Aphis gossypii;Plutella xylostella;Ostrinia furnacalis;Helicoverpa armigera;Leucinodes orbonalis;Trichoplusia ni;Hyphantria cunea;Spodoptera frugiperda;Pieris*

								rapae;Chilo suppressalis;Leptinotarsa decemlineata;Lobesia botrana;Earias insulana;cotton boll weevil;Meloidogyne incognita;Aedes aegypti;Diaphorina citri;Helicoverpa punctigera;Helicoverpa zea;Chilo partellus;Blattella germanica;Spodoptera litura
GCF_019192705.1	*Bacillus thuringiensis*	JRO03	Spp1Aa1	1	No	Spp1A	Lepidoptera;Blattodea	*Spodoptera litura;Blattella germanica*
GCF_018791885.1	*Bacillus thuringiensis*	SY49-1	Cry1Ia42,Cry2Aa22,Cry2Ab41,Vip3Aa79,Spp1Aa1	5	Yes	cry1I,cry2A,cry2A,vip3Aa,Spp1A	Coleoptera;Lepidoptera;Rhabditida;Diptera;Hemiptera;Blattodea;Aedes aegypti	*Leptinotarsa decemlineata;Ostrinia furnacalis;Lobesia botrana;Plutella xylostella;Earias insulana;cotton boll weevil;Meloidogyne incognita;Helicoverpa armigera;Aedes aegypti;Diaphorina citri;Helicoverpa punctigera;Spodoptera frugiperda;Helicoverpa zea;Chilo partellus;Blattella germanica;Spodoptera litura*
GCF_018603645.1	*Bacillus thuringiensis*	CF4_25	Spp1Aa1	1	Yes	Spp1A	Lepidoptera;Blattodea	*Spodoptera litura;Blattella germanica*
GCF_017751245.1	*Bacillus thuringi*	Bt-GS57	Cry1Ia42,Cry2Ab41,Cry9Ea12,Cry1Ca18,Cry1	8	Yes	cry1I,cry2A,Cry9E,cry1C,cry1D,cry1A,vip	Coleoptera;Lepidoptera;Rhabditida;	*Leptinotarsa decemlineata;Ostrinia furnacalis;Lobesia botrana;Plutella*

	ensis		Da2,Cry1Aa3,Vip3Aa79,Spp1Aa1			3Aa,Spp1A	Diptera;Hemiptera;Dipteran;Blattodea;Aedes aegypti	*xylostella;Earias insulana;cotton boll weevil;Meloidogyne incognita;Helicoverpa armigera;Aedes aegypti;Diaphorina citri;Agrotis exclamationis;Cydia pomonella;Myzus persicae;Ostrinia nubilalis;Spodoptera exigua;Spodoptera frugiperda;Bombyx mori;Spodoptera litura;Epiphyas postvittana;Leucinodes orbonalis;Trichoplusia ni;Hyphantria cunea;Pieris rapae;Chilo suppressalis;Helicoverpa punctigera;Helicoverpa zea;Chilo partellus;Blattella germanica*
GCF_017378115.1	*Bacillus thuringiensis*	TAND672	Cry10Aa4,Cry4Aa5,Mpp60Ba3,Mpp60Aa3	4	No	cry10A,cry4A,Mpp60B,Mpp60A	Diptera;Coleoptera;Hemiptera	*Aedes aegypti;cotton boll weevil;Culex pipiens;Culex quinquefasciatus;Acyrthosiphon pisum*
GCF_017312215.1	*Bacillus thuringiensis*	FCC7	Cry8Qa2,Vpa2Ai2,Vpb1Ea1,Vpb1Ea2	4	Yes	cry8Q	Coleoptera;Lepidoptera	*Anthonomus grandis;Anticarsia gemmatalis*
GCF_017165575.1	*Bacillus thuringiensis*	S601	Cry1Ba8,Mpp5Ab1,Vpa2Bb4	3	Yes	cry1B,Mpp5A	Lepidoptera;Hemiptera;Diptera;Coleoptera	*Plutella xylostella;Ostrinia nubilalis;Spodoptera frugiperda;Maruca vitrata;Manduca sexta;cotton boll weevil;Colaphellus bowringi;Leptinotarsa decemlineata*

GCF_016068365.1	*Bacillus thuringiensis*	G25-18	Cry1Ea7,Spp1Aa1	2	No	cry1E,Spp1A	Lepidoptera;Hemiptera;Rhabditida;Blattodea	*Spodoptera exigua;Spodoptera frugiperda;Malacosoma disstria;Bursaphelenchus xylophilus;Spodoptera litura;Blattella germanica*
GCF_015912835.1	*Bacillus thuringiensis*	G25-91	Cry21Ga1,Cry21Ha1,Cry21Fa1,Spp1Aa1	4	Yes	Cry21G,Cry21H,Cry21F,Spp1A	Rhabditida;Lepidoptera;Blattodea	*Heligmosomoides polygyrus bakeri;Caenorhabditis elegans;Spodoptera litura;Blattella germanica*
GCF_015881165.1	*Bacillus thuringiensis*	G25-50	Cry5Ba1,Cry5Ac1,Cry5Aa1,Spp1Aa1	4	Yes	cry5B,cry5A,cry5A,Spp1A	Rhabditida;Lepidoptera;Hymenoptera;Blattodea	*Caenorhabditis elegans;Ancylostoma ceylanicum;Heligmosomoides polygyrus bakeri;Meloidogyne incognita;Haemonchus contortus;Trichostrongylus colubriformis;Ostertagia circumcincta;Diprion pini;Cephacia abietis;Helicoverpa armigera;Spodoptera litura;Blattella germanica*
GCF_015881085.1	*Bacillus thuringiensis*	G25-45	App6Ba1,Spp1Aa1	2	No	App6B,Spp1A	Rhabditida;Coleoptera;Lepidoptera;Blattodea	*Caenorhabditis elegans;Bursaphelenchus xylophilus;Meloidogyne incognita;Hypera postica;Spodoptera litura;Blattella germanica*
GCF_015846095.1	*Bacillus thuringiensis*	G25-6	Cry70Ca1,Spp1Aa1,Vpb4Ba1	3	No	Spp1A	Lepidoptera;Blattodea	*Spodoptera litura;Blattella germanica*
GCF_015	*Bacillus*	G25-9	Spp1Aa1	1	No	Spp1A	Lepidoptera;Blatto	*Spodoptera litura;Blattella*

846085.1	*thuringiensis*						dea	*germanica*
GCF_015846045.1	*Bacillus thuringiensis*	G25-13	Spp1Aa1	1	Yes	Spp1A	Lepidoptera;Blattodea	*Spodoptera litura;Blattella germanica*
GCF_015845815.1	*Bacillus thuringiensis*	G25-123	Spp1Aa1	1	Yes	Spp1A	Lepidoptera;Blattodea	*Spodoptera litura;Blattella germanica*
GCF_015845695.1	*Bacillus thuringiensis*	G25-121	Spp1Aa1	1	Yes	Spp1A	Lepidoptera;Blattodea	*Spodoptera litura;Blattella germanica*
GCF_015845685.1	*Bacillus thuringiensis*	G25-120	Spp1Aa1	1	Yes	Spp1A	Lepidoptera;Blattodea	*Spodoptera litura;Blattella germanica*
GCF_015845285.1	*Bacillus thuringiensis*	G25-98	Cry7Ab6,Cry7Ja1,Spp1Aa1	3	Yes	cry7A,Spp1A	Coleoptera;Lepidoptera;Blattodea	*Leptinotarsa decemlineata;Henosepilachna vigintiomaculata;Acanthoscelides obtectus;Henosepilachna vigintioctomaculata;Cylas puncticollis;Spodoptera litura;Blattella germanica*
GCF_015845275.1	*Bacillus thuringiensis*	G25-96	Cry1Ia42,Cry2Aa22,Cry2Ab41,Cry1Aa17,Cry1Ac5,Vip3Aa79,Spp1Aa1	7	Yes	cry1I,cry2A,cry2A,cry1A,cry1A,vip3Aa,Spp1A	Coleoptera;Lepidoptera;Rhabditida;Diptera;Hemiptera;Blattodea;Aedes aegypti	*Leptinotarsa decemlineata;Ostrinia furnacalis;Lobesia botrana;Plutella xylostella;Earias insulana;cotton boll weevil;Meloidogyne incognita;Helicoverpa armigera;Aedes aegypti;Diaphorina citri;Leucinodes*

								orbonalis;Trichoplusia ni;Hyphantria cunea;Spodoptera frugiperda;Pieris rapae;Chilo suppressalis;Helicoverpa punctigera;Helicoverpa zea;Chilo partellus;Blattella germanica;Spodoptera litura
GCF_015845255.1	*Bacillus thuringiensis*	G25-95	Cry7Fb1,Cry7Ga2,Cry7Da3,Spp1Aa1	4	Yes	cry7G,Spp1A	Coleoptera;Lepidoptera;Blattodea	*Diabrotica undecimpunctata howardi;Diabrotica virgifera virgifera;Spodoptera litura;Blattella germanica*
GCF_015845205.1	*Bacillus thuringiensis*	G25-94	Cry7Fb1,Cry7Ga2,Cry7Da3,Spp1Aa1	4	Yes	cry7G,Spp1A	Coleoptera;Lepidoptera;Blattodea	*Diabrotica undecimpunctata howardi;Diabrotica virgifera virgifera;Spodoptera litura;Blattella germanica*
GCF_015845195.1	*Bacillus thuringiensis*	G25-93	Cry1Ab24,Cry1Ja2,Cry1Da3,Cry1Hb2,Cry1Id2,Cry1Nb1,Cry2Ad1,Cry1Ka1,Cry1Ib5,Cry1Fb4,Cry1Bb1,Vip3Af3,Vip3Ag6,Spp1Aa1,Vpa2Ac2,Vpb1Ca1	16	No	cry1A,cry1J,cry1D,cry1H,cry1I,cry1N,cry2A,cry1K,cry1I,cry1F,cry1B,vip3Af,vip3Ag,Spp1A	Lepidoptera;Coleoptera;Rhabditida;Diptera;Hemiptera;Blattodea	*Plutella xylostella;Ostrinia furnacalis;Helicoverpa armigera;Leucinodes orbonalis;Trichoplusia ni;Hyphantria cunea;Spodoptera frugiperda;Pieris rapae;Chilo suppressalis;Manduca sexta;Lobesia botrana;Diabrotica undecimpunctata howardi;Ostrinia nubilalis;Spodoptera exigua;Bombyx mori;Spodoptera litura;Epiphyas postvittana;Leptinotarsa decemlineata;Earias insulana;cotton boll weevil;Meloidogyne*

								incognita;Colaphellus bowringi;Aedes aegypti;Diaphorina citri;Artogeia rapae;Agrotis ipsilon;Diabrotica virgifera virgifera;Maruca vitrata;Spodoptera littoralis;Chrysodeixis chalcites;Blattella germanica
GCF_015845125.1	*Bacillus thuringiensis*	G25-87	Cry21Fa1,Cry21Ha1,Cry21Ga1,Spp1Aa1	4	Yes	Cry21F,Cry21H,Cry21G,Spp1A	Rhabditida;Lepidoptera;Blattodea	*Heligmosomoides polygyrus bakeri;Caenorhabditis elegans;Spodoptera litura;Blattella germanica*
GCF_015845085.1	*Bacillus thuringiensis*	G25-85	Cry1Ba8,Mpp5Ab1,Spp1Aa1,Vpa2Bb4,Vpb1Bb2,Vpb1Bc1	6	Yes	cry1B,Mpp5A,Spp1A	Lepidoptera;Hemiptera;Diptera;Coleoptera;Blattodea	*Plutella xylostella;Ostrinia nubilalis;Spodoptera frugiperda;Maruca vitrata;Manduca sexta;cotton boll weevil;Colaphellus bowringi;Leptinotarsa decemlineata;Spodoptera litura;Blattella germanica*
GCF_015844985.1	*Bacillus thuringiensis*	G25-78	Cry21Fa1,Cry21Ga1,Cry21Ha1,Spp1Aa1	4	Yes	Cry21F,Cry21G,Cry21H,Spp1A	Rhabditida;Lepidoptera;Blattodea	*Heligmosomoides polygyrus bakeri;Caenorhabditis elegans;Spodoptera litura;Blattella germanica*
GCF_015844945.1	*Bacillus thuringiensis*	G25-76	Cry1Ba8,Mpp5Ab1,Spp1Aa1,Vpa2Bb4,Vpb1Bc1,Vpb1Bb2	6	Yes	cry1B,Mpp5A,Spp1A	Lepidoptera;Hemiptera;Diptera;Coleoptera;Blattodea	*Plutella xylostella;Ostrinia nubilalis;Spodoptera frugiperda;Maruca vitrata;Manduca sexta;cotton boll weevil;Colaphellus bowringi;Leptinotarsa decemlineata;Spodoptera*

								litura;Blattella germanica
GCF_015844555.1	*Bacillus thuringiensis*	G25-51	Cry5Ca2,Cry5Fa1,Spp1Aa1	3	Yes	cry5C,Spp1A	Rhabditida;Lepidoptera;Blattodea	*Meloidogyne incognita;Spodoptera litura;Blattella germanica*
GCF_014982505.1	*Bacillus thuringiensis*	871Blist1	Spp1Aa1	1	Yes	Spp1A	Lepidoptera;Blattodea	*Spodoptera litura;Blattella germanica*
GCF_014932945.1	*Bacillus thuringiensis*	s2744	Spp1Aa1	1	Yes	Spp1A	Lepidoptera;Blattodea	*Spodoptera litura;Blattella germanica*
GCF_014932895.1	*Bacillus thuringiensis*	s1930			No			
GCF_014932865.1	*Bacillus thuringiensis*	s1783	Mpp3Aa1,Mpp4Aa1,Mpp2Aa4,Mtx1Aa1,Spp1Aa1,Tpp1Aa2,Tpp2Aa2	7	No	Tpp1A+Tpp2A,Mpp3A,Mpp2A,Mtx1A,Spp1A	Diptera;Coleoptera;Lepidoptera;Blattodea	*Culex quinquefasciatus;Anopheles stephensi;Culex pipiens;Aedes atropalpus;Anopheles gambiae;Aedes aegypti;Cydia pomonella;Anthonomus grandis Culex quinquefasciatus;Aedes albopictus;Spodoptera litura;Blattella germanica*
GCF_014932845.1	*Bacillus thuringiensis*	S2728	Spp1Aa1	1	Yes	Spp1A	Lepidoptera;Blattodea	*Spodoptera litura;Blattella germanica*
GCF_013377585.1	*Bacillus thuringiensis serovar*	KNU-25	Cry1Ab9,Cry1Aa3,Cry1Ia42,Cry2Aa22,Cry2Ab41,Cry1Ac4,Vip3Aa79,Spp1Aa1	8	Yes	cry1A,cry1A,cry1I,cry2A,cry2A,cry1A,vip3Aa,Spp1A	Lepidoptera;Coleoptera;Rhabditida;Diptera;Hemiptera;Blattodea;Aedes	*Plutella xylostella;Ostrinia furnacalis;Helicoverpa armigera;Leucinodes orbonalis;Trichoplusia ni;Hyphantria*

	kurstaki						aegypti	*cunea;Spodoptera frugiperda;Pieris rapae;Chilo suppressalis;Leptinotarsa decemlineata;Lobesia botrana;Earias insulana;cotton boll weevil;Meloidogyne incognita;Aedes aegypti;Diaphorina citri;Helicoverpa punctigera;Helicoverpa zea;Chilo partellus;Blattella germanica;Spodoptera litura*
GCF_013377575.1	*Bacillus thuringiensis serovar israelensis*	KNU-26	Cry11Aa3,Cry10Aa4,Cry4Ba7,Cry4Aa5,Cyt1Ca1,Cyt1Aa11,Cyt2Ba17,Mpp60Ba3,Mpp60Aa3	9	No	cry11A,cry10A,cry4B,cry4A,cyt1A,cyt2B,Mpp60B,Mpp60A	Diptera;Coleoptera;Hemiptera;Lepidoptera	*Aedes aegypti;Anopheles albimanus;Culex quinquefasciatu;Culex pipiens;cotton boll weevil;Anopheles gambiae;Diaphorina citri;Culex quinquefasciatus;Acyrthosiphon pisum;Culex pipien;Plutella xylostella;Anopheles stephensi;Bradysia difformis*
GCF_013375565.1	*Bacillus thuringiensis*	B116	Spp1Aa1	1	Yes	Spp1A	Lepidoptera;Blattodea	*Spodoptera litura;Blattella germanica*
GCF_013346135.1	*Bacillus thuringiensis*	IIF1SG-B5	Spp1Aa1	1	Yes	Spp1A	Lepidoptera;Blattodea	*Spodoptera litura;Blattella germanica*
GCF_013346115.1	*Bacillus thuringiensis*	IIF4SW-P2	Spp1Aa1	1	Yes	Spp1A	Lepidoptera;Blattodea	*Spodoptera litura;Blattella germanica*

GCF_013345935.1	*Bacillus thuringiensis*	IIF7SG-B4	Spp1Aa1	1	Yes	Spp1A	Lepidoptera;Blattodea	*Spodoptera litura;Blattella germanica*
GCF_013345915.1	*Bacillus thuringiensis*	IIF4SW-P5	Spp1Aa1	1	Yes	Spp1A	Lepidoptera;Blattodea	*Spodoptera litura;Blattella germanica*
GCF_013345905.1	*Bacillus thuringiensis*	IIF2SG-B1	Spp1Aa1	1	Yes	Spp1A	Lepidoptera;Blattodea	*Spodoptera litura;Blattella germanica*
GCF_013345885.1	*Bacillus thuringiensis*	IIF4SW-B3	Spp1Aa1	1	Yes	Spp1A	Lepidoptera;Blattodea	*Spodoptera litura;Blattella germanica*
GCF_013345765.1	*Bacillus thuringiensis*	IIF3SG-B3	Spp1Aa1	1	Yes	Spp1A	Lepidoptera;Blattodea	*Spodoptera litura;Blattella germanica*
GCF_013267795.1	*Bacillus thuringiensis*	FDAARGOS_794			No			
GCF_013267335.1	*Bacillus thuringiensis*	FDAARGOS_793	Bmp1-other	1	No			
GCF_013267295.1	*Bacillus thuringiensis*	FDAARGOS_796	Cry11Aa3,Cry10Aa4,Cry4Ba7,Cry4Aa5,Cyt1Ca1,Cyt1Aa11,Cyt2Ba17	7	No	cry11A,cry10A,cry4B,cry4A,cyt1A,cyt2B	Diptera;Coleoptera;Hemiptera;Lepidoptera	*Aedes aegypti;Anopheles albimanus;Culex quinquefasciatu;Culex pipiens;cotton boll weevil;Anopheles gambiae;Diaphorina citri;Culex quinquefasciatus;Acyrthosiphon pisum;Culex pipien;Plutella*

								xylostella;Anopheles stephensi;Bradysia difformis
GCF_013004265.1	*Bacillus thuringiensis*	CH_48	Spp1Aa1	1	No	Spp1A	Lepidoptera;Blattodea	*Spodoptera litura;Blattella germanica*
GCF_012222085.2	*Bacillus thuringiensis serovar andalousiensis*	NRRL B-23139			No			
GCF_009935745.1	*Bacillus thuringiensis*	26-1	Cry1Ia42,Cry2Ab41,Cry9Ea12,Cry1Da2,Cry1Ca18,Vip3Aa79,Spp1Aa1	7	Yes	cry1I,cry2A,Cry9E,cry1D,cry1C,vip3Aa,Spp1A	Coleoptera;Lepidoptera;Rhabditida;Diptera;Hemiptera;Dipteran;Blattodea;Aedes aegypti	*Leptinotarsa decemlineata;Ostrinia furnacalis;Lobesia botrana;Plutella xylostella;Earias insulana;cotton boll weevil;Meloidogyne incognita;Helicoverpa armigera;Aedes aegypti;Diaphorina citri;Agrotis exclamationis;Cydia pomonella;Ostrinia nubilalis;Spodoptera exigua;Spodoptera frugiperda;Bombyx mori;Spodoptera litura;Epiphyas postvittana;Myzus persicae;Helicoverpa punctigera;Helicoverpa zea;Chilo partellus;Blattella germanica*
GCF_009935545.1	*Bacillus thuringi*	14-2	Cry1Ia42,Cry2Ab41,Cry9Ea12,Cry1Da2,Cry1	7	Yes	cry1I,cry2A,Cry9E,cry1D,cry1C,vip3Aa,Sp	Coleoptera;Lepidoptera;Rhabditida;	*Leptinotarsa decemlineata;Ostrinia furnacalis;Lobesia botrana;Plutella*

	ensis		Ca18,Vip3Aa79,Spp1Aa1			p1A	Diptera;Hemiptera;Dipteran;Blattodea;Aedes aegypti	*xylostella;Earias insulana;cotton boll weevil;Meloidogyne incognita;Helicoverpa armigera;Aedes aegypti;Diaphorina citri;Agrotis exclamationis;Cydia pomonella;Ostrinia nubilalis;Spodoptera exigua;Spodoptera frugiperda;Bombyx mori;Spodoptera litura;Epiphyas postvittana;Myzus persicae;Helicoverpa punctigera;Helicoverpa zea;Chilo partellus;Blattella germanica*
GCF_009935505.1	*Bacillus thuringiensis*	20-1	Cry1Ia42,Cry2Ab41,Cry9Ea12,Cry1Da2,Cry1Ca18,Vip3Aa79,Spp1Aa1	7	Yes	cry1I,cry2A,Cry9E,cry1D,cry1C,vip3Aa,Spp1A	Coleoptera;Lepidoptera;Rhabditida;Diptera;Hemiptera;Dipteran;Blattodea;Aedes aegypti	*Leptinotarsa decemlineata;Ostrinia furnacalis;Lobesia botrana;Plutella xylostella;Earias insulana;cotton boll weevil;Meloidogyne incognita;Helicoverpa armigera;Aedes aegypti;Diaphorina citri;Agrotis exclamationis;Cydia pomonella;Ostrinia nubilalis;Spodoptera exigua;Spodoptera frugiperda;Bombyx mori;Spodoptera litura;Epiphyas postvittana;Myzus persicae;Helicoverpa punctigera;Helicoverpa zea;Chilo partellus;Blattella germanica*

GCF_009935435.1	*Bacillus thuringiensis*	21-1	Cry1Ia42,Cry2Ab41,Cry9Ea12,Cry1Da2,Cry1Ca18,Vip3Aa79,Spp1Aa1	7	Yes	cry1I,cry2A,Cry9E,cry1D,cry1C,vip3Aa,Spp1A	Coleoptera;Lepidoptera;Rhabditida;Diptera;Hemiptera;Dipteran;Blattodea;Aedes aegypti	*Leptinotarsa decemlineata;Ostrinia furnacalis;Lobesia botrana;Plutella xylostella;Earias insulana;cotton boll weevil;Meloidogyne incognita;Helicoverpa armigera;Aedes aegypti;Diaphorina citri;Agrotis exclamationis;Cydia pomonella;Ostrinia nubilalis;Spodoptera exigua;Spodoptera frugiperda;Bombyx mori;Spodoptera litura;Epiphyas postvittana;Myzus persicae;Helicoverpa punctigera;Helicoverpa zea;Chilo partellus;Blattella germanica*
GCF_009935335.1	*Bacillus thuringiensis*	25-6	Cry1Ia42,Cry2Ab41,Cry9Ea12,Cry1Ca18,Cry1Da2,Vip3Aa79,Spp1Aa1	7	Yes	cry1I,cry2A,Cry9E,cry1C,cry1D,vip3Aa,Spp1A	Coleoptera;Lepidoptera;Rhabditida;Diptera;Hemiptera;Dipteran;Blattodea;Aedes aegypti	*Leptinotarsa decemlineata;Ostrinia furnacalis;Lobesia botrana;Plutella xylostella;Earias insulana;cotton boll weevil;Meloidogyne incognita;Helicoverpa armigera;Aedes aegypti;Diaphorina citri;Agrotis exclamationis;Cydia pomonella;Myzus persicae;Ostrinia nubilalis;Spodoptera exigua;Spodoptera frugiperda;Bombyx mori;Spodoptera litura;Epiphyas postvittana;Helicoverpa*

								punctigera;Helicoverpa zea;Chilo partellus;Blattella germanica
GCF_009935315.1	*Bacillus thuringiensis*	27-3	Cry1Ia42,Cry2Ab41,Cry9Ea12,Cry1Ca18,Cry1Da2,Cry1Aa3,Vip3Aa79,Spp1Aa1	8	Yes	cry1I,cry2A,Cry9E,cry1C,cry1D,cry1A,vip3Aa,Spp1A	Coleoptera;Lepidoptera;Rhabditida;Diptera;Hemiptera;Dipteran;Blattodea;Aedes aegypti	*Leptinotarsa decemlineata;Ostrinia furnacalis;Lobesia botrana;Plutella xylostella;Earias insulana;cotton boll weevil;Meloidogyne incognita;Helicoverpa armigera;Aedes aegypti;Diaphorina citri;Agrotis exclamationis;Cydia pomonella;Myzus persicae;Ostrinia nubilalis;Spodoptera exigua;Spodoptera frugiperda;Bombyx mori;Spodoptera litura;Epiphyas postvittana;Leucinodes orbonalis;Trichoplusia ni;Hyphantria cunea;Pieris rapae;Chilo suppressalis;Helicoverpa punctigera;Helicoverpa zea;Chilo partellus;Blattella germanica*
GCF_009935245.1	*Bacillus thuringiensis*	30-1	Cry1Ia42,Cry2Ab41,Cry9Ea12,Cry1Ca18,Cry1Da2,Vip3Aa79,Spp1Aa1	7	Yes	cry1I,cry2A,Cry9E,cry1C,cry1D,vip3Aa,Spp1A	Coleoptera;Lepidoptera;Rhabditida;Diptera;Hemiptera;Dipteran;Blattodea;Aedes aegypti	*Leptinotarsa decemlineata;Ostrinia furnacalis;Lobesia botrana;Plutella xylostella;Earias insulana;cotton boll weevil;Meloidogyne incognita;Helicoverpa armigera;Aedes aegypti;Diaphorina citri;Agrotis exclamationis;Cydia pomonella;Myzus persicae;Ostrinia*

								nubilalis;Spodoptera exigua;Spodoptera frugiperda;Bombyx mori;Spodoptera litura;Epiphyas postvittana;Helicoverpa punctigera;Helicoverpa zea;Chilo partellus;Blattella germanica
GCF_009935235.1	*Bacillus thuringiensis*	30-4	Cry1Ia42,Cry2Ab41,Cry9Ea12,Cry1Da2,Cry1Ca18,Vip3Aa79,Spp1Aa1	7	Yes	cry1I,cry2A,Cry9E,cry1D,cry1C,vip3Aa,Spp1A	Coleoptera;Lepidoptera;Rhabditida;Diptera;Hemiptera;Dipteran;Blattodea;Aedes aegypti	*Leptinotarsa decemlineata;Ostrinia furnacalis;Lobesia botrana;Plutella xylostella;Earias insulana;cotton boll weevil;Meloidogyne incognita;Helicoverpa armigera;Aedes aegypti;Diaphorina citri;Agrotis exclamationis;Cydia pomonella;Ostrinia nubilalis;Spodoptera exigua;Spodoptera frugiperda;Bombyx mori;Spodoptera litura;Epiphyas postvittana;Myzus persicae;Helicoverpa punctigera;Helicoverpa zea;Chilo partellus;Blattella germanica*
GCF_009865315.1	*Bacillus thuringiensis*	SID4914	Spp1Aa1	1	Yes	Spp1A	Lepidoptera;Blattodea	*Spodoptera litura;Blattella germanica*
GCF_009647315.1	*Bacillus thuringiensis*	4A1	Cry1Ba8,Cry1Ab9,Mpp5Ab1,Vpa2Bb4	4	Yes	cry1B,cry1A,Mpp5A	Lepidoptera;Hemiptera;Diptera;Coleoptera	*Plutella xylostella;Ostrinia nubilalis;Spodoptera frugiperda;Maruca vitrata;Manduca*

								sexta;cotton boll weevil;Ostrinia furnacalis;Helicoverpa armigera;Leucinodes orbonalis;Trichoplusia ni;Hyphantria cunea;Pieris rapae;Chilo suppressalis;Colaphellus bowringi;Leptinotarsa decemlineata
GCF_009647275.1	*Bacillus thuringiensis*	4AZ2			No			
GCF_009647255.1	*Bacillus thuringiensis*	4A2	Cry1Ba8,Cry1Ab9,Mpp5Ab1,Vpa2Bb4	4	Yes	cry1B,cry1A,Mpp5A	Lepidoptera;Hemiptera;Diptera;Coleoptera	*Plutella xylostella;Ostrinia nubilalis;Spodoptera frugiperda;Maruca vitrata;Manduca sexta;cotton boll weevil;Ostrinia furnacalis;Helicoverpa armigera;Leucinodes orbonalis;Trichoplusia ni;Hyphantria cunea;Pieris rapae;Chilo suppressalis;Colaphellus bowringi;Leptinotarsa decemlineata*
GCF_009647195.1	*Bacillus thuringiensis*	4B1	Cry28Aa2,Cry26Aa1,Mpp83Aa1	3	No			
GCF_009647175.1	*Bacillus thuringiensis*	4D2	Cry1Ia42,Cry2Aa22,Cry2Ab41,Vip3Aa79,Spp1Aa1	5	Yes	cry1I,cry2A,cry2A,vip3Aa,Spp1A	Coleoptera;Lepidoptera;Rhabditida;Diptera;Hemiptera;Blattodea;Aedes aegypti	*Leptinotarsa decemlineata;Ostrinia furnacalis;Lobesia botrana;Plutella xylostella;Earias insulana;cotton boll weevil;Meloidogyne incognita;Helicoverpa*

								armigera;Aedes aegypti;Diaphorina citri;Helicoverpa punctigera;Spodoptera frugiperda;Helicoverpa zea;Chilo partellus;Blattella germanica;Spodoptera litura
GCF_009647145.1	*Bacillus thuringiensis*	4D3	Cry1Ba8	1	Yes	cry1B	Lepidoptera;Hemiptera;Diptera;Coleoptera	*Plutella xylostella;Ostrinia nubilalis;Spodoptera frugiperda;Maruca vitrata;Manduca sexta;cotton boll weevil*
GCF_009647135.1	*Bacillus thuringiensis*	4D5	Cry1Ia42,Cry2Aa22,Cry2Ab41,Vip3Aa79,Spp1Aa1	5	Yes	cry1I,cry2A,cry2A,vip3Aa,Spp1A	Coleoptera;Lepidoptera;Rhabditida;Diptera;Hemiptera;Blattodea;Aedes aegypti	*Leptinotarsa decemlineata;Ostrinia furnacalis;Lobesia botrana;Plutella xylostella;Earias insulana;cotton boll weevil;Meloidogyne incognita;Helicoverpa armigera;Aedes aegypti;Diaphorina citri;Helicoverpa punctigera;Spodoptera frugiperda;Helicoverpa zea;Chilo partellus;Blattella germanica;Spodoptera litura*
GCF_009647095.1	*Bacillus thuringiensis*	4E7	Cry1Ac9,Spp1Aa1	2	Yes	cry1A,Spp1A	Lepidoptera;Blattodea	*Plutella xylostella;Ostrinia furnacalis;Helicoverpa armigera;Leucinodes orbonalis;Trichoplusia ni;Hyphantria cunea;Spodoptera frugiperda;Pieris rapae;Chilo suppressalis;Spodoptera litura;Blattella germanica*

GCF_009647075.1	*Bacillus thuringiensis*	4F1	Cry2Aa22,Cry2Ab4,Cry1Ia42,Cry1Ac2,Cry1Ea7,Vip3Aa59,Spp1Aa1	7	Yes	cry2A,cry2A,cry1I,cry1A,cry1E,vip3Aa,Spp1A	Lepidoptera;Diptera;Hemiptera;Coleoptera;Rhabditida;Blattodea;Aedes aegypti	*Helicoverpa armigera;Aedes aegypti;Diaphorina citri;Leptinotarsa decemlineata;Ostrinia furnacalis;Lobesia botrana;Plutella xylostella;Earias insulana;cotton boll weevil;Meloidogyne incognita;Leucinodes orbonalis;Trichoplusia ni;Hyphantria cunea;Spodoptera frugiperda;Pieris rapae;Chilo suppressalis;Spodoptera exigua;Malacosoma disstria;Bursaphelenchus xylophilus;Helicoverpa punctigera;Helicoverpa zea;Chilo partellus;Blattella germanica;Spodoptera litura*
GCF_009647015.1	*Bacillus thuringiensis*	4F2	Cry1Ac2,Spp1Aa1	2	Yes	cry1A,Spp1A	Lepidoptera;Blattodea	*Plutella xylostella;Ostrinia furnacalis;Helicoverpa armigera;Leucinodes orbonalis;Trichoplusia ni;Hyphantria cunea;Spodoptera frugiperda;Pieris rapae;Chilo suppressalis;Spodoptera litura;Blattella germanica*
GCF_009646995.1	*Bacillus thuringiensis*	4F3	Cry2Aa22,Cry2Ab4,Cry1Ea7,Vip3Aa59,Spp1Aa1	5	Yes	cry2A,cry2A,cry1E,vip3Aa,Spp1A	Lepidoptera;Diptera;Hemiptera;Rhabditida;Blattodea;Aedes aegypti	*Helicoverpa armigera;Aedes aegypti;Diaphorina citri;Spodoptera exigua;Spodoptera frugiperda;Malacosoma*

								disstria;Bursaphelenchus xylophilus;Helicoverpa punctigera;Helicoverpa zea;Chilo partellus;Blattella germanica;Spodoptera litura
GCF_009646985.1	*Bacillus thuringiensis*	4F4	Cry2Aa22,Cry2Ab4,Cry1Ia42,Cry1Ac2,Cry1Ea7,Vip3Aa59,Spp1Aa1	7	Yes	cry2A,cry2A,cry1I,cry1A,cry1E,vip3Aa,Spp1A	Lepidoptera;Diptera;Hemiptera;Coleoptera;Rhabditida;Blattodea;Aedes aegypti	*Helicoverpa armigera;Aedes aegypti;Diaphorina citri;Leptinotarsa decemlineata;Ostrinia furnacalis;Lobesia botrana;Plutella xylostella;Earias insulana;cotton boll weevil;Meloidogyne incognita;Leucinodes orbonalis;Trichoplusia ni;Hyphantria cunea;Spodoptera frugiperda;Pieris rapae;Chilo suppressalis;Spodoptera exigua;Malacosoma disstria;Bursaphelenchus xylophilus;Helicoverpa punctigera;Helicoverpa zea;Chilo partellus;Blattella germanica;Spodoptera litura*
GCF_009646975.1	*Bacillus thuringiensis*	4G2	Cry1Bd1,Cry1Ga1,Cry1Ib3,Cry1Db1,Cry1Fb5,Cry2Ac6,Cry1Cb2,Cry1Gb2,Vip3Af3,Spp1Aa1,Vpa2Ae3,Vpb1Ac1	12	Yes	Vpa2Ae + Vpb1Ac,cry1B,cry1G,cry1I,cry1D,cry1F,cry2A,cry1C,cry1G,vip3Af,Spp1A	Hemiptera;Lepidoptera;Diptera;Coleoptera;Rhabditida;Dipteran;Blattodea	*Aphis gossypii;Plutella xylostella;Ostrinia nubilalis;Spodoptera frugiperda;Maruca vitrata;Manduca sexta;cotton boll weevil;Pieris rapae;Thaumetopoea*

								pityocampa;Myzus persicae;Leptinotarsa decemlineata;Ostrinia furnacalis;Lobesia botrana;Earias insulana;Meloidogyne incognita;Spodoptera exigua;Bombyx mori;Spodoptera litura;Epiphyas postvittana;Agrotis ipsilon;Diabrotica virgifera virgifera;Helicoverpa armigera;Aedes aegypti;Diaphorina citri;Chilo suppressalis;Blattella germanica
GCF_009646925.1	*Bacillus thuringiensis*	4G3	Cry1Bd1,Cry1Ga1,Cry1Ib3,Cry1Fb5,Cry2Ac6,Cry1Cb2,Cry1Gb2,Vip3Aa17,Spp1Aa1,Vpa2Ae3,Vpb1Ac1	11	Yes	Vpa2Ae + Vpb1Ac,cry1B,cry1G,cry1I,cry1F,cry2A,cry1C,cry1G,vip3Aa,Spp1A	Hemiptera;Lepidoptera;Diptera;Coleoptera;Rhabditida;Dipteran;Blattodea;Aedes aegypti	*Aphis gossypii;Plutella xylostella;Ostrinia nubilalis;Spodoptera frugiperda;Maruca vitrata;Manduca sexta;cotton boll weevil;Pieris rapae;Thaumetopoea pityocampa;Myzus persicae;Leptinotarsa decemlineata;Ostrinia furnacalis;Lobesia botrana;Earias insulana;Meloidogyne incognita;Agrotis ipsilon;Diabrotica virgifera virgifera;Helicoverpa armigera;Aedes aegypti;Diaphorina citri;Helicoverpa*

								punctigera;Helicoverpa zea;Chilo partellus;Blattella germanica;Spodoptera litura
GCF_009646895.1	*Bacillus thuringiensis*	4H4			No			
GCF_009646865.1	*Bacillus thuringiensis*	4I2	Spp1Aa1	1	Yes	Spp1A	Lepidoptera;Blattodea	*Spodoptera litura;Blattella germanica*
GCF_009646855.1	*Bacillus thuringiensis*	4I1	Spp1Aa1	1	Yes	Spp1A	Lepidoptera;Blattodea	*Spodoptera litura;Blattella germanica*
GCF_009646805.1	*Bacillus thuringiensis*	4I5	Cry1Ab9,Cry1Ba4	2	Yes	cry1A,cry1B	Lepidoptera;Hemiptera;Diptera;Coleoptera	*Plutella xylostella;Ostrinia furnacalis;Helicoverpa armigera;Leucinodes orbonalis;Trichoplusia ni;Hyphantria cunea;Spodoptera frugiperda;Pieris rapae;Chilo suppressalis;Ostrinia nubilalis;Maruca vitrata;Manduca sexta;cotton boll weevil*
GCF_009646775.1	*Bacillus thuringiensis*	4K3			No			
GCF_009646745.1	*Bacillus thuringiensis*	4K7			Yes			
GCF_009646735.1	*Bacillus thuringi*	4L2	Cry2Ab4,Cry2Aa8,Vip3Aa59	3	No	cry2A,cry2A,vip3Aa	Lepidoptera;Diptera;Hemiptera;Blatt	*Helicoverpa armigera;Aedes aegypti;Diaphorina citri;Helicoverpa*

	ensis						odea;Aedes aegypti	*punctigera;Spodoptera frugiperda;Helicoverpa zea;Chilo partellus;Blattella germanica*
GCF_009646715.1	*Bacillus thuringiensis*	4L1	Cry1Ia42,Cry2Aa22,Cry2Ab4,Vip3Aa59	4	No	cry1I,cry2A,cry2A,vip3Aa	Coleoptera;Lepidoptera;Rhabditida;Diptera;Hemiptera;Blattodea;Aedes aegypti	*Leptinotarsa decemlineata;Ostrinia furnacalis;Lobesia botrana;Plutella xylostella;Earias insulana;cotton boll weevil;Meloidogyne incognita;Helicoverpa armigera;Aedes aegypti;Diaphorina citri;Helicoverpa punctigera;Spodoptera frugiperda;Helicoverpa zea;Chilo partellus;Blattella germanica*
GCF_009646675.1	*Bacillus thuringiensis*	4M2	Cry1Ea7,Cry1Ma1,Vip3Ba2,Mpp15Aa1,Mpp93Aa1	5	No	cry1E,cry1M,vip3B,Mpp15A,Mpp93A	Lepidoptera;Hemiptera;Rhabditida;Pieris rapae;Cydia pomonella	*Spodoptera exigua;Spodoptera frugiperda;Malacosoma disstria;Bursaphelenchus xylophilus;corn earworm (Helicoverpa zea);black cut worm (Agrotis ipsilon);cabbage looper (Trichoplusia ni);saltmarch caterpillar (Estigmene acrea);tobacco budworm (Heliothis virescens);and lettuce armyworm (Pseudaletia unipuncta).;Agrotis ipsilon;Helicoverpa armigera;Chrysodeixis includens;Ostrinia nubilalis;Manduca sexta*

GCF_009646665.1	*Bacillus thuringiensis*	4M1	Cry1Ea7	1	No	cry1E	Lepidoptera;Hemiptera;Rhabditida	*Spodoptera exigua;Spodoptera frugiperda;Malacosoma disstria;Bursaphelenchus xylophilus*
GCF_009646615.1	*Bacillus thuringiensis*	4M5	Cry1Na1,Cry1Ie3,Cry9Aa4,Cry9Ee3,Cry9Da3,Vip3Aa51	6	Yes	cry1N,cry1I,Cry9A,Cry9E,Cry9D,vip3Aa	Lepidoptera;Coleoptera;Rhabditida;Blattodea;Aedes aegypti	*Plutella xylostella;Ostrinia furnacalis;Colaphellus bowringi;Leptinotarsa decemlineata;Lobesia botrana;Earias insulana;cotton boll weevil;Meloidogyne incognita;Pieris brassicae;Phthorimaea operculella;Chilo suppressalis;Agrotis exclamationis;Cydia pomonella;Bombyx mori;Hyphantria cunea;Helicoverpa punctigera;Spodoptera frugiperda;Helicoverpa zea;Chilo partellus;Blattella germanica*
GCF_009646555.1	*Bacillus thuringiensis*	4P1	Cry1Ma2,Cry2Ba1,Vip3Ba2,Mpp15Aa1,Mpp93Aa1	5	Yes	cry1M,cry2B,vip3B,Mpp15A,Mpp93A	Lepidoptera;Diptera;Pieris rapae;Cydia pomonella	*corn earworm (Helicoverpa zea);black cut worm (Agrotis ipsilon);cabbage looper (Trichoplusia ni);saltmarch caterpillar (Estigmene acrea);tobacco budworm (Heliothis virescens);and lettuce armyworm (Pseudaletia unipuncta).;Manduca sexta;Ostrinia nubilalis;Aedes aegypti;Agrotis ipsilon;Spodoptera*

								frugiperda;Helicoverpa armigera;Spodoptera exigua;Chrysodeixis includens
GCF_009646525.1	*Bacillus thuringiensis*	4Q4	Mpp60Ba3,Mpp60Aa3	2	No	Mpp60B,Mpp60A	Diptera	*Culex quinquefasciatus*
GCF_009646515.1	*Bacillus thuringiensis*	4S2	Cry7Fb1	1	Yes			
GCF_009646485.1	*Bacillus thuringiensis*	4S3	Cry7Fb1	1	Yes			
GCF_009646455.1	*Bacillus thuringiensis*	4XX1	Bmp1-other	1	No			
GCF_009646385.1	*Bacillus thuringiensis*	4XX5	Spp1Aa1	1	Yes	Spp1A	Lepidoptera;Blattodea	*Spodoptera litura;Blattella germanica*
GCF_009646365.1	*Bacillus thuringiensis*	4XX3	Bmp1-other	1	No			
GCF_009025915.1	*Bacillus thuringiensis*	JW-1	Cry11Aa3,Cry10Aa4,Cry4Ba7,Cry4Aa5,Cyt1Ca1,Cyt1Aa11,Cyt2Ba17	7	No	cry11A,cry10A,cry4B,cry4A,cyt1A,cyt2B	Diptera;Coleoptera;Hemiptera;Lepidoptera	*Aedes aegypti;Anopheles albimanus;Culex quinquefasciatu;Culex pipiens;cotton boll weevil;Anopheles gambiae;Diaphorina citri;Culex quinquefasciatus;Acyrthosiphon pisum;Culex pipien;Plutella*

								xylostella;Anopheles stephensi;Bradysia difformis
GCF_008923385.1	*Bacillus thuringiensis*	RM11(2019)	Spp1Aa1	1	Yes	Spp1A	Lepidoptera;Blattodea	*Spodoptera litura;Blattella germanica*
GCF_008829845.1	*Bacillus thuringiensis*	TB08	Cry1Ia42,Cry2Aa22,Cry2Ab41,Vip3Aa79,Spp1Aa1	5	Yes	cry1I,cry2A,cry2A,vip3Aa,Spp1A	Coleoptera;Lepidoptera;Rhabditida;Diptera;Hemiptera;Blattodea;Aedes aegypti	*Leptinotarsa decemlineata;Ostrinia furnacalis;Lobesia botrana;Plutella xylostella;Earias insulana;cotton boll weevil;Meloidogyne incognita;Helicoverpa armigera;Aedes aegypti;Diaphorina citri;Helicoverpa punctigera;Spodoptera frugiperda;Helicoverpa zea;Chilo partellus;Blattella germanica;Spodoptera litura*
GCF_008829815.1	*Bacillus thuringiensis*	QB15	Cry1Ia42,Cry2Aa22,Cry2Ab41,Vip3Aa79,Spp1Aa1	5	Yes	cry1I,cry2A,cry2A,vip3Aa,Spp1A	Coleoptera;Lepidoptera;Rhabditida;Diptera;Hemiptera;Blattodea;Aedes aegypti	*Leptinotarsa decemlineata;Ostrinia furnacalis;Lobesia botrana;Plutella xylostella;Earias insulana;cotton boll weevil;Meloidogyne incognita;Helicoverpa armigera;Aedes aegypti;Diaphorina citri;Helicoverpa punctigera;Spodoptera frugiperda;Helicoverpa zea;Chilo partellus;Blattella germanica;Spodoptera litura*
GCF_008	*Bacillus*	PT18	Cry1Ia42,Cry2Ab41,Cr	7	Yes	cry1I,cry2A,Cry9E,cr	Coleoptera;Lepido	*Leptinotarsa decemlineata;Ostrinia*

829805.1	*thuringiensis*		y9Ea12,Cry1Ca18,Cry1Da2,Vip3Aa79,Spp1Aa1			y1C,cry1D,vip3Aa,Spp1A	ptera;Rhabditida;Diptera;Hemiptera;Dipteran;Blattodea;Aedes aegypti	*furnacalis;Lobesia botrana;Plutella xylostella;Earias insulana;cotton boll weevil;Meloidogyne incognita;Helicoverpa armigera;Aedes aegypti;Diaphorina citri;Agrotis exclamationis;Cydia pomonella;Myzus persicae;Ostrinia nubilalis;Spodoptera exigua;Spodoptera frugiperda;Bombyx mori;Spodoptera litura;Epiphyas postvittana;Helicoverpa punctigera;Helicoverpa zea;Chilo partellus;Blattella germanica*
GCF_008829775.1	*Bacillus thuringiensis*	DBG15	Cry1Ia42,Cry2Aa22,Cry2Ab41,Vip3Aa79,Spp1Aa1	5	Yes	cry1I,cry2A,cry2A,vip3Aa,Spp1A	Coleoptera;Lepidoptera;Rhabditida;Diptera;Hemiptera;Blattodea;Aedes aegypti	*Leptinotarsa decemlineata;Ostrinia furnacalis;Lobesia botrana;Plutella xylostella;Earias insulana;cotton boll weevil;Meloidogyne incognita;Helicoverpa armigera;Aedes aegypti;Diaphorina citri;Helicoverpa punctigera;Spodoptera frugiperda;Helicoverpa zea;Chilo partellus;Blattella germanica;Spodoptera litura*
GCF_008829765.1	*Bacillus thuringiensis*	PT02	Cry1Ia42,Cry2Aa22,Cry2Ab41,Vip3Aa79,Spp1Aa1	5	Yes	cry1I,cry2A,cry2A,vip3Aa,Spp1A	Coleoptera;Lepidoptera;Rhabditida;Diptera;Hemiptera	*Leptinotarsa decemlineata;Ostrinia furnacalis;Lobesia botrana;Plutella xylostella;Earias insulana;cotton boll*

							;Blattodea;Aedes aegypti	*weevil;Meloidogyne incognita;Helicoverpa armigera;Aedes aegypti;Diaphorina citri;Helicoverpa punctigera;Spodoptera frugiperda;Helicoverpa zea;Chilo partellus;Blattella germanica;Spodoptera litura*
GCF_008829745.1	*Bacillus thuringiensis*	NT06	Cry1Ia42,Cry2Aa22,Cry2Ab41,Vip3Aa79,Spp1Aa1	5	Yes	cry1I,cry2A,cry2A,vip3Aa,Spp1A	Coleoptera;Lepidoptera;Rhabditida;Diptera;Hemiptera;Blattodea;Aedes aegypti	*Leptinotarsa decemlineata;Ostrinia furnacalis;Lobesia botrana;Plutella xylostella;Earias insulana;cotton boll weevil;Meloidogyne incognita;Helicoverpa armigera;Aedes aegypti;Diaphorina citri;Helicoverpa punctigera;Spodoptera frugiperda;Helicoverpa zea;Chilo partellus;Blattella germanica;Spodoptera litura*
GCF_008829485.1	*Bacillus thuringiensis*	TH19	Cry1Ia42,Cry2Aa22,Cry2Ab15,Vip3Aa79,Spp1Aa1	5	Yes	cry1I,cry2A,cry2A,vip3Aa,Spp1A	Coleoptera;Lepidoptera;Rhabditida;Diptera;Hemiptera;Blattodea;Aedes aegypti	*Leptinotarsa decemlineata;Ostrinia furnacalis;Lobesia botrana;Plutella xylostella;Earias insulana;cotton boll weevil;Meloidogyne incognita;Helicoverpa armigera;Aedes aegypti;Diaphorina citri;Helicoverpa punctigera;Spodoptera frugiperda;Helicoverpa zea;Chilo*

								partellus;Blattella germanica;Spodoptera litura
GCF_007829295.1	*Bacillus thuringiensis*	1230	Cry11Aa3,Cry4Ba7,Cry10Aa4,Cyt1Ca1,Cyt1Aa11,Cyt2Ba17,Mpp60Ba3,Mpp60Aa3	8	No	cry11A,cry4B,cry10A,cyt1A,cyt2B,Mpp60B,Mpp60A	Diptera;Hemiptera;Coleoptera;Lepidoptera	*Aedes aegypti;Anopheles albimanus;Culex quinquefasciatu;Culex pipiens;Anopheles gambiae;Diaphorina citri;cotton boll weevil;Culex quinquefasciatus;Culex pipien;Plutella xylostella;Anopheles stephensi;Bradysia difformis*
GCF_007680385.1	*Bacillus thuringiensis*	DE0031	Spp1Aa1	1	No	Spp1A	Lepidoptera;Blattodea	*Spodoptera litura;Blattella germanica*
GCF_007678535.1	*Bacillus thuringiensis*	DE0161	Cry1Ia42,Cry2Aa22,Cry2Ab41,Vip3Aa79,Spp1Aa1	5	Yes	cry1I,cry2A,cry2A,vip3Aa,Spp1A	Coleoptera;Lepidoptera;Rhabditida;Diptera;Hemiptera;Blattodea;Aedes aegypti	*Leptinotarsa decemlineata;Ostrinia furnacalis;Lobesia botrana;Plutella xylostella;Earias insulana;cotton boll weevil;Meloidogyne incognita;Helicoverpa armigera;Aedes aegypti;Diaphorina citri;Helicoverpa punctigera;Spodoptera frugiperda;Helicoverpa zea;Chilo partellus;Blattella germanica;Spodoptera litura*
GCF_007678495.1	*Bacillus thuringiensis*	DE0163	Bmp1-other	1	No			
GCF_007	*Bacillus*	DE017	Spp1Aa1	1	Yes	Spp1A	Lepidoptera;Blatto	*Spodoptera litura;Blattella*

678275.1	*thuringiensis*	7					dea	*germanica*
GCF_007676905.1	*Bacillus thuringiensis*	DE0280	Spp1Aa1	1	Yes	Spp1A	Lepidoptera;Blattodea	*Spodoptera litura;Blattella germanica*
GCF_007674265.1	*Bacillus thuringiensis*	DE0298	Spp1Aa1	1	Yes	Spp1A	Lepidoptera;Blattodea	*Spodoptera litura;Blattella germanica*
GCF_007673915.1	*Bacillus thuringiensis*	DE0326	Spp1Aa1	1	No	Spp1A	Lepidoptera;Blattodea	*Spodoptera litura;Blattella germanica*
GCF_007673835.1	*Bacillus thuringiensis*	DE0332	Spp1Aa1	1	Yes	Spp1A	Lepidoptera;Blattodea	*Spodoptera litura;Blattella germanica*
GCF_007673665.1	*Bacillus thuringiensis*	DE0343			No			
GCF_007673265.1	*Bacillus thuringiensis*	DE0365	Spp1Aa1	1	Yes	Spp1A	Lepidoptera;Blattodea	*Spodoptera litura;Blattella germanica*
GCF_007667455.1	*Bacillus thuringiensis*	DE0472			No			
GCF_007666995.1	*Bacillus thuringiensis*	DE0502	Spp1Aa1	1	Yes	Spp1A	Lepidoptera;Blattodea	*Spodoptera litura;Blattella germanica*
GCF_007	*Bacillus*	DE058	Cry1Ia42,Cry2Aa22,Cr	5	Yes	cry1I,cry2A,cry2A,vip	Coleoptera;Lepido	*Leptinotarsa decemlineata;Ostrinia*

665595.1	*thuringiensis*	9	y2Ab41,Vip3Aa79,Spp1Aa1			3Aa,Spp1A	ptera;Rhabditida;Diptera;Hemiptera;Blattodea;Aedes aegypti	*furnacalis;Lobesia botrana;Plutella xylostella;Earias insulana;cotton boll weevil;Meloidogyne incognita;Helicoverpa armigera;Aedes aegypti;Diaphorina citri;Helicoverpa punctigera;Spodoptera frugiperda;Helicoverpa zea;Chilo partellus;Blattella germanica;Spodoptera litura*
GCF_007665355.1	*Bacillus thuringiensis*	DE0604	App4Aa1,Spp1Aa1	2	No	App4A,Spp1A	Lepidoptera;Hemiptera;Blattodea	*Plutella xylostella;Laodelphax striatellus;Spodoptera litura;Blattella germanica*
GCF_007665345.1	*Bacillus thuringiensis*	DE0606	Spp1Aa1	1	Yes	Spp1A	Lepidoptera;Blattodea	*Spodoptera litura;Blattella germanica*
GCF_005048915.1	*Bacillus thuringiensis*	MW	Spp1Aa1	1	No	Spp1A	Lepidoptera;Blattodea	*Spodoptera litura;Blattella germanica*
GCF_004519705.1	*Bacillus thuringiensis*	DPC6431			No			
GCF_004343085.1	*Bacillus thuringiensis*	LP_2_YM			No			
GCF_004330415.1	*Bacillus thuringiensis*	MS12	Cry1Ia42,Cry2Ab41,Cry9Ea12,Cry1Ca18,Cry1Da2,Cry1Ab9,Vip3Aa7	8	Yes	cry1I,cry2A,Cry9E,cry1C,cry1D,cry1A,vip3Aa,Spp1A	Coleoptera;Lepidoptera;Rhabditida;Diptera;Hemiptera	*Leptinotarsa decemlineata;Ostrinia furnacalis;Lobesia botrana;Plutella xylostella;Earias insulana;cotton boll*

			9,Spp1Aa1				;Dipteran;Blattodea;Aedes aegypti	*weevil;Meloidogyne incognita;Helicoverpa armigera;Aedes aegypti;Diaphorina citri;Agrotis exclamationis;Cydia pomonella;Myzus persicae;Ostrinia nubilalis;Spodoptera exigua;Spodoptera frugiperda;Bombyx mori;Spodoptera litura;Epiphyas postvittana;Leucinodes orbonalis;Trichoplusia ni;Hyphantria cunea;Pieris rapae;Chilo suppressalis;Helicoverpa punctigera;Helicoverpa zea;Chilo partellus;Blattella germanica*
GCF_004309715.1	*Bacillus thuringiensis*	T26			No			
GCF_004194475.2	*Bacillus thuringiensis*	T0139	Cry11Aa3,Cry10Aa4,Cry4Ba7,Cry4Aa5,Cyt1Ca1,Cyt1Aa11,Cyt2Ba17	7	No	cry11A,cry10A,cry4B,cry4A,cyt1A,cyt2B	Diptera;Coleoptera;Hemiptera;Lepidoptera	*Aedes aegypti;Anopheles albimanus;Culex quinquefasciatu;Culex pipiens;cotton boll weevil;Anopheles gambiae;Diaphorina citri;Culex quinquefasciatus;Acyrthosiphon pisum;Culex pipien;Plutella xylostella;Anopheles stephensi;Bradysia difformis*
GCF_004	*Bacillus*	T0131	Cry11Aa3,Cry10Aa4,C	7	No	cry11A,cry10A,cry4B	Diptera;Coleopter	*Aedes aegypti;Anopheles*

194435.2	*thuringiensis*		ry4Ba7,Cry4Aa5,Cyt1Ca1,Cyt1Aa11,Cyt2Ba17			,cry4A,cyt1A,cyt2B	a;Hemiptera;Lepidoptera	*albimanus;Culex quinquefasciatu;Culex pipiens;cotton boll weevil;Anopheles gambiae;Diaphorina citri;Culex quinquefasciatus;Acyrthosiphon pisum;Culex pipien;Plutella xylostella;Anopheles stephensi;Bradysia difformis*
GCF_004167115.1	*Bacillus thuringiensis*	T414	Cry1Ia42,Cry2Aa22,Cry2Ab41,Vip3Aa79,Spp1Aa1	5	Yes	cry1I,cry2A,cry2A,vip3Aa,Spp1A	Coleoptera;Lepidoptera;Rhabditida;Diptera;Hemiptera;Blattodea;Aedes aegypti	*Leptinotarsa decemlineata;Ostrinia furnacalis;Lobesia botrana;Plutella xylostella;Earias insulana;cotton boll weevil;Meloidogyne incognita;Helicoverpa armigera;Aedes aegypti;Diaphorina citri;Helicoverpa punctigera;Spodoptera frugiperda;Helicoverpa zea;Chilo partellus;Blattella germanica;Spodoptera litura*
GCF_003991175.1	*Bacillus thuringiensis*	C15	Cry21Aa3,Spp1Aa1	2	No	Cry21A,Spp1A	Rhabditida;Lepidoptera;Blattodea	*Heligmosomoides polygyrus bakeri;Caenorhabditis elegans;Spodoptera litura;Blattella germanica*
GCF_003719805.2	*Bacillus thuringiensis*	INTA Mo4-4	Cry3Aa6,Mpp23Aa1,Spp1Aa1,Vpa2Ah1,Vpb1Ae1,Xpp37Aa1	6	No	Mpp23A+Xpp37A,Vpa2Ah + Vpb1Ae,Cry3A,Spp1A	Coleoptera;Hemiptera;Lepidoptera;Blattodea	*Cylas puncticollis;Anthonomus grandis;Aphis gossypii;Leptinotarsa decemlineata;Tenebrio molitor;Hypera postica;Macrosiphum*

								euphorbiae;Spodoptera litura;Blattella germanica
GCF_003626955.1	*Bacillus thuringiensis*	QZL38	Cry8Ea5,Cry8Fa1,Cry8Ca5,Mpp5Ab1	4	No	cry8E,cry8F,cry8C,Mpp5A	Coleoptera	*Holotrichia parallela;Anomala corpulenta;H. oblita;Phyllotreta striolata;Anomala cuprea;Anomala rufocuprea;Popillia japonica;Alphitobius diaperinus;Anomala exoleta;Colaphellus bowringi;Leptinotarsa decemlineata*
GCF_003445395.2	*Bacillus thuringiensis serovar israelensis*	AM65-52	Cry11Aa3,Cry10Aa4,Cry4Aa5,Cyt1Ca1,Cyt1Aa11,Cyt2Ba17,Mpp60Ba3,Mpp60Aa3	8	No	cry11A,cry10A,cry4A,cyt1A,cyt2B,Mpp60B,Mpp60A	Diptera;Coleoptera;Hemiptera;Lepidoptera	*Aedes aegypti;Anopheles albimanus;Culex quinquefasciatu;Culex pipiens;cotton boll weevil;Culex quinquefasciatus;Acyrthosiphon pisum;Culex pipien;Diaphorina citri;Plutella xylostella;Anopheles stephensi;Anopheles gambiae;Bradysia difformis*
GCF_003444735.1	*Bacillus thuringiensis*	BLB406	Spp1Aa1,Xpp22Aa3	2	Yes	Spp1A,Xpp22A	Lepidoptera;Blattodea;Coleoptera	*Spodoptera litura;Blattella germanica;Anthonomus grandis*
GCF_003408695.1	*Bacillus thuringiensis*	IPPBIOTSUC-C9F1	Cry8Ma2,Cry8Ha1,Tpp78Aa1	3	No	cry8H,Tpp78A	Coleoptera;Hemiptera;Neuroptera	*Holotrichia parallela;Holotrichia oblita;Anoplophora glabripennis;Laodelphax striatellus;Bemisia tabaci;Diaphorina citri;Chrysoperla nipponensis*

GCF_003399465.1	*Bacillus thuringiensis*	BAM	App4Aa1,Spp1Aa1	2	Yes	App4A,Spp1A	Lepidoptera;Hemiptera;Blattodea	*Plutella xylostella;Laodelphax striatellus;Spodoptera litura;Blattella germanica*
GCF_003312445.1	*Bacillus thuringiensis*	kn11	Cry1Ia42,Cry2Ab41,Cry9Ea12,Cry1Ca18,Vip3Aa79,Spp1Aa1	6	Yes	cry1I,cry2A,Cry9E,cry1C,vip3Aa,Spp1A	Coleoptera;Lepidoptera;Rhabditida;Diptera;Hemiptera;Dipteran;Blattodea;Aedes aegypti	*Leptinotarsa decemlineata;Ostrinia furnacalis;Lobesia botrana;Plutella xylostella;Earias insulana;cotton boll weevil;Meloidogyne incognita;Helicoverpa armigera;Aedes aegypti;Diaphorina citri;Agrotis exclamationis;Cydia pomonella;Myzus persicae;Helicoverpa punctigera;Spodoptera frugiperda;Helicoverpa zea;Chilo partellus;Blattella germanica;Spodoptera litura*
GCF_003112375.2	*Bacillus thuringiensis*	m401	Spp1Aa1	1	Yes	Spp1A	Lepidoptera;Blattodea	*Spodoptera litura;Blattella germanica*
GCF_003054785.2	*Bacillus thuringiensis*	BT62	Cry8Sa1,Cry8Ib2,Spp1Aa1	3	Yes	cry8S,cry8I,Spp1A	Coleoptera;Lepidoptera;Blattodea	*Holotrichia serrata;Holotrichia parallela;Spodoptera litura;Blattella germanica*
GCF_003046465.1	*Bacillus thuringiensis*	Bt-ICA/UNMSM10RA	Cry1Ac9,Spp1Aa1	2	Yes	cry1A,Spp1A	Lepidoptera;Blattodea	*Plutella xylostella;Ostrinia furnacalis;Helicoverpa armigera;Leucinodes orbonalis;Trichoplusia ni;Hyphantria cunea;Spodoptera frugiperda;Pieris rapae;Chilo suppressalis;Spodoptera*

								litura;Blattella germanica
GCF_002998855.1	*Bacillus thuringiensis*	GBAC46	Spp1Aa1	1	No	Spp1A	Lepidoptera;Blattodea	*Spodoptera litura;Blattella germanica*
GCF_002969075.2	*Bacillus thuringiensis*	BA04	Cry1Ib1,Spp1Aa1	2	Yes	cry1I,Spp1A	Coleoptera;Lepidoptera;Rhabditida;Blattodea	*Leptinotarsa decemlineata;Ostrinia furnacalis;Lobesia botrana;Plutella xylostella;Earias insulana;cotton boll weevil;Meloidogyne incognita;Spodoptera litura;Blattella germanica*
GCF_002921215.1	*Bacillus thuringiensis*	BR145	Cry1Ia42,Cry2Aa22,Cry2Ab41,Vip3Aa79,Spp1Aa1	5	Yes	cry1I,cry2A,cry2A,vip3Aa,Spp1A	Coleoptera;Lepidoptera;Rhabditida;Diptera;Hemiptera;Blattodea;Aedes aegypti	*Leptinotarsa decemlineata;Ostrinia furnacalis;Lobesia botrana;Plutella xylostella;Earias insulana;cotton boll weevil;Meloidogyne incognita;Helicoverpa armigera;Aedes aegypti;Diaphorina citri;Helicoverpa punctigera;Spodoptera frugiperda;Helicoverpa zea;Chilo partellus;Blattella germanica;Spodoptera litura*
GCF_002912215.1	*Bacillus thuringiensis serovar kurstaki*	BGSC 4D4	Cry1Ac9,Spp1Aa1	2	Yes	cry1A,Spp1A	Lepidoptera;Blattodea	*Plutella xylostella;Ostrinia furnacalis;Helicoverpa armigera;Leucinodes orbonalis;Trichoplusia ni;Hyphantria cunea;Spodoptera frugiperda;Pieris rapae;Chilo suppressalis;Spodoptera litura;Blattella germanica*

GCF_002912105.1	*Bacillus thuringiensis serovar kurstaki*	7.1o	Cry1Ab9,Cry1Ia42,Cry2Aa22,Cry2Ab41,Vip3Aa79,Spp1Aa1	6	Yes	cry1A,cry1I,cry2A,cry2A,vip3Aa,Spp1A	Lepidoptera;Coleoptera;Rhabditida;Diptera;Hemiptera;Blattodea;Aedes aegypti	*Plutella xylostella;Ostrinia furnacalis;Helicoverpa armigera;Leucinodes orbonalis;Trichoplusia ni;Hyphantria cunea;Spodoptera frugiperda;Pieris rapae;Chilo suppressalis;Leptinotarsa decemlineata;Lobesia botrana;Earias insulana;cotton boll weevil;Meloidogyne incognita;Aedes aegypti;Diaphorina citri;Helicoverpa punctigera;Helicoverpa zea;Chilo partellus;Blattella germanica;Spodoptera litura*
GCF_002911965.1	*Bacillus thuringiensis serovar alesti*	BGSC 4C3	Spp1Aa1	1	No	Spp1A	Lepidoptera;Blattodea	*Spodoptera litura;Blattella germanica*
GCF_002911925.1	*Bacillus thuringiensis serovar galleriae*	BGSC 4G4	Cry1Bd1,Cry1Cb2,Cry1Ga1,Cry1Ib3,Cry1Db1,Cry1Fb5,Cry2Ac6,Cry2Ab4,Cry1Gb2,Cry1Ab35,Cry2Ad1,Vip3Af3,Spp1Aa1,Vpa2Ae3,Vpb1Ac1	15	Yes	Vpa2Ae + Vpb1Ac,cry1B,cry1C,cry1G,cry1I,cry1D,cry1F,cry2A,cry2A,cry1G,cry1A,cry2A,vip3Af,Spp1A	Hemiptera;Lepidoptera;Diptera;Coleoptera;Dipteran;Rhabditida;Blattodea	*Aphis gossypii;Plutella xylostella;Ostrinia nubilalis;Spodoptera frugiperda;Maruca vitrata;Manduca sexta;cotton boll weevil;Myzus persicae;Aedes aegypti;Pieris rapae;Thaumetopoea pityocampa;Leptinotarsa decemlineata;Ostrinia*

								furnacalis;Lobesia botrana;Earias insulana;Meloidogyne incognita;Spodoptera exigua;Bombyx mori;Spodoptera litura;Epiphyas postvittana;Agrotis ipsilon;Diabrotica virgifera virgifera;Helicoverpa armigera;Diaphorina citri;Leucinodes orbonalis;Trichoplusia ni;Hyphantria cunea;Chilo suppressalis;Blattella germanica
GCF_002911865.1	*Bacillus thuringiensis serovar kurstaki*	BGSC 4D1	Cry1Ia42,Cry2Aa22,Cry2Ab41,Vip3Aa79,Spp1Aa1	5	Yes	cry1I,cry2A,cry2A,vip3Aa,Spp1A	Coleoptera;Lepidoptera;Rhabditida;Diptera;Hemiptera;Blattodea;Aedes aegypti	*Leptinotarsa decemlineata;Ostrinia furnacalis;Lobesia botrana;Plutella xylostella;Earias insulana;cotton boll weevil;Meloidogyne incognita;Helicoverpa armigera;Aedes aegypti;Diaphorina citri;Helicoverpa punctigera;Spodoptera frugiperda;Helicoverpa zea;Chilo partellus;Blattella germanica;Spodoptera litura*
GCF_002911825.1	*Bacillus thuringiensis serovar sotto*	BGSC 4E3	Cry1Aa9,Spp1Aa1	2	Yes	cry1A,Spp1A	Lepidoptera;Blattodea	*Plutella xylostella;Ostrinia furnacalis;Helicoverpa armigera;Leucinodes orbonalis;Trichoplusia ni;Hyphantria cunea;Spodoptera frugiperda;Pieris*

								rapae;Chilo suppressalis;Spodoptera litura;Blattella germanica
GCF_002911795.1	*Bacillus thuringiensis serovar medellin*	NRRL 23135	Cry30Aa1,Cry29Aa1,Cry11Bb1,Cyt1Ca1,Cyt2Bc1,Cyt1Ab1,Spp1Aa1	7	Yes	Cry11B,cyt2B,cyt1A,Spp1A	Diptera;Hemiptera;Coleoptera;Lepidoptera;Blattodea	*Aedes aegypti;Anopheles albimanus;Culex quinquefasciatus;Anopheles stephensi;Anopheles gambiae;Culex pipiens;Bradysia difformis;Culex pipien;Diaphorina citri;Plutella xylostella;Spodoptera litura;Blattella germanica*
GCF_002911785.1	*Bacillus thuringiensis serovar japonensis*	strain 62	Cry8Sa1,Cry8Ib2,Gpp34Aa4,Mpp38Aa1,Spp1Aa1,Tpp35Aa4	6	Yes	Gpp34A+Tpp35A,cry8S,cry8I,Spp1A	Coleoptera;Lepidoptera;Blattodea	*Diabrotica virgifera virgifera;Diabrotica barberi;Sitotroga cerealella;Diabrotica undecimpunctata;Holotrichia serrata;Holotrichia parallela;Spodoptera litura;Blattella germanica*
GCF_002911755.1	*Bacillus thuringiensis serovar thuringiensis*	BGSC 4A3	Cry1Ba8,Cry1Ab9,Mpp5Ab1,Spp1Aa1,Vpa2Bb4,Vpb1Bc1	6	Yes	cry1B,cry1A,Mpp5A,Spp1A	Lepidoptera;Hemiptera;Diptera;Coleoptera;Blattodea	*Plutella xylostella;Ostrinia nubilalis;Spodoptera frugiperda;Maruca vitrata;Manduca sexta;cotton boll weevil;Ostrinia furnacalis;Helicoverpa armigera;Leucinodes orbonalis;Trichoplusia ni;Hyphantria cunea;Pieris rapae;Chilo suppressalis;Colaphellus bowringi;Leptinotarsa*

								decemlineata;Spodoptera litura;Blattella germanica
GCF_002911745.1	*Bacillus thuringiensis serovar japonensis*	Buibui	Cry8Ca1,Spp1Aa1	2	No	cry8C,Spp1A	Coleoptera;Lepidoptera;Blattodea	*Holotrichia parallela;Anomala cuprea;Anomala rufocuprea;Popillia japonica;Alphitobius diaperinus;Anomala corpulenta;Anomala exoleta;Colaphellus bowringi;Spodoptera litura;Blattella germanica*
GCF_002893585.1	*Bacillus thuringiensis*	HBF-18	Cry8Ga3,Spp1Aa1,Vpa2Ag1,Vpb1Ad1	4	Yes	Vpa2Ag + Vpb1Ad,cry8G,Spp1A	Coleoptera;Lepidoptera;Blattodea	*Anomala corpulenta;Holotrichia oblita;Holotrichia parallela;Phyllotreta striolata;Spodoptera litura;Blattella germanica*
GCF_002893515.1	*Bacillus thuringiensis*	78-2	Cry8Ca5,Mpp5Ab1	2	No	cry8C,Mpp5A	Coleoptera	*Holotrichia parallela;Anomala cuprea;Anomala rufocuprea;Popillia japonica;Alphitobius diaperinus;Anomala corpulenta;Anomala exoleta;Colaphellus bowringi;Leptinotarsa decemlineata*
GCF_002893505.1	*Bacillus thuringiensis*	Fcd114	Cry8Ga3,Spp1Aa1,Vpa2Ag1,Vpb1Ad1	4	Yes	Vpa2Ag + Vpb1Ad,cry8G,Spp1A	Coleoptera;Lepidoptera;Blattodea	*Anomala corpulenta;Holotrichia oblita;Holotrichia parallela;Phyllotreta striolata;Spodoptera litura;Blattella germanica*
GCF_002	*Bacillus*	AFS03	Cry1Ia42,Cry2Aa22,Cr	5	Yes	cry1I,cry2A,cry2A,vip	Coleoptera;Lepido	*Leptinotarsa decemlineata;Ostrinia*

589045.1	*thuringiensis*	4084	y2Ab41,Vip3Aa79,Spp1Aa1			3Aa,Spp1A	ptera;Rhabditida;Diptera;Hemiptera;Blattodea;Aedes aegypti	*furnacalis;Lobesia botrana;Plutella xylostella;Earias insulana;cotton boll weevil;Meloidogyne incognita;Helicoverpa armigera;Aedes aegypti;Diaphorina citri;Helicoverpa punctigera;Spodoptera frugiperda;Helicoverpa zea;Chilo partellus;Blattella germanica;Spodoptera litura*
GCF_002589015.1	*Bacillus thuringiensis*	AFS031473	Cry1Ia42,Cry2Aa22,Cry2Ab41,Vip3Aa79,Spp1Aa1	5	Yes	cry1I,cry2A,cry2A,vip3Aa,Spp1A	Coleoptera;Lepidoptera;Rhabditida;Diptera;Hemiptera;Blattodea;Aedes aegypti	*Leptinotarsa decemlineata;Ostrinia furnacalis;Lobesia botrana;Plutella xylostella;Earias insulana;cotton boll weevil;Meloidogyne incognita;Helicoverpa armigera;Aedes aegypti;Diaphorina citri;Helicoverpa punctigera;Spodoptera frugiperda;Helicoverpa zea;Chilo partellus;Blattella germanica;Spodoptera litura*
GCF_002586085.1	*Bacillus thuringiensis*	AFS030866	Cry68Aa1,Cry70Ba1,Spp1Aa1	3	Yes	Spp1A	Lepidoptera;Blattodea	*Spodoptera litura;Blattella germanica*
GCF_002585935.1	*Bacillus thuringiensis*	AFS031210	Spp1Aa1	1	Yes	Spp1A	Lepidoptera;Blattodea	*Spodoptera litura;Blattella germanica*
GCF_002	*Bacillus*	AFS03	App4Aa1,Spp1Aa1	2	Yes	App4A,Spp1A	Lepidoptera;Hemi	*Plutella xylostella;Laodelphax*

585795.1	*thuringiensis*	1814					ptera;Blattodea	*striatellus;Spodoptera litura;Blattella germanica*
GCF_002585635.1	*Bacillus thuringiensis*	AFS034546	Spp1Aa1	1	Yes	Spp1A	Lepidoptera;Blattodea	*Spodoptera litura;Blattella germanica*
GCF_002585315.1	*Bacillus thuringiensis*	AFS036383	App4Aa1,Spp1Aa1	2	Yes	App4A,Spp1A	Lepidoptera;Hemiptera;Blattodea	*Plutella xylostella;Laodelphax striatellus;Spodoptera litura;Blattella germanica*
GCF_002584925.1	*Bacillus thuringiensis*	AFS029767	Spp1Aa1	1	Yes	Spp1A	Lepidoptera;Blattodea	*Spodoptera litura;Blattella germanica*
GCF_002584205.1	*Bacillus thuringiensis*	AFS034708	Spp1Aa1	1	Yes	Spp1A	Lepidoptera;Blattodea	*Spodoptera litura;Blattella germanica*
GCF_002583895.1	*Bacillus thuringiensis*	AFS036350	Spp1Aa1	1	Yes	Spp1A	Lepidoptera;Blattodea	*Spodoptera litura;Blattella germanica*
GCF_002583105.1	*Bacillus thuringiensis*	AFS032723	Spp1Aa1,Tpp80Ad1	2	No	Spp1A,Tpp80A	Lepidoptera;Blattodea;Diptera;Hemiptera	*Spodoptera litura;Blattella germanica;Culex pipiens pallens;Culex quinquefasciatus;Aedes aegypti;Anopheles gambiae;Laodelphax striatellus*
GCF_002581075.1	*Bacillus thuringiensis*	AFS043930	Cry1Ia42,Cry2Aa22,Cry2Ab41,Vip3Aa79,Spp1Aa1	5	Yes	cry1I,cry2A,cry2A,vip3Aa,Spp1A	Coleoptera;Lepidoptera;Rhabditida;Diptera;Hemiptera;Blattodea;Aedes aegypti	*Leptinotarsa decemlineata;Ostrinia furnacalis;Lobesia botrana;Plutella xylostella;Earias insulana;cotton boll weevil;Meloidogyne incognita;Helicoverpa*

								armigera;Aedes aegypti;Diaphorina citri;Helicoverpa punctigera;Spodoptera frugiperda;Helicoverpa zea;Chilo partellus;Blattella germanica;Spodoptera litura
GCF_002577565.1	*Bacillus thuringiensis*	AFS040438	Tpp80Ad1,Xpp22Ba1	2	No	Tpp80A,Xpp22B	Diptera;Hemiptera;Coleoptera;Lepidoptera	*Culex pipiens pallens;Culex quinquefasciatus;Aedes aegypti;Anopheles gambiae;Laodelphax striatellus;Anthonomus grandis;Plutella xylostella*
GCF_002577445.1	*Bacillus thuringiensis*	AFS040987	App4Aa1,Spp1Aa1	2	No	App4A,Spp1A	Lepidoptera;Hemiptera;Blattodea	*Plutella xylostella;Laodelphax striatellus;Spodoptera litura;Blattella germanica*
GCF_002576755.1	*Bacillus thuringiensis*	AFS047607	Spp1Aa1	1	No	Spp1A	Lepidoptera;Blattodea	*Spodoptera litura;Blattella germanica*
GCF_002576655.1	*Bacillus thuringiensis*	AFS048789	Mpp5Ab1,Spp1Aa1,Vpa2Bb4	3	Yes	Mpp5A,Spp1A	Coleoptera;Lepidoptera;Blattodea	*Colaphellus bowringi;Leptinotarsa decemlineata;Spodoptera litura;Blattella germanica*
GCF_002576585.1	*Bacillus thuringiensis*	AFS050081	Cry5Ba1,Cry5Aa1,Spp1Aa1	3	Yes	cry5B,cry5A,Spp1A	Rhabditida;Lepidoptera;Hymenoptera;Blattodea	*Caenorhabditis elegans;Ancylostoma ceylanicum;Heligmosomoides polygyrus bakeri;Meloidogyne incognita;Haemonchus contortus;Trichostrongylus colubriformis;Ostertagia circumcincta;Diprion pini;Cephacia*

								abietis;Helicoverpa armigera;Spodoptera litura;Blattella germanica
GCF_002576055.1	*Bacillus thuringiensis*	AFS046081	Spp1Aa1	1	No	Spp1A	Lepidoptera;Blattodea	*Spodoptera litura;Blattella germanica*
GCF_002575925.1	*Bacillus thuringiensis*	AFS047635	Mpp5Ab1,Spp1Aa1,Vpa2Bb4	3	Yes	Mpp5A,Spp1A	Coleoptera;Lepidoptera;Blattodea	*Colaphellus bowringi;Leptinotarsa decemlineata;Spodoptera litura;Blattella germanica*
GCF_002575705.1	*Bacillus thuringiensis*	AFS049207	Spp1Aa1	1	No	Spp1A	Lepidoptera;Blattodea	*Spodoptera litura;Blattella germanica*
GCF_002575645.1	*Bacillus thuringiensis*	AFS050550	Spp1Aa1,Tpp80Ad1	2	No	Spp1A,Tpp80A	Lepidoptera;Blattodea;Diptera;Hemiptera	*Spodoptera litura;Blattella germanica;Culex pipiens pallens;Culex quinquefasciatus;Aedes aegypti;Anopheles gambiae;Laodelphax striatellus*
GCF_002575195.1	*Bacillus thuringiensis*	AFS053253	App4Aa1,Spp1Aa1	2	No	App4A,Spp1A	Lepidoptera;Hemiptera;Blattodea	*Plutella xylostella;Laodelphax striatellus;Spodoptera litura;Blattella germanica*
GCF_002575015.1	*Bacillus thuringiensis*	AFS054136	Spp1Aa1,Tpp80Ad1	2	No	Spp1A,Tpp80A	Lepidoptera;Blattodea;Diptera;Hemiptera	*Spodoptera litura;Blattella germanica;Culex pipiens pallens;Culex quinquefasciatus;Aedes aegypti;Anopheles gambiae;Laodelphax striatellus*
GCF_002	*Bacillus*	AFS05	Spp1Aa1	1	No	Spp1A	Lepidoptera;Blatto	*Spodoptera litura;Blattella*

574725.1	*thuringiensis*	4059					dea	*germanica*
GCF_002574695.1	*Bacillus thuringiensis*	AFS057784			No			
GCF_002574505.1	*Bacillus thuringiensis*	AFS055470	Spp1Aa1,Tpp80Ad1	2	No	Spp1A,Tpp80A	Lepidoptera;Blattodea;Diptera;Hemiptera	*Spodoptera litura;Blattella germanica;Culex pipiens pallens;Culex quinquefasciatus;Aedes aegypti;Anopheles gambiae;Laodelphax striatellus*
GCF_002574475.1	*Bacillus thuringiensis*	AFS055708	App4Aa1,Spp1Aa1	2	Yes	App4A,Spp1A	Lepidoptera;Hemiptera;Blattodea	*Plutella xylostella;Laodelphax striatellus;Spodoptera litura;Blattella germanica*
GCF_002574125.1	*Bacillus thuringiensis*	AFS058254	Spp1Aa1	1	No	Spp1A	Lepidoptera;Blattodea	*Spodoptera litura;Blattella germanica*
GCF_002574115.1	*Bacillus thuringiensis*	AFS057829	Cry1Ba8,Cry1Aa3,Cry1Ia42,Cry2Aa22,Cry2Ab41,Vip3Aa79,Spp1Aa1	7	Yes	cry1B,cry1A,cry1I,cry2A,cry2A,vip3Aa,Spp1A	Lepidoptera;Hemiptera;Diptera;Coleoptera;Rhabditida;Blattodea;Aedes aegypti	*Plutella xylostella;Ostrinia nubilalis;Spodoptera frugiperda;Maruca vitrata;Manduca sexta;cotton boll weevil;Ostrinia furnacalis;Helicoverpa armigera;Leucinodes orbonalis;Trichoplusia ni;Hyphantria cunea;Pieris rapae;Chilo suppressalis;Leptinotarsa decemlineata;Lobesia botrana;Earias insulana;Meloidogyne*

								incognita;Aedes aegypti;Diaphorina citri;Helicoverpa punctigera;Helicoverpa zea;Chilo partellus;Blattella germanica;Spodoptera litura
GCF_002572135.1	*Bacillus thuringiensis*	AFS061780	Cry1Ia42,Cry2Aa22,Cry2Ab41,Vip3Aa79,Spp1Aa1	5	Yes	cry1I,cry2A,cry2A,vip3Aa,Spp1A	Coleoptera;Lepidoptera;Rhabditida;Diptera;Hemiptera;Blattodea;Aedes aegypti	*Leptinotarsa decemlineata;Ostrinia furnacalis;Lobesia botrana;Plutella xylostella;Earias insulana;cotton boll weevil;Meloidogyne incognita;Helicoverpa armigera;Aedes aegypti;Diaphorina citri;Helicoverpa punctigera;Spodoptera frugiperda;Helicoverpa zea;Chilo partellus;Blattella germanica;Spodoptera litura*
GCF_002570585.1	*Bacillus thuringiensis*	AFS059914	App4Aa1,Spp1Aa1,Tpp80Ad1	3	Yes	App4A,Spp1A,Tpp80A	Lepidoptera;Hemiptera;Blattodea;Diptera	*Plutella xylostella;Laodelphax striatellus;Spodoptera litura;Blattella germanica;Culex pipiens pallens;Culex quinquefasciatus;Aedes aegypti;Anopheles gambiae*
GCF_002570475.1	*Bacillus thuringiensis*	AFS061760	Spp1Aa1,Tpp80Ad1	2	No	Spp1A,Tpp80A	Lepidoptera;Blattodea;Diptera;Hemiptera	*Spodoptera litura;Blattella germanica;Culex pipiens pallens;Culex quinquefasciatus;Aedes aegypti;Anopheles gambiae;Laodelphax striatellus*

GCF_002569275.1	*Bacillus thuringiensis*	AFS075487			Yes			
GCF_002568525.1	*Bacillus thuringiensis*	AFS065825	Spp1Aa1,Tpp80Ad1	2	No	Spp1A,Tpp80A	Lepidoptera;Blattodea;Diptera;Hemiptera	*Spodoptera litura;Blattella germanica;Culex pipiens pallens;Culex quinquefasciatus;Aedes aegypti;Anopheles gambiae;Laodelphax striatellus*
GCF_002568515.1	*Bacillus thuringiensis*	AFS066836	Mpp5Ab1,Spp1Aa1,Vpa2Bb4	3	Yes	Mpp5A,Spp1A	Coleoptera;Lepidoptera;Blattodea	*Colaphellus bowringi;Leptinotarsa decemlineata;Spodoptera litura;Blattella germanica*
GCF_002568045.1	*Bacillus thuringiensis*	AFS065753			Yes			
GCF_002567995.1	*Bacillus thuringiensis*	AFS066175	App4Aa1,Spp1Aa1	2	Yes	App4A,Spp1A	Lepidoptera;Hemiptera;Blattodea	*Plutella xylostella;Laodelphax striatellus;Spodoptera litura;Blattella germanica*
GCF_002567945.1	*Bacillus thuringiensis*	AFS066485	Spp1Aa1,Tpp80Ad1	2	No	Spp1A,Tpp80A	Lepidoptera;Blattodea;Diptera;Hemiptera	*Spodoptera litura;Blattella germanica;Culex pipiens pallens;Culex quinquefasciatus;Aedes aegypti;Anopheles gambiae;Laodelphax striatellus*
GCF_002567875.1	*Bacillus thuringiensis*	AFS066710	Mpp5Ab1,Spp1Aa1,Vpa2Bb4	3	Yes	Mpp5A,Spp1A	Coleoptera;Lepidoptera;Blattodea	*Colaphellus bowringi;Leptinotarsa decemlineata;Spodoptera litura;Blattella germanica*
GCF_002	*Bacillus*	AFS07	Spp1Aa1	1	No	Spp1A	Lepidoptera;Blatto	*Spodoptera litura;Blattella*

567585.1	*thuringiensis*	0359					dea	*germanica*
GCF_002566645.1	*Bacillus thuringiensis*	AFS076262	App4Aa1,Spp1Aa1	2	No	App4A,Spp1A	Lepidoptera;Hemiptera;Blattodea	*Plutella xylostella;Laodelphax striatellus;Spodoptera litura;Blattella germanica*
GCF_002566545.1	*Bacillus thuringiensis*	AFS078332			Yes			
GCF_002565905.1	*Bacillus thuringiensis*	AFS076260			Yes			
GCF_002565195.1	*Bacillus thuringiensis*	AFS075396	Spp1Aa1,Tpp80Ad1	2	No	Spp1A,Tpp80A	Lepidoptera;Blattodea;Diptera;Hemiptera	*Spodoptera litura;Blattella germanica;Culex pipiens pallens;Culex quinquefasciatus;Aedes aegypti;Anopheles gambiae;Laodelphax striatellus*
GCF_002564505.1	*Bacillus thuringiensis*	AFS088016	Cry68Aa1,Cry70Ba1,Spp1Aa1	3	Yes	Spp1A	Lepidoptera;Blattodea	*Spodoptera litura;Blattella germanica*
GCF_002562915.1	*Bacillus thuringiensis*	AFS029508	Spp1Aa1	1	No	Spp1A	Lepidoptera;Blattodea	*Spodoptera litura;Blattella germanica*
GCF_002562665.1	*Bacillus thuringiensis*	AFS007900	App4Aa1,Spp1Aa1	2	No	App4A,Spp1A	Lepidoptera;Hemiptera;Blattodea	*Plutella xylostella;Laodelphax striatellus;Spodoptera litura;Blattella germanica*
GCF_002	*Bacillus*	AFS01	App4Aa1,Spp1Aa1	2	No	App4A,Spp1A	Lepidoptera;Hemi	*Plutella xylostella;Laodelphax*

561175.1	*thuringiensis*	7371					ptera;Blattodea	*striatellus;Spodoptera litura;Blattella germanica*
GCF_002560815.1	*Bacillus thuringiensis*	AFS022064	Spp1Aa1,Tpp80Ad1	2	No	Spp1A,Tpp80A	Lepidoptera;Blattodea;Diptera;Hemiptera	*Spodoptera litura;Blattella germanica;Culex pipiens pallens;Culex quinquefasciatus;Aedes aegypti;Anopheles gambiae;Laodelphax striatellus*
GCF_002560695.1	*Bacillus thuringiensis*	AFS022504	Cry5Ba1,Cry5Aa1,Spp1Aa1	3	Yes	cry5B,cry5A,Spp1A	Rhabditida;Lepidoptera;Hymenoptera;Blattodea	*Caenorhabditis elegans;Ancylostoma ceylanicum;Heligmosomoides polygyrus bakeri;Meloidogyne incognita;Haemonchus contortus;Trichostrongylus colubriformis;Ostertagia circumcincta;Diprion pini;Cephacia abietis;Helicoverpa armigera;Spodoptera litura;Blattella germanica*
GCF_002559535.1	*Bacillus thuringiensis*	AFS000517	Spp1Aa1,Tpp80Ad1	2	No	Spp1A,Tpp80A	Lepidoptera;Blattodea;Diptera;Hemiptera	*Spodoptera litura;Blattella germanica;Culex pipiens pallens;Culex quinquefasciatus;Aedes aegypti;Anopheles gambiae;Laodelphax striatellus*
GCF_002558525.1	*Bacillus thuringiensis*	AFS005140	Cry68Aa1,Cry70Ba1,Spp1Aa1	3	Yes	Spp1A	Lepidoptera;Blattodea	*Spodoptera litura;Blattella germanica*
GCF_002	*Bacillus*	AFS01	Cry68Aa1,Cry70Ba1,S	3	Yes	Spp1A	Lepidoptera;Blatto	*Spodoptera litura;Blattella*

558015.1	*thuringiensis*	3213	pp1Aa1				dea	*germanica*
GCF_002555365.1	*Bacillus thuringiensis*	AFS018409	Cry1Ia42,Cry2Aa22,Cry2Ab41,Vip3Aa79,Spp1Aa1	5	Yes	cry1I,cry2A,cry2A,vip3Aa,Spp1A	Coleoptera;Lepidoptera;Rhabditida;Diptera;Hemiptera;Blattodea;Aedes aegypti	*Leptinotarsa decemlineata;Ostrinia furnacalis;Lobesia botrana;Plutella xylostella;Earias insulana;cotton boll weevil;Meloidogyne incognita;Helicoverpa armigera;Aedes aegypti;Diaphorina citri;Helicoverpa punctigera;Spodoptera frugiperda;Helicoverpa zea;Chilo partellus;Blattella germanica;Spodoptera litura*
GCF_002553105.1	*Bacillus thuringiensis*	AFS094730	Spp1Aa1,Tpp80Ad1	2	No	Spp1A,Tpp80A	Lepidoptera;Blattodea;Diptera;Hemiptera	*Spodoptera litura;Blattella germanica;Culex pipiens pallens;Culex quinquefasciatus;Aedes aegypti;Anopheles gambiae;Laodelphax striatellus*
GCF_002553035.1	*Bacillus thuringiensis*	AFS096978	App4Aa1,Spp1Aa1,Tpp80Ad1,Xpp22Ba1	4	Yes	App4A,Spp1A,Tpp80A,Xpp22B	Lepidoptera;Hemiptera;Blattodea;Diptera;Coleoptera	*Plutella xylostella;Laodelphax striatellus;Spodoptera litura;Blattella germanica;Culex pipiens pallens;Culex quinquefasciatus;Aedes aegypti;Anopheles gambiae;Anthonomus grandis*
GCF_002552415.1	*Bacillus thuringi*	AFS094706			Yes			

	ensis							
GCF_002552325.1	*Bacillus thuringiensis*	AFS095482	Spp1Aa1	1	No	Spp1A	Lepidoptera;Blattodea	*Spodoptera litura;Blattella germanica*
GCF_002551885.1	*Bacillus thuringiensis*	AFS097917	App4Aa1,Spp1Aa1	2	Yes	App4A,Spp1A	Lepidoptera;Hemiptera;Blattodea	*Plutella xylostella;Laodelphax striatellus;Spodoptera litura;Blattella germanica*
GCF_002551805.1	*Bacillus thuringiensis*	AFS098336			Yes			
GCF_002551695.1	*Bacillus thuringiensis*	AFS099677	App4Aa1,Spp1Aa1	2	Yes	App4A,Spp1A	Lepidoptera;Hemiptera;Blattodea	*Plutella xylostella;Laodelphax striatellus;Spodoptera litura;Blattella germanica*
GCF_002550915.1	*Bacillus thuringiensis*	AFS090355	App4Aa1,Spp1Aa1	2	No	App4A,Spp1A	Lepidoptera;Hemiptera;Blattodea	*Plutella xylostella;Laodelphax striatellus;Spodoptera litura;Blattella germanica*
GCF_002550215.1	*Bacillus thuringiensis*	AFS098399	Cry5Ba1,Spp1Aa1	2	Yes	cry5B,Spp1A	Rhabditida;Lepidoptera;Blattodea	*Caenorhabditis elegans;Ancylostoma ceylanicum;Heligmosomoides polygyrus bakeri;Meloidogyne incognita;Spodoptera litura;Blattella germanica*
GCF_002550185.1	*Bacillus thuringiensis*	AFS098506	Spp1Aa1	1	No	Spp1A	Lepidoptera;Blattodea	*Spodoptera litura;Blattella germanica*
GCF_002268645.2	*Bacillus thuringiensis*	1126_1	Cry1Ia42,Cry2Ab41,Cry9Ea12,Cry1Ca18,Vip3Aa79,Spp1Aa1	6	Yes	cry1I,cry2A,Cry9E,cry1C,vip3Aa,Spp1A	Coleoptera;Lepidoptera;Rhabditida;Diptera;Hemiptera	*Leptinotarsa decemlineata;Ostrinia furnacalis;Lobesia botrana;Plutella xylostella;Earias insulana;cotton boll*

							;Dipteran;Blattodea;Aedes aegypti	*weevil;Meloidogyne incognita;Helicoverpa armigera;Aedes aegypti;Diaphorina citri;Agrotis exclamationis;Cydia pomonella;Myzus persicae;Helicoverpa punctigera;Spodoptera frugiperda;Helicoverpa zea;Chilo partellus;Blattella germanica;Spodoptera litura*
GCF_002236945.1	*Bacillus thuringiensis*	HD133	Cry1Ia42,Cry2Ab41,Cry9Ea12,Cry1Ca18,Cry1Da2,Vip3Aa79,Spp1Aa1	7	Yes	cry1I,cry2A,Cry9E,cry1C,cry1D,vip3Aa,Spp1A	Coleoptera;Lepidoptera;Rhabditida;Diptera;Hemiptera;Dipteran;Blattodea;Aedes aegypti	*Leptinotarsa decemlineata;Ostrinia furnacalis;Lobesia botrana;Plutella xylostella;Earias insulana;cotton boll weevil;Meloidogyne incognita;Helicoverpa armigera;Aedes aegypti;Diaphorina citri;Agrotis exclamationis;Cydia pomonella;Myzus persicae;Ostrinia nubilalis;Spodoptera exigua;Spodoptera frugiperda;Bombyx mori;Spodoptera litura;Epiphyas postvittana;Helicoverpa punctigera;Helicoverpa zea;Chilo partellus;Blattella germanica*
GCF_002192515.1	*Bacillus thuringiensis*	BM-BT15426			No			

GCF_002184245.1	*Bacillus thuringiensis*	YGd22-03	Mpp64Ba1,Mpp64Ca1,Spp1Aa1	3	Yes	Mpp64B+Mpp64C,Spp1A	Hemiptera;Lepidoptera;Blattodea	*Sogatella furcifera;Laodelphax striatellus;Spodoptera litura;Blattella germanica*
GCF_002173755.1	*Bacillus thuringiensis*	SCG04-02	Cry7Ba1,Cry7Ia1,Spp1Aa1	3	No	cry7B,Spp1A	Lepidoptera;Blattodea	*Plutella xylostella;Spodoptera litura;Blattella germanica*
GCF_002148155.1	*Bacillus thuringiensis serovar medellin*	T30001	Cry30Aa1,Cry29Aa1,Cry11Bb1,Cyt1Ca1,Cyt2Bc1,Cyt1Ab1,Spp1Aa1	7	Yes	Cry11B,cyt2B,cyt1A,Spp1A	Diptera;Hemiptera;Coleoptera;Lepidoptera;Blattodea	*Aedes aegypti;Anopheles albimanus;Culex quinquefasciatus;Anopheles stephensi;Anopheles gambiae;Culex pipiens;Bradysia difformis;Culex pipien;Diaphorina citri;Plutella xylostella;Spodoptera litura;Blattella germanica*
GCF_002148105.1	*Bacillus thuringiensis serovar indiana*	h16	Spp1Aa1	1	No	Spp1A	Lepidoptera;Blattodea	*Spodoptera litura;Blattella germanica*
GCF_002148045.1	*Bacillus thuringiensis serovar jegathesan*	BGSC4CF1	Cry24Aa1,Cry11Ba1,Cry30Ca2,Cry25Aa1,Cry19Aa1,Cyt2Bb1,Mpp60Ba3,Mpp60Aa1,Spp1Aa1	9	No	cry24A,Cry11B,cry25A,Cry19A,cyt2B,Mpp60B,Mpp60A,Spp1A	Diptera;Lepidoptera;Blattodea	*Aedes aegypti;Culex quinquefasciatus;Anopheles albimanus;Anopheles stephensi;Culex pipiens;Anopheles gambiae;Bradysia difformis;Spodoptera litura;Blattella germanica*
GCF_002148025.1	*[Bacillus*	BGSC4AH1	Spp1Aa1,Vpb4Ca1	2	No	Spp1A	Lepidoptera;Blattodea	*Spodoptera litura;Blattella germanica*

	thuringiensis] serovar konkukian							
GCF_002148015.1	*Bacillus thuringiensis serovar palmanyolensis*	BGSC 4BS1	Spp1Aa1	1	No	Spp1A	Lepidoptera;Blattodea	*Spodoptera litura;Blattella germanica*
GCF_002147965.1	*Bacillus thuringiensis serovar iberica*	BGSC 4BW1	Spp1Aa1	1	No	Spp1A	Lepidoptera;Blattodea	*Spodoptera litura;Blattella germanica*
GCF_002147955.1	*Bacillus thuringiensis serovar pirenaica*	BGSC 4BU1	Cry8Ba2,Spp1Aa1,Vpa2Ai2,Vpb1Ea1	4	Yes	cry8B,Spp1A	Coleoptera;Lepidoptera;Blattodea	*Anthonomus grandis;Chrysomela scripta;Xanthogaleruca luteola;Spodoptera litura;Blattella germanica*
GCF_002147895.1	*Bacillus thuringiensis serovar higo*	BGSC 4AU1	Cry27Aa1,Cry20Ba2,Cry19Ba1,Spp1Aa1	4	No	cry27A,cry19B,Spp1A	Diptera;Lepidoptera;Blattodea	*Anopheles stephensi;Aedes aegypti;Culex pipiens molestus;Spodoptera litura;Blattella germanica*
GCF_002	*Bacillus*	BGSC	Cry66Aa2,Cry41Ba2,B	3	No			

147835.1	*thuringiensis serovar oswaldocruzi*	4AS1	mp1-other					
GCF_002147825.1	*Bacillus thuringiensis serovar yunnanensis*	BGSC 4AM1	Spp1Aa1,Tpp80Ad1	2	Yes	Spp1A,Tpp80A	Lepidoptera;Blattodea;Diptera;Hemiptera	*Spodoptera litura;Blattella germanica;Culex pipiens pallens;Culex quinquefasciatus;Aedes aegypti;Anopheles gambiae;Laodelphax striatellus*
GCF_002147725.1	*Bacillus thuringiensis serovar aizawai*	T07183	Cry1Ia42,Cry2Ab41,Cry9Ea12,Cry1Ca18,Cry1Da2,Vip3Aa79,Spp1Aa1	7	Yes	cry1I,cry2A,Cry9E,cry1C,cry1D,vip3Aa,Spp1A	Coleoptera;Lepidoptera;Rhabditida;Diptera;Hemiptera;Dipteran;Blattodea;Aedes aegypti	*Leptinotarsa decemlineata;Ostrinia furnacalis;Lobesia botrana;Plutella xylostella;Earias insulana;cotton boll weevil;Meloidogyne incognita;Helicoverpa armigera;Aedes aegypti;Diaphorina citri;Agrotis exclamationis;Cydia pomonella;Myzus persicae;Ostrinia nubilalis;Spodoptera exigua;Spodoptera frugiperda;Bombyx mori;Spodoptera litura;Epiphyas postvittana;Helicoverpa punctigera;Helicoverpa zea;Chilo partellus;Blattella germanica*
GCF_002147685.1	*Bacillus thuringi*	T07153	Cry1Aa3,Cry1Ia42,Cry2Ab41,Cry9Ea12,Cry1	8	Yes	cry1A,cry1I,cry2A,Cry9E,cry1C,cry1D,vip3	Lepidoptera;Coleoptera;Rhabditida;	*Plutella xylostella;Ostrinia furnacalis;Helicoverpa*

	ensis serovar aizawai		Ca18,Cry1Da2,Vip3Aa79,Spp1Aa1			Aa,Spp1A	Diptera;Hemiptera;Dipteran;Blattodea;Aedes aegypti	*armigera;Leucinodes orbonalis;Trichoplusia ni;Hyphantria cunea;Spodoptera frugiperda;Pieris rapae;Chilo suppressalis;Leptinotarsa decemlineata;Lobesia botrana;Earias insulana;cotton boll weevil;Meloidogyne incognita;Aedes aegypti;Diaphorina citri;Agrotis exclamationis;Cydia pomonella;Myzus persicae;Ostrinia nubilalis;Spodoptera exigua;Bombyx mori;Spodoptera litura;Epiphyas postvittana;Helicoverpa punctigera;Helicoverpa zea;Chilo partellus;Blattella germanica*
GCF_002147645.1	*Bacillus thuringiensis serovar aizawai*	T07148	Cry1Ia42,Cry2Ab41,Cry9Ea12,Cry1Ca18,Cry1Da2,Vip3Aa79,Spp1Aa1	7	Yes	cry1I,cry2A,Cry9E,cry1C,cry1D,vip3Aa,Spp1A	Coleoptera;Lepidoptera;Rhabditida;Diptera;Hemiptera;Dipteran;Blattodea;Aedes aegypti	*Leptinotarsa decemlineata;Ostrinia furnacalis;Lobesia botrana;Plutella xylostella;Earias insulana;cotton boll weevil;Meloidogyne incognita;Helicoverpa armigera;Aedes aegypti;Diaphorina citri;Agrotis exclamationis;Cydia pomonella;Myzus persicae;Ostrinia nubilalis;Spodoptera exigua;Spodoptera frugiperda;Bombyx mori;Spodoptera litura;Epiphyas*

								postvittana;Helicoverpa punctigera;Helicoverpa zea;Chilo partellus;Blattella germanica
GCF_002147635.1	*Bacillus thuringiensis serovar aizawai*	T07128	Cry1Aa3,Cry1Ia42,Cry2Ab41,Cry9Ea12,Vip3Aa79,Spp1Aa1	6	Yes	cry1A,cry1I,cry2A,Cry9E,vip3Aa,Spp1A	Lepidoptera;Coleoptera;Rhabditida;Diptera;Hemiptera;Blattodea;Aedes aegypti	*Plutella xylostella;Ostrinia furnacalis;Helicoverpa armigera;Leucinodes orbonalis;Trichoplusia ni;Hyphantria cunea;Spodoptera frugiperda;Pieris rapae;Chilo suppressalis;Leptinotarsa decemlineata;Lobesia botrana;Earias insulana;cotton boll weevil;Meloidogyne incognita;Aedes aegypti;Diaphorina citri;Agrotis exclamationis;Cydia pomonella;Helicoverpa punctigera;Helicoverpa zea;Chilo partellus;Blattella germanica;Spodoptera litura*
GCF_002147605.1	*Bacillus thuringiensis serovar aizawai*	T07030	Cry1Ia42,Cry2Ab41,Cry1Ca18,Cry1Da2,Vip3Aa79,Spp1Aa1	6	Yes	cry1I,cry2A,cry1C,cry1D,vip3Aa,Spp1A	Coleoptera;Lepidoptera;Rhabditida;Diptera;Hemiptera;Dipteran;Blattodea;Aedes aegypti	*Leptinotarsa decemlineata;Ostrinia furnacalis;Lobesia botrana;Plutella xylostella;Earias insulana;cotton boll weevil;Meloidogyne incognita;Helicoverpa armigera;Aedes aegypti;Diaphorina citri;Myzus persicae;Ostrinia nubilalis;Spodoptera exigua;Spodoptera*

								frugiperda;Bombyx mori;Spodoptera litura;Epiphyas postvittana;Helicoverpa punctigera;Helicoverpa zea;Chilo partellus;Blattella germanica
GCF_002147575.1	*Bacillus thuringiensis serovar aizawai*	T07005	Cry1Aa3,Cry1Ia42,Cry2Ab41,Cry9Ea12,Cry1Ca18,Cry1Da2,Vip3Aa79,Spp1Aa1	8	Yes	cry1A,cry1I,cry2A,Cry9E,cry1C,cry1D,vip3Aa,Spp1A	Lepidoptera;Coleoptera;Rhabditida;Diptera;Hemiptera;Dipteran;Blattodea;Aedes aegypti	*Plutella xylostella;Ostrinia furnacalis;Helicoverpa armigera;Leucinodes orbonalis;Trichoplusia ni;Hyphantria cunea;Spodoptera frugiperda;Pieris rapae;Chilo suppressalis;Leptinotarsa decemlineata;Lobesia botrana;Earias insulana;cotton boll weevil;Meloidogyne incognita;Aedes aegypti;Diaphorina citri;Agrotis exclamationis;Cydia pomonella;Myzus persicae;Ostrinia nubilalis;Spodoptera exigua;Bombyx mori;Spodoptera litura;Epiphyas postvittana;Helicoverpa punctigera;Helicoverpa zea;Chilo partellus;Blattella germanica*
GCF_002147565.1	*Bacillus thuringiensis serovar aizawai*	T07019	Cry1Ac9,Cry1Aa3,Cry1Ia42,Cry2Ab41,Cry9Ea12,Cry1Ca18,Cry1Da2,Vip3Aa79,Spp1Aa1	9	Yes	cry1A,cry1A,cry1I,cry2A,Cry9E,cry1C,cry1D,vip3Aa,Spp1A	Lepidoptera;Coleoptera;Rhabditida;Diptera;Hemiptera;Dipteran;Blattodea;Aedes aegypti	*Plutella xylostella;Ostrinia furnacalis;Helicoverpa armigera;Leucinodes orbonalis;Trichoplusia ni;Hyphantria cunea;Spodoptera frugiperda;Pieris*

								rapae;Chilo suppressalis;Leptinotarsa decemlineata;Lobesia botrana;Earias insulana;cotton boll weevil;Meloidogyne incognita;Aedes aegypti;Diaphorina citri;Agrotis exclamationis;Cydia pomonella;Myzus persicae;Ostrinia nubilalis;Spodoptera exigua;Bombyx mori;Spodoptera litura;Epiphyas postvittana;Helicoverpa punctigera;Helicoverpa zea;Chilo partellus;Blattella germanica
GCF_002147555.1	*Bacillus thuringiensis*	I13	Cry1Ia42,Cry2Ab41,Cry9Ea12,Cry1Ca18,Cry1Da2,Vip3Aa79,Spp1Aa1	7	Yes	cry1I,cry2A,Cry9E,cry1C,cry1D,vip3Aa,Spp1A	Coleoptera;Lepidoptera;Rhabditida;Diptera;Hemiptera;Dipteran;Blattodea;Aedes aegypti	*Leptinotarsa decemlineata;Ostrinia furnacalis;Lobesia botrana;Plutella xylostella;Earias insulana;cotton boll weevil;Meloidogyne incognita;Helicoverpa armigera;Aedes aegypti;Diaphorina citri;Agrotis exclamationis;Cydia pomonella;Myzus persicae;Ostrinia nubilalis;Spodoptera exigua;Spodoptera frugiperda;Bombyx mori;Spodoptera litura;Epiphyas postvittana;Helicoverpa punctigera;Helicoverpa zea;Chilo partellus;Blattella germanica*

GCF_002147535.1	*Bacillus thuringiensis*	HD5	Cry2Aa22,Cry1Ia42,Cry1Ac2,Cry1Ea7,Spp1Aa1	5	Yes	cry2A,cry1I,cry1A,cry1E,Spp1A	Lepidoptera;Diptera;Hemiptera;Coleoptera;Rhabditida;Blattodea	*Helicoverpa armigera;Aedes aegypti;Diaphorina citri;Leptinotarsa decemlineata;Ostrinia furnacalis;Lobesia botrana;Plutella xylostella;Earias insulana;cotton boll weevil;Meloidogyne incognita;Leucinodes orbonalis;Trichoplusia ni;Hyphantria cunea;Spodoptera frugiperda;Pieris rapae;Chilo suppressalis;Spodoptera exigua;Malacosoma disstria;Bursaphelenchus xylophilus;Spodoptera litura;Blattella germanica*
GCF_002147495.1	*Bacillus thuringiensis serovar darmstadiensis*	YBT032	Cry1Ea7,Spp1Aa1	2	No	cry1E,Spp1A	Lepidoptera;Hemiptera;Rhabditida;Blattodea	*Spodoptera exigua;Spodoptera frugiperda;Malacosoma disstria;Bursaphelenchus xylophilus;Spodoptera litura;Blattella germanica*
GCF_002147475.1	*Bacillus thuringiensis serovar ostriniae*	BGSC4Z1	Spp1Aa1,Tpp80Ad1	2	Yes	Spp1A,Tpp80A	Lepidoptera;Blattodea;Diptera;Hemiptera	*Spodoptera litura;Blattella germanica;Culex pipiens pallens;Culex quinquefasciatus;Aedes aegypti;Anopheles gambiae;Laodelphax striatellus*
GCF_002	*Bacillus*	BGSC	Cry1Ac9,Cry1Aa3,Cry	6	Yes	cry1A,cry1A,cry1I,cry	Lepidoptera;Coleo	*Plutella xylostella;Ostrinia*

147435.1	*thuringiensis serovar colmeri*	4X1	1Ia42,Cry2Ab41,Vip3Aa79,Spp1Aa1			2A,vip3Aa,Spp1A	ptera;Rhabditida;Diptera;Hemiptera;Blattodea;Aedes aegypti	*furnacalis;Helicoverpa armigera;Leucinodes orbonalis;Trichoplusia ni;Hyphantria cunea;Spodoptera frugiperda;Pieris rapae;Chilo suppressalis;Leptinotarsa decemlineata;Lobesia botrana;Earias insulana;cotton boll weevil;Meloidogyne incognita;Aedes aegypti;Diaphorina citri;Helicoverpa punctigera;Helicoverpa zea;Chilo partellus;Blattella germanica;Spodoptera litura*
GCF_002147425.1	*Bacillus thuringiensis serovar tohokuensis*	BGSC 4V1	Spp1Aa1	1	No	Spp1A	Lepidoptera;Blattodea	*Spodoptera litura;Blattella germanica*
GCF_002147415.1	*Bacillus thuringiensis serovar kumamtoensis*	BGSC 4W1	Cry7Ab8,Spp1Aa1	2	Yes	cry7A,Spp1A	Coleoptera;Lepidoptera;Blattodea	*Leptinotarsa decemlineata;Henosepilachna vigintiomaculata;Acanthoscelides obtectus;Henosepilachna vigintioctomaculata;Cylas puncticollis;Spodoptera litura;Blattella germanica*
GCF_002147375.1	*Bacillus thuringi*	BGSC 4T1	Cry1Bd1,Cry1Cb2,Cry1Ga1,Cry1Ib3,Cry1Fb5,	16	Yes	Vpa2Ae + Vpb1Ac,cry1B,cry1C,	Hemiptera;Lepidoptera;Diptera;Cole	*Aphis gossypii;Plutella xylostella;Ostrinia*

	ensis serovar wuhanensis		Cry2Ac6,Cry2Ab4,Cry1Gb2,Cry1Ab35,Cry9Aa6,Cry1Db2,Vip3Af3,Vip3Aa17,Spp1Aa1,Vpa2Ae3,Vpb1Ac1			cry1G,cry1I,cry1F,cry2A,cry2A,cry1G,cry1A,Cry9A,cry1D,vip3Af,vip3Aa,Spp1A	optera;Dipteran;Rhabditida;Blattodea;Aedes aegypti	*nubilalis;Spodoptera frugiperda;Maruca vitrata;Manduca sexta;cotton boll weevil;Myzus persicae;Aedes aegypti;Pieris rapae;Thaumetopoea pityocampa;Leptinotarsa decemlineata;Ostrinia furnacalis;Lobesia botrana;Earias insulana;Meloidogyne incognita;Agrotis ipsilon;Diabrotica virgifera virgifera;Helicoverpa armigera;Diaphorina citri;Leucinodes orbonalis;Trichoplusia ni;Hyphantria cunea;Chilo suppressalis;Pieris brassicae;Phthorimaea operculella;Spodoptera exigua;Bombyx mori;Spodoptera litura;Epiphyas postvittana;Helicoverpa punctigera;Helicoverpa zea;Chilo partellus;Blattella germanica*
GCF_002147325.1	*Bacillus thuringiensis serovar thompsoni*	BGSC 4O1	Cry1Bb1,Cry1Ab24,Cry1Hb2,Cry2Ad1,Cry1Ib5,Cry1Fb4,Cry1Ka1,Vip3Af3,Vip3Ag6,Spp1Aa1,Vpa2Ac2,Vpb1Ca1	12	No	cry1B,cry1A,cry1H,cry2A,cry1I,cry1F,cry1K,vip3Af,vip3Ag,Spp1A	Lepidoptera;Hemiptera;Diptera;Coleoptera;Rhabditida;Blattodea	*Plutella xylostella;Ostrinia nubilalis;Spodoptera frugiperda;Maruca vitrata;Manduca sexta;cotton boll weevil;Ostrinia furnacalis;Helicoverpa armigera;Leucinodes*

								orbonalis;Trichoplusia ni;Hyphantria cunea;Pieris rapae;Chilo suppressalis;Spodoptera exigua;Aedes aegypti;Diaphorina citri;Leptinotarsa decemlineata;Lobesia botrana;Earias insulana;Meloidogyne incognita;Agrotis ipsilon;Diabrotica virgifera virgifera;Artogeia rapae;Spodoptera littoralis;Chrysodeixis chalcites;Spodoptera litura;Blattella germanica
GCF_002147315.1	*Bacillus thuringiensis serovar toumanoffi*	BGSC 4N1	Cry1Bb1,Cry1Ab24,Cry1Ja2,Cry1Da3,Cry1Hb2,Cry1Id2,Cry1Nb1,Cry2Ad1,Vip3Af3,App4Aa1,Spp1Aa1,Vpa2Ac2,Vpb1Ca1	13	Yes	cry1B,cry1A,cry1J,cry1D,cry1H,cry1I,cry1N,cry2A,vip3Af,App4A,Spp1A	Lepidoptera;Hemiptera;Diptera;Coleoptera;Rhabditida;Blattodea	*Plutella xylostella;Ostrinia nubilalis;Spodoptera frugiperda;Maruca vitrata;Manduca sexta;cotton boll weevil;Ostrinia furnacalis;Helicoverpa armigera;Leucinodes orbonalis;Trichoplusia ni;Hyphantria cunea;Pieris rapae;Chilo suppressalis;Lobesia botrana;Diabrotica undecimpunctata howardi;Spodoptera exigua;Bombyx mori;Spodoptera litura;Epiphyas postvittana;Leptinotarsa decemlineata;Earias insulana;Meloidogyne*

								incognita;Colaphellus bowringi;Aedes aegypti;Diaphorina citri;Agrotis ipsilon;Laodelphax striatellus;Blattella germanica
GCF_002147255.1	*Bacillus thuringiensis serovar aizawai*	BGSC 4J5	Cry2Aa22,Cry1Ia42,Cry1Ac2,Cry1Ea7,Spp1Aa1	5	Yes	cry2A,cry1I,cry1A,cry1E,Spp1A	Lepidoptera;Diptera;Hemiptera;Coleoptera;Rhabditida;Blattodea	*Helicoverpa armigera;Aedes aegypti;Diaphorina citri;Leptinotarsa decemlineata;Ostrinia furnacalis;Lobesia botrana;Plutella xylostella;Earias insulana;cotton boll weevil;Meloidogyne incognita;Leucinodes orbonalis;Trichoplusia ni;Hyphantria cunea;Spodoptera frugiperda;Pieris rapae;Chilo suppressalis;Spodoptera exigua;Malacosoma disstria;Bursaphelenchus xylophilus;Spodoptera litura;Blattella germanica*
GCF_002147245.1	*Bacillus thuringiensis serovar tolworthi*	BGSC 4L3	Cry1Ia42,Cry2Aa22,Cry2Ab41,Cry1Aa17,Cry1Ac5,Vip3Aa79,Spp1Aa1	7	Yes	cry1I,cry2A,cry2A,cry1A,cry1A,vip3Aa,Spp1A	Coleoptera;Lepidoptera;Rhabditida;Diptera;Hemiptera;Blattodea;Aedes aegypti	*Leptinotarsa decemlineata;Ostrinia furnacalis;Lobesia botrana;Plutella xylostella;Earias insulana;cotton boll weevil;Meloidogyne incognita;Helicoverpa armigera;Aedes aegypti;Diaphorina citri;Leucinodes orbonalis;Trichoplusia ni;Hyphantria cunea;Spodoptera frugiperda;Pieris*

								rapae;Chilo suppressalis;Helicoverpa punctigera;Helicoverpa zea;Chilo partellus;Blattella germanica;Spodoptera litura
GCF_002147235.1	*Bacillus thuringiensis serovar aizawai*	BGSC 4J4	Cry1Aa3,Cry1Ia42,Cry2Ab41,Cry9Ea12,Vip3Aa79,Spp1Aa1	6	Yes	cry1A,cry1I,cry2A,Cry9E,vip3Aa,Spp1A	Lepidoptera;Coleoptera;Rhabditida;Diptera;Hemiptera;Blattodea;Aedes aegypti	*Plutella xylostella;Ostrinia furnacalis;Helicoverpa armigera;Leucinodes orbonalis;Trichoplusia ni;Hyphantria cunea;Spodoptera frugiperda;Pieris rapae;Chilo suppressalis;Leptinotarsa decemlineata;Lobesia botrana;Earias insulana;cotton boll weevil;Meloidogyne incognita;Aedes aegypti;Diaphorina citri;Agrotis exclamationis;Cydia pomonella;Helicoverpa punctigera;Helicoverpa zea;Chilo partellus;Blattella germanica;Spodoptera litura*
GCF_002147185.1	*Bacillus thuringiensis serovar aizawai*	BGSC 4J3	Cry1Ia42,Cry2Ab41,Cry9Ea12,Cry1Ca18,Cry1Da2,Vip3Aa79,Spp1Aa1	7	Yes	cry1I,cry2A,Cry9E,cry1C,cry1D,vip3Aa,Spp1A	Coleoptera;Lepidoptera;Rhabditida;Diptera;Hemiptera;Dipteran;Blattodea;Aedes aegypti	*Leptinotarsa decemlineata;Ostrinia furnacalis;Lobesia botrana;Plutella xylostella;Earias insulana;cotton boll weevil;Meloidogyne incognita;Helicoverpa armigera;Aedes aegypti;Diaphorina citri;Agrotis exclamationis;Cydia*

								pomonella;Myzus persicae;Ostrinia nubilalis;Spodoptera exigua;Spodoptera frugiperda;Bombyx mori;Spodoptera litura;Epiphyas postvittana;Helicoverpa punctigera;Helicoverpa zea;Chilo partellus;Blattella germanica
GCF_002147165.1	*Bacillus thuringiensis serovar aizawai*	BGSC 4J1	Cry1Ia42,Cry2Ab41,Cry1Ca18,Cry1Da2,Cry1Aa3,Vip3Aa79,Spp1Aa1	7	Yes	cry1I,cry2A,cry1C,cry1D,cry1A,vip3Aa,Spp1A	Coleoptera;Lepidoptera;Rhabditida;Diptera;Hemiptera;Dipteran;Blattodea;Aedes aegypti	*Leptinotarsa decemlineata;Ostrinia furnacalis;Lobesia botrana;Plutella xylostella;Earias insulana;cotton boll weevil;Meloidogyne incognita;Helicoverpa armigera;Aedes aegypti;Diaphorina citri;Myzus persicae;Ostrinia nubilalis;Spodoptera exigua;Spodoptera frugiperda;Bombyx mori;Spodoptera litura;Epiphyas postvittana;Leucinodes orbonalis;Trichoplusia ni;Hyphantria cunea;Pieris rapae;Chilo suppressalis;Helicoverpa punctigera;Helicoverpa zea;Chilo partellus;Blattella germanica*
GCF_002147155.1	*Bacillus thuringiensis*	BGSC 4J2	Cry1Aa3,Cry1Ia42,Cry2Ab41,Cry1Ca18,Cry1Da2,Vip3Aa79,Spp1Aa	7	Yes	cry1A,cry1I,cry2A,cry1C,cry1D,vip3Aa,Spp1A	Lepidoptera;Coleoptera;Rhabditida;Diptera;Hemiptera	*Plutella xylostella;Ostrinia furnacalis;Helicoverpa armigera;Leucinodes*

	serovar aizawai		1				;Dipteran;Blattodea;Aedes aegypti	*orbonalis;Trichoplusia ni;Hyphantria cunea;Spodoptera frugiperda;Pieris rapae;Chilo suppressalis;Leptinotarsa decemlineata;Lobesia botrana;Earias insulana;cotton boll weevil;Meloidogyne incognita;Aedes aegypti;Diaphorina citri;Myzus persicae;Ostrinia nubilalis;Spodoptera exigua;Bombyx mori;Spodoptera litura;Epiphyas postvittana;Helicoverpa punctigera;Helicoverpa zea;Chilo partellus;Blattella germanica*
GCF_002147125.1	*Bacillus thuringiensis serovar canadensis*	BGSC 4H1			No			
GCF_002147105.1	*Bacillus thuringiensis serovar galleriae*	BGSC 4G1	Cry1Bd1,Cry1Cb2,Cry1Ga1,Cry1Ib3,Cry1Db1,Cry1Fb5,Cry2Ac6,Cry2Ab4,Cry1Gb2,Cry1Ab35,Cry9Aa6,Vip3Af3,Vip3Aa17,Spp1Aa1,Vpa2Ae3,Vpb1Ac1	16	Yes	Vpa2Ae + Vpb1Ac,cry1B,cry1C,cry1G,cry1I,cry1D,cry1F,cry2A,cry2A,cry1G,cry1A,Cry9A,vip3Af,vip3Aa,Spp1A	Hemiptera;Lepidoptera;Diptera;Coleoptera;Dipteran;Rhabditida;Blattodea;Aedes aegypti	*Aphis gossypii;Plutella xylostella;Ostrinia nubilalis;Spodoptera frugiperda;Maruca vitrata;Manduca sexta;cotton boll weevil;Myzus persicae;Aedes aegypti;Pieris rapae;Thaumetopoea pityocampa;Leptinotarsa*

								decemlineata;Ostrinia furnacalis;Lobesia botrana;Earias insulana;Meloidogyne incognita;Spodoptera exigua;Bombyx mori;Spodoptera litura;Epiphyas postvittana;Agrotis ipsilon;Diabrotica virgifera virgifera;Helicoverpa armigera;Diaphorina citri;Leucinodes orbonalis;Trichoplusia ni;Hyphantria cunea;Chilo suppressalis;Pieris brassicae;Phthorimaea operculella;Helicoverpa punctigera;Helicoverpa zea;Chilo partellus;Blattella germanica
GCF_002147085.1	*Bacillus thuringiensis serovar azorensis*	BGSC 4CB1	Cry1Bd1,Cry1Cb2,Cry1Ga1,Cry1Ib3,Cry1Fb5,Cry2Ac6,Cry2Ab4,Cry9Aa6,Cry1Gb2,Cry1Ab35,Vip3Af3,Vip3Aa17,Spp1Aa1,Vpa2Ae3,Vpb1Ac1	15	Yes	Vpa2Ae + Vpb1Ac,cry1B,cry1C,cry1G,cry1I,cry1F,cry2A,cry2A,Cry9A,cry1G,cry1A,vip3Af,vip3Aa,Spp1A	Hemiptera;Lepidoptera;Diptera;Coleoptera;Dipteran;Rhabditida;Blattodea;Aedes aegypti	*Aphis gossypii;Plutella xylostella;Ostrinia nubilalis;Spodoptera frugiperda;Maruca vitrata;Manduca sexta;cotton boll weevil;Myzus persicae;Aedes aegypti;Pieris rapae;Thaumetopoea pityocampa;Leptinotarsa decemlineata;Ostrinia furnacalis;Lobesia botrana;Earias insulana;Meloidogyne incognita;Agrotis ipsilon;Diabrotica*

								virgifera virgifera;Helicoverpa armigera;Diaphorina citri;Pieris brassicae;Phthorimaea operculella;Chilo suppressalis;Leucinodes orbonalis;Trichoplusia ni;Hyphantria cunea;Spodoptera exigua;Helicoverpa punctigera;Helicoverpa zea;Chilo partellus;Blattella germanica;Spodoptera litura
GCF_002147055.1	*Bacillus thuringiensis serovar londrina*	BGSC 4BF1	Cry8Kb3,Spp1Aa1,Vpa2Ai2,Vpb1Ea2,Vpb1Ea1	5	Yes	cry8K,Spp1A	Coleoptera;Lepidoptera;Blattodea	*Anthonomus grandis;cotton boll weevil;Spodoptera litura;Blattella germanica*
GCF_002147025.1	*Bacillus thuringiensis serovar soonche on*	BGSC 4BB1	Cry7Ba1,Cry7Ia1	2	No	cry7B	Lepidoptera	*Plutella xylostella*
GCF_002146985.1	*Bacillus thuringiensis serovar brasilensis*	BGSC 4AY1	Bmp1-other	1	No			

GCF_002146945.1	*Bacillus thuringiensis serovar subtoxicus*	BGSC 4I4	Cry1Ba4,Spp1Aa1	2	Yes	cry1B,Spp1A	Lepidoptera;Hemiptera;Diptera;Coleoptera;Blattodea	*Plutella xylostella;Ostrinia nubilalis;Spodoptera frugiperda;Maruca vitrata;Manduca sexta;cotton boll weevil;Spodoptera litura;Blattella germanica*
GCF_002146925.1	*Bacillus thuringiensis serovar canadensis*	BGSC 4H2	Spp1Aa1	1	No	Spp1A	Lepidoptera;Blattodea	*Spodoptera litura;Blattella germanica*
GCF_002146885.1	*Bacillus thuringiensis serovar graciosensis*	BGSC 4CD1	Cry21Da1,Cry21Ea1,Cry21Ca2	3	No	Cry21D,Cry21E,Cry21C	Rhabditida	*Heligmosomoides polygyrus bakeri;Caenorhabditis elegans*
GCF_002146755.1	*Bacillus thuringiensis serovar kim*	BGSC 4BP1	Cry7Ka1,Cry7Gc1,Spp1Aa1	3	Yes	cry7G,Spp1A	Coleoptera;Lepidoptera;Blattodea	*Diabrotica undecimpunctata howardi;Diabrotica virgifera virgifera;Spodoptera litura;Blattella germanica*
GCF_002146705.1	*Bacillus thuringiensis serovar muju*	BGSC 4BL1	Spp1Aa1	1	No	Spp1A	Lepidoptera;Blattodea	*Spodoptera litura;Blattella germanica*

GCF_002146655.1	*Bacillus thuringiensis serovar roskildiensis*	BGSC 4BG1	Cry7Ba1,Cry7Ia1	2	No	cry7B	Lepidoptera	*Plutella xylostella*
GCF_002146625.1	*Bacillus thuringiensis serovar finitimus*	BGSC 4B2	Cry28Aa2,Cry26Aa1,Mpp83Aa1	3	No			
GCF_002146615.1	*Bacillus thuringiensis serovar novosibirsk*	BGSC 4AX1	Cry11Aa3,Cry10Aa4,Cyt1Ca1,Cyt1Aa11,Cyt2Ba17,Mpp60Ba3,Mpp60Aa3	7	No	cry11A,cry10A,cyt1A,cyt2B,Mpp60B,Mpp60A	Diptera;Coleoptera;Hemiptera;Lepidoptera	*Aedes aegypti;Anopheles albimanus;Culex quinquefasciatu;Culex pipiens;cotton boll weevil;Culex quinquefasciatus;Culex pipien;Diaphorina citri;Plutella xylostella;Anopheles stephensi;Anopheles gambiae;Bradysia difformis*
GCF_002146545.1	*Bacillus thuringiensis serovar japonensis*	BGSC 4AT1	Cry1Ie3,Cry9Da4,Cry1Na2,Cry9Eb3,Vip3Aa51,Spp1Aa1	6	Yes	cry1I,Cry9D,cry1N,Cry9E,vip3Aa,Spp1A	Coleoptera;Lepidoptera;Rhabditida;Blattodea;Aedes aegypti	*Leptinotarsa decemlineata;Ostrinia furnacalis;Lobesia botrana;Plutella xylostella;Earias insulana;cotton boll weevil;Meloidogyne incognita;Bombyx mori;Hyphantria cunea;Colaphellus bowringi;Agrotis exclamationis;Cydia*

								pomonella;Helicoverpa punctigera;Spodoptera frugiperda;Helicoverpa zea;Chilo partellus;Blattella germanica;Spodoptera litura
GCF_002146525.1	*Bacillus thuringiensis serovar seoulensis*	BGSC 4AQ1	Mpp82Aa1,Bmp1-other	2	Yes			
GCF_002146505.1	*Bacillus thuringiensis serovar coreanensis*	BGSC 4AL1	Cry7Ba1,Cry7Ia1	2	No	cry7B	Lepidoptera	*Plutella xylostella*
GCF_002146465.1	*Bacillus thuringiensis serovar nigeriensis*	BGSC 4AZ1	Spp1Aa1	1	No	Spp1A	Lepidoptera;Blattodea	*Spodoptera litura;Blattella germanica*
GCF_002146445.1	*Bacillus thuringiensis serovar jinghon*	BGSC 4AR1	Cry1Ie3,Cry9Da4,Cry1Na2,Cry9Eb3,Vip3Aa51,Spp1Aa1	6	Yes	cry1I,Cry9D,cry1N,Cry9E,vip3Aa,Spp1A	Coleoptera;Lepidoptera;Rhabditida;Blattodea;Aedes aegypti	*Leptinotarsa decemlineata;Ostrinia furnacalis;Lobesia botrana;Plutella xylostella;Earias insulana;cotton boll weevil;Meloidogyne incognita;Bombyx mori;Hyphantria*

	giensis							*cunea;Colaphellus bowringi;Agrotis exclamationis;Cydia pomonella;Helicoverpa punctigera;Spodoptera frugiperda;Helicoverpa zea;Chilo partellus;Blattella germanica;Spodoptera litura*
GCF_002146425.1	*Bacillus thuringiensis serovar fukuokaensis*	BGSC 4AP1	Cry1Ie3,Cry9Aa4,Cry1Na2,Vip3Aa51,Spp1Aa1	5	Yes	cry1I,Cry9A,cry1N,vip3Aa,Spp1A	Coleoptera;Lepidoptera;Rhabditida;Blattodea;Aedes aegypti	*Leptinotarsa decemlineata;Ostrinia furnacalis;Lobesia botrana;Plutella xylostella;Earias insulana;cotton boll weevil;Meloidogyne incognita;Pieris brassicae;Phthorimaea operculella;Chilo suppressalis;Colaphellus bowringi;Helicoverpa punctigera;Spodoptera frugiperda;Helicoverpa zea;Chilo partellus;Blattella germanica;Spodoptera litura*
GCF_002146405.1	*Bacillus thuringiensis serovar sumiyoshiensis*	BGSC 4AO1	Cry9Aa4,Cry9Ee3,Cry1Ie3,Cry1Na2,Vip3Aa51,Spp1Aa1	6	Yes	Cry9A,Cry9E,cry1I,cry1N,vip3Aa,Spp1A	Lepidoptera;Coleoptera;Rhabditida;Blattodea;Aedes aegypti	*Plutella xylostella;Pieris brassicae;Phthorimaea operculella;Chilo suppressalis;Agrotis exclamationis;Cydia pomonella;Leptinotarsa decemlineata;Ostrinia furnacalis;Lobesia botrana;Earias insulana;cotton boll*

								weevil;Meloidogyne incognita;Colaphellus bowringi;Helicoverpa punctigera;Spodoptera frugiperda;Helicoverpa zea;Chilo partellus;Blattella germanica;Spodoptera litura
GCF_002146335.1	*Bacillus thuringiensis serovar thuringiensis*	BGSC 4A4	Cry1Ba8,Mpp5Ab1,Spp1Aa1,Vpa2Bb4,Vpb1Bb2,Vpb1Bc1	6	Yes	cry1B,Mpp5A,Spp1A	Lepidoptera;Hemiptera;Diptera;Coleoptera;Blattodea	*Plutella xylostella;Ostrinia nubilalis;Spodoptera frugiperda;Maruca vitrata;Manduca sexta;cotton boll weevil;Colaphellus bowringi;Leptinotarsa decemlineata;Spodoptera litura;Blattella germanica*
GCF_002146315.1	*Bacillus thuringiensis serovar amagiensis*	BGSC 4AE1	Cry1Bd1,Cry1Cb2,Cry1Ga1,Cry1Ib3,Cry1Fb5,Cry2Ac6,Cry1Gb2,Cry1Ab35,Cry1Db2,Cry2Ab4,Cry9Aa6,Vip3Af3,Vip3Aa17,Spp1Aa1,Vpa2Ae3,Vpb1Ac1	16	Yes	Vpa2Ae + Vpb1Ac,cry1B,cry1C,cry1G,cry1I,cry1F,cry2A,cry1G,cry1A,cry1D,cry2A,Cry9A,vip3Af,vip3Aa,Spp1A	Hemiptera;Lepidoptera;Diptera;Coleoptera;Dipteran;Rhabditida;Blattodea;Aedes aegypti	*Aphis gossypii;Plutella xylostella;Ostrinia nubilalis;Spodoptera frugiperda;Maruca vitrata;Manduca sexta;cotton boll weevil;Myzus persicae;Aedes aegypti;Pieris rapae;Thaumetopoea pityocampa;Leptinotarsa decemlineata;Ostrinia furnacalis;Lobesia botrana;Earias insulana;Meloidogyne incognita;Agrotis ipsilon;Diabrotica virgifera virgifera;Helicoverpa armigera;Diaphorina*

								citri;Leucinodes orbonalis;Trichoplusia ni;Hyphantria cunea;Chilo suppressalis;Spodoptera exigua;Bombyx mori;Spodoptera litura;Epiphyas postvittana;Pieris brassicae;Phthorimaea operculella;Helicoverpa punctigera;Helicoverpa zea;Chilo partellus;Blattella germanica
GCF_002024545.1	*Bacillus thuringiensis*	DNG9	App4Aa1,Spp1Aa1	2	Yes	App4A,Spp1A	Lepidoptera;Hemiptera;Blattodea	*Plutella xylostella;Laodelphax striatellus;Spodoptera litura;Blattella germanica*
GCF_001883945.1	*Bacillus thuringiensis*	FN31			No			
GCF_001880125.1	*Bacillus thuringiensis serovar kurstaki*	BP865	Cry1Ia42,Cry2Aa22,Cry2Ab41,Vip3Aa79,Spp1Aa1	5	Yes	cry1I,cry2A,cry2A,vip3Aa,Spp1A	Coleoptera;Lepidoptera;Rhabditida;Diptera;Hemiptera;Blattodea;Aedes aegypti	*Leptinotarsa decemlineata;Ostrinia furnacalis;Lobesia botrana;Plutella xylostella;Earias insulana;cotton boll weevil;Meloidogyne incognita;Helicoverpa armigera;Aedes aegypti;Diaphorina citri;Helicoverpa punctigera;Spodoptera frugiperda;Helicoverpa zea;Chilo partellus;Blattella germanica;Spodoptera litura*
GCF_001880115.1	*Bacillus thuringi*	HD-133	Cry1Ia42,Cry2Ab41,Cry9Ea12,Cry1Ca18,Vip3	6	Yes	cry1I,cry2A,Cry9E,cry1C,vip3Aa,Spp1A	Coleoptera;Lepidoptera;Rhabditida;	*Leptinotarsa decemlineata;Ostrinia furnacalis;Lobesia botrana;Plutella*

	ensis serovar aizawai		Aa79,Spp1Aa1				Diptera;Hemiptera;Dipteran;Blattodea;Aedes aegypti	*xylostella;Earias insulana;cotton boll weevil;Meloidogyne incognita;Helicoverpa armigera;Aedes aegypti;Diaphorina citri;Agrotis exclamationis;Cydia pomonella;Myzus persicae;Helicoverpa punctigera;Spodoptera frugiperda;Helicoverpa zea;Chilo partellus;Blattella germanica;Spodoptera litura*
GCF_001757755.1	*Bacillus thuringiensis*	GOE5	Bmp1-other	1	No			
GCF_001757745.1	*Bacillus thuringiensis*	GOE4			Yes			
GCF_001685565.1	*Bacillus thuringiensis*	MYBT18246	Cry13Aa2,Spp1Aa1	2	Yes	Cry13A,Spp1A	Rhabditida;Lepidoptera;Blattodea	*Bursaphelenchus xylophilus;Spodoptera litura;Blattella germanica*
GCF_001675515.1	*Bacillus thuringiensis serovar coreanensis*	ST7	Mpp45Aa1,Spp1Aa1	2	Yes	Spp1A	Lepidoptera;Blattodea	*Spodoptera litura;Blattella germanica*
GCF_001640965.1	*Bacillus thuringi*	BGSC4C1	Cry2Ab41,Cry1Ae1,Vip3Aa80,Spp1Aa1,Vpa2	6	No	Vpa2Ae + Vpb1Ac,cry2A,cry1A,	Hemiptera;Lepidoptera;Diptera;Blatt	*Aphis gossypii;Helicoverpa armigera;Aedes aegypti;Diaphorina*

	ensis serovar alesti		Ae3,Vpb1Ac2			vip3Aa,Spp1A	odea;Aedes aegypti	*citri;Plutella xylostella;Ostrinia furnacalis;Leucinodes orbonalis;Trichoplusia ni;Hyphantria cunea;Spodoptera frugiperda;Pieris rapae;Chilo suppressalis;Helicoverpa punctigera;Helicoverpa zea;Chilo partellus;Blattella germanica;Spodoptera litura*
GCF_001598095.1	*Bacillus thuringiensis*	HD12	Cry1Bb1,Cry1Ab24,Cry1Ja2,Cry1Da3,Cry1Hb2,Cry1Id2,Cry1Nb1,Cry2Ad1,Cry1Fb4,Vip3Af3,Spp1Aa1,Vpa2Ac2,Vpb1Ca1	13	No	cry1B,cry1A,cry1J,cry1D,cry1H,cry1I,cry1N,cry2A,cry1F,vip3Af,Spp1A	Lepidoptera;Hemiptera;Diptera;Coleoptera;Rhabditida;Blattodea	*Plutella xylostella;Ostrinia nubilalis;Spodoptera frugiperda;Maruca vitrata;Manduca sexta;cotton boll weevil;Ostrinia furnacalis;Helicoverpa armigera;Leucinodes orbonalis;Trichoplusia ni;Hyphantria cunea;Pieris rapae;Chilo suppressalis;Lobesia botrana;Diabrotica undecimpunctata howardi;Spodoptera exigua;Bombyx mori;Spodoptera litura;Epiphyas postvittana;Leptinotarsa decemlineata;Earias insulana;Meloidogyne incognita;Colaphellus bowringi;Aedes aegypti;Diaphorina citri;Agrotis ipsilon;Diabrotica virgifera virgifera;Blattella*

								germanica
GCF_001595725.1	*Bacillus thuringiensis*	Bt185	Cry8Ea5,Cry8Fa1,Mpp5Ab1	3	No	cry8E,cry8F,Mpp5A	Coleoptera	*Holotrichia parallela;Anomala corpulenta;H. oblita;Phyllotreta striolata;Colaphellus bowringi;Leptinotarsa decemlineata*
GCF_001455345.1	*Bacillus thuringiensis*	CTC			Yes			
GCF_001420855.1	*Bacillus thuringiensis*	YWC2-8	Cry2Aa22,Cry1Ac5,Spp1Aa1	3	Yes	cry2A,cry1A,Spp1A	Lepidoptera;Diptera;Hemiptera;Blattodea	*Helicoverpa armigera;Aedes aegypti;Diaphorina citri;Plutella xylostella;Ostrinia furnacalis;Leucinodes orbonalis;Trichoplusia ni;Hyphantria cunea;Spodoptera frugiperda;Pieris rapae;Chilo suppressalis;Spodoptera litura;Blattella germanica*
GCF_001276195.1	*Bacillus thuringiensis*	BMB3201	Cry2Aa22,Cry1Ac2,Cry2Ab4,Cry1Ia42,Vip3Aa59,Spp1Aa1	6	Yes	cry2A,cry1A,cry2A,cry1I,vip3Aa,Spp1A	Lepidoptera;Diptera;Hemiptera;Coleoptera;Rhabditida;Blattodea;Aedes aegypti	*Helicoverpa armigera;Aedes aegypti;Diaphorina citri;Plutella xylostella;Ostrinia furnacalis;Leucinodes orbonalis;Trichoplusia ni;Hyphantria cunea;Spodoptera frugiperda;Pieris rapae;Chilo suppressalis;Leptinotarsa decemlineata;Lobesia botrana;Earias insulana;cotton boll weevil;Meloidogyne incognita;Helicoverpa*

								punctigera;Helicoverpa zea;Chilo partellus;Blattella germanica;Spodoptera litura
GCF_001183785.1	*Bacillus thuringiensis serovar indiana*	HD521	Cry7Fb1,Cry7Da3,Cry7Ga2,Spp1Aa1	4	Yes	cry7G,Spp1A	Coleoptera;Lepidoptera;Blattodea	*Diabrotica undecimpunctata howardi;Diabrotica virgifera virgifera;Spodoptera litura;Blattella germanica*
GCF_001017635.1	*Bacillus thuringiensis*	YC-10	Cry1Ia42,Cry2Aa22,Cry2Ab41,Cry1Aa17,Cry1Ac5,Vip3Aa79,Spp1Aa1	7	No	cry1I,cry2A,cry2A,cry1A,cry1A,vip3Aa,Spp1A	Coleoptera;Lepidoptera;Rhabditida;Diptera;Hemiptera;Blattodea;Aedes aegypti	*Leptinotarsa decemlineata;Ostrinia furnacalis;Lobesia botrana;Plutella xylostella;Earias insulana;cotton boll weevil;Meloidogyne incognita;Helicoverpa armigera;Aedes aegypti;Diaphorina citri;Leucinodes orbonalis;Trichoplusia ni;Hyphantria cunea;Spodoptera frugiperda;Pieris rapae;Chilo suppressalis;Helicoverpa punctigera;Helicoverpa zea;Chilo partellus;Blattella germanica;Spodoptera litura*
GCF_000969665.1	*Bacillus thuringiensis serovar mexicanensis*	27	Cry1Ac9,Cry2Ab41,Vip3Aa79,Spp1Aa1	4	No	cry1A,cry2A,vip3Aa,Spp1A	Lepidoptera;Diptera;Hemiptera;Blattodea;Aedes aegypti	*Plutella xylostella;Ostrinia furnacalis;Helicoverpa armigera;Leucinodes orbonalis;Trichoplusia ni;Hyphantria cunea;Spodoptera frugiperda;Pieris rapae;Chilo suppressalis;Aedes*

								aegypti;Diaphorina citri;Helicoverpa punctigera;Helicoverpa zea;Chilo partellus;Blattella germanica;Spodoptera litura
GCF_000948325.1	*Bacillus thuringiensis*	Lr3/2			No			
GCF_000948235.1	*Bacillus thuringiensis*	Lr7/2	Spp1Aa1	1	No	Spp1A	Lepidoptera;Blattodea	*Spodoptera litura;Blattella germanica*
GCF_000878525.1	*Bacillus thuringiensis Sbt003*	Sbt003	Cry5Ca2,Cry5Da2,Cry65Aa2,Cry5Ea2,Spp1Aa1	5	Yes	cry5C,cry5D,cry65A,Spp1A	Rhabditida;cells;Lepidoptera;Blattodea	*Meloidogyne incognita;Caenorhabditis elegans;MDA-MB-231 cells;Spodoptera litura;Blattella germanica*
GCF_000835235.1	*Bacillus thuringiensis serovar kurstaki*	HD 1	Cry1Ab9,Cry1Ia42,Cry2Aa22,Cry2Ab41,Cry1Aa17,Cry1Ac5,Vip3Aa79,Spp1Aa1	8	Yes	cry1A,cry1I,cry2A,cry2A,cry1A,cry1A,vip3Aa,Spp1A	Lepidoptera;Coleoptera;Rhabditida;Diptera;Hemiptera;Blattodea;Aedes aegypti	*Plutella xylostella;Ostrinia furnacalis;Helicoverpa armigera;Leucinodes orbonalis;Trichoplusia ni;Hyphantria cunea;Spodoptera frugiperda;Pieris rapae;Chilo suppressalis;Leptinotarsa decemlineata;Lobesia botrana;Earias insulana;cotton boll weevil;Meloidogyne incognita;Aedes aegypti;Diaphorina citri;Helicoverpa punctigera;Helicoverpa zea;Chilo partellus;Blattella*

								germanica;Spodoptera litura
GCF_000835025.1	*Bacillus thuringiensis HD1002*	HD1002	Mpp60Ba3,Mpp60Aa3	2	No	Mpp60B,Mpp60A	Diptera	*Culex quinquefasciatus*
GCF_000833085.1	*Bacillus thuringiensis*	97-27			No			
GCF_000832485.1	*Bacillus thuringiensis*	HD1011	Bmp1-other	1	No			
GCF_000816555.1	*Bacillus thuringiensis*	AK47	Cry1Ia42,Cry2Aa22,Cry2Ab41,Vip3Aa79,Spp1Aa1	5	Yes	cry1I,cry2A,cry2A,vip3Aa,Spp1A	Coleoptera;Lepidoptera;Rhabditida;Diptera;Hemiptera;Blattodea;Aedes aegypti	*Leptinotarsa decemlineata;Ostrinia furnacalis;Lobesia botrana;Plutella xylostella;Earias insulana;cotton boll weevil;Meloidogyne incognita;Helicoverpa armigera;Aedes aegypti;Diaphorina citri;Helicoverpa punctigera;Spodoptera frugiperda;Helicoverpa zea;Chilo partellus;Blattella germanica;Spodoptera litura*
GCF_000803665.1	*Bacillus thuringiensis serovar galleriae*	4G5	Cry1Ac9,Cry1Aa3,Cry1Ia42,Cry2Ab41,Cry9Ea12,Cry1Ca18,Cry1Da2,Vip3Aa79,Spp1Aa1	9	Yes	cry1A,cry1A,cry1I,cry2A,Cry9E,cry1C,cry1D,vip3Aa,Spp1A	Lepidoptera;Coleoptera;Rhabditida;Diptera;Hemiptera;Dipteran;Blattodea;Aedes aegypti	*Plutella xylostella;Ostrinia furnacalis;Helicoverpa armigera;Leucinodes orbonalis;Trichoplusia ni;Hyphantria cunea;Spodoptera frugiperda;Pieris rapae;Chilo*

								suppressalis;Leptinotarsa decemlineata;Lobesia botrana;Earias insulana;cotton boll weevil;Meloidogyne incognita;Aedes aegypti;Diaphorina citri;Agrotis exclamationis;Cydia pomonella;Myzus persicae;Ostrinia nubilalis;Spodoptera exigua;Bombyx mori;Spodoptera litura;Epiphyas postvittana;Helicoverpa punctigera;Helicoverpa zea;Chilo partellus;Blattella germanica
GCF_000774075.2	*Bacillus thuringiensis*	XL6	Cry7Ea3,Cry61Aa3,Cry67Aa2,Cry7Fa2,Cry7Gd1,Cry7Cb1	6	No	cry7G,cry7C	Coleoptera;Orthoptera	*Diabrotica undecimpunctata howardi;Diabrotica virgifera virgifera;Locusta migratoria manilensis*
GCF_000747545.1	*Bacillus thuringiensis serovar kurstaki str. YBT-1520*	YBT-1520	Cry1Aa3,Cry1Ia42,Cry2Aa22,Cry2Ab41,Cry1Ac4,Vip3Aa79,Spp1Aa1	7	Yes	cry1A,cry1I,cry2A,cry2A,cry1A,vip3Aa,Spp1A	Lepidoptera;Coleoptera;Rhabditida;Diptera;Hemiptera;Blattodea;Aedes aegypti	*Plutella xylostella;Ostrinia furnacalis;Helicoverpa armigera;Leucinodes orbonalis;Trichoplusia ni;Hyphantria cunea;Spodoptera frugiperda;Pieris rapae;Chilo suppressalis;Leptinotarsa decemlineata;Lobesia botrana;Earias insulana;cotton boll weevil;Meloidogyne incognita;Aedes aegypti;Diaphorina citri;Helicoverpa punctigera;Helicoverpa zea;Chilo*

								partellus;Blattella germanica;Spodoptera litura
GCF_000600315.1	*Bacillus thuringiensis*	NBIN-866	Cry1Bd1,Cry1Ab35,Cry1Cb2,Cry1Ga1,Cry1Gb2,Cry1Ib3,Cry1Db1,Cry1Fb5,Cry2Ac6,Cry2Ab4,Vip3Af3,Spp1Aa1,Vpa2Ae3,Vpb1Ac1	14	Yes	Vpa2Ae + Vpb1Ac,cry1B,cry1A,cry1C,cry1G,cry1G,cry1I,cry1D,cry1F,cry2A,cry2A,vip3Af,Spp1A	Hemiptera;Lepidoptera;Diptera;Coleoptera;Dipteran;Rhabditida;Blattodea	*Aphis gossypii;Plutella xylostella;Ostrinia nubilalis;Spodoptera frugiperda;Maruca vitrata;Manduca sexta;cotton boll weevil;Ostrinia furnacalis;Helicoverpa armigera;Leucinodes orbonalis;Trichoplusia ni;Hyphantria cunea;Pieris rapae;Chilo suppressalis;Myzus persicae;Aedes aegypti;Thaumetopoea pityocampa;Leptinotarsa decemlineata;Lobesia botrana;Earias insulana;Meloidogyne incognita;Spodoptera exigua;Bombyx mori;Spodoptera litura;Epiphyas postvittana;Agrotis ipsilon;Diabrotica virgifera virgifera;Diaphorina citri;Blattella germanica*
GCF_000585975.1	*Bacillus thuringiensis serovar israelensis*	4Q7			No			

GCF_000571955.1	*Bacillus thuringiensis serovar tolworthi NA205-3*	NA205-3	Cry1Ba8,Cry1Ia42,Cry2Aa22,Cry2Ab41,Vip3Aa79,Mpp5Ab1,Spp1Aa1,Vpa2Bb4,Vpb1Bb2	9	Yes	cry1B,cry1I,cry2A,cry2A,vip3Aa,Mpp5A,Spp1A	Lepidoptera;Hemiptera;Diptera;Coleoptera;Rhabditida;Blattodea;Aedes aegypti	*Plutella xylostella;Ostrinia nubilalis;Spodoptera frugiperda;Maruca vitrata;Manduca sexta;cotton boll weevil;Leptinotarsa decemlineata;Ostrinia furnacalis;Lobesia botrana;Earias insulana;Meloidogyne incognita;Helicoverpa armigera;Aedes aegypti;Diaphorina citri;Helicoverpa punctigera;Helicoverpa zea;Chilo partellus;Blattella germanica;Colaphellus bowringi;Spodoptera litura*
GCF_000530375.1	*Bacillus thuringiensis DB27*	DB27	Cry21Fa1,Cry21Ha1,Spp1Aa1	3	Yes	Cry21F,Cry21H,Spp1A	Rhabditida;Lepidoptera;Blattodea	*Heligmosomoides polygyrus bakeri;Caenorhabditis elegans;Spodoptera litura;Blattella germanica*
GCF_000497525.1	*Bacillus thuringiensis YBT-1518*	YBT-1518	Cry5Ba2,App6Aa2,Spp1Aa1,Xpp55Aa2	4	Yes	cry5B,App6A,Spp1A,Xpp55A	Rhabditida;Lepidoptera;Blattodea;Coleoptera	*Caenorhabditis elegans;Ancylostoma ceylanicum;Heligmosomoides polygyrus bakeri;Meloidogyne incognita;Bursaphelenchus xylophilus;Spodoptera litura;Blattella germanica;Meloidogyne javanica;Phyllotreta cruciferae*
GCF_000468995.3	*Bacillus thuringi*	Hu4-2	Cry9Ea12,Cry1Ia7,Cry1Ca18,Vip3Ca2,Spp1A	5	Yes	Cry9E,cry1I,cry1C,vip3C,Spp1A	Lepidoptera;Coleoptera;Rhabditida;	*Agrotis exclamationis;Cydia pomonella;Leptinotarsa*

	ensis serovar aizawai str. Hu4-2		a1				Dipteran;Blattodea	*decemlineata;Ostrinia furnacalis;Lobesia botrana;Plutella xylostella;Earias insulana;cotton boll weevil;Meloidogyne incognita;Myzus persicae;Aedes aegypti;Chrysodeixis chalcites;Mamestra brassicae;Trichoplusia ni;Spodoptera eridania;Helicoverpa zea;Chrysodeixis includens;Spodoptera litura;Blattella germanica*
GCF_000468975.2	*Bacillus thuringiensis serovar aizawai str. Leapi01*	Leapi01	Cry1Ia42,Cry2Ab41,Cry9Ea12,Vip3Aa79,Spp1Aa1	5	Yes	cry1I,cry2A,Cry9E,vip3Aa,Spp1A	Coleoptera;Lepidoptera;Rhabditida;Diptera;Hemiptera;Blattodea;Aedes aegypti	*Leptinotarsa decemlineata;Ostrinia furnacalis;Lobesia botrana;Plutella xylostella;Earias insulana;cotton boll weevil;Meloidogyne incognita;Helicoverpa armigera;Aedes aegypti;Diaphorina citri;Agrotis exclamationis;Cydia pomonella;Helicoverpa punctigera;Spodoptera frugiperda;Helicoverpa zea;Chilo partellus;Blattella germanica;Spodoptera litura*
GCF_000387405.2	*Bacillus thuringiensis T01-328*	T01-328	Cry1Ia42,Cry2Aa22,Cry2Ab41,Cry1Ba8,Vip3Aa79,Mpp5Ab1,Spp1Aa1,Vpa2Bb4,Vpb1Bb2	9	Yes	cry1I,cry2A,cry2A,cry1B,vip3Aa,Mpp5A,Spp1A	Coleoptera;Lepidoptera;Rhabditida;Diptera;Hemiptera;Blattodea;Aedes aegypti	*Leptinotarsa decemlineata;Ostrinia furnacalis;Lobesia botrana;Plutella xylostella;Earias insulana;cotton boll weevil;Meloidogyne incognita;Helicoverpa*

								armigera;Aedes aegypti;Diaphorina citri;Ostrinia nubilalis;Spodoptera frugiperda;Maruca vitrata;Manduca sexta;Helicoverpa punctigera;Helicoverpa zea;Chilo partellus;Blattella germanica;Colaphellus bowringi;Spodoptera litura
GCF_000342025.1	*Bacillus thuringiensis DAR 81934*	DAR 81934	Spp1Aa1	1	No	Spp1A	Lepidoptera;Blattodea	*Spodoptera litura;Blattella germanica*
GCF_000341665.1	*Bacillus thuringiensis serovar thuringiensis str. IS5056*	IS5056	Cry1Ba8,Cry1Aa3,Cry1Ia42,Cry2Aa22,Cry2Ab41,Cry1Ab21,Vip3Aa79,Mpp5Ab1,Spp1Aa1,Vpa2Bb4,Vpb1Bb2,Vpb1Bc1	12	Yes	cry1B,cry1A,cry1I,cry2A,cry2A,cry1A,vip3Aa,Mpp5A,Spp1A	Lepidoptera;Hemiptera;Diptera;Coleoptera;Rhabditida;Blattodea;Aedes aegypti	*Plutella xylostella;Ostrinia nubilalis;Spodoptera frugiperda;Maruca vitrata;Manduca sexta;cotton boll weevil;Ostrinia furnacalis;Helicoverpa armigera;Leucinodes orbonalis;Trichoplusia ni;Hyphantria cunea;Pieris rapae;Chilo suppressalis;Leptinotarsa decemlineata;Lobesia botrana;Earias insulana;Meloidogyne incognita;Aedes aegypti;Diaphorina citri;Helicoverpa punctigera;Helicoverpa zea;Chilo partellus;Blattella*

								germanica;Colaphellus bowringi;Spodoptera litura
GCF_000338755.1	*Bacillus thuringiensis serovar kurstaki str. HD73*	HD73	Cry1Ac9,Spp1Aa1	2	Yes	cry1A,Spp1A	Lepidoptera;Blattodea	*Plutella xylostella;Ostrinia furnacalis;Helicoverpa armigera;Leucinodes orbonalis;Trichoplusia ni;Hyphantria cunea;Spodoptera frugiperda;Pieris rapae;Chilo suppressalis;Spodoptera litura;Blattella germanica*
GCF_000292705.1	*Bacillus thuringiensis HD-789*	HD-789	Cry11Aa3,Cry10Aa4,Cry4Ba7,Cry4Aa5,Cyt1Ca1,Cyt1Aa11,Cyt2Ba17,Mpp60Ba3,Mpp60Aa3	9	No	cry11A,cry10A,cry4B,cry4A,cyt1A,cyt2B,Mpp60B,Mpp60A	Diptera;Coleoptera;Hemiptera;Lepidoptera	*Aedes aegypti;Anopheles albimanus;Culex quinquefasciatu;Culex pipiens;cotton boll weevil;Anopheles gambiae;Diaphorina citri;Culex quinquefasciatus;Acyrthosiphon pisum;Culex pipien;Plutella xylostella;Anopheles stephensi;Bradysia difformis*
GCF_000292455.1	*Bacillus thuringiensis HD-771*	HD-771	Cry1Aa9,Spp1Aa1	2	Yes	cry1A,Spp1A	Lepidoptera;Blattodea	*Plutella xylostella;Ostrinia furnacalis;Helicoverpa armigera;Leucinodes orbonalis;Trichoplusia ni;Hyphantria cunea;Spodoptera frugiperda;Pieris rapae;Chilo suppressalis;Spodoptera litura;Blattella germanica*
GCF_000193355.1	*Bacillus thuringiensis*	CT-43	Cry1Ba8,Cry1Aa3,Cry1Ia42,Cry2Aa22,Cry2Ab41,Vip3Aa79,Spp1Aa1	7	Yes	cry1B,cry1A,cry1I,cry2A,cry2A,vip3Aa,Spp1A	Lepidoptera;Hemiptera;Diptera;Coleoptera;Rhabditida;	*Plutella xylostella;Ostrinia nubilalis;Spodoptera frugiperda;Maruca vitrata;Manduca*

	serovar chinensis CT-43						Blattodea;Aedes aegypti	*sexta;cotton boll weevil;Ostrinia furnacalis;Helicoverpa armigera;Leucinodes orbonalis;Trichoplusia ni;Hyphantria cunea;Pieris rapae;Chilo suppressalis;Leptinotarsa decemlineata;Lobesia botrana;Earias insulana;Meloidogyne incognita;Aedes aegypti;Diaphorina citri;Helicoverpa punctigera;Helicoverpa zea;Chilo partellus;Blattella germanica;Spodoptera litura*
GCF_000161715.1	*Bacillus thuringiensis IBL 200*	IBL 200	Cry1Bb1,Cry1Ab24,Cry1Ja2,Cry1Da3,Cry1Hb2,Cry1Id2,Cry1Nb1,Cry2Ad1,Vip3Af3,App4Aa1,Spp1Aa1,Vpa2Ac2,Vpb1Ca1	13	Yes	cry1B,cry1A,cry1J,cry1D,cry1H,cry1I,cry1N,cry2A,vip3Af,App4A,Spp1A	Lepidoptera;Hemiptera;Diptera;Coleoptera;Rhabditida;Blattodea	*Plutella xylostella;Ostrinia nubilalis;Spodoptera frugiperda;Maruca vitrata;Manduca sexta;cotton boll weevil;Ostrinia furnacalis;Helicoverpa armigera;Leucinodes orbonalis;Trichoplusia ni;Hyphantria cunea;Pieris rapae;Chilo suppressalis;Lobesia botrana;Diabrotica undecimpunctata howardi;Spodoptera exigua;Bombyx mori;Spodoptera litura;Epiphyas postvittana;Leptinotarsa decemlineata;Earias insulana;Meloidogyne*

								incognita;Colaphellus bowringi;Aedes aegypti;Diaphorina citri;Agrotis ipsilon;Laodelphax striatellus;Blattella germanica
GCF_000161675.1	*Bacillus thuringiensis serovar huazhongensis BGSC 4BD1*	BGSC 4BD1	Cry8La1,Cry8Ib2	2	No	cry8I	Coleoptera	*Holotrichia parallela*
GCF_000161655.1	*Bacillus thuringiensis serovar pondicheriensis BGSC 4BA1*	BGSC 4BA1	Bmp1-other	1	No			
GCF_000161635.1	*Bacillus thuringiensis serovar andalousiensis BGSC 4AW1*	BGSC 4AW1	Cry41Ba2,Cry66Aa2,App4Aa1,Bmp1-other	4	No	App4A	Lepidoptera;Hemiptera	*Plutella xylostella;Laodelphax striatellus*

GCF_000161595.1	*Bacillus thuringiensis serovar monterrey BGSC 4AJ1*	BGSC 4AJ1	Cry7Ab4,Vpb4Aa1,Bmp1-other	3	No	cry7A	Coleoptera	*Leptinotarsa decemlineata;Henosepilachna vigintiomaculata;Acanthoscelides obtectus;Henosepilachna vigintioctomaculata;Cylas puncticollis*
GCF_000161575.1	*Bacillus thuringiensis serovar kurstaki str. T03a001*	T03a001	Cry1Ac9,Spp1Aa1	2	Yes	cry1A,Spp1A	Lepidoptera;Blattodea	*Plutella xylostella;Ostrinia furnacalis;Helicoverpa armigera;Leucinodes orbonalis;Trichoplusia ni;Hyphantria cunea;Spodoptera frugiperda;Pieris rapae;Chilo suppressalis;Spodoptera litura;Blattella germanica*
GCF_000161555.1	*Bacillus thuringiensis serovar pakistani str. T13001*	T13001	Cry5Ca2,Cry5Da2,Cry65Aa2,Cry5Ea2,Spp1Aa1	5	Yes	cry5C,cry5D,cry65A,Spp1A	Rhabditida;cells;Lepidoptera;Blattodea	*Meloidogyne incognita;Caenorhabditis elegans;MDA-MB-231 cells;Spodoptera litura;Blattella germanica*
GCF_000161535.1	*Bacillus thuringiensis serovar sotto*	T04001	Cry1Aa9,Spp1Aa1	2	Yes	cry1A,Spp1A	Lepidoptera;Blattodea	*Plutella xylostella;Ostrinia furnacalis;Helicoverpa armigera;Leucinodes orbonalis;Trichoplusia ni;Hyphantria cunea;Spodoptera frugiperda;Pieris*

	str. T04001							*rapae;Chilo suppressalis;Spodoptera litura;Blattella germanica*
GCF_000161515.1	*Bacillus thuringiensis serovar thuringiensis str. T01001*	T01001	Cry1Ba8,Cry1Ab9,Mpp5Ab1,Spp1Aa1,Vpa2Bb4,Vpb1Bb2,Vpb1Bc1	7	Yes	cry1B,cry1A,Mpp5A,Spp1A	Lepidoptera;Hemiptera;Diptera;Coleoptera;Blattodea	*Plutella xylostella;Ostrinia nubilalis;Spodoptera frugiperda;Maruca vitrata;Manduca sexta;cotton boll weevil;Ostrinia furnacalis;Helicoverpa armigera;Leucinodes orbonalis;Trichoplusia ni;Hyphantria cunea;Pieris rapae;Chilo suppressalis;Colaphellus bowringi;Leptinotarsa decemlineata;Spodoptera litura;Blattella germanica*
GCF_000092165.1	*Bacillus thuringiensis BMB171*	BMB171			Yes			
GCA_905204295.1	*Bacillus thuringiensis*	SRR5713945-bin.19			No			
GCA_057656875.1	*Bacillus thuringiensis*	Soil_C_36			No			
GCA_057474625.1	*Bacillus thuringiensis*	CH070	Spp1Aa1	1	No	Spp1A	Lepidoptera;Blattodea	*Spodoptera litura;Blattella germanica*

GCA_055738025.1	*Bacillus thuringiensis*	CC1	Cry5Ba1,Spp1Aa1	2	Yes	cry5B,Spp1A	Rhabditida;Lepidoptera;Blattodea	*Caenorhabditis elegans;Ancylostoma ceylanicum;Heligmosomoides polygyrus bakeri;Meloidogyne incognita;Spodoptera litura;Blattella germanica*
GCA_053710365.1	*Bacillus thuringiensis*	FDA1236639-S005-001	Vpb1Aa3	1	No			
GCA_053632215.1	*Bacillus thuringiensis*	26MDFML001421-B			No			
GCA_053023985.1	*Bacillus thuringiensis*	WPHL-022239-25	Spp1Aa1	1	No	Spp1A	Lepidoptera;Blattodea	*Spodoptera litura;Blattella germanica*
GCA_050941515.1	*Bacillus thuringiensis*	YE6_3_8	Spp1Aa1	1	No	Spp1A	Lepidoptera;Blattodea	*Spodoptera litura;Blattella germanica*
GCA_050941495.1	*Bacillus thuringiensis*	YE6_3_7	Cry1Ca18,Cry1Da2,Cry2Ab41,Cry1Aa3,Cry1Ia42,Cry3Aa6,Cry9Ea12,Vip3Aa79,Spp1Aa1	9	Yes	cry1C,cry1D,cry2A,cry1A,cry1I,Cry3A,Cry9E,vip3Aa,Spp1A	Lepidoptera;Diptera;Diptera;Hemiptera;Coleoptera;Rhabditida;Blattodea;Aedes aegypti	*Plutella xylostella;Myzus persicae;Aedes aegypti;Ostrinia nubilalis;Spodoptera exigua;Spodoptera frugiperda;Bombyx mori;Spodoptera litura;Epiphyas postvittana;Helicoverpa armigera;Diaphorina citri;Ostrinia furnacalis;Leucinodes*

								orbonalis;Trichoplusia ni;Hyphantria cunea;Pieris rapae;Chilo suppressalis;Leptinotarsa decemlineata;Lobesia botrana;Earias insulana;cotton boll weevil;Meloidogyne incognita;Tenebrio molitor;Hypera postica;Macrosiphum euphorbiae;Agrotis exclamationis;Cydia pomonella;Helicoverpa punctigera;Helicoverpa zea;Chilo partellus;Blattella germanica
GCA_050941475.1	*Bacillus thuringiensis*	Y18	Cry1Ca18,Cry1Da2,Cry2Ab41,Cry1Ia42,Cry9Ea12,Vip3Aa79,Spp1Aa1	7	Yes	cry1C,cry1D,cry2A,cry1I,Cry9E,vip3Aa,Spp1A	Lepidoptera;Diptera;Diptera;Hemiptera;Coleoptera;Rhabditida;Blattodea;Aedes aegypti	*Plutella xylostella;Myzus persicae;Aedes aegypti;Ostrinia nubilalis;Spodoptera exigua;Spodoptera frugiperda;Bombyx mori;Spodoptera litura;Epiphyas postvittana;Helicoverpa armigera;Diaphorina citri;Leptinotarsa decemlineata;Ostrinia furnacalis;Lobesia botrana;Earias insulana;cotton boll weevil;Meloidogyne incognita;Agrotis exclamationis;Cydia*

								pomonella;Helicoverpa punctigera;Helicoverpa zea;Chilo partellus;Blattella germanica
GCA_050941455.1	*Bacillus thuringiensis*	Y17	Cry1Ca18,Cry1Da2,Cry2Ab41,Cry1Ia42,Cry9Ea12,Vip3Aa79,Spp1Aa1	7	Yes	cry1C,cry1D,cry2A,cry1I,Cry9E,vip3Aa,Spp1A	Lepidoptera;Diptera;Diptera;Hemiptera;Coleoptera;Rhabditida;Blattodea;Aedes aegypti	*Plutella xylostella;Myzus persicae;Aedes aegypti;Ostrinia nubilalis;Spodoptera exigua;Spodoptera frugiperda;Bombyx mori;Spodoptera litura;Epiphyas postvittana;Helicoverpa armigera;Diaphorina citri;Leptinotarsa decemlineata;Ostrinia furnacalis;Lobesia botrana;Earias insulana;cotton boll weevil;Meloidogyne incognita;Agrotis exclamationis;Cydia pomonella;Helicoverpa punctigera;Helicoverpa zea;Chilo partellus;Blattella germanica*
GCA_050941435.1	*Bacillus thuringiensis*	Y15	Cry1Ca18,Cry1Da2,Cry2Ab41,Cry1Ia42,Cry9Ea12,Vip3Aa79,Spp1Aa1	7	Yes	cry1C,cry1D,cry2A,cry1I,Cry9E,vip3Aa,Spp1A	Lepidoptera;Diptera;Diptera;Hemiptera;Coleoptera;Rhabditida;Blattodea;Aedes aegypti	*Plutella xylostella;Myzus persicae;Aedes aegypti;Ostrinia nubilalis;Spodoptera exigua;Spodoptera frugiperda;Bombyx mori;Spodoptera litura;Epiphyas postvittana;Helicoverpa*

								armigera;Diaphorina citri;Leptinotarsa decemlineata;Ostrinia furnacalis;Lobesia botrana;Earias insulana;cotton boll weevil;Meloidogyne incognita;Agrotis exclamationis;Cydia pomonella;Helicoverpa punctigera;Helicoverpa zea;Chilo partellus;Blattella germanica
GCA_050941415.1	*Bacillus thuringiensis*	Y13	Cry1Ca18,Cry1Da2,Cry2Ab41,Cry1Ia42,Cry9Ea12,Vip3Aa79,Spp1Aa1	7	Yes	cry1C,cry1D,cry2A,cry1I,Cry9E,vip3Aa,Spp1A	Lepidoptera;Diptera;Diptera;Hemiptera;Coleoptera;Rhabditida;Blattodea;Aedes aegypti	*Plutella xylostella;Myzus persicae;Aedes aegypti;Ostrinia nubilalis;Spodoptera exigua;Spodoptera frugiperda;Bombyx mori;Spodoptera litura;Epiphyas postvittana;Helicoverpa armigera;Diaphorina citri;Leptinotarsa decemlineata;Ostrinia furnacalis;Lobesia botrana;Earias insulana;cotton boll weevil;Meloidogyne incognita;Agrotis exclamationis;Cydia pomonella;Helicoverpa punctigera;Helicoverpa zea;Chilo*

								partellus;Blattella germanica
GCA_050941395.1	*Bacillus thuringiensis*	Y23	Cry1Ca18,Cry1Da2,Cry2Ab41,Cry1Ia42,Cry9Ea12,Vip3Aa79,Spp1Aa1	7	Yes	cry1C,cry1D,cry2A,cry1I,Cry9E,vip3Aa,Spp1A	Lepidoptera;Diptera n;Diptera;Hemiptera;Coleoptera;Rhabditida;Blattodea;Aedes aegypti	*Plutella xylostella;Myzus persicae;Aedes aegypti;Ostrinia nubilalis;Spodoptera exigua;Spodoptera frugiperda;Bombyx mori;Spodoptera litura;Epiphyas postvittana;Helicoverpa armigera;Diaphorina citri;Leptinotarsa decemlineata;Ostrinia furnacalis;Lobesia botrana;Earias insulana;cotton boll weevil;Meloidogyne incognita;Agrotis exclamationis;Cydia pomonella;Helicoverpa punctigera;Helicoverpa zea;Chilo partellus;Blattella germanica*
GCA_050941375.1	*Bacillus thuringiensis*	Y22	Cry1Ca18,Cry1Da2,Cry2Ab41,Cry1Ia42,Cry9Ea12,Vip3Aa79,Spp1Aa1	7	Yes	cry1C,cry1D,cry2A,cry1I,Cry9E,vip3Aa,Spp1A	Lepidoptera;Diptera n;Diptera;Hemiptera;Coleoptera;Rhabditida;Blattodea;Aedes aegypti	*Plutella xylostella;Myzus persicae;Aedes aegypti;Ostrinia nubilalis;Spodoptera exigua;Spodoptera frugiperda;Bombyx mori;Spodoptera litura;Epiphyas postvittana;Helicoverpa armigera;Diaphorina citri;Leptinotarsa*

								decemlineata;Ostrinia furnacalis;Lobesia botrana;Earias insulana;cotton boll weevil;Meloidogyne incognita;Agrotis exclamationis;Cydia pomonella;Helicoverpa punctigera;Helicoverpa zea;Chilo partellus;Blattella germanica
GCA_050941355.1	*Bacillus thuringiensis*	Y21	Cry1Ca18,Cry1Da2,Cry2Ab41,Cry1Ia42,Cry9Ea12,Vip3Aa79,Spp1Aa1	7	Yes	cry1C,cry1D,cry2A,cry1I,Cry9E,vip3Aa,Spp1A	Lepidoptera;Diptera n;Diptera;Hemiptera;Coleoptera;Rhabditida;Blattodea;Aedes aegypti	*Plutella xylostella;Myzus persicae;Aedes aegypti;Ostrinia nubilalis;Spodoptera exigua;Spodoptera frugiperda;Bombyx mori;Spodoptera litura;Epiphyas postvittana;Helicoverpa armigera;Diaphorina citri;Leptinotarsa decemlineata;Ostrinia furnacalis;Lobesia botrana;Earias insulana;cotton boll weevil;Meloidogyne incognita;Agrotis exclamationis;Cydia pomonella;Helicoverpa punctigera;Helicoverpa zea;Chilo partellus;Blattella germanica*
GCA_050	*Bacillus*	Y20	Cry70Bb1,Cry3Aa6,Vi	4	Yes	Cry3A,vip3Aa,Spp1A	Coleoptera;Hemip	*Leptinotarsa decemlineata;Tenebrio*

941335.1	*thuringiensis*		p3Aa79,Spp1Aa1				tera;Lepidoptera;Blattodea;Aedes aegypti	*molitor;Hypera postica;Macrosiphum euphorbiae;Helicoverpa punctigera;Spodoptera frugiperda;Helicoverpa zea;Chilo partellus;Blattella germanica;Spodoptera litura*
GCA_050938315.1	*Bacillus thuringiensis*	Y19	Cry1Ca18,Cry1Da2,Cry2Ab41,Cry3Aa6,Cry1Ia42,Cry9Ea12,Vip3Aa79,Spp1Aa1	8	Yes	cry1C,cry1D,cry2A,Cry3A,cry1I,Cry9E,vip3Aa,Spp1A	Lepidoptera;Diptera;Diptera;Hemiptera;Coleoptera;Rhabditida;Blattodea;Aedes aegypti	*Plutella xylostella;Myzus persicae;Aedes aegypti;Ostrinia nubilalis;Spodoptera exigua;Spodoptera frugiperda;Bombyx mori;Spodoptera litura;Epiphyas postvittana;Helicoverpa armigera;Diaphorina citri;Leptinotarsa decemlineata;Tenebrio molitor;Hypera postica;Macrosiphum euphorbiae;Ostrinia furnacalis;Lobesia botrana;Earias insulana;cotton boll weevil;Meloidogyne incognita;Agrotis exclamationis;Cydia pomonella;Helicoverpa punctigera;Helicoverpa zea;Chilo partellus;Blattella germanica*

GCA_050938275.1	*Bacillus thuringiensis*	Y09	Cry1Ca18,Cry1Da2,Cry2Ab41,Cry3Aa6,Cry1Ia42,Cry9Ea12,Vip3Aa79,Spp1Aa1	8	Yes	cry1C,cry1D,cry2A,Cry3A,cry1I,Cry9E,vip3Aa,Spp1A	Lepidoptera;Diptera;Diptera;Hemiptera;Coleoptera;Rhabditida;Blattodea;Aedes aegypti	*Plutella xylostella;Myzus persicae;Aedes aegypti;Ostrinia nubilalis;Spodoptera exigua;Spodoptera frugiperda;Bombyx mori;Spodoptera litura;Epiphyas postvittana;Helicoverpa armigera;Diaphorina citri;Leptinotarsa decemlineata;Tenebrio molitor;Hypera postica;Macrosiphum euphorbiae;Ostrinia furnacalis;Lobesia botrana;Earias insulana;cotton boll weevil;Meloidogyne incognita;Agrotis exclamationis;Cydia pomonella;Helicoverpa punctigera;Helicoverpa zea;Chilo partellus;Blattella germanica*
GCA_050935735.1	*Bacillus thuringiensis*	YE6_2_18	Cry70Bb1,Spp1Aa1	2	Yes	Spp1A	Lepidoptera;Blattodea	*Spodoptera litura;Blattella germanica*
GCA_050929235.1	*Bacillus thuringiensis*	NYVetLIRN-342-800-512	App4Aa1,Spp1Aa1,Tpp80Ad1	3	Yes	App4A,Spp1A,Tpp80A	Lepidoptera;Hemiptera;Blattodea;Diptera	*Plutella xylostella;Laodelphax striatellus;Spodoptera litura;Blattella germanica;Culex pipiens pallens;Culex*

								quinquefasciatus;Aedes aegypti;Anopheles gambiae
GCA_050530105.1	*Bacillus thuringiensis*	FDA1259959-S007-001	Vpb1Aa3	1	No			
GCA_050530065.1	*Bacillus thuringiensis*	FDA1259958-S005-001	Vpb1Aa3	1	No			
GCA_050291425.1	*Bacillus thuringiensis*	3002312282	Spp1Aa1,Tpp80Ad1,Xpp22Ba1	3	Yes	Spp1A,Tpp80A,Xpp22B	Lepidoptera;Blattodea;Diptera;Hemiptera;Coleoptera	*Spodoptera litura;Blattella germanica;Culex pipiens pallens;Culex quinquefasciatus;Aedes aegypti;Anopheles gambiae;Laodelphax striatellus;Anthonomus grandis;Plutella xylostella*
GCA_050164575.1	*Bacillus thuringiensis*	GM78f			No			
GCA_049380125.1	*Bacillus thuringiensis*	22	Spp1Aa1	1	No	Spp1A	Lepidoptera;Blattodea	*Spodoptera litura;Blattella germanica*
GCA_044949885.1	*Bacillus thuringiensis*	NA 69	Cry7Ab6,Cry7Ja1,Spp1Aa1	3	Yes	cry7A,Spp1A	Coleoptera;Lepidoptera;Blattodea	*Leptinotarsa decemlineata;Henosepilachna vigintiomaculata;Acanthoscelides obtectus;Henosepilachna*

								vigintioctomaculata;Cylas puncticollis;Spodoptera litura;Blattella germanica
GCA_040105495.1	*Bacillus thuringiensis*	B1	Cry2Ab41,Cry1Ia42,Cry2Aa22,Cry1Ab9,Vip3Aa79,Spp1Aa1	6	Yes	cry2A,cry1I,cry2A,cry1A,vip3Aa,Spp1A	Lepidoptera;Diptera;Hemiptera;Coleoptera;Rhabditida;Blattodea;Aedes aegypti	*Helicoverpa armigera;Aedes aegypti;Diaphorina citri;Leptinotarsa decemlineata;Ostrinia furnacalis;Lobesia botrana;Plutella xylostella;Earias insulana;cotton boll weevil;Meloidogyne incognita;Leucinodes orbonalis;Trichoplusia ni;Hyphantria cunea;Spodoptera frugiperda;Pieris rapae;Chilo suppressalis;Helicoverpa punctigera;Helicoverpa zea;Chilo partellus;Blattella germanica;Spodoptera litura*
GCA_039593315.1	*Bacillus thuringiensis*	SMAGNO154			No			
GCA_038928665.1	*Bacillus thuringiensis*	PS03091			No			
GCA_038928505.1	*Bacillus thuringiensis*	PS03092	Spp1Aa1	1	No	Spp1A	Lepidoptera;Blattodea	*Spodoptera litura;Blattella germanica*
GCA_037	*Bacillus*	B-404	Cry1Ba8,Cry1Ab9,Mpp	5	Yes	cry1B,cry1A,Mpp5A,	Lepidoptera;Hemi	*Plutella xylostella;Ostrinia*

907485.1	*thuringiensis*	0	5Ab1,Spp1Aa1,Vpa2Bb4			Spp1A	ptera;Diptera;Coleoptera;Blattodea	*nubilalis;Spodoptera frugiperda;Maruca vitrata;Manduca sexta;cotton boll weevil;Ostrinia furnacalis;Helicoverpa armigera;Leucinodes orbonalis;Trichoplusia ni;Hyphantria cunea;Pieris rapae;Chilo suppressalis;Colaphellus bowringi;Leptinotarsa decemlineata;Spodoptera litura;Blattella germanica*
GCA_037907445.1	*Bacillus thuringiensis*	B-14989	Cry5Ca2,Cry5Ba1,Cry5Ad1,Spp1Aa1	4	Yes	cry5C,cry5B,cry5A,Spp1A	Rhabditida;Lepidoptera;Hymenoptera;Blattodea	*Meloidogyne incognita;Caenorhabditis elegans;Ancylostoma ceylanicum;Heligmosomoides polygyrus bakeri;Haemonchus contortus;Trichostrongylus colubriformis;Ostertagia circumcincta;Diprion pini;Cephacia abietis;Helicoverpa armigera;Spodoptera litura;Blattella germanica*
GCA_037907385.1	*Bacillus thuringiensis*	B-14991	Cry5Ba1,Cry5Ca2,Cry5Ad1,Spp1Aa1	4	Yes	cry5B,cry5C,cry5A,Spp1A	Rhabditida;Lepidoptera;Hymenoptera;Blattodea	*Caenorhabditis elegans;Ancylostoma ceylanicum;Heligmosomoides polygyrus bakeri;Meloidogyne incognita;Haemonchus contortus;Trichostrongylus colubriformis;Ostertagia*

								circumcincta;Diprion pini;Cephacia abietis;Helicoverpa armigera;Spodoptera litura;Blattella germanica
GCA_037907365.1	*Bacillus thuringiensis*	B-14992	Cry10Aa4,Cry11Aa3,Cyt1Ca1,Cyt1Aa11,Cyt2Ba17,Mpp60Aa3,Mpp60Ba3	7	No	cry10A,cry11A,cyt1A,cyt2B,Mpp60A,Mpp60B	Diptera;Coleoptera;Hemiptera;Lepidoptera	*Aedes aegypti;cotton boll weevil;Anopheles albimanus;Culex quinquefasciatu;Culex pipiens;Culex quinquefasciatus;Culex pipien;Diaphorina citri;Plutella xylostella;Anopheles stephensi;Anopheles gambiae;Bradysia difformis*
GCA_037907345.1	*Bacillus thuringiensis*	BD-372			Yes			
GCA_037907325.1	*Bacillus thuringiensis*	HD-418	Cry1Aa9,Spp1Aa1	2	Yes	cry1A,Spp1A	Lepidoptera;Blattodea	*Plutella xylostella;Ostrinia furnacalis;Helicoverpa armigera;Leucinodes orbonalis;Trichoplusia ni;Hyphantria cunea;Spodoptera frugiperda;Pieris rapae;Chilo suppressalis;Spodoptera litura;Blattella germanica*
GCA_037907285.1	*Bacillus thuringiensis*	HD-468	Cry1Aa9,Spp1Aa1	2	Yes	cry1A,Spp1A	Lepidoptera;Blattodea	*Plutella xylostella;Ostrinia furnacalis;Helicoverpa armigera;Leucinodes orbonalis;Trichoplusia ni;Hyphantria cunea;Spodoptera frugiperda;Pieris rapae;Chilo suppressalis;Spodoptera*

								litura;Blattella germanica
GCA_036326085.1	*Bacillus thuringiensis*	HD-570	Cry2Ab41,Cry1Aa3,Cry1Ia42,Cry2Aa22,Cry1Ba8,Vip3Aa79,Mpp5Ab1,Spp1Aa1,Vpa2Bb4,Vpb1Bb2,Vpb1Bc1	11	Yes	cry2A,cry1A,cry1I,cry2A,cry1B,vip3Aa,Mpp5A,Spp1A	Lepidoptera;Diptera;Hemiptera;Coleoptera;Rhabditida;Blattodea;Aedes aegypti	*Helicoverpa armigera;Aedes aegypti;Diaphorina citri;Plutella xylostella;Ostrinia furnacalis;Leucinodes orbonalis;Trichoplusia ni;Hyphantria cunea;Spodoptera frugiperda;Pieris rapae;Chilo suppressalis;Leptinotarsa decemlineata;Lobesia botrana;Earias insulana;cotton boll weevil;Meloidogyne incognita;Ostrinia nubilalis;Maruca vitrata;Manduca sexta;Helicoverpa punctigera;Helicoverpa zea;Chilo partellus;Blattella germanica;Colaphellus bowringi;Spodoptera litura*
GCA_036217505.1	*Bacillus thuringiensis*	B-23367	Cry11Aa3,Cyt1Aa11,Cyt2Ba17,Mpp60Ba3,Mpp60Aa3	5	No	cry11A,cyt1A,cyt2B,Mpp60B,Mpp60A	Diptera;Hemiptera;Coleoptera;Lepidoptera	*Aedes aegypti;Anopheles albimanus;Culex quinquefasciatu;Culex pipiens;Culex quinquefasciatus;Culex pipien;Diaphorina citri;Plutella xylostella;Anopheles stephensi;Anopheles gambiae;Bradysia difformis*
GCA_036215445.1	*Bacillus thuringi*	HD-297	Cry1Ba8,Cry1Aa3,Cry1Ia42,Vip3Aa79	4	No	cry1B,cry1A,cry1I,vip3Aa	Lepidoptera;Hemiptera;Diptera;Cole	*Plutella xylostella;Ostrinia nubilalis;Spodoptera*

	ensis						optera;Rhabditida;Blattodea;Aedes aegypti	*frugiperda;Maruca vitrata;Manduca sexta;cotton boll weevil;Ostrinia furnacalis;Helicoverpa armigera;Leucinodes orbonalis;Trichoplusia ni;Hyphantria cunea;Pieris rapae;Chilo suppressalis;Leptinotarsa decemlineata;Lobesia botrana;Earias insulana;Meloidogyne incognita;Helicoverpa punctigera;Helicoverpa zea;Chilo partellus;Blattella germanica*
GCA_036215395.1	*Bacillus thuringiensis*	HD-318	Cry1Ba8,Cry1Ia42	2	Yes	cry1B,cry1I	Lepidoptera;Hemiptera;Diptera;Coleoptera;Rhabditida	*Plutella xylostella;Ostrinia nubilalis;Spodoptera frugiperda;Maruca vitrata;Manduca sexta;cotton boll weevil;Leptinotarsa decemlineata;Ostrinia furnacalis;Lobesia botrana;Earias insulana;Meloidogyne incognita*
GCA_036214925.1	*Bacillus thuringiensis*	HD-404	Cry1Aa9,Spp1Aa1	2	Yes	cry1A,Spp1A	Lepidoptera;Blattodea	*Plutella xylostella;Ostrinia furnacalis;Helicoverpa armigera;Leucinodes orbonalis;Trichoplusia ni;Hyphantria cunea;Spodoptera frugiperda;Pieris rapae;Chilo suppressalis;Spodoptera litura;Blattella germanica*
GCA_036213225.1	*Bacillus thuringi*	NRS-1211			No			

	ensis							
GCA_035818055.1	*Bacillus thuringiensis*	HD-657	Mpp60Ba3,Mpp60Aa3	2	No	Mpp60B,Mpp60A	Diptera	*Culex quinquefasciatus*
GCA_032825435.1	*Bacillus thuringiensis*	PS3	Cry1Da2,Cry1Ca18,Cry2Ab41,Cry1Ia42,Cry9Ea12,Cry1Aa3,Vip3Aa79,Spp1Aa1	8	Yes	cry1D,cry1C,cry2A,cry1I,Cry9E,cry1A,vip3Aa,Spp1A	Lepidoptera;Diptera;Diptera;Hemiptera;Coleoptera;Rhabditida;Blattodea;Aedes aegypti	*Ostrinia nubilalis;Plutella xylostella;Spodoptera exigua;Spodoptera frugiperda;Bombyx mori;Spodoptera litura;Epiphyas postvittana;Myzus persicae;Aedes aegypti;Helicoverpa armigera;Diaphorina citri;Leptinotarsa decemlineata;Ostrinia furnacalis;Lobesia botrana;Earias insulana;cotton boll weevil;Meloidogyne incognita;Agrotis exclamationis;Cydia pomonella;Leucinodes orbonalis;Trichoplusia ni;Hyphantria cunea;Pieris rapae;Chilo suppressalis;Helicoverpa punctigera;Helicoverpa zea;Chilo partellus;Blattella germanica*
GCA_031941285.1	*Bacillus thuringiensis*	pediatric patient 2	Spp1Aa1	1	No	Spp1A	Lepidoptera;Blattodea	*Spodoptera litura;Blattella germanica*

GCA_029712825.1	*Bacillus thuringiensis*	4A4	Cry1Ba8,Mpp5Ab1,Spp1Aa1,Vpa2Bb4,Vpb1Bc1,Vpb1Bb2	6	Yes	cry1B,Mpp5A,Spp1A	Lepidoptera;Hemiptera;Diptera;Coleoptera;Blattodea	*Plutella xylostella;Ostrinia nubilalis;Spodoptera frugiperda;Maruca vitrata;Manduca sexta;cotton boll weevil;Colaphellus bowringi;Leptinotarsa decemlineata;Spodoptera litura;Blattella germanica*
GCA_029710845.1	*Bacillus thuringiensis*	tky2-1	Spp1Aa1	1	No	Spp1A	Lepidoptera;Blattodea	*Spodoptera litura;Blattella germanica*
GCA_029710825.1	*Bacillus thuringiensis*	gfu2-1			Yes			
GCA_029708935.1	*Bacillus thuringiensis*	CH048	Spp1Aa1	1	No	Spp1A	Lepidoptera;Blattodea	*Spodoptera litura;Blattella germanica*
GCA_029707105.1	*Bacillus thuringiensis*	16_381	Cry1Ca18,Cry1Ia42,Cry1Aa45,Cry2Ab41,Cry9Ea12,Cry1Ab28,Vip3Aa79,Spp1Aa1	8	Yes	cry1C,cry1I,cry1A,cry2A,Cry9E,cry1A,vip3Aa,Spp1A	Lepidoptera;Diptera n;Coleoptera;Rhabditida;Diptera;Hemiptera;Blattodea;Aedes aegypti	*Plutella xylostella;Myzus persicae;Aedes aegypti;Leptinotarsa decemlineata;Ostrinia furnacalis;Lobesia botrana;Earias insulana;cotton boll weevil;Meloidogyne incognita;Helicoverpa armigera;Leucinodes orbonalis;Trichoplusia ni;Hyphantria cunea;Spodoptera frugiperda;Pieris rapae;Chilo suppressalis;Diaphorina citri;Agrotis exclamationis;Cydia*

								pomonella;Helicoverpa punctigera;Helicoverpa zea;Chilo partellus;Blattella germanica;Spodoptera litura
GCA_029707065.1	*Bacillus thuringiensis*	16_1643	Cry1Ia42,Cry1Ca18,Cry1Da2,Cry2Ab41,Cry9Ea12,Vip3Aa79,Spp1Aa1	7	Yes	cry1I,cry1C,cry1D,cry2A,Cry9E,vip3Aa,Spp1A	Coleoptera;Lepidoptera;Rhabditida;Dipteran;Diptera;Hemiptera;Blattodea;Aedes aegypti	*Leptinotarsa decemlineata;Ostrinia furnacalis;Lobesia botrana;Plutella xylostella;Earias insulana;cotton boll weevil;Meloidogyne incognita;Myzus persicae;Aedes aegypti;Ostrinia nubilalis;Spodoptera exigua;Spodoptera frugiperda;Bombyx mori;Spodoptera litura;Epiphyas postvittana;Helicoverpa armigera;Diaphorina citri;Agrotis exclamationis;Cydia pomonella;Helicoverpa punctigera;Helicoverpa zea;Chilo partellus;Blattella germanica*
GCA_029707045.1	*Bacillus thuringiensis*	16_1549	Cry2Ab41,Cry2Aa22,Cry1Ia42,Vip3Aa79,Spp1Aa1	5	Yes	cry2A,cry2A,cry1I,vip3Aa,Spp1A	Lepidoptera;Diptera;Hemiptera;Coleoptera;Rhabditida;Blattodea;Aedes aegypti	*Helicoverpa armigera;Aedes aegypti;Diaphorina citri;Leptinotarsa decemlineata;Ostrinia furnacalis;Lobesia botrana;Plutella xylostella;Earias insulana;cotton boll weevil;Meloidogyne incognita;Helicoverpa punctigera;Spodoptera*

								frugiperda;Helicoverpa zea;Chilo partellus;Blattella germanica;Spodoptera litura
GCA_029707025.1	*Bacillus thuringiensis*	16_1310	Cry2Ab41,Cry2Aa22,Cry1Ia42,Vip3Aa79,Spp1Aa1	5	Yes	cry2A,cry2A,cry1I,vip3Aa,Spp1A	Lepidoptera;Diptera;Hemiptera;Coleoptera;Rhabditida;Blattodea;Aedes aegypti	*Helicoverpa armigera;Aedes aegypti;Diaphorina citri;Leptinotarsa decemlineata;Ostrinia furnacalis;Lobesia botrana;Plutella xylostella;Earias insulana;cotton boll weevil;Meloidogyne incognita;Helicoverpa punctigera;Spodoptera frugiperda;Helicoverpa zea;Chilo partellus;Blattella germanica;Spodoptera litura*
GCA_029707005.1	*Bacillus thuringiensis*	16_379	Cry2Ab41,Cry2Aa22,Cry1Ia42,Vip3Aa79,Spp1Aa1	5	Yes	cry2A,cry2A,cry1I,vip3Aa,Spp1A	Lepidoptera;Diptera;Hemiptera;Coleoptera;Rhabditida;Blattodea;Aedes aegypti	*Helicoverpa armigera;Aedes aegypti;Diaphorina citri;Leptinotarsa decemlineata;Ostrinia furnacalis;Lobesia botrana;Plutella xylostella;Earias insulana;cotton boll weevil;Meloidogyne incognita;Helicoverpa punctigera;Spodoptera frugiperda;Helicoverpa zea;Chilo partellus;Blattella germanica;Spodoptera litura*
GCA_029	*Bacillus*	16_38	Cry2Ab41,Cry2Aa22,C	5	Yes	cry2A,cry2A,cry1I,vip	Lepidoptera;Dipte	*Helicoverpa armigera;Aedes*

706985.1	*thuringiensis*	0	ry1Ia42,Vip3Aa79,Spp1Aa1			3Aa,Spp1A	ra;Hemiptera;Coleoptera;Rhabditida;Blattodea;Aedes aegypti	*aegypti;Diaphorina citri;Leptinotarsa decemlineata;Ostrinia furnacalis;Lobesia botrana;Plutella xylostella;Earias insulana;cotton boll weevil;Meloidogyne incognita;Helicoverpa punctigera;Spodoptera frugiperda;Helicoverpa zea;Chilo partellus;Blattella germanica;Spodoptera litura*
GCA_029706965.1	*Bacillus thuringiensis*	16_372	Cry2Ab41,Cry2Aa22,Cry1Ia42,Vip3Aa79,Spp1Aa1	5	Yes	cry2A,cry2A,cry1I,vip3Aa,Spp1A	Lepidoptera;Diptera;Hemiptera;Coleoptera;Rhabditida;Blattodea;Aedes aegypti	*Helicoverpa armigera;Aedes aegypti;Diaphorina citri;Leptinotarsa decemlineata;Ostrinia furnacalis;Lobesia botrana;Plutella xylostella;Earias insulana;cotton boll weevil;Meloidogyne incognita;Helicoverpa punctigera;Spodoptera frugiperda;Helicoverpa zea;Chilo partellus;Blattella germanica;Spodoptera litura*
GCA_029706945.1	*Bacillus thuringiensis*	16_350	Cry2Aa22,Cry1Ia42,Cry2Ab41,Cry1Ab28,Vip3Aa79,Spp1Aa1	6	Yes	cry2A,cry1I,cry2A,cry1A,vip3Aa,Spp1A	Lepidoptera;Diptera;Hemiptera;Coleoptera;Rhabditida;Blattodea;Aedes aegypti	*Helicoverpa armigera;Aedes aegypti;Diaphorina citri;Leptinotarsa decemlineata;Ostrinia furnacalis;Lobesia botrana;Plutella*

								xylostella;Earias insulana;cotton boll weevil;Meloidogyne incognita;Leucinodes orbonalis;Trichoplusia ni;Hyphantria cunea;Spodoptera frugiperda;Pieris rapae;Chilo suppressalis;Helicoverpa punctigera;Helicoverpa zea;Chilo partellus;Blattella germanica;Spodoptera litura
GCA_029706925.1	*Bacillus thuringiensis*	08_135	Cry1Ia42,Cry1Ca18,Cry1Da2,Cry2Ab41,Cry9Ea12,Vip3Aa79,Spp1Aa1	7	Yes	cry1I,cry1C,cry1D,cry2A,Cry9E,vip3Aa,Spp1A	Coleoptera;Lepidoptera;Rhabditida;Dipteran;Diptera;Hemiptera;Blattodea;Aedes aegypti	*Leptinotarsa decemlineata;Ostrinia furnacalis;Lobesia botrana;Plutella xylostella;Earias insulana;cotton boll weevil;Meloidogyne incognita;Myzus persicae;Aedes aegypti;Ostrinia nubilalis;Spodoptera exigua;Spodoptera frugiperda;Bombyx mori;Spodoptera litura;Epiphyas postvittana;Helicoverpa armigera;Diaphorina citri;Agrotis exclamationis;Cydia pomonella;Helicoverpa punctigera;Helicoverpa zea;Chilo partellus;Blattella germanica*
GCA_029706905.1	*Bacillus thuringiensis*	08_127	Cry1Aa45,Cry1Ia42,Cry1Ca18,Cry1Da2,Cry2Ab41,Cry9Ea12,Cry1A	9	Yes	cry1A,cry1I,cry1C,cry1D,cry2A,Cry9E,cry1A,vip3Aa,Spp1A	Lepidoptera;Coleoptera;Rhabditida;Dipteran;Diptera;	*Plutella xylostella;Ostrinia furnacalis;Helicoverpa armigera;Leucinodes*

			b28,Vip3Aa79,Spp1Aa1				Hemiptera;Blattodea;Aedes aegypti	*orbonalis;Trichoplusia ni;Hyphantria cunea;Spodoptera frugiperda;Pieris rapae;Chilo suppressalis;Leptinotarsa decemlineata;Lobesia botrana;Earias insulana;cotton boll weevil;Meloidogyne incognita;Myzus persicae;Aedes aegypti;Ostrinia nubilalis;Spodoptera exigua;Bombyx mori;Spodoptera litura;Epiphyas postvittana;Diaphorina citri;Agrotis exclamationis;Cydia pomonella;Helicoverpa punctigera;Helicoverpa zea;Chilo partellus;Blattella germanica*
GCA_029706865.1	*Bacillus thuringiensis*	08_138	Cry1Ia42,Cry1Ca18,Cry1Da2,Cry2Ab41,Cry9Ea12,Vip3Aa79,Spp1Aa1	7	Yes	cry1I,cry1C,cry1D,cry2A,Cry9E,vip3Aa,Spp1A	Coleoptera;Lepidoptera;Rhabditida;Dipteran;Diptera;Hemiptera;Blattodea;Aedes aegypti	*Leptinotarsa decemlineata;Ostrinia furnacalis;Lobesia botrana;Plutella xylostella;Earias insulana;cotton boll weevil;Meloidogyne incognita;Myzus persicae;Aedes aegypti;Ostrinia nubilalis;Spodoptera exigua;Spodoptera frugiperda;Bombyx mori;Spodoptera litura;Epiphyas postvittana;Helicoverpa armigera;Diaphorina citri;Agrotis exclamationis;Cydia pomonella;Helicoverpa*

								punctigera;Helicoverpa zea;Chilo partellus;Blattella germanica
GCA_029706845.1	*Bacillus thuringiensis*	08_74	Cry1Ca18,Cry1Da2,Cry2Ab41,Cry1Ia42,Cry9Ea12,Vip3Aa79,Spp1Aa1	7	Yes	cry1C,cry1D,cry2A,cry1I,Cry9E,vip3Aa,Spp1A	Lepidoptera;Diptera;Diptera;Hemiptera;Coleoptera;Rhabditida;Blattodea;Aedes aegypti	*Plutella xylostella;Myzus persicae;Aedes aegypti;Ostrinia nubilalis;Spodoptera exigua;Spodoptera frugiperda;Bombyx mori;Spodoptera litura;Epiphyas postvittana;Helicoverpa armigera;Diaphorina citri;Leptinotarsa decemlineata;Ostrinia furnacalis;Lobesia botrana;Earias insulana;cotton boll weevil;Meloidogyne incognita;Agrotis exclamationis;Cydia pomonella;Helicoverpa punctigera;Helicoverpa zea;Chilo partellus;Blattella germanica*
GCA_029706825.1	*Bacillus thuringiensis*	08_037	Cry2Ab41,Cry2Aa22,Cry1Ia42,Vip3Aa79,Spp1Aa1	5	Yes	cry2A,cry2A,cry1I,vip3Aa,Spp1A	Lepidoptera;Diptera;Hemiptera;Coleoptera;Rhabditida;Blattodea;Aedes aegypti	*Helicoverpa armigera;Aedes aegypti;Diaphorina citri;Leptinotarsa decemlineata;Ostrinia furnacalis;Lobesia botrana;Plutella xylostella;Earias insulana;cotton boll weevil;Meloidogyne incognita;Helicoverpa*

								punctigera;Spodoptera frugiperda;Helicoverpa zea;Chilo partellus;Blattella germanica;Spodoptera litura
GCA_029706805.1	*Bacillus thuringiensis*	08_38	Cry2Ab41,Cry2Aa22,Cry1Ia42,Vip3Aa79,Spp1Aa1	5	Yes	cry2A,cry2A,cry1I,vip3Aa,Spp1A	Lepidoptera;Diptera;Hemiptera;Coleoptera;Rhabditida;Blattodea;Aedes aegypti	*Helicoverpa armigera;Aedes aegypti;Diaphorina citri;Leptinotarsa decemlineata;Ostrinia furnacalis;Lobesia botrana;Plutella xylostella;Earias insulana;cotton boll weevil;Meloidogyne incognita;Helicoverpa punctigera;Spodoptera frugiperda;Helicoverpa zea;Chilo partellus;Blattella germanica;Spodoptera litura*
GCA_029706785.1	*Bacillus thuringiensis*	08_89	Cry2Ab41,Cry1Aa3,Cry1Ia42,Cry1Ca18,Cry1Da2,Cry9Ea12,Vip3Aa79,Spp1Aa1	8	Yes	cry2A,cry1A,cry1I,cry1C,cry1D,Cry9E,vip3Aa,Spp1A	Lepidoptera;Diptera;Hemiptera;Coleoptera;Rhabditida;Dipteran;Blattodea;Aedes aegypti	*Helicoverpa armigera;Aedes aegypti;Diaphorina citri;Plutella xylostella;Ostrinia furnacalis;Leucinodes orbonalis;Trichoplusia ni;Hyphantria cunea;Spodoptera frugiperda;Pieris rapae;Chilo suppressalis;Leptinotarsa decemlineata;Lobesia botrana;Earias insulana;cotton boll weevil;Meloidogyne incognita;Myzus persicae;Ostrinia*

								nubilalis;Spodoptera exigua;Bombyx mori;Spodoptera litura;Epiphyas postvittana;Agrotis exclamationis;Cydia pomonella;Helicoverpa punctigera;Helicoverpa zea;Chilo partellus;Blattella germanica
GCA_029706705.1	*Bacillus thuringiensis*	ABTS_1857	Cry1Ia42,Cry1Ca18,Cry1Da2,Cry9Ea12,Cry2Ab41,Vip3Aa79,Spp1Aa1	7	Yes	cry1I,cry1C,cry1D,Cry9E,cry2A,vip3Aa,Spp1A	Coleoptera;Lepidoptera;Rhabditida;Dipteran;Diptera;Hemiptera;Blattodea;Aedes aegypti	*Leptinotarsa decemlineata;Ostrinia furnacalis;Lobesia botrana;Plutella xylostella;Earias insulana;cotton boll weevil;Meloidogyne incognita;Myzus persicae;Aedes aegypti;Ostrinia nubilalis;Spodoptera exigua;Spodoptera frugiperda;Bombyx mori;Spodoptera litura;Epiphyas postvittana;Agrotis exclamationis;Cydia pomonella;Helicoverpa armigera;Diaphorina citri;Helicoverpa punctigera;Helicoverpa zea;Chilo partellus;Blattella germanica*
GCA_029706445.1	*Bacillus thuringiensis*	17_971	Cry1Ia42,Cry1Ca18,Cry1Da2,Cry2Ab41,Cry9Ea12,Vip3Aa79,Spp1Aa1	7	Yes	cry1I,cry1C,cry1D,cry2A,Cry9E,vip3Aa,Spp1A	Coleoptera;Lepidoptera;Rhabditida;Dipteran;Diptera;Hemiptera;Blattodea;Aedes aegypti	*Leptinotarsa decemlineata;Ostrinia furnacalis;Lobesia botrana;Plutella xylostella;Earias insulana;cotton boll weevil;Meloidogyne incognita;Myzus persicae;Aedes aegypti;Ostrinia nubilalis;Spodoptera*

								exigua;Spodoptera frugiperda;Bombyx mori;Spodoptera litura;Epiphyas postvittana;Helicoverpa armigera;Diaphorina citri;Agrotis exclamationis;Cydia pomonella;Helicoverpa punctigera;Helicoverpa zea;Chilo partellus;Blattella germanica
GCA_029706425.1	*Bacillus thuringiensis*	17_970	Cry1Ia42,Cry1Ca18,Cry1Da2,Cry2Ab41,Cry9Ea12,Vip3Aa79,Spp1Aa1	7	Yes	cry1I,cry1C,cry1D,cry2A,Cry9E,vip3Aa,Spp1A	Coleoptera;Lepidoptera;Rhabditida;Dipteran;Diptera;Hemiptera;Blattodea;Aedes aegypti	*Leptinotarsa decemlineata;Ostrinia furnacalis;Lobesia botrana;Plutella xylostella;Earias insulana;cotton boll weevil;Meloidogyne incognita;Myzus persicae;Aedes aegypti;Ostrinia nubilalis;Spodoptera exigua;Spodoptera frugiperda;Bombyx mori;Spodoptera litura;Epiphyas postvittana;Helicoverpa armigera;Diaphorina citri;Agrotis exclamationis;Cydia pomonella;Helicoverpa punctigera;Helicoverpa zea;Chilo partellus;Blattella germanica*
GCA_029706405.1	*Bacillus thuringiensis*	17_969	Cry2Ab41,Cry1Aa3,Cry1Ia42,Cry1Ca18,Cry1Da2,Cry9Ea12,Vip3Aa79,Spp1Aa1	8	Yes	cry2A,cry1A,cry1I,cry1C,cry1D,Cry9E,vip3Aa,Spp1A	Lepidoptera;Diptera;Hemiptera;Coleoptera;Rhabditida;Dipteran;Blattodea	*Helicoverpa armigera;Aedes aegypti;Diaphorina citri;Plutella xylostella;Ostrinia furnacalis;Leucinodes*

							;Aedes aegypti	*orbonalis;Trichoplusia ni;Hyphantria cunea;Spodoptera frugiperda;Pieris rapae;Chilo suppressalis;Leptinotarsa decemlineata;Lobesia botrana;Earias insulana;cotton boll weevil;Meloidogyne incognita;Myzus persicae;Ostrinia nubilalis;Spodoptera exigua;Bombyx mori;Spodoptera litura;Epiphyas postvittana;Agrotis exclamationis;Cydia pomonella;Helicoverpa punctigera;Helicoverpa zea;Chilo partellus;Blattella germanica*
GCA_029706365.1	*Bacillus thuringiensis*	17_968	Cry1Ia42,Cry1Ca18,Cry1Da2,Cry9Ea12,Cry2Ab41,Vip3Aa79,Spp1Aa1	7	Yes	cry1I,cry1C,cry1D,Cry9E,cry2A,vip3Aa,Spp1A	Coleoptera;Lepidoptera;Rhabditida;Dipteran;Diptera;Hemiptera;Blattodea;Aedes aegypti	*Leptinotarsa decemlineata;Ostrinia furnacalis;Lobesia botrana;Plutella xylostella;Earias insulana;cotton boll weevil;Meloidogyne incognita;Myzus persicae;Aedes aegypti;Ostrinia nubilalis;Spodoptera exigua;Spodoptera frugiperda;Bombyx mori;Spodoptera litura;Epiphyas postvittana;Agrotis exclamationis;Cydia pomonella;Helicoverpa armigera;Diaphorina citri;Helicoverpa*

								punctigera;Helicoverpa zea;Chilo partellus;Blattella germanica
GCA_029706305.1	*Bacillus thuringiensis*	15_915	Cry2Aa22,Cry1Ia42,Cry2Ab41,Vip3Aa79,Spp1Aa1	5	Yes	cry2A,cry1I,cry2A,vip3Aa,Spp1A	Lepidoptera;Diptera;Hemiptera;Coleoptera;Rhabditida;Blattodea;Aedes aegypti	*Helicoverpa armigera;Aedes aegypti;Diaphorina citri;Leptinotarsa decemlineata;Ostrinia furnacalis;Lobesia botrana;Plutella xylostella;Earias insulana;cotton boll weevil;Meloidogyne incognita;Helicoverpa punctigera;Spodoptera frugiperda;Helicoverpa zea;Chilo partellus;Blattella germanica;Spodoptera litura*
GCA_029706285.1	*Bacillus thuringiensis*	15_603	Cry2Ab41,Cry2Aa22,Cry1Ia42,Vip3Aa79,Spp1Aa1	5	Yes	cry2A,cry2A,cry1I,vip3Aa,Spp1A	Lepidoptera;Diptera;Hemiptera;Coleoptera;Rhabditida;Blattodea;Aedes aegypti	*Helicoverpa armigera;Aedes aegypti;Diaphorina citri;Leptinotarsa decemlineata;Ostrinia furnacalis;Lobesia botrana;Plutella xylostella;Earias insulana;cotton boll weevil;Meloidogyne incognita;Helicoverpa punctigera;Spodoptera frugiperda;Helicoverpa zea;Chilo partellus;Blattella germanica;Spodoptera litura*
GCA_029706265.1	*Bacillus thuringi*	15_605	Cry2Ab41,Cry2Aa22,Cry1Ia42,Vip3Aa79,Spp	5	Yes	cry2A,cry2A,cry1I,vip3Aa,Spp1A	Lepidoptera;Diptera;Hemiptera;Cole	*Helicoverpa armigera;Aedes aegypti;Diaphorina*

	ensis		1Aa1				optera;Rhabditida;Blattodea;Aedes aegypti	*citri;Leptinotarsa decemlineata;Ostrinia furnacalis;Lobesia botrana;Plutella xylostella;Earias insulana;cotton boll weevil;Meloidogyne incognita;Helicoverpa punctigera;Spodoptera frugiperda;Helicoverpa zea;Chilo partellus;Blattella germanica;Spodoptera litura*
GCA_029706245.1	*Bacillus thuringiensis*	15_602	Cry2Aa22,Cry1Ia42,Cry2Ab41,Vip3Aa79,Spp1Aa1	5	Yes	cry2A,cry1I,cry2A,vip3Aa,Spp1A	Lepidoptera;Diptera;Hemiptera;Coleoptera;Rhabditida;Blattodea;Aedes aegypti	*Helicoverpa armigera;Aedes aegypti;Diaphorina citri;Leptinotarsa decemlineata;Ostrinia furnacalis;Lobesia botrana;Plutella xylostella;Earias insulana;cotton boll weevil;Meloidogyne incognita;Helicoverpa punctigera;Spodoptera frugiperda;Helicoverpa zea;Chilo partellus;Blattella germanica;Spodoptera litura*
GCA_029706175.1	*Bacillus thuringiensis*	16_1122	Cry2Ab41,Cry2Aa22,Cry1Ia42,Vip3Aa79,Spp1Aa1	5	Yes	cry2A,cry2A,cry1I,vip3Aa,Spp1A	Lepidoptera;Diptera;Hemiptera;Coleoptera;Rhabditida;Blattodea;Aedes aegypti	*Helicoverpa armigera;Aedes aegypti;Diaphorina citri;Leptinotarsa decemlineata;Ostrinia furnacalis;Lobesia botrana;Plutella xylostella;Earias insulana;cotton boll*

								weevil;Meloidogyne incognita;Helicoverpa punctigera;Spodoptera frugiperda;Helicoverpa zea;Chilo partellus;Blattella germanica;Spodoptera litura
GCA_029706165.1	*Bacillus thuringiensis*	16_440	Cry2Ab41,Cry2Aa22,Cry1Ia42,Vip3Aa79,Spp1Aa1	5	Yes	cry2A,cry2A,cry1I,vip3Aa,Spp1A	Lepidoptera;Diptera;Hemiptera;Coleoptera;Rhabditida;Blattodea;Aedes aegypti	*Helicoverpa armigera;Aedes aegypti;Diaphorina citri;Leptinotarsa decemlineata;Ostrinia furnacalis;Lobesia botrana;Plutella xylostella;Earias insulana;cotton boll weevil;Meloidogyne incognita;Helicoverpa punctigera;Spodoptera frugiperda;Helicoverpa zea;Chilo partellus;Blattella germanica;Spodoptera litura*
GCA_029706085.1	*Bacillus thuringiensis*	17_1202	Cry2Ab41,Cry2Aa22,Cry1Ia42,Vip3Aa79,Spp1Aa1	5	Yes	cry2A,cry2A,cry1I,vip3Aa,Spp1A	Lepidoptera;Diptera;Hemiptera;Coleoptera;Rhabditida;Blattodea;Aedes aegypti	*Helicoverpa armigera;Aedes aegypti;Diaphorina citri;Leptinotarsa decemlineata;Ostrinia furnacalis;Lobesia botrana;Plutella xylostella;Earias insulana;cotton boll weevil;Meloidogyne incognita;Helicoverpa punctigera;Spodoptera frugiperda;Helicoverpa zea;Chilo*

								partellus;Blattella germanica;Spodoptera litura
GCA_029706055.1	*Bacillus thuringiensis*	16_898	Cry2Ab41,Cry2Aa22,Cry1Ia42,Vip3Aa79,Spp1Aa1	5	Yes	cry2A,cry2A,cry1I,vip3Aa,Spp1A	Lepidoptera;Diptera;Hemiptera;Coleoptera;Rhabditida;Blattodea;Aedes aegypti	*Helicoverpa armigera;Aedes aegypti;Diaphorina citri;Leptinotarsa decemlineata;Ostrinia furnacalis;Lobesia botrana;Plutella xylostella;Earias insulana;cotton boll weevil;Meloidogyne incognita;Helicoverpa punctigera;Spodoptera frugiperda;Helicoverpa zea;Chilo partellus;Blattella germanica;Spodoptera litura*
GCA_029706035.1	*Bacillus thuringiensis*	16_418	Cry2Ab41,Cry2Aa22,Cry1Ia42,Vip3Aa79,Spp1Aa1	5	Yes	cry2A,cry2A,cry1I,vip3Aa,Spp1A	Lepidoptera;Diptera;Hemiptera;Coleoptera;Rhabditida;Blattodea;Aedes aegypti	*Helicoverpa armigera;Aedes aegypti;Diaphorina citri;Leptinotarsa decemlineata;Ostrinia furnacalis;Lobesia botrana;Plutella xylostella;Earias insulana;cotton boll weevil;Meloidogyne incognita;Helicoverpa punctigera;Spodoptera frugiperda;Helicoverpa zea;Chilo partellus;Blattella germanica;Spodoptera litura*
GCA_029706015.1	*Bacillus thuringi*	17_263	Cry1Ia42,Cry1Ca18,Cry1Da2,Cry2Ab41,Cry9	7	Yes	cry1I,cry1C,cry1D,cry2A,Cry9E,vip3Aa,Spp	Coleoptera;Lepidoptera;Rhabditida;	*Leptinotarsa decemlineata;Ostrinia furnacalis;Lobesia botrana;Plutella*

	ensis		Ea12,Vip3Aa79,Spp1Aa1			1A	Dipteran;Diptera;Hemiptera;Blattodea;Aedes aegypti	*xylostella;Earias insulana;cotton boll weevil;Meloidogyne incognita;Myzus persicae;Aedes aegypti;Ostrinia nubilalis;Spodoptera exigua;Spodoptera frugiperda;Bombyx mori;Spodoptera litura;Epiphyas postvittana;Helicoverpa armigera;Diaphorina citri;Agrotis exclamationis;Cydia pomonella;Helicoverpa punctigera;Helicoverpa zea;Chilo partellus;Blattella germanica*
GCA_029705995.1	*Bacillus thuringiensis*	17_264	Cry1Ia42,Cry1Ca18,Cry1Da2,Cry2Ab41,Cry9Ea12,Cry1Ab9,Vip3Aa79,Spp1Aa1	8	Yes	cry1I,cry1C,cry1D,cry2A,Cry9E,cry1A,vip3Aa,Spp1A	Coleoptera;Lepidoptera;Rhabditida;Dipteran;Diptera;Hemiptera;Blattodea;Aedes aegypti	*Leptinotarsa decemlineata;Ostrinia furnacalis;Lobesia botrana;Plutella xylostella;Earias insulana;cotton boll weevil;Meloidogyne incognita;Myzus persicae;Aedes aegypti;Ostrinia nubilalis;Spodoptera exigua;Spodoptera frugiperda;Bombyx mori;Spodoptera litura;Epiphyas postvittana;Helicoverpa armigera;Diaphorina citri;Agrotis exclamationis;Cydia pomonella;Leucinodes orbonalis;Trichoplusia ni;Hyphantria cunea;Pieris rapae;Chilo*

								suppressalis;Helicoverpa punctigera;Helicoverpa zea;Chilo partellus;Blattella germanica
GCA_029705975.1	*Bacillus thuringiensis*	10_48	Cry2Ab41,Cry1Aa3,Cry1Ia42,Cry1Ca18,Cry1Da2,Cry9Ea12,Vip3Aa79,Spp1Aa1	8	Yes	cry2A,cry1A,cry1I,cry1C,cry1D,Cry9E,vip3Aa,Spp1A	Lepidoptera;Diptera;Hemiptera;Coleoptera;Rhabditida;Dipteran;Blattodea;Aedes aegypti	*Helicoverpa armigera;Aedes aegypti;Diaphorina citri;Plutella xylostella;Ostrinia furnacalis;Leucinodes orbonalis;Trichoplusia ni;Hyphantria cunea;Spodoptera frugiperda;Pieris rapae;Chilo suppressalis;Leptinotarsa decemlineata;Lobesia botrana;Earias insulana;cotton boll weevil;Meloidogyne incognita;Myzus persicae;Ostrinia nubilalis;Spodoptera exigua;Bombyx mori;Spodoptera litura;Epiphyas postvittana;Agrotis exclamationis;Cydia pomonella;Helicoverpa punctigera;Helicoverpa zea;Chilo partellus;Blattella germanica*
GCA_029705955.1	*Bacillus thuringiensis*	14_16	Cry2Ab41,Cry2Aa22,Cry1Ia42,Vip3Aa79,Spp1Aa1	5	Yes	cry2A,cry2A,cry1I,vip3Aa,Spp1A	Lepidoptera;Diptera;Hemiptera;Coleoptera;Rhabditida;Blattodea;Aedes aegypti	*Helicoverpa armigera;Aedes aegypti;Diaphorina citri;Leptinotarsa decemlineata;Ostrinia furnacalis;Lobesia botrana;Plutella xylostella;Earias insulana;cotton boll*

								weevil;Meloidogyne incognita;Helicoverpa punctigera;Spodoptera frugiperda;Helicoverpa zea;Chilo partellus;Blattella germanica;Spodoptera litura
GCA_029705935.1	*Bacillus thuringiensis*	10_49	Cry1Ia42,Cry1Ca18,Cry1Da2,Cry2Ab41,Cry9Ea12,Vip3Aa79,Spp1Aa1	7	Yes	cry1I,cry1C,cry1D,cry2A,Cry9E,vip3Aa,Spp1A	Coleoptera;Lepidoptera;Rhabditida;Dipteran;Diptera;Hemiptera;Blattodea;Aedes aegypti	*Leptinotarsa decemlineata;Ostrinia furnacalis;Lobesia botrana;Plutella xylostella;Earias insulana;cotton boll weevil;Meloidogyne incognita;Myzus persicae;Aedes aegypti;Ostrinia nubilalis;Spodoptera exigua;Spodoptera frugiperda;Bombyx mori;Spodoptera litura;Epiphyas postvittana;Helicoverpa armigera;Diaphorina citri;Agrotis exclamationis;Cydia pomonella;Helicoverpa punctigera;Helicoverpa zea;Chilo partellus;Blattella germanica*
GCA_029705915.1	*Bacillus thuringiensis*	10_47	Cry1Ia42,Cry1Ca18,Cry1Da2,Cry2Ab41,Cry9Ea12,Vip3Aa79,Spp1Aa1	7	Yes	cry1I,cry1C,cry1D,cry2A,Cry9E,vip3Aa,Spp1A	Coleoptera;Lepidoptera;Rhabditida;Dipteran;Diptera;Hemiptera;Blattodea;Aedes aegypti	*Leptinotarsa decemlineata;Ostrinia furnacalis;Lobesia botrana;Plutella xylostella;Earias insulana;cotton boll weevil;Meloidogyne incognita;Myzus persicae;Aedes aegypti;Ostrinia nubilalis;Spodoptera exigua;Spodoptera*

								frugiperda;Bombyx mori;Spodoptera litura;Epiphyas postvittana;Helicoverpa armigera;Diaphorina citri;Agrotis exclamationis;Cydia pomonella;Helicoverpa punctigera;Helicoverpa zea;Chilo partellus;Blattella germanica
GCA_029705895.1	*Bacillus thuringiensis*	10_50	Cry2Ab41,Cry1Aa3,Cry1Ia42,Cry1Ca18,Cry1Da2,Cry9Ea12,Vip3Aa79,Spp1Aa1	8	Yes	cry2A,cry1A,cry1I,cry1C,cry1D,Cry9E,vip3Aa,Spp1A	Lepidoptera;Diptera;Hemiptera;Coleoptera;Rhabditida;Dipteran;Blattodea;Aedes aegypti	*Helicoverpa armigera;Aedes aegypti;Diaphorina citri;Plutella xylostella;Ostrinia furnacalis;Leucinodes orbonalis;Trichoplusia ni;Hyphantria cunea;Spodoptera frugiperda;Pieris rapae;Chilo suppressalis;Leptinotarsa decemlineata;Lobesia botrana;Earias insulana;cotton boll weevil;Meloidogyne incognita;Myzus persicae;Ostrinia nubilalis;Spodoptera exigua;Bombyx mori;Spodoptera litura;Epiphyas postvittana;Agrotis exclamationis;Cydia pomonella;Helicoverpa punctigera;Helicoverpa zea;Chilo partellus;Blattella germanica*
GCA_029	*Bacillus*	12_17	Cry2Ab41,Cry2Aa22,C	5	Yes	cry2A,cry2A,cry1I,vip	Lepidoptera;Dipte	*Helicoverpa armigera;Aedes*

705795.1	*thuringiensis*		ry1Ia42,Vip3Aa79,Spp1Aa1			3Aa,Spp1A	ra;Hemiptera;Coleoptera;Rhabditida;Blattodea;Aedes aegypti	*aegypti;Diaphorina citri;Leptinotarsa decemlineata;Ostrinia furnacalis;Lobesia botrana;Plutella xylostella;Earias insulana;cotton boll weevil;Meloidogyne incognita;Helicoverpa punctigera;Spodoptera frugiperda;Helicoverpa zea;Chilo partellus;Blattella germanica;Spodoptera litura*
GCA_029705735.1	*Bacillus thuringiensis*	17_265	Cry1Ia42,Cry1Ca18,Cry1Da2,Cry2Ab41,Cry9Ea12,Cry1Ab9,Vip3Aa79,Spp1Aa1	8	Yes	cry1I,cry1C,cry1D,cry2A,Cry9E,cry1A,vip3Aa,Spp1A	Coleoptera;Lepidoptera;Rhabditida;Dipteran;Diptera;Hemiptera;Blattodea;Aedes aegypti	*Leptinotarsa decemlineata;Ostrinia furnacalis;Lobesia botrana;Plutella xylostella;Earias insulana;cotton boll weevil;Meloidogyne incognita;Myzus persicae;Aedes aegypti;Ostrinia nubilalis;Spodoptera exigua;Spodoptera frugiperda;Bombyx mori;Spodoptera litura;Epiphyas postvittana;Helicoverpa armigera;Diaphorina citri;Agrotis exclamationis;Cydia pomonella;Leucinodes orbonalis;Trichoplusia ni;Hyphantria cunea;Pieris rapae;Chilo suppressalis;Helicoverpa punctigera;Helicoverpa zea;Chilo*

								partellus;Blattella germanica
GCA_029705715.1	*Bacillus thuringiensis*	17_429	Cry1Ab24,Cry1Nb1,Cry1Ja1,Cry1Id2,Cry2Ad1,Cry1Da3,Vip3Af3,Spp1Aa1,Vpa2Ac2,Vpb1Ca1	10	No	cry1A,cry1N,cry1J,cry1I,cry2A,cry1D,vip3Af,Spp1A	Lepidoptera;Coleoptera;Rhabditida;Diptera;Hemiptera;Blattodea	*Plutella xylostella;Ostrinia furnacalis;Helicoverpa armigera;Leucinodes orbonalis;Trichoplusia ni;Hyphantria cunea;Spodoptera frugiperda;Pieris rapae;Chilo suppressalis;Colaphellus bowringi;Manduca sexta;Lobesia botrana;Diabrotica undecimpunctata howardi;Leptinotarsa decemlineata;Earias insulana;cotton boll weevil;Meloidogyne incognita;Aedes aegypti;Diaphorina citri;Ostrinia nubilalis;Spodoptera exigua;Bombyx mori;Spodoptera litura;Epiphyas postvittana;Agrotis ipsilon;Blattella germanica*
GCA_029705695.1	*Bacillus thuringiensis*	17_334	Cry2Ab41,Cry2Aa22,Cry1Ia42,Vip3Aa79,Spp1Aa1	5	Yes	cry2A,cry2A,cry1I,vip3Aa,Spp1A	Lepidoptera;Diptera;Hemiptera;Coleoptera;Rhabditida;Blattodea;Aedes aegypti	*Helicoverpa armigera;Aedes aegypti;Diaphorina citri;Leptinotarsa decemlineata;Ostrinia furnacalis;Lobesia botrana;Plutella xylostella;Earias insulana;cotton boll weevil;Meloidogyne incognita;Helicoverpa punctigera;Spodoptera frugiperda;Helicoverpa zea;Chilo partellus;Blattella*

								germanica;Spodoptera litura
GCA_029705455.1	*Bacillus thuringiensis*	17_430	Cry2Ab41,Cry2Aa22,Cry1Ia42,Vip3Aa79,Spp1Aa1	5	Yes	cry2A,cry2A,cry1I,vip3Aa,Spp1A	Lepidoptera;Diptera;Hemiptera;Coleoptera;Rhabditida;Blattodea;Aedes aegypti	*Helicoverpa armigera;Aedes aegypti;Diaphorina citri;Leptinotarsa decemlineata;Ostrinia furnacalis;Lobesia botrana;Plutella xylostella;Earias insulana;cotton boll weevil;Meloidogyne incognita;Helicoverpa punctigera;Spodoptera frugiperda;Helicoverpa zea;Chilo partellus;Blattella germanica;Spodoptera litura*
GCA_029705435.1	*Bacillus thuringiensis*	10_46	Cry1Ia42,Cry1Ca18,Cry1Da2,Cry2Ab41,Cry9Ea12,Vip3Aa79,Spp1Aa1	7	Yes	cry1I,cry1C,cry1D,cry2A,Cry9E,vip3Aa,Spp1A	Coleoptera;Lepidoptera;Rhabditida;Dipteran;Diptera;Hemiptera;Blattodea;Aedes aegypti	*Leptinotarsa decemlineata;Ostrinia furnacalis;Lobesia botrana;Plutella xylostella;Earias insulana;cotton boll weevil;Meloidogyne incognita;Myzus persicae;Aedes aegypti;Ostrinia nubilalis;Spodoptera exigua;Spodoptera frugiperda;Bombyx mori;Spodoptera litura;Epiphyas postvittana;Helicoverpa armigera;Diaphorina citri;Agrotis exclamationis;Cydia pomonella;Helicoverpa punctigera;Helicoverpa zea;Chilo partellus;Blattella germanica*

GCA_029705415.1	*Bacillus thuringiensis*	17_529	Cry2Ab41,Cry2Aa22,Cry1Ia42,Vip3Aa79,Spp1Aa1	5	Yes	cry2A,cry2A,cry1I,vip3Aa,Spp1A	Lepidoptera;Diptera;Hemiptera;Coleoptera;Rhabditida;Blattodea;Aedes aegypti	*Helicoverpa armigera;Aedes aegypti;Diaphorina citri;Leptinotarsa decemlineata;Ostrinia furnacalis;Lobesia botrana;Plutella xylostella;Earias insulana;cotton boll weevil;Meloidogyne incognita;Helicoverpa punctigera;Spodoptera frugiperda;Helicoverpa zea;Chilo partellus;Blattella germanica;Spodoptera litura*
GCA_029705395.1	*Bacillus thuringiensis*	07_30	Cry2Ab41,Cry2Aa22,Cry1Ia42,Vip3Aa79,Spp1Aa1	5	Yes	cry2A,cry2A,cry1I,vip3Aa,Spp1A	Lepidoptera;Diptera;Hemiptera;Coleoptera;Rhabditida;Blattodea;Aedes aegypti	*Helicoverpa armigera;Aedes aegypti;Diaphorina citri;Leptinotarsa decemlineata;Ostrinia furnacalis;Lobesia botrana;Plutella xylostella;Earias insulana;cotton boll weevil;Meloidogyne incognita;Helicoverpa punctigera;Spodoptera frugiperda;Helicoverpa zea;Chilo partellus;Blattella germanica;Spodoptera litura*
GCA_029705375.1	*Bacillus thuringiensis*	10_02	Cry1Ca18,Cry1Da2,Cry2Ab41,Cry9Ea12,Cry1Ia42,Vip3Aa79,Spp1Aa1	7	Yes	cry1C,cry1D,cry2A,Cry9E,cry1I,vip3Aa,Spp1A	Lepidoptera;Diptera n;Diptera;Hemiptera;Coleoptera;Rhabditida;Blattodea;	*Plutella xylostella;Myzus persicae;Aedes aegypti;Ostrinia nubilalis;Spodoptera exigua;Spodoptera*

							Aedes aegypti	*frugiperda;Bombyx mori;Spodoptera litura;Epiphyas postvittana;Helicoverpa armigera;Diaphorina citri;Agrotis exclamationis;Cydia pomonella;Leptinotarsa decemlineata;Ostrinia furnacalis;Lobesia botrana;Earias insulana;cotton boll weevil;Meloidogyne incognita;Helicoverpa punctigera;Helicoverpa zea;Chilo partellus;Blattella germanica*
GCA_029705355.1	*Bacillus thuringiensis*	17_528	Cry1Ia42,Cry1Ca18,Cry1Da2,Cry2Ab41,Cry9Ea12,Vip3Aa79,Spp1Aa1	7	Yes	cry1I,cry1C,cry1D,cry2A,Cry9E,vip3Aa,Spp1A	Coleoptera;Lepidoptera;Rhabditida;Dipteran;Diptera;Hemiptera;Blattodea;Aedes aegypti	*Leptinotarsa decemlineata;Ostrinia furnacalis;Lobesia botrana;Plutella xylostella;Earias insulana;cotton boll weevil;Meloidogyne incognita;Myzus persicae;Aedes aegypti;Ostrinia nubilalis;Spodoptera exigua;Spodoptera frugiperda;Bombyx mori;Spodoptera litura;Epiphyas postvittana;Helicoverpa armigera;Diaphorina citri;Agrotis exclamationis;Cydia pomonella;Helicoverpa punctigera;Helicoverpa zea;Chilo partellus;Blattella germanica*

GCA_029705295.1	*Bacillus thuringiensis*	10_01	Cry1Ca18,Cry1Da2,Cry2Ab41,Cry1Ia42,Cry9Ea12,Vip3Aa79,Spp1Aa1	7	Yes	cry1C,cry1D,cry2A,cry1I,Cry9E,vip3Aa,Spp1A	Lepidoptera;Diptera;Diptera;Hemiptera;Coleoptera;Rhabditida;Blattodea;Aedes aegypti	*Plutella xylostella;Myzus persicae;Aedes aegypti;Ostrinia nubilalis;Spodoptera exigua;Spodoptera frugiperda;Bombyx mori;Spodoptera litura;Epiphyas postvittana;Helicoverpa armigera;Diaphorina citri;Leptinotarsa decemlineata;Ostrinia furnacalis;Lobesia botrana;Earias insulana;cotton boll weevil;Meloidogyne incognita;Agrotis exclamationis;Cydia pomonella;Helicoverpa punctigera;Helicoverpa zea;Chilo partellus;Blattella germanica*
GCA_029705275.1	*Bacillus thuringiensis*	09_69	Cry2Ab41,Cry2Aa22,Cry1Ia42,Vip3Aa79,Spp1Aa1	5	Yes	cry2A,cry2A,cry1I,vip3Aa,Spp1A	Lepidoptera;Diptera;Hemiptera;Coleoptera;Rhabditida;Blattodea;Aedes aegypti	*Helicoverpa armigera;Aedes aegypti;Diaphorina citri;Leptinotarsa decemlineata;Ostrinia furnacalis;Lobesia botrana;Plutella xylostella;Earias insulana;cotton boll weevil;Meloidogyne incognita;Helicoverpa punctigera;Spodoptera frugiperda;Helicoverpa zea;Chilo*

								partellus;Blattella germanica;Spodoptera litura
GCA_029705235.1	*Bacillus thuringiensis*	09_68	Cry2Ab41,Cry2Aa22,Cry1Ia42,Vip3Aa79,Spp1Aa1	5	Yes	cry2A,cry2A,cry1I,vip3Aa,Spp1A	Lepidoptera;Diptera;Hemiptera;Coleoptera;Rhabditida;Blattodea;Aedes aegypti	*Helicoverpa armigera;Aedes aegypti;Diaphorina citri;Leptinotarsa decemlineata;Ostrinia furnacalis;Lobesia botrana;Plutella xylostella;Earias insulana;cotton boll weevil;Meloidogyne incognita;Helicoverpa punctigera;Spodoptera frugiperda;Helicoverpa zea;Chilo partellus;Blattella germanica;Spodoptera litura*
GCA_029705215.1	*Bacillus thuringiensis*	10_05	Cry1Ca18,Cry1Da2,Cry2Ab41,Cry9Ea12,Cry1Ia42,Vip3Aa79,Spp1Aa1	7	Yes	cry1C,cry1D,cry2A,Cry9E,cry1I,vip3Aa,Spp1A	Lepidoptera;Diptera;Diptera;Hemiptera;Coleoptera;Rhabditida;Blattodea;Aedes aegypti	*Plutella xylostella;Myzus persicae;Aedes aegypti;Ostrinia nubilalis;Spodoptera exigua;Spodoptera frugiperda;Bombyx mori;Spodoptera litura;Epiphyas postvittana;Helicoverpa armigera;Diaphorina citri;Agrotis exclamationis;Cydia pomonella;Leptinotarsa decemlineata;Ostrinia furnacalis;Lobesia botrana;Earias insulana;cotton boll weevil;Meloidogyne*

								incognita;Helicoverpa punctigera;Helicoverpa zea;Chilo partellus;Blattella germanica
GCA_029705195.1	*Bacillus thuringiensis*	10_04	Cry1Ca18,Cry1Da2,Cry2Ab41,Cry1Ia42,Cry9Ea12,Vip3Aa79,Spp1Aa1	7	Yes	cry1C,cry1D,cry2A,cry1I,Cry9E,vip3Aa,Spp1A	Lepidoptera;Diptera;Diptera;Hemiptera;Coleoptera;Rhabditida;Blattodea;Aedes aegypti	*Plutella xylostella;Myzus persicae;Aedes aegypti;Ostrinia nubilalis;Spodoptera exigua;Spodoptera frugiperda;Bombyx mori;Spodoptera litura;Epiphyas postvittana;Helicoverpa armigera;Diaphorina citri;Leptinotarsa decemlineata;Ostrinia furnacalis;Lobesia botrana;Earias insulana;cotton boll weevil;Meloidogyne incognita;Agrotis exclamationis;Cydia pomonella;Helicoverpa punctigera;Helicoverpa zea;Chilo partellus;Blattella germanica*
GCA_029705175.1	*Bacillus thuringiensis*	08_126	Cry2Ab41,Cry1Aa3,Cry1Ia42,Cry1Ca18,Cry1Da2,Cry9Ea12,Vip3Aa79,Spp1Aa1	8	Yes	cry2A,cry1A,cry1I,cry1C,cry1D,Cry9E,vip3Aa,Spp1A	Lepidoptera;Diptera;Hemiptera;Coleoptera;Rhabditida;Dipteran;Blattodea;Aedes aegypti	*Helicoverpa armigera;Aedes aegypti;Diaphorina citri;Plutella xylostella;Ostrinia furnacalis;Leucinodes orbonalis;Trichoplusia ni;Hyphantria cunea;Spodoptera frugiperda;Pieris rapae;Chilo*

								suppressalis;Leptinotarsa decemlineata;Lobesia botrana;Earias insulana;cotton boll weevil;Meloidogyne incognita;Myzus persicae;Ostrinia nubilalis;Spodoptera exigua;Bombyx mori;Spodoptera litura;Epiphyas postvittana;Agrotis exclamationis;Cydia pomonella;Helicoverpa punctigera;Helicoverpa zea;Chilo partellus;Blattella germanica
GCA_029705155.1	*Bacillus thuringiensis*	10_03	Cry1Ca18,Cry1Da2,Cry2Ab41,Cry9Ea12,Cry1Ia42,Vip3Aa79,Spp1Aa1	7	Yes	cry1C,cry1D,cry2A,Cry9E,cry1I,vip3Aa,Spp1A	Lepidoptera;Diptera;Diptera;Hemiptera;Coleoptera;Rhabditida;Blattodea;Aedes aegypti	*Plutella xylostella;Myzus persicae;Aedes aegypti;Ostrinia nubilalis;Spodoptera exigua;Spodoptera frugiperda;Bombyx mori;Spodoptera litura;Epiphyas postvittana;Helicoverpa armigera;Diaphorina citri;Agrotis exclamationis;Cydia pomonella;Leptinotarsa decemlineata;Ostrinia furnacalis;Lobesia botrana;Earias insulana;cotton boll weevil;Meloidogyne incognita;Helicoverpa punctigera;Helicoverpa zea;Chilo*

								partellus;Blattella germanica
GCA_029705135.1	*Bacillus thuringiensis*	07_31	Cry2Ab41,Cry2Aa22,Cry1Ia42,Vip3Aa79,Spp1Aa1	5	Yes	cry2A,cry2A,cry1I,vip3Aa,Spp1A	Lepidoptera;Diptera;Hemiptera;Coleoptera;Rhabditida;Blattodea;Aedes aegypti	*Helicoverpa armigera;Aedes aegypti;Diaphorina citri;Leptinotarsa decemlineata;Ostrinia furnacalis;Lobesia botrana;Plutella xylostella;Earias insulana;cotton boll weevil;Meloidogyne incognita;Helicoverpa punctigera;Spodoptera frugiperda;Helicoverpa zea;Chilo partellus;Blattella germanica;Spodoptera litura*
GCA_029705115.1	*Bacillus thuringiensis*	07_29	Cry2Ab41,Cry2Aa22,Cry1Ia42,Vip3Aa79,Spp1Aa1	5	Yes	cry2A,cry2A,cry1I,vip3Aa,Spp1A	Lepidoptera;Diptera;Hemiptera;Coleoptera;Rhabditida;Blattodea;Aedes aegypti	*Helicoverpa armigera;Aedes aegypti;Diaphorina citri;Leptinotarsa decemlineata;Ostrinia furnacalis;Lobesia botrana;Plutella xylostella;Earias insulana;cotton boll weevil;Meloidogyne incognita;Helicoverpa punctigera;Spodoptera frugiperda;Helicoverpa zea;Chilo partellus;Blattella germanica;Spodoptera litura*
GCA_029705095.1	*Bacillus thuringiensis*	08_125	Cry1Ia42,Cry1Ca18,Cry1Da2,Cry9Ea12,Cry2Ab41,Vip3Aa79,Spp1A	7	Yes	cry1I,cry1C,cry1D,Cry9E,cry2A,vip3Aa,Spp1A	Coleoptera;Lepidoptera;Rhabditida;Dipteran;Diptera;	*Leptinotarsa decemlineata;Ostrinia furnacalis;Lobesia botrana;Plutella xylostella;Earias insulana;cotton boll*

			a1				Hemiptera;Blattodea;Aedes aegypti	*weevil;Meloidogyne incognita;Myzus persicae;Aedes aegypti;Ostrinia nubilalis;Spodoptera exigua;Spodoptera frugiperda;Bombyx mori;Spodoptera litura;Epiphyas postvittana;Agrotis exclamationis;Cydia pomonella;Helicoverpa armigera;Diaphorina citri;Helicoverpa punctigera;Helicoverpa zea;Chilo partellus;Blattella germanica*
GCA_029705075.1	*Bacillus thuringiensis*	08_122	Cry1Ia42,Cry1Ca18,Cry1Da2,Cry2Ab41,Cry9Ea12,Vip3Aa79,Spp1Aa1	7	Yes	cry1I,cry1C,cry1D,cry2A,Cry9E,vip3Aa,Spp1A	Coleoptera;Lepidoptera;Rhabditida;Dipteran;Diptera;Hemiptera;Blattodea;Aedes aegypti	*Leptinotarsa decemlineata;Ostrinia furnacalis;Lobesia botrana;Plutella xylostella;Earias insulana;cotton boll weevil;Meloidogyne incognita;Myzus persicae;Aedes aegypti;Ostrinia nubilalis;Spodoptera exigua;Spodoptera frugiperda;Bombyx mori;Spodoptera litura;Epiphyas postvittana;Helicoverpa armigera;Diaphorina citri;Agrotis exclamationis;Cydia pomonella;Helicoverpa punctigera;Helicoverpa zea;Chilo partellus;Blattella germanica*
GCA_029	*Bacillus*	07_32	Cry2Ab41,Cry1Ia42,Vi	4	Yes	cry2A,cry1I,vip3Aa,S	Lepidoptera;Dipte	*Helicoverpa armigera;Aedes*

705055.1	*thuringiensis*		p3Aa79,Spp1Aa1			pp1A	ra;Hemiptera;Coleoptera;Rhabditida;Blattodea;Aedes aegypti	*aegypti;Diaphorina citri;Leptinotarsa decemlineata;Ostrinia furnacalis;Lobesia botrana;Plutella xylostella;Earias insulana;cotton boll weevil;Meloidogyne incognita;Helicoverpa punctigera;Spodoptera frugiperda;Helicoverpa zea;Chilo partellus;Blattella germanica;Spodoptera litura*
GCA_029705035.1	*Bacillus thuringiensis*	08_121	Cry1Ia42,Cry1Ca18,Cry1Da2,Cry2Ab41,Cry9Ea12,Vip3Aa79,Spp1Aa1	7	Yes	cry1I,cry1C,cry1D,cry2A,Cry9E,vip3Aa,Spp1A	Coleoptera;Lepidoptera;Rhabditida;Dipteran;Diptera;Hemiptera;Blattodea;Aedes aegypti	*Leptinotarsa decemlineata;Ostrinia furnacalis;Lobesia botrana;Plutella xylostella;Earias insulana;cotton boll weevil;Meloidogyne incognita;Myzus persicae;Aedes aegypti;Ostrinia nubilalis;Spodoptera exigua;Spodoptera frugiperda;Bombyx mori;Spodoptera litura;Epiphyas postvittana;Helicoverpa armigera;Diaphorina citri;Agrotis exclamationis;Cydia pomonella;Helicoverpa punctigera;Helicoverpa zea;Chilo partellus;Blattella germanica*
GCA_029705015.1	*Bacillus thuringi*	08_124	Cry2Ab41,Cry1Aa3,Cry1Ia42,Cry1Ca18,Cry1	8	Yes	cry2A,cry1A,cry1I,cry1C,cry1D,Cry9E,vip3	Lepidoptera;Diptera;Hemiptera;Cole	*Helicoverpa armigera;Aedes aegypti;Diaphorina citri;Plutella*

	ensis		Da2,Cry9Ea12,Vip3Aa79,Spp1Aa1			Aa,Spp1A	optera;Rhabditida;Dipteran;Blattodea;Aedes aegypti	*xylostella;Ostrinia furnacalis;Leucinodes orbonalis;Trichoplusia ni;Hyphantria cunea;Spodoptera frugiperda;Pieris rapae;Chilo suppressalis;Leptinotarsa decemlineata;Lobesia botrana;Earias insulana;cotton boll weevil;Meloidogyne incognita;Myzus persicae;Ostrinia nubilalis;Spodoptera exigua;Bombyx mori;Spodoptera litura;Epiphyas postvittana;Agrotis exclamationis;Cydia pomonella;Helicoverpa punctigera;Helicoverpa zea;Chilo partellus;Blattella germanica*
GCA_029704995.1	*Bacillus thuringiensis*	08_123	Cry1Ia42,Cry1Ca18,Cry1Da2,Cry2Ab41,Cry9Ea12,Vip3Aa79,Spp1Aa1	7	Yes	cry1I,cry1C,cry1D,cry2A,Cry9E,vip3Aa,Spp1A	Coleoptera;Lepidoptera;Rhabditida;Dipteran;Diptera;Hemiptera;Blattodea;Aedes aegypti	*Leptinotarsa decemlineata;Ostrinia furnacalis;Lobesia botrana;Plutella xylostella;Earias insulana;cotton boll weevil;Meloidogyne incognita;Myzus persicae;Aedes aegypti;Ostrinia nubilalis;Spodoptera exigua;Spodoptera frugiperda;Bombyx mori;Spodoptera litura;Epiphyas postvittana;Helicoverpa armigera;Diaphorina citri;Agrotis*

								exclamationis;Cydia pomonella;Helicoverpa punctigera;Helicoverpa zea;Chilo partellus;Blattella germanica
GCA_029704975.1	*Bacillus thuringiensis*	14_361	Cry2Ab41,Cry2Aa22,Cry1Ia42,Cry1Ab9,Vip3Aa79,Spp1Aa1	6	Yes	cry2A,cry2A,cry1I,cry1A,vip3Aa,Spp1A	Lepidoptera;Diptera;Hemiptera;Coleoptera;Rhabditida;Blattodea;Aedes aegypti	*Helicoverpa armigera;Aedes aegypti;Diaphorina citri;Leptinotarsa decemlineata;Ostrinia furnacalis;Lobesia botrana;Plutella xylostella;Earias insulana;cotton boll weevil;Meloidogyne incognita;Leucinodes orbonalis;Trichoplusia ni;Hyphantria cunea;Spodoptera frugiperda;Pieris rapae;Chilo suppressalis;Helicoverpa punctigera;Helicoverpa zea;Chilo partellus;Blattella germanica;Spodoptera litura*
GCA_029704925.1	*Bacillus thuringiensis*	14_311	Cry1Aa45,Cry1Ia42,Cry1Ca18,Cry1Da2,Cry2Ab41,Cry1Ab28,Cry9Ea12,Vip3Aa79,Spp1Aa1	9	Yes	cry1A,cry1I,cry1C,cry1D,cry2A,cry1A,Cry9E,vip3Aa,Spp1A	Lepidoptera;Coleoptera;Rhabditida;Dipteran;Diptera;Hemiptera;Blattodea;Aedes aegypti	*Plutella xylostella;Ostrinia furnacalis;Helicoverpa armigera;Leucinodes orbonalis;Trichoplusia ni;Hyphantria cunea;Spodoptera frugiperda;Pieris rapae;Chilo suppressalis;Leptinotarsa decemlineata;Lobesia botrana;Earias insulana;cotton boll*

								weevil;Meloidogyne incognita;Myzus persicae;Aedes aegypti;Ostrinia nubilalis;Spodoptera exigua;Bombyx mori;Spodoptera litura;Epiphyas postvittana;Diaphorina citri;Agrotis exclamationis;Cydia pomonella;Helicoverpa punctigera;Helicoverpa zea;Chilo partellus;Blattella germanica
GCA_029704915.1	*Bacillus thuringiensis*	14_313	Cry1Aa45,Cry1Ia42,Cry1Ca18,Cry1Da2,Cry2Ab41,Cry9Ea12,Cry1Ab28,Vip3Aa79,Spp1Aa1	9	Yes	cry1A,cry1I,cry1C,cry1D,cry2A,Cry9E,cry1A,vip3Aa,Spp1A	Lepidoptera;Coleoptera;Rhabditida;Dipteran;Diptera;Hemiptera;Blattodea;Aedes aegypti	*Plutella xylostella;Ostrinia furnacalis;Helicoverpa armigera;Leucinodes orbonalis;Trichoplusia ni;Hyphantria cunea;Spodoptera frugiperda;Pieris rapae;Chilo suppressalis;Leptinotarsa decemlineata;Lobesia botrana;Earias insulana;cotton boll weevil;Meloidogyne incognita;Myzus persicae;Aedes aegypti;Ostrinia nubilalis;Spodoptera exigua;Bombyx mori;Spodoptera litura;Epiphyas postvittana;Diaphorina citri;Agrotis exclamationis;Cydia pomonella;Helicoverpa punctigera;Helicoverpa zea;Chilo partellus;Blattella germanica*
GCA_029	*Bacillus*	14_31	Cry1Ca18,Cry1Da2,Cry	8	Yes	cry1C,cry1D,Cry9E,cr	Lepidoptera;Dipte	*Plutella xylostella;Myzus*

704895.1	*thuringiensis*	2	9Ea12,Cry2Ab41,Cry1Ia42,Cry1Ab28,Vip3Aa79,Spp1Aa1			y2A,cry1I,cry1A,vip3Aa,Spp1A	ran;Diptera;Hemiptera;Coleoptera;Rhabditida;Blattodea;Aedes aegypti	*persicae;Aedes aegypti;Ostrinia nubilalis;Spodoptera exigua;Spodoptera frugiperda;Bombyx mori;Spodoptera litura;Epiphyas postvittana;Agrotis exclamationis;Cydia pomonella;Helicoverpa armigera;Diaphorina citri;Leptinotarsa decemlineata;Ostrinia furnacalis;Lobesia botrana;Earias insulana;cotton boll weevil;Meloidogyne incognita;Leucinodes orbonalis;Trichoplusia ni;Hyphantria cunea;Pieris rapae;Chilo suppressalis;Helicoverpa punctigera;Helicoverpa zea;Chilo partellus;Blattella germanica*
GCA_029704875.1	*Bacillus thuringiensis*	14_310	Cry1Aa45,Cry1Ia42,Cry1Ca18,Cry1Da2,Cry2Ab41,Cry1Ab28,Cry9Ea12,Vip3Aa79,Spp1Aa1	9	Yes	cry1A,cry1I,cry1C,cry1D,cry2A,cry1A,Cry9E,vip3Aa,Spp1A	Lepidoptera;Coleoptera;Rhabditida;Dipteran;Diptera;Hemiptera;Blattodea;Aedes aegypti	*Plutella xylostella;Ostrinia furnacalis;Helicoverpa armigera;Leucinodes orbonalis;Trichoplusia ni;Hyphantria cunea;Spodoptera frugiperda;Pieris rapae;Chilo suppressalis;Leptinotarsa decemlineata;Lobesia botrana;Earias insulana;cotton boll*

								weevil;Meloidogyne incognita;Myzus persicae;Aedes aegypti;Ostrinia nubilalis;Spodoptera exigua;Bombyx mori;Spodoptera litura;Epiphyas postvittana;Diaphorina citri;Agrotis exclamationis;Cydia pomonella;Helicoverpa punctigera;Helicoverpa zea;Chilo partellus;Blattella germanica
GCA_029704855.1	*Bacillus thuringiensis*	14_309	Cry1Ia42,Cry1Ca18,Cry1Da2,Cry2Ab41,Cry1Ab28,Cry9Ea12,Vip3Aa79,Spp1Aa1	8	Yes	cry1I,cry1C,cry1D,cry2A,cry1A,Cry9E,vip3Aa,Spp1A	Coleoptera;Lepidoptera;Rhabditida;Dipteran;Diptera;Hemiptera;Blattodea;Aedes aegypti	*Leptinotarsa decemlineata;Ostrinia furnacalis;Lobesia botrana;Plutella xylostella;Earias insulana;cotton boll weevil;Meloidogyne incognita;Myzus persicae;Aedes aegypti;Ostrinia nubilalis;Spodoptera exigua;Spodoptera frugiperda;Bombyx mori;Spodoptera litura;Epiphyas postvittana;Helicoverpa armigera;Diaphorina citri;Leucinodes orbonalis;Trichoplusia ni;Hyphantria cunea;Pieris rapae;Chilo suppressalis;Agrotis exclamationis;Cydia pomonella;Helicoverpa punctigera;Helicoverpa zea;Chilo partellus;Blattella germanica*

GCA_029704835.1	*Bacillus thuringiensis*	14_265	Cry1Ca18,Cry1Ia42,Cry1Aa3,Cry2Ab41,Cry9Ea12,Vip3Aa79,Spp1Aa1	7	Yes	cry1C,cry1I,cry1A,cry2A,Cry9E,vip3Aa,Spp1A	Lepidoptera;Diptera n;Coleoptera;Rhabditida;Diptera;Hemiptera;Blattodea;Aedes aegypti	*Plutella xylostella;Myzus persicae;Aedes aegypti;Leptinotarsa decemlineata;Ostrinia furnacalis;Lobesia botrana;Earias insulana;cotton boll weevil;Meloidogyne incognita;Helicoverpa armigera;Leucinodes orbonalis;Trichoplusia ni;Hyphantria cunea;Spodoptera frugiperda;Pieris rapae;Chilo suppressalis;Diaphorina citri;Agrotis exclamationis;Cydia pomonella;Helicoverpa punctigera;Helicoverpa zea;Chilo partellus;Blattella germanica;Spodoptera litura*
GCA_029704815.1	*Bacillus thuringiensis*	14_264	Cry2Ab41,Cry1Aa3,Cry1Ia42,Cry1Ca18,Cry1Da2,Cry9Ea12,Vip3Aa79,Spp1Aa1	8	Yes	cry2A,cry1A,cry1I,cry1C,cry1D,Cry9E,vip3Aa,Spp1A	Lepidoptera;Diptera;Hemiptera;Coleoptera;Rhabditida;Dipteran;Blattodea;Aedes aegypti	*Helicoverpa armigera;Aedes aegypti;Diaphorina citri;Plutella xylostella;Ostrinia furnacalis;Leucinodes orbonalis;Trichoplusia ni;Hyphantria cunea;Spodoptera frugiperda;Pieris rapae;Chilo suppressalis;Leptinotarsa decemlineata;Lobesia botrana;Earias insulana;cotton boll weevil;Meloidogyne incognita;Myzus persicae;Ostrinia*

								nubilalis;Spodoptera exigua;Bombyx mori;Spodoptera litura;Epiphyas postvittana;Agrotis exclamationis;Cydia pomonella;Helicoverpa punctigera;Helicoverpa zea;Chilo partellus;Blattella germanica
GCA_029704795.1	*Bacillus thuringiensis*	14_262	Cry1Ia42,Cry1Ca18,Cry1Da2,Cry9Ea12,Cry2Ab41,Vip3Aa79,Spp1Aa1	7	Yes	cry1I,cry1C,cry1D,Cry9E,cry2A,vip3Aa,Spp1A	Coleoptera;Lepidoptera;Rhabditida;Dipteran;Diptera;Hemiptera;Blattodea;Aedes aegypti	*Leptinotarsa decemlineata;Ostrinia furnacalis;Lobesia botrana;Plutella xylostella;Earias insulana;cotton boll weevil;Meloidogyne incognita;Myzus persicae;Aedes aegypti;Ostrinia nubilalis;Spodoptera exigua;Spodoptera frugiperda;Bombyx mori;Spodoptera litura;Epiphyas postvittana;Agrotis exclamationis;Cydia pomonella;Helicoverpa armigera;Diaphorina citri;Helicoverpa punctigera;Helicoverpa zea;Chilo partellus;Blattella germanica*
GCA_029704775.1	*Bacillus thuringiensis*	14_176	Cry2Ab41,Cry1Aa3,Cry1Ia42,Cry1Ca18,Cry1Da2,Cry9Ea12,Vip3Aa79,Spp1Aa1	8	Yes	cry2A,cry1A,cry1I,cry1C,cry1D,Cry9E,vip3Aa,Spp1A	Lepidoptera;Diptera;Hemiptera;Coleoptera;Rhabditida;Dipteran;Blattodea;Aedes aegypti	*Helicoverpa armigera;Aedes aegypti;Diaphorina citri;Plutella xylostella;Ostrinia furnacalis;Leucinodes orbonalis;Trichoplusia ni;Hyphantria cunea;Spodoptera frugiperda;Pieris*

								rapae;Chilo suppressalis;Leptinotarsa decemlineata;Lobesia botrana;Earias insulana;cotton boll weevil;Meloidogyne incognita;Myzus persicae;Ostrinia nubilalis;Spodoptera exigua;Bombyx mori;Spodoptera litura;Epiphyas postvittana;Agrotis exclamationis;Cydia pomonella;Helicoverpa punctigera;Helicoverpa zea;Chilo partellus;Blattella germanica
GCA_029704755.1	*Bacillus thuringiensis*	14_263	Cry1Aa45,Cry1Ia42,Cry1Ca18,Cry1Da2,Cry2Ab41,Cry9Ea12,Cry1Ab28,Vip3Aa79,Spp1Aa1	9	Yes	cry1A,cry1I,cry1C,cry1D,cry2A,Cry9E,cry1A,vip3Aa,Spp1A	Lepidoptera;Coleoptera;Rhabditida;Dipteran;Diptera;Hemiptera;Blattodea;Aedes aegypti	*Plutella xylostella;Ostrinia furnacalis;Helicoverpa armigera;Leucinodes orbonalis;Trichoplusia ni;Hyphantria cunea;Spodoptera frugiperda;Pieris rapae;Chilo suppressalis;Leptinotarsa decemlineata;Lobesia botrana;Earias insulana;cotton boll weevil;Meloidogyne incognita;Myzus persicae;Aedes aegypti;Ostrinia nubilalis;Spodoptera exigua;Bombyx mori;Spodoptera litura;Epiphyas postvittana;Diaphorina citri;Agrotis exclamationis;Cydia*

								pomonella;Helicoverpa punctigera;Helicoverpa zea;Chilo partellus;Blattella germanica
GCA_029704285.1	*Bacillus thuringiensis*	14_178	Cry2Ab41,Cry1Aa3,Cry1Ia42,Cry1Ca18,Cry1Da2,Cry9Ea12,Vip3Aa79,Spp1Aa1	8	Yes	cry2A,cry1A,cry1I,cry1C,cry1D,Cry9E,vip3Aa,Spp1A	Lepidoptera;Diptera;Hemiptera;Coleoptera;Rhabditida;Dipteran;Blattodea;Aedes aegypti	*Helicoverpa armigera;Aedes aegypti;Diaphorina citri;Plutella xylostella;Ostrinia furnacalis;Leucinodes orbonalis;Trichoplusia ni;Hyphantria cunea;Spodoptera frugiperda;Pieris rapae;Chilo suppressalis;Leptinotarsa decemlineata;Lobesia botrana;Earias insulana;cotton boll weevil;Meloidogyne incognita;Myzus persicae;Ostrinia nubilalis;Spodoptera exigua;Bombyx mori;Spodoptera litura;Epiphyas postvittana;Agrotis exclamationis;Cydia pomonella;Helicoverpa punctigera;Helicoverpa zea;Chilo partellus;Blattella germanica*
GCA_029704265.1	*Bacillus thuringiensis*	14_179	Cry2Ab41,Cry1Aa3,Cry1Ia42,Cry1Ca18,Cry1Da2,Cry9Ea12,Vip3Aa79,Spp1Aa1	8	Yes	cry2A,cry1A,cry1I,cry1C,cry1D,Cry9E,vip3Aa,Spp1A	Lepidoptera;Diptera;Hemiptera;Coleoptera;Rhabditida;Dipteran;Blattodea;Aedes aegypti	*Helicoverpa armigera;Aedes aegypti;Diaphorina citri;Plutella xylostella;Ostrinia furnacalis;Leucinodes orbonalis;Trichoplusia ni;Hyphantria cunea;Spodoptera frugiperda;Pieris*

								rapae;Chilo suppressalis;Leptinotarsa decemlineata;Lobesia botrana;Earias insulana;cotton boll weevil;Meloidogyne incognita;Myzus persicae;Ostrinia nubilalis;Spodoptera exigua;Bombyx mori;Spodoptera litura;Epiphyas postvittana;Agrotis exclamationis;Cydia pomonella;Helicoverpa punctigera;Helicoverpa zea;Chilo partellus;Blattella germanica
GCA_029704205.1	*Bacillus thuringiensis*	14_22	Cry2Ab41,Cry2Aa22,Cry1Ia42,Vip3Aa79,Spp1Aa1	5	Yes	cry2A,cry2A,cry1I,vip3Aa,Spp1A	Lepidoptera;Diptera;Hemiptera;Coleoptera;Rhabditida;Blattodea;Aedes aegypti	*Helicoverpa armigera;Aedes aegypti;Diaphorina citri;Leptinotarsa decemlineata;Ostrinia furnacalis;Lobesia botrana;Plutella xylostella;Earias insulana;cotton boll weevil;Meloidogyne incognita;Helicoverpa punctigera;Spodoptera frugiperda;Helicoverpa zea;Chilo partellus;Blattella germanica;Spodoptera litura*
GCA_029704185.1	*Bacillus thuringiensis*	15_482	Cry2Ab41,Cry2Aa22,Cry1Ia42,Vip3Aa79,Spp1Aa1	5	Yes	cry2A,cry2A,cry1I,vip3Aa,Spp1A	Lepidoptera;Diptera;Hemiptera;Coleoptera;Rhabditida;	*Helicoverpa armigera;Aedes aegypti;Diaphorina citri;Leptinotarsa*

							Blattodea;Aedes aegypti	*decemlineata;Ostrinia furnacalis;Lobesia botrana;Plutella xylostella;Earias insulana;cotton boll weevil;Meloidogyne incognita;Helicoverpa punctigera;Spodoptera frugiperda;Helicoverpa zea;Chilo partellus;Blattella germanica;Spodoptera litura*
GCA_029704145.1	*Bacillus thuringiensis*	NB_176	Cry3Aa6,Mpp23Aa1,Spp1Aa1,Vpa2Ah1,Vpb1Ae1,Xpp37Aa1	6	No	Mpp23A+Xpp37A,Vpa2Ah + Vpb1Ae,Cry3A,Spp1A	Coleoptera;Hemiptera;Lepidoptera;Blattodea	*Cylas puncticollis;Anthonomus grandis;Aphis gossypii;Leptinotarsa decemlineata;Tenebrio molitor;Hypera postica;Macrosiphum euphorbiae;Spodoptera litura;Blattella germanica*
GCA_029704125.1	*Bacillus thuringiensis*	15_1331	Cry2Ab41,Cry2Aa22,Cry1Ia42,Cry1Ab9,Vip3Aa79,Spp1Aa1	6	Yes	cry2A,cry2A,cry1I,cry1A,vip3Aa,Spp1A	Lepidoptera;Diptera;Hemiptera;Coleoptera;Rhabditida;Blattodea;Aedes aegypti	*Helicoverpa armigera;Aedes aegypti;Diaphorina citri;Leptinotarsa decemlineata;Ostrinia furnacalis;Lobesia botrana;Plutella xylostella;Earias insulana;cotton boll weevil;Meloidogyne incognita;Leucinodes orbonalis;Trichoplusia ni;Hyphantria cunea;Spodoptera frugiperda;Pieris rapae;Chilo suppressalis;Helicoverpa*

								punctigera;Helicoverpa zea;Chilo partellus;Blattella germanica;Spodoptera litura
GCA_029704105.1	*Bacillus thuringiensis*	15_599	Cry2Ab41,Cry2Aa22,Cry1Ia42,Vip3Aa79,Spp1Aa1	5	Yes	cry2A,cry2A,cry1I,vip3Aa,Spp1A	Lepidoptera;Diptera;Hemiptera;Coleoptera;Rhabditida;Blattodea;Aedes aegypti	*Helicoverpa armigera;Aedes aegypti;Diaphorina citri;Leptinotarsa decemlineata;Ostrinia furnacalis;Lobesia botrana;Plutella xylostella;Earias insulana;cotton boll weevil;Meloidogyne incognita;Helicoverpa punctigera;Spodoptera frugiperda;Helicoverpa zea;Chilo partellus;Blattella germanica;Spodoptera litura*
GCA_029704075.1	*Bacillus thuringiensis*	15_1009	Cry2Ab41,Cry2Aa22,Cry1Ia42,Cry1Ab9,Vip3Aa79,Spp1Aa1	6	Yes	cry2A,cry2A,cry1I,cry1A,vip3Aa,Spp1A	Lepidoptera;Diptera;Hemiptera;Coleoptera;Rhabditida;Blattodea;Aedes aegypti	*Helicoverpa armigera;Aedes aegypti;Diaphorina citri;Leptinotarsa decemlineata;Ostrinia furnacalis;Lobesia botrana;Plutella xylostella;Earias insulana;cotton boll weevil;Meloidogyne incognita;Leucinodes orbonalis;Trichoplusia ni;Hyphantria cunea;Spodoptera frugiperda;Pieris rapae;Chilo suppressalis;Helicoverpa punctigera;Helicoverpa zea;Chilo*

								partellus;Blattella germanica;Spodoptera litura
GCA_029704055.1	*Bacillus thuringiensis*	15_598	Cry2Ab41,Cry2Aa22,Cry1Ia42,Vip3Aa79,Spp1Aa1	5	Yes	cry2A,cry2A,cry1I,vip3Aa,Spp1A	Lepidoptera;Diptera;Hemiptera;Coleoptera;Rhabditida;Blattodea;Aedes aegypti	*Helicoverpa armigera;Aedes aegypti;Diaphorina citri;Leptinotarsa decemlineata;Ostrinia furnacalis;Lobesia botrana;Plutella xylostella;Earias insulana;cotton boll weevil;Meloidogyne incognita;Helicoverpa punctigera;Spodoptera frugiperda;Helicoverpa zea;Chilo partellus;Blattella germanica;Spodoptera litura*
GCA_029704015.1	*Bacillus thuringiensis*	15_1011	Cry2Ab41,Cry2Aa22,Cry1Ia42,Vip3Aa79,Spp1Aa1	5	Yes	cry2A,cry2A,cry1I,vip3Aa,Spp1A	Lepidoptera;Diptera;Hemiptera;Coleoptera;Rhabditida;Blattodea;Aedes aegypti	*Helicoverpa armigera;Aedes aegypti;Diaphorina citri;Leptinotarsa decemlineata;Ostrinia furnacalis;Lobesia botrana;Plutella xylostella;Earias insulana;cotton boll weevil;Meloidogyne incognita;Helicoverpa punctigera;Spodoptera frugiperda;Helicoverpa zea;Chilo partellus;Blattella germanica;Spodoptera litura*
GCA_029703995.1	*Bacillus thuringi*	15_601	Cry2Ab41,Cry2Aa22,Cry1Ia42,Vip3Aa79,Spp	5	Yes	cry2A,cry2A,cry1I,vip3Aa,Spp1A	Lepidoptera;Diptera;Hemiptera;Cole	*Helicoverpa armigera;Aedes aegypti;Diaphorina*

	ensis		1Aa1				optera;Rhabditida;Blattodea;Aedes aegypti	*citri;Leptinotarsa decemlineata;Ostrinia furnacalis;Lobesia botrana;Plutella xylostella;Earias insulana;cotton boll weevil;Meloidogyne incognita;Helicoverpa punctigera;Spodoptera frugiperda;Helicoverpa zea;Chilo partellus;Blattella germanica;Spodoptera litura*
GCA_029703955.1	*Bacillus thuringiensis*	CIP53137	Cry1Ab9,Cry1Ba8,Mpp5Ab1,Spp1Aa1,Vpa2Bb4	5	Yes	cry1A,cry1B,Mpp5A,Spp1A	Lepidoptera;Hemiptera;Diptera;Coleoptera;Blattodea	*Plutella xylostella;Ostrinia furnacalis;Helicoverpa armigera;Leucinodes orbonalis;Trichoplusia ni;Hyphantria cunea;Spodoptera frugiperda;Pieris rapae;Chilo suppressalis;Ostrinia nubilalis;Maruca vitrata;Manduca sexta;cotton boll weevil;Colaphellus bowringi;Leptinotarsa decemlineata;Spodoptera litura;Blattella germanica*
GCA_029703935.1	*Bacillus thuringiensis*	15_600	Cry2Ab41,Cry2Aa22,Cry1Ia42,Cry1Ab28,Vip3Aa79,Spp1Aa1	6	Yes	cry2A,cry2A,cry1I,cry1A,vip3Aa,Spp1A	Lepidoptera;Diptera;Hemiptera;Coleoptera;Rhabditida;Blattodea;Aedes aegypti	*Helicoverpa armigera;Aedes aegypti;Diaphorina citri;Leptinotarsa decemlineata;Ostrinia furnacalis;Lobesia botrana;Plutella xylostella;Earias insulana;cotton boll weevil;Meloidogyne*

								incognita;Leucinodes orbonalis;Trichoplusia ni;Hyphantria cunea;Spodoptera frugiperda;Pieris rapae;Chilo suppressalis;Helicoverpa punctigera;Helicoverpa zea;Chilo partellus;Blattella germanica;Spodoptera litura
GCA_029703915.1	*Bacillus thuringiensis*	14_374	Cry1Ca18,Cry1Da2,Cry2Ab41,Cry1Ia42,Cry9Ea12,Vip3Aa79,Spp1Aa1	7	Yes	cry1C,cry1D,cry2A,cry1I,Cry9E,vip3Aa,Spp1A	Lepidoptera;Diptera;Diptera;Hemiptera;Coleoptera;Rhabditida;Blattodea;Aedes aegypti	*Plutella xylostella;Myzus persicae;Aedes aegypti;Ostrinia nubilalis;Spodoptera exigua;Spodoptera frugiperda;Bombyx mori;Spodoptera litura;Epiphyas postvittana;Helicoverpa armigera;Diaphorina citri;Leptinotarsa decemlineata;Ostrinia furnacalis;Lobesia botrana;Earias insulana;cotton boll weevil;Meloidogyne incognita;Agrotis exclamationis;Cydia pomonella;Helicoverpa punctigera;Helicoverpa zea;Chilo partellus;Blattella germanica*
GCA_029703895.1	*Bacillus thuringi*	GC_91	Cry1Ca18,Cry1Da2,Cry2Ab41,Cry1Ia42,Cry9E	7	Yes	cry1C,cry1D,cry2A,cry1I,Cry9E,vip3Aa,Sp	Lepidoptera;Diptera;Diptera;Hemip	*Plutella xylostella;Myzus persicae;Aedes aegypti;Ostrinia*

	ensis		a12,Vip3Aa79,Spp1Aa1			p1A	tera;Coleoptera;Rhabditida;Blattodea;Aedes aegypti	*nubilalis;Spodoptera exigua;Spodoptera frugiperda;Bombyx mori;Spodoptera litura;Epiphyas postvittana;Helicoverpa armigera;Diaphorina citri;Leptinotarsa decemlineata;Ostrinia furnacalis;Lobesia botrana;Earias insulana;cotton boll weevil;Meloidogyne incognita;Agrotis exclamationis;Cydia pomonella;Helicoverpa punctigera;Helicoverpa zea;Chilo partellus;Blattella germanica*
GCA_029703875.1	*Bacillus thuringiensis*	14_388	Cry2Ab41,Cry2Aa22,Cry1Ia42,Vip3Aa79,Spp1Aa1	5	Yes	cry2A,cry2A,cry1I,vip3Aa,Spp1A	Lepidoptera;Diptera;Hemiptera;Coleoptera;Rhabditida;Blattodea;Aedes aegypti	*Helicoverpa armigera;Aedes aegypti;Diaphorina citri;Leptinotarsa decemlineata;Ostrinia furnacalis;Lobesia botrana;Plutella xylostella;Earias insulana;cotton boll weevil;Meloidogyne incognita;Helicoverpa punctigera;Spodoptera frugiperda;Helicoverpa zea;Chilo partellus;Blattella germanica;Spodoptera litura*

GCA_029703855.1	*Bacillus thuringiensis*	14_372	Cry2Ab41,Cry1Aa3,Cry1Ia42,Cry1Ca18,Cry1Da2,Cry9Ea12,Vip3Aa79,Spp1Aa1	8	Yes	cry2A,cry1A,cry1I,cry1C,cry1D,Cry9E,vip3Aa,Spp1A	Lepidoptera;Diptera;Hemiptera;Coleoptera;Rhabditida;Dipteran;Blattodea;Aedes aegypti	*Helicoverpa armigera;Aedes aegypti;Diaphorina citri;Plutella xylostella;Ostrinia furnacalis;Leucinodes orbonalis;Trichoplusia ni;Hyphantria cunea;Spodoptera frugiperda;Pieris rapae;Chilo suppressalis;Leptinotarsa decemlineata;Lobesia botrana;Earias insulana;cotton boll weevil;Meloidogyne incognita;Myzus persicae;Ostrinia nubilalis;Spodoptera exigua;Bombyx mori;Spodoptera litura;Epiphyas postvittana;Agrotis exclamationis;Cydia pomonella;Helicoverpa punctigera;Helicoverpa zea;Chilo partellus;Blattella germanica*
GCA_029703835.1	*Bacillus thuringiensis*	14_364	Cry1Ca18,Cry1Da2,Cry2Ab41,Cry1Ia42,Cry9Ea12,Vip3Aa79,Spp1Aa1	7	Yes	cry1C,cry1D,cry2A,cry1I,Cry9E,vip3Aa,Spp1A	Lepidoptera;Dipteran;Diptera;Hemiptera;Coleoptera;Rhabditida;Blattodea;Aedes aegypti	*Plutella xylostella;Myzus persicae;Aedes aegypti;Ostrinia nubilalis;Spodoptera exigua;Spodoptera frugiperda;Bombyx mori;Spodoptera litura;Epiphyas postvittana;Helicoverpa armigera;Diaphorina citri;Leptinotarsa*

								decemlineata;Ostrinia furnacalis;Lobesia botrana;Earias insulana;cotton boll weevil;Meloidogyne incognita;Agrotis exclamationis;Cydia pomonella;Helicoverpa punctigera;Helicoverpa zea;Chilo partellus;Blattella germanica
GCA_029703815.1	*Bacillus thuringiensis*	14_370	Cry1Ca18,Cry1Da2,Cry2Ab41,Cry1Ia42,Cry9Ea12,Vip3Aa79,Spp1Aa1	7	Yes	cry1C,cry1D,cry2A,cry1I,Cry9E,vip3Aa,Spp1A	Lepidoptera;Diptera;Diptera;Hemiptera;Coleoptera;Rhabditida;Blattodea;Aedes aegypti	*Plutella xylostella;Myzus persicae;Aedes aegypti;Ostrinia nubilalis;Spodoptera exigua;Spodoptera frugiperda;Bombyx mori;Spodoptera litura;Epiphyas postvittana;Helicoverpa armigera;Diaphorina citri;Leptinotarsa decemlineata;Ostrinia furnacalis;Lobesia botrana;Earias insulana;cotton boll weevil;Meloidogyne incognita;Agrotis exclamationis;Cydia pomonella;Helicoverpa punctigera;Helicoverpa zea;Chilo partellus;Blattella germanica*
GCA_029	*Bacillus*	14_37	Cry2Ab41,Cry1Aa3,Cr	8	Yes	cry2A,cry1A,cry1I,cry	Lepidoptera;Dipte	*Helicoverpa armigera;Aedes*

703795.1	*thuringiensis*	1	y1Ia42,Cry1Ca18,Cry1Da2,Cry9Ea12,Vip3Aa79,Spp1Aa1			1C,cry1D,Cry9E,vip3Aa,Spp1A	ra;Hemiptera;Coleoptera;Rhabditida;Dipteran;Blattodea;Aedes aegypti	*aegypti;Diaphorina citri;Plutella xylostella;Ostrinia furnacalis;Leucinodes orbonalis;Trichoplusia ni;Hyphantria cunea;Spodoptera frugiperda;Pieris rapae;Chilo suppressalis;Leptinotarsa decemlineata;Lobesia botrana;Earias insulana;cotton boll weevil;Meloidogyne incognita;Myzus persicae;Ostrinia nubilalis;Spodoptera exigua;Bombyx mori;Spodoptera litura;Epiphyas postvittana;Agrotis exclamationis;Cydia pomonella;Helicoverpa punctigera;Helicoverpa zea;Chilo partellus;Blattella germanica*
GCA_029703775.1	*Bacillus thuringiensis*	14_373	Cry1Ia42,Cry1Ca18,Cry1Da2,Cry2Ab41,Cry9Ea12,Vip3Aa79,Spp1Aa1	7	Yes	cry1I,cry1C,cry1D,cry2A,Cry9E,vip3Aa,Spp1A	Coleoptera;Lepidoptera;Rhabditida;Dipteran;Diptera;Hemiptera;Blattodea;Aedes aegypti	*Leptinotarsa decemlineata;Ostrinia furnacalis;Lobesia botrana;Plutella xylostella;Earias insulana;cotton boll weevil;Meloidogyne incognita;Myzus persicae;Aedes aegypti;Ostrinia nubilalis;Spodoptera exigua;Spodoptera frugiperda;Bombyx mori;Spodoptera litura;Epiphyas postvittana;Helicoverpa*

								armigera;Diaphorina citri;Agrotis exclamationis;Cydia pomonella;Helicoverpa punctigera;Helicoverpa zea;Chilo partellus;Blattella germanica
GCA_029703735.1	*Bacillus thuringiensis*	15_1007	Cry2Ab41,Cry2Aa22,Cry1Ia42,Cry1Ab9,Vip3Aa79,Spp1Aa1	6	Yes	cry2A,cry2A,cry1I,cry1A,vip3Aa,Spp1A	Lepidoptera;Diptera;Hemiptera;Coleoptera;Rhabditida;Blattodea;Aedes aegypti	*Helicoverpa armigera;Aedes aegypti;Diaphorina citri;Leptinotarsa decemlineata;Ostrinia furnacalis;Lobesia botrana;Plutella xylostella;Earias insulana;cotton boll weevil;Meloidogyne incognita;Leucinodes orbonalis;Trichoplusia ni;Hyphantria cunea;Spodoptera frugiperda;Pieris rapae;Chilo suppressalis;Helicoverpa punctigera;Helicoverpa zea;Chilo partellus;Blattella germanica;Spodoptera litura*
GCA_029703715.1	*Bacillus thuringiensis*	15_1008	Cry2Ab41,Cry2Aa22,Cry1Ia42,Cry1Ab9,Vip3Aa79,Spp1Aa1	6	Yes	cry2A,cry2A,cry1I,cry1A,vip3Aa,Spp1A	Lepidoptera;Diptera;Hemiptera;Coleoptera;Rhabditida;Blattodea;Aedes aegypti	*Helicoverpa armigera;Aedes aegypti;Diaphorina citri;Leptinotarsa decemlineata;Ostrinia furnacalis;Lobesia botrana;Plutella xylostella;Earias insulana;cotton boll weevil;Meloidogyne incognita;Leucinodes*

								orbonalis;Trichoplusia ni;Hyphantria cunea;Spodoptera frugiperda;Pieris rapae;Chilo suppressalis;Helicoverpa punctigera;Helicoverpa zea;Chilo partellus;Blattella germanica;Spodoptera litura
GCA_029703675.1	*Bacillus thuringiensis*	17_619	Cry1Ca18,Cry1Da2,Cry2Ab41,Cry1Ia42,Cry9Ea12,Vip3Aa79,Spp1Aa1	7	Yes	cry1C,cry1D,cry2A,cry1I,Cry9E,vip3Aa,Spp1A	Lepidoptera;Diptera;Diptera;Hemiptera;Coleoptera;Rhabditida;Blattodea;Aedes aegypti	*Plutella xylostella;Myzus persicae;Aedes aegypti;Ostrinia nubilalis;Spodoptera exigua;Spodoptera frugiperda;Bombyx mori;Spodoptera litura;Epiphyas postvittana;Helicoverpa armigera;Diaphorina citri;Leptinotarsa decemlineata;Ostrinia furnacalis;Lobesia botrana;Earias insulana;cotton boll weevil;Meloidogyne incognita;Agrotis exclamationis;Cydia pomonella;Helicoverpa punctigera;Helicoverpa zea;Chilo partellus;Blattella germanica*
GCA_029703655.1	*Bacillus thuringiensis*	17_531	Cry2Ab41,Cry2Aa22,Cry1Ia42,Vip3Aa79,Spp1Aa1	5	Yes	cry2A,cry2A,cry1I,vip3Aa,Spp1A	Lepidoptera;Diptera;Hemiptera;Coleoptera;Rhabditida;	*Helicoverpa armigera;Aedes aegypti;Diaphorina citri;Leptinotarsa*

							Blattodea;Aedes aegypti	*decemlineata;Ostrinia furnacalis;Lobesia botrana;Plutella xylostella;Earias insulana;cotton boll weevil;Meloidogyne incognita;Helicoverpa punctigera;Spodoptera frugiperda;Helicoverpa zea;Chilo partellus;Blattella germanica;Spodoptera litura*
GCA_029703615.1	*Bacillus thuringiensis*	17_530	Cry2Ab41,Cry2Aa22,Cry1Ia42,Vip3Aa79,Spp1Aa1	5	Yes	cry2A,cry2A,cry1I,vip3Aa,Spp1A	Lepidoptera;Diptera;Hemiptera;Coleoptera;Rhabditida;Blattodea;Aedes aegypti	*Helicoverpa armigera;Aedes aegypti;Diaphorina citri;Leptinotarsa decemlineata;Ostrinia furnacalis;Lobesia botrana;Plutella xylostella;Earias insulana;cotton boll weevil;Meloidogyne incognita;Helicoverpa punctigera;Spodoptera frugiperda;Helicoverpa zea;Chilo partellus;Blattella germanica;Spodoptera litura*
GCA_029703595.1	*Bacillus thuringiensis*	17_622	Cry1Da2,Cry1Ca18,Cry2Ab41,Cry1Ia42,Cry9Ea12,Vip3Aa79,Spp1Aa1	7	Yes	cry1D,cry1C,cry2A,cry1I,Cry9E,vip3Aa,Spp1A	Lepidoptera;Diptera;Diptera;Hemiptera;Coleoptera;Rhabditida;Blattodea;Aedes aegypti	*Ostrinia nubilalis;Plutella xylostella;Spodoptera exigua;Spodoptera frugiperda;Bombyx mori;Spodoptera litura;Epiphyas postvittana;Myzus persicae;Aedes aegypti;Helicoverpa armigera;Diaphorina*

								citri;Leptinotarsa decemlineata;Ostrinia furnacalis;Lobesia botrana;Earias insulana;cotton boll weevil;Meloidogyne incognita;Agrotis exclamationis;Cydia pomonella;Helicoverpa punctigera;Helicoverpa zea;Chilo partellus;Blattella germanica
GCA_029703575.1	*Bacillus thuringiensis*	17_620	Cry1Da2,Cry1Ca18,Cry2Ab41,Cry1Ia42,Cry9Ea12,Vip3Aa79,Spp1Aa1	7	Yes	cry1D,cry1C,cry2A,cry1I,Cry9E,vip3Aa,Spp1A	Lepidoptera;Diptera n;Diptera;Hemiptera;Coleoptera;Rhabditida;Blattodea;Aedes aegypti	*Ostrinia nubilalis;Plutella xylostella;Spodoptera exigua;Spodoptera frugiperda;Bombyx mori;Spodoptera litura;Epiphyas postvittana;Myzus persicae;Aedes aegypti;Helicoverpa armigera;Diaphorina citri;Leptinotarsa decemlineata;Ostrinia furnacalis;Lobesia botrana;Earias insulana;cotton boll weevil;Meloidogyne incognita;Agrotis exclamationis;Cydia pomonella;Helicoverpa punctigera;Helicoverpa zea;Chilo partellus;Blattella germanica*
GCA_029	*Bacillus*	17_62	Cry1Ca18,Cry1Da2,Cry	7	Yes	cry1C,cry1D,cry2A,cr	Lepidoptera;Dipte	*Plutella xylostella;Myzus*

703555.1	*thuringiensis*	1	2Ab41,Cry1Ia42,Cry9Ea12,Vip3Aa79,Spp1Aa1			y1I,Cry9E,vip3Aa,Spp1A	ran;Diptera;Hemiptera;Coleoptera;Rhabditida;Blattodea;Aedes aegypti	*persicae;Aedes aegypti;Ostrinia nubilalis;Spodoptera exigua;Spodoptera frugiperda;Bombyx mori;Spodoptera litura;Epiphyas postvittana;Helicoverpa armigera;Diaphorina citri;Leptinotarsa decemlineata;Ostrinia furnacalis;Lobesia botrana;Earias insulana;cotton boll weevil;Meloidogyne incognita;Agrotis exclamationis;Cydia pomonella;Helicoverpa punctigera;Helicoverpa zea;Chilo partellus;Blattella germanica*
GCA_029703535.1	*Bacillus thuringiensis*	17_623	Cry1Ca18,Cry1Da2,Cry2Ab41,Cry1Ia42,Cry9Ea12,Vip3Aa79,Spp1Aa1	7	Yes	cry1C,cry1D,cry2A,cry1I,Cry9E,vip3Aa,Spp1A	Lepidoptera;Diptera;Diptera;Hemiptera;Coleoptera;Rhabditida;Blattodea;Aedes aegypti	*Plutella xylostella;Myzus persicae;Aedes aegypti;Ostrinia nubilalis;Spodoptera exigua;Spodoptera frugiperda;Bombyx mori;Spodoptera litura;Epiphyas postvittana;Helicoverpa armigera;Diaphorina citri;Leptinotarsa decemlineata;Ostrinia furnacalis;Lobesia botrana;Earias*

								insulana;cotton boll weevil;Meloidogyne incognita;Agrotis exclamationis;Cydia pomonella;Helicoverpa punctigera;Helicoverpa zea;Chilo partellus;Blattella germanica
GCA_029703515.1	*Bacillus thuringiensis*	17_885	Cry2Ab41,Cry2Aa22,Cry1Ia42,Vip3Aa79,Spp1Aa1	5	Yes	cry2A,cry2A,cry1I,vip3Aa,Spp1A	Lepidoptera;Diptera;Hemiptera;Coleoptera;Rhabditida;Blattodea;Aedes aegypti	*Helicoverpa armigera;Aedes aegypti;Diaphorina citri;Leptinotarsa decemlineata;Ostrinia furnacalis;Lobesia botrana;Plutella xylostella;Earias insulana;cotton boll weevil;Meloidogyne incognita;Helicoverpa punctigera;Spodoptera frugiperda;Helicoverpa zea;Chilo partellus;Blattella germanica;Spodoptera litura*
GCA_029699345.1	*Bacillus thuringiensis*	MicBio3	Spp1Aa1	1	No	Spp1A	Lepidoptera;Blattodea	*Spodoptera litura;Blattella germanica*
GCA_029699325.1	*Bacillus thuringiensis*	MicBio39	Cry7Aa2,Spp1Aa1	2	No	cry7A,Spp1A	Coleoptera;Lepidoptera;Blattodea	*Leptinotarsa decemlineata;Henosepilachna vigintiomaculata;Acanthoscelides obtectus;Henosepilachna vigintioctomaculata;Cylas puncticollis;Spodoptera*

								litura;Blattella germanica
GCA_029696385.1	*Bacillus thuringiensis*	16SBCL437	Cry2Aa22,Cry1Ia42,Cry2Ab41,Cry1Ab28,Vip3Aa79,Spp1Aa1	6	Yes	cry2A,cry1I,cry2A,cry1A,vip3Aa,Spp1A	Lepidoptera;Diptera;Hemiptera;Coleoptera;Rhabditida;Blattodea;Aedes aegypti	*Helicoverpa armigera;Aedes aegypti;Diaphorina citri;Leptinotarsa decemlineata;Ostrinia furnacalis;Lobesia botrana;Plutella xylostella;Earias insulana;cotton boll weevil;Meloidogyne incognita;Leucinodes orbonalis;Trichoplusia ni;Hyphantria cunea;Spodoptera frugiperda;Pieris rapae;Chilo suppressalis;Helicoverpa punctigera;Helicoverpa zea;Chilo partellus;Blattella germanica;Spodoptera litura*
GCA_029696085.1	*Bacillus thuringiensis*	10CEB12	Cry2Ab41,Cry2Aa22,Cry1Ia42,Vip3Aa79,Spp1Aa1	5	Yes	cry2A,cry2A,cry1I,vip3Aa,Spp1A	Lepidoptera;Diptera;Hemiptera;Coleoptera;Rhabditida;Blattodea;Aedes aegypti	*Helicoverpa armigera;Aedes aegypti;Diaphorina citri;Leptinotarsa decemlineata;Ostrinia furnacalis;Lobesia botrana;Plutella xylostella;Earias insulana;cotton boll weevil;Meloidogyne incognita;Helicoverpa punctigera;Spodoptera frugiperda;Helicoverpa zea;Chilo partellus;Blattella germanica;Spodoptera litura*

GCA_029696065.1	*Bacillus thuringiensis*	10CEB11	Cry2Ab41,Cry2Aa22,Cry1Ia42,Cry1Ab9,Vip3Aa79,Spp1Aa1	6	Yes	cry2A,cry2A,cry1I,cry1A,vip3Aa,Spp1A	Lepidoptera;Diptera;Hemiptera;Coleoptera;Rhabditida;Blattodea;Aedes aegypti	*Helicoverpa armigera;Aedes aegypti;Diaphorina citri;Leptinotarsa decemlineata;Ostrinia furnacalis;Lobesia botrana;Plutella xylostella;Earias insulana;cotton boll weevil;Meloidogyne incognita;Leucinodes orbonalis;Trichoplusia ni;Hyphantria cunea;Spodoptera frugiperda;Pieris rapae;Chilo suppressalis;Helicoverpa punctigera;Helicoverpa zea;Chilo partellus;Blattella germanica;Spodoptera litura*
GCA_029692725.1	*Bacillus thuringiensis*	CH188	Cry11Aa3,Cry10Aa4,Cyt1Aa11,Cyt2Ba17,Cyt1Ca1,Mpp60Ba3,Mpp60Aa3	7	No	cry11A,cry10A,cyt1A,cyt2B,Mpp60B,Mpp60A	Diptera;Coleoptera;Hemiptera;Lepidoptera	*Aedes aegypti;Anopheles albimanus;Culex quinquefasciatu;Culex pipiens;cotton boll weevil;Culex quinquefasciatus;Culex pipien;Diaphorina citri;Plutella xylostella;Anopheles stephensi;Anopheles gambiae;Bradysia difformis*
GCA_025291795.1	*Bacillus thuringiensis*	BHI1-bin.2	Cry1Hb2,Cry1Bb1,Cry1Ab24,Cry1Nb1,Cry1Ja1,Cry1Id2,Cry1Fb4,Cry1Ka1,Cry1Ib5,Cry1Da3	15	No	cry1H,cry1B,cry1A,cry1N,cry1J,cry1I,cry1F,cry1K,cry1I,cry1D,vip3Af,vip3Ag,Spp1A	Lepidoptera;Hemiptera;Diptera;Coleoptera;Rhabditida;Blattodea	*Spodoptera exigua;Ostrinia furnacalis;Plutella xylostella;Ostrinia nubilalis;Spodoptera*

			,Vip3Af3,Vip3Ag6,Spp1Aa1,Vpa2Ac2,Vpb1Ca1					*frugiperda;Maruca vitrata;Manduca sexta;cotton boll weevil;Helicoverpa armigera;Leucinodes orbonalis;Trichoplusia ni;Hyphantria cunea;Pieris rapae;Chilo suppressalis;Colaphellus bowringi;Lobesia botrana;Diabrotica undecimpunctata howardi;Leptinotarsa decemlineata;Earias insulana;Meloidogyne incognita;Agrotis ipsilon;Diabrotica virgifera virgifera;Artogeia rapae;Bombyx mori;Spodoptera litura;Epiphyas postvittana;Spodoptera littoralis;Chrysodeixis chalcites;Blattella germanica*

Table S6. Distribution of PisP-like proteins in *Bacillus*-related and other bacteria associated with insect or nematode hosts.

Bacterial species	RefSeq genomes analyzed (N)	Close PisP homologs<br>≥80% coverage, ≥80% identity, conserved domain	PisP-like proteins<br>≥80% coverage, ≥25% identity, conserved domain	Distant PisP-like candidates<br>≥50% coverage, ≥25% identity, conserved domain
Bacillus cereus	2,501	1,325 (53.0%)	2,487 (99.4%)	2,487 (99.4%)
Bacillus subtilis	1,268	0 (0%)	13 (1.0%)	1,256 (99.1%)
Bacillus mycoides	266	0 (0%)	263 (98.9%)	264 (99.2%)
Bacillus cytotoxicus	87	0 (0%)	45 (51.7%)	45 (51.7%)
Bacillus pumilus	385	0 (0%)	90 (23.4%)	385 (100%)
Bacillus toyonensis	378	9 (2.4%)	376 (99.5%)	376 (99.5%)
Bacillus velezensis	912	0 (0%)	4 (0.4%)	904 (99.1%)
Bacillus	279	2 (0.7%)	278 (99.6%)	278 (99.6%)

wiedmannii				
Brevibacillus laterosporus	72	0 (0%)	72 (100%)	72 (100%)
Lysinibacillus sphaericus	53	0 (0%)	0 (0%)	49 (92.5%)
Paenibacillus larvae	80	0 (0%)	0 (0%)	72 (90.0%)
Serratia marcescens	1,801	0 (0%)	1,788 (99.3%)	1,788 (99.3%)

Table S7. Bacterial strains, *Caenorhabditis elegans* strains and primers used in this study.

**Bacterial strains used in this study**	
**Strains**	**Description**
BMB171	acrystalliferous mutant of Bt strain
BMB171/Cry5Ba	BMB171 containing plasmid pBMB0215-Cry5Ba2
Δ*plcR*/BMB171	*plcR* knockout mutant of BMB171
Δ*pisP*/BMB171	*pisP* knockout mutant of BMB171
Δ*luxS*/BMB171	*luxS* knockout mutant of BMB171
Δ*nprR*/BMB171	*nprR* knockout mutant of BMB171
Δ*papR*/BMB171	*papR knockout mutant of BMB171*
Δ*plcRpisP*/BMB171	*plcR* / *pisP* double knockout mutant of BMB171
plcR-flag/ΔplcR	Δ*plcR*/BMB171 containing plasmid pHT304- *plcR::flag*
OE- *plcR/* BMB171	BMB171 overexpressing *plcR*
OE-*pisP/* BMB171	BMB171 overexpressing *pisP*
P*pisP*-lacZ/ BMB171	BMB171 containing a recombinant plasmid with the lacZ gene driven by the *pisP* promoter.
P*pisP*-lacZ/ BMB171Δ*plcR*	Δ*plcR*/BMB171 containing a recombinant plasmid with the lacZ gene driven by

	the *pisP* promoter.
BL21(DE3)	F- ompT rb- mb- DE3
Cry5Ba/ BL21	Expressing *Cry5Ba2* in BL21
pET28a-*plcR*/BL21	Expressing *plcR* in BL21
P*pisP*-GFP/ BMB171	Co-expressing *pisP* and *gfp* in BMB171
P*pisP*-GFP/ BMB171Δ*plcR*	Co-expressing *pisP* and *gfp* in Δ*plcR*/BMB171
DH5α	F- φ80lacZΔM15 Δ(lacZYA-argF) U169 deoR recA1 endA1 hsdR17 (rk-,mk+) phoA supE44 λ- thi-1 gyrA96 relA1
OP50	Feeding *C.elegans*

The information of aminopeptidase mutants of *C. elegans* used in this research

**Mutant Gene**	**Strain**	**Genotype**
daf-16::gfp	TJ356	*daf-16*(mu86) I
lys-7::gfp	SAL105	*pha-1*(e2123) III denEx2

daf-16	CF1038	*daf-16*(mu86) I

**Primers (qPCR primers)**	**Primer sequences (5′–3′)**
tba-1-F	TCAACATGCCATCGCCGCC
tba-1-R	TCCAAGCGAGCCAGGCTTCAG
F25B5.1-F	AGAGATGGCACAGAGTTA
F25B5.1-R	AACGAACCTTCAGCATAC
F26A1.9-F	ACGATGGTGTCAGAAGTA
F26A1.9-R	GAAGAACTGTTGTTGTTGTC
C53B4.3-F	TGTTGTTCGCATTCTTCA
C53B4.3-R	TTCTGTATTGGTAGCATTGA
R08D7.7-F	AAGGATACGGAAGTGAGT
R08D7.7-R	GGATGCGAAGAATGATGA
Y82E9BL.7-F	ATGCTACAAGACCGATATG
Y82E9BL.7-R	TTGAAGTGTGGAAGAATAGAA
C18E9.7-F	CTACGAGGTCTTGATTCTG
C18E9.7-R	CCTGTTCCTTGAGTTGTAA

T20D4.10-F	ATTATTGGCACTGTGTTCT
T20D4.10-R	AATTATTGAGTTGCTTGAAGAT
col-93-F	CTACTCGGCTGTTACCTT
col-93-R	GTGCTTGCAGAACTTGAT
cpg-22-F	CATCATCATCTTCATCATCTTC
cpg-22-R	GGTTCTTCACTCACTGTT
F21C10.7-F	AGCGATGTTGATGTTAGTT
F21C10.7-R	AGTCTTGATGTTGATGTTGA
cutl-15-F	CTTGTTGGTGGTGAAGAA
cutl-15-R	CAGTTGGAAGTTGTGGAT
nas-8 -F	CGAGACGAACATATCACAA
nas-8 -R	CATAGGTATTAGGTAAGCAAGT
AC3.5 -F	TCAATAAGGCTATAAGAGTAAGG
AC3.5 -R	TTCCAGAGACCATCATCA
T17H7.7 -F	ACAACACCTTCAACAACA
T17H7.7 -R	CAACGGAGTAGTCTTCTTC
thn-2-F	GAGGCTTCCAACTTACGGCT

thn-2-R	CAAGGGATGCTGGTGGAACT
lys-7-F	AGGTTCCCCCGATTGTTGAC
lys-7-R	GCTGGGTTGTATGCACGAAC
mtl-1--F	AAACAAGCAATGCAAGTG
mtl-1--R	CAGTCTCCCTTACATCCA
spp-2-F	CTGTGAAGACTTATGATGGAT
spp-2-R	TGTGGAGCAAATGGAATC
daf-16-F	AGCAACCATTCCCCATCACC
daf-16-R	ATTGAGCTCCGCCTCCAATG
pisP-qRT-F	GTAGTTGCTGTGCGGATT
pisP-qRT-R	CTGTGGTCGTGAAGTGTT
mogR-qRT-F	CGGTGTAGTGAAAGGAAAGAAGAC
mogR-qRT-R	AGTTGTAACGACTGCAATTCATCT
RPL18-SF-qRT-F	TCAAGTCTCAGGATGTGTA
RPL18-SF-qRT-R	AACAGCCTACGAAGAATG
cyp-35A2-SF-qRT-F	TTAATATCTCTATCGTGAACATACT
cyp-35A2-SF-qRT-R	CCTCATTATCAAGTCGCATA

XP_050561655.1-SF-F	CTGAGGATGTGAAGATAGGT
XP_050561655.1-SF-R	TGTAGGCGTATGGATGAC
hsp-12.3-SF-qRT-F	AGACAAGTTCCAAATCAA
hsp-12.3-SF-qRT-R	GTTCATCCTTCTTCTCTT
nnt-1-SF-qRT-F	TACCAGCAGTAGTTAGCA
nnt-1-SF-qRT-R	AGAATTATATCAGATTGGAATGTG
SF-gloverin1-F	AGAGGCAGTGGTATGTCCCT
SF-gloverin1-R	TGAGCACCCAATTCAGGCTT
SF-cecropin-F	GAGAAGTTGGGCCAAAACATCCG
SF-cecropin-R	TCACTTTCCTATGGCTGCTGCT
SF-moricin-F	ATCGTGGTGGTTGCACTTCT
SF-moricin-R	GACCTTTGCCAATCGCTGTG
SF-lebocin2-F	CCGGTGCTGCCATCTATCAT
SF-lebocin2-R	TACCTCCTACATGCCCGTGA
SF-gallerimycin-F	ACGATCAGAGGACCGGAGTT
SF-gallerimycin-R	TCGCAGACATTGGCATCCAT
SF-spodoptericin-F	AAGCCGAATCCAAGGGACAG

SF-spodoptericin-R	TTCCTCGAAATCGCACGACA
SF-lepidoptin-F	ACGTGATCCATACAGTGCGG
SF-lepidoptin-R	GGGATGGTGACACACACGTA
BMB171_RS07690-F	TACATACATTGAAGGAATTG
BMB171_RS07690-R	ATGCGATTATAGCCATAA
BMB171_RS16655-F	CGAATTAGTATGGAAGGAA
BMB171_RS16655-R	TTAGCCGCAATTAAGTAA
BMB171_RS01800-F	AAGCAACGGAAGAAGAACTAA
BMB171_R01800-R	TGTCATATTCACGCCATACTC
BMB171_RS21640-F	TGCTCGCCATCACTGCTTA
BMB171_R21640-R	TAGTCCGCTTTCTGGTTCTGT
BMB171_RS07425-F	AGCGTTATGCCGATTCCTACA
BMB171_RS07425-R	CGACCAGACTGTTGCTCCAT
BMB171_RS09300-F	AATGAGGTAGATGGATTATTAGGAA
BMB171_RS09300-R	TACTTGCCAATATCGCTGTTA
BMB171_RS11935-F	GAAGGATGTGATGAGGATG
BMB171_RS11935-R	GAATACTCGTTACCGCATAT

BMB171_RS06420-F	AGAACGTGGTCTTTATGCGAAA
BMB171_RS06420-R	GCTCCAGCCAATAGTGAAGTT
**Gene knockout primers**	
sgRNA-pisP-F	AAAC CTCTCATGACCTTTGATCAT
sgRNA-pisP-R	AAGGCCAACGAGGCC AATAGCATTCATCGCTATTAA
pisP-up-F	AAGGCCATGTTGGCC TATATACCTCCAATAGAGAAATT
pisP-up-R	AAGGCCAACATGGCC TTGGCTTTTTTTAATCGTCC
pisP-down-F	AAGGCCTTATTGGCC TCAAGTAAATCCAAAGAACC
pisP-down-R	AAGGCCTTATTGGCC TCAAGTAAATCCAAAGAACC
plcR-verify-F	TTTATATGTTTGTGCAAGG
plcR-verify-R	ACATTAAAAATCGCTCGA
papR-verify-F	ATAGACGTATACCAAAATC
papR-verify-R	CAATTTAACCATGCAGAT
pisp-verify-F	GGAACAAAACTATTTATCTCT
pisp-verify-R	GATGATGTTTTCTCGGAT
PapR-up-F	ACGCGTCGACAATCAACAGTTAGCTGGAA
PapR-up-R	ACGCGTCGACATTCCATCACCCACATTT

PapR-down-F	ACGCGTCGACAAAACAACCGTCTTACTT
PapR-down-R	ACGCGTCGACTGTTGTTCAATTTAACCATG
PapR-sgRNA-F	TACGTATTAACGTTAGCGATGGCA
PapR-sgRNA-R	AAACTGCCATCGCTAACGTTAATA
NprR-up-F	ACGCGTCGACGAGCGTGTGTGCCAGCTAC
NprR-up-R	GGCATGCTGTCCTCCTCCATTCTCATT
NprR-down-F	GGCATGCACATGAAAAAGATGGTAT
NprR-down-R	CGGGGTACCCCTCATATTTCGGTGTTAC
Spc-F	GGGCATGCAGCAGCAGATTACGC
Spc-R	GCGCATGCCAATTCCGACAGGCTTTG
**ChIP-Seq primers**	
PplcR-PlcR-F	GCGCTGGTACCTCTACTGATTTTATTTACGA
PplcR-PlcR-R	CGCGGATCCATATCCTAAAAAATGGGTAT
TerplcR-F	AAAACTGCAGCACATTAAAAATCGCTCGAG
TerplcR-R	CAAGTCATGAAACAGAAGT
**ChIP-PCR primers**	
inhA-ChIP-F	GATGCTCACTCACAAGAA

inhA-ChIP-R	TTGCTCAAGGCTAACATT
mogR-ChIP-F	CGGTGTAGTGAAAGGAAAGAAGAC
mogR-ChIP-R	AGTTGTAACGACTGCAATTCATCT
pisP-ChIP-F	GGACAGGGAGTAAGGACACAA
pisP-ChIP-R	CCGCACAGCAACTACTAACG
BMB171_RS08810-F	TCCTTGTAGTGTGAATGTATT
BMB171_RS08810-R	AGTTGATAATCGTGAGAAGTAT
nheA-ChIP-F	CATTCTTCATAATCAATCCAG
nheA-ChIP-R	TATCTACATATCACAACAGAAA
BMB171_RS12715-F	TTAGTAAGTATGAAGATATGACG
BMB171_RS12715-R	ATGCACCTTGTTCATATAC
**lacZ primers**	
P*pisP*-lacZ-F	CGGGATCC ATCACGAGTAAGAAATCAAG
P*pisP*-lacZ-R	CGGGATCC ATCACGAGTAAGAAATCAAG
**protein expression primers**	
PlcR-F	CCGCTCGAGTTATCTACTGATTTTATTTA
PlcR-R	TGCTCTAGA CAAGTCATGAAACAGAAGT

pisP-21b-F	TGGTGATGGTGATGCTCGAG TGGTCGATTAAACATTTTTA
pisP-21b-R	TACG ATGATCAAAGGTCATGAGAG
**EMSA primers**	
pisP-EMSA-F	ATCACGAGTAAGAAATCAAG
pisP-EMSA-R	GGGAATTCCATATGATGCACGCAGAAAAATTAGG
**Co-expression of fluorescent fusion protein primers**	
Fus-gfp-F	GGAGGTTCAGGTGGAGGCGGTTCAGGCGGAGGTGGC ATGGTGAGCAAGGGCG
Fus-gfp-R	CCCAAGCTT TTACTTGTACAGCTCGT
Fus-pisP-F	GCCACCTCCGCCTGAACCGCCTCCACCTGAACCTCC TGGTCGATTAAACATTTT
Fus-pisP-R	TTAGCTTCTTGGGGTCTAGA ATAAAGATAATTTGGCCATAC
**Gene overexpression primers**	
pisP-PHT315-F	ACCATGATTACGCCAAGCTT CAAAACTTATATTTATTACT
pisP-PHT315-R	TTAGCTTCTTGGGGTCTAGA CTGTTGATAAAGGGCAAAG
PlcR-HR-PHT315-F	ACCATGATTACGCCAAGCTT ATTATATGCAATTATGCATACC

PlcR-HR-PHT315-R	AAGAAGGAGATATACATATG ATGGCATATCCATTTCGTAA
**Yeast two-hybrid primers**	
PisP-BD-NdeI-F	GGATCTCAGAGGAGGACCTGCATATGATGGCATATCCATTTCGTAA
PisP-BD-EcoRI-R	CAGGTCGACGGATCCCCGGGAATTCTTATGGTCGATTAAACATTTT
DAF-16-AD-NdeI-F	ACGACGTACCAGATTACGCTCATATG ATGCAACTCGagCAAAAGTC
DAF-16-AD-XhoI-R	TCTACGATTCATCTGCAGCTCGAG TTACAAATCAAAATGAATATG
AKT-1-AD- NdeI-F	ACGACGTACCAGATTACGCTCATATG ATGTCGATGACAAGCTTGTC
AKT-1-AD-XhoI-R	TCTACGATTCATCTGCAGCTCGAG TTATCCCATATCATAATCTTCG
INS-1-AD- NdeI-F	ACGACGTACCAGATTACGCTCATATG ATGTACTGGTTTCGTCAAGT
INS-1-AD-XhoI-R	TCTACGATTCATCTGCAGCTCGAG TCAATTATCGTCCTGATTGC
INS-18-AD- NdeI-F	ACGACGTACCAGATTACGCTCATATG ATGGTCCACCGACTTTTCAT
INS-18-AD-XhoI-R	TCTACGATTCATCTGCAGCTCGAG TTAAATTGGGGCACAGTAGG
INS-39-AD- NdeI-F	ACGACGTACCAGATTACGCTCATATG ATGAACACCTTCTTCTTTCT
INS-39-AD-XhoI-R	TCTACGATTCATCTGCAGCTCGAG TCAGAGCAGGGTGACGAAGT
AGE-1 -AD- NdeI-F	ACGACGTACCAGATTACGCTCATATG ATGTCTATGGGACGAAGCCC
AGE-1 -AD-XhoI-R	TCTACGATTCATCTGCAGCTCGAG TCAGTAGTGTTTGACTGCGT
PAR-1 -AD- NdeI-F	ACGACGTACCAGATTACGCTCATATG ATGTTCAATGCGGCATACTC

PAR-1 -AD-XhoI-R	TCTACGATTCATCTGCAGCTCGAG CTATTGGTTGTTGCCGAACG
SEK-1 -AD- NdeI-F	ACGACGTACCAGATTACGCTCATATG ATGGAGCGAAAAGGACGTGA
SEK-1 -AD-XhoI-R	TCTACGATTCATCTGCAGCTCGAG TCATCGTCGCCAAACAGTGT
PDK-1-AD- NdeI-F	ACGACGTACCAGATTACGCTCATATG ATGGAGGATCTCACACCAAC
PDK-1-AD-XhoI-R	TCTACGATTCATCTGCAGCTCGAG TCAAGGCGACTTCTTGTCCA

Table S8. Experimentally reported target orders and representative pest species associated with pesticidal proteins based on published literature and the BPPRC database.

Pesticidal protein class	Toxin type	Previous name	Reference Pesticidal protein	Experimentally reported target order	Representative target pest species	References
App4A	Monomeric toxin	Cry78Ba	App4Aa1	Lepidoptera; Hemiptera	*Plutella xylostella; Laodelphax striatellus*	10.3390/microorganisms12122450; US patent 8461421
App6A	Monomeric toxin	Cry6Aa	App6Aa2; App6Aa3	Rhabditida	*Caenorhabditis elegans; Bursaphelenchus xylophilus; Meloidogyne incognita*	10.1016/j.pestbp.2024.106282; 10.1016/j.pt.2025.01.004
App6B	Monomeric toxin	Cry6B = CryVIB	App6Ba1	Rhabditida; Coleoptera	*Caenorhabditis elegans; Bursaphelenchus xylophilus; Meloidogyne incognita; Hypera postica*	10.1016/j.jip.2022.107726; 10.1007/s00284-010-9749-4
cry10A	Monomeric toxin		Cry10Aa4	Diptera; Coleoptera	*Aedes aegypti; cotton boll weevil*	10.1128/AEM.00409-09; 10.1016/j.plantsci.2024.112079
cry11A	Monomeric toxin	CryD;CryIVD; CryXI	Cry11Aa3	Diptera	*Aedes aegypti; Anopheles albimanus; Culex quinquefasciatu; Culex*	10.3390/toxins17090451; 10.1128/AEM.64.11.4174-4179.1998

		A(a)			*pipiens*	
Cry11B	Monomeric toxin		Cry11Ba1; Cry11Bb1	Diptera	*Aedes aegypti; Anopheles albimanus; Culex quinquefasciatus*	10.1016/s0167-4838(98)00168-x
cry12A	Monomeric toxin		Cry12Aa1	Rhabditida	*Caenorhabditis elegans; Root-knot nematode; Meloidogyne incognita; Soybean cyst nematode; Heterodera glycines; Bursaphelenchus xylophilus*	10.1073/pnas.0538072100; 10.1016/j.pt.2025.01.004; 10.1016/j.jip.2022.107726
Cry13A	Monomeric toxin		Cry13Aa2	Rhabditida	*Bursaphelenchus xylophilus*	10.1016/j.jip.2022.107726; 10.1016/j.pt.2025.01.004
Cry14A	Monomeric toxin		Cry14Aa1	Rhabditida	*Heterodera glycines; Caenorhabditis elegans; Haemonchus contortus*	10.1371/journal.ppat.1012611; 10.1016/j.pt.2025.01.004
Cry19A	Monomeric toxin		Cry19Aa1	Diptera	*Anopheles stephensi; Culex pipiens;*	10.1111/j.1574-6968.2009.01583.x; 10.1128/AEM.70.6.3769-3771.2004
cry19B	Monomeric toxin		Cry19Ba1	Diptera	*Culex pipiens molestus*	10.1016/s0723-2020(98)80022-2
cry1A	Monomeric toxin		Cry1Aa17; Cry1Aa3; Cry1Aa45; Cry1Aa9; Cry1Ab21;	Lepidoptera	*Plutella xylostella; Ostrinia furnacalis; Helicoverpa armigera; Leucinodes orbonalis; Trichoplusia ni; Hyphantria cunea;*	10.1093/femsre/fuad026; 10.1016/j.jip.2006.01.004

			Cry1Ab24; Cry1Ab28; Cry1Ab35; Cry1Ab37; Cry1Ab9; Cry1Ac11; Cry1Ac2; Cry1Ac20; Cry1Ac4; Cry1Ac5; Cry1Ac9; Cry1Ad2; Cry1Ae1; Cry1Ah1		*Spodoptera frugiperda; Pieris rapae; Chilo suppressalis*	
cry1B	Monomeric toxin		Cry1Ba4; Cry1Ba8; Cry1Bb1; Cry1Bd1; Cry1Bd2; Cry1Be6; Cry1Bk1	Lepidoptera; Hemiptera ; Diptera; Coleoptera	*Plutella xylostella; Ostrinia nubilalis; Spodoptera frugiperda; Maruca vitrata; Manduca sexta; cotton boll weevil*	10.1016/j.plantsci.2024.112079; 10.1093/femsre/fuad026; 10.3390/toxins11030173; 10.1006/jipa.1997.4680; 10.1006/jipa.2000.4962
cry1C	Monomeric toxin		Cry1Ca18; Cry1Cb2	Lepidoptera; Dipteran	*Plutella xylostella; Myzus persicae; Aedes aegypti*	10.1007/s00284-002-3982-4; 10.3390/insects16111085
cry1D	Monomeric toxin		Cry1Da2; Cry1Da3;	Lepidoptera	*Ostrinia nubilalis; Plutella xylostella; Spodoptera*	10.1093/jee/97.6.1805

			Cry1Db1; Cry1Db2		*exigua; Spodoptera frugiperda; Bombyx mori; Spodoptera litura; Epiphyas postvittana*	
cry1E	Monomeric toxin		Cry1Ea12; Cry1Ea7	Lepidoptera; Hemiptera; Rhabditida	*Spodoptera exigua; Spodoptera frugiperda; Malacosoma disstria; Bursaphelenchus xylophilus*	10.1006/jipa.1993.1116; 10.1016/j.jip.2024.108122; 10.1094/PHYTO-05-17-0179-R
cry1F	Monomeric toxin		Cry1Fa2; Cry1Fb4; Cry1Fb5	Lepidoptera; Coleoptera	*Agrotis ipsilon; Spodoptera frugiperda; Diabrotica virgifera virgifera*	10.1016/j.jip.2009.07.012; 10.1093/jee/tox146; WO2016109214A1
cry1G	Monomeric toxin		Cry1Ga1; Cry1Gb2	Lepidoptera	*Pieris rapae; Thaumetopoea pityocampa; Plutella xylostella; Myzus persicae*	10.1007/s002849910046
cry1H	Monomeric toxin		Cry1Hb1; Cry1Hb2	Lepidoptera	*Spodoptera exigua; Ostrinia furnacalis*	10.1128/aem.61.6.2086-2092.1995; 10.1007/s13213-015-1058-5
cry1I	Monomeric toxin	CryV / CryV1	Cry1Ia37; Cry1Ia42; Cry1Ia43; Cry1Ia7; Cry1Ib1; Cry1Ib3; Cry1Ib5; Cry1Ib6; Cry1Id2;	Coleoptera; Lepidoptera; Rhabditida	*Leptinotarsa decemlineata; Ostrinia furnacalis; Lobesia botrana; Plutella xylostella; Earias insulana; cotton boll weevil; Meloidogyne incognita*	10.1128/AEM.02861-05; 10.1016/j.plantsci.2024.112079;10.1016/j.pestbp.2025.106419

			Cry1Ie3			
cry1J	Monomeric toxin		Cry1Ja1; Cry1Ja2	Lepidoptera; Coleoptera	*Plutella xylostella; Manduca sexta; Lobesia botrana; Diabrotica undecimpunctata howardi*	10.1128/AEM.67.12.5729-5734.2001; US5356623A
cry1K	Monomeric toxin		Cry1Ka1; Cry1Ka2	Lepidoptera	*Artogeia rapae; Pieris rapae*	10.1111/j.1574-6968.1995.tb07931.x; 10.1111/j.1574-6968.1995.tb07931.x
cry1M	Monomeric toxin		Cry1Ma1; Cry1Ma2	Lepidoptera	*corn earworm (Helicoverpa zea); black cut worm (Agrotis ipsilon); cabbage looper (Trichoplusia ni); saltmarch caterpillar (Estigmene acrea); tobacco budworm (Heliothis virescens); and lettuce armyworm (Pseudaletia unipuncta).*	US20090098051A1
cry1N	Monomeric toxin		Cry1Na1; Cry1Na2; Cry1Na3; Cry1Nb1	Lepidoptera; Coleoptera	*Plutella xylostella; Ostrinia furnacalis; Colaphellus bowringi*	10.1093/jee/tow031
Cry21A	Monomeric toxin		Cry21Aa2; Cry21Aa3	Rhabditida	*Heligmosomoides polygyrus bakeri; Caenorhabditis elegans*	10.1007/s10158-012-0135-8; 10.1073/pnas.0538072100

Cry21C	Monomeric toxin		Cry21Ca2	Rhabditida	*Heligmosomoides polygyrus bakeri; Caenorhabditis elegans*	10.1007/s10158-012-0135-8
Cry21D	Monomeric toxin		Cry21Da1	Rhabditida	*Heligmosomoides polygyrus bakeri; Caenorhabditis elegans*	10.1007/s10158-012-0135-8
Cry21E	Monomeric toxin		Cry21Ea1	Rhabditida	*Heligmosomoides polygyrus bakeri; Caenorhabditis elegans*	10.1007/s10158-012-0135-8
Cry21F	Monomeric toxin		Cry21Fa1	Rhabditida	*Heligmosomoides polygyrus bakeri; Caenorhabditis elegans*	10.1007/s10158-012-0135-8
Cry21G	Monomeric toxin		Cry21Ga1	Rhabditida	*Heligmosomoides polygyrus bakeri; Caenorhabditis elegans*	10.1007/s10158-012-0135-8
Cry21H	Monomeric toxin		Cry21Ha1	Rhabditida	*Heligmosomoides polygyrus bakeri; Caenorhabditis elegans*	10.1007/s10158-012-0135-8
cry24A	Monomeric toxin		Cry24Aa1	Diptera	*Aedes aegypti; Culex quinquefasciatus; Anopheles albimanus*	10.1128/AEM.00078-13
cry25A	Monomeric toxin		Cry25Aa1	Diptera		10.1128/AEM.00078-13
cry27A	Monomeric toxin		Cry27Aa1	Diptera	*Anopheles stephensi; Aedes aegypti; Culex pipiens*	10.1016/S0723-2020(00)80042-9

					molestus	
cry29A+Cry11B	Binary toxin		Cry29Aa1+Cry11Bb	Diptera	*Aedes aegypti*	10.1078/072320203770865783
cry2A	Monomeric toxin		Cry2Aa22; Cry2Aa8; Cry2Ab15; Cry2Ab4; Cry2Ab41; Cry2Ac6; Cry2Ad1; Cry2Ah3	Lepidoptera; Diptera; Hemiptera	*Helicoverpa armigera;Aedes aegypti; Diaphorina citri*	10.1016/j.jip.2014.06.005; 10.1099/13500872-140-8-1869; 10.1111/j.1570-7458.1995.tb02003.x; 10.3390/toxins12120773
cry2B	Monomeric toxin	CryIIB	Cry2Ba1	Lepidoptera; Diptera	*Manduca sexta; Ostrinia nubilalis;Aedes aegypti*	10.1128/jb.172.6.2826-2832.1990; 10.3390/toxins12060355
cry30G	Monomeric toxin		Cry30Ga2	Diptera; Lepidoptera	*Aedes aegypti; Lepidoptera*	10.1016/j.jip.2009.12.004
Cry3A	Monomeric toxin		Cry3Aa6	Coleoptera; Hemiptera	*Leptinotarsa decemlineata; Tenebrio molitor; Hypera postica; Macrosiphum euphorbiae*	10.1021/bp000150d; 10.3390/toxins12070430; 10.1111/j.1570-7458.1995.tb02003.x; 10.3390/microorganisms13020371
cry40D	Monomeric toxin		Cry40Da1	Diptera	*Culex quinquefasciatus; Bradysia odoriphaga*	CN200910119992.9
cry4A	Monomeric toxin		Cry4Aa5	Diptera; Hemiptera	*Culex pipiens; Culex quinquefasciatus; Aedes aegypti; Acyrthosiphon pisum*	10.1128/JB.188.9.3391-3401.2006; 10.1016/0378-1097(92)90584-b; 10.1128/AEM.00686-09

cry4B	Monomeric toxin		Cry4Ba6; Cry4Ba7	Diptera; Hemiptera	*Anopheles gambiae; Diaphorina citri*	10.1111/1744-7917.12654; 10.1128/JB.188.9.3391-3401.2006
cry4C	Monomeric toxin		Cry4Ca1; Cry4Ca2	Diptera	*Aedes aegypti*	10.1016/j.jip.2009.12.004
cry50B	Monomeric toxin		Cry50Ba2	Diptera	*Culex quinquefasciatus*	10.1016/j.toxicon.2017.08.025
cry53A	Monomeric toxin		Cry53Ab1	Lepidoptera	*Spodoptera frugiperda*	CN202110389006.2
cry54A	Monomeric toxin		Cry54Aa2	Lepidoptera; Diptera	*Laphygma exigua; Aedes aegypti; Helicoverpa armigera*	10.1007/s00284-009-9386-y
cry54B	Monomeric toxin		Cry54Ba2	Diptera	*Culex quinquefasciatus*	10.1016/j.jip.2011.11.003
cry56A	Monomeric toxin		Cry56Aa4	Lepidoptera; Diptera	*Aedes aegypti; Aedes albopictus; Plutella xylostella; Helicoverpa armigera; Spodoptera frugiperda*	10.1007/s10529-009-0147-6
cry5A	Monomeric toxin		Cry5Aa1; Cry5Ac1; Cry5Ad1	Lepidoptera; Hymenoptera; Rhabditida	*Caenorhabditis elegans; Haemonchus contortus; Trichostrongylus colubriformis; Ostertagia circumcincta; Diprion pini; Cephacia abietis; Helicoverpa armigera*	10.1007/s10930-009-9169-0; 10.1038/s41598-021-82495-8; 10.1016/s0965-1748(01)00030-3

cry5B	Monomeric toxin		Cry5Ba1; Cry5Ba2	Rhabditida	*Caenorhabditis elegans; Ancylostoma ceylanicum; Heligmosomoides polygyrus bakeri; Meloidogyne incognita*	10.1128/AEM.01048-12; 10.1073/pnas.0538072100
cry5C	Monomeric toxin		Cry5Ca2	Rhabditida	*Meloidogyne incognita*	10.1128/AEM.03505-16
cry5D	Monomeric toxin		Cry5Da2	Rhabditida	*Meloidogyne incognita; Caenorhabditis elegans*	10.1128/AEM.03505-16
cry65A	Monomeric toxin		Cry65Aa2	cells	*MDA-MB-231 cells*	10.1038/srep08291
cry69A	Monomeric toxin		Cry69Ab1	Diptera	*Culex quinquefasciatus*	10.1007/s11274-013-1548-1
cry70A	Monomeric toxin		Cry70Aa1	Diptera	*Aedes mosquitoes*	CN102173942A
cry71A	Monomeric toxin		Cry71Aa1	Lepidoptera	*Spodoptera exigua; cotton bollworm; beet armyworm; and diamondback armyworm.*	10.1038/s41598-021-96133-w; CN103525835B
cry72A	Monomeric toxin		Cry72Aa1; Cry72Aa2	Lepidoptera	*cotton bollworm; beet armyworm; and diamondback armyworm.*	10.1038/s41598-021-96133-w
cry73B	Monomeric toxin		Cry73Ba2	Hemiptera	*Myzus persicae*	10.1016/j.jip.2022.107731; 10.3390/toxins6113144
cry7A	Monomeric toxin	CryIIIC	Cry7Aa2; Cry7Ab4;	Coleoptera	*Leptinotarsa decemlineata; Henosepilachna*	10.3390/toxins12070430; 10.1603/EC09432

			Cry7Ab6; Cry7Ab8		*vigintiomaculata; Acanthoscelides obtectus; Henosepilachna vigintioctomaculata; Cylas puncticollis*	
cry7B	Monomeric toxin		Cry7Ba1	Lepidoptera	*Plutella xylostella*	10.1111/j.1462-2920.2011.02557.x
cry7C	Monomeric toxin		Cry7Cb1	Orthoptera	*Locusta migratoria manilensis*	10.1128/AEM.02462-10
cry7G	Monomeric toxin		Cry7Ga2; Cry7Gc1; Cry7Gd1	Coleoptera	*Diabrotica undecimpunctata howardi; Diabrotica virgifera virgifera;*	US7803925B2
cry8A	Monomeric toxin		Cry8Ac1	Coleoptera	*Leptinotarsa decemlineata; Diabrotica spp.; Cotinis spp; Holotrichia oblita; Holotrichia parallela*	10.3390/toxins12070430; 10.1016/j.micres.2013.03.003
cry8B	Monomeric toxin		Cry8Ba2	Coleoptera	*Anthonomus grandis; Chrysomela scripta; Xanthogaleruca luteola*	10.3390/toxins12070430; 10.1371/journal.pone.0294654
cry8C	Monomeric toxin		Cry8Ca1; Cry8Ca5	Coleoptera	*Holotrichia parallela; Anomala cuprea; Anomala rufocuprea; Popillia japonica; Alphitobius diaperinus ; Anomala corpulenta; Anomala exoleta;*	10.3390/toxins12070430; 10.1016/j.jip.2008.05.009

					Colaphellus bowringi	
cry8E	Monomeric toxin		Cry8Ea5	Coleoptera	*Holotrichia parallela*	10.3390/toxins12070430; 10.1016/j.jsb.2009.07.004
cry8F	Monomeric toxin		Cry8Fa1	Coleoptera	*Holotrichia parallela; Anomala corpulenta; H. oblita; Phyllotreta striolata*	10.3390/toxins18040191; 10.3390/toxins11080440
cry8G	Monomeric toxin		Cry8Ga3	Coleoptera	*Phyllotreta striolata; Holotrichia parallela; Holotrichia oblita; Anomala corpulenta;*	10.1007/s00253-009-1971-2; 10.3390/toxins18040191
cry8H	Monomeric toxin		Cry8Ha1	Coleoptera	*Holotrichia parallela; Holotrichia oblita ; Anoplophora glabripennis*	10.3390/toxins12100642
cry8I	Monomeric toxin		Cry8Ia1; Cry8Ia4; Cry8Ib2	Coleoptera	*Holotrichia parallela*	10.3390/toxins12100642
cry8K	Monomeric toxin		Cry8Kb3	Coleoptera	*Anthonomus grandis; cotton boll weevil*	10.1016/j.plantsci.2024.112079; 10.3390/toxins12070430
cry8Q	Monomeric toxin		Cry8Qa2	Coleoptera; Lepidoptera	*Anthonomus grandis; Anticarsia gemmatalis*	10.1016/j.jip.2021.107563; 10.1159/000365929
cry8S	Monomeric toxin		Cry8Sa1	Coleoptera	*Holotrichia serrata*	10.3390/toxins12070430
Cry9A	Monomeric toxin		Cry9Aa4; Cry9Aa6	Lepidoptera	*Plutella xylostella; Pieris brassicae; Phthorimaea operculella; Chilo*	10.1021/acs.jafc.3c08070; 10.1074/jbc.RA118.003490

					suppressalis	
Cry9B	Monomeric toxin		Cry9Ba1	Lepidoptera	*Manduca sexta; Anticarsia gemmatalis*	10.1016/j.jip.2008.03.012
Cry9D	Monomeric toxin		Cry9Da4	Coleoptera	*Bombyx mori; Hyphantria cunea*	10.1016/j.ibmb.2023.104030; 10.3390/ijms26083497
Cry9E	Monomeric toxin		Cry9Ea12; Cry9Eb3; Cry9Ee3	Lepidoptera	*Agrotis exclamationis; Cydia pomonella*	10.1002/ps.6157; 10.1371/journal.pone.0283077
cyt1A	Monomeric toxin		Cyt1Aa11; Cyt1Aa4; Cyt1Ab1	Diptera; Hemiptera; Coleoptera; Lepidoptera	*Aedes aegypti; Culex quinquefasciatus; Culex pipien; Diaphorina citri; Plutella xylostella*	10.1128/AEM.71.1.185-189.2005; 10.1111/1744-7917.12654; 10.1128/AEM.64.11.4368-4371.1998; 10.1128/AEM.67.12.5859-5861.2001
cyt2A	Monomeric toxin		Cyt2Aa4	Diptera	*Aedes aegypti; Culex quinquefasciatus*	10.1016/j.femsle.2005.08.038; 10.5483/bmbrep.2013.46.3.100
cyt2B	Monomeric toxin		Cyt2Ba17; Cyt2Bb1; Cyt2Bc1	Diptera	*Aedes aegypti; Anopheles stephensi; Anopheles gambiae; Culex quinquefasciatus; Culex pipiens; Bradysia difformis*	10.1128/AEM.67.7.3280-3284.2001; 10.1128/AEM.68.3.1228-1231.2002; 10.3969/j.issn.1673-4254.2017.06.06
Gpp34A+Tpp35A	Binary toxin	Cry34	Gpp34Aa1+Tpp35Aa1	Coleoptera; Lepidoptera	*Diabrotica virgifera virgifera; Diabrotica barberi; Sitotroga cerealella;*	10.1016/j.jspr.2010.01.003; 10.1093/jee/toad221; 10.1128/AEM.71.4.1765-1774.200

					Diabrotica undecimpunctata	5
Mpp23A+Xpp37A	Binary toxin	Cry23Aa1	Mpp23Aa1+Xpp37Aa1	Coleoptera	*Cylas puncticollis; Anthonomus grandis*	10.3390/toxins15010055; 10.1016/j.plantsci.2024.112079
Mpp2A	Monomeric toxin	Mtx2	Mpp2Aa4	Coleoptera; Diptera	*Cydia pomonella; Anthonomus grandis Culex quinquefasciatus; Aedes aegypti*	10.1074/jbc.271.24.14183; 10.1016/0378-1119(95)00836-5
Mpp51A	Monomeric toxin	Cry51Aa	Mpp51Aa1	Thysanoptera; Hemiptera; Coleoptera	*Frankliniella spp.; Diaphorina citri; Lygus hesperus ; Lygus lineolaris; Leptinotarsa decemlineata*	10.1016/j.jip.2022.107845; 10.1093/jee/toae312; 10.1021/acs.jafc.2c07244
Mpp5A	Monomeric toxin	Sip1A	Mpp5Aa1; Mpp5Ab1; Mpp5Ac1	Coleoptera	*Colaphellus bowringi; Leptinotarsa decemlineata*	10.1007/s00253-006-0332-7
Mpp60A	Monomeric toxin	Cry60A	Mpp60Aa1; Mpp60Aa3	Diptera	*Culex quinquefasciatus*	10.1128/AEM.00078-13
Mpp60B	Monomeric toxin	Cry60B	Mpp60Ba3	Diptera	*Culex quinquefasciatus*	10.1128/AEM.00078-13
Mpp64B+Mpp64C	Binary toxin	Cry64Ba	Mpp64Ba1+Mpp64C	Hemiptera	*Sogatella furcifera; Laodelphax striatellus*	10.1128/AEM.01996-17
Mpp64B+Mpp64C	Binary toxin	Cry64Ca	Mpp64Ca1	Hemiptera	*Sogatella furcifera; Laodelphax striatellus*	10.1128/AEM.01996-17
Mtx1A	Monomeric toxin	Mtx1	Mtx1Aa1	Diptera	*Culex quinquefasciatus; Aedes aegypti ; Aedes albopictus*	10.1007/s00284-004-4274-y; 10.1128/AEM.00654-07

Spp1A	Monomeric toxin		Spp1Aa1	Lepidoptera; Blattodea	*Spodoptera litura; Blattella germanica*	10.3390/microorganisms12122450; 10.1128/AEM.00021-07
Tpp1A+Tpp2A	Binary toxin	BinA	Tpp1Aa2+Tpp2Aa1	Diptera	*Culex quinquefasciatus; Anopheles stephensi; Culex pipiens; Aedes atropalpus; Anopheles stephensi; Anopheles gambiae*	10.1128/jb.175.2.510-518.1993; 10.3390/toxins12120773
Tpp1A+Tpp2A	Binary toxin	BinB2	Tpp1Aa2+Tpp2Aa1	Diptera	*Culex quinquefasciatus; Anopheles stephensi*	10.1128/jb.175.2.510-518.1993; 10.3390/toxins12120773
Tpp35A+Gpp34A	Binary toxin	Cry35A	Tpp35Aa4+Gpp34Aa	Coleoptera; Lepidoptera	*Diabrotica virgifera virgifera;Diabrotica undecimpunctata; Sitotroga cerealella*	10.1093/jee/toad221; 10.1016/j.jspr.2010.01.003; 10.1128/AEM.71.4.1765-1774.2005
Tpp35B+Gpp34B	Binary toxin	Cry35Ba3	Tpp35Ba3+Gpp34Ba1	Coleoptera	*Diabrotica virgifera virgifera; Diabrotica barberi*	10.1128/AEM.71.4.1765-1774.2005
Tpp78A	Monomeric toxin	Cry78Aa1	Tpp78Aa1	Hemiptera; Neuroptera	*Laodelphax striatellus; Bemisia tabaci; Diaphorina citri; Chrysoperla nipponensis*	10.1002/ps.7620; 10.1016/j.jip.2024.108122; 10.1038/s42003-022-03754-6; 10.1016/j.jip.2018.07.007
Tpp78B	Monomeric toxin	Cry78Ba1	Tpp78Bb1	Hemiptera	*Laodelphax striatellus; Bemisia tabaci; Diaphorina citri*	10.3390/microorganisms12122450; 10.1016/j.jip.2024.108122; 10.1021/acs.jafc.9b07429
Tpp80A	Monomeric toxin	Cry80Aa1	Tpp80Aa2; Tpp80Ab1; Tpp80Ad1	Diptera; Hemiptera	*Culex pipiens pallens; Culex quinquefasciatus; Aedes aegypti ; Anopheles gambiae;*	10.3390/toxins14120863; 10.1016/j.jip.2020.107386

					Laodelphax striatellus	
vip3Aa	Monomeric toxin		Vip3Aa13; Vip3Aa17; Vip3Aa51; Vip3Aa59; Vip3Aa79; Vip3Aa80;	Lepidoptera; Blattodea; Aedes aegypti	*Helicoverpa punctigera; Spodoptera frugiperda; Helicoverpa zea; Chilo partellus; Blattella germanica*	10.1016/j.jip.2020.107342; 10.1007/s13562-012-0148-8; 10.1128/MMBR.00060-15; 10.1515/biol-2020-0014
vip3Af	Monomeric toxin		Vip3Af3	Lepidoptera	*Agrotis ipsilon; Spodoptera frugiperda; Spodoptera exigua; Helicoverpa armigera; Lobesia botrana; Ostrinia nubilalis; Ostrinia furnacalis; Chilo suppressalis*	10.1590/S0100-204X2014000200001; 10.1128/MMBR.00060-15; 10.1002/ps.8402
vip3Ag	Monomeric toxin		Vip3Ag6	Lepidoptera	*Lobesia botrana; Spodoptera littoralis; Chrysodeixis chalcites ; Spodoptera exigua*	10.1128/MMBR.00060-15; 10.1016/j.biocontrol.2013.05.003
vip3Ah	Monomeric toxin		Vip3Ah2	Lepidoptera	*Agrotis ipsilon; Manduca sexta*	Trop Biomed 2016 Mar 1;33(1):45-54.
vip3B	Monomeric toxin		Vip3Ba2	Lepidoptera	*Agrotis ipsilon; Spodoptera frugiperda; Helicoverpa armigera; Spodoptera exigua; Chrysodeixis includens; Ostrinia nubilalis*	10.1038/s41598-017-11702-2
vip3C	Monomeric toxin		Vip3Ca2	Lepidoptera	*Chrysodeixis chalcites; Mamestra brassicae;*	10.1128/aem.00253-25; 10.1016/j.jip.2016.10.001

					Trichoplusia ni; Spodoptera eridania; Helicoverpa zea; Chrysodeixis includens	
Vpb4D	Monomeric toxin	Vip4Da 2	Vpb4Da2	Coleoptera; Lepidoptera; Hemiptera; Diptera	*Diabrotica virgifera virgifera; Western Corn Rootwor; Helicoverpa zea; Lygus hesperus*	10.1371/journal.pone.0242791; WO2017019787A1; WO2017019787A1; 10.1371/journal.pone.0260532; US EPA. 2024. Environmental Risk Assessment for the Plant-Incorporated Protectants Brevibacillus laterosporus Mpp75Aa1.1 Protein and the Genetic Material Necessary for its Production (Vector PV-ZMIR525664); Bacillus thuringiensis Vpb4Da2 Protein and the Genetic Material Necessary for its Production (Vector V-ZMIR525664); dsRNA Transcript Comprising a DvSnf7.1 Inverted Repeat Sequence Derived from Diabrotica.聽 US Environmental Protection Agency. Washington, DC.聽 https://www.regulations.gov/docum

						ent/EPA-HQ-OPP-2023-0523-000
Xpp22B	Monomeric toxin	Cry22B	Xpp22Ba1; Xpp22Bb1	Coleoptera; Lepidoptera	*Anthonomus grandis; Plutella xylostella*	Patent US 6,541,448 B2; Patent US 8,461,421 B2
Xpp37A+Mpp23A	Binary toxin	Cry37A	Xpp37Aa1+ Mpp23Aa1	Hemiptera; Coleoptera	*Diaphorina citri; Anthonomus grandis; Cylas puncticollis*	10.1016/j.jip.2026.108720; 10.3390/insects13010027; 10.1016/j.plantsci.2024.112079
Xpp55A	Monomeric toxin	Cry55A	Xpp55Aa2	Rhabditida; Coleoptera	*Bursaphelenchus xylophilus; Meloidogyne incognita; Meloidogyne javanica; Phyllotreta cruciferae*	10.1016/j.jip.2022.107726;10.1128/AEM.01346-08; 10.1111/j.1751-7915.2011.00295.x; Patent US5973231A
Gpp34B + Tpp35B	Binary toxin	Gpp34Ba1 + Tpp35Ba1	Gpp34Ba1 + Tpp35Ba1	Coleoptera	*Diabrotica virgifera virgifera*	10.1128/AEM.71.4.1765-1774.2005
Mpp15A	Monomeric toxin		Mpp15Aa1	Lepidoptera; Pieris rapae;Cydia pomonella	*Manduca sexta*	10.1128/AEM.00752-08
Mpp3A	Monomeric toxin		Mpp3Aa1	Diptera	*Aedes aegypti;Culex quinquefasciatus*	10.1128/aem.62.6.2174-2176.1996
Mpp93A	Monomeric toxin		Mpp93Aa1	Lepidoptera	*Manduca sexta*	10.1128/jb.174.2.549-557.1992
Vpa2Ae + Vpb1Ac	Binary toxin	Vpa2Ae3	Vpa2Ae3 + Vpb1Ac1	Hemiptera	*Aphis gossypii*	10.1111/j.1574-6968.2011.02409.x

Vpa2Ag + Vpb1Ad	Binary toxin	Vpa2Ag1	Vpa2Ag1 + Vpb1Ad1	Coleoptera	*Anomala corpulenta;Holotrichia oblita;Holotrichia parallela*	10.1007/s00253-014-5966-2;10.3390/toxins11080440
Vpa2Ah + Vpb1Ae	Binary toxin	Vpa2Ah1	Vpa2Ah1 + Vpb1Ae1	Hemiptera	*Aphis gossypii*	10.4014/jmb.1105.05030
Xpp22A	Monomeric toxin	Cry22A	Xpp22Aa3	Coleoptera	*Anthonomus grandis*	Patent US 6,541,448 B2
Xpp76Aa + Xpp77Aa	Binary toxin	Cry76A	Xpp76Aa1 + Xpp77Aa1	Coleoptera	*Diabrotica virgifera virgifera*	US Patent US 10,889,830 B2
